# Comparison of protein expression during wild-type, and E1B-55k-deletion, adenovirus infection using quantitative time-course proteomics

**DOI:** 10.1099/jgv.0.000781

**Published:** 2017-06-20

**Authors:** Yen Rong Fu, Andrew S. Turnell, Simon Davis, Kate J. Heesom, Vanessa C. Evans, David A. Matthews

**Affiliations:** ^1^​ School of Cellular and Molecular Medicine, University Walk, University of Bristol, Bristol BS8 1TD, UK; ^2^​ Institute of Cancer and Genomic Sciences, College of Medical and Dental Sciences, University of Birmingham, Edgbaston, Birmingham B15 2TT, UK; ^3^​ Proteomics Facility, Faculty of Biomedical Sciences, University Walk, University of Bristol, Bristol BS8 1TD, UK

**Keywords:** adenovirus, d11520, E1B-55k, proteomics

## Abstract

Adenovirus has evolved strategies to usurp host-cell factors and machinery to facilitate its life cycle, including cell entry, replication, assembly and egress. Adenovirus continues, therefore, to be an important model system for investigating fundamental cellular processes. The role of adenovirus E1B-55k in targeting host-cell proteins that possess antiviral activity for proteasomal degradation is now well established. To expand our understanding of E1B-55k in regulating the levels of host-cell proteins, we performed comparative proteome analysis of wild-type, and E1B-55k-deletion, adenovirus-infected cancer cells. As such we performed quantitative MS/MS analysis to monitor protein expression changes affected by viral E1B-55k. We identified 5937 proteins, and of these, 69 and 58 proteins were down-regulated during wild-type and E1B-55k (*dl*1520) adenovirus infection, respectively. This analysis revealed that there are many, previously unidentified, cellular proteins subjected to degradation by adenovirus utilizing pathways independent of E1B-55k expression. Moreover, we found that ALCAM, EPHA2 and PTPRF, three cellular proteins that function in the regulation of cell–cell contacts, appeared to be degraded by E1B-55k/E4orf3 and/or E1B-55k/E4orf6 complexes. These molecules, like integrin α3 (a known substrate of E1B-55k/E4orf6), are critical regulators of cell signalling, cell adhesion and cell surface modulation, and their degradation during infection is, potentially, pertinent to adenovirus propagation. The data presented in this study illustrate the broad nature of protein down-regulation mediated by adenovirus.

## Abbreviations

Ad, adenovirus; DDR, DNA damage response; E1A, early region 1A; E1B, early region 1B; MRN, Mre11-Rad50-Nbs1 complex; MS/MS, tandem mass spectrometry; TMT, tandem mass tagging.

## Introduction

Adenoviruses are non-enveloped, icosahedral particles containing a linear double-stranded DNA (dsDNA) genome that is approximately 36 kbp in size. Adenovirus (Ad) was originally isolated from human adenoids in tissue culture in 1953 [1] and since then has been widely studied as a model viral system and as a powerful tool for uncovering fundamental cellular processes from cell-cycle control to splicing [2–4]. As part of its wide-ranging effects in the infected cell, adenovirus triggers virally induced degradation of cellular proteins to facilitate its own replication. Through early region 1A and 1B (E1A and E1B) and E4 proteins it has been known for some time that adenovirus induces the specific degradation of host-cell proteins to favour viral replication [5, 6]. A well-understood target is the cellular DNA damage response (DDR), principally the Mre11-Rad50-Nbs1 (MRN) complex, which would normally be triggered as the viral linear dsDNA genome builds up in the cell, mimicking extensive dsDNA breaks in the host-cell genome [7]. To counteract this, the adenoviral E1B-55k/E4orf6 proteins cooperate to redirect a cullin-containing ubiquitin ligase to specifically target a key component of the DDR known as MRE11. This consequently inhibits activation of the protein kinases ATM and ATR (reviewed in [8]). Other substrates of the E1B-55k/E4orf6 complex that are independent of MRN and play a major role in the host-cell intrinsic defence mechanism against adenovirus include p53, DNA ligase IV, integrin α3, Bloom helicase, DAXX/ATRX, Tip60 and SPOC1 [9–15].

The adenovirus E4orf3 protein is also critical for inducing specific degradation of host proteins. Following infection, this viral protein reorganizes promyelocytic leukaemia (PML) nuclear bodies into distinctive track-like structures in the nucleus, where it then transiently binds to E1B-55k [16, 17]. It is thought that E4orf3 sequesters MRN components into these tracks and forms a complex with E1B-55k and E4orf6 in order to transport MRN to cytoplasmic aggresomes for the degradation of MRE11 [18, 19]. Even in the absence of E1B-55K and E4orf6, the relocalization of MRN to nuclear tracks by E4orf3 alone is enough to inactivate the DDR [20]. In fact, it has been shown that E4orf3 can independently utilize the ubiquitin–proteasome pathway to promote the degradation of transcriptional intermediary factor 1γ (TIF1γ) [21]. An additional cellular substrate of E4orf3 has recently been identified; the general transcription factor II–I (GTF2I) is targeted by E4orf3 for ubiquitination and proteasomal degradation, which stimulates gene expression from a GTF2I-repressed viral promoter [22].

Allied to these observations, genetically modified adenoviruses that are deleted for one or more viral early gene(s) have been developed for experimental and clinical purposes (reviewed in [23, 24]). An adenovirus deletion mutant called dl1520 (also known as ONYX-015) that lacks the *E1B-55k* gene product is the first conditionally replicating adenovirus tested in humans to treat cancer [25]. This mutant virus was reported in 1987 [26] and from 1996 was developed as an anti-cancer agent in clinical trials in the United States [27]. The dl1520 virus was originally thought to selectively replicate in and lyse p53-deficient cancer cells. However, increasing evidence revealed that the precise role of p53 in determining dl1520 replication selectivity was unclear [28–31]. Current evidence suggests that both p53 inactivation and preferential viral late RNA export, which has been proposed to determine the tumour selectivity of the E1B-55k-deleted oncolytic adenovirus, are associated with E1B-55k-mediated ubiquitination [9, 32, 33]. A system-wide comparison of protein abundance in wild-type adenovirus- and dl1520-infected human cells should provide greater information on how E1B-55k affects the host cell.

Quantitative tandem mass spectrometry (MS/MS)-based proteomics is widely used to identify and quantify a large number of proteins in a high-throughput manner. Tandem mass tagging (TMT) of peptide samples enables multiplex relative quantitation by mass spectrometry (MS). This approach has been utilized to identity a large number of proteins and quantitate the relative abundance of proteins in response to stimuli [34, 35]. In this study, we used this technique to identify proteins that are depleted in wild-type adenovirus-infected cells and/or dl1520-infected cells.

We have previously published a dataset of quantitative proteomics to identify proteins that may be subjected to degradation by wild-type adenovirus [36]. This study represents a more focused analysis of the role of E1B-55k in virally mediated degradation of cellular proteins, as this protein and its effects on cellular protein degradation have been widely studied. We analysed protein abundance changes in A549 cells during wild-type or dl1520 virus infection using time-course proteomics. This study provides the first global analysis of E1B-55k-associated protein expression changes in human carcinoma cells. Moreover, we validated E1B-55k-mediated degradation of more novel proteins in adenovirus-infected cells, and identified those early-region adenovirus proteins that contribute to their degradation. Thus, we showed that quantitative proteomic analysis of virally infected cells is effective at identifying novel targets of specific virally induced degradation. Moreover, the dataset provides insights into the abundance changes of almost 5900 cellular proteins that occur during wild-type adenovirus or dl1520 infection. This should serve as a useful resource, potentially leading to a greater understanding of the role of E1B-55k during infection.

## Results

### Comparative proteome analysis of wild-type, and E1B-55k-deletion, adenovirus-infected cells

**Fig. 1.**
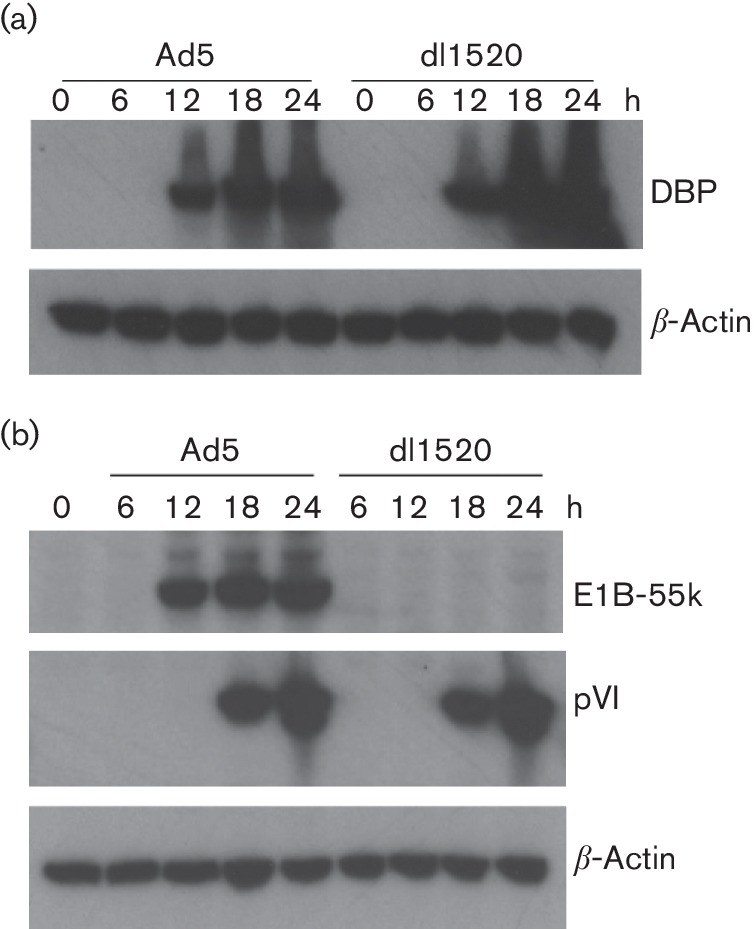
Infection of A549 cells with Ad5 and dl1520. A549 cells were infected with Ad5 and dl1520 at a multiplicity of infection (MOI) of 10 for 0, 6, 12, 18 and 24 h. The cell lysates were harvested and the presence of the viral proteins DBP (a), E1B-55k (b) and pVI (b) were monitored as markers of viral infection by Western blotting. β-Actin was an internal control.

To gain a comprehensive overview of the whole-cell protein expression in response to infection by wild-type human adenovirus type 5 (Ad5) or E1B-55k-deleted adenovirus (dl1520), we exploited 10-plex TMT quantitation to compare relative protein abundance over a time course. As A549 cells are highly permissive for both Ad5 and dl1520 adenovirus infection [29], we used A549 cells as the host cells in this study. We first examined the accumulation of viral early and viral late proteins in A549 cells, as a function of time of infection. The results of the preliminary experiments were used to design a time series that covered the virus infectious cycle, while focusing on the period (6–24 h after infection) in which the synthesis of viral macromolecules and changes in viral protein expression were maximal. Western blot analysis showed that the viral-early E2A DBP and E1B-55k proteins were expressed at 12 h post-infection (h p.i.) (Fig. 1a, b), while the viral-late protein VI (pVI) was produced at 18 h p.i. (Fig. 1b).

**Fig. 2.**
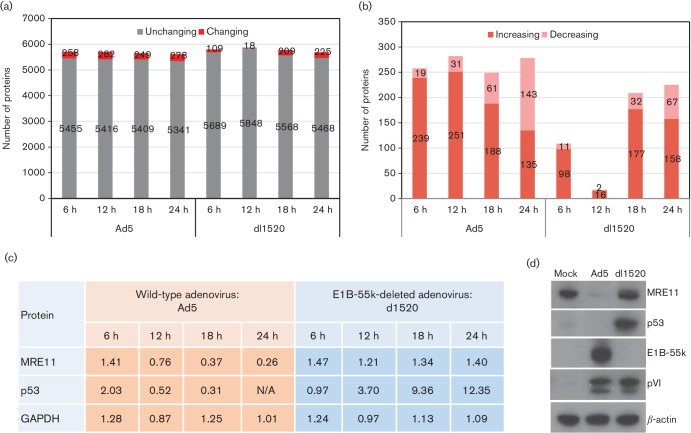
TMT-based proteomic time course of protein expression in adenovirus-infected A549 cells. Ad5- and dl1520-virus-infected A549 cells were sampled over 24 h and the relative abundance of cellular proteins was monitored by an LC-MS/MS system. We used proteins identified by at least two peptides in this analysis. (a) Summary of the proteomics data. The number of proteins exhibiting a change in abundance (changing) or remaining unchanged (unchanging) after Ad5 or dl1520 infection of A549 cells at all time points is indicated. (b) The number of proteins that showed a 2.5-fold-or-greater increase (increasing) or a 50 % decrease (decreasing) during infection. (c) Temporal profiles of MRE11 and p53 obtained in the MS/MS analysis. GAPDH was an internal control. The protein abundance ratio is shown for wild-type or dl1520 adenovirus-infected cells compared to uninfected cells. N/A indicates no abundance ratio could be determined in that experiment, even though the protein was identified. (d) The cell lysates harvested at 24 h p.i. were assessed for the presence of the cellular proteins MRE11 and p53 by Western blotting. The viral early protein E1B-55k and viral late protein pVI were monitored as markers of adenoviral infection. β-Actin served as a loading control.

At four time points following Ad5 or dl1520 infection (6, 12, 18 and 24 h) with uninfected cells (0 h), Ad5- and dl1520-virus-infected A549 cells were harvested, and cells from the nine experiments were processed as described in the Methods section for quantitative proteomic analyses. The raw data files were processed and quantified using Proteome Discoverer software. At a false discovery rate (FDR) of 1 %, this analysis identified 8190 proteins (Table S1, available in the online Supplementary Material). For further proteome analysis, the protein abundance ratio was represented for wild-type or dl1520 adenovirus-infected cells compared to uninfected cells. We retained proteins identified and quantitated by at least two peptides and with an abundance ratio variability of less than 200 %. Protein abundance ratios are normally reported as the median value of all the ratios from peptides quantitated and identified as belonging to the protein in question. Allied to this, the abundance ratio variability percentage is an indicator of how divergent the peptide ratios used to calculate a particular protein ratio are – large percentage values indicate that individual peptides may be reporting very different abundance ratios, which in our experience means that the median value may be misleading (Table S1). Any protein exhibiting a 2.5-fold-or-greater increase, or a 50 % decrease, based on the protein relative abundance of proteins at 24 h p.i., was flagged as having significantly changed abundance. By this criterion, following virus infection, more than 5300 proteins were apparently unchanged in protein abundance, and less than 5 % of identified proteins were changed in abundance (Fig. 2a). The number of virus down-regulated proteins gradually increased during virus infection (Fig. 2b). The quality of the proteome-wide quantitation was demonstrated by the consistency of the dynamic MS-based profiles for key marker proteins (Fig. 2c, d). In line with previous studies, MRE11 and p53, two known targets of E1B-55k/E4orf6 E3 ubiquitin ligase, were decreased during wild-type adenovirus infection, whilst in dl1520-infected A549 cells, MRE11 protein levels remained unchanged and p53 protein abundance increased significantly (Fig. 2c). Moreover, in agreement with observations reported by research groups [37, 38], a transient increase in p53 protein abundance early in Ad5 infection (at 6 h p.i.) and during dl1520 infection (6–24 h p.i.) was also determined in the MS analysis (Fig. 2c).

### Adenovirus E1B-55k protein regulates expression of cellular proteins and cellular programmes

**Fig. 3.**
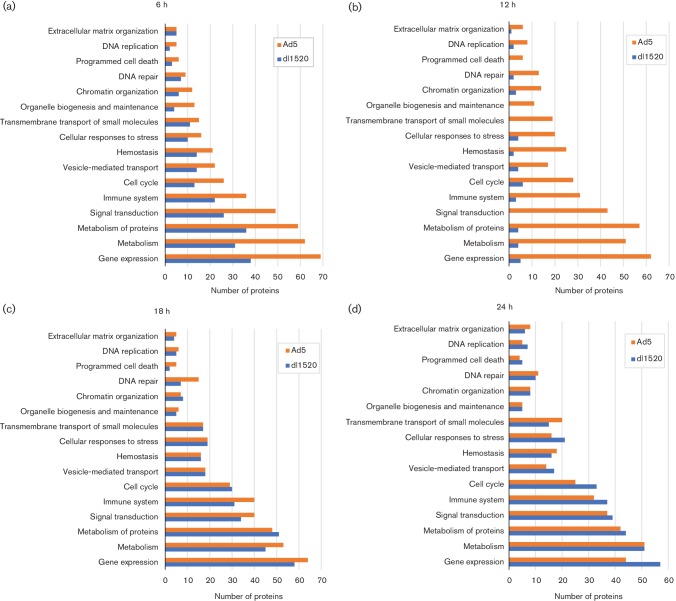
Biological pathway enrichment analysis using the Reactome database. Cellular proteins exhibiting changes during Ad5 infection and during dl1520 were analysed by the Reactome database (www.reactome.org/): (a) 6 h post-infection; (b) 12 h post-infection; (c) 18 h post-infection; (d) 24 h post-infection. The number of proteins is indicated at the bottom of the figure. Ad5-regulated proteins are shown in orange and dl1520-regulated proteins are shown in blue.

To understand the cellular programmes regulated by the altered proteins during Ad5 infection and dl1520 infection, we performed biological pathway enrichment analysis using the Reactome database [39, 40]. This revealed that the changes induced spanned a broad range of categories (Fig. 3 and Table S2). During wide-type adenovirus (Ad5) infection, the cellular proteins most affected were associated with chromatin organization, DNA replication and cell cycle at 6 h p.i.; with chromatin organization, DNA replication and cellular responses to stress at 12 h p.i.; with DNA replication, cellular responses to stress and DNA repair at 18 h p.i.; and with DNA replication, cellular responses to stress and cell cycle at 24 h p.i. (Table S2). Compared to Ad5 infection, during dl1520 infection there were fewer cellular proteins affected in the early phase (Fig. 3a, b), whereas during the late phase the number of cellular proteins affected was comparable to the changes seen during Ad5 infection (Fig. 3c, d) and the cellular proteins most affected were implicated in DNA replication and cellular responses to stress and cell cycle (Table S2). The expression of the viral early E2A DBP and E1B-55k proteins and the late protein VI (pVI) shown in Fig. 1, illustrates that the two viruses were at comparable stages in their viral replication cycles in this experiment.

### Protein abundance is not exclusively regulated by E1B-55k

We first identified those proteins that decreased significantly during Ad5 infection: 69 proteins were shown to be decreased more than twofold in response to Ad5 infection. Secondly, the 69 proteins degraded during Ad5 infection were examined to see how their abundance changed during dl1520 infection to highlight those proteins whose degradation may be at least partly E1B-55k mediated. The results are showed in Table 1. Among the 69 proteins, 41 exhibited a 50 % decrease in dl1520-infected A549 cells. Thus, many cellular proteins may be degraded by adenovirus by mechanisms independent of E1B-55k status. This is consistent with a recent study showing that GTF2I degradation is dependent upon adenovirus E4orf3 expression [22], something also reflected in this data set. We have also included RNA-Seq data on changes to gene expression during adenovirus infection, which we reported in an earlier study of adenovirus infection of HeLa cells [36]. However, changes in gene expression as a proxy for protein expression need to be treated cautiously, as this does not take into account other factors that affect protein abundance, such as translation initiation, whether a protein is exported from the cell, or the half-life of proteins.

**Table 1.** Proteins decreased in A549 cells infected with adenovirus A list of proteins that decreased in abundance more than twofold in Ad5- and/or dl1520-infected A549 cells compared to uninfected A549 cells at 24 h p.i.; the data were generated in this study. Fold change in gene expression between Ad5-infected HeLa cells at 24 h p.i. and uninfected HeLa cells; the RNA–Seq data were obtained from our previous studies [36].

UniProt accession	Protein description	Gene name	mRNA fold change in HeLa cells
**Protein decreased in amount >twofold during Ad5 infection and during dl1520 infection**			
A0A024RCT1	TAP binding protein (Tapasin), isoform CRA_c	TAPBP	0.56
P55268	Laminin subunit beta-2	LAMB2	1.56
X5CMH5	TAP2	TAP2	3.92
Q99650	Oncostatin-M-specific receptor subunit beta	OSMR	1.03
B2RMV2	CYTSA protein	CYTSA	2.15
P35568	Insulin receptor substrate 1	IRS1	0.24
Q0IIN7	Nuclear receptor coactivator 3	NCOA3	0.79
P05121	Plasminogen activator inhibitor 1	SERPINE1	0.11
Q8N6N3	UPF0690 protein C1orf52	C1orf52	0.46
Q14641	Early placenta insulin-like peptide	INSL4	0
B7Z168	Neuregulin 1 isoform HRG-alpha	NRG1	0
Q9UK54	Haemoglobin beta subunit variant	HBB	Not detected
X5D2J9	General transcription factor IIi isoform D	GTF2I	0.79
Q7Z5L9	Interferon regulatory factor 2-binding protein 2	IRF2BP2	0.61
P15428	15-hydroxyprostaglandin dehydrogenase [NAD(+)]	HPGD	0.72
P17676	CCAAT/enhancer-binding protein beta	CEBPB	0.24
H0Y485	Insulin-like growth factor-binding protein 3	IGFBP3	0.87
P40189	Interleukin-6 receptor subunit beta	IL6ST	0.97
A0A087WV90	Dystrophin	DMD	1.19
Q12860	Contactin-1	CNTN1	Not detected
Q8IY57	YY1-associated factor 2	YAF2	0.6
Q9NPA3	Mid1-interacting protein 1	MID1IP1	0.35
Q5MJ32	Protection of telomeres protein 1 variant 5	POT1	1.36
O00767	Acyl-CoA desaturase	SCD	2.64
A2AJT9	Uncharacterized protein CXorf23	CXorf23	0
Q8IUC4	Rhophilin-2	RHPN2	0.55
Q5VUA4	Zinc finger protein 318	ZNF318	0.41
O14757	Serine/threonine-protein kinase Chk1	CHEK1	1.16
P14384	Carboxypeptidase M	CPM	1.28
O43318	Mitogen-activated protein kinase kinase kinase 7	MAP3K7	0.7
P35869	Aryl hydrocarbon receptor	AHR	0.29
Q8NCD3	Holliday junction recognition protein	HJURP	0.11
P78545	ETS-related transcription factor Elf-3	ELF3	0.07
Q07973	1,25-dihydroxyvitamin D(3) 24-hydroxylase, mitochondrial	CYP24A1	0.18
A0A087WWW9	B-cell lymphoma/leukaemia 10	BCL10	0.31
P35354	Prostaglandin G/H synthase 2	PTGS2	0
Q59GZ8	Urokinase plasminogen activator preproprotein variant	PLAUR	0.79
P63313	Thymosin beta-10	TMSB10	0.54
B2RUU3	Dedicator of cytokinesis 1	DOCK1	0.28
F5H7B7	ATP-binding cassette sub-family A member 13	ABCA13	Not detected
P62328	Thymosin beta-4	TMSB4X	Not detected
**Protein decreased in amount twofold during Ad5 infection and slightly decreased (1.5–2 fold) during dl1520 infection**			
H3BS02	[3-methyl-2-oxobutanoate dehydrogenase (lipoamide)] kinase	BCKDK	1.3
Q6QWC0	TAP1	TAP1	Not detected
Q9NZV1	Cysteine-rich motor neuron 1 protein	CRIM1	0.24
Q13641	Trophoblast glycoprotein	TPBG	0.48
Q96L35	EPH receptor B4, isoform CRA_b	EPHB4	0.51
Q96HR8	H/ACA ribonucleoprotein complex non-core subunit NAF1	NAF1	1.19
P17275	Transcription factor jun-B	JUNB	0.17
A2ACR1	Proteasome subunit beta type	PSMB9	Not detected
Q9H1E3	Nuclear ubiquitous casein and cyclin-dependent kinase substrate 1	NUCKS1	1
Q96DI9	POLDIP3 protein	POLDIP3	0.87
Q96BU1	S100P-binding protein	S100PBP	1.36
H3BUT5	Nuclear envelope phosphatase-regulatory subunit 1	CNEP1R1	0
A0A024R4D3	Melanophilin, isoform CRA_b	MLPH	Not detected
Q9UMY1	Nucleolar protein 7	NOL7	1.11
Q6IE81	Protein Jade-1	JADE1	0.37
Q6MZZ4	Putative uncharacterized protein DKFZp686H0575	ELF1	0.45
Q14814	Myocyte-specific enhancer factor 2D	MEF2D	0.56
**Protein decreased in amount >twofold during Ad5 infection but did not decrease during dl1520 infection**			
B6E4×6	Cellular tumour antigen p53	p53	1.24
P49959	Double-strand break repair protein MRE11A	MRE11A	2.16
Q92878	DNA repair protein RAD50	RAD50	2.18
Q13740	CD166 antigen	ALCAM	1.36
P29317	Ephrin type-A receptor 2	EPHA2	0.41
F1T0K4	DmX-like protein 1	DMXL1	0.89
E9PMH5	Baculoviral IAP repeat-containing protein 2	BIRC2	0.49
O60934	Nibrin	NBN	1.19
Q8NAF0	Zinc finger protein 579	ZNF579	0
P10586	Receptor-type tyrosine-protein phosphatase F	PTPRF	4.71
Q7Z589	Protein EMSY	EMSY	0.69

### The adenovirus early-region proteins E1B-55k, E4orf3 and E4orf6 mediate the removal of the cellular proteins ALCAM, EPHA2 and PTPRF

Comparing protein abundance changes between Ad5-infected A549 cells and dl1520-infected A549 cells, our data highlighted cellular proteins previously known to be targeted by adenovirus for E1B-55k-mediated degradation, including MRE11, RAD50 and NBN (also known as NBS1) [7] (Table 2). We looked for additional cellular targets with a similar pattern of degradation during Ad5 infection: that is, a decline in protein abundance in the presence of E1B-55k. More than ten proteins meeting this criterion were identified, including three cell-adhesion molecules, ALCAM (CD166), EPHA2 (Eck) and PTPRF (LAR). As cell-adhesion molecules play roles in cell detachment and promoting the release and spread of progeny virions, they may represent important new substrates for degradation by the E1B-55k/E4orf6 E3 ubiquitin ligase, and therefore we decided to analyse this class of cell proteins further (Table 2).

**Table 2.** Proteins decreased in A549 cells infected with Ad5, but remained unchanged during dl1520 infection

Protein name	Ad5*				dl1520†			
	**6** h	**12** h	**18** h	**24** h	**6** h	**12** h	**18** h	**24** h
MRE11A‡	1.41	0.76	0.37	0.26	1.47	1.21	1.34	1.40
RAD50‡	1.35	0.69	0.35	0.30	1.38	1.28	1.29	1.21
NBN‡	1.18	1.19	0.58	0.42	1.23	1.21	1.10	0.92
ALCAM	1.11	0.77	0.52	0.47	1.07	1.20	1.37	1.14
EPHA2	1.24	1.23	0.54	0.46	1.32	1.24	1.23	1.09
PTPRF	0.79	0.85	0.59	0.43	0.82	0.81	0.74	0.73

*Protein abundance ratio is shown for wild-type adenovirus (Ad5)-infected cells compared to mock-infected cells.

†Protein abundance ratio is shown for E1B-55k-deleted adenovirus (dl1520)-infected cells compared to mock-infected cells.

‡Known cellular proteins whose levels are reduced via adenoviral E1B-55k functions.

**Fig. 4.**
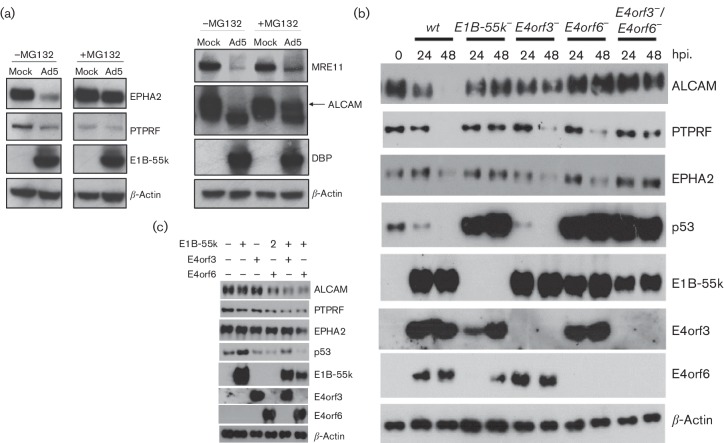
Ad5-induced degradation of the cellular proteins ALCAM, EPHA2 and PTPRF is mediated by the adenovirus early-region proteins E1B-55k, E4orf3 and/or E4orf6. (a) Effect of proteasome inhibition on ALCAM, EPHA2 and PTPRF levels. A549 cells were uninfected (mock) or infected with Ad5 or dl1520 at an MOI of 10. After 6 h the uninfected and adenovirus-infected cells were treated with the proteasome inhibitor MG132 at a final concentration of 20 µM. The cells were incubated under normal conditions for a further 18 h and then harvested. ALCAM, EPHA2, PTPRF, MRE11, E1B-55k and DBP were visualized by immunoblotting using the appropriate antibodies. β-Actin served as a loading control. The left-hand part of the panel was cropped from the same gel exposure in the case of each of the antigens shown. (b) Infection of A549 cells. A549 cells were left uninfected or infected with either Ad5 or mutant Ad5 preparations at an MOI of 5. Total cell extracts were prepared 24 and 48 h p.i., and proteins were separated by SDS-PAGE and subjected to immunoblotting using E1B-55k, E4orf3, E4orf6, p53, ALCAM, EPHA2 and PTPRF antibodies. β-Actin served as a loading control. (c) A549 cells were transfected with plasmids expressing combinations of adenovirus E1B-55k, E4orf3 and E4orf6, as indicated. After 48 h the total cell lysate was harvested and, as before, cellular proteins were detected by Western blots with β-actin used as a loading control.

In order to independently validate the TMT data for the selected proteins, we performed Western blot analysis on Ad5-infected A549 cells treated with the proteasome inhibitor, MG132. The results confirmed that the protein levels of ALCAM, EPHA2 and PTPRF declined significantly in A549 cells infected with Ad5 virus (Fig. 4a). However, only the degradation of EPHA2 and ALCAM was sensitive to proteasome inhibition by MG132. When cells were treated with 20 µM MG132, EPHA2 and ALCAM the protein levels in Ad5-infected cells remained comparable to those of mock-infected cells, whereas, surprisingly, PTPRF levels were decreased in MG132-treated cells, even without viral infection. These results suggested that proteasome activity is required for adenovirus-mediated EPHA2 and ALCAM reduction. Since MG132 alone triggers degradation of PTPRF protein, its virally triggered degradation pathway remains less clear.

We next examined which early region proteins, E1B-55k, E4orf3 and/or E4orf6, were involved in the adenovirus-induced degradation of these target proteins, or whether an alternative degradative pathway had occurred. We examined the effect of wild-type or early-region gene-mutated adenovirus [E1B-55k-(dl1520), E4orf3-(pm4150), E4orf6-(pm4154), E4orf3-/E4orf6-(pm4155)] infection on ALCAM, EPHA2 and PTPRF (Fig. 4b). Allied to this, we wanted to determine if these adenovirus proteins alone could affect levels of these cellular proteins by transfecting A549 cells with plasmids expressing one or two viral early proteins (Fig. 4c). Intriguingly, although wild-type adenovirus (wt) reliably degraded ALCAM, PTPRF and EPHA2, the pathways triggering their degradation appear to be distinct and complex. From the transfection studies, expression of E4orf6 alone, or E1B-55k/E4orf3 or E1B-55k/E4orf6 combinations, can target ALCAM for some degradation (Fig. 4c). A549 cells infected with a virus lacking either E1B-55k or E4orf3 showed partial degradation of ALCAM, but the protein levels of ALCAM in cells infected with a virus lacking E4orf6 or E4orf3 and E4orf6 together remained unchanged (Fig. 4b). These results implicate E4orf6, E1B-55k/E4orf6 and E4orf3/E4orf6 in degradation of ALCAM. PTPRF levels decreased in A549 cells infected with a viral mutant that does not express E4orf3 (pm4150) or E4orf6 (pm4154), but were unaffected by E1B-55k-deleted (dl1520) and E4orf3/E4orf6-deleted viruses (pm4155) (Fig. 4b). The reduction of PTPRF was detected in A549 cells expressing either E1B-55k/E4orf3 or E1B-55k–E4orf6 (Fig. 4c) and partial degradation was observed by those expressing E4orf6 alone (Fig. 4c). The reduction in EPHA2 was still evident following infection with either E4orf3-deleted virus or E4orf6-deleted virus (Fig. 4b), while from transfection studies, E1B-55k/E4orf6 was involved in the reduction of EPHA2 (Fig. 4c). We determined that proteasome activity is required for ALCAM and EPHA2 reduction (Fig. 4a), and the effects of adenovirus infection on ALCAM and EPHA2 levels (Fig. 4b, c) support the contention that at least the E1B-55k/E4orf6 complex targets ALCAM and EPHA2 for ubiquitination and subsequent proteasome-mediated degradation.

## Discussion

In this time-course study, global analysis of the host proteome remodelling during adenovirus infection was performed using a more focused approach: comparing protein abundance in wild-type vs E1B-55k-deletion-mutant adenovirus-infected cells. This produced a temporal map of protein abundance changes for 5937 proteins in Ad5-infected A549 cells compared to dl1520-infected A549 cells. Bioinformatics analysis using the Reactome database revealed that the adenovirus-regulated proteins were involved in several cellular programmes, including chromatin organization, DNA replication, cell cycle and cellular response to stress (Fig. 3 and Table S2).

Adenovirus early-region proteins interact together to modify critical cellular pathways and prepare the host cell for viral replication. As a key example of this, E1B-55k, E4orf3 and E4orf6 have been the subject of much research and there are several well-characterized interactions between these viral proteins and the host cells (reviewed in [41]). Our proteomics data revealed some 69 cellular proteins that are degraded following adenovirus infection, but of these, E1B-55k appears to be required for the shut-off and removal of just 11 cellular proteins, some novel and some previously known (Table 1).

Dallaire *et al.* [10] identified the cell-surface molecule, integrin α3, as a substrate of the E1B-55k-mediated E3 ligase complex and suggested that other adhesion molecules may also be affected by E4orf6/E1B-55k. In this study, we found that three novel cell adhesion molecules, ALCAM, EPHA2 and PTPRF, are affected by E1B-55k, E4orf6 and/or E4orf3, and the pathways triggering their degradation are more complex than has previously been reported.

ALCAM protein was degraded by wild-type adenovirus (Fig. 4b) and the analysis of infection with single or double mutants revealed that there appear to be two or more mechanisms promoting degradation. In the context of transfection experiments (Fig. 4c), the degradation of ALCAM appears to occur on the expression of E4orf6 alone, E1B-55k/E4orf3 or E1B-55k/E4orf6. In the context of a full virus infection, E4orf6 is required for ALCAM reduction (Fig. 4b). In addition, removal of ALCAM is dependent on the proteasome activity (Fig. 4a). Together, these data suggest that ALCAM is a target of adenoviral E1B-55k/E4orf6 ubiquitin ligase. However, additional studies will be required to explain in detail how adenovirus infection modulates overall ALCAM abundance.

Nevertheless, degradation of ALCAM may be a strategy for cell detachment and restricting immune surveillance via adenovirus-induced targeted ubiquitination. ALCAM, also known as CD166, is an activated leukocyte cell-adhesion molecule that mediates both heterotypic cell–cell contacts and homotypic cell–cell contacts [42, 43]. In addition, ALCAM has been identified as a bona fide target of viral protein K5, a modulator of immune recognition from Kaposi's sarcoma-associated herpesvirus, which prevents activation of cytotoxic T cells and natural killer cells [44]. Potentially, the clearance of ALCAM during infection might inhibit ALCAM-dependent immune responses and promote adenovirus propagation.

EPHA2, also called Eck (epithelial cell kinase), is a receptor tyrosine kinase that binds to membrane-bound ephrin-A family ligands and functions to regulate cell migration and cell growth [45]. Our data suggest that EPHA2 is a novel substrate for Ad5 E1B-55k-mediated proteasomal degradation. Studies with mutant adenoviruses revealed that EPHA2 degradation is dependent upon E1B-55k/E4orf6 expression (Fig. 4b, c). However, our data also suggest that there might be redundant pathways involved in the E1B-55k/E4orf3-mediated EPHA2 reduction (Fig. 4b, c).

PTPRF, also known as LAR (leukocyte common antigen related), is a receptor-like protein tyrosine phosphatase located in the plasma membrane. The degradation of PTPRF appears to be dependent upon adenoviral E1B-55k/E4orf6 or E1B-55k/E4orf3 protein expression (Fig. 4b, c). However, as PTPRF protein levels were reduced in MG132-treated uninfected A549 cells via an unknown mechanism (Fig. 4a), further work is required to determine exactly how adenovirus reduces overall levels of this protein in the cell. Evidence from recent work indicates that PTPRF modulates cell migration through dephosphorylating EPHA2 [46]. The EPHA2 receptor is also known to interact with integrin α3 (a known substrate of the E1B-55k/E4orf6 E3 ubiquitin ligase complex) in U251MG glioblastoma cells, and contributes to the crosstalk between Eph and integrin signalling pathways at membrane protrusions [10, 47]. Taken together, our data suggest that it is likely that adenovirus regulates cell-surface proteins through E1B-55k, and the relationship between adenovirus infection and cell-surface molecules is more complex than is currently understood.

There is also a formal possibility that the reduction of protein abundance could be an indirect result of changes in mRNA levels or it might be because adenovirus induces the export and shedding of these cellular proteins. However, as EPHA2 and ALCAM degradation is readily blocked by MG132, we argue that the weight of evidence suggests that their degradation is a post-translational effect mediated by combinations of E1B-55k and/or E4orf6 and E4orf3. For PTPRF, additional work is needed to understand exactly how adenovirus mediates this change in cellular abundance. Nevertheless, regardless of the precise mechanism, our data significantly widen the list of cell-surface proteins downregulated by adenovirus infection.

As pointed out in the Introduction, a number of proteins are known to be down-regulated by adenovirus infection (e.g. DNA ligase IV and integrin α3), but they were not identified in this study. This reflects one of the downsides of mass-spectrometry-based proteomics, namely that it is not yet possible to guarantee that all proteins will be confidently identified and quantitated.

In conclusion, we have characterized relative protein abundance changes in A549 cells infected with wild-type adenovirus and/or E1B-55k-deleted adenovirus. From these data we have obtained a range of novel cellular proteins targeted by adenovirus for degradation, including the identification of novel E1B-55k-dependent targets. This study identifies a wider range of cellular targets for adenovirus-mediated degradation and our follow-up analysis revealed that adenovirus-mediated protein degradation may involve multiple (and potentially partially redundant) pathways. Moreover, the time-course dataset for protein abundance changes is a valuable data resource for the study of wild-type and E1B-55k-deleted adenoviruses.

## Methods

### Cells and viruses

Human lung carcinoma cells (A549) were cultured in Dulbecco’smodified Eagle’s medium with 4.5 g l^−1^ glucose and l-glutamine (DMEM, Lonza), supplemented with 10 % fetal bovine serum (FBS), streptomycin (100 µg ml^−1^) and penicillin (100 IU ml^−1^) (Life Technologies). Wild-type human adenovirus type 5 (Ad5) was propagated as described previously [48] and preparations were purified twice by caesium chloride density gradient centrifugation [49]. The mutant human adenovirus preparations, deleted in E1B-55k (dl1520), E4orf3 (pm4150), E4orf6 (pm4154) and E4orf3 and E4orf6 (pm4155), have been described previously [21, 26, 50]. We thank Thomas Dobner (HPI, Hamburg) for kindly providing E4orf3 (pm4150), E4orf6 (pm4154) and E4orf3 and E4orf6 (pm4155) deletion viruses.

### Adenovirus infection, TMT labelling and high pH reversed-phase chromatography

A549 cells were seeded at a concentration of 1×10^6^ cells and left overnight to adhere. The cultures were infected with either Ad5 or dl1520 at a multiplicity of infection (MOI) of 10. The cells were incubated under normal conditions for 0, 6, 12, 18 and 24 h, and then harvested and washed twice in PBS. For each sample, the cell pellets were resuspended in five-cell-pellet volumes of lysis buffer containing 8 M urea in 50 mM triethylammonium bicarbonate (TEAB) (Thermo Scientific), pH 8, and ×1 protease inhibitor cocktail (Roche, 11836170001), followed by sonication (15 s×2 on ice, at 20 % output). Then, the samples were spun at 17 000 *g* for 10 min at 4 °C. The protein concentration of the cell extracts was determined using the BCA protein assay kit (Thermo Fisher Scientific).

Aliquots of 100 µg of ten samples per experiment were digested with trypsin (2.5 µg trypsin per 100 µg protein; 37 °C, overnight) and labelled with tandem mass tag (TMT) ten-plex reagents according to the manufacturer’s protocol (Thermo Fisher Scientific); the labelled samples were then pooled.

A 50 µg aliquot of the pooled sample was evaporated to dryness and resuspended in buffer A (20 mM ammonium hydroxide, pH 10) prior to fractionation by high-pH reversed-phase chromatography using an Ultimate 3000 liquid chromatography system (Thermo Fisher Scientific). In brief, the sample was loaded onto an XBridge BEH C18 column (130 Å, 3.5 µm, 2.1×150 mm; Waters, UK) in buffer A and the peptides were eluted with an increasing gradient of buffer B (20 mM ammonium hydroxide in acetonitrile, pH 10) from 0–95 % over 60 min. The resulting fractions were evaporated to dryness and resuspended in 1 % formic acid prior to analysis by nano-LC MS/MS using an Orbitrap Fusion Tribrid mass spectrometer (Thermo Scientific).

### Nano-LC mass spectrometry

High-pH RP fractions were further fractionated using an Ultimate 3000 nano HPLC system in line with an Orbitrap Fusion Tribrid mass spectrometer (Thermo Scientific). In brief, peptides in 1 % (vol/vol) formic acid were injected onto an Acclaim PepMap C18 nano-trap column (Thermo Scientific). After washing with 0.5 % (vol/vol) acetonitrile 0.1 % (vol/vol), formic acid peptides were resolved on a 250 mm×75 µm Acclaim PepMap C18 reverse-phase analytical column (Thermo Scientific) over a 150 min organic gradient, using seven gradient segments (1–6 % solvent B over 1 min, 6–15 % B over 58 min, 15–32 % B over 58 min, 32–40 % B over 5 min, 40–90 % B over 1 min, held at 90 % B for 6 min and then reduced to 1 % B over 1 min) with a flow rate of 300 nl min^−1^. Solvent A was 0.1 % formic acid and solvent B was aqueous 80 % acetonitrile in 0.1 % formic acid. Peptides were ionized by nano-electrospray ionization at 2.0 kV using a stainless steel emitter with an internal diameter of 30 µm (Thermo Scientific) and a capillary temperature of 275 °C.

All spectra were acquired using an Orbitrap Fusion Tribrid mass spectrometer controlled by Xcalibur 1.9 software (Thermo Scientific) and operated in data-dependent acquisition mode using an SPS-MS3 workflow. FTMS1 spectra were collected at a resolution of 120 000, with an automatic gain control (AGC) target of 200 000 and a max injection time of 50 ms. The top *N* most intense ions were selected for MS/MS. Precursors were filtered according to charge state (to include charge states 2–7) and with monoisotopic precursor selection. Previously interrogated precursors were excluded using a dynamic window (40s+/−10 p.p.m.). The MS2 precursors were isolated with a quadrupole mass filter set to a width of 1.2 *m/z*. ITMS2 spectra were collected with an AGC target of 5000, a max injection time of 70 ms and a CID collision energy of 35 %.

For FTMS3 analysis, the Orbitrap was operated at 60 000× resolution with an AGC target of 50 000 and a max injection time of 120 ms. Precursors were fragmented by high-energy collision dissociation (HCD) at a normalized collision energy of 55 % to ensure maximal TMT reporter ion yield. Synchronous precursor selection (SPS) was enabled to include up to five MS2 fragment ions in the FTMS3 scan.

### Data analysis

The raw data files were processed and quantified using Proteome Discoverer software v1.4 (Thermo Scientific) and searched against a combined database consisting of the UniProt human database (downloaded 8 November 2014 : 126 385 entries) and adenovirus protein sequences using the SEQUEST algorithm. Peptide precursor mass tolerance was set at 10 ppm and MS/MS tolerance was set at 0.6 Da. Search criteria included oxidation of methionine (+15.9949) as a variable modification and carbamidomethylation of cysteine (+57.0214) and the addition of the TMT (+229.163) to peptide N-termini and lysine as fixed modifications. Searches were performed with full tryptic digestion and a maximum of one missed cleavage was allowed. The reverse database search option was enabled and all peptide data were filtered to satisfy a false discovery rate (FDR) of 1 %.

### Infection of human A549 cells with Ad5 and mutant virus and treatment with MG132

A549 cells were seeded at a concentration of 2×10^5^ cells per well in a six-well plate and left overnight to adhere. The cultures were either uninfected, or infected with either Ad5 or dl1520 preps at an MOI of 10. After 6 h the uninfected and adenovirus-infected cells were treated with the proteasome inhibitor MG132 at a final concentration of 20 µM. The cells were incubated under normal conditions for a further 18 h and then harvested and washed twice in PBS. The cells were resuspended in PBS and SDS loading buffer containing 10 mM 2-mercaptoethanol and sonicated five times to ensure complete the extraction of total cellular proteins.

### Transfection of A549 cells

A549 cells were seeded at 5×10^5^ cells on 6 cm dishes. Four hours after plating, the cells were washed twice in Optimem (Life Technologies) and incubated with different combinations of pcDNA3, pcDNA3-Ad5-E1B-55k, pcDNA3-Ad5-E4orf3 and pcDNA3-Ad5-E4orf6 (see Fig. 4c legend) for 6 h, in the presence of Lipofectamine 2000 (Life Technologies), according to manufacturer’s recommendations. After transfection, cells were washed once in DMEM medium and incubated for an additional 24 h in DMEM before being washed twice in PBS and harvested and processed for subsequent analysis of cellular proteins by SDS-PAGE.

### Western blotting and antibodies

Test samples were first denatured by heating at 95 °C for 10 min and then separated by electrophoresis on a Tris-glycine polyacrylamide gel. Proteins were transferred to Hybond-P PVDF membrane (GE Healthcare, UK) and probed with a specific primary antibody, followed by a horseradish peroxidase (HRP)-conjugated secondary antibody. Blots were developed with the ECL detection system (GE Healthcare, UK). The primary antibodies used were monoclonal anti-DBP [48], mouse anti-E1B-55k [51], rabbit anti-pVI [48], polyclonal goat anti-MRE11, monoclonal mouse anti-p53 (Santa Cruz), polyclonal rabbit anti-ALCAM (Sino Biotechnology), polyclonal rabbit anti-β-actin (Cell Signaling), monoclonal mouse anti-Eck/EphA2 (Merck Millipore) and monoclonal mouse anti-LAR/PTPRF (BD Biosciences). Anti-E4orf3 and anti-E4orf6 antibodies were provided by Thomas Dobner (HPI, Hamburg). The secondary antibodies used were goat anti-rabbit IgG-HRP, goat anti-mouse IgG-HRP and donkey anti-goat IgG-HRP (Santa Cruz).

## Supplementary Data

1909 Supplementary File 1
