# Supplementary material for: Comparison of protein expression during wild-type, and E1B-55k-deletion, adenovirus infection using quantitative time-course proteomics
Source: J Gen Virol. 2017 Jun 20;98(6):1377–88. doi: 10.1099/jgv.0.000781 (PMC5656791; doi:10.1099/jgv.0.000781)
Supplement: Supplementary File 1 [file jgv-98-1377-s001.pdf]

		Ad5				dl1520			
**Accession**	**Protein Description**	**6 h**	**12 h**	**18 h**	**24 h**	**6 h**	**12 h**	**18 h**	**24 h**
Q96DI9	POLDIP3 protein (Fragment) OS=Homo sapiens GN=POLDIP3 PE	1.295	1.064	0.544	0.356	1.085	1.106	0.806	0.574
Q92878	DNA repair protein RAD50 OS=Homo sapiens GN=RAD50 PE=1 S	1.350	0.688	0.350	0.297	1.377	1.277	1.293	1.209
P49959	Double-strand break repair protein MRE11A OS=Homo sapiens G	1.414	0.761	0.370	0.263	1.470	1.211	1.339	1.400
X5D2J9	General transcription factor IIi isoform D (Fragment) OS=Homo s	1.172	0.613	0.278	0.224	0.976	0.683	0.442	0.360
O60934	Nibrin OS=Homo sapiens GN=NBN PE=1 SV=1 - [NBN_HUMAN]	1.185	1.189	0.579	0.416	1.229	1.210	1.102	0.922
Q13740	CD166 antigen OS=Homo sapiens GN=ALCAM PE=1 SV=2 - [CD1	1.108	0.769	0.522	0.470	1.072	1.198	1.373	1.135
P29317	Ephrin type-A receptor 2 OS=Homo sapiens GN=EPHA2 PE=1 SV	1.242	1.232	0.538	0.458	1.325	1.237	1.225	1.088
B6E4X6	Cellular tumor antigen p53 OS=Homo sapiens PE=2 SV=1 - [B6E	2.026	0.518	0.307		0.969	3.698	9.360	12.348
P10586	Receptor-type tyrosine-protein phosphatase F OS=Homo sapiens	0.790	0.855	0.594	0.434	0.819	0.806	0.743	0.729
V9HWK1	Triosephosphate isomerase OS=Homo sapiens GN=HEL-S-49 PE=	1.083	0.831	1.097	0.979	1.037	0.930	1.019	1.013
P04792	Heat shock protein beta-1 OS=Homo sapiens GN=HSPB1 PE=1 S	1.283	0.782	1.223	1.008	1.154	0.895	1.055	0.987
B8ZZU8	Transcription elongation factor B (SIII), polypeptide 2 (18kDa, elo	1.638	1.284	1.577	1.134	1.235	1.243	1.307	1.362
P05787	Keratin, type II cytoskeletal 8 OS=Homo sapiens GN=KRT8 PE=1	1.438	1.777	1.172	0.798	1.166	1.139	1.233	0.918
P05783	Keratin, type I cytoskeletal 18 OS=Homo sapiens GN=KRT18 PE=	2.293	2.390	1.813	1.036	2.032	1.170	1.702	1.333
P10809	60 kDa heat shock protein, mitochondrial OS=Homo sapiens GN=	1.385	1.446	1.286	1.440	1.049	1.249	1.450	1.455
P61604	10 kDa heat shock protein, mitochondrial OS=Homo sapiens GN=	1.790	1.809	1.624	1.576	1.247	1.203	1.643	1.769
A0A024RB53	Heterogeneous nuclear ribonucleoprotein A1, isoform CRA_a OS=	1.679	1.750	1.395	1.141	1.481	1.391	1.416	1.046
O75368	SH3 domain-binding glutamic acid-rich-like protein OS=Homo sap	1.546	1.028	1.875	1.626	1.207	1.128	1.634	1.477
Q9BSY4	Coiled-coil-helix-coiled-coil-helix domain-containing protein 5 OS=	1.802	1.784	2.088	1.438	1.708	1.640	1.510	1.772
Q6FG99	RPLP1 protein OS=Homo sapiens GN=RPLP1 PE=2 SV=1 - [Q6FG	1.936	1.418	1.823	1.469	1.693	1.057	1.593	1.747
G3V1A4	Cofilin 1 (Non-muscle), isoform CRA_a OS=Homo sapiens GN=CF	1.546	0.983	1.112	0.995	1.231	0.965	1.348	1.102
P63244	Guanine nucleotide-binding protein subunit beta-2-like 1 OS=Hon	2.012	1.692	1.903	1.347	1.913	1.318	1.680	1.544
R4GMU8	Ragulator complex protein LAMTOR5 OS=Homo sapiens GN=LAM		1.736	1.929	1.553	1.526	1.332	1.855	1.809
P04075	Fructose-bisphosphate aldolase A OS=Homo sapiens GN=ALDOA	1.316	0.839	1.268	1.110	1.172	0.934	1.101	1.124
Q06830	Peroxiredoxin-1 OS=Homo sapiens GN=PRDX1 PE=1 SV=1 - [PRI	1.251	0.774	1.140	0.947	1.092	1.024	1.184	0.967
P37802	Transgelin-2 OS=Homo sapiens GN=TAGLN2 PE=1 SV=3 - [TAGL	1.222	0.857	1.185	1.079	1.120	0.924	1.050	1.068
P08670	Vimentin OS=Homo sapiens GN=VIM PE=1 SV=4 - [VIME_HUMA	1.360	1.954	1.096	1.019	1.093	1.160	1.059	1.079
P51149	Ras-related protein Rab-7a OS=Homo sapiens GN=RAB7A PE=1 S	1.302	1.456	1.186	0.933	1.230	1.191	1.285	1.195
Q99714	3-hydroxyacyl-CoA dehydrogenase type-2 OS=Homo sapiens GN=	1.257	1.218	1.229	1.226	1.015	1.116	1.277	1.353
P80723	Brain acid soluble protein 1 OS=Homo sapiens GN=BASP1 PE=1	1.367	1.297	1.168	0.935	1.279	1.110	1.120	1.114
P30086	Phosphatidylethanolamine-binding protein 1 OS=Homo sapiens G	1.279	0.824	1.350	1.301	1.061	0.894	1.154	1.415
Q96C01	Protein FAM136A OS=Homo sapiens GN=FAM136A PE=1 SV=1 -	1.989	2.293	1.957	2.057	1.853	1.500	2.157	2.355
Q9UK76	Hematological and neurological expressed 1 protein OS=Homo sa	0.950	0.718	0.964	0.856	0.926	1.020	0.923	0.900
Q99880	Histone H2B type 1-L OS=Homo sapiens GN=HIST1H2BL PE=1 S	1.181	1.182	0.949	0.860	1.225	1.154	1.191	1.095
O75531	Barrier-to-autointegration factor OS=Homo sapiens GN=BANF1 P	2.517	3.332	3.555	3.398	1.258	1.267	2.196	3.848
P42330	Aldo-keto reductase family 1 member C3 OS=Homo sapiens GN=	1.140	0.752	1.289	0.656	1.255	0.589	1.317	1.003
P62820	Ras-related protein Rab-1A OS=Homo sapiens GN=RAB1A PE=1	1.069	1.200	0.967	0.921	1.019	1.148	1.154	1.075
P62244	40S ribosomal protein S15a OS=Homo sapiens GN=RPS15A PE=	1.602	1.150	1.220	0.968	1.156	1.004	1.133	1.070

P61247	40S ribosomal protein S3a OS=Homo sapiens GN=RPS3A PE=1 S	1.795	1.653	1.749	0.982	1.420	1.272	1.435	1.270
P00558	Phosphoglycerate kinase 1 OS=Homo sapiens GN=PGK1 PE=1 SV	1.215	0.792	1.215	1.121	1.045	0.977	1.141	1.121
Q7RTV0	PHD finger-like domain-containing protein 5A OS=Homo sapiens	1.750	1.475	1.485	0.912	1.403	1.339	1.339	1.189
O60218	Aldo-keto reductase family 1 member B10 OS=Homo sapiens GN	1.189	0.938	1.414	1.203	1.104	1.079	1.243	1.125
O95182	NADH dehydrogenase [ubiquinone] 1 alpha subcomplex subunit 7	1.337	1.290	1.249	1.238	1.004	1.189	1.324	1.262
P23396	40S ribosomal protein S3 OS=Homo sapiens GN=RPS3 PE=1 SV=	1.712	1.605	1.581	1.042	1.481	1.251	1.494	1.291
Q32Q12	Nucleoside diphosphate kinase OS=Homo sapiens GN=NME1-NM	1.286	0.959	1.469	1.348	1.137	1.163	1.381	1.293
P05387	60S acidic ribosomal protein P2 OS=Homo sapiens GN=RPLP2 PE	0.924	1.224	1.070	1.042	1.577	1.392	1.283	0.984
Q75MT9	Malate dehydrogenase (Fragment) OS=Homo sapiens GN=MDH2	1.273	1.375	1.121	1.198	1.098	1.161	1.298	1.283
Q2KS06	E2A DNA-binding protein GN=E2A_DBP PE=4 SV=1 - [Q2KS06_A	1.568	2.550	4.109	4.200	1.467	1.670	3.226	7.757
P14854	Cytochrome c oxidase subunit 6B1 OS=Homo sapiens GN=COX6E	1.441	1.444	1.540	1.576	1.345	1.162	1.499	1.431
Q9GZT3	SRA stem-loop-interacting RNA-binding protein, mitochondrial OS	1.234	1.435	0.998	1.048	0.798	1.083	0.875	0.942
P07737	Profilin-1 OS=Homo sapiens GN=PFN1 PE=1 SV=2 - [PROF1_HU	1.466	0.835	1.451	1.253	1.174	0.926	1.165	1.156
Q2Q9H2	Glucose-6-phosphate 1-dehydrogenase (Fragment) OS=Homo sa	1.156	0.527	1.056	0.753	0.988	1.003	1.110	0.709
A7BI36	p180/ribosome receptor OS=Homo sapiens GN=RRBP1 PE=2 SV=	1.165	1.328	0.677	0.413	0.988	1.060	0.806	0.501
V9HW35	Epididymis secretory protein Li 55 OS=Homo sapiens GN=HEL-S-	1.360	1.339	1.258	1.237	1.103	1.127	1.264	1.249
Q8WYB3	Beta II spectrin-short isoform (Fragment) OS=Homo sapiens PE=	2.088	2.074	1.759	1.253	1.629	1.511	1.818	1.543
O75947	ATP synthase subunit d, mitochondrial OS=Homo sapiens GN=AT	1.321	1.596	1.172	1.379	0.863	1.004	1.316	1.413
P22626	Heterogeneous nuclear ribonucleoproteins A2/B1 OS=Homo sapie	1.500	1.494	1.314	1.154	1.437	1.249	1.367	1.197
E5RHG8	Transcription elongation factor B polypeptide 1 (Fragment) OS=H	1.204	1.065	1.142	0.923	1.049	1.119	1.043	0.935
O43707	Alpha-actinin-4 OS=Homo sapiens GN=ACTN4 PE=1 SV=2 - [ACT	1.491	1.214	1.080	0.831	1.421	1.196	1.212	1.099
P08727	Keratin, type I cytoskeletal 19 OS=Homo sapiens GN=KRT19 PE=	1.299	1.556	0.873	0.474	1.033	1.039	0.949	0.667
P06733	Alpha-enolase OS=Homo sapiens GN=ENO1 PE=1 SV=2 - [ENOA	1.262	0.844	1.375	1.009	1.088	0.965	1.109	1.070
Q5RLJ0	CLE OS=Homo sapiens PE=2 SV=1 - [Q5RLJ0_HUMAN]	1.548	1.555	1.408	1.116	1.578	1.262	1.488	1.472
P07437	Tubulin beta chain OS=Homo sapiens GN=TUBB PE=1 SV=2 - [T	1.271	0.784	1.045	0.992	1.116	0.987	0.929	0.901
P63218	Guanine nucleotide-binding protein G(I)/G(S)/G(O) subunit gamm	0.813	1.055	0.813	0.863	0.820	1.026	0.942	1.031
P03271	IVa2 5 GN=IVA2 - [PIVA2_ADE05]	1.630	1.538	3.250	2.876	1.703	1.239	2.058	3.229
P11142	Heat shock cognate 71 kDa protein OS=Homo sapiens GN=HSPA	1.426	1.363	1.581	1.268	1.287	1.316	1.391	1.367
Q15181	Inorganic pyrophosphatase OS=Homo sapiens GN=PPA1 PE=1 SV	1.860	1.543	1.782	1.439	1.484	1.229	1.447	1.800
P00352	Retinal dehydrogenase 1 OS=Homo sapiens GN=ALDH1A1 PE=1	1.452	0.951	1.419	1.146	1.220	0.974	1.275	1.116
H3BPC4	SUMO-conjugating enzyme UBC9 (Fragment) OS=Homo sapiens G	1.857	1.700	1.626	1.391	1.995	1.500	1.738	1.660
P30041	Peroxiredoxin-6 OS=Homo sapiens GN=PRDX6 PE=1 SV=3 - [PRI	1.536	1.119	1.602	1.474	1.223	1.046	1.454	1.482
E5RIW3	Tubulin-specific chaperone A OS=Homo sapiens GN=TBCA PE=1	1.631	1.171	1.770	1.477	1.427	1.158	1.641	1.543
P16152	Carbonyl reductase [NADPH] 1 OS=Homo sapiens GN=CBR1 PE=	1.482	1.093	1.535	1.396	1.232	1.056	1.404	1.314
P21796	Voltage-dependent anion-selective channel protein 1 OS=Homo s	1.419	1.478	1.181	1.192	1.057	1.203	1.336	1.295
Q09666	Neuroblast differentiation-associated protein AHNAK OS=Homo sa	1.337	1.554	1.023	0.997	1.216	1.109	1.072	1.066
C9J1Z8	ADP-ribosylation factor 5 (Fragment) OS=Homo sapiens GN=ARF	1.518	1.119	1.422	1.055	1.587	1.227	1.322	1.272
P07355	Annexin A2 OS=Homo sapiens GN=ANXA2 PE=1 SV=2 - [ANXA2_	1.305	1.263	1.287	1.262	1.221	1.113	1.357	1.295
P62258	14-3-3 protein epsilon OS=Homo sapiens GN=YWHAE PE=1 SV=	2.651	1.683	2.213	2.029	1.775	1.123	2.020	2.037
O75934	Pre-mRNA-splicing factor SPF27 OS=Homo sapiens GN=BCAS2 PI	1.551	1.574	1.264	1.244	1.654	1.547	1.693	1.557

D6RBR1	Calpastatin (Fragment) OS=Homo sapiens GN=CAST PE=4 SV=1	2.946	1.943	2.802	1.787	1.921	0.929	2.097	1.868
P62072	Mitochondrial import inner membrane translocase subunit Tim10	3.207	3.018	2.500	2.971	2.817	1.472	2.661	3.169
Q9Y281	Cofilin-2 OS=Homo sapiens GN=CFL2 PE=1 SV=1 - [COF2_HUMA	1.498	1.095	1.390	1.230	1.225	0.961	1.044	1.180
R4GNH3	26S protease regulatory subunit 6A OS=Homo sapiens GN=PSMC	1.333	1.055	1.169	0.955	1.300	1.140	1.093	0.978
P60981	Destrin OS=Homo sapiens GN=DSTN PE=1 SV=3 - [DEST_HUMA	1.692	1.188	1.607	1.373	1.359	1.104	1.256	1.270
B2R7D7	cDNA, FLJ93394, highly similar to Homo sapiens anterior gradient	2.102	1.224	1.727	1.630	1.551	1.139	1.585	1.432
P31939	Bifunctional purine biosynthesis protein PURH OS=Homo sapiens	1.073	0.705	1.256	1.054	1.010	1.007	1.048	1.073
A8K8D9	Glucose-6-phosphate 1-dehydrogenase OS=Homo sapiens PE=2	1.443	1.067	1.584	1.208	1.213	0.991	1.270	1.290
Q9H0U4	Ras-related protein Rab-1B OS=Homo sapiens GN=RAB1B PE=1	1.149	1.176	1.028	1.034	0.941	1.094	1.273	1.175
O00151	PDZ and LIM domain protein 1 OS=Homo sapiens GN=PDLIM1 PI	1.300	1.082	1.095	0.747	1.188	1.049	0.989	0.950
P02545	Prelamin-A/C OS=Homo sapiens GN=LMNA PE=1 SV=1 - [LMNA_	1.456	1.451	1.152	1.195	1.442	1.244	1.264	1.258
C9JFR7	Cytochrome c (Fragment) OS=Homo sapiens GN=CYCS PE=1 SV	1.167	1.260	1.115	1.093	1.198	1.268	1.368	1.470
Q99653	Calcineurin B homologous protein 1 OS=Homo sapiens GN=CHP1	0.990	0.913	0.681	0.603	0.799	1.084	0.822	0.716
P14618	Pyruvate kinase PKM OS=Homo sapiens GN=PKM PE=1 SV=4 - [	2.156	1.532	2.444	2.091	1.735	0.962	1.645	2.078
B4DTV8	cDNA FLJ61399, highly similar to Spectrin alpha chain, brain OS=	1.562	1.446	1.281	0.894	1.490	1.145	1.260	1.319
A0A087WWU8	Tropomyosin alpha-3 chain OS=Homo sapiens GN=TPM3 PE=4 S	1.622	1.028	1.008	1.349	1.637	1.213	1.287	1.126
P08107	Heat shock 70 kDa protein 1A/1B OS=Homo sapiens GN=HSPA1A	1.566	2.866	3.492	3.010	1.271	3.076	3.150	3.358
Q9UBI6	Guanine nucleotide-binding protein G(I)/G(S)/G(O) subunit gamm	1.698	1.780	1.448	1.593	1.050	1.309	1.552	1.454
O14548	Cytochrome c oxidase subunit 7A-related protein, mitochondrial C	1.028	1.212	0.934	0.945	0.786	0.990	1.030	1.044
P61970	Nuclear transport factor 2 OS=Homo sapiens GN=NUTF2 PE=1 S	1.185	0.778	1.175	1.088	0.961	0.926	0.930	0.925
P61204	ADP-ribosylation factor 3 OS=Homo sapiens GN=ARF3 PE=1 SV=	1.694	1.413	1.772	1.481	1.561	1.104	1.521	1.428
P78371	T-complex protein 1 subunit beta OS=Homo sapiens GN=CCT2 PI	1.503	1.142	1.295	1.055	1.358	1.114	1.277	1.217
Q13011	Delta(3,5)-Delta(2,4)-dienoyl-CoA isomerase, mitochondrial OS=H	1.221	1.210	1.039	1.161	1.018	1.091	1.158	1.158
A8K4W8	cDNA FLJ77917, highly similar to Homo sapiens ubiquitin-conjuga	1.400	1.729	1.824	1.683	1.672	1.114	1.645	1.756
P52895	Aldo-keto reductase family 1 member C2 OS=Homo sapiens GN=	0.999	0.687	0.923	0.928	0.785	0.802	0.836	0.705
Q7Z4Y4	GTP:AMP phosphotransferase AK3, mitochondrial OS=Homo sapie	1.058	1.140	1.074	1.132	0.969	1.154	1.265	1.129
P07237	Protein disulfide-isomerase OS=Homo sapiens GN=P4HB PE=1 SV	1.455	1.050	1.329	1.368	1.489	1.249	1.451	1.509
P09936	Ubiquitin carboxyl-terminal hydrolase isozyme L1 OS=Homo sapie	1.249	0.881	1.579	1.204	1.011	0.945	1.171	1.123
P68371	Tubulin beta-4B chain OS=Homo sapiens GN=TUBB4B PE=1 SV=	2.109	1.434	2.286		2.006	0.955	1.768	1.924
Q9Y5J7	Mitochondrial import inner membrane translocase subunit Tim9 C	1.858	1.766	1.585	1.831	1.589	1.287	1.682	1.867
P04083	Annexin A1 OS=Homo sapiens GN=ANXA1 PE=1 SV=2 - [ANXA1_	1.154	0.604	0.948	0.740	1.173	0.914	0.917	0.819
P50990	T-complex protein 1 subunit theta OS=Homo sapiens GN=CCT8 F	1.456	1.158	1.387	1.088	1.309	1.013	1.226	1.173
Q03135	Caveolin-1 OS=Homo sapiens GN=CAV1 PE=1 SV=4 - [CAV1_HU	1.146	1.240	0.929	0.833	1.102	1.225	1.098	0.916
P37235	Hippocalcin-like protein 1 OS=Homo sapiens GN=HPCAL1 PE=1 S	3.072	2.851	3.245	2.523	3.014	1.696	2.685	2.587
A8K486	Peptidyl-prolyl cis-trans isomerase OS=Homo sapiens PE=2 SV=1	1.382	0.936	1.442	1.212	1.179	0.951	1.160	1.161
P30050	60S ribosomal protein L12 OS=Homo sapiens GN=RPL12 PE=1 S	1.600	1.342	1.593	0.991	1.432	1.245	1.304	1.224
A8K690	cDNA FLJ76863, highly similar to Homo sapiens stress-induced-pl	1.640	1.267	1.546	1.228	1.401	1.162	1.496	1.389
P04406	Glyceraldehyde-3-phosphate dehydrogenase OS=Homo sapiens G	1.283	0.867	1.253	1.009	1.239	0.972	1.130	1.094
P55072	Transitional endoplasmic reticulum ATPase OS=Homo sapiens GN	1.455	1.081	1.198	0.920	1.473	1.257	1.208	1.025
G5E9W7	28S ribosomal protein S22, mitochondrial OS=Homo sapiens GN=	1.078	1.220	0.992	1.046	0.892	1.146	1.176	1.104

P25786	Proteasome subunit alpha type-1 OS=Homo sapiens GN=PSMA1	1.400	0.979	1.104	0.813	1.289	1.123	1.114	1.003
Q14019	Coactosin-like protein OS=Homo sapiens GN=COTL1 PE=1 SV=3	1.223	0.638	1.172	0.846	0.792	0.898	0.711	0.738
Q07065	Cytoskeleton-associated protein 4 OS=Homo sapiens GN=CKAP4	1.149	1.237	1.043	0.983	0.902	1.136	1.150	1.005
Q13813	Spectrin alpha chain, non-erythrocytic 1 OS=Homo sapiens GN=S	1.557	1.689	1.207	0.953	1.440	1.393	1.215	1.308
Q14847	LIM and SH3 domain protein 1 OS=Homo sapiens GN=LASP1 PE=	1.499	0.860	1.067	0.796	1.360	0.893	1.009	0.907
P61224	Ras-related protein Rap-1b OS=Homo sapiens GN=RAP1B PE=1 S	1.065	1.408	0.839	0.849	0.921	1.143	1.077	1.108
P12537	Peripentonal hexon-associated protein preIIIa GN=PreIIIA - [HEX	1.568	1.589	7.284	9.049	1.106	1.398	2.800	3.439
Q01082	Spectrin beta chain, non-erythrocytic 1 OS=Homo sapiens GN=SI	1.603	1.674	1.250	0.977	1.510	1.349	1.301	1.346
Q15149	Plectin OS=Homo sapiens GN=PLEC PE=1 SV=3 - [PLEC_HUMAN	1.451	1.702	1.299	1.183	1.309	1.324	1.397	1.357
P49411	Elongation factor Tu, mitochondrial OS=Homo sapiens GN=TUFM	1.079	1.130	0.992	1.013	0.956	1.112	1.099	1.055
P04133	Hexon protein GN=Hexon_pII - [HEX_ADE05]	1.492	1.590	3.606	4.959	1.139	1.037	1.487	2.644
P21333	Filamin-A OS=Homo sapiens GN=FLNA PE=1 SV=4 - [FLNA_HUM	2.117	1.485	1.475	1.048	1.849	1.265	1.403	1.314
Q9BTQ7	Similar to ribosomal protein L23 (Fragment) OS=Homo sapiens P	1.458	1.274	1.439	1.200	1.225	1.152	1.345	1.097
O00488	Zinc finger protein 593 OS=Homo sapiens GN=ZNF593 PE=1 SV=	1.832	2.630	2.189	1.261	1.860	2.199	2.233	2.038
O75369	Filamin-B OS=Homo sapiens GN=FLNB PE=1 SV=2 - [FLNB_HUM	1.847	1.320	1.247	0.871	1.736	1.215	1.164	1.090
P61326	Protein mago nashi homolog OS=Homo sapiens GN=MAGOH PE=	1.321	1.420	1.184	0.963	1.442	1.293	1.424	1.374
B5MC59	Replication protein A 14 kDa subunit OS=Homo sapiens GN=RPA	2.614	3.293	4.121	3.861	1.601	1.326	1.977	2.422
Q9NP72	Ras-related protein Rab-18 OS=Homo sapiens GN=RAB18 PE=1 S	1.254	1.466	1.159	1.072	1.076	1.333	1.346	1.240
P62888	60S ribosomal protein L30 OS=Homo sapiens GN=RPL30 PE=1 S	1.572	1.308	1.463	0.938	1.391	1.273	1.197	1.133
P62316	Small nuclear ribonucleoprotein Sm D2 OS=Homo sapiens GN=SI	1.823	1.758	1.389	1.128	1.811	1.413	1.705	1.456
P39019	40S ribosomal protein S19 OS=Homo sapiens GN=RPS19 PE=1 S	1.442	1.490	1.477	0.993	1.279	1.245	1.267	1.166
Q9UI09	NADH dehydrogenase [ubiquinone] 1 alpha subcomplex subunit 1	1.302	1.471	1.347	1.229	0.946	1.203	1.243	1.101
B3KX11	T-complex protein 1 subunit gamma OS=Homo sapiens PE=2 SV=	1.322	1.047	1.139	0.856	1.263	0.967	1.142	0.901
J3KPX7	Prohibitin-2 OS=Homo sapiens GN=PHB2 PE=1 SV=2 - [J3KPX7_	1.247	1.324	1.063	1.036	0.998	1.112	1.233	1.121
A0A024R5Z9	Pyruvate kinase OS=Homo sapiens GN=PKM2 PE=3 SV=1 - [A0A	1.423	0.866	1.483	1.246	1.269	1.028	1.254	1.135
B3KQT9	Protein disulfide-isomerase OS=Homo sapiens PE=2 SV=1 - [B3K	1.459	1.154	1.416	1.444	1.384	1.189	1.381	1.471
Q9NQR4	Omega-amidase NIT2 OS=Homo sapiens GN=NIT2 PE=1 SV=1 -	1.393	1.041	1.269	1.052	1.091	1.062	1.273	1.134
P10599	Thioredoxin OS=Homo sapiens GN=TXN PE=1 SV=3 - [THIO_HU	1.350	0.896	1.519	1.450	1.168	0.961	1.263	1.286
P52272	Heterogeneous nuclear ribonucleoprotein M OS=Homo sapiens G	1.291	1.396	1.030	0.849	1.374	1.226	1.167	0.872
P29692	Elongation factor 1-delta OS=Homo sapiens GN=EEF1D PE=1 SV	1.281	1.036	1.293	1.009	1.170	1.175	1.089	1.203
A0A024QZN4	Vinculin, isoform CRA_c OS=Homo sapiens GN=VCL PE=4 SV=1	1.810	1.168	1.624	1.346	1.490	1.075	1.462	1.311
P12236	ADP/ATP translocase 3 OS=Homo sapiens GN=SLC25A6 PE=1 SV	1.472	1.682	1.155	1.227	0.863	1.223	1.453	1.214
P45880	Voltage-dependent anion-selective channel protein 2 OS=Homo s	1.450	1.641	1.342	1.336	1.258	1.198	1.489	1.396
P28074	Proteasome subunit beta type-5 OS=Homo sapiens GN=PSMB5 P	1.239	1.002	1.075	0.843	1.223	1.070	1.035	0.850
P06576	ATP synthase subunit beta, mitochondrial OS=Homo sapiens GN=	1.163	1.272	1.033	1.079	0.932	1.097	1.044	1.103
P15121	Aldose reductase OS=Homo sapiens GN=AKR1B1 PE=1 SV=3 - [A	1.209	0.870	1.249	1.042	1.084	0.933	1.009	1.015
P09525	Annexin A4 OS=Homo sapiens GN=ANXA4 PE=1 SV=4 - [ANXA4_	1.273	0.867	1.422	1.224	1.167	0.976	1.410	1.300
P54920	Alpha-soluble NSF attachment protein OS=Homo sapiens GN=NA	2.556	1.760	2.271	1.761	2.017	1.325	2.234	2.188
Q9Y277	Voltage-dependent anion-selective channel protein 3 OS=Homo s	1.279	1.441	1.177	1.184	0.986	1.247	1.296	1.222
A0A024RB99	Serine hydroxymethyltransferase OS=Homo sapiens GN=SHMT2	1.309	1.350	1.162	1.169	1.036	1.129	1.343	1.293

P06493	Cyclin-dependent kinase 1 OS=Homo sapiens GN=CDK1 PE=1 SV	1.639	1.352	1.750	1.353	1.403	1.340	1.872	2.146
Q05DH1	Proteasome subunit alpha type (Fragment) OS=Homo sapiens GN	1.347	1.007	1.101	0.838	1.376	1.125	1.105	0.901
Q01469	Fatty acid-binding protein, epidermal OS=Homo sapiens GN=FAB	2.968	2.588	2.865	2.580	1.879	0.831	1.752	2.507
U3KQ85	Protein C10 OS=Homo sapiens GN=C12orf57 PE=1 SV=1 - [U3K	2.216	1.626	2.525	1.900	1.819	1.274	1.949	1.747
Q8WVW5	Putative uncharacterized protein (Fragment) OS=Homo sapiens P	1.153	1.052	0.861	0.865	0.945	1.191	0.905	0.828
Q9BYD1	39S ribosomal protein L13, mitochondrial OS=Homo sapiens GN=	1.190	1.305	1.257	1.056	1.023	1.126	1.292	1.270
B7Z4V2	cDNA FLJ51907, highly similar to Stress-70 protein, mitochondrial	1.658	1.620	1.437	1.470	1.314	1.276	1.533	1.414
P04632	Calpain small subunit 1 OS=Homo sapiens GN=CAPNS1 PE=1 SV	1.724	0.939	1.543	1.209	1.672	1.164	1.414	0.971
Q6FH24	VBP1 protein OS=Homo sapiens GN=VBP1 PE=2 SV=1 - [Q6FH2	1.443	1.077	1.464	1.161	1.342	1.087	1.373	1.344
P50454	Serpin H1 OS=Homo sapiens GN=SERPINH1 PE=1 SV=2 - [SERP	1.353	1.008	1.200	1.219	1.234	1.169	1.302	1.196
F8W1R7	Retinal cone rhodopsin-sensitive cGMP 3',5'-cyclic phosphodiester	1.467	0.914	1.006	0.742	1.379	1.028	1.089	1.000
Q5TCU3	Tropomyosin beta chain OS=Homo sapiens GN=TPM2 PE=1 SV=	1.530	1.239	1.280	0.991	1.304	1.299	1.291	1.192
P62136	Serine/threonine-protein phosphatase PP1-alpha catalytic subunit	1.146	1.178	0.995	0.917	1.080	1.222	1.194	1.113
P35998	26S protease regulatory subunit 7 OS=Homo sapiens GN=PSMC2	1.521	1.117	1.315	0.914	1.545	1.267	1.268	0.985
P28838	Cytosol aminopeptidase OS=Homo sapiens GN=LAP3 PE=1 SV=3	1.321	1.259	1.304	1.288	1.031	1.091	1.182	1.233
P51148	Ras-related protein Rab-5C OS=Homo sapiens GN=RAB5C PE=1	1.161	1.213	1.238	1.082	1.001	1.200	1.172	1.123
Q9UKD2	mRNA turnover protein 4 homolog OS=Homo sapiens GN=MRTO	1.890	1.840	1.435	1.312	1.839	1.513	1.805	1.485
P24936	Hexon-associated protein preVIII GN=preVIII - [HEX8_ADE05]	1.799	1.895	2.498	3.724	1.861	1.554	2.159	2.510
Q8N5N7	39S ribosomal protein L50, mitochondrial OS=Homo sapiens GN=	1.255	1.272	1.018	1.136	0.943	1.126	1.133	1.096
P63167	Dynein light chain 1, cytoplasmic OS=Homo sapiens GN=DYNLL1	1.678	1.456	1.762	1.711	1.645	1.146	1.820	2.204
C9J9K3	40S ribosomal protein SA (Fragment) OS=Homo sapiens GN=RPS	1.671	1.550	1.699	1.348	1.670	1.289	1.564	1.430
Q6IBN1	HNRPK protein OS=Homo sapiens GN=HNRPK PE=2 SV=1 - [Q6I	1.435	1.235	1.190	0.875	1.488	1.372	1.259	0.956
Q6IB68	MYCBP protein OS=Homo sapiens GN=MYCBP PE=2 SV=1 - [Q6I	1.300	1.246	1.084	0.899	1.181	1.408	1.302	1.148
Q53HV2	Chaperonin containing TCP1, subunit 7 (Eta) variant (Fragment) (	1.520	1.050	1.222	0.937	1.194	1.018	1.160	1.040
P55084	Trifunctional enzyme subunit beta, mitochondrial OS=Homo sapie	1.204	1.315	1.058	1.023	0.958	1.184	1.099	1.060
B4DEN6	cDNA FLJ55549, highly similar to 3-ketoacyl-CoA thiolase, peroxis	1.284	1.236	1.204	1.100	1.441	1.321	1.338	1.246
P40939	Trifunctional enzyme subunit alpha, mitochondrial OS=Homo sapi	1.135	1.201	1.008	0.969	0.952	1.120	1.106	1.017
Q15004	PCNA-associated factor OS=Homo sapiens GN=KIAA0101 PE=1 S	1.403	2.530	2.552	1.944	1.352	2.053	2.219	2.104
O14737	Programmed cell death protein 5 OS=Homo sapiens GN=PDCD5	1.381	0.780	1.125	0.909	1.150	0.920	1.194	1.319
Q53G71	Calreticulin variant (Fragment) OS=Homo sapiens PE=2 SV=1 - [	1.619	1.142	1.511	1.507	1.561	1.235	1.479	1.614
P52907	F-actin-capping protein subunit alpha-1 OS=Homo sapiens GN=C	3.274	2.876	1.810	1.547	1.797	1.230	1.561	1.467
P24933	Late 100 kDa protein GN=100K - [L100_ADE05]	1.531	1.179	3.572	2.571	1.125	1.254	2.434	3.904
Q02790	Peptidyl-prolyl cis-trans isomerase FKBP4 OS=Homo sapiens GN=	1.675	1.119	1.666	1.244	1.385	1.075	1.377	1.343
P14550	Alcohol dehydrogenase [NADP(+)] OS=Homo sapiens GN=AKR1A	0.863	0.720	1.057	0.813	0.954	0.942	0.980	0.905
A8K766	cDNA FLJ77343, highly similar to Homo sapiens electron-transfer-	1.087	1.089	1.007	1.009	0.933	1.073	1.082	1.068
P19784	Casein kinase II subunit alpha' OS=Homo sapiens GN=CSNK2A2	1.447	1.472	1.589	1.378	1.423	1.221	1.608	1.748
Q7L0Y3	Mitochondrial ribonuclease P protein 1 OS=Homo sapiens GN=TR	1.381	1.536	1.161	1.047	1.081	1.266	1.314	1.202
Q9Y3E5	Peptidyl-tRNA hydrolase 2, mitochondrial OS=Homo sapiens GN=	1.257	1.181	1.180	1.040	0.997	1.063	1.169	0.961
B2R6X6	Peptidyl-prolyl cis-trans isomerase OS=Homo sapiens PE=2 SV=1	1.125	1.373	1.137	1.087	0.992	1.340	1.197	1.336
Q53H82	Beta-lactamase-like protein 2 OS=Homo sapiens GN=LACTB2 PE=	1.488	1.132	1.426	1.156	1.328	1.162	1.250	1.243

P62805	Histone H4 OS=Homo sapiens GN=HIST1H4A PE=1 SV=2 - [H4_	1.191	1.576	1.120	1.158	0.900	1.051	1.158	1.373
Q9Y6N5	Sulfide:quinone oxidoreductase, mitochondrial OS=Homo sapiens	1.351	1.443	1.134	1.139	0.997	0.999	1.106	1.089
Q9Y221	60S ribosome subunit biogenesis protein NIP7 homolog OS=Hom	2.426	2.484	2.167	2.157	2.317	1.554	2.392	2.303
P22061	Protein-L-isoaspartate(D-aspartate) O-methyltransferase OS=Hon	1.583	1.086	1.449	1.205	1.380	1.117	1.419	1.360
P61158	Actin-related protein 3 OS=Homo sapiens GN=ACTR3 PE=1 SV=3	1.254	1.192	1.170	0.982	1.325	1.151	1.178	1.116
P68104	Elongation factor 1-alpha 1 OS=Homo sapiens GN=EEF1A1 PE=1	1.646	1.332	1.959	1.245	1.614	1.179	1.791	1.278
P48047	ATP synthase subunit O, mitochondrial OS=Homo sapiens GN=AT	1.187	1.212	1.079	1.082	0.913	1.104	1.145	1.135
P08729	Keratin, type II cytoskeletal 7 OS=Homo sapiens GN=KRT7 PE=1	1.452	1.719	1.145	0.774	1.285	1.197	1.191	0.822
C9J0J7	Profilin-2 OS=Homo sapiens GN=PFN2 PE=1 SV=1 - [C9J0J7_HU	1.275	0.668	1.105	0.840	1.108	0.896	1.058	0.977
P62249	40S ribosomal protein S16 OS=Homo sapiens GN=RPS16 PE=1 S	1.766	1.774	1.678	1.068	1.507	1.391	1.570	1.347
P61019	Ras-related protein Rab-2A OS=Homo sapiens GN=RAB2A PE=1	1.190	1.305	1.051	1.016	1.019	1.141	1.170	1.034
P46783	40S ribosomal protein S10 OS=Homo sapiens GN=RPS10 PE=1 S	1.676	1.538	1.621	0.986	1.383	1.223	1.497	1.274
B4DY09	Interleukin enhancer-binding factor 2 OS=Homo sapiens GN=ILF	1.427	1.370	1.011	0.931	1.408	1.242	1.253	0.975
P50395	Rab GDP dissociation inhibitor beta OS=Homo sapiens GN=GDI2	1.376	0.879	1.267	1.198	1.250	1.083	1.345	1.269
O60701	UDP-glucose 6-dehydrogenase OS=Homo sapiens GN=UGDH PE=	1.122	0.809	1.053	0.771	1.086	0.948	0.871	0.783
P62701	40S ribosomal protein S4, X isoform OS=Homo sapiens GN=RPS4	1.873	1.648	1.628	1.063	1.566	1.356	1.428	1.476
E4W6B6	RPL27/NME2 fusion protein (Fragment) OS=Homo sapiens GN=R	1.430	1.262	1.539	1.037	1.378	1.192	1.390	1.222
H7C613	Dehydrogenase/reductase SDR family member on chromosome X	1.224	1.267	1.051	1.173	1.401	1.106	1.298	1.205
V9HWC6	Peptidyl-prolyl cis-trans isomerase OS=Homo sapiens GN=HEL-S-	1.470	1.113	1.391	1.231	1.376	1.226	1.437	1.343
P56211	cAMP-regulated phosphoprotein 19 OS=Homo sapiens GN=ARPP	0.838	0.570	0.744	0.537	0.857	1.040	0.892	0.665
P56277	Cx9C motif-containing protein 4 OS=Homo sapiens GN=CMC4 PE	1.175	1.463	1.191	1.031	1.347	1.231	1.449	1.263
P29401	Transketolase OS=Homo sapiens GN=TKT PE=1 SV=3 - [TKT_HU	1.019	0.849	0.986	0.812	1.058	0.937	0.843	0.783
P07108	Acyl-CoA-binding protein OS=Homo sapiens GN=DBI PE=1 SV=2	3.650	2.277	4.225	3.546	2.685	1.213	3.498	3.494
X1WI28	60S ribosomal protein L10 (Fragment) OS=Homo sapiens GN=RP	1.828	1.415	1.545	1.361	1.428	1.422	1.311	1.373
H3BSC1	Ras-related protein Rab-11A OS=Homo sapiens GN=RAB11A PE=	3.730	3.960	3.726	3.563	3.058	1.727	3.638	3.387
A8K6D2	cDNA FLJ76620, highly similar to Homo sapiens C2f protein (C2F)	1.389	1.673	1.255	0.941	1.363	1.234	1.406	1.141
E7EQ69	N-alpha-acetyltransferase 50 OS=Homo sapiens GN=NAA50 PE=1	2.269	1.682	2.071	1.534	1.865	1.302	2.022	1.687
P34932	Heat shock 70 kDa protein 4 OS=Homo sapiens GN=HSPA4 PE=1	1.378	1.076	1.267	0.980	1.280	1.142	1.168	1.101
O95433	Activator of 90 kDa heat shock protein ATPase homolog 1 OS=Ho	1.588	1.127	1.892	1.496	1.327	1.110	1.512	1.500
P13667	Protein disulfide-isomerase A4 OS=Homo sapiens GN=PDIA4 PE=	1.429	1.051	1.290	1.308	1.456	1.172	1.365	1.376
E9PKG1	Protein arginine N-methyltransferase 1 OS=Homo sapiens GN=PF	1.485	1.246	1.165	1.004	1.683	1.339	1.296	0.988
O75955	Flotillin-1 OS=Homo sapiens GN=FLOT1 PE=1 SV=3 - [FLOT1_HU	1.158	1.362	0.965	1.050	1.044	1.092	1.048	1.004
Q8N4H5	Mitochondrial import receptor subunit TOM5 homolog OS=Homo	1.331	1.898	1.454	1.270	1.175	1.266	1.617	1.332
P50991	T-complex protein 1 subunit delta OS=Homo sapiens GN=CCT4 P	1.508	1.094	1.385	1.098	1.371	1.053	1.270	1.118
O00299	Chloride intracellular channel protein 1 OS=Homo sapiens GN=CL	1.132	0.743	1.027	0.827	0.963	0.873	0.850	0.835
Q15233	Non-POU domain-containing octamer-binding protein OS=Homo s	1.242	1.516	1.201	1.075	1.331	1.372	1.369	1.180
Mature	VI GN=matVI	1.069	1.505	2.540	4.170	1.252	1.320	0.957	1.892
P18669	Phosphoglycerate mutase 1 OS=Homo sapiens GN=PGAM1 PE=1	1.404	0.970	1.484	1.170	1.127	1.010	1.282	1.186
Q9BRX5	DNA replication complex GINS protein PSF3 OS=Homo sapiens G	1.387	1.051	1.245	0.891	1.369	1.081	1.325	1.084
Q96EL3	39S ribosomal protein L53, mitochondrial OS=Homo sapiens GN=	1.556	1.534	1.247	1.152	1.339	1.356	1.409	1.307

Q9NPE3	H/ACA ribonucleoprotein complex subunit 3 OS=Homo sapiens G	1.393	1.502	1.250	1.416	1.314	1.437	1.181	1.337
P16402	Histone H1.3 OS=Homo sapiens GN=HIST1H1D PE=1 SV=2 - [H	1.214	2.144	1.257	1.437	0.902	0.851	0.827	1.338
Q9UKY7	Protein CDV3 homolog OS=Homo sapiens GN=CDV3 PE=1 SV=1	1.343	1.023	1.225	0.838	1.194	1.000	1.150	1.029
Q9UI30	Multifunctional methyltransferase subunit TRM112-like protein OS	1.128	0.934	1.202	0.998	0.979	1.101	1.217	1.083
B7Z2S5	cDNA FLJ56075, highly similar to Thioredoxin reductase 1, cytopl	1.616	1.153	1.556	1.121	1.417	1.070	1.231	1.113
P61106	Ras-related protein Rab-14 OS=Homo sapiens GN=RAB14 PE=1 S	1.612	1.444	1.359	1.312	1.232	1.266	1.406	1.294
Q8TDN6	Ribosome biogenesis protein BRX1 homolog OS=Homo sapiens G	1.624	1.834	1.335	1.203	1.555	1.343	1.489	1.628
V9HW25	Epididymis secretory protein Li 273 OS=Homo sapiens GN=HEL-S	1.737	1.265	1.370	1.071	1.532	1.386	1.470	1.291
P56385	ATP synthase subunit e, mitochondrial OS=Homo sapiens GN=AT	1.331	1.625	1.147	1.299	0.932	0.958	1.383	1.381
B1AK87	Capping protein (Actin filament) muscle Z-line, beta, isoform CRA	1.224	1.182	1.106	1.031	1.266	1.123	1.069	1.047
B5MCX3	Septin-2 OS=Homo sapiens GN=SEPT2 PE=1 SV=1 - [B5MCX3_H	1.552	1.051	1.348	0.912	1.398	1.056	1.230	1.248
Q96BS4	FBL protein (Fragment) OS=Homo sapiens GN=FBL PE=1 SV=2 -	1.529	1.765	1.451	1.368	1.570	1.439	1.663	1.603
P11021	78 kDa glucose-regulated protein OS=Homo sapiens GN=HSPA5	1.413	1.104	1.246	1.189	1.322	1.183	1.244	1.235
B4DM33	cDNA FLJ52068, highly similar to Microtubule-associated protein	1.462	0.878	1.407	0.938	1.165	1.055	1.399	1.024
P42704	Leucine-rich PPR motif-containing protein, mitochondrial OS=Hon	1.366	1.484	1.233	1.175	1.110	1.162	1.355	1.249
O00231	26S proteasome non-ATPase regulatory subunit 11 OS=Homo sap	1.694	1.123	1.348	0.967	1.566	1.135	1.177	0.924
Q86UP2	Kinectin OS=Homo sapiens GN=KTN1 PE=1 SV=1 - [KTN1_HUM	1.010	1.478	0.899	0.867	0.975	1.130	0.958	1.145
P62191	26S protease regulatory subunit 4 OS=Homo sapiens GN=PSMC1	1.358	1.120	1.141	0.788	1.322	1.110	0.994	0.873
P24938	Minor core protein V GN=PV - [VCOM_ADE05]	1.340	1.510	3.038	5.884	1.282	1.422	2.509	5.240
B4DEH8	Polyadenylate-binding protein 2 OS=Homo sapiens GN=PABPN1	1.269	1.353	1.095	0.794	1.291	1.325	1.247	1.094
Q9NZM1	Myoferlin OS=Homo sapiens GN=MYOF PE=1 SV=1 - [MYOF_HU	1.325	1.329	1.015	1.048	1.154	1.165	1.034	0.965
P41223	Protein BUD31 homolog OS=Homo sapiens GN=BUD31 PE=1 SV	1.315	1.410	1.271	0.905	1.324	1.429	1.189	0.954
P30040	Endoplasmic reticulum resident protein 29 OS=Homo sapiens GN	1.465	1.187	1.334	1.312	1.527	1.186	1.337	1.577
B4E0X8	cDNA FLJ61021, highly similar to Far upstream element-binding p	1.373	1.396	0.902	0.363	0.994	1.090	0.941	0.954
Q9BU08	Putative uncharacterized protein (Fragment) OS=Homo sapiens P	1.341	0.999	1.197	0.999	1.190	1.003	1.130	1.043
A8K6Y1	cDNA FLJ75526, highly similar to Homo sapiens proliferation-assc	1.707	1.392	1.615	1.105	1.562	1.138	1.420	1.200
Q9BRP8	Partner of Y14 and mago OS=Homo sapiens GN=WIBG PE=1 SV	1.349	1.065	1.356	1.038	0.737	1.058	1.050	1.048
Q13162	Peroxiredoxin-4 OS=Homo sapiens GN=PRDX4 PE=1 SV=1 - [PR	2.273	1.542	2.076	2.050	2.184	1.519	2.105	2.199
P04080	Cystatin-B OS=Homo sapiens GN=CSTB PE=1 SV=2 - [CYTB_HU	3.042	1.868	3.226	2.429	1.712	0.818	2.049	2.514
P20290	Transcription factor BTF3 OS=Homo sapiens GN=BTF3 PE=1 SV=	1.692	1.530	1.476	1.195	1.129	1.030	0.874	1.343
B4DIZ2	cDNA FLJ57995, moderately similar to Ubiquitin-conjugating enzy	1.314	0.870	1.226	1.190	1.097	1.025	1.181	1.227
B2R5M8	Isocitrate dehydrogenase [NADP] OS=Homo sapiens PE=2 SV=1	1.085	0.855	1.218	0.983	1.075	1.017	1.106	0.989
P00441	Superoxide dismutase [Cu-Zn] OS=Homo sapiens GN=SOD1 PE=	1.097	0.833	1.164	1.251	0.998	0.961	1.160	1.116
P27695	DNA-(apurinic or apyrimidinic site) lyase OS=Homo sapiens GN=	1.320	1.292	1.238	1.018	1.417	1.063	1.108	1.011
Q9H7Z7	Prostaglandin E synthase 2 OS=Homo sapiens GN=PTGES2 PE=1	1.405	1.367	1.060	1.042	1.358	1.250	1.234	1.298
B4DY28	cDNA FLJ61189, highly similar to Cysteine and glycine-rich protei	7.073	4.861	5.692	4.001	2.066	0.940	1.792	2.115
P62906	60S ribosomal protein L10a OS=Homo sapiens GN=RPL10A PE=1	1.443	1.396	1.336	0.958	1.325	1.335	1.345	1.176
G3V0I5	NADH dehydrogenase (Ubiquinone) flavoprotein 1, 51kDa, isoforr	1.034	0.980	0.929	0.854	0.869	1.183	1.137	1.117
B4DW52	cDNA FLJ55253, highly similar to Actin, cytoplasmic 1 OS=Homo	1.350	1.222	1.246	1.137	1.354	1.351	1.332	1.172
P62280	40S ribosomal protein S11 OS=Homo sapiens GN=RPS11 PE=1 S	1.628	1.542	1.556	1.034	1.380	1.215	1.376	1.193

P18085	ADP-ribosylation factor 4 OS=Homo sapiens GN=ARF4 PE=1 SV=	2.411	1.773	2.130	1.529	2.140	1.454	1.904	1.663
P25789	Proteasome subunit alpha type-4 OS=Homo sapiens GN=PSMA4	1.711	1.245	1.435	1.161	1.652	1.164	1.300	1.279
A0A024R4X0	Cytochrome b5 reductase 3, isoform CRA_a OS=Homo sapiens G	2.580	2.314	2.204	2.563	1.987	1.421	2.449	2.252
Q16891	MICOS complex subunit MIC60 OS=Homo sapiens GN=IMMT PE=	1.396	1.415	1.246	1.326	1.112	1.162	1.275	1.350
Q96AE4	Far upstream element-binding protein 1 OS=Homo sapiens GN=F	0.985	1.044	1.043	0.967	0.969	1.304	1.024	
Q8WUH6	Transmembrane protein 263 OS=Homo sapiens GN=TMEM263 PE	1.556	1.076	1.517	1.008	1.294	0.869	1.670	1.405
O15511	Actin-related protein 2/3 complex subunit 5 OS=Homo sapiens GI	1.156	0.982	1.142	0.901	1.127	0.989	1.021	0.894
O75964	ATP synthase subunit g, mitochondrial OS=Homo sapiens GN=AT	1.000	0.967	0.894	1.067	0.674	0.930	1.034	1.022
Q8WYJ5	Protein kinase C inhibitor-2 OS=Homo sapiens PE=2 SV=1 - [Q8V	1.083	1.163	0.961	0.996	0.887	1.083	1.111	1.054
P12004	Proliferating cell nuclear antigen OS=Homo sapiens GN=PCNA PE	1.301	1.241	1.749	1.540	1.296	1.205	1.319	1.525
A8K7F6	cDNA FLJ78244, highly similar to Homo sapiens eukaryotic transla	1.970	1.641	2.232	1.633	1.564	1.258	1.529	1.799
P18124	60S ribosomal protein L7 OS=Homo sapiens GN=RPL7 PE=1 SV=	1.729	1.630	1.492	1.370	1.626	1.333	1.573	1.627
P14678	Small nuclear ribonucleoprotein-associated proteins B and B' OS=	1.016	1.206			0.910	1.193	1.274	
P61026	Ras-related protein Rab-10 OS=Homo sapiens GN=RAB10 PE=1 S	1.600	1.460	1.302	1.159	1.436	1.274	1.388	1.185
P54652	Heat shock-related 70 kDa protein 2 OS=Homo sapiens GN=HSP/	1.316	1.090	1.095	0.989	1.109	1.120	1.133	1.037
Q96AG4	Leucine-rich repeat-containing protein 59 OS=Homo sapiens GN=	1.266	1.394	1.237	0.995	0.968	1.171	1.305	1.139
A8KA83	cDNA FLJ78586, highly similar to Homo sapiens VAMP (vesicle-as	1.179	1.307	0.969	0.835	0.986	1.158	1.072	0.852
B4DZ87	cDNA FLJ57240, highly similar to Mitochondrial proteins import re	1.480	1.629	1.331	1.253	1.188	1.256	1.553	1.399
P09493	Tropomyosin alpha-1 chain OS=Homo sapiens GN=TPM1 PE=1 S	3.458	2.500	2.105	1.559	2.917	1.581	2.016	1.808
Q9Y2Q3	Glutathione S-transferase kappa 1 OS=Homo sapiens GN=GSTK1	1.414	1.501	1.336	1.266	1.298	1.254	1.557	1.525
P12235	ADP/ATP translocase 1 OS=Homo sapiens GN=SLC25A4 PE=1 SV	1.542	2.004	1.772	1.709	1.225	1.540	2.011	1.957
P14868	Aspartate--tRNA ligase, cytoplasmic OS=Homo sapiens GN=DARS	1.313	1.227	1.313	1.049	1.286	1.091	1.225	1.178
B3KQF5	cDNA FLJ90381 fis, clone NT2RP2005035, highly similar to Calum	1.578	1.181	1.402	1.548	1.560	1.296	1.460	1.616
P62333	26S protease regulatory subunit 10B OS=Homo sapiens GN=PSM	1.519	1.095	1.217	0.868	1.446	1.066	1.086	1.026
Q15365	Poly(rC)-binding protein 1 OS=Homo sapiens GN=PCBP1 PE=1 S	1.393	1.133	1.314	0.989	1.363	1.052	1.208	1.129
Q9BV61	TRAP1 protein (Fragment) OS=Homo sapiens GN=TRAP1 PE=2 S	0.987	1.038	0.883	0.909	0.899	1.057	1.071	1.076
P13804	Electron transfer flavoprotein subunit alpha, mitochondrial OS=H	1.291	1.302	1.233	1.252	1.090	1.145	1.318	1.365
A9CQZ4	Dihydropyrimidinase-like 2 long form (Fragment) OS=Homo sapie	1.136	1.005	1.452	1.011	1.226	0.814	1.025	1.112
Q9NR30	Nucleolar RNA helicase 2 OS=Homo sapiens GN=DDX21 PE=1 S	1.807	2.315	1.784	1.580	1.557	1.497	1.823	1.523
P19623	Spermidine synthase OS=Homo sapiens GN=SRM PE=1 SV=1 - [	1.047	0.826	1.086	1.051	0.948	0.912	1.045	1.088
P36578	60S ribosomal protein L4 OS=Homo sapiens GN=RPL4 PE=1 SV=	1.585	1.413	1.387	0.999	1.536	1.321	1.474	1.309
Q53GA7	Tubulin alpha 6 variant (Fragment) OS=Homo sapiens PE=2 SV=	1.555	0.983	1.475	1.151	1.289	1.004	1.191	1.178
P35249	Replication factor C subunit 4 OS=Homo sapiens GN=RFC4 PE=1	1.479	1.430	1.372	1.375	1.233	1.189	1.212	1.339
P30519	Heme oxygenase 2 OS=Homo sapiens GN=HMOX2 PE=1 SV=2 -	1.946	2.059	2.084	1.996	1.512	1.416	1.885	1.882
Q15907	Ras-related protein Rab-11B OS=Homo sapiens GN=RAB11B PE=	1.231	1.276	1.297	1.430	1.188	1.135	1.302	1.115
P35579	Myosin-9 OS=Homo sapiens GN=MYH9 PE=1 SV=4 - [MYH9_HU	1.698	0.958	1.094	0.781	1.373	1.036	1.121	1.003
Q99471	Prefoldin subunit 5 OS=Homo sapiens GN=PFDN5 PE=1 SV=2 - [	1.236	0.891	1.156	0.863	1.134	1.027	1.086	0.945
O76021	Ribosomal L1 domain-containing protein 1 OS=Homo sapiens GN	2.091	2.304	1.687	1.512	1.997	1.526	2.076	1.904
P60900	Proteasome subunit alpha type-6 OS=Homo sapiens GN=PSMA6	1.299	1.088	1.111	0.898	1.304	1.150	1.114	0.941
Q00688	Peptidyl-prolyl cis-trans isomerase FKBP3 OS=Homo sapiens GN=	1.142	0.957	1.210	0.841	1.026	1.027	0.996	0.905

P49748	Very long-chain specific acyl-CoA dehydrogenase, mitochondrial (	1.221	1.271	0.980	1.011	1.035	1.145	1.041	1.120
P30533	Alpha-2-macroglobulin receptor-associated protein OS=Homo sap	1.425	1.146	1.495	1.377	1.337	1.360	1.712	1.667
P56381	ATP synthase subunit epsilon, mitochondrial OS=Homo sapiens G	1.958	1.904	1.524	1.471	1.282	1.254	1.645	1.494
P23193	Transcription elongation factor A protein 1 OS=Homo sapiens GN	1.182	0.939	0.998	0.755	1.279	1.085	1.047	0.848
Q96I99	Succinyl-CoA ligase [GDP-forming] subunit beta, mitochondrial OS	1.289	1.349	1.203	1.159	1.038	1.175	1.250	1.205
Q04837	Single-stranded DNA-binding protein, mitochondrial OS=Homo sa	2.110	1.648	1.053	1.511	1.363	1.229	1.368	1.585
A8K7T4	cDNA FLJ75774, highly similar to Homo sapiens lectin, mannose-l	1.466	1.137	0.868	0.607	1.195	0.944	1.051	0.651
P24534	Elongation factor 1-beta OS=Homo sapiens GN=EEF1B2 PE=1 SV	1.464	1.033	1.497	1.188	1.330	1.321	1.302	1.301
Q96HQ2	CDKN2AIP N-terminal-like protein OS=Homo sapiens GN=CDKN2	1.310	1.410	1.018	0.718	1.388	1.198	1.140	0.899
Q9H6F5	Coiled-coil domain-containing protein 86 OS=Homo sapiens GN=(	1.409	1.767	1.830	1.482	1.285	1.477	2.297	1.760
O43678	NADH dehydrogenase [ubiquinone] 1 alpha subcomplex subunit 2	1.456	1.498	1.276	1.370	1.146	1.143	1.395	1.322
H7BZJ3	Protein disulfide-isomerase A3 (Fragment) OS=Homo sapiens GN	1.320	1.086	1.251	1.406	1.505	1.138	1.486	1.507
P09382	Galectin-1 OS=Homo sapiens GN=LGALS1 PE=1 SV=2 - [LEG1_H	1.183	1.108	1.323	1.038	1.083	0.938	1.084	1.133
Q9BTT4	Mediator of RNA polymerase II transcription subunit 10 OS=Homo	1.865	1.930	1.374	0.990	1.765	1.462	1.654	1.305
P16435	NADPH--cytochrome P450 reductase OS=Homo sapiens GN=POR	1.498	1.461	1.340	1.283	1.154	1.338	1.305	1.102
O94905	Erlin-2 OS=Homo sapiens GN=ERLIN2 PE=1 SV=1 - [ERLN2_HUI	1.470	1.434	1.308	1.235	1.142	1.338	1.440	1.205
P26038	Moesin OS=Homo sapiens GN=MSN PE=1 SV=3 - [MOES_HUMAI	1.279	0.956	1.130	0.878	1.069	0.907	1.019	0.958
Q00610	Clathrin heavy chain 1 OS=Homo sapiens GN=CLTC PE=1 SV=5 -	1.438	1.323	1.299	1.095	1.329	1.075	1.246	1.278
B5BU83	Stathmin OS=Homo sapiens GN=STMN1 PE=2 SV=1 - [B5BU83_	1.216	0.784	1.107	0.945	1.146	0.836	1.063	1.007
P18859	ATP synthase-coupling factor 6, mitochondrial OS=Homo sapiens	1.283	1.395	1.253	1.147	1.072	1.186	1.454	1.227
O43399	Tumor protein D54 OS=Homo sapiens GN=TPD52L2 PE=1 SV=2	1.435	1.333	1.465	1.148	1.479	1.044	1.563	1.568
Q13885	Tubulin beta-2A chain OS=Homo sapiens GN=TUBB2A PE=1 SV=	1.579	1.504	1.298	1.344	1.408	1.147	1.526	1.081
P06748	Nucleophosmin OS=Homo sapiens GN=NPM1 PE=1 SV=2 - [NPM	1.383	1.368	1.237	1.184	1.414	1.331	1.305	1.232
P12538	Penton protein GN=penton_base - [PEN3_ADE05]	2.355	2.648	15.296	24.912	1.939	1.993	6.014	23.905
P08758	Annexin A5 OS=Homo sapiens GN=ANXA5 PE=1 SV=2 - [ANXA5_	1.147	0.777	1.122	1.143	1.231	0.977	1.151	1.196
H7BYY1	Tropomyosin 1 (Alpha), isoform CRA_m OS=Homo sapiens GN=T	1.681	1.280	1.107	0.925	1.689	1.239	1.300	1.156
P13639	Elongation factor 2 OS=Homo sapiens GN=EEF2 PE=1 SV=4 - [E	1.472	1.019	1.556	1.201	1.275	1.137	1.376	1.318
Q96I24	Far upstream element-binding protein 3 OS=Homo sapiens GN=F	1.375	1.493	1.005	0.769	1.390	1.263	1.258	1.016
P68366	Tubulin alpha-4A chain OS=Homo sapiens GN=TUBA4A PE=1 SV=	1.690	1.096	1.482	1.223	1.330	1.025	1.371	1.148
A5PLK7	RCC2 protein (Fragment) OS=Homo sapiens GN=RCC2 PE=2 SV=	1.242	0.998	1.059	0.824	1.146	0.843	0.906	1.003
Q92499	ATP-dependent RNA helicase DDX1 OS=Homo sapiens GN=DDX1	1.450	1.491	1.337	1.164	1.384	1.219	1.470	1.213
P54577	Tyrosine--tRNA ligase, cytoplasmic OS=Homo sapiens GN=YARS I	1.584	0.953	1.310	1.029	1.311	1.044	1.231	1.001
P30042	ES1 protein homolog, mitochondrial OS=Homo sapiens GN=C21c	1.131	1.208	1.154	1.061	0.986	1.249	1.233	1.160
Q8WUD4	Coiled-coil domain-containing protein 12 OS=Homo sapiens GN=(	1.371	1.620	1.250	0.896	1.365	1.517	1.431	1.009
Q9Y3B7	39S ribosomal protein L11, mitochondrial OS=Homo sapiens GN=	1.274	1.394	1.153	1.037	0.986	1.171	1.257	1.185
P30043	Flavin reductase (NADPH) OS=Homo sapiens GN=BLVRB PE=1 S	0.999	0.769	1.342	1.143	1.022	1.000	1.006	1.045
Q6IAA8	Ragulator complex protein LAMTOR1 OS=Homo sapiens GN=LAM	2.742	2.288	2.243	1.354	1.743	1.376	2.178	2.361
O15514	DNA-directed RNA polymerase II subunit RPB4 OS=Homo sapiens	2.035	1.923	1.527	1.219	2.050	1.432	1.851	1.596
P11940	Polyadenylate-binding protein 1 OS=Homo sapiens GN=PABPC1 F	1.359	1.388	1.390	1.084	1.311	1.279	1.283	1.301
P62195	26S protease regulatory subunit 8 OS=Homo sapiens GN=PSMC5	1.276	0.962	1.006	0.763	1.233	1.089	0.984	0.818

P12814	Alpha-actinin-1 OS=Homo sapiens GN=ACTN1 PE=1 SV=2 - [ACT	1.643	1.201	1.254	0.952	1.553	1.192	1.326	1.360
Q9NV31	U3 small nucleolar ribonucleoprotein protein IMP3 OS=Homo sap	1.591	1.538	1.134	0.997	1.552	1.364	1.353	1.189
O75340	Programmed cell death protein 6 OS=Homo sapiens GN=PDCD6	1.487	1.329	1.430	1.340	1.439	1.221	1.474	1.486
P62312	U6 snRNA-associated Sm-like protein LSm6 OS=Homo sapiens GN	1.382	1.292	1.071	0.832	1.289	1.235	1.172	0.967
Q9HB71	Calcyclin-binding protein OS=Homo sapiens GN=CACYBP PE=1 S	1.814	1.228	1.786	1.326	1.495	1.145	1.709	1.693
Q86Y39	NADH dehydrogenase [ubiquinone] 1 alpha subcomplex subunit 1	1.419	1.191	1.038	0.883	1.085	0.960	1.202	1.266
A0A024R201	Proteasome (Prosome, macropain) 26S subunit, non-ATPase, 13,	1.566	1.124	1.187	0.984	1.417	1.149	1.171	0.985
Q9Y3D9	28S ribosomal protein S23, mitochondrial OS=Homo sapiens GN=	1.985	2.118	1.993	2.090	1.538	1.204	1.673	1.828
A0A024RDQ0	Heat shock 105kDa/110kDa protein 1, isoform CRA_a OS=Homo	1.590	1.311	1.569	1.145	1.422	1.279	1.490	1.363
Q15738	Sterol-4-alpha-carboxylate 3-dehydrogenase, decarboxylating OS=	1.606	1.398	1.351	1.408	1.144	1.268	1.639	1.191
Q13561	Dynactin subunit 2 OS=Homo sapiens GN=DCTN2 PE=1 SV=4 - [	1.542	1.014	1.394	0.957	1.239	1.015	1.280	1.214
P51572	B-cell receptor-associated protein 31 OS=Homo sapiens GN=BCA	1.352	1.304	1.194	1.192	1.072	1.213	1.266	1.173
B5BTY4	ATP-dependent RNA helicase DDX3X OS=Homo sapiens GN=DDX	1.320	1.170	1.078	0.770	1.322	1.266	1.248	0.999
Q6NTA2	HNRNPL protein (Fragment) OS=Homo sapiens GN=HNRNPL PE=	1.325	1.349	1.106	0.952	1.375	1.254	1.283	1.245
P05198	Eukaryotic translation initiation factor 2 subunit 1 OS=Homo sapi	1.463	1.199	1.367	0.970	1.211	1.115	1.234	1.401
A0A024R3E0	Interleukin 18 (Interferon-gamma-inducing factor), isoform CRA_	1.036	0.713	1.125	0.988	0.939	0.917	0.886	0.786
O60506	Heterogeneous nuclear ribonucleoprotein Q OS=Homo sapiens GI	1.448	1.590	1.105	0.847	1.286	1.215	1.211	1.090
Q9Y3A4	Ribosomal RNA-processing protein 7 homolog A OS=Homo sapier	1.478	1.650	1.288	1.219	1.322	1.333	1.459	1.453
P59998	Actin-related protein 2/3 complex subunit 4 OS=Homo sapiens GI	1.370	1.197	1.151	0.989	1.184	1.015	1.140	1.060
P54136	Arginine--tRNA ligase, cytoplasmic OS=Homo sapiens GN=RARS	1.341	1.255	1.293	0.981	1.133	1.061	1.205	1.132
P35270	Sepiapterin reductase OS=Homo sapiens GN=SPR PE=1 SV=1 - [	1.457	1.175	1.637	1.383	1.303	1.134	1.341	1.474
P10412	Histone H1.4 OS=Homo sapiens GN=HIST1H1E PE=1 SV=2 - [H1	1.092	2.528	1.208	1.237	0.865	0.925	0.847	1.456
Q9UKK9	ADP-sugar pyrophosphatase OS=Homo sapiens GN=NUDT5 PE=1	1.246	0.865	1.340	1.183	1.087	0.967	1.086	1.162
P0CW22	40S ribosomal protein S17-like OS=Homo sapiens GN=RPS17L PE	1.442	1.422	1.338	1.064	1.422	1.375	1.163	1.083
H7C4E5	Cytochrome c oxidase copper chaperone (Fragment) OS=Homo s	0.885	0.852	0.854	0.851	1.329	1.260	1.224	0.578
Q9NVP1	ATP-dependent RNA helicase DDX18 OS=Homo sapiens GN=DDX	1.645	1.748	1.414	1.149	1.541	1.411	1.568	1.523
P62917	60S ribosomal protein L8 OS=Homo sapiens GN=RPL8 PE=1 SV=	1.365	1.388	1.380	0.966	1.354	1.231	1.314	1.190
P62424	60S ribosomal protein L7a OS=Homo sapiens GN=RPL7A PE=1 S	1.340	1.389	1.191	1.003	1.201	1.340	1.184	1.060
P19105	Myosin regulatory light chain 12A OS=Homo sapiens GN=MYL12A	2.051	1.194	1.316	0.904	1.549	1.034	1.370	1.210
Q15005	Signal peptidase complex subunit 2 OS=Homo sapiens GN=SPCS	1.306	1.338	1.078	1.018	1.052	1.163	1.195	1.019
D6RCD0	Estradiol 17-beta-dehydrogenase 11 OS=Homo sapiens GN=HSD	1.489	1.298	1.114	0.924	1.133	1.103	1.186	0.993
Q2KS15	52 kDa protein GN=L1_52_55K PE=4 SV=1 - [Q2KS15_ADE05]	1.649	1.716	5.463	6.145	0.965	1.236	4.261	6.158
E7EU96	Casein kinase II subunit alpha OS=Homo sapiens GN=CSNK2A1 I	1.832	1.732	1.693	1.572	1.572	1.398	1.645	1.953
P01112	GTPase HRas OS=Homo sapiens GN=HRAS PE=1 SV=1 - [RASH_	1.243	1.627	1.423	1.123	1.093	1.188	1.529	1.553
Q9UNZ5	Leydig cell tumor 10 kDa protein homolog OS=Homo sapiens GN=	1.206	1.885	1.476	1.195	1.249	1.314	1.449	1.644
Q14914	Prostaglandin reductase 1 OS=Homo sapiens GN=PTGR1 PE=1 S	1.373	0.891	1.512	1.069	1.245	0.994	1.175	1.191
B3KMC9	cDNA FLJ10711 fis, clone NT2RP3000917, highly similar to 5'-3' e	1.411	1.476	1.056	0.818	1.367	1.323	1.264	1.054
Q9H098	Protein FAM107B OS=Homo sapiens GN=FAM107B PE=1 SV=1 -	2.032	1.223	1.562	1.225	1.501	0.908	1.243	1.207
Q9Y617	Phosphoserine aminotransferase OS=Homo sapiens GN=PSAT1 P	1.186	0.807	1.116	0.891	1.032	0.969	1.022	1.098
E9PN66	Tumor protein p53-inducible protein 11 (Fragment) OS=Homo sa	0.795	0.672	0.521	0.888	0.761	0.945	1.136	0.549

E5RJR5	S-phase kinase-associated protein 1 OS=Homo sapiens GN=SKP1	1.475	1.402	1.349	1.157	1.354	1.328	1.509	1.289
P62750	60S ribosomal protein L23a OS=Homo sapiens GN=RPL23A PE=1	1.490	1.581	1.428	1.029	1.399	1.342	1.335	1.193
Q12904	Aminoacyl tRNA synthase complex-interacting multifunctional pro	0.990	0.977	0.895	0.721	0.998	0.982	0.963	0.829
M0QWZ7	Serine--tRNA ligase, mitochondrial OS=Homo sapiens GN=SARS2	1.260	1.314	1.103	1.273	0.982	1.278	1.252	1.191
P08238	Heat shock protein HSP 90-beta OS=Homo sapiens GN=HSP90AE	1.244	0.859	1.217	1.008	1.129	1.060	1.128	1.043
A0A024R394	Cysteine and histidine-rich domain (CHORD)-containing 1, isoforn	1.820	1.273	1.958	1.549	1.394	1.109	1.400	1.399
A0A024R1Y2	ATP citrate lyase, isoform CRA_a OS=Homo sapiens GN=ACLY PE	0.429	0.327	0.404	0.302	0.466	0.898	0.508	0.418
Q9Y2S6	Translation machinery-associated protein 7 OS=Homo sapiens GN	0.602	0.621	1.336	0.910	0.779	0.934	0.689	0.599
F8W1G3	Bax inhibitor 1 (Fragment) OS=Homo sapiens GN=TMBIM6 PE=4	3.226	3.624	2.756	2.423	2.038	1.752	3.048	2.536
Q9Y3I0	tRNA-splicing ligase RtcB homolog OS=Homo sapiens GN=RTCB I	1.475	1.339	1.192	1.013	1.352	1.215	1.282	1.132
P53396	ATP-citrate synthase OS=Homo sapiens GN=ACLY PE=1 SV=3 - [	1.335	0.821	0.916	0.766	1.159	0.853	0.946	0.751
Q9Y2R9	28S ribosomal protein S7, mitochondrial OS=Homo sapiens GN=I	1.756	1.721	1.688	1.418	1.044	1.227	1.562	1.716
Q9BY32	Inosine triphosphate pyrophosphatase OS=Homo sapiens GN=IT	2.194	1.697	2.604	2.135	1.768	1.238	1.866	2.024
O00746	Nucleoside diphosphate kinase, mitochondrial OS=Homo sapiens	1.108	1.277	0.950	1.018	1.070	1.198	1.315	1.088
Q12981	Vesicle transport protein SEC20 OS=Homo sapiens GN=BNIP1 PE	1.416	1.920	1.512	1.475	1.385	1.512	1.836	1.380
C9J0K6	Sorcin OS=Homo sapiens GN=SRI PE=1 SV=1 - [C9J0K6_HUMAN	1.466	0.735	1.579	1.151	1.634	1.136	1.294	1.336
Q9UBX3	Mitochondrial dicarboxylate carrier OS=Homo sapiens GN=SLC25	1.467	1.580	1.184	1.127	0.970	1.011	1.320	1.440
Q13838	Spliceosome RNA helicase DDX39B OS=Homo sapiens GN=DDX3	1.571	1.865	1.479	1.170	1.617	1.632	1.527	1.598
P22570	NADPH:adrenodoxin oxidoreductase, mitochondrial OS=Homo sa	1.404	1.339	1.110	1.265	1.055	1.144	1.262	1.427
Q8WUW1	Protein BRICK1 OS=Homo sapiens GN=BRK1 PE=1 SV=1 - [BRK1	1.984	1.238	1.430	1.047	1.216	1.412	1.317	1.193
P00390	Glutathione reductase, mitochondrial OS=Homo sapiens GN=GSR	1.198	0.891	1.144	0.949	1.198	1.036	1.170	1.038
A0A024RC87	Ribonuclease/angiogenin inhibitor 1, isoform CRA_a OS=Homo sa	2.015	1.346	2.042	1.735	1.729	1.057	1.667	1.673
B2R4A2	Cytochrome b-c1 complex subunit 7 OS=Homo sapiens PE=2 SV=	0.830	0.910	0.715	0.800	0.673	0.964	0.826	0.887
P14927	Cytochrome b-c1 complex subunit 7 OS=Homo sapiens GN=UQC	1.298	1.365	1.102	1.202	1.057	1.030	1.257	1.319
P04843	Dolichyl-diphosphooligosaccharide--protein glycosyltransferase su	1.183	1.201	1.012	1.027	0.938	1.156	1.142	1.009
P07339	Cathepsin D OS=Homo sapiens GN=CTSD PE=1 SV=1 - [CATD_H	1.300	1.002	1.008	0.906	1.238	1.016	1.056	0.979
Q9BWJ5	Splicing factor 3B subunit 5 OS=Homo sapiens GN=SF3B5 PE=1 S	1.628	1.720	1.342	1.099	1.476	1.248	1.516	1.181
P10606	Cytochrome c oxidase subunit 5B, mitochondrial OS=Homo sapie	4.330	0.586	0.381	0.432	0.773	1.406	0.738	0.511
O95336	6-phosphogluconolactonase OS=Homo sapiens GN=PGLS PE=1 S	1.167	0.762	1.326	1.227	1.029	0.969	1.038	1.007
P62979	Ubiquitin-40S ribosomal protein S27a OS=Homo sapiens GN=RPS	3.583	3.966	4.438	2.945	2.803	1.218	2.199	2.552
A0A024R5H8	RAB6A, member RAS oncogene family, isoform CRA_b OS=Homo	1.264	1.439	1.198	1.235	1.145	1.234	1.277	1.288
G3V1D1	Ferritin OS=Homo sapiens GN=FTH1 PE=1 SV=1 - [G3V1D1_HUI	2.855	2.385	1.006	0.785	2.721	1.513	1.459	1.052
Mature	VII GN=mature_VII	2.049	1.734	4.337	6.052	1.223	1.155	2.739	6.172
P21964	Catechol O-methyltransferase OS=Homo sapiens GN=COMT PE=	1.816	2.051	1.790	1.798	1.265	1.342	1.611	1.475
Q9Y5M8	Signal recognition particle receptor subunit beta OS=Homo sapier	1.551	1.928	1.829	1.662	1.224	1.543	1.866	1.571
B2R960	cDNA, FLJ94230, highly similar to Homo sapiens thioredoxin-like	1.640	1.002	1.344	1.051	1.515	1.204	1.238	1.093
J3KTA4	Probable ATP-dependent RNA helicase DDX5 OS=Homo sapiens (	1.635	2.038	1.516	1.383	1.518	1.672	1.768	1.467
Q8N5I9	Uncharacterized protein C12orf45 OS=Homo sapiens GN=C12orf4	1.574	1.714	1.616	1.095	1.517	1.743	1.626	1.571
P60903	Protein S100-A10 OS=Homo sapiens GN=S100A10 PE=1 SV=2 -	1.054	1.131	0.962	1.119	1.088	1.371	1.169	1.019
B3KM48	cDNA FLJ10286 fis, clone HEMBB1001384, highly similar to COP9	1.222	0.975	1.082	0.826	1.205	1.127	1.037	1.012

P20700	Lamin-B1 OS=Homo sapiens GN=LMNB1 PE=1 SV=2 - [LMNB1_H	1.585	1.609	1.566	1.598	1.457	1.344	1.681	1.684
P62277	40S ribosomal protein S13 OS=Homo sapiens GN=RPS13 PE=1 S	1.702	1.517	1.560	1.026	1.554	1.336	1.468	1.420
P42166	Lamina-associated polypeptide 2, isoform alpha OS=Homo sapien	1.476	1.546	0.952	0.673	1.518	1.468	1.420	0.761
B3KX19	cDNA FLJ44500 fis, clone UTERU3000828, highly similar to 116 k	1.520	1.426	1.214	1.064	1.504	1.322	1.378	1.371
Q16658	Fascin OS=Homo sapiens GN=FSCN1 PE=1 SV=3 - [FSCN1_HUM	1.385	0.955	1.416	1.146	1.293	1.025	1.191	1.292
O00567	Nucleolar protein 56 OS=Homo sapiens GN=NOP56 PE=1 SV=4 -	1.574	1.992	1.297	1.216	1.390	1.366	1.427	1.331
P07741	Adenine phosphoribosyltransferase OS=Homo sapiens GN=APRT	1.186	0.924	1.414	1.300	0.932	0.965	1.165	1.091
O00483	Cytochrome c oxidase subunit NDUFA4 OS=Homo sapiens GN=N	1.212	1.252	1.148	1.286	1.043	1.045	1.391	1.171
P84095	Rho-related GTP-binding protein RhoG OS=Homo sapiens GN=Rh	1.599	1.628	1.333	1.077	1.342	1.297	1.539	1.459
P82930	28S ribosomal protein S34, mitochondrial OS=Homo sapiens GN=	1.449	1.442	1.208	1.033	1.023	1.285	1.333	1.220
P18077	60S ribosomal protein L35a OS=Homo sapiens GN=RPL35A PE=1	2.001	1.665	1.733	1.406	1.817	1.216	1.751	1.620
Q92945	Far upstream element-binding protein 2 OS=Homo sapiens GN=K	1.173	1.229	0.728	0.510	1.266	1.218	0.907	0.625
P17987	T-complex protein 1 subunit alpha OS=Homo sapiens GN=TCP1 F	1.267	0.978	1.197	0.896	1.217	0.948	1.172	1.075
B5BUB5	Autoantigen La (Fragment) OS=Homo sapiens GN=SSB PE=2 SV	1.159	1.096	1.212	0.937	1.293	1.086	1.082	1.014
A0A024R6W0	Aspartate aminotransferase OS=Homo sapiens GN=GOT2 PE=3 S	1.450	1.442	1.330	1.482	1.186	1.357	1.480	1.485
O43715	TP53-regulated inhibitor of apoptosis 1 OS=Homo sapiens GN=Tl	1.489	1.790	1.408	1.458	1.467	1.434	1.766	2.137
B2R8R5	cDNA, FLJ94025, highly similar to Homo sapiens tripartite motif-c	1.400	1.519	1.073	0.780	1.497	1.528	1.433	1.200
P49588	Alanine--tRNA ligase, cytoplasmic OS=Homo sapiens GN=AARS P	1.257	0.824	1.164	0.932	1.112	0.876	1.072	0.943
Q96DA6	Mitochondrial import inner membrane translocase subunit TIM14	1.273	1.517	1.233	1.250	0.962	1.104	1.397	1.408
Q96CT7	Coiled-coil domain-containing protein 124 OS=Homo sapiens GN=	1.389	1.192	1.656	1.103	1.203	1.091	1.265	1.314
Q9Y3F4	Serine-threonine kinase receptor-associated protein OS=Homo sa	1.960	1.289	1.865	1.349	1.705	1.179	1.755	1.695
P27348	14-3-3 protein theta OS=Homo sapiens GN=YWHAQ PE=1 SV=1	2.029	1.586	1.948	1.950	1.992	1.087	1.943	1.787
Q99584	Protein S100-A13 OS=Homo sapiens GN=S100A13 PE=1 SV=1 -	1.138	1.004	1.068	0.860	0.987	1.002	1.017	0.976
P19338	Nucleolin OS=Homo sapiens GN=NCL PE=1 SV=3 - [NUCL_HUMA	1.311	1.369	1.031	0.924	1.362	1.224	1.273	0.907
P52209	6-phosphogluconate dehydrogenase, decarboxylating OS=Homo	1.470	0.922	1.380	1.144	1.153	1.016	1.352	1.131
P51991	Heterogeneous nuclear ribonucleoprotein A3 OS=Homo sapiens (	1.435	1.405	1.597	1.267	1.540	1.384	1.622	1.317
Q99497	Protein DJ-1 OS=Homo sapiens GN=PARK7 PE=1 SV=2 - [PARK7	1.075	0.794	1.010	1.027	0.942	0.942	0.976	0.910
Q54A51	Basigin (Ok blood group), isoform CRA_a OS=Homo sapiens GN=	1.099	1.464	1.160	1.209	1.046	1.292	1.314	1.167
P25705	ATP synthase subunit alpha, mitochondrial OS=Homo sapiens GN	1.218	1.295	1.096	1.064	0.995	1.124	1.224	1.107
Q8TAE8	Growth arrest and DNA damage-inducible proteins-interacting pro	1.402	1.513	1.280	1.329	0.926	0.971	1.362	1.387
B2RB23	cDNA, FLJ95265, highly similar to Homo sapiens acetyl-Coenzyme	1.095	1.118	0.951	1.003	0.915	1.130	1.150	1.158
P36404	ADP-ribosylation factor-like protein 2 OS=Homo sapiens GN=ARL	1.262	1.081	1.242	1.032	1.136	0.990	1.210	1.106
P31946	14-3-3 protein beta/alpha OS=Homo sapiens GN=YWHAB PE=1 S	1.324	0.989	1.380	1.094	1.213	1.054	1.208	1.160
P09132	Signal recognition particle 19 kDa protein OS=Homo sapiens GN=	1.436	1.282	1.367	1.115	1.378	1.151	1.411	1.292
P30084	Enoyl-CoA hydratase, mitochondrial OS=Homo sapiens GN=ECHS	1.462	1.423	1.225	1.232	1.123	1.180	1.321	1.384
Q8WUY1	Protein THEM6 OS=Homo sapiens GN=THEM6 PE=1 SV=2 - [THI	1.752	1.820	1.626	1.498	1.530	1.394	1.928	1.574
B4DQT8	cDNA FLJ61158, highly similar to ADP-ribosylation factor-like prot	3.615	3.430	3.070	2.489	2.698	1.511	2.565	2.852
P43686	26S protease regulatory subunit 6B OS=Homo sapiens GN=PSMC	1.491	1.156	1.178	0.935	1.383	1.200	1.157	0.981
P28066	Proteasome subunit alpha type-5 OS=Homo sapiens GN=PSMA5	1.495	1.275	1.344	0.960	1.502	1.177	1.050	1.009
Q9Y333	U6 snRNA-associated Sm-like protein LSm2 OS=Homo sapiens GN	3.286	2.960	2.526	1.732	3.222	1.870	2.768	2.503

Q9BYN0	Sulfiredoxin-1 OS=Homo sapiens GN=SRXN1 PE=1 SV=2 - [SRXN	1.903	1.550	1.488	1.433	1.759	1.824	1.741	1.195
P23919	Thymidylate kinase OS=Homo sapiens GN=DTYMK PE=1 SV=4 -	1.255	1.024	1.188	1.067	0.989	1.024	1.088	1.109
Q9UKM9	RNA-binding protein Raly OS=Homo sapiens GN=RALY PE=1 SV=	1.384	1.424	0.908	0.681	1.516	1.285	1.302	1.195
P25398	40S ribosomal protein S12 OS=Homo sapiens GN=RPS12 PE=1 S	3.050	2.953	3.119	2.337	2.345	1.604	2.157	2.437
F5GZF0	Cyclin-dependent kinase 2-associated protein 1 (Fragment) OS=H	1.282	1.337	0.894	0.768	1.205	1.361	1.221	1.241
Q14764	Major vault protein OS=Homo sapiens GN=MVP PE=1 SV=4 - [MV	1.219	0.903	1.002	0.786	1.213	0.868	0.977	0.809
P30838	Aldehyde dehydrogenase, dimeric NADP-preferring OS=Homo sap	1.140	0.868	1.130	0.932	1.054	0.985	1.045	0.966
Q5CAQ5	Tumor rejection antigen (Gp96) 1 OS=Homo sapiens GN=TRA1 P	1.400	1.002	1.272	1.196	1.390	1.135	1.232	1.253
A6NGP5	Hematological and neurological-expressed 1-like protein OS=Hom	1.178	0.920	1.250	1.010	1.087	0.972	1.179	1.124
P16403	Histone H1.2 OS=Homo sapiens GN=HIST1H1C PE=1 SV=2 - [H1	0.947	1.767	0.892	0.944	0.674	0.842	0.653	1.083
Q9HAV7	GrpE protein homolog 1, mitochondrial OS=Homo sapiens GN=GI	1.697	1.737	1.434	1.665	1.449	1.376	1.937	1.709
P82909	28S ribosomal protein S36, mitochondrial OS=Homo sapiens GN=	1.120	1.103	0.947	0.943	1.042	1.256	1.240	1.076
P08559	Pyruvate dehydrogenase E1 component subunit alpha, somatic fo	1.339	1.347	1.121	1.102	1.187	1.102	1.198	1.164
P05141	ADP/ATP translocase 2 OS=Homo sapiens GN=SLC25A5 PE=1 SV	1.608	1.770	1.447	1.423	1.453	1.179	1.881	1.641
P20042	Eukaryotic translation initiation factor 2 subunit 2 OS=Homo sapi	1.848	1.756	1.555	1.083	1.670	1.132	1.612	1.614
B2R659	cDNA, FLJ92803, highly similar to Homo sapiens hydroxysteroid (	1.202	1.348	1.240	1.296	1.156	1.193	1.410	1.374
B1Q3B3	Ferritin (Fragment) OS=Homo sapiens GN=FTL PE=3 SV=1 - [B1	2.144	1.950	0.544	0.550	1.957	1.394	1.204	0.686
P28331	NADH-ubiquinone oxidoreductase 75 kDa subunit, mitochondrial (	1.259	1.251	1.031	1.131	0.960	1.100	1.199	1.151
Q16352	Alpha-internexin OS=Homo sapiens GN=INA PE=1 SV=2 - [AINX	1.411	1.783	1.162	1.031	1.222	1.288	1.286	1.093
Q96B45	UPF0693 protein C10orf32 OS=Homo sapiens GN=C10orf32 PE=	1.292	1.312	1.390	1.309	1.256	1.367	1.307	1.673
Q9NX24	H/ACA ribonucleoprotein complex subunit 2 OS=Homo sapiens GI	1.603	1.412	1.355	1.206	1.495	1.306	1.386	1.244
Q9UFG5	UPF0449 protein C19orf25 OS=Homo sapiens GN=C19orf25 PE=	1.310	1.393	1.252	1.643	1.282	1.558	1.711	1.677
P07900	Heat shock protein HSP 90-alpha OS=Homo sapiens GN=HSP90A	1.420	1.051	1.483	1.193	1.252	1.175	1.401	1.410
P62081	40S ribosomal protein S7 OS=Homo sapiens GN=RPS7 PE=1 SV=	1.862	1.548	1.565	1.211	1.531	1.255	1.581	1.360
F5GZS6	4F2 cell-surface antigen heavy chain OS=Homo sapiens GN=SLC3	1.050	1.520	1.205	1.147	1.014	1.379	1.170	1.172
Q15293	Reticulocalbin-1 OS=Homo sapiens GN=RCN1 PE=1 SV=1 - [RCN	3.284	1.876	3.002	2.834	2.955	1.598	2.853	3.112
B2R7C5	cDNA, FLJ93378, highly similar to Homo sapiens MCM3 minichron	1.205	1.269	1.111	0.794	1.368	1.321	1.152	0.894
P67936	Tropomyosin alpha-4 chain OS=Homo sapiens GN=TPM4 PE=1 S	1.505	0.931	1.020	0.798	1.233	1.143	1.092	0.938
Q92917	G patch domain and KOW motifs-containing protein OS=Homo sa	1.163	1.120	1.102	0.804	1.273	1.120	1.102	0.907
B4E380	Histone H3 OS=Homo sapiens PE=2 SV=1 - [B4E380_HUMAN]		0.237			0.421	0.907	0.358	0.183
O43684	Mitotic checkpoint protein BUB3 OS=Homo sapiens GN=BUB3 PE	1.168	1.380	1.057	0.853	1.331	1.237	1.213	1.013
P20339	Ras-related protein Rab-5A OS=Homo sapiens GN=RAB5A PE=1	1.027	1.311	0.999	1.086	1.048	1.320	1.033	1.091
K7EMU8	DNA (cytosine-5)-methyltransferase 1 OS=Homo sapiens GN=DN	1.189	1.839	1.378	0.911	1.022	1.095	1.608	1.302
A0A024RDF4	Heterogeneous nuclear ribonucleoprotein D (AU-rich element RNA	0.546	0.516	0.313	0.309	0.558	1.139	0.448	0.485
P31153	S-adenosylmethionine synthase isoform type-2 OS=Homo sapiens	1.575	1.308	1.624	1.178	1.541	1.460	1.400	1.129
B3KR50	cDNA FLJ33691 fis, clone BRAWH2002976, highly similar to GROV	1.685	1.109	1.486	1.153	1.433	1.141	1.409	1.324
A0A024R652	Methylenetetrahydrofolate dehydrogenase (NADP+ dependent) 1	1.360	0.951	1.458	1.157	1.287	1.043	1.250	1.246
P33176	Kinesin-1 heavy chain OS=Homo sapiens GN=KIF5B PE=1 SV=1	1.277	0.916	1.059	0.712	0.962	1.000	1.105	0.771
A0A087WXM6	60S ribosomal protein L17 (Fragment) OS=Homo sapiens GN=RP	1.704	1.580	1.564	1.085	1.487	1.382	1.425	1.223
E5RI56	Uncharacterized protein (Fragment) OS=Homo sapiens PE=4 SV=	1.886	1.337	1.569	1.282	1.648	1.353	1.585	1.523

P16104	Histone H2AX OS=Homo sapiens GN=H2AFX PE=1 SV=2 - [H2AX	1.022	1.237	0.709	0.686	1.028	1.244	1.104	1.056
Q14683	Structural maintenance of chromosomes protein 1A OS=Homo sa	1.413	1.605	1.290	1.088	1.273	1.225	1.359	1.217
P33992	DNA replication licensing factor MCM5 OS=Homo sapiens GN=MC	1.259	1.345	1.171	0.823	1.288	1.324	1.191	0.861
Q08257	Quinone oxidoreductase OS=Homo sapiens GN=CRYZ PE=1 SV=	1.392	1.192	0.992	0.980	1.010	1.054	1.112	1.071
P05091	Aldehyde dehydrogenase, mitochondrial OS=Homo sapiens GN=A	1.369	1.565	1.753	1.549	1.241	1.183	1.486	1.545
O75367	Core histone macro-H2A.1 OS=Homo sapiens GN=H2AFY PE=1 S	1.277	1.567	1.127	0.909	1.165	1.213	1.294	1.251
B2RDE8	cDNA, FLJ96580, highly similar to Homo sapiens hepatoma-derive	2.303	1.569	1.167	0.998	1.913	1.038	1.076	1.281
Q6PUV4	Complexin-2 OS=Homo sapiens GN=CPLX2 PE=1 SV=2 - [CPLX2	1.265	0.836	1.399	1.024	1.171	1.164	1.393	1.041
Q9Y295	Developmentally-regulated GTP-binding protein 1 OS=Homo sapi	1.287	1.156	1.318	0.841	1.032	1.044	1.342	1.088
B4DIG4	cDNA FLJ57115, highly similar to 39S ribosomal protein L19, mitc	1.239	1.470	1.219	1.200	1.152	1.289	1.420	1.265
P39023	60S ribosomal protein L3 OS=Homo sapiens GN=RPL3 PE=1 SV=	3.504	2.322	2.730	1.955	2.109	1.359	2.333	2.572
P09874	Poly [ADP-ribose] polymerase 1 OS=Homo sapiens GN=PARP1 PE	1.193	1.901	1.666	1.555	1.180	1.176	1.366	1.940
F5H2U8	High mobility group protein HMGI-C OS=Homo sapiens GN=HMG	1.557	1.735	1.012	0.965	0.981	0.961	0.959	0.999
Q08211	ATP-dependent RNA helicase A OS=Homo sapiens GN=DHX9 PE=	1.494	1.530	1.361	1.300	1.455	1.364	1.563	1.550
Q13347	Eukaryotic translation initiation factor 3 subunit I OS=Homo sapie	1.401	1.359	1.127	0.937	1.191	1.043	1.151	0.986
Q9GZY8	Mitochondrial fission factor OS=Homo sapiens GN=MFF PE=1 SV	1.099	1.077	0.935	0.886	0.895	1.267	0.862	0.934
P16989	Y-box-binding protein 3 OS=Homo sapiens GN=YBX3 PE=1 SV=4	3.663	3.338	3.548	2.739	2.438	1.603	2.915	2.918
Q9GZS3	WD repeat-containing protein 61 OS=Homo sapiens GN=WDR61	1.515	1.597	1.551	1.179	1.519	1.294	1.341	1.189
B4DMF5	Glutamate dehydrogenase OS=Homo sapiens PE=2 SV=1 - [B4D	1.385	1.501	1.184	1.284	1.164	1.199	1.300	1.391
P62753	40S ribosomal protein S6 OS=Homo sapiens GN=RPS6 PE=1 SV=	1.853	2.010	1.793	1.186	1.772	1.346	1.584	1.375
O00148	ATP-dependent RNA helicase DDX39A OS=Homo sapiens GN=DD	1.307	1.791	1.273	1.126	1.459	1.562	1.309	1.275
Q9P0M9	39S ribosomal protein L27, mitochondrial OS=Homo sapiens GN=	1.150	1.364	0.988	1.087	0.922	1.040	1.153	1.213
O43402	ER membrane protein complex subunit 8 OS=Homo sapiens GN=	1.300	1.381	1.382	1.230	1.458	1.364	1.364	1.343
Q9NX63	MICOS complex subunit MIC19 OS=Homo sapiens GN=CHCHD3 I	1.373	1.465	1.285	1.340	1.305	1.262	1.384	1.459
B4E1U1	cDNA FLJ52181, highly similar to Phosphatidylinositol transfer pro	2.311	1.634	2.525	2.202	2.071	1.113	2.410	2.315
B5BUB1	RuvB-like 1 (Fragment) OS=Homo sapiens GN=RUVBL1 PE=2 SV	1.460	1.211	1.313	1.084	1.336	1.233	1.291	1.245
P61088	Ubiquitin-conjugating enzyme E2 N OS=Homo sapiens GN=UBE2	1.625	1.192	1.688	1.387	1.520	1.071	1.490	1.337
P52597	Heterogeneous nuclear ribonucleoprotein F OS=Homo sapiens GN	1.142	1.328	1.038	0.808	1.276	1.368	1.065	0.968
P31947	14-3-3 protein sigma OS=Homo sapiens GN=SFN PE=1 SV=1 - [	1.697	1.240	1.939	1.508	1.384	1.028	1.620	1.632
D6RDG3	Transcription factor BTF3 (Fragment) OS=Homo sapiens GN=BTF		0.878			0.969	1.348	1.085	1.074
B2R514	cDNA, FLJ92300, Homo sapiens COP9 subunit 6 (MOV34 homolog	1.414	1.045	1.322	0.890	1.312	1.236	1.079	1.000
M0R3D6	60S ribosomal protein L18a (Fragment) OS=Homo sapiens GN=R	1.649	1.580	1.585	1.165	1.594	1.398	1.550	1.358
Q9Y266	Nuclear migration protein nudC OS=Homo sapiens GN=NUDC PE	1.521	1.056	1.557	1.290	1.351	1.117	1.481	1.317
P55769	NHP2-like protein 1 OS=Homo sapiens GN=NHP2L1 PE=1 SV=3 -	1.536	1.518	1.114	1.043	1.678	1.351	1.314	1.394
H0YN26	Acidic leucine-rich nuclear phosphoprotein 32 family member A O	1.113	1.009	1.007	0.888	1.195	1.040	0.970	0.868
E9PPM8	Putative deoxyribose-phosphate aldolase OS=Homo sapiens GN=	1.650	1.278	1.439	1.262	1.377	1.029	1.364	1.214
Q15121	Astrocytic phosphoprotein PEA-15 OS=Homo sapiens GN=PEA15	1.144	0.902	1.304	1.152	1.109	1.213	1.274	1.228
Q86W42	THO complex subunit 6 homolog OS=Homo sapiens GN=THOC6	1.640	1.593	1.626	1.234	1.464	1.400	1.814	1.797
B3KRM2	Serine/threonine-protein phosphatase OS=Homo sapiens PE=2 S	1.704	1.208	1.467	1.193	1.572	1.251	1.511	1.203
I3L397	Eukaryotic translation initiation factor 5A-1 (Fragment) OS=Homo	1.415	1.004	1.373	1.183	1.227	0.942	1.187	1.314

P07814	Bifunctional glutamate/proline--tRNA ligase OS=Homo sapiens GN	1.258	1.106	1.181	0.867	1.084	0.992	1.049	0.935
P23526	Adenosylhomocysteinase OS=Homo sapiens GN=AHCY PE=1 SV=	1.435	0.954	1.386	1.150	1.327	1.000	1.393	1.356
Q6WCQ1	Myosin phosphatase Rho-interacting protein OS=Homo sapiens G	3.029	2.874	1.961	1.591	2.410	1.326	2.496	1.701
P31350	Ribonucleoside-diphosphate reductase subunit M2 OS=Homo sap	2.614	4.738	7.276	5.352	2.091	3.810	8.685	8.364
G3V3U6	39S ribosomal protein L52, mitochondrial OS=Homo sapiens GN=	1.573	1.897	1.411	1.434		1.722	1.292	1.945
P12956	X-ray repair cross-complementing protein 6 OS=Homo sapiens GN	1.071	1.147	1.086	0.850	0.983	1.182	0.888	0.657
P63173	60S ribosomal protein L38 OS=Homo sapiens GN=RPL38 PE=1 S	1.518	1.468	1.492	1.061	1.312	1.232	1.330	1.124
O43920	NADH dehydrogenase [ubiquinone] iron-sulfur protein 5 OS=Hom	1.147	1.375	1.048	1.063	0.886	1.333	1.279	1.176
P38919	Eukaryotic initiation factor 4A-III OS=Homo sapiens GN=EIF4A3	1.482	1.485	1.317	1.060	1.538	1.288	1.480	1.364
P11216	Glycogen phosphorylase, brain form OS=Homo sapiens GN=PYGB	1.192	0.831	1.122	0.863	1.119	0.936	1.146	0.903
C9J712	Profilin-2 OS=Homo sapiens GN=PFN2 PE=1 SV=1 - [C9J712_HU	2.175	1.400	1.873	1.362	1.765	0.887	1.552	1.596
Q8N1F7	Nuclear pore complex protein Nup93 OS=Homo sapiens GN=NUP	1.776	1.600	1.561	1.697	1.629	1.386	1.737	1.877
O15523	ATP-dependent RNA helicase DDX3Y OS=Homo sapiens GN=DDX	1.273	1.238	1.208	0.849	1.147	1.269	1.279	1.108
Q9H9J2	39S ribosomal protein L44, mitochondrial OS=Homo sapiens GN=	1.615	1.556	1.383	1.145	1.191	1.371	1.602	1.435
J3KMY5	Epididymal secretory protein E1 OS=Homo sapiens GN=NPC2 PE=	1.261	1.295	1.340	1.420	1.517	1.176	1.549	1.593
Q96BM9	ADP-ribosylation factor-like protein 8A OS=Homo sapiens GN=AR	1.702	2.017	1.667	1.503	1.706	1.456	1.682	1.682
P38159	RNA-binding motif protein, X chromosome OS=Homo sapiens GN	1.433	1.492	1.241	1.001	1.363	1.307	1.406	1.329
Q92905	COP9 signalosome complex subunit 5 OS=Homo sapiens GN=CO	1.687	1.344	1.564	1.208	1.538	1.151	1.374	1.199
C9JNW5	60S ribosomal protein L24 OS=Homo sapiens GN=RPL24 PE=1 S	1.454	1.476	1.390	0.980	1.254	1.193	1.189	1.206
P63104	14-3-3 protein zeta/delta OS=Homo sapiens GN=YWHAZ PE=1 S	1.245	0.864	1.044	0.928	1.181	0.942	1.052	1.039
D3YTB1	60S ribosomal protein L32 (Fragment) OS=Homo sapiens GN=RP	1.678	1.486	1.437	1.122	1.519	1.252	1.492	1.264
Q9Y5Q8	General transcription factor 3C polypeptide 5 OS=Homo sapiens G	1.424	1.803	1.257	1.215	1.245	1.274	1.735	1.915
K7ERF1	Eukaryotic translation initiation factor 3 subunit K OS=Homo sapi	2.184	1.999	2.202	1.548	1.910	1.391	1.927	1.666
Q10471	Polypeptide N-acetylgalactosaminyltransferase 2 OS=Homo sapie	1.464	1.580	1.164	1.216	1.298	1.341	1.369	1.315
Q01780	Exosome component 10 OS=Homo sapiens GN=EXOSC10 PE=1 S	1.301	1.646	1.122	0.876	1.173	1.273	1.313	1.035
O00116	Alkyldihydroxyacetonephosphate synthase, peroxisomal OS=Hom	1.334	1.386	1.193	1.129	1.194	1.257	1.370	1.265
Q92520	Protein FAM3C OS=Homo sapiens GN=FAM3C PE=1 SV=1 - [FAM	1.418	1.460	1.268	1.242	1.136	1.277	1.478	1.346
P09211	Glutathione S-transferase P OS=Homo sapiens GN=GSTP1 PE=1	1.423	0.967	1.406	1.005	1.115	0.949	1.282	1.278
A8K4Z4	cDNA FLJ75549, highly similar to Homo sapiens ribosomal protein	1.819	1.297	1.625	1.218	1.592	1.355	1.579	1.454
Q9NQT5	Exosome complex component RRP40 OS=Homo sapiens GN=EXO	1.830	2.159	1.801	1.508	2.041	1.530	2.270	1.687
Q5JRI1	Serine/arginine-rich-splicing factor 10 OS=Homo sapiens GN=SRS	1.594	1.754	1.354	1.152	1.297	1.280	1.546	1.581
O00425	Insulin-like growth factor 2 mRNA-binding protein 3 OS=Homo sa	1.259	1.204	1.013	0.720	1.177	0.991	1.078	0.766
P12429	Annexin A3 OS=Homo sapiens GN=ANXA3 PE=1 SV=3 - [ANXA3_	1.102	0.638	1.049	0.970	1.044	0.869	0.973	1.031
O95757	Heat shock 70 kDa protein 4L OS=Homo sapiens GN=HSPA4L PE	1.367	1.056	1.385	1.024	1.366	1.197	1.456	1.319
O75083	WD repeat-containing protein 1 OS=Homo sapiens GN=WDR1 PE	1.549	1.093	1.378	1.223	1.302	1.102	1.413	1.151
Q01813	ATP-dependent 6-phosphofructokinase, platelet type OS=Homo s	1.341	0.898	1.263	0.973	1.073	0.974	1.108	1.045
Q5HYL6	Putative uncharacterized protein DKFZp686E1899 OS=Homo sapi	1.355	1.173	1.025	0.831	1.325	1.083	1.052	0.936
Q9Y3C1	Nucleolar protein 16 OS=Homo sapiens GN=NOP16 PE=1 SV=2 -	1.474	1.622	1.300	1.183	1.436	1.321	1.566	1.341
B3KTM6	Ribosomal protein L5, isoform CRA_b OS=Homo sapiens GN=RPL	3.607	3.182	3.515	2.549	2.943	1.310	2.586	2.898
P46063	ATP-dependent DNA helicase Q1 OS=Homo sapiens GN=RECQL	1.177	1.196	1.249	0.942	1.068	1.069	1.139	1.128

Q9P287	BRCA2 and CDKN1A-interacting protein OS=Homo sapiens GN=B	1.297	1.217	1.252	0.985	1.348	1.156	1.145	1.203
P61981	14-3-3 protein gamma OS=Homo sapiens GN=YWHAG PE=1 SV=	1.453	1.073	1.455	1.144	1.265	1.135	1.299	1.281
Q96HC4	PDZ and LIM domain protein 5 OS=Homo sapiens GN=PDLIM5 P	1.525	0.882	1.191	0.917	1.245	0.975	1.156	0.860
P62857	40S ribosomal protein S28 OS=Homo sapiens GN=RPS28 PE=1 S	1.860	1.662	1.913	1.696	1.978	1.295	1.670	1.734
Q9NZI8	Insulin-like growth factor 2 mRNA-binding protein 1 OS=Homo sa	1.459	1.418	1.203	0.767	1.221	1.104	1.203	0.926
C6EMX8	HsMcm7 OS=Homo sapiens PE=2 SV=1 - [C6EMX8_HUMAN]	1.224	1.192	1.089	0.781	1.355	1.295	1.139	0.962
Q6YN16	Hydroxysteroid dehydrogenase-like protein 2 OS=Homo sapiens (	1.456	1.308	1.059	1.321	1.299	1.163	1.312	1.487
P26885	Peptidyl-prolyl cis-trans isomerase FKBP2 OS=Homo sapiens GN=	1.806	1.234	1.599	1.398	1.372	1.167	1.321	1.432
B4DY08	Heterogeneous nuclear ribonucleoproteins C1/C2 OS=Homo sapie	1.503	1.651	1.181	1.017	1.639	1.431	1.679	1.443
C9JYQ9	60S ribosomal protein L22-like 1 OS=Homo sapiens GN=RPL22L1	1.472	1.302	1.240	0.874	1.484	1.335	1.333	1.261
A8K7Q1	cDNA FLJ77770, highly similar to Homo sapiens nucleobindin 1 (N	1.497	1.421	1.101	1.073	1.275	1.290	1.245	1.037
Q9Y2Z0	Suppressor of G2 allele of SKP1 homolog OS=Homo sapiens GN=	2.425	1.788	2.368	1.696	1.906	1.166	1.812	1.918
B2R7W0	cDNA, FLJ93628, Homo sapiens methylene tetrahydrofolate dehy	1.303	1.224	0.810	0.750	1.016	1.255	1.114	0.942
P31942	Heterogeneous nuclear ribonucleoprotein H3 OS=Homo sapiens (	1.212	1.243	0.927	0.749	1.395	1.231	1.181	0.892
P09669	Cytochrome c oxidase subunit 6C OS=Homo sapiens GN=COX6C	0.542	0.616	0.488	0.623	0.856	0.982	0.951	0.681
O15446	DNA-directed RNA polymerase I subunit RPA34 OS=Homo sapien	1.534	1.927	1.447	1.321	1.376	1.210	1.366	1.285
P53621	Coatomer subunit alpha OS=Homo sapiens GN=COPA PE=1 SV=	1.493	1.072	1.411	0.954	1.282	1.098	1.277	1.047
Q86U28	Iron-sulfur cluster assembly 2 homolog, mitochondrial OS=Homo	1.258	1.200	1.367	1.052	1.079	1.182	1.424	1.159
E9PE17	28S ribosomal protein S17, mitochondrial (Fragment) OS=Homo s	1.498	1.737	1.249	1.225	1.150	1.262	1.462	1.548
Q9NZN4	EH domain-containing protein 2 OS=Homo sapiens GN=EHD2 PE	1.297	1.047	0.953	0.589	1.153	0.887	0.819	0.690
Q9BWF3	RNA-binding protein 4 OS=Homo sapiens GN=RBM4 PE=1 SV=1	1.419	1.859	1.720	1.343	1.464	1.831	1.966	1.820
P62241	40S ribosomal protein S8 OS=Homo sapiens GN=RPS8 PE=1 SV=	1.511	1.396	1.394	0.918	1.322	1.240	1.358	1.187
P55735	Protein SEC13 homolog OS=Homo sapiens GN=SEC13 PE=1 SV=	2.267	1.725	2.227	1.812	1.860	1.271	2.048	2.053
B3KNN7	cDNA FLJ30049 fis, clone ADRGL1000033, highly similar to 26S p	1.382	1.016	1.130	0.885	1.361	1.110	1.034	0.925
B4DGM4	cDNA FLJ61114, highly similar to Homo sapiens intersex-lik (IXL),	1.754	1.677	1.372	1.150	1.531	1.316	1.460	1.145
A1L172	Acyl-CoA thioesterase 1 OS=Homo sapiens GN=ACOT1 PE=2 SV=	1.140	1.196	1.026	1.077	0.948	1.174	1.135	1.078
P16401	Histone H1.5 OS=Homo sapiens GN=HIST1H1B PE=1 SV=3 - [H	0.959	2.281	1.423	1.394	0.785	0.853	0.860	1.531
Q9Y490	Talin-1 OS=Homo sapiens GN=TLN1 PE=1 SV=3 - [TLN1_HUMAN	1.468	1.001	1.305	1.037	1.214	1.010	1.239	1.053
A8K6Q8	cDNA FLJ75881, highly similar to Homo sapiens transferrin recept		0.338			0.322	1.157		
B4DQI6	Transformer-2 protein homolog alpha OS=Homo sapiens GN=TRA	1.652	1.704	1.227	2.078	1.996	1.503	2.814	2.894
Q9Y3D2	Methionine-R-sulfoxide reductase B2, mitochondrial OS=Homo sa	1.250	1.256	1.161	0.884	0.925	1.126	1.201	0.956
E9PES6	High mobility group protein B3 (Fragment) OS=Homo sapiens GN	1.198	1.041	1.973	1.326	0.898	0.965	1.412	2.134
B3KN05	cDNA FLJ13129 fis, clone NT2RP3002969, highly similar to Long-c	1.196	1.112	0.908	0.887	0.935	1.071	1.016	0.816
Q9Y5L4	Mitochondrial import inner membrane translocase subunit Tim13	1.394	1.432	1.302	1.180	1.244	1.364	1.285	1.351
Q96HS1	Serine/threonine-protein phosphatase PGAM5, mitochondrial OS=	1.500	1.653	1.424	1.509	1.357	1.286	1.504	1.476
Q1KMD3	Heterogeneous nuclear ribonucleoprotein U-like protein 2 OS=Ho	1.699	1.696	1.325	1.233	1.672	1.206	1.663	1.528
Q1ED39	Lysine-rich nucleolar protein 1 OS=Homo sapiens GN=KNOP1 PE=	1.717	1.862	1.487	1.280	1.641	1.389	1.673	1.575
P13073	Cytochrome c oxidase subunit 4 isoform 1, mitochondrial OS=Hor	1.442	1.530	1.324	1.401	1.094	1.190	1.439	1.490
P25685	DnaJ homolog subfamily B member 1 OS=Homo sapiens GN=DN.	1.517	1.138	1.330	0.974	1.321	1.234	1.254	1.145
P35241	Radixin OS=Homo sapiens GN=RDX PE=1 SV=1 - [RADI_HUMAN	1.461	1.105	1.039	0.862	1.187	0.966	1.086	0.957

O15235	28S ribosomal protein S12, mitochondrial OS=Homo sapiens GN=	2.097	2.271	1.387	1.282	1.266	1.181	1.451	1.252
O43143	Putative pre-mRNA-splicing factor ATP-dependent RNA helicase D	1.544	1.425	1.151	1.098	1.404	1.289	1.383	1.121
Q59GB4	Dihydropyrimidinase-like 2 variant (Fragment) OS=Homo sapiens	1.177	0.761	1.043	0.792	1.108	0.818	0.917	0.806
P36873	Serine/threonine-protein phosphatase PP1-gamma catalytic subu	1.792	1.895	1.250	1.138	1.684	1.637	1.885	1.476
P00492	Hypoxanthine-guanine phosphoribosyltransferase OS=Homo sapi	1.668	1.200	1.610	1.591	1.469	1.058	1.416	1.570
Q6P587	Acylpyruvase FAHD1, mitochondrial OS=Homo sapiens GN=FAHD	1.425	1.252	1.151	1.207	1.140	1.149	1.351	1.198
A0A024R7U6	MCM4 minichromosome maintenance deficient 4 (S. cerevisiae), i	1.210	1.300	1.174	0.882	1.352	1.388	1.212	0.982
A0A087X2D5	39S ribosomal protein L45, mitochondrial OS=Homo sapiens GN=	1.590	1.169	1.096	1.029	1.083	1.326	1.186	1.067
F8W1A4	Adenylate kinase 2, mitochondrial OS=Homo sapiens GN=AK2 PE	1.299	1.387	1.250	1.367	1.226	1.265	1.299	1.383
P26641	Elongation factor 1-gamma OS=Homo sapiens GN=EEF1G PE=1	1.570	1.282	1.661	1.264	1.500	1.285	1.522	1.472
Q9BUP3	Oxidoreductase HTATIP2 OS=Homo sapiens GN=HTATIP2 PE=1 S	1.741	1.627	1.754	1.404	1.703	1.424	1.727	1.739
A0A087WUK2	Heterogeneous nuclear ribonucleoprotein D-like OS=Homo sapier	1.527	1.478	1.253	0.981	1.542	1.317	1.410	1.247
O43768	Alpha-endosulfine OS=Homo sapiens GN=ENSA PE=1 SV=1 - [EI	1.363	1.040	1.384	1.154	1.093	1.038	1.107	1.305
A6NLN1	Polypyrimidine tract binding protein 1, isoform CRA_b OS=Homo	1.410	1.623	1.117	0.778	1.372	1.389	1.271	0.968
Q04917	14-3-3 protein eta OS=Homo sapiens GN=YWHAH PE=1 SV=4 -	1.651	1.210	1.513	1.152	1.502	1.007	1.345	1.262
P07099	Epoxide hydrolase 1 OS=Homo sapiens GN=EPHX1 PE=1 SV=1 -	1.361	1.305	1.340	1.400	1.072	1.304	1.533	1.131
B4DP54	cDNA FLJ52712, highly similar to Tubulin beta-6 chain OS=Homo	1.505	0.954	1.562	1.091	1.291	1.056	1.154	1.137
B4DT77	Annexin OS=Homo sapiens PE=2 SV=1 - [B4DT77_HUMAN]	1.321	0.804	1.343	1.061	1.229	1.099	1.421	1.314
Q16762	Thiosulfate sulfurtransferase OS=Homo sapiens GN=TST PE=1 S	1.923	1.658	1.843	1.267	1.620	1.161	2.080	1.779
Q92522	Histone H1x OS=Homo sapiens GN=H1FX PE=1 SV=1 - [H1X_HU	1.282	1.442	0.989	0.760	1.088	0.864	0.916	0.997
P41208	Centrin-2 OS=Homo sapiens GN=CETN2 PE=1 SV=1 - [CETN2_H	1.418	1.664	1.600	1.546	1.266	1.347	1.719	1.708
X6RAL5	Histone deacetylase complex subunit SAP18 OS=Homo sapiens G	2.199	1.682	1.587	1.501	1.788	1.439	1.993	1.804
Q8WW12	PEST proteolytic signal-containing nuclear protein OS=Homo sapi	1.293	1.039	1.094	0.873	1.331	1.012	1.170	1.008
O43615	Mitochondrial import inner membrane translocase subunit TIM44	1.232	1.361	1.081	1.121	0.982	1.160	1.273	1.150
P50213	Isocitrate dehydrogenase [NAD] subunit alpha, mitochondrial OS=	1.451	1.480	1.254	1.256	1.106	1.227	1.359	1.394
P82673	28S ribosomal protein S35, mitochondrial OS=Homo sapiens GN=	1.157	1.349	0.961	1.004	0.984	1.030	1.163	1.059
P62140	Serine/threonine-protein phosphatase PP1-beta catalytic subunit	1.403	1.455	1.094	1.030	1.162	1.190	1.051	1.127
Q9BRQ6	MICOS complex subunit MIC25 OS=Homo sapiens GN=CHCHD6	1.182	1.317	1.337	1.322	1.258	1.103	1.255	1.426
Q4W4Y1	Dopamine receptor interacting protein 4 OS=Homo sapiens GN=	1.488	0.872	1.196	0.999	1.205	1.002	1.255	1.130
Q9NP97	Dynein light chain roadblock-type 1 OS=Homo sapiens GN=DYNL	1.197	0.804	1.122	0.859	1.104	1.016	1.111	0.952
P56537	Eukaryotic translation initiation factor 6 OS=Homo sapiens GN=E	1.552	1.365	1.329	1.118	1.547	1.414	1.426	1.196
P05114	Non-histone chromosomal protein HMG-14 OS=Homo sapiens GN	2.196	2.478	2.231	2.477	2.489	1.416	2.824	2.725
Q14344	Guanine nucleotide-binding protein subunit alpha-13 OS=Homo s	1.310	1.894	1.343	1.921	1.757	1.278	2.071	1.360
Q8N183	Mimitin, mitochondrial OS=Homo sapiens GN=NDUFAF2 PE=1 SV	1.591	1.603	1.612	1.461	1.255	1.194	1.486	1.366
B4DI38	Adenylyl cyclase-associated protein OS=Homo sapiens PE=2 SV=	1.443	0.861	1.176	0.949	1.236	0.954	1.081	0.959
A8K6X3	cDNA FLJ78679, highly similar to Homo sapiens DEAD (Asp-Glu-A	1.291	1.217	1.076	0.734	1.289	1.213	1.141	0.906
A0A024R1X8	Junction plakoglobin, isoform CRA_a OS=Homo sapiens GN=JUP	1.194	1.354	0.992	0.917	1.108	1.017	1.048	1.020
Q07955	Serine/arginine-rich splicing factor 1 OS=Homo sapiens GN=SRS	1.629	1.750	1.608	1.475	1.459	1.391	1.711	1.726
E5KSX8	Mitochondrial transcription factor A OS=Homo sapiens PE=4 SV=	1.232	1.401	1.141	1.099	1.009	1.190	1.276	1.178
P50402	Emerin OS=Homo sapiens GN=EMD PE=1 SV=1 - [EMD_HUMAN]	1.198	1.474	0.942	0.897	1.110	1.100	1.214	1.008

Q9NRV9	Heme-binding protein 1 OS=Homo sapiens GN=HEBP1 PE=1 SV=	1.256	1.127	1.388	1.325	1.177	1.112	1.225	1.302
Q5TD07	Ribosyldihydronicotinamide dehydrogenase [quinone] OS=Homo	1.411	1.132	1.471	1.643	0.991	1.023	1.357	1.234
Q92841	Probable ATP-dependent RNA helicase DDX17 OS=Homo sapiens	1.295	1.503	1.271	1.070	1.271	1.411	1.377	1.085
O60664	Perilipin-3 OS=Homo sapiens GN=PLIN3 PE=1 SV=3 - [PLIN3_HU	1.207	0.873	1.058	0.967	1.082	0.926	0.958	0.974
P30453	HLA class I histocompatibility antigen, A-34 alpha chain OS=Hom	2.157	3.246	2.847	2.753	2.029	1.706	2.901	2.738
Q8IVF2	Protein AHNAK2 OS=Homo sapiens GN=AHNAK2 PE=1 SV=2 - [A	1.353	1.610	0.987	0.988	1.199	1.174	1.074	0.995
C9JR33	NAD-dependent protein deacetylase sirtuin-2 (Fragment) OS=Hom	1.576	1.144	1.685	1.109	1.300	1.094	1.619	1.621
Q9UHV9	Prefoldin subunit 2 OS=Homo sapiens GN=PFDN2 PE=1 SV=1 - [	1.263	0.935	1.205	0.955	1.085	1.058	1.049	1.093
Q6P2Q9	Pre-mRNA-processing-splicing factor 8 OS=Homo sapiens GN=PR	1.481	1.586	1.250	1.006	1.429	1.336	1.403	1.195
B3KT70	cDNA FLJ37767 fis, clone BRHIP2024911, highly similar to Homo	1.466	1.602	1.214	1.031	1.211	1.278	1.272	1.200
Q9C002	Normal mucosa of esophagus-specific gene 1 protein OS=Homo s	1.907	1.564	1.247	1.122	1.274	0.958	1.301	1.359
Q9H773	dCTP pyrophosphatase 1 OS=Homo sapiens GN=DCTPP1 PE=1 S	2.334	1.808	2.555	2.252	2.193	1.182	2.146	2.071
P07602	Prosaposin OS=Homo sapiens GN=PSAP PE=1 SV=2 - [SAP_HUM	2.402	2.093	1.777	1.885	2.193	1.389	2.156	1.969
Q9Y5S9	RNA-binding protein 8A OS=Homo sapiens GN=RBM8A PE=1 SV=	1.490	1.357	1.248	1.212	1.533	1.267	1.498	1.404
P31150	Rab GDP dissociation inhibitor alpha OS=Homo sapiens GN=GDI1	1.827	1.348	2.162	1.647	1.632	1.107	1.738	1.708
P15880	40S ribosomal protein S2 OS=Homo sapiens GN=RPS2 PE=1 SV=	1.556	1.536	1.436	0.944	1.496	1.298	1.304	1.212
A8K4T2	cDNA FLJ76281, highly similar to Homo sapiens DnaJ (Hsp40) ho	1.772	1.308	1.707	1.376	1.506	1.256	1.627	1.502
Q9BUE6	Iron-sulfur cluster assembly 1 homolog, mitochondrial OS=Homo	1.584	1.871	1.251	1.092	1.159	1.294	1.596	1.147
P17568	NADH dehydrogenase [ubiquinone] 1 beta subcomplex subunit 7	1.207	1.481	1.079	1.119	0.959	1.071	1.239	1.249
P15559	NAD(P)H dehydrogenase [quinone] 1 OS=Homo sapiens GN=NQ	0.973	0.760	1.391	1.153	1.107	0.972	0.954	1.029
Q9NX14	NADH dehydrogenase [ubiquinone] 1 beta subcomplex subunit 1	1.601	1.870	1.345	1.297	1.202	1.240	1.586	1.518
Q9UFW8	CGG triplet repeat-binding protein 1 OS=Homo sapiens GN=CGG	1.209	1.530	1.636	1.352	1.066	1.188	1.719	2.078
O15372	Eukaryotic translation initiation factor 3 subunit H OS=Homo sapi	2.020	2.053	1.813	1.455	1.820	1.283	1.736	1.611
Q08ES8	Cell growth-inhibiting protein 34 OS=Homo sapiens PE=2 SV=1 -	1.645	1.471	1.483	1.293	1.433	1.280	1.393	1.245
Q9P0M6	Core histone macro-H2A.2 OS=Homo sapiens GN=H2AFY2 PE=1	1.186	1.385	1.156	1.022	0.872	1.246	1.399	1.248
Q13242	Serine/arginine-rich splicing factor 9 OS=Homo sapiens GN=SRSF	1.465	1.549	1.329	1.262	1.478	1.389	1.782	1.777
B2RBD5	cDNA, FLJ95457, highly similar to Homo sapiens tubulin, beta, 4 (	1.122	0.738	0.647	0.844	1.028	0.760	0.948	0.893
O95292	Vesicle-associated membrane protein-associated protein B/C OS=	1.328	1.348	1.082	1.033	1.062	1.318	1.281	1.078
P00491	Purine nucleoside phosphorylase OS=Homo sapiens GN=PNP PE=	1.789	1.257	1.763	1.601	1.607	1.139	1.711	1.527
Q9NXG2	THUMP domain-containing protein 1 OS=Homo sapiens GN=THU	1.292	1.148	1.141	0.777	1.173	1.201	0.922	0.873
A4QPB0	IQ motif containing GTPase activating protein 1 OS=Homo sapier	1.382	1.036	1.034	0.719	1.175	1.105	0.978	0.891
B9EGI2	Myosin phosphatase Rho interacting protein OS=Homo sapiens G					0.630			
O95167	NADH dehydrogenase [ubiquinone] 1 alpha subcomplex subunit 3	9.326	10.631	8.284	8.073	4.526	1.364	7.458	8.985
Q6I9Y2	THO complex subunit 7 homolog OS=Homo sapiens GN=THOC7	1.648	1.704	1.423	1.369	1.670	1.327	1.974	1.950
P61313	60S ribosomal protein L15 OS=Homo sapiens GN=RPL15 PE=1 S	1.121	0.954	1.039	0.793	1.209	1.224	1.120	0.887
P10301	Ras-related protein R-Ras OS=Homo sapiens GN=RRAS PE=1 SV	1.269	1.679	1.095	1.220	1.314	1.199	1.171	1.122
Q7KZA3	Ferrochelatase OS=Homo sapiens GN=DKFZp686P18130 PE=2 S\	1.604	1.650	1.210	1.314	1.144	1.284	1.405	1.319
Q5T123	SH3 domain-binding glutamic acid-rich-like protein 3 OS=Homo s	0.987	0.591	0.990	0.743	1.072	0.870	0.947	0.794
A8K3S0	cDNA FLJ76127, highly similar to Homo sapiens replication factor	1.235	1.349	1.294	1.141	1.322	1.297	1.473	1.289
P61513	60S ribosomal protein L37a OS=Homo sapiens GN=RPL37A PE=1	0.937	0.956	0.934	0.737	1.155	1.132	1.044	0.951

Q5EBL8	PDZ domain-containing protein 11 OS=Homo sapiens GN=PDZD1	1.311	1.220	1.376	1.192	1.190	1.174	1.337	1.454
Q7Z7K0	COX assembly mitochondrial protein homolog OS=Homo sapiens	4.032	3.142	2.947	2.607	2.148	1.231	1.919	2.486
Q08J23	tRNA (cytosine(34)-C(5))-methyltransferase OS=Homo sapiens G	1.247	1.162	1.155	0.839	1.275	1.127	1.057	0.834
P48444	Coatomer subunit delta OS=Homo sapiens GN=ARCN1 PE=1 SV=	1.323	0.866	1.351	0.935	1.341	1.089	1.215	1.046
Q9Y230	RuvB-like 2 OS=Homo sapiens GN=RUVBL2 PE=1 SV=3 - [RUVB	1.357	1.256	1.232	1.013	1.300	1.191	1.236	1.190
P37268	Squalene synthase OS=Homo sapiens GN=FDFT1 PE=1 SV=1 - [	1.552	1.435	1.211	1.070	1.131	1.253	1.231	1.086
P05023	Sodium/potassium-transporting ATPase subunit alpha-1 OS=Hom	1.262	1.797	1.425	1.465	1.209	1.295	1.399	1.493
Q4ZG57	Putative uncharacterized protein MCM6 (Fragment) OS=Homo sa	1.303	1.355	1.280	0.968	1.475	1.411	1.214	1.061
Q14254	Flotillin-2 OS=Homo sapiens GN=FLOT2 PE=1 SV=2 - [FLOT2_HU	1.189	1.492	1.093	1.182	1.066	1.105	1.121	1.075
B3KW56	Eukaryotic translation initiation factor 3 subunit E OS=Homo sapi	1.676	1.635	1.416	1.075	1.592	1.291	1.469	1.173
P78406	mRNA export factor OS=Homo sapiens GN=RAE1 PE=1 SV=1 - [	1.592	1.520	1.393	1.363	1.598	1.351	1.405	1.157
A0A024R2Z6	Guanine nucleotide binding protein-like 3 (Nucleolar), isoform CR	1.531	1.655	1.557	1.135	1.651	1.521	1.626	1.228
P49419	Alpha-aminoadipic semialdehyde dehydrogenase OS=Homo sapie	1.229	1.233	1.218	1.182	1.061	1.127	1.307	1.245
Q53GQ0	Estradiol 17-beta-dehydrogenase 12 OS=Homo sapiens GN=HSD	1.451	1.412	1.223	1.155	1.108	1.267	1.330	1.228
A0A024R1T5	2',3'-cyclic nucleotide 3' phosphodiesterase, isoform CRA_a OS=H	1.278	1.650	1.225	1.080	1.142	1.236	1.404	1.318
Q13573	SNW domain-containing protein 1 OS=Homo sapiens GN=SNW1	1.396	1.390	1.176	0.850	1.231	1.313	1.219	0.947
O15212	Prefoldin subunit 6 OS=Homo sapiens GN=PFDN6 PE=1 SV=1 - [	1.427	0.994	1.392	1.100	1.356	1.032	1.274	1.192
Q14696	LDLR chaperone MESD OS=Homo sapiens GN=MESDC2 PE=1 SV	1.588	1.212	1.530	1.552	1.486	1.402	1.642	1.570
Q6IPH7	RPL14 protein OS=Homo sapiens GN=RPL14 PE=2 SV=1 - [Q6IP	1.407	1.727	1.458	1.148	1.199	1.273	1.289	1.271
Q13501	Sequestosome-1 OS=Homo sapiens GN=SQSTM1 PE=1 SV=1 - [	2.708	2.206	1.361	0.891	2.831	2.222	1.489	0.925
P16615	Sarcoplasmic/endoplasmic reticulum calcium ATPase 2 OS=Homo	1.468	1.330	1.213	1.117	1.266	1.220	1.362	1.138
P49915	GMP synthase [glutamine-hydrolyzing] OS=Homo sapiens GN=GI	1.655	1.391	1.452	1.216	1.494	1.183	1.450	1.437
P46781	40S ribosomal protein S9 OS=Homo sapiens GN=RPS9 PE=1 SV=	1.856	1.661	1.475	0.966	1.597	1.299	1.693	1.582
Q02218	2-oxoglutarate dehydrogenase, mitochondrial OS=Homo sapiens	1.454	1.521	1.362	1.443	1.320	1.277	1.610	1.528
B2RAH7	cDNA, FLJ94921, highly similar to Homo sapiens prolyl endopepti	1.477	0.859	1.422	1.200	1.209	1.072	1.222	1.234
Q7Z4G1	COMM domain-containing protein 6 OS=Homo sapiens GN=COMI	1.272	0.932	1.250	1.051	1.407	1.307	1.212	1.303
P07195	L-lactate dehydrogenase B chain OS=Homo sapiens GN=LDHB PE	1.249	0.968	1.315	1.158	1.191	1.060	1.261	1.207
Q8WVC2	40S ribosomal protein S21 OS=Homo sapiens GN=RPS21 PE=1 S	3.766	2.989	3.339	2.469	3.735	1.512	3.315	3.368
B0AZQ4	cDNA, FLJ79494, highly similar to Structural maintenance of chro	1.349	1.366	1.201	1.140	1.295	1.301	1.305	1.401
P26639	Threonine--tRNA ligase, cytoplasmic OS=Homo sapiens GN=TARS	1.462	0.963	1.385	1.050	1.278	0.991	1.269	1.147
A8K0B6	cDNA FLJ77858, highly similar to Homo sapiens N-acylsphingosin	1.302	1.153	1.367	1.397	1.336	1.012	1.300	1.334
Q9UEH5	24-kDa subunit of complex I (Fragment) OS=Homo sapiens GN=	2.759	2.090	2.231	1.978	1.528	1.264	1.774	1.843
P35221	Catenin alpha-1 OS=Homo sapiens GN=CTNNA1 PE=1 SV=1 - [C	1.370	1.510	1.255	1.074	1.189	1.231	1.235	1.150
Q9NZU5	LIM and cysteine-rich domains protein 1 OS=Homo sapiens GN=I	1.593	1.095	1.369	1.075	1.357	1.107	1.209	1.090
Q6GMV3	Putative peptidyl-tRNA hydrolase PTRHD1 OS=Homo sapiens GN=	2.091	1.676	2.281	2.109	1.619	1.131	2.012	2.065
P43487	Ran-specific GTPase-activating protein OS=Homo sapiens GN=RA	1.330	0.937	1.404	1.118	1.159	0.964	1.168	1.286
F2Z2Y6	U6 snRNA-associated Sm-like protein LSm8 OS=Homo sapiens GI	1.658	1.318	1.142	0.930	1.447	1.211	1.177	0.946
Q9NRX1	RNA-binding protein PNO1 OS=Homo sapiens GN=PNO1 PE=1 S\	1.464	1.705	1.275	1.177	1.260	1.195	1.311	1.218
P62318	Small nuclear ribonucleoprotein Sm D3 OS=Homo sapiens GN=SI	1.450	1.333	1.007	0.885	1.431	1.234	1.212	1.064
P35237	Serpin B6 OS=Homo sapiens GN=SERPINB6 PE=1 SV=3 - [SPB6_	1.275	0.808	1.176	1.170	0.965	0.908	0.995	0.965

J9JID7	Lamin B2, isoform CRA_a OS=Homo sapiens GN=LMNB2 PE=1 S	1.392	1.525	1.333	1.466	1.381	1.275	1.522	1.571
Q9NTX5	Ethylmalonyl-CoA decarboxylase OS=Homo sapiens GN=ECHDC1	1.209	1.072	1.190	1.065	1.035	1.086	1.295	1.064
A8K670	cDNA FLJ75703, highly similar to Homo sapiens nitric oxide synth	1.447	1.455	1.385	1.056	1.342	1.339	1.392	0.782
A8MXP9	Matrin-3 OS=Homo sapiens GN=MATR3 PE=1 SV=1 - [A8MXP9_	1.290	1.364	1.055	0.821	1.443	1.404	1.271	1.056
B5BU32	Thymidine kinase OS=Homo sapiens GN=TK1 PE=2 SV=1 - [B5B	1.601	1.431	2.313	2.149	1.546	1.491	2.445	2.254
Q13868	Exosome complex component RRP4 OS=Homo sapiens GN=EXO	1.661	1.805	1.554	1.391	1.682	1.329	1.768	1.683
P53618	Coatomer subunit beta OS=Homo sapiens GN=COPB1 PE=1 SV=	1.626	1.127	1.517	1.103	1.506	1.215	1.374	1.311
P48739	Phosphatidylinositol transfer protein beta isoform OS=Homo sapi	1.544	1.707	1.590	1.334	1.511	1.256	1.652	1.600
Q8WUD1	Ras-related protein Rab-2B OS=Homo sapiens GN=RAB2B PE=1	1.021	1.093	0.980	0.846	0.779	1.149	0.941	0.941
P13797	Plastin-3 OS=Homo sapiens GN=PLS3 PE=1 SV=4 - [PLST_HUM	1.838	1.207	1.552	1.325	1.388	1.109	1.416	1.307
P19388	DNA-directed RNA polymerases I, II, and III subunit RPABC1 OS=	1.270	1.361	1.161	1.013	1.148	1.264	1.306	1.123
P61457	Pterin-4-alpha-carbinolamine dehydratase OS=Homo sapiens GN=	5.376	4.006	6.022	5.100	4.715	1.147	5.268	5.315
Q05639	Elongation factor 1-alpha 2 OS=Homo sapiens GN=EEF1A2 PE=1	1.761	1.101	1.080	0.842	1.452	1.116	1.350	1.178
B2RDW0	cDNA, FLJ96792, highly similar to Homo sapiens calmodulin 2 (p	3.200	2.633	2.983	2.890	3.143	1.191	2.726	3.046
B7Z592	cDNA FLJ61635, highly similar to Homo sapiens likely ortholog of	1.343	1.256	1.432	0.954	1.110	1.112	1.295	1.115
Q99798	Aconitate hydratase, mitochondrial OS=Homo sapiens GN=ACO2	1.497	1.414	1.287	1.390	1.115	1.163	1.514	1.491
J3KS15	Peptidyl-tRNA hydrolase ICT1, mitochondrial (Fragment) OS=Hon	1.825	1.919	1.367	1.490	1.332	1.402	1.812	1.611
P37837	Transaldolase OS=Homo sapiens GN=TALDO1 PE=1 SV=2 - [TAL	1.135	0.970	1.143	0.890	1.142	0.992	0.985	0.877
Q7Z6I8	UPF0461 protein C5orf24 OS=Homo sapiens GN=C5orf24 PE=1 S	1.231	1.581	1.222	0.919	1.035	1.077	1.307	0.904
B4DSH1	cDNA FLJ51295, highly similar to Cell division cycle 5-like protein	1.434	1.475	1.193	1.128	1.194	1.351	1.413	1.411
Q86UY0	Protein BLOC1S5-TXNDC5 OS=Homo sapiens GN=TXNDC5 PE=2	1.640	1.047	1.399	1.395	1.521	1.204	1.428	1.557
P53701	Cytochrome c-type heme lyase OS=Homo sapiens GN=HCCS PE=	1.757	1.925	1.791	1.711	1.179	1.263	1.834	1.776
Q9UMX5	Neudesin OS=Homo sapiens GN=NENF PE=1 SV=1 - [NENF_HU	1.140	0.728	1.090	1.139	1.082	1.072	1.305	1.095
Q9UNE7	E3 ubiquitin-protein ligase CHIP OS=Homo sapiens GN=STUB1 P	1.526	1.343	1.242	1.193	1.436	1.193	1.461	1.244
P61011	Signal recognition particle 54 kDa protein OS=Homo sapiens GN=	1.404	1.180	1.304	0.830	1.235	1.116	1.150	1.020
P40925	Malate dehydrogenase, cytoplasmic OS=Homo sapiens GN=MDH	1.122	0.750	1.193	1.023	0.987	0.996	1.137	0.964
P25787	Proteasome subunit alpha type-2 OS=Homo sapiens GN=PSMA2	0.955	0.707	0.834	0.630	0.960	1.043	0.880	0.674
P12270	Nucleoprotein TPR OS=Homo sapiens GN=TPR PE=1 SV=3 - [TP	1.385	1.470	1.308	1.332	1.419	1.326	1.460	1.563
Q96EE9	MTHFS protein (Fragment) OS=Homo sapiens GN=MTHFS PE=2	2.226	1.226	1.240	2.389	1.211	1.385	1.552	2.144
P42167	Lamina-associated polypeptide 2, isoforms beta/gamma OS=Hom	0.958	1.319	0.821	0.778	0.974	1.073	0.971	1.045
K7ESB0	Centromere protein S (Fragment) OS=Homo sapiens GN=APITD1	1.241	1.072	1.405	1.108	1.365	1.192	1.335	1.715
O60739	Eukaryotic translation initiation factor 1b OS=Homo sapiens GN=	5.797	4.198	4.351	2.763	4.524	1.537	3.972	3.645
A0A087WXS7	ATPase ASNA1 OS=Homo sapiens GN=ASNA1 PE=4 SV=1 - [A0A	1.577	1.314	1.460	1.355	1.312	1.151	1.299	1.294
Q15417	Calponin-3 OS=Homo sapiens GN=CNN3 PE=1 SV=1 - [CNN3_H	1.315	0.933	1.073	0.772	1.238	1.016	1.073	0.915
Q02878	60S ribosomal protein L6 OS=Homo sapiens GN=RPL6 PE=1 SV=	1.458	1.329	1.363	1.045	1.520	1.341	1.435	1.231
Q96CP2	FLYWCH family member 2 OS=Homo sapiens GN=FLYWCH2 PE=	0.958	0.756	0.992	0.738	1.009	1.215	1.130	0.587
Q9BY44	Eukaryotic translation initiation factor 2A OS=Homo sapiens GN=	1.302	1.232	1.181	0.775	1.067	1.093	1.110	0.945
P49327	Fatty acid synthase OS=Homo sapiens GN=FASN PE=1 SV=3 - [F	1.461	0.857	1.307	0.967	1.202	0.929	1.231	0.990
B4DEK2	cDNA FLJ59182, highly similar to Splicing factor, arginine/serine-r	2.324	2.308	2.281	1.762	1.612	1.466	1.633	2.216
P38606	V-type proton ATPase catalytic subunit A OS=Homo sapiens GN=	1.690	1.369	1.724	1.406	1.633	1.293	1.782	1.758

P18754	Regulator of chromosome condensation OS=Homo sapiens GN=R	1.366	1.431	1.231	1.133	1.236	1.297	1.375	1.032
Q9UHD8	Septin-9 OS=Homo sapiens GN=SEPT9 PE=1 SV=2 - [SEPT9_HU	1.131	0.934	0.929	0.674	1.040	1.004	0.974	0.795
F4ZW66	NF110b OS=Homo sapiens PE=2 SV=1 - [F4ZW66_HUMAN]	3.490	3.449	2.496	1.758	2.856	1.744	2.489	2.330
P32969	60S ribosomal protein L9 OS=Homo sapiens GN=RPL9 PE=1 SV=	1.972	1.694	1.906	1.362	1.671	1.365	1.647	1.326
Q8NCR1	VPS53 protein OS=Homo sapiens GN=VPS53 PE=2 SV=2 - [Q8N(	1.284	0.779	1.053	1.281	1.044	1.209	1.265	0.659
P07954	Fumarate hydratase, mitochondrial OS=Homo sapiens GN=FH PE	1.193	1.154	1.244	1.084	1.083	1.115	1.343	1.214
Q16698	2,4-dienoyl-CoA reductase, mitochondrial OS=Homo sapiens GN=	1.125	1.270	1.113	1.160	1.010	1.236	1.281	1.236
A0A024QZU5	Peroxisomal D3,D2-enoyl-CoA isomerase, isoform CRA_a OS=Hor	1.528	1.457	1.299	1.223	1.181	1.091	1.099	1.207
B3KM36	cDNA FLJ10153 fis, clone HEMBA1003417, highly similar to BAG f	1.357	1.308	1.390	1.479	1.479	1.180	1.461	1.659
Q96HE7	ERO1-like protein alpha OS=Homo sapiens GN=ERO1L PE=1 SV=	2.021	1.477	1.790	1.775	2.096	1.323	1.742	1.747
A8KAL2	cDNA FLJ76981, highly similar to Homo sapiens golgi autoantiger	1.218	1.418	1.169	1.213	1.032	1.257	1.244	1.216
Q8N6T3	ADP-ribosylation factor GTPase-activating protein 1 OS=Homo sa	1.408	1.016	1.218	0.856	1.438	1.262	1.135	1.112
J3KS05	Chromobox protein homolog 1 (Fragment) OS=Homo sapiens GN	1.875	1.773	1.778	1.799	1.692	1.688	1.885	1.601
P56378	6.8 kDa mitochondrial proteolipid OS=Homo sapiens GN=MP68 P	1.700	2.168	1.589	1.637	1.086	1.110	1.685	1.874
R4GN98	Protein S100 (Fragment) OS=Homo sapiens GN=S100A6 PE=1 S\	2.174	1.442	1.716	1.866	1.453	0.803	1.478	1.619
P30153	Serine/threonine-protein phosphatase 2A 65 kDa regulatory subu	1.449	1.162	1.380	1.041	1.393	1.109	1.448	1.230
Q14697	Neutral alpha-glucosidase AB OS=Homo sapiens GN=GANAB PE=	1.389	0.889	1.137	1.245	1.532	1.166	1.307	1.224
Q16527	Cysteine and glycine-rich protein 2 OS=Homo sapiens GN=CSRP2	2.431	2.247	2.559	1.798	1.934	1.201	2.217	2.258
Q7Z434	Mitochondrial antiviral-signaling protein OS=Homo sapiens GN=M	1.585	1.738	1.170	1.186	1.403	1.276	1.455	1.322
B2R983	cDNA, FLJ94267, highly similar to Homo sapiens glutathione S-tra	1.224	0.847	1.272	1.199	1.131	0.995	1.166	1.097
P31689	DnaJ homolog subfamily A member 1 OS=Homo sapiens GN=DN	2.356	2.992	3.826	2.997	2.053	2.083	2.333	2.228
B2R858	cDNA, FLJ93750, Homo sapiens DEAD (Asp-Glu-Ala-Asp) box poly	1.714	1.573	1.515	0.980	1.514	1.397	1.471	1.167
Q9UJV9	Probable ATP-dependent RNA helicase DDX41 OS=Homo sapiens	1.230	1.250	1.051	0.789	1.245	1.176	1.069	0.817
Q06210	Glutamine--fructose-6-phosphate aminotransferase [isomerizing]	1.327	0.876	1.129	0.896	1.284	1.016	1.061	0.896
P54886	Delta-1-pyrroline-5-carboxylate synthase OS=Homo sapiens GN=	1.488	1.678	1.387	1.373	1.278	1.286	1.645	1.429
A4GYY8	Putative uncharacterized protein DKFZp686F17268 (Fragment) OS	1.180	0.886	1.051	0.847	1.159	1.097	0.936	0.875
Q9NRX4	14 kDa phosphohistidine phosphatase OS=Homo sapiens GN=PH	1.590	1.040	1.657	1.339	1.144	0.867	1.475	1.397
A8K3Q7	Annexin OS=Homo sapiens PE=2 SV=1 - [A8K3Q7_HUMAN]	1.261	0.996	1.392	1.226	1.090	0.982	1.454	1.499
P82933	28S ribosomal protein S9, mitochondrial OS=Homo sapiens GN=N	1.463	2.036	1.816	1.728	0.971	1.194	1.302	1.364
Q8WVM8	Sec1 family domain-containing protein 1 OS=Homo sapiens GN=S	1.384	1.370	1.261	1.185	1.348	1.240	1.351	1.331
K7EIN2	Protein syndesmos (Fragment) OS=Homo sapiens GN=NUDT16L1	1.320	1.220	1.009	0.825	1.224	1.372	1.127	0.940
P09417	Dihydropteridine reductase OS=Homo sapiens GN=QDPR PE=1 S	1.464	1.102	1.593	1.168	1.503	1.126	1.514	1.519
F5H6E2	Unconventional myosin-Ic OS=Homo sapiens GN=MYO1C PE=1 S	1.350	0.956	0.947	0.706	1.091	1.095	0.967	0.879
B2R5U1	cDNA, FLJ92620, highly similar to Homo sapiens staphylococcal n	1.420	1.419	1.302	1.026	1.359	1.242	1.316	1.164
P61020	Ras-related protein Rab-5B OS=Homo sapiens GN=RAB5B PE=1	1.053	1.098	1.115	0.984	1.017	1.198	1.150	0.961
O75396	Vesicle-trafficking protein SEC22b OS=Homo sapiens GN=SEC22E	1.304	1.401	1.152	1.178	1.040	1.230	1.289	1.248
Q9BSD7	Cancer-related nucleoside-triphosphatase OS=Homo sapiens GN=	1.157	0.975	1.078	1.003	1.137	1.133	1.007	1.024
Q9NV06	DDB1- and CUL4-associated factor 13 OS=Homo sapiens GN=DC	1.716	1.884	1.251	0.883	1.525	1.490	1.555	0.923
D6R9P3	Heterogeneous nuclear ribonucleoprotein A/B OS=Homo sapiens	1.653	1.812	1.369	0.994	1.480	1.356	1.431	1.267
Q9BVC5	Ashwin OS=Homo sapiens GN=C2orf49 PE=1 SV=1 - [ASHWN_H	1.217	1.542	0.908	0.710	1.610	1.377	1.158	0.795

Q14247	Src substrate cortactin OS=Homo sapiens GN=CTTN PE=1 SV=2	1.429	1.320	1.216	1.015	1.348	1.083	1.312	1.204
P55145	Mesencephalic astrocyte-derived neurotrophic factor OS=Homo sa	1.221	0.836	1.141	0.984	1.572	1.134	1.351	1.059
O75607	Nucleoplasmin-3 OS=Homo sapiens GN=NPM3 PE=1 SV=3 - [NP	1.547	1.522	1.247	1.023	1.561	1.303	1.443	1.323
P43304	Glycerol-3-phosphate dehydrogenase, mitochondrial OS=Homo sa	1.493	1.521	1.134	1.255	1.115	1.070	1.281	1.234
Q9BQ61	Uncharacterized protein C19orf43 OS=Homo sapiens GN=C19orf4	1.367	1.198	1.091	0.697	1.244	1.295	1.085	0.893
Q53FD7	Nucleoporin 54kDa variant (Fragment) OS=Homo sapiens PE=2 S	1.541	1.693	1.665	1.532	1.647	1.417	1.643	1.812
O75489	NADH dehydrogenase [ubiquinone] iron-sulfur protein 3, mitocho	1.500	1.562	1.334	1.389	1.190	1.178	1.505	1.526
P63313	Thymosin beta-10 OS=Homo sapiens GN=TMSB10 PE=1 SV=2 -	0.135	0.149	0.137	0.135	0.258	0.790	0.267	0.176
C9JQ41	Coiled-coil domain-containing protein 58 OS=Homo sapiens GN=(	1.574	1.771	1.757	1.667	1.132	1.313	1.709	1.762
B4E0N6	cDNA FLJ56280, highly similar to Endoplasmic reticulum-Golgi int	1.248	1.142	1.020	0.988	1.068	1.095	1.056	0.943
I3L1P8	Mitochondrial 2-oxoglutarate/malate carrier protein (Fragment) O	1.183	1.255	1.025	1.019	0.936	1.067	1.154	1.123
Q96BR5	Cytochrome c oxidase assembly factor 7 OS=Homo sapiens GN=(	1.990	2.343	1.724	1.816	1.714	1.581	2.052	1.931
P55010	Eukaryotic translation initiation factor 5 OS=Homo sapiens GN=E	2.084	1.331	2.061	1.355	1.403	1.203	1.530	1.492
O43390	Heterogeneous nuclear ribonucleoprotein R OS=Homo sapiens GI	1.304	1.721	1.193	1.086	1.235	1.494	1.766	1.122
Q13151	Heterogeneous nuclear ribonucleoprotein A0 OS=Homo sapiens (	1.427	1.489	1.054	0.835	1.470	1.417	1.297	1.091
Q9NUQ9	Protein FAM49B OS=Homo sapiens GN=FAM49B PE=1 SV=1 - [FA	1.656	1.357	1.510	1.327	1.501	1.048	1.440	1.254
Q9BU76	Multiple myeloma tumor-associated protein 2 OS=Homo sapiens (	1.527	1.921	1.443	1.284	1.597	1.454	1.440	1.326
Q9BZE1	39S ribosomal protein L37, mitochondrial OS=Homo sapiens GN=	1.466	1.525	1.256	1.161	1.069	1.204	1.300	1.251
B3KMV8	cDNA FLJ12766 fis, clone NT2RP2001520, highly similar to Calciu	1.309	1.340	1.125	1.291	0.960	1.137	1.501	1.330
Q99460	26S proteasome non-ATPase regulatory subunit 1 OS=Homo sapi	1.779	1.205	1.389	0.967	1.570	1.188	1.243	1.164
P29084	Transcription initiation factor IIE subunit beta OS=Homo sapiens	1.164	1.364	1.136	0.846	1.183	1.221	1.174	0.862
Q07812	Apoptosis regulator BAX OS=Homo sapiens GN=BAX PE=1 SV=1	1.330	1.093	1.047	0.993	1.464	1.066	1.512	1.704
O75506	Heat shock factor-binding protein 1 OS=Homo sapiens GN=HSBP	1.311	1.177	1.346	1.169	1.349	1.117	1.513	1.341
H6VRF8	Keratin 1 OS=Homo sapiens GN=KRT1 PE=3 SV=1 - [H6VRF8_HU	1.700	1.051	1.965	1.707	0.648	0.834	3.510	0.819
Q9P0K7	Ankycorbin OS=Homo sapiens GN=RAI14 PE=1 SV=2 - [RAI14_H	1.451	1.570	1.230	1.129	1.352	1.276	1.302	1.306
Q59HE3	Calpastatin isoform a variant (Fragment) OS=Homo sapiens PE=2	0.983	0.833	0.958	0.589	0.962	1.039	0.990	0.825
F4ZW65	NF90b OS=Homo sapiens PE=2 SV=1 - [F4ZW65_HUMAN]	1.438	1.195	0.792	0.783	1.529	0.977	1.139	0.807
F5GYN4	Ubiquitin thioesterase OTUB1 OS=Homo sapiens GN=OTUB1 PE=	1.559	1.094	1.845	1.485	1.499	1.113	1.585	1.381
Q9BXW7	Cat eye syndrome critical region protein 5 OS=Homo sapiens GN=	1.301	1.346	1.303	1.289	0.941	1.231	1.204	1.184
Q8NFH5	Nucleoporin NUP53 OS=Homo sapiens GN=NUP35 PE=1 SV=1 -	1.413	1.703	1.413	1.532	1.594	1.367	1.723	1.732
P35606	Coatomer subunit beta' OS=Homo sapiens GN=COPB2 PE=1 SV=	1.510	1.058	1.455	1.153	1.525	1.044	1.336	1.152
P54578	Ubiquitin carboxyl-terminal hydrolase 14 OS=Homo sapiens GN=U	1.648	1.323	1.705	1.504	1.664	1.227	1.474	1.351
B3KTT6	cDNA FLJ38699 fis, clone KIDNE2002168, highly similar to Short	1.296	1.357	1.430	1.421	1.058	1.243	1.376	1.573
B4DRV2	cDNA FLJ53646, highly similar to Succinyl-CoA ligase (ADP-formir	1.597	1.622	1.347	1.362	1.173	1.155	1.455	1.548
J3QR09	Ribosomal protein L19 OS=Homo sapiens GN=RPL19 PE=1 SV=1	1.621	1.502	1.530	1.089	1.441	1.321	1.362	1.276
Q2KS04	33 kDa protein OS=Human adenovirus C serotype 5 GN=L4 PE=4	1.500	3.558	18.301	22.021	1.881	1.714	6.155	12.796
O75643	U5 small nuclear ribonucleoprotein 200 kDa helicase OS=Homo s	1.632	1.672	1.447	1.220	1.496	1.385	1.642	1.500
Q5U071	High-mobility group box 2 OS=Homo sapiens PE=2 SV=1 - [Q5U(	1.037	1.041	1.476	1.288	0.958	0.981	1.802	2.448
Q9BYG3	MKI67 FHA domain-interacting nucleolar phosphoprotein OS=Hor	2.504	2.736	2.183	1.934	2.002	1.769	2.237	1.696
P13693	Translationally-controlled tumor protein OS=Homo sapiens GN=T	1.902	1.481	2.613	2.140	1.386	1.291	1.741	1.611

Q9BWM7	Sideroflexin-3 OS=Homo sapiens GN=SFXN3 PE=1 SV=2 - [SFXN	1.128	1.477	1.084	0.964	1.069	0.986	1.058	0.986
P31930	Cytochrome b-c1 complex subunit 1, mitochondrial OS=Homo sa	1.221	1.361	1.095	1.113	1.045	1.123	1.181	1.327
Q99536	Synaptic vesicle membrane protein VAT-1 homolog OS=Homo sa	1.250	1.045	1.243	1.051	1.116	0.980	1.124	1.037
G3V1C3	Apoptosis inhibitor 5 OS=Homo sapiens GN=API5 PE=1 SV=1 - [	1.290	1.539	1.332	0.934	1.358	1.271	1.195	1.078
Q16851	UTP--glucose-1-phosphate uridylyltransferase OS=Homo sapiens	1.356	0.825	1.291	1.053	1.214	0.978	1.170	1.058
H0Y4R1	Inosine-5'-monophosphate dehydrogenase 2 (Fragment) OS=Hor	1.550	1.108	1.448	1.254	1.316	1.098	1.333	1.276
P49207	60S ribosomal protein L34 OS=Homo sapiens GN=RPL34 PE=1 S	1.755	1.405	1.650	1.068	1.737	1.171	1.730	1.325
A0A087WY71	AP-2 complex subunit mu OS=Homo sapiens GN=AP2M1 PE=4 S	1.148	1.271	1.070	0.719	1.210	0.940	1.110	1.137
Q06323	Proteasome activator complex subunit 1 OS=Homo sapiens GN=I	1.238	0.902	1.072	0.949	1.238	1.004	1.110	0.989
Q2TAY7	WD40 repeat-containing protein SMU1 OS=Homo sapiens GN=SN	1.580	1.681	1.674	1.607	1.577	1.403	1.660	1.668
P23246	Splicing factor, proline- and glutamine-rich OS=Homo sapiens GN	1.484	1.738	1.240	1.134	1.402	1.397	1.491	1.177
Q9NZL4	Hsp70-binding protein 1 OS=Homo sapiens GN=HSPBP1 PE=1 S\	1.702	1.118	1.848	1.392	1.307	1.173	1.450	1.702
Q9UBQ7	Glyoxylate reductase/hydroxypyruvate reductase OS=Homo sapie	1.069	0.832	1.178	1.026	0.891	1.037	1.032	0.962
B3GQE6	DEAD box polypeptide 27 OS=Homo sapiens GN=DDX27 PE=2 S\	1.453	1.535	1.322	1.096	1.437	1.328	1.386	1.161
Q9BV57	1,2-dihydroxy-3-keto-5-methylthiopentene dioxygenase OS=Hom	1.083	0.809	1.173	1.108	1.030	0.933	1.048	1.032
P40938	Replication factor C subunit 3 OS=Homo sapiens GN=RFC3 PE=1	2.082	2.221	2.367	2.235	1.936	1.429	1.901	2.040
P35268	60S ribosomal protein L22 OS=Homo sapiens GN=RPL22 PE=1 S	1.999	1.407	1.731	1.302	1.631	1.158	1.673	1.750
Q92930	Ras-related protein Rab-8B OS=Homo sapiens GN=RAB8B PE=1	0.740	0.950	0.663	0.617	0.839	1.016	0.844	0.800
Q9Y6G9	Cytoplasmic dynein 1 light intermediate chain 1 OS=Homo sapier	1.531	1.200	1.262	1.012	1.409	1.187	1.413	1.179
B7Z475	cDNA FLJ55712, highly similar to F-box-like/WD repeat protein TE	1.972	1.789	1.464	1.204	1.793	1.284	1.687	1.799
P09960	Leukotriene A-4 hydrolase OS=Homo sapiens GN=LTA4H PE=1 S\	1.337	0.935	1.491	1.350	1.139	1.038	1.166	1.160
Q15008	26S proteasome non-ATPase regulatory subunit 6 OS=Homo sapi	1.954	1.535	1.524	1.380	1.910	1.255	1.602	1.370
Q9UBT2	SUMO-activating enzyme subunit 2 OS=Homo sapiens GN=UBA2	1.150	1.258	1.093	0.845	1.304	1.108	1.161	0.882
O00232	26S proteasome non-ATPase regulatory subunit 12 OS=Homo sa	1.385	1.048	1.146	0.984	1.348	1.141	1.111	0.954
O43660	Pleiotropic regulator 1 OS=Homo sapiens GN=PLRG1 PE=1 SV=1	1.477	1.634	1.415	1.023	1.251	1.404	1.481	1.288
Q96EE3	Nucleoporin SEH1 OS=Homo sapiens GN=SEH1L PE=1 SV=3 - [S	2.208	2.848	2.421	2.687	2.110	1.542	2.258	2.669
P31949	Protein S100-A11 OS=Homo sapiens GN=S100A11 PE=1 SV=2 -	1.284	1.192	1.354	1.176	1.115	1.180	1.241	1.171
Q49B96	Cytochrome c oxidase assembly protein COX19 OS=Homo sapien	1.860	1.642	1.291	1.391	1.500	1.121	1.569	1.714
O60869	Endothelial differentiation-related factor 1 OS=Homo sapiens GN=	1.517	1.151	1.357	0.777	1.493	1.042	1.411	1.170
Q6NT72	UNC84B protein (Fragment) OS=Homo sapiens GN=UNC84B PE=	1.083	1.247	0.739	0.660	1.081	1.053	1.063	0.765
P45973	Chromobox protein homolog 5 OS=Homo sapiens GN=CBX5 PE=	2.096	2.240	1.961	1.929	1.750	1.395	1.857	1.705
B4DE36	Glucose-6-phosphate isomerase OS=Homo sapiens PE=2 SV=1 -	1.167	0.873	1.296	1.188	1.079	0.977	1.075	1.099
Q9Y5J6	Mitochondrial import inner membrane translocase subunit Tim10	1.721	1.287	1.671	1.327	1.407	1.280	1.781	1.815
O00410	Importin-5 OS=Homo sapiens GN=IPO5 PE=1 SV=4 - [IPO5_HUI	1.646	1.223	1.806	1.388	1.445	1.173	1.508	1.431
P22695	Cytochrome b-c1 complex subunit 2, mitochondrial OS=Homo sa	1.122	1.287	1.079	1.053	0.913	1.082	1.217	1.112
H0UID5	Adaptor-related protein complex 2, beta 1 subunit, isoform CRA_	1.152	1.256	1.113	0.877	1.174	1.100	1.110	1.091
D3DR65	SPFH domain family, member 1, isoform CRA_a OS=Homo sapier	1.202	1.147	1.048	1.011	0.868	1.220	1.342	1.019
A8W995	U exon protein GN=U_exon - [A8W995_ADE05]	2.911	5.764	19.155	20.278	1.971	1.941	5.467	6.404
Q86U75	Dihydropyrimidinase-like 2 OS=Homo sapiens PE=2 SV=1 - [Q86	1.078	1.064	1.030	1.109	1.388	1.178	1.381	1.440
Q9UHG3	Prenylcysteine oxidase 1 OS=Homo sapiens GN=PCYOX1 PE=1 S	1.516	1.469	1.462	1.416	1.316	1.198	1.546	1.484

P47755	F-actin-capping protein subunit alpha-2 OS=Homo sapiens GN=C	1.599	1.494	1.344	1.005	1.378	1.057	1.168	1.327
Q2KS18	extralong Terminal protein GN=weird_long_ptp - [Q2KS18_ADE0!	2.137	4.511	8.894	12.048	1.185	1.905	11.693	19.158
Q16822	Phosphoenolpyruvate carboxykinase [GTP], mitochondrial OS=Hc	1.271	1.191	0.924	0.915	1.053	1.095	1.139	1.050
P41227	N-alpha-acetyltransferase 10 OS=Homo sapiens GN=NAA10 PE=:	2.048	1.633	1.881	1.362	1.859	1.335	1.905	1.649
Q96MG7	Melanoma-associated antigen G1 OS=Homo sapiens GN=NDNL2	1.545	1.512	1.240	1.208	1.386	1.271	1.321	1.450
Q86XP3	ATP-dependent RNA helicase DDX42 OS=Homo sapiens GN=DDX	1.262	1.310	0.935	0.592	1.232	1.186	1.021	0.663
Q13435	Splicing factor 3B subunit 2 OS=Homo sapiens GN=SF3B2 PE=1	1.387	1.408	1.102	0.858	1.464	1.329	1.346	1.058
P31943	Heterogeneous nuclear ribonucleoprotein H OS=Homo sapiens GI	0.899	1.112	0.821	0.611	0.982	1.311	0.940	0.875
P11388	DNA topoisomerase 2-alpha OS=Homo sapiens GN=TOP2A PE=1	1.371	1.706	1.200	1.103	1.065	1.172	1.343	1.317
B4DXN6	Eukaryotic translation initiation factor 3 subunit B OS=Homo sapi	1.895	1.797	1.789	1.302	1.721	1.272	1.540	1.419
Q86TY5	Galectin OS=Homo sapiens PE=2 SV=1 - [Q86TY5_HUMAN]	1.245	0.875	1.203	1.072	1.048	0.940	1.130	1.062
Q15185	Prostaglandin E synthase 3 OS=Homo sapiens GN=PTGES3 PE=1	2.009	1.518	1.907	1.550	1.728	1.088	1.634	1.687
Q9Y305	Acyl-coenzyme A thioesterase 9, mitochondrial OS=Homo sapiens	1.191	1.309	1.014	1.005	1.069	1.088	1.040	1.059
E9PGT6	COP9 signalosome complex subunit 8 OS=Homo sapiens GN=CO	1.022	1.068	1.006	0.841	1.060	1.157	1.005	1.098
B2R7U4	cDNA, FLJ93605, highly similar to Homo sapiens heme oxygenase	2.254	1.831	1.178	0.719	1.886	1.599	1.343	0.847
B2R9K8	cDNA, FLJ94440, highly similar to Homo sapiens chaperonin cont	1.519	1.122	1.411	1.001	1.335	1.095	1.377	1.215
P43034	Platelet-activating factor acetylhydrolase IB subunit alpha OS=Ho	1.691	0.985	1.269	1.000	1.370	1.095	1.279	1.089
Q9UHB9	Signal recognition particle subunit SRP68 OS=Homo sapiens GN=	1.661	1.411	1.577	1.123	1.450	1.137	1.466	1.270
E9PBG7	Calcium/calmodulin-dependent protein kinase type II subunit delt	1.623	1.452	1.098	0.878	1.627	1.136	1.477	1.486
B2R791	cDNA, FLJ93335, highly similar to Homo sapiens PRP3 pre-mRNA	1.417	1.433	0.974	0.729	1.216	1.169	1.099	0.858
Q9NQ88	Fructose-2,6-bisphosphatase TIGAR OS=Homo sapiens GN=TIGA	1.200	0.815	1.431	1.002	1.016	0.986	1.395	1.380
P62487	DNA-directed RNA polymerase II subunit RPB7 OS=Homo sapiens	1.280	1.612	1.469	1.319	1.407	1.516	1.479	1.270
P36957	Dihydrolipoyllysine-residue succinyltransferase component of 2-o:	1.105	1.174	0.989	1.120	0.952	1.104	1.196	1.091
A0A024R8L7	Acyl-coenzyme A oxidase OS=Homo sapiens GN=ACOX1 PE=3 S\	1.164	1.333	1.067	1.031	1.131	1.286	1.369	1.259
P61081	NEDD8-conjugating enzyme Ubc12 OS=Homo sapiens GN=UBE2I	1.477	1.008	1.424	1.048	1.460	1.043	1.441	1.343
P22234	Multifunctional protein ADE2 OS=Homo sapiens GN=PAICS PE=1	1.725	1.189	1.581	1.330	1.517	1.046	1.522	1.344
Q13442	28 kDa heat- and acid-stable phosphoprotein OS=Homo sapiens	1.560	1.044	1.342	0.920	1.222	1.014	1.282	1.175
O75475	PC4 and SFRS1-interacting protein OS=Homo sapiens GN=PSIP1	1.537	1.535	1.249	1.098	1.233	1.196	1.420	1.312
Q14204	Cytoplasmic dynein 1 heavy chain 1 OS=Homo sapiens GN=DYN	1.483	0.965	1.325	0.994	1.276	1.070	1.285	1.047
D3DSV0	HCG2043421, isoform CRA_b OS=Homo sapiens GN=hCG_20434	1.379	0.931	0.745	0.497	1.339	1.000	0.793	0.638
B2R6U8	cDNA, FLJ93125, highly similar to Homo sapiens cleavage and po	1.274	1.576	1.161	0.909	1.316	1.302	1.243	0.992
E7ETK0	40S ribosomal protein S24 OS=Homo sapiens GN=RPS24 PE=1 S	1.749	1.508	1.507	1.010	1.603	1.221	1.484	1.393
Q7KZX8	G1 to S phase transition 1 OS=Homo sapiens PE=2 SV=1 - [Q7K	1.943	1.340	1.521	1.388	1.712	1.115	1.636	1.561
Q9H7B2	Ribosome production factor 2 homolog OS=Homo sapiens GN=RI	1.525	1.624	1.372	1.023	1.425	1.341	1.449	1.336
P22102	Trifunctional purine biosynthetic protein adenosine-3 OS=Homo s	1.296	0.845	1.179	1.034	1.103	0.928	1.136	1.050
Q9NTK5	Obg-like ATPase 1 OS=Homo sapiens GN=OLA1 PE=1 SV=2 - [O	1.502	0.994	1.481	1.305	1.250	1.044	1.466	1.343
Q502X2	Diablo homolog (Drosophila) OS=Homo sapiens GN=DIABLO PE=	1.653	1.736	1.435	1.643	1.607	1.295	1.678	1.747
Q9BQ04	RNA-binding protein 4B OS=Homo sapiens GN=RBM4B PE=1 SV=	1.786	2.285	2.196	1.793	1.647	2.007	2.334	1.955
P53999	Activated RNA polymerase II transcriptional coactivator p15 OS=I	1.764	1.776	1.658	1.735	1.273	0.876	1.125	1.425
Q9BXS5	AP-1 complex subunit mu-1 OS=Homo sapiens GN=AP1M1 PE=1	1.183	1.286	1.030	0.811	1.053	1.006	1.048	0.847

B2R4C1	cDNA, FLJ92036, highly similar to Homo sapiens ribosomal protei	1.978	2.005	1.900	1.298	1.749	1.394	1.773	1.485
F8VZJ2	Nascent polypeptide-associated complex subunit alpha, muscle-sp	1.489	1.184	1.505	0.954	1.302	1.035	1.286	1.119
B4E2V5	cDNA FLJ52062, highly similar to Erythrocyte band 7 integral mer	1.263	1.202	1.058	0.954	1.224	1.056	1.090	0.957
Q6IBS0	Twinfilin-2 OS=Homo sapiens GN=TWF2 PE=1 SV=2 - [TWF2_HU	1.121	0.755	1.107	0.879	1.073	0.916	1.051	0.896
P26373	60S ribosomal protein L13 OS=Homo sapiens GN=RPL13 PE=1 S	1.506	1.496	1.445	1.171	1.403	1.321	1.292	1.153
P29218	Inositol monophosphatase 1 OS=Homo sapiens GN=IMPA1 PE=1	1.174	0.898	1.186	1.009	1.058	0.991	1.162	1.012
Q6IB91	PCK2 protein OS=Homo sapiens GN=PCK2 PE=2 SV=1 - [Q6IB91	1.045	0.967	0.705	0.782	0.853	1.028	0.879	0.843
Q56VL3	OCIA domain-containing protein 2 OS=Homo sapiens GN=OCIAD	2.466	2.866	1.996	2.146	1.817	1.292	2.043	1.977
Q00839	Heterogeneous nuclear ribonucleoprotein U OS=Homo sapiens GI	2.909	2.103	2.050	1.639	2.003	1.312	2.017	1.818
P62328	Thymosin beta-4 OS=Homo sapiens GN=TMSB4X PE=1 SV=2 - [	0.035	0.038	0.022	0.038	0.272	0.908	0.119	0.051
Q01081	Splicing factor U2AF 35 kDa subunit OS=Homo sapiens GN=U2AF	1.266	1.434	1.077	1.042	1.184	1.161	1.133	0.897
P47897	Glutamine--tRNA ligase OS=Homo sapiens GN=QARS PE=1 SV=1	1.284	1.333	1.342	1.068	1.121	1.150	1.208	1.187
B4DLR3	cDNA FLJ54020, highly similar to Heterogeneous nuclear ribonucl	0.712	0.177	0.705	0.248	0.300	1.106	0.192	0.229
Q9UBE0	SUMO-activating enzyme subunit 1 OS=Homo sapiens GN=SAE1	1.209	1.176	1.330	0.948	1.453	1.031	1.189	0.974
P62826	GTP-binding nuclear protein Ran OS=Homo sapiens GN=RAN PE=	1.326	0.852	1.184	1.075	1.145	1.068	1.070	1.012
Q05193	Dynamin-1 OS=Homo sapiens GN=DNM1 PE=1 SV=2 - [DYN1_H	1.525	0.980	1.426	0.963	1.419	1.074	1.280	1.082
Q53GI2	Testis expressed sequence 264 variant (Fragment) OS=Homo sap	1.713	2.012	1.591	1.403	1.425	1.442	1.705	1.573
Q9UJU6	Drebrin-like protein OS=Homo sapiens GN=DBNL PE=1 SV=1 - [I	1.199	0.714	1.217	0.917	1.290	0.885	1.016	1.154
P78527	DNA-dependent protein kinase catalytic subunit OS=Homo sapier	1.303	1.584	1.139	0.929	1.058	1.140	1.132	0.976
Q9BVJ6	U3 small nucleolar RNA-associated protein 14 homolog A OS=Hor	1.425	1.476	1.002	0.778	1.315	1.257	1.249	0.967
Q02880	DNA topoisomerase 2-beta OS=Homo sapiens GN=TOP2B PE=1 S	1.223	1.568	1.215	1.203	0.992	1.057	1.285	1.079
P49773	Histidine triad nucleotide-binding protein 1 OS=Homo sapiens GN	1.452	0.938	1.363	1.255	1.119	0.914	1.240	1.300
Q15084	Protein disulfide-isomerase A6 OS=Homo sapiens GN=PDIA6 PE=	1.893	1.455	1.726	1.633	1.865	1.244	1.697	1.748
Q86SX6	Glutaredoxin-related protein 5, mitochondrial OS=Homo sapiens (	1.344	1.567	0.821	1.370	1.308	1.307	1.588	1.350
O75223	Gamma-glutamylcyclotransferase OS=Homo sapiens GN=GGCT P	1.416	1.025	1.360	1.291	1.035	1.009	1.270	1.287
Q6RFH5	WD repeat-containing protein 74 OS=Homo sapiens GN=WDR74	2.321	2.381	1.735	1.065	1.960	1.979	2.184	1.920
E9PCW1	Golgi SNAP receptor complex member 1 OS=Homo sapiens GN=(	1.472	1.688	1.251	1.093	1.271	1.337	1.522	1.306
P51648	Fatty aldehyde dehydrogenase OS=Homo sapiens GN=ALDH3A2	1.344	1.362	1.188	1.090	1.081	1.141	1.245	1.104
Q86V81	THO complex subunit 4 OS=Homo sapiens GN=ALYREF PE=1 SV	1.254	1.429	1.182	1.007	1.185	1.347	1.214	1.289
Q07973	1,25-dihydroxyvitamin D(3) 24-hydroxylase, mitochondrial OS=H	1.152	0.655	0.233	0.206	0.950	0.589	0.304	0.234
Q9UKN8	General transcription factor 3C polypeptide 4 OS=Homo sapiens (	1.898	2.107	1.839	1.476	1.721	1.467	1.908	2.144
P36543	V-type proton ATPase subunit E 1 OS=Homo sapiens GN=ATP6V1	1.570	1.361	1.478	1.433	1.633	1.440	1.759	1.655
P25325	3-mercaptopyruvate sulfurtransferase OS=Homo sapiens GN=MP	1.190	0.968	1.073	0.992	1.014	0.905	1.062	1.099
P49792	E3 SUMO-protein ligase RanBP2 OS=Homo sapiens GN=RANBP2	1.523	1.780	1.543	1.464	1.491	1.412	1.741	1.819
P55327	Tumor protein D52 OS=Homo sapiens GN=TPD52 PE=1 SV=2 - [	1.239	0.823	1.721	1.153	1.085	1.059	1.162	1.001
Q9H444	Charged multivesicular body protein 4b OS=Homo sapiens GN=C	1.487	0.877	1.227	0.910	1.042	1.036	1.159	1.028
P28070	Proteasome subunit beta type-4 OS=Homo sapiens GN=PSMB4 P	1.105	0.932	1.046	0.808	1.183	1.017	0.946	0.881
Q9NS69	Mitochondrial import receptor subunit TOM22 homolog OS=Homo	1.820	2.391	2.006	1.863	1.483	1.236	1.890	1.753
P36405	ADP-ribosylation factor-like protein 3 OS=Homo sapiens GN=ARL	1.019	0.795	1.222	1.078	0.938	0.936	0.994	0.996
Q9UES0	SNARE protein Ykt6 (Fragment) OS=Homo sapiens PE=2 SV=1 -	1.230	1.022	1.487	1.238	1.043	1.068	1.373	1.134

B2RDZ9	cDNA, FLJ96850 OS=Homo sapiens PE=2 SV=1 - [B2RDZ9_HUM/	1.905	2.015	1.639	1.474	1.743	1.447	1.857	1.985
P39748	Flap endonuclease 1 OS=Homo sapiens GN=FEN1 PE=1 SV=1 - [	1.109	1.042	1.196	0.798	0.978	1.117	1.006	0.856
C9JZR2	Catenin delta-1 OS=Homo sapiens GN=CTNND1 PE=1 SV=2 - [C	1.218	1.429	1.055	0.969	1.069	0.968	1.165	0.968
P46926	Glucosamine-6-phosphate isomerase 1 OS=Homo sapiens GN=GI	1.108	0.752	1.196	0.921	1.086	1.063	1.029	0.947
O00194	Ras-related protein Rab-27B OS=Homo sapiens GN=RAB27B PE=	1.307	1.283	1.041	0.990	1.155	1.100	1.080	0.984
Q9NR31	GTP-binding protein SAR1a OS=Homo sapiens GN=SAR1A PE=1	1.980	1.382	1.921	1.259	1.722	1.189	1.465	1.596
Q8WXF1	Paraspeckle component 1 OS=Homo sapiens GN=PSPC1 PE=1 SV	1.277	1.455	1.102	0.892	1.220	1.353	1.171	0.897
Q53GR7	Solute carrier family 25, member 13 (Citrin) variant (Fragment) C	1.277	1.391	1.130	1.099	0.912	1.095	1.159	1.185
P00338	L-lactate dehydrogenase A chain OS=Homo sapiens GN=LDHA PE	1.431	0.907	1.222	1.078	1.114	0.993	1.134	1.009
O95817	BAG family molecular chaperone regulator 3 OS=Homo sapiens G	1.457	0.993	1.115	0.908	1.353	1.280	1.404	1.236
P17096	High mobility group protein HMG-I/HMG-Y OS=Homo sapiens GN	2.012	2.157	1.495	1.468	1.571	1.208	1.449	1.531
Q86SZ5	Full-length cDNA clone CS0DL005YG10 of B cells (Ramos cell line	1.757	1.069	1.509	1.103	1.365	1.036	1.266	1.188
P37108	Signal recognition particle 14 kDa protein OS=Homo sapiens GN=	1.086	1.079	1.096	0.951	0.998	1.085	1.020	1.139
Q16718	NADH dehydrogenase [ubiquinone] 1 alpha subcomplex subunit 5	1.560	1.693	1.408	1.482	1.241	1.193	1.468	1.520
E9PLK3	Puromycin-sensitive aminopeptidase OS=Homo sapiens GN=NPEI	1.222	0.868	1.162	0.974	1.143	0.968	1.117	1.068
B2RDK6	cDNA, FLJ96656, highly similar to Homo sapiens SPFH domain fa	1.090	1.282	1.167	1.174	0.928	1.219	1.331	0.982
P78346	Ribonuclease P protein subunit p30 OS=Homo sapiens GN=RPP3	1.462	1.421	1.128	0.890	1.438	1.288	1.452	1.060
Q8NDP0	Putative uncharacterized protein DKFZp586G1518 (Fragment) OS	1.628	0.977	1.231	0.854	1.420	1.074	1.233	0.987
Q14137	Ribosome biogenesis protein BOP1 OS=Homo sapiens GN=BOP1	1.553	1.585	1.436	1.403	1.517	1.430	1.555	1.357
O94776	Metastasis-associated protein MTA2 OS=Homo sapiens GN=MTA2	1.096	1.121	0.765	0.542	1.035	1.285	1.068	0.806
Q9ULC4	Malignant T-cell-amplified sequence 1 OS=Homo sapiens GN=MC	1.499	1.138	1.249	1.106	1.289	1.116	1.260	1.029
B2R6J2	cDNA, FLJ92973, highly similar to Homo sapiens villin 2 (ezrin) (V	1.417	1.089	1.117	0.874	1.314	0.958	1.024	0.934
B4DL49	cDNA FLJ58073, moderately similar to Cathepsin B (EC 3.4.22.1)	2.968	1.941	1.706	1.581	2.123	1.191	2.046	1.789
Q9Y6M9	NADH dehydrogenase [ubiquinone] 1 beta subcomplex subunit 9	1.388	1.584	1.458	1.438	1.113	1.127	1.298	1.421
Q15785	Mitochondrial import receptor subunit TOM34 OS=Homo sapiens	1.302	0.953	1.131	0.996	1.175	1.147	1.329	1.266
L0R6Q1	Alternative protein SLC35A4 OS=Homo sapiens GN=SLC35A4 PE=	1.602	1.980	1.611	1.683	1.053	1.421	1.662	1.474
Q96FQ6	Protein S100-A16 OS=Homo sapiens GN=S100A16 PE=1 SV=1 -	1.214	0.963	1.184	0.780	1.107	1.009	1.074	0.905
P21912	Succinate dehydrogenase [ubiquinone] iron-sulfur subunit, mitocl	1.300	1.389	1.226	1.223	1.010	1.373	1.193	1.386
P27694	Replication protein A 70 kDa DNA-binding subunit OS=Homo sapi	1.281	1.666	2.042	1.913	1.307	1.522	1.611	1.630
Q9NWT1	p21-activated protein kinase-interacting protein 1 OS=Homo sapi	1.804	1.793	1.426	1.152	1.682	1.355	1.615	1.453
B4DZC0	cDNA FLJ51771, highly similar to SWI/SNF-related matrix-associa	1.366	1.654	1.376	1.175	1.241	1.402	1.524	1.349
B2R761	cDNA, FLJ93299, highly similar to Homo sapiens sterol carrier pro	1.460	1.412	1.402	1.553	1.349	1.265	1.504	1.564
Q12860	Contactin-1 OS=Homo sapiens GN=CNTN1 PE=1 SV=1 - [CNTN1	1.010	0.808	0.501	0.425	0.860	0.818	0.547	0.336
P49736	DNA replication licensing factor MCM2 OS=Homo sapiens GN=MC	1.248	1.305	1.234	0.917	1.367	1.324	1.169	1.027
Q9Y5J9	Mitochondrial import inner membrane translocase subunit Tim8 B	1.224	1.361	1.029	0.988	1.306	1.213	1.364	1.271
Q04760	Lactoylglutathione lyase OS=Homo sapiens GN=GLO1 PE=1 SV=	2.184	1.598	2.529	2.137	1.722	1.019	1.960	2.060
P05026	Sodium/potassium-transporting ATPase subunit beta-1 OS=Homo	1.346	1.258	1.055	0.999	1.146	1.172	1.053	1.087
A8KAH7	cDNA FLJ75444, highly similar to Homo sapiens protein kinase, c/	1.205	1.167	1.100	0.986	1.167	1.116	1.244	1.142
P13010	X-ray repair cross-complementing protein 5 OS=Homo sapiens GI	1.335	1.458	1.349	1.135	1.267	1.165	1.159	0.975
P07384	Calpain-1 catalytic subunit OS=Homo sapiens GN=CAPN1 PE=1 S	1.515	1.036	1.380	1.121	1.470	1.074	1.365	1.085

A8K622	cDNA FLJ75871, highly similar to Homo sapiens staufen, RNA bin	1.438	1.200	1.028	0.819	1.304	1.290	1.274	0.922
Q9UHX1	Poly(U)-binding-splicing factor PUF60 OS=Homo sapiens GN=PUF	1.372	1.530	1.349	0.856	1.353	1.309	1.292	1.081
A0A024QZR0	Leucine rich repeat containing 20, isoform CRA_a OS=Homo sapi	1.159	0.884	1.324	1.052	1.263	0.972	1.214	1.202
B4DJK9	Perilipin OS=Homo sapiens PE=2 SV=1 - [B4DJK9_HUMAN]	1.240	1.557	1.350	1.086	1.240	1.476	1.532	1.425
F8WAJ0	Probable ATP-dependent RNA helicase DDX31 OS=Homo sapiens	1.609	1.984	1.397	1.014	1.602	1.554	1.749	1.321
P61289	Proteasome activator complex subunit 3 OS=Homo sapiens GN=F	1.357	1.331	1.289	0.970	1.513	1.260	1.211	1.207
P41091	Eukaryotic translation initiation factor 2 subunit 3 OS=Homo sapi	1.980	1.578	1.742	1.070	1.582	1.191	1.552	1.603
Q96CS3	FAS-associated factor 2 OS=Homo sapiens GN=FAF2 PE=1 SV=2	1.536	1.303	1.137	1.020	1.138	1.345	1.266	1.098
Q9NPJ6	Mediator of RNA polymerase II transcription subunit 4 OS=Homo	1.439	1.569	1.236	0.969	1.458	1.279	1.466	1.179
Q8N5K1	CDGSH iron-sulfur domain-containing protein 2 OS=Homo sapien	1.646	1.793	1.592	2.628	1.324	1.656	1.739	1.877
J3QT77	Serum paraoxonase/arylesterase 2 OS=Homo sapiens GN=PON2	5.316	4.935	4.976	4.523	2.908	1.312	4.789	4.150
Q9NZ45	CDGSH iron-sulfur domain-containing protein 1 OS=Homo sapien	1.446	1.847	1.605	1.857	1.165	1.309	1.969	1.865
B5BU25	U2 small nuclear RNA auxiliary factor 2 isoform b OS=Homo sapie	1.964	2.281	1.757	1.494	1.692	1.401	2.049	1.414
X6RCK5	Dynactin subunit 3 (Fragment) OS=Homo sapiens GN=DCTN3 PE	1.458	1.006	1.283	1.058	1.334	1.121	1.275	1.179
B5BUD2	Replication factor C 2 isoform 1 (Fragment) OS=Homo sapiens GI	2.449	2.523	2.374	2.055	1.863	1.551	2.423	2.464
K4DI93	Cullin 4B, isoform CRA_e OS=Homo sapiens GN=CUL4B PE=1 SV	1.207	1.092	0.981	0.765	1.261	1.052	0.932	0.718
Q99961	Endophilin-A2 OS=Homo sapiens GN=SH3GL1 PE=1 SV=1 - [SH3	1.476	0.952	1.441	1.155	1.214	1.060	1.318	1.311
Q9BSQ6	RPL13A protein (Fragment) OS=Homo sapiens GN=RPL13A PE=2	1.723	1.628	1.555	1.345	1.743	1.323	1.689	1.372
Q9Y237	Peptidyl-prolyl cis-trans isomerase NIMA-interacting 4 OS=Homo	1.504	0.992	1.101	1.089	1.212	0.958	1.431	1.292
Q13185	Chromobox protein homolog 3 OS=Homo sapiens GN=CBX3 PE=	1.276	1.354	1.136	0.822	1.372	1.345	1.278	1.151
K7EKE6	Lon protease homolog, mitochondrial OS=Homo sapiens GN=LON	1.226	1.189	1.073	1.020	0.962	1.157	1.139	1.075
Q9BVJ8	HEXA protein (Fragment) OS=Homo sapiens GN=HEXA PE=2 SV=	1.533	1.324	1.573	1.719	1.528	1.222	1.609	1.690
P84090	Enhancer of rudimentary homolog OS=Homo sapiens GN=ERH PI	4.656	4.666	4.056	3.689	3.963	1.716	4.052	4.578
Q9HC52	Chromobox protein homolog 8 OS=Homo sapiens GN=CBX8 PE=	1.306	1.455	1.266	0.980	1.045	1.215	1.240	1.114
Q59GU6	Sorting nexin 1 isoform a variant (Fragment) OS=Homo sapiens F	1.328	0.920	1.144	0.945	1.222	1.065	1.161	1.138
Q9HCD5	Nuclear receptor coactivator 5 OS=Homo sapiens GN=NCOA5 PE	1.373	1.759	1.499	1.299	1.356	1.738	1.791	1.651
B2RCD8	cDNA, FLJ96012, highly similar to Homo sapiens interferon regula	1.321	1.327	1.022	0.839	1.374	1.331	1.217	0.905
B7Z9I1	Medium-chain-specific acyl-CoA dehydrogenase, mitochondrial OS	1.314	1.406	1.214	1.167	1.059	1.215	1.235	1.308
A0A024R4E5	High density lipoprotein binding protein (Vigilin), isoform CRA_a C	1.352	1.568	1.182	0.745	1.280	1.217	1.111	0.791
P67809	Nuclease-sensitive element-binding protein 1 OS=Homo sapiens C	1.584	1.502	1.406	1.120	1.499	1.274	1.304	1.213
P61254	60S ribosomal protein L26 OS=Homo sapiens GN=RPL26 PE=1 S	1.481	1.385	1.426	1.061	1.398	1.318	1.263	1.117
P30048	Thioredoxin-dependent peroxide reductase, mitochondrial OS=Hc	1.473	1.364	1.208	1.286	1.090	1.199	1.296	1.369
O75534	Cold shock domain-containing protein E1 OS=Homo sapiens GN=	1.839	1.622	1.699	1.238	1.616	1.214	1.517	1.398
E7ETU9	Procollagen-lysine,2-oxoglutarate 5-dioxygenase 2 OS=Homo sap	0.901	0.659	0.695	0.637	1.071	1.170	0.666	0.653
Q9H2H8	Peptidyl-prolyl cis-trans isomerase-like 3 OS=Homo sapiens GN=F	1.279	1.422	1.210	1.113	1.501	1.509	1.466	1.595
Q5QNZ2	ATP synthase F(0) complex subunit B1, mitochondrial OS=Homo	1.326	1.710	1.443	1.626	0.952	1.039	1.417	1.576
Q12788	Transducin beta-like protein 3 OS=Homo sapiens GN=TBL3 PE=1	1.477	1.502	1.550	1.483	1.611	1.272	1.680	1.485
Q9HBH5	Retinol dehydrogenase 14 OS=Homo sapiens GN=RDH14 PE=1 S	1.185	1.337	1.100	1.053	1.179	1.324	1.388	1.207
P61586	Transforming protein RhoA OS=Homo sapiens GN=RHOA PE=1 S	1.792	1.433	1.418	1.111	1.269	1.115	1.391	1.269
Q9UHR4	Brain-specific angiogenesis inhibitor 1-associated protein 2-like pr	1.555	1.413	1.274	1.027	1.302	1.151	1.356	1.239

Q9BVJ7	Dual specificity protein phosphatase 23 OS=Homo sapiens GN=D	1.190	1.055	1.417	1.219	1.079	1.045	1.221	1.114
Q6DD88	Atlastin-3 OS=Homo sapiens GN=ATL3 PE=1 SV=1 - [ATLA3_HU	1.530	1.724	1.393	1.374	1.330	1.291	1.652	1.352
E9PJB8	Selenoprotein S OS=Homo sapiens GN=VIMP PE=1 SV=1 - [E9PJ	1.915	2.817	2.459	1.490	1.680	2.599	2.995	1.530
P82914	28S ribosomal protein S15, mitochondrial OS=Homo sapiens GN=	1.368	1.358	1.035	0.956	0.909	1.042	1.194	1.174
P61006	Ras-related protein Rab-8A OS=Homo sapiens GN=RAB8A PE=1	0.807	0.935	0.735	0.411	0.921	1.075	1.078	0.493
Q12797	Aspartyl/asparaginyl beta-hydroxylase OS=Homo sapiens GN=AS	1.297	1.284	0.987	1.004	0.971	1.109	1.115	0.939
Q15046	Lysine--tRNA ligase OS=Homo sapiens GN=KARS PE=1 SV=3 - [S	1.267	1.155	1.132	1.018	1.234	1.050	1.178	1.158
Q14257	Reticulocalbin-2 OS=Homo sapiens GN=RCN2 PE=1 SV=1 - [RCN	2.213	1.608	2.235	1.866	1.555	1.268	1.547	1.990
P00374	Dihydrofolate reductase OS=Homo sapiens GN=DHFR PE=1 SV=	1.416	0.975	1.579	1.320	1.218	1.032	1.482	1.511
P04040	Catalase OS=Homo sapiens GN=CAT PE=1 SV=3 - [CATA_HUMAI	1.884	1.943	1.848	1.985	1.687	1.184	1.700	2.114
Q9UJZ1	Stomatin-like protein 2, mitochondrial OS=Homo sapiens GN=ST	1.068	1.167	1.020	1.076	1.120	1.159	1.308	1.092
Q9Y678	Coatomer subunit gamma-1 OS=Homo sapiens GN=COPG1 PE=1	1.604	1.168	1.467	1.104	1.461	1.075	1.324	1.112
Q8TCS8	Polyribonucleotide nucleotidyltransferase 1, mitochondrial OS=Ho	1.419	1.539	1.297	1.362	1.089	1.322	1.403	1.298
P31327	Carbamoyl-phosphate synthase [ammonia], mitochondrial OS=Ho	1.298	1.352	1.186	1.225	1.067	1.165	1.291	1.178
Q9Y262	Eukaryotic translation initiation factor 3 subunit L OS=Homo sapi	1.963	1.847	1.904	1.391	1.762	1.268	1.621	1.585
P46060	Ran GTPase-activating protein 1 OS=Homo sapiens GN=RANGAP	1.588	1.698	1.536	1.449	1.524	1.463	1.621	1.741
B7SBB1	Aryl hydrocarbon receptor interacting protein OS=Homo sapiens (	1.369	1.108	1.435	1.201	1.195	1.230	1.303	1.225
G3V529	ATP-dependent RNA helicase DDX24 OS=Homo sapiens GN=DDX	1.621	1.925	1.340	1.005	1.514	1.407	1.623	1.178
Q9BTV4	Transmembrane protein 43 OS=Homo sapiens GN=TMEM43 PE=	1.421	1.374	1.288	1.180	1.193	1.374	1.443	1.284
P09429	High mobility group protein B1 OS=Homo sapiens GN=HMGB1 PE	1.109	0.974	1.711	1.393	0.895	0.847	1.611	2.576
Q9GZU8	Protein FAM192A OS=Homo sapiens GN=FAM192A PE=1 SV=1 -	1.116	0.983	0.940	0.760	1.131	1.075	1.011	0.923
P09001	39S ribosomal protein L3, mitochondrial OS=Homo sapiens GN=M	1.421	1.546	1.124	1.071	0.980	1.099	1.253	1.132
H0YEF3	Ribonuclease H2 subunit C (Fragment) OS=Homo sapiens GN=RI	1.363	1.214	1.654	1.517	1.368	1.132	1.304	1.295
P33316	Deoxyuridine 5'-triphosphate nucleotidohydrolase, mitochondrial (	1.371	1.275	1.342	1.315	1.095	1.019	1.220	1.268
Q9UMS4	Pre-mRNA-processing factor 19 OS=Homo sapiens GN=PRPF19 P	1.305	1.421	1.269	1.112	1.424	1.334	1.434	1.573
Q9NW13	RNA-binding protein 28 OS=Homo sapiens GN=RBM28 PE=1 SV=	1.485	1.768	1.353	1.229	1.206	1.450	1.412	1.506
O96000	NADH dehydrogenase [ubiquinone] 1 beta subcomplex subunit 1(	1.105	1.237	0.923	0.958	0.875	1.105	1.152	0.997
Q8IVT2	Mitotic interactor and substrate of PLK1 OS=Homo sapiens GN=M	1.559	1.742	1.246	1.060	1.362	1.385	1.295	1.192
Q8N5L8	Ribonuclease P protein subunit p25-like protein OS=Homo sapien	2.836	2.360	1.607	1.930	1.926	1.640	2.625	1.621
Q9Y5B9	FACT complex subunit SPT16 OS=Homo sapiens GN=SUPT16H P	1.702	1.910	2.190	1.923	1.523	1.366	2.245	2.509
P62879	Guanine nucleotide-binding protein G(I)/G(S)/G(T) subunit beta-2	1.302	1.645	1.366	1.375	1.129	1.146	1.188	1.201
Q5JRC6	PHD finger protein 6 OS=Homo sapiens GN=PHF6 PE=1 SV=1 - [	1.446	1.455	1.135	1.135	1.195	1.237	1.209	1.049
Q9HDC9	Adipocyte plasma membrane-associated protein OS=Homo sapier	1.479	1.472	1.342	1.303	1.212	1.306	1.526	1.398
Q9ULW0	Targeting protein for Xklp2 OS=Homo sapiens GN=TPX2 PE=1 SV	1.253	1.754	1.328	1.086	1.034	1.457	1.886	1.777
Q8N4P8	GTPBP4 protein (Fragment) OS=Homo sapiens GN=GTPBP4 PE=.	1.773	1.997	1.300	1.071	1.593	1.446	1.640	1.458
Q9BTT5	Similar to NADH dehydrogenase (Ubiquinone) 1 alpha subcomple	1.236	1.463	1.411	1.160	1.025	1.206	1.438	1.205
P21281	V-type proton ATPase subunit B, brain isoform OS=Homo sapiens	1.278	1.128	1.252	1.191	1.397	1.290	1.400	1.329
Q6IPN0	Reticulon OS=Homo sapiens GN=RTN4 PE=2 SV=1 - [Q6IPN0_H	1.111	1.270	0.928	0.919	1.022	1.100	1.055	1.142
O43159	Ribosomal RNA-processing protein 8 OS=Homo sapiens GN=RRP8	1.426	1.704	1.340	1.153	1.452	1.529	1.610	1.517
Q9NSE4	Isoleucine--tRNA ligase, mitochondrial OS=Homo sapiens GN=IAI	1.260	1.302	1.118	1.047	1.103	1.175	1.310	1.160

Q6PIU2	Neutral cholesterol ester hydrolase 1 OS=Homo sapiens GN=NCE	1.332	1.156	1.034	0.952	1.093	1.122	1.157	0.949
E7EQG2	Eukaryotic initiation factor 4A-II OS=Homo sapiens GN=EIF4A2 P	1.155	0.741	0.964	0.896	1.016	0.923	0.968	0.846
B4DEZ3	NADH dehydrogenase [ubiquinone] 1 alpha subcomplex subunit 1	0.921	1.196	1.253	0.974	0.844	1.139	1.148	0.991
O94874	E3 UFM1-protein ligase 1 OS=Homo sapiens GN=UFL1 PE=1 SV=	1.642	1.823	1.648	1.340	1.639	1.330	1.577	1.539
Q9H488	GDP-fucose protein O-fucosyltransferase 1 OS=Homo sapiens GN	1.409	0.892	1.394	1.234	1.350	1.269	1.476	1.444
B4DDF4	Calponin-2 OS=Homo sapiens GN=CNN2 PE=1 SV=1 - [B4DDF4_	1.323	0.912	1.248	0.866	1.122	0.935	1.087	1.041
P22314	Ubiquitin-like modifier-activating enzyme 1 OS=Homo sapiens GN	1.179	0.920	1.183	0.971	1.063	0.944	1.032	1.010
Q15286	Ras-related protein Rab-35 OS=Homo sapiens GN=RAB35 PE=1 S	1.195	1.229	0.984	0.974	1.010	1.202	1.097	1.047
Q6FI81	Anamorsin OS=Homo sapiens GN=CIAPIN1 PE=1 SV=2 - [CPIN1	2.504	1.833	3.175	2.713	2.079	1.171	2.054	2.159
P42696	RNA-binding protein 34 OS=Homo sapiens GN=RBM34 PE=1 SV=	1.953	2.106	1.634	1.219	1.693	1.496	1.740	1.691
Q9Y4L1	Hypoxia up-regulated protein 1 OS=Homo sapiens GN=HYOU1 PI	1.451	1.013	1.237	1.219	1.406	1.199	1.284	1.196
P06737	Glycogen phosphorylase, liver form OS=Homo sapiens GN=PYGL	1.214	0.787	1.156	0.951	1.089	0.939	1.155	0.919
B4DV28	cDNA FLJ54170, highly similar to Cytosolic nonspecific dipeptidas	1.527	1.072	1.392	1.157	1.327	0.979	1.315	1.226
Q8N128	Protein FAM177A1 OS=Homo sapiens GN=FAM177A1 PE=1 SV=1	1.841	1.691	1.232	1.233	1.797	1.165	1.457	1.224
A4FU11	RPS4Y1 protein (Fragment) OS=Homo sapiens GN=RPS4Y1 PE=2	2.461	2.375	2.057	1.475	1.888	1.389	1.962	1.880
Q13283	Ras GTPase-activating protein-binding protein 1 OS=Homo sapier	1.140	1.347	1.046	0.747	1.040	1.233	1.119	0.841
P46778	60S ribosomal protein L21 OS=Homo sapiens GN=RPL21 PE=1 S	1.362	1.198	1.136	0.859	1.260	1.154	1.114	0.984
P35580	Myosin-10 OS=Homo sapiens GN=MYH10 PE=1 SV=3 - [MYH10_	1.512	0.969	1.116	0.833	1.278	1.104	1.233	1.025
Q9H0A0	N-acetyltransferase 10 OS=Homo sapiens GN=NAT10 PE=1 SV=2	1.630	1.880	1.571	1.423	1.441	1.480	1.754	1.610
B3KTJ9	cDNA FLJ38393 fis, clone FEBRA2007212 OS=Homo sapiens PE=	1.365	1.438	1.016	0.729	1.450	1.272	1.326	1.007
P42766	60S ribosomal protein L35 OS=Homo sapiens GN=RPL35 PE=1 S	1.301	1.597	1.316	1.024	1.323	1.208	1.283	1.357
Q9Y512	Sorting and assembly machinery component 50 homolog OS=Hor	1.335	1.606	1.361	1.386	1.255	1.220	1.459	1.435
B4DG62	cDNA FLJ56506, highly similar to Hexokinase-1 (EC 2.7.1.1) OS=I	1.440	1.407	1.439	1.377	1.223	1.269	1.510	1.680
B2R5M9	cDNA, FLJ92537, highly similar to Homo sapiens procollagen-lysir	1.423	1.062	1.163	1.169	1.435	1.166	1.391	1.485
P11387	DNA topoisomerase 1 OS=Homo sapiens GN=TOP1 PE=1 SV=2 -	1.196	1.727	1.242	1.171	1.008	1.151	1.212	1.339
P52701	DNA mismatch repair protein Msh6 OS=Homo sapiens GN=MSH6	1.163	1.038	1.063	0.791	1.209	1.181	0.893	0.738
B3KY11	cDNA FLJ46571 fis, clone THYMU3041428, highly similar to Proba	1.233	1.417	1.031	0.872	1.161	1.243	1.298	1.115
P24941	Cyclin-dependent kinase 2 OS=Homo sapiens GN=CDK2 PE=1 SV	2.421	3.964	3.635	2.943	2.617	2.085	3.565	2.747
O60675	Transcription factor MafK OS=Homo sapiens GN=MAFK PE=1 SV=	0.978	1.093	0.701	0.530	1.010	1.295	0.883	0.664
P49720	Proteasome subunit beta type-3 OS=Homo sapiens GN=PSMB3 P	1.535	0.990	1.378	1.147	1.548	1.074	1.310	1.131
Q92506	Estradiol 17-beta-dehydrogenase 8 OS=Homo sapiens GN=HSD1	1.227	1.206	1.149	1.171	1.314	1.163	1.339	1.292
B7Z5J7	cDNA FLJ58682, highly similar to Vesicle-fusing ATPase (EC 3.6.4	1.736	1.346	1.521	1.483	1.578	1.249	1.742	1.756
A4D1E9	GTP-binding protein 10 OS=Homo sapiens GN=GTPBP10 PE=1 S\	1.581	1.611	1.336	1.216	1.154	1.317	1.589	1.434
O95782	AP-2 complex subunit alpha-1 OS=Homo sapiens GN=AP2A1 PE=	1.526	1.483	1.413	1.062	1.262	1.096	1.382	1.336
Q68D38	Putative uncharacterized protein DKFZp686O15119 (Fragment) O	1.656	1.578	1.280	1.084	1.313	1.296	1.437	1.397
Q9NVS2	28S ribosomal protein S18a, mitochondrial OS=Homo sapiens GN	1.256	1.204	1.233	0.982	0.971	1.104	1.173	1.326
P51153	Ras-related protein Rab-13 OS=Homo sapiens GN=RAB13 PE=1 S	1.521	1.518	1.199	1.176	1.090	1.129	1.420	0.990
K7EM56	40S ribosomal protein S15 OS=Homo sapiens GN=RPS15 PE=1 S	1.440	1.294	1.316	0.736	1.390	1.030	1.189	1.184
F8W8K9	AP-1 complex subunit sigma-3 OS=Homo sapiens GN=AP1S3 PE=	1.963	1.828	1.414	1.386	1.606	1.240	1.658	1.509
D3DUJ0	AFG3 ATPase family gene 3-like 2 (Yeast), isoform CRA_a (Fragm	1.248	1.402	1.139	1.232	0.962	1.072	1.202	1.191

Q96EI5	Transcription elongation factor A protein-like 4 OS=Homo sapiens	1.211	1.027	0.914	0.652	1.254	1.098	1.057	0.833
A8K6U7	cDNA FLJ78252, highly similar to Homo sapiens heterogeneous n	1.378	1.476	1.194	0.808	1.364	1.263	1.356	0.947
O95639	Cleavage and polyadenylation specificity factor subunit 4 OS=Hor	1.867	1.537	1.804	1.246	1.356	1.374	1.655	1.573
Q96E11	Ribosome-recycling factor, mitochondrial OS=Homo sapiens GN=	1.133	1.244	1.004	0.979	0.954	1.099	1.081	1.080
B4DT46	cDNA FLJ57889, highly similar to Cytochrome b-245 light chain O	1.392	0.951	0.493	0.695	0.946	0.857	0.847	0.763
B5BU53	Cyclin-dependent kinase 9 OS=Homo sapiens GN=CDK9 PE=2 SV	1.504	1.457	1.453	1.062	1.134	1.216	1.446	1.133
P20618	Proteasome subunit beta type-1 OS=Homo sapiens GN=PSMB1 P	1.544	1.179	1.292	1.048	1.494	1.136	1.195	1.137
P51398	28S ribosomal protein S29, mitochondrial OS=Homo sapiens GN=	1.550	1.657	1.377	1.368	1.253	1.170	1.416	1.491
Q9H4M9	EH domain-containing protein 1 OS=Homo sapiens GN=EHD1 PE	1.674	1.050	1.191	0.918	1.271	1.009	1.067	0.989
P08579	U2 small nuclear ribonucleoprotein B'' OS=Homo sapiens GN=SN	1.452	1.356	1.217	0.983	0.903	1.224	1.144	0.749
A0A024R7L5	UPF1 regulator of nonsense transcripts homolog (Yeast), isoform	1.586	1.577	1.558	1.114	1.379	1.327	1.625	1.391
G8JLQ3	Biogenesis of lysosome-related organelles complex 1 subunit 1 O	1.211	1.176	1.165	1.020	1.274	1.441	1.157	1.078
Q93009	Ubiquitin carboxyl-terminal hydrolase 7 OS=Homo sapiens GN=U	1.374	1.479	1.469	1.350	1.422	1.332	1.611	1.735
Q9GZR2	RNA exonuclease 4 OS=Homo sapiens GN=REXO4 PE=1 SV=2 -	1.330	1.662	1.361	1.204	1.388	1.457	1.628	1.486
P03253	Adenain 23K protease from Ad 5 transcriptome GN=23KPro - [AD		0.412	1.628	3.541	0.641	0.952	0.962	1.764
P46087	Probable 28S rRNA (cytosine(4447)-C(5))-methyltransferase OS=	1.457	1.813	1.236	1.022	1.353	1.351	1.387	1.212
Q13439	Golgin subfamily A member 4 OS=Homo sapiens GN=GOLGA4 PE	1.490	1.598	1.326	1.272	1.436	1.351	1.428	1.566
Q9BQG0	Myb-binding protein 1A OS=Homo sapiens GN=MYBBP1A PE=1 S	0.964	1.317	1.011	0.910	1.573	0.788	0.676	0.960
Q13616	Cullin-1 OS=Homo sapiens GN=CUL1 PE=1 SV=2 - [CUL1_HUMA	1.242	1.215	1.157	0.878	1.192	1.120	1.075	0.978
E5KLJ7	Mitochondrial dynamin-like 120 kDa protein OS=Homo sapiens GI	1.253	1.383	1.272	1.112	1.255	1.191	1.403	1.377
Q9Y2X3	Nucleolar protein 58 OS=Homo sapiens GN=NOP58 PE=1 SV=1 -	1.790	1.860	1.494	1.424	1.663	1.413	1.585	1.476
B3KQA0	cDNA FLJ90015 fis, clone HEMBA1000634, highly similar to Homo	2.035	2.260	3.992	2.832	2.559	1.604	1.799	1.653
P49916	DNA ligase 3 OS=Homo sapiens GN=LIG3 PE=1 SV=2 - [DNLI3_	1.242	1.462	1.552	1.471	1.035	1.150	1.342	1.394
Q9H1K1	Iron-sulfur cluster assembly enzyme ISCU, mitochondrial OS=Hor	1.152	1.222	0.943	0.910	1.018	1.172	1.197	1.200
E5KND7	Elongation factor G, mitochondrial OS=Homo sapiens GN=GFM1	1.401	1.501	1.225	1.137	1.160	1.219	1.459	1.251
B2RE46	cDNA, FLJ96923, highly similar to Homo sapiens ribophorin II (RI	1.224	1.275	1.220	1.136	1.116	1.135	1.251	1.107
Q13217	DnaJ homolog subfamily C member 3 OS=Homo sapiens GN=DN	1.583	1.373	1.398	1.335	1.351	1.244	1.391	1.346
Q16531	DNA damage-binding protein 1 OS=Homo sapiens GN=DDB1 PE=	1.487	1.511	1.442	1.192	1.439	1.204	1.344	1.419
E7EX90	Uncharacterized protein OS=Homo sapiens GN=DCTN1 PE=1 SV=	1.446	1.040	1.299	0.969	1.317	1.091	1.196	1.087
P52815	39S ribosomal protein L12, mitochondrial OS=Homo sapiens GN=	1.374	1.324	1.139	1.188	1.125	1.132	1.298	1.187
P60953	Cell division control protein 42 homolog OS=Homo sapiens GN=C	1.324	1.355	1.322	0.967	1.127	1.150	1.206	1.172
F1T0J2	Golgin subfamily B member 1 OS=Homo sapiens GN=GOLGB1 PE	1.521	1.683	1.450	1.405	1.207	1.440	1.671	1.467
P62304	Small nuclear ribonucleoprotein E OS=Homo sapiens GN=SNRPE	1.177	1.251	1.023	0.936	1.281	0.998	1.172	1.036
Q9HBH1	Peptide deformylase, mitochondrial OS=Homo sapiens GN=PDF F	1.389	1.512	1.224	1.305	1.009	1.256	1.332	1.392
O43290	U4/U6.U5 tri-snRNP-associated protein 1 OS=Homo sapiens GN=	1.619	1.800	1.269	1.052	1.309	1.343	1.516	1.409
Q5VU21	PAI-1 mRNA-binding protein variant OS=Homo sapiens PE=2 SV=	1.660	1.545	1.960	1.208	1.342	1.086	1.447	1.307
C7DJS2	Glutathione S-transferase pi (Fragment) OS=Homo sapiens GN=C	0.840	0.595	0.982	0.845	0.907	1.057	0.854	0.839
O43447	Peptidyl-prolyl cis-trans isomerase H OS=Homo sapiens GN=PPIH	1.571	1.310	1.210	1.000	1.310	1.293	1.348	1.250
Q6P1J9	Parafibromin OS=Homo sapiens GN=CDC73 PE=1 SV=1 - [CDC7:	1.433	1.753	1.209	0.846	1.358	1.325	1.263	1.005
P30837	Aldehyde dehydrogenase X, mitochondrial OS=Homo sapiens GN	1.242	1.347	1.108	1.245	0.992	1.192	1.305	1.286

Q59EC0	Adenosine deaminase, RNA-specific isoform ADAR-a variant (Frag	0.836	0.970	0.872	0.761	1.210	1.068	0.974	
P47985	Cytochrome b-c1 complex subunit Rieske, mitochondrial OS=Hom	1.314	1.247	1.016	1.078	0.994	1.091	1.184	1.150
A8K2R3	cDNA FLJ75083, highly similar to Homo sapiens amine oxidase (fl	1.772	1.494	1.038	0.735	1.617	1.228	1.357	1.174
Q53FH8	Sorting nexin 5 variant (Fragment) OS=Homo sapiens PE=2 SV=	1.528	1.079	1.563	1.182	1.366	1.118	1.606	1.187
D3DQ38	Chromosome 1 open reading frame 123, isoform CRA_a OS=Hom	1.501	0.933	1.977	1.592	1.418	1.111	1.492	1.398
P55039	Developmentally-regulated GTP-binding protein 2 OS=Homo sapi	1.428	1.002	1.412	1.160	1.224	1.069	1.451	1.209
Q8IUE6	Histone H2A type 2-B OS=Homo sapiens GN=HIST2H2AB PE=1 S	1.258	1.306	0.953	0.928	0.949	1.227	1.086	0.907
Q9Y3A2	Probable U3 small nucleolar RNA-associated protein 11 OS=Homo	1.474	2.013	1.046	0.706	1.368	1.246	1.222	0.783
Q9Y263	Phospholipase A-2-activating protein OS=Homo sapiens GN=PLA/	1.250	1.095	1.097	1.061	1.327	1.115	1.202	1.003
E9PRZ9	Small acidic protein (Fragment) OS=Homo sapiens GN=C11orf58	1.795	1.120	2.237	1.482	2.239	1.133	1.709	1.476
O60488	Long-chain-fatty-acid--CoA ligase 4 OS=Homo sapiens GN=ACSL4	1.363	1.536	1.376	1.155	1.170	1.221	1.330	1.565
O15371	Eukaryotic translation initiation factor 3 subunit D OS=Homo sapi	2.210	2.320	2.161	1.567	1.681	1.306	1.800	1.629
P24752	Acetyl-CoA acetyltransferase, mitochondrial OS=Homo sapiens GI	1.357	1.397	1.174	1.120	1.085	1.128	1.318	1.254
B2WTI3	Bifunctional arginine demethylase and lysyl-hydroxylase JMJD6 O	1.711	1.501	1.361	1.025	1.670	1.302	1.386	1.205
O95831	Apoptosis-inducing factor 1, mitochondrial OS=Homo sapiens GN	1.255	1.292	1.134	1.264	1.005	1.121	1.253	1.301
P98179	Putative RNA-binding protein 3 OS=Homo sapiens GN=RBM3 PE=	1.750	1.694	1.336	1.142	1.642	2.098	1.933	1.260
A8K4A8	cDNA FLJ76156, highly similar to Homo sapiens aspartyl-tRNA syr	1.231	1.319	1.100	1.090	0.940	1.103	1.195	1.196
P61163	Alpha-centractin OS=Homo sapiens GN=ACTR1A PE=1 SV=1 - [A	1.595	1.206	1.342	1.224	1.578	1.201	1.474	1.538
A0A087WWS1	THO complex subunit 1 OS=Homo sapiens GN=THOC1 PE=4 SV=	1.473	1.608	1.277	1.076	1.341	1.319	1.494	1.596
B2RNR6	Zinc finger RNA binding protein OS=Homo sapiens GN=ZFR PE=2	1.576	1.693	1.201	0.907	1.536	1.465	1.486	0.995
Q9NYU2	UDP-glucose:glycoprotein glucosyltransferase 1 OS=Homo sapien	1.429	1.011	1.311	1.295	1.352	1.175	1.376	1.440
Q99567	Nuclear pore complex protein Nup88 OS=Homo sapiens GN=NUF	2.119	1.731	1.485	2.208	2.003	1.422	2.248	2.267
Q5LJA5	Ubiquitin carboxyl-terminal hydrolase OS=Homo sapiens GN=UCH	1.396	1.180	1.248	0.872	1.370	1.065	1.286	1.065
Q14376	UDP-glucose 4-epimerase OS=Homo sapiens GN=GALE PE=1 SV	1.865	1.341	1.706	1.408	1.573	1.038	1.544	1.386
Q16831	Uridine phosphorylase 1 OS=Homo sapiens GN=UPP1 PE=1 SV=	1.401	1.025	1.034	0.714	1.046	0.975	0.878	0.679
B4DT15	cDNA FLJ56150, highly similar to Zinc phosphodiesterase ELAC pr	1.350	1.381	1.340	1.013	1.234	1.248	1.308	1.005
Q9HCC0	Methylcrotonoyl-CoA carboxylase beta chain, mitochondrial OS=H	1.286	1.521	1.155	1.117	1.064	1.219	1.482	1.214
O75306	NADH dehydrogenase [ubiquinone] iron-sulfur protein 2, mitocho	1.456	1.225	1.309	1.296	1.115	1.259	1.366	1.302
O75828	Carbonyl reductase [NADPH] 3 OS=Homo sapiens GN=CBR3 PE=	1.407	1.443	2.127	2.227	1.228	1.108	1.379	1.813
O15305	Phosphomannomutase 2 OS=Homo sapiens GN=PMM2 PE=1 SV=	1.167	0.849	1.308	1.064	1.101	1.054	1.134	1.116
P67870	Casein kinase II subunit beta OS=Homo sapiens GN=CSNK2B PE	3.573	3.427	3.640	3.062	2.258	1.227	2.243	3.319
P49458	Signal recognition particle 9 kDa protein OS=Homo sapiens GN=S	1.625	1.901	1.885	1.661	1.504	1.303	1.641	1.845
Q9UJ70	N-acetyl-D-glucosamine kinase OS=Homo sapiens GN=NAGK PE=	1.250	0.772	1.246	1.094	1.149	1.013	1.243	1.246
A8K9K8	cDNA FLJ75823, highly similar to Homo sapiens dimethyladenosir	1.287	1.533	1.005	0.807	1.382	1.276	1.196	0.945
Q9BU61	NADH dehydrogenase [ubiquinone] 1 alpha subcomplex assembly	1.358	1.498	1.234	1.354	1.038	1.384	1.546	1.497
P18615	Negative elongation factor E OS=Homo sapiens GN=NELFE PE=1	1.207	1.229	0.840	0.634	1.154	1.221	1.051	0.782
Q99848	Probable rRNA-processing protein EBP2 OS=Homo sapiens GN=E	1.524	1.630	1.320	1.199	1.485	1.353	1.571	1.501
A8K9T8	cDNA FLJ76106, highly similar to Homo sapiens neurolysin (meta	1.590	1.377	1.368	1.362	1.312	1.240	1.349	1.282
A3F768	NF-kappaB repressing factor OS=Homo sapiens GN=NRF PE=2 S	1.548	1.658	1.245	0.978	1.493	1.454	1.352	1.119
Q13948	Protein CASP OS=Homo sapiens GN=CUX1 PE=1 SV=2 - [CASP_I	1.374	1.453	1.498	1.151	1.142	1.101	1.438	1.419

P31040	Succinate dehydrogenase [ubiquinone] flavoprotein subunit, mito	1.283	1.344	1.048	1.117	1.086	1.112	1.352	1.324
Q2VIN3	RBM1 (Fragment) OS=Homo sapiens PE=4 SV=1 - [Q2VIN3_HUM	1.407	1.297	1.222	1.003	1.359	1.262	1.473	1.348
B2R5Y4	cDNA, FLJ92684, highly similar to Homo sapiens IK cytokine, dow	1.347	1.681	1.176	0.985	1.352	1.371	1.491	1.193
Q9Y3B4	Splicing factor 3B subunit 6 OS=Homo sapiens GN=SF3B6 PE=1	1.035	1.146	0.904	0.678	1.034	1.178	0.962	0.748
A8K984	Structural maintenance of chromosomes protein OS=Homo sapie	1.269	1.047	1.110	0.764	1.121	1.067	1.111	0.928
Q86UE4	Protein LYRIC OS=Homo sapiens GN=MTDH PE=1 SV=2 - [LYRIC	1.406	1.473	1.159	1.101	1.127	1.238	1.101	1.070
Q13405	39S ribosomal protein L49, mitochondrial OS=Homo sapiens GN=	1.309	1.501	1.155	1.061	0.983	1.216	1.345	1.351
Q86X69	PON2 protein OS=Homo sapiens PE=2 SV=1 - [Q86X69_HUMAN]	0.494	0.806	0.691	0.609	0.918	1.270	0.876	0.612
D3DVF0	F11 receptor, isoform CRA_a OS=Homo sapiens GN=F11R PE=4 S	0.963	1.314	1.192	1.442	0.943	1.160	1.382	1.521
H3BNT4	M-phase phosphoprotein 6 OS=Homo sapiens GN=MPHOSPH6 PE	1.334	1.508	1.423	1.305	1.360	1.385	1.295	1.237
Q9H4A4	Aminopeptidase B OS=Homo sapiens GN=RNPEP PE=1 SV=2 - [A	1.296	0.966	1.345	1.168	1.180	0.951	1.234	1.136
Q9NUJ1	Mycophenolic acid acyl-glucuronide esterase, mitochondrial OS=H	1.571	1.756	1.524	1.465	1.238	1.211	1.573	1.576
Q15459	Splicing factor 3A subunit 1 OS=Homo sapiens GN=SF3A1 PE=1	1.648	1.480	1.205	1.047	1.469	1.279	1.423	1.093
O75533	Splicing factor 3B subunit 1 OS=Homo sapiens GN=SF3B1 PE=1	1.479	1.703	1.349	0.975	1.434	1.350	1.515	1.118
O76094	Signal recognition particle subunit SRP72 OS=Homo sapiens GN=	1.407	1.130	1.229	0.866	1.350	1.096	1.130	1.141
Q70UQ0	Inhibitor of nuclear factor kappa-B kinase-interacting protein OS=	1.132	1.307	0.953	0.815	1.032	1.112	1.109	1.016
Q9NYK5	39S ribosomal protein L39, mitochondrial OS=Homo sapiens GN=	1.487	1.451	1.422	1.343	1.084	1.158	1.354	1.329
Q15007	Pre-mRNA-splicing regulator WTAP OS=Homo sapiens GN=WTAP	1.682	1.619	1.297	0.940	1.380	1.385	1.636	1.384
P04899	Guanine nucleotide-binding protein G(i) subunit alpha-2 OS=Hom	5.560	7.321	5.680	6.378	3.370	1.833	4.160	5.053
P36551	Oxygen-dependent coproporphyrinogen-III oxidase, mitochondria	1.235	1.344	1.174	1.207	1.188	1.244	1.309	1.299
Q9H2U2	Inorganic pyrophosphatase 2, mitochondrial OS=Homo sapiens G	2.301	2.332	2.250	2.018	1.681	1.252	2.083	2.439
Q02252	Methylmalonate-semialdehyde dehydrogenase [acylating], mitoch	1.297	1.190	1.271	1.214	0.941	1.166	1.335	1.290
Q92552	28S ribosomal protein S27, mitochondrial OS=Homo sapiens GN=	1.892	2.136	1.658	1.540	1.351	1.349	1.864	1.851
Q9HB66	Alternative protein MKKS OS=Homo sapiens GN=MKKS PE=2 SV=	1.626	1.583	1.374	1.230	1.258	1.247	1.460	1.430
J9R021	Eukaryotic translation initiation factor 3 subunit A OS=Homo sapi	1.384	1.377	1.310	0.944	1.217	1.130	1.210	0.996
G3V1P3	Loss of heterozygosity 12 chromosomal region 1 protein OS=Hom	1.647	1.783	1.564	1.484	1.597	1.284	1.206	1.324
P43897	Elongation factor Ts, mitochondrial OS=Homo sapiens GN=TSFM	1.624	1.868	1.631	1.204	1.425	1.347	1.804	1.341
Q96EY7	Pentatricopeptide repeat domain-containing protein 3, mitochond	1.175	1.378	0.998	1.125	0.882	0.954	1.111	1.172
A8K7D9	Importin subunit alpha OS=Homo sapiens PE=2 SV=1 - [A8K7D9	1.595	1.188	1.552	1.165	1.370	1.474	1.833	1.692
Q5RKV6	Exosome complex component MTR3 OS=Homo sapiens GN=EXOS	1.562	1.568	1.242	1.031	1.650	1.222	1.459	1.491
P48735	Isocitrate dehydrogenase [NADP], mitochondrial OS=Homo sapie	1.221	1.401	1.192	1.212	1.041	1.331	1.656	1.437
O95202	LETM1 and EF-hand domain-containing protein 1, mitochondrial O	1.403	1.650	1.220	1.337	1.106	1.139	1.381	1.374
O75380	NADH dehydrogenase [ubiquinone] iron-sulfur protein 6, mitocho	3.341	3.432	2.757	2.985	2.169	1.467	3.098	3.317
P61160	Actin-related protein 2 OS=Homo sapiens GN=ACTR2 PE=1 SV=1	1.551	1.375	1.371	1.061	1.438	1.086	1.325	1.055
Q96PK6	RNA-binding protein 14 OS=Homo sapiens GN=RBM14 PE=1 SV=	1.510	1.715	1.393	1.452	1.447	1.543	1.738	1.678
J3QQS6	Cdc42 effector protein 4 (Fragment) OS=Homo sapiens GN=CDC	0.967	0.857	1.122	0.741	1.372	1.060	1.077	0.887
E9PDF6	Unconventional myosin-Ib OS=Homo sapiens GN=MYO1B PE=1 S	1.664	1.207	1.181	0.976	1.384	1.131	1.307	1.165
B2R4N3	cDNA, FLJ92155, highly similar to Homo sapiens ubiquitin-like 5 (	1.690	1.436	1.442	1.091	1.773	1.624	1.390	1.032
O95168	NADH dehydrogenase [ubiquinone] 1 beta subcomplex subunit 4	0.972	1.221	0.894	0.908	0.825	0.904	1.066	0.976
Q13547	Histone deacetylase 1 OS=Homo sapiens GN=HDAC1 PE=1 SV=1	2.092	1.436	1.538	0.957	1.647	1.181	1.930	1.775

B4E3A8	cDNA FLJ53963, highly similar to Leukocyte elastase inhibitor OS=	1.237	0.815	1.179	0.930	1.044	0.903	1.084	0.928
P62263	40S ribosomal protein S14 OS=Homo sapiens GN=RPS14 PE=1 S	2.000	2.139	2.007	1.378	1.704	1.370	1.752	1.426
Q8IVS2	Malonyl-CoA-acyl carrier protein transacylase, mitochondrial OS=I	2.223	2.313	1.811	2.124	1.322	1.374	2.490	2.110
Q9UKX7	Nuclear pore complex protein Nup50 OS=Homo sapiens GN=NUP	1.562	1.532	1.361	1.284	1.378	1.317	1.413	1.245
Q14558	Phosphoribosyl pyrophosphate synthase-associated protein 1 OS=	1.157	0.804	1.055	0.878	0.999	0.983	1.043	0.879
M0R0F0	40S ribosomal protein S5 (Fragment) OS=Homo sapiens GN=RPS	1.414	1.278	1.200	0.820	1.437	1.326	1.428	1.057
P04181	Ornithine aminotransferase, mitochondrial OS=Homo sapiens GN	1.201	1.433	1.112	1.087	1.034	1.210	1.319	1.483
B4DF93	Mitochondrial GTPase 1 OS=Homo sapiens PE=2 SV=1 - [B4DF93	1.465	1.802	1.297	1.128	1.032	1.324	1.518	1.223
Q15166	Serum paraoxonase/lactonase 3 OS=Homo sapiens GN=PON3 PE	1.086	1.050	1.114	1.116	0.934	1.054	1.231	1.047
B2R9K5	cDNA, FLJ94435 OS=Homo sapiens PE=2 SV=1 - [B2R9K5_HUMA	1.450	1.824	0.918	0.685	1.398	1.395	1.460	0.897
P18031	Tyrosine-protein phosphatase non-receptor type 1 OS=Homo sap	1.244	1.499	0.980	0.904	1.001	1.228	1.214	0.997
O60216	Double-strand-break repair protein rad21 homolog OS=Homo sap	1.797	1.868	1.431	1.281	1.597	1.373	1.774	1.916
B2RAX6	cDNA, FLJ95176, Homo sapiens CGI-48 protein (CGI-48), mRNA (	1.165	1.471	1.247	0.916	1.210	1.310	1.389	1.178
P52758	Ribonuclease UK114 OS=Homo sapiens GN=HRSP12 PE=1 SV=1	1.561	1.417	1.333	1.409	1.213	1.223	1.532	1.494
P46779	60S ribosomal protein L28 OS=Homo sapiens GN=RPL28 PE=1 S	1.561	1.429	1.475	1.080	1.469	1.270	1.391	1.273
Q8IXM3	39S ribosomal protein L41, mitochondrial OS=Homo sapiens GN=	1.038	1.178	0.850	1.099	0.787	1.156	1.108	0.895
Q9BR63	FARSB protein (Fragment) OS=Homo sapiens GN=FARSB PE=2 S	1.531	1.193	1.364	1.021	1.275	1.219	1.288	1.102
Q5JTJ3	Cytochrome c oxidase assembly factor 6 homolog OS=Homo sapi	1.532	1.522	1.503	1.460	1.408	1.384	1.574	1.526
Q96DZ1	Endoplasmic reticulum lectin 1 OS=Homo sapiens GN=ERLEC1 PE	1.540	1.675	1.507	1.442	1.389	1.254	1.439	1.417
P62269	40S ribosomal protein S18 OS=Homo sapiens GN=RPS18 PE=1 S	1.434	1.259	1.236	0.864	1.312	1.352	1.279	1.180
E9PMS6	LIM domain only protein 7 OS=Homo sapiens GN=LMO7 PE=1 SV	1.423	1.505	0.900	0.651	1.239	1.201	1.006	0.881
Q9NWU5	39S ribosomal protein L22, mitochondrial OS=Homo sapiens GN=	1.319	1.589	1.208	1.053	1.097	1.161	1.420	1.270
B7ZAQ5	cDNA, FLJ79269, highly similar to DNA polymerase subunit delta	1.375	1.138	1.100	1.013	1.203	1.055	1.047	0.947
O00142	Thymidine kinase 2, mitochondrial OS=Homo sapiens GN=TK2 PE	0.905	1.281	1.408	1.179	0.873	1.239	1.089	1.139
B2R7M1	cDNA, FLJ93507, highly similar to Homo sapiens ATPase, H+ tran	1.737	1.967	1.642	1.694	1.744	1.177	1.825	1.904
Q96A35	39S ribosomal protein L24, mitochondrial OS=Homo sapiens GN=	1.228	1.279	1.040	1.071	0.912	1.087	1.150	1.108
A8K4K9	cDNA FLJ76169 OS=Homo sapiens PE=2 SV=1 - [A8K4K9_HUMA	1.245	1.453	1.279	1.076	1.104	1.134	1.293	1.124
O15525	Transcription factor MafG OS=Homo sapiens GN=MAFG PE=1 SV	1.607	1.564	0.996	0.707	1.526	1.146	1.106	0.959
P00568	Adenylate kinase isoenzyme 1 OS=Homo sapiens GN=AK1 PE=1	1.043	0.806	1.225	1.022	0.994	0.955	1.024	1.079
Q9H3K6	BolA-like protein 2 OS=Homo sapiens GN=BOLA2 PE=1 SV=1 - [	1.207	0.837	1.359	1.008	1.031	0.798	1.100	1.023
P84101	Small EDRK-rich factor 2 OS=Homo sapiens GN=SERF2 PE=1 SV=	1.241	1.216	1.135	0.486	1.016	1.359	1.048	
A8K492	cDNA FLJ76789, highly similar to Homo sapiens methionine-tRNA	1.254	1.069	1.160	0.866	1.058	1.005	1.056	1.038
A8K132	cDNA FLJ75476, highly similar to Homo sapiens glutaminase (GLS	1.536	1.559	1.120	1.145	1.285	1.351	1.443	1.163
L0R819	Alternative protein ASNSD1 OS=Homo sapiens GN=ASNSD1 PE=4	1.747	1.293	1.766	1.113	1.452	1.258	1.438	1.295
P11172	Uridine 5'-monophosphate synthase OS=Homo sapiens GN=UMPS	1.241	0.991	1.279	1.132	1.176	1.084	1.282	1.171
B4DJX1	Acetyltransferase component of pyruvate dehydrogenase complex	1.443	1.477	1.223	1.298	1.173	1.142	1.481	1.446
O43819	Protein SCO2 homolog, mitochondrial OS=Homo sapiens GN=SCO	1.147	1.289	0.938	1.006	0.880	1.069	1.173	1.140
O60508	Pre-mRNA-processing factor 17 OS=Homo sapiens GN=CDC40 PE	2.044	2.064	1.514	1.360	1.836	1.500	1.966	1.505
Q15393	Splicing factor 3B subunit 3 OS=Homo sapiens GN=SF3B3 PE=1 S	1.486	1.493	1.299	0.989	1.464	1.281	1.373	1.143
B2R739	cDNA, FLJ93269, highly similar to Homo sapiens mitochondrial rib	1.454	1.713	1.229	1.377	1.086	1.293	1.473	1.388

P50552	Vasodilator-stimulated phosphoprotein OS=Homo sapiens GN=VA	1.294	0.659	0.869	0.625	1.173	0.895	0.859	0.656
M4VP52	Apolipoprotein B editing enzyme catalytic polypeptide-like 3C OS=	1.993	1.799	1.898	1.499	2.009	1.042	1.926	1.712
Q9NUF9	Nucleoside diphosphate kinase (Fragment) OS=Homo sapiens GN	1.396	1.419	1.405	1.446	1.196	1.174	1.494	1.546
P24666	Low molecular weight phosphotyrosine protein phosphatase OS=	0.430	0.521	0.573	0.677	0.896	0.983	0.978	0.675
B3KN06	Coronin OS=Homo sapiens PE=2 SV=1 - [B3KN06_HUMAN]	1.937	1.358	1.337	1.005	1.827	1.230	1.380	1.335
B4DUD5	cDNA FLJ58787, highly similar to Cleavage stimulation factor 64 k	1.227	1.292	0.920	0.674	1.253	1.256	1.154	1.059
Q96MF7	E3 SUMO-protein ligase NSE2 OS=Homo sapiens GN=NSMCE2 PE	1.619	1.470	1.309	1.191	1.395	1.192	1.583	1.371
Q9UFN0	Protein NipSnap homolog 3A OS=Homo sapiens GN=NIPSNAP3A	5.239	6.009	4.990	4.954	1.721	0.964	3.013	4.067
Q9Y2S7	Polymerase delta-interacting protein 2 OS=Homo sapiens GN=PO	1.249	1.354	1.102	0.964	0.943	1.258	1.186	1.107
O75131	Copine-3 OS=Homo sapiens GN=CPNE3 PE=1 SV=1 - [CPNE3_HU	1.120	0.821	1.084	1.105	1.076	0.992	1.352	1.079
Q9NX40	OCIA domain-containing protein 1 OS=Homo sapiens GN=OCIAD	1.133	1.166	1.205	1.153	1.290	1.445	1.340	1.000
O43169	Cytochrome b5 type B OS=Homo sapiens GN=CYB5B PE=1 SV=2	3.195	2.885	2.995	2.845	2.313	1.604	2.784	2.435
Q9HD33	39S ribosomal protein L47, mitochondrial OS=Homo sapiens GN=	1.597	1.724	1.373	1.583	1.323	1.179	1.714	1.646
B4DY50	cDNA FLJ55528, highly similar to Transducin beta-like 2 protein C	1.436	1.582	1.375	0.963	1.143	1.233	1.428	1.056
P09661	U2 small nuclear ribonucleoprotein A' OS=Homo sapiens GN=SNI	1.541	1.544	1.196	1.015	1.424	1.304	1.401	1.278
B3KNS4	HCG2043597, isoform CRA_a OS=Homo sapiens GN=hCG_20435	1.401	1.502	1.587	1.504	1.485	1.435	1.652	1.514
O75530	Polycomb protein EED OS=Homo sapiens GN=EED PE=1 SV=2 -	1.421	1.527	1.386	1.168	1.383	1.461	1.538	1.317
P27144	Adenylate kinase 4, mitochondrial OS=Homo sapiens GN=AK4 PE	1.228	1.385	1.238	1.211	1.018	1.202	1.408	1.278
P40616	ADP-ribosylation factor-like protein 1 OS=Homo sapiens GN=ARL	2.343	1.573	1.862	1.865	2.344	1.238	1.572	2.082
P49448	Glutamate dehydrogenase 2, mitochondrial OS=Homo sapiens GN	1.448	1.917	1.399	1.607	1.141	1.602	1.820	1.842
Q07960	Rho GTPase-activating protein 1 OS=Homo sapiens GN=ARHGAP	1.236	0.845	1.131	0.899	0.986	0.983	1.098	1.019
Q16777	Histone H2A type 2-C OS=Homo sapiens GN=HIST2H2AC PE=1 S	1.296	1.067	0.955	0.615	1.050	1.204	0.999	1.171
P49721	Proteasome subunit beta type-2 OS=Homo sapiens GN=PSMB2 P	2.324	1.404	1.532	1.317	1.739	1.110	1.855	1.799
P12931	Proto-oncogene tyrosine-protein kinase Src OS=Homo sapiens GN	1.135	0.903	0.901	0.804	1.238	1.086	1.093	0.878
B4DRW1	cDNA FLJ55805, highly similar to Keratin, type II cytoskeletal 4 O	1.450	1.740	1.230	0.727	1.308	1.121	1.417	0.548
P23786	Carnitine O-palmitoyltransferase 2, mitochondrial OS=Homo sapie	1.690	1.558	1.359	1.468	1.193	1.279	1.501	1.502
O43837	Isocitrate dehydrogenase [NAD] subunit beta, mitochondrial OS=	1.851	1.862	1.855	1.929	1.370	1.341	1.854	1.811
P82675	28S ribosomal protein S5, mitochondrial OS=Homo sapiens GN=N	1.376	1.362	1.021	0.949	1.031	1.095	1.214	1.185
P52565	Rho GDP-dissociation inhibitor 1 OS=Homo sapiens GN=ARHGDI/	1.390	0.958	1.635	1.347	1.102	0.949	1.284	1.329
B2R8A2	cDNA, FLJ93804, highly similar to Homo sapiens gp25L2 protein (	0.544	0.653	0.580	0.598	0.899	1.283	0.947	0.577
Q15717	ELAV-like protein 1 OS=Homo sapiens GN=ELAVL1 PE=1 SV=2 -	1.306	1.303	0.909	0.590	1.314	1.153	1.108	0.731
O96008	Mitochondrial import receptor subunit TOM40 homolog OS=Homo	1.306	1.520	1.173	1.288	1.029	1.216	1.486	1.301
Q8IY31	Intraflagellar transport protein 20 homolog OS=Homo sapiens GN	0.965	1.131	0.977	0.716	0.951	0.938	0.893	0.608
B2RDP6	cDNA, FLJ96709, highly similar to Homo sapiens glutamate rich W	1.973	1.773	1.940	1.613	1.727	1.694	1.892	2.181
J3KN29	26S proteasome non-ATPase regulatory subunit 9 OS=Homo sapi	1.395	0.887	1.370	1.140	1.212	1.072	1.216	1.205
B4DEH0	Mitochondrial ribosomal protein L10, isoform CRA_d OS=Homo sa	1.493	1.746	1.212	1.203	1.260	1.250	1.431	1.458
P45954	Short/branched chain specific acyl-CoA dehydrogenase, mitochon	1.247	1.369	1.405	1.443	1.015	1.285	1.374	1.402
Q06124	Tyrosine-protein phosphatase non-receptor type 11 OS=Homo sa	1.521	1.073	1.450	1.105	1.259	1.054	1.346	1.239
B1AK13	3-hydroxymethyl-3-methylglutaryl-Coenzyme A lyase (Hydroxyme	0.976	0.885	0.737	0.710	0.740	1.019	0.840	0.898
P82932	28S ribosomal protein S6, mitochondrial OS=Homo sapiens GN=N	1.630	1.645	1.653	1.504	1.272	1.133	1.474	1.503

B4DEX5	Prion protein interacting protein, isoform CRA_d OS=Homo sapie	1.477	1.220	1.364	1.011	1.319	1.304	1.406	1.309
Q8N0T1	Uncharacterized protein C8orf59 OS=Homo sapiens GN=C8orf59	1.906	2.351	1.767	0.859	2.039	1.909	2.408	1.996
Q7Z2W9	39S ribosomal protein L21, mitochondrial OS=Homo sapiens GN=	1.221	1.460	1.248	1.482	1.144	1.034	1.235	1.396
Q8WXI9	Transcriptional repressor p66-beta OS=Homo sapiens GN=GATAD	0.922	1.057	0.693	0.669	0.921	1.124	0.904	0.826
P60891	Ribose-phosphate pyrophosphokinase 1 OS=Homo sapiens GN=F	1.843	0.998	1.649	1.471	1.958	1.017	1.628	1.536
P51114	Fragile X mental retardation syndrome-related protein 1 OS=Hom	1.751	1.633	1.608	1.358	1.560	1.158	1.632	1.363
B2RD09	cDNA, FLJ96406, highly similar to Homo sapiens NOL1/NOP2/Sur	1.669	1.648	1.142	0.731	1.365	1.179	1.177	1.128
O60832	H/ACA ribonucleoprotein complex subunit 4 OS=Homo sapiens GI	1.411	1.551	1.117	1.118	1.357	1.323	1.541	1.299
A8K3B0	cDNA FLJ77877, highly similar to Human ENO2 neuron specific (g	3.273	1.935	3.053	2.434	1.881	1.058	1.799	2.283
P46776	60S ribosomal protein L27a OS=Homo sapiens GN=RPL27A PE=1	2.066	1.632	1.821	1.392	1.743	1.190	1.704	1.517
P06280	Alpha-galactosidase A OS=Homo sapiens GN=GLA PE=1 SV=1 -	1.321	1.232	1.354	1.338	1.450	1.175	1.515	1.466
Q05DU1	GNL3L protein (Fragment) OS=Homo sapiens GN=GNL3L PE=2 S	2.060	2.815	2.335	1.798	1.699	1.703	2.687	2.278
O00161	Synaptosomal-associated protein 23 OS=Homo sapiens GN=SNAI	1.648	1.989	2.114	1.786	1.224	1.311	1.589	1.766
H0YD13	CD44 antigen OS=Homo sapiens GN=CD44 PE=1 SV=2 - [H0YD1	1.161	1.638	0.977	1.122	1.087	1.198	0.976	1.082
Q96CP5	PMPCB protein (Fragment) OS=Homo sapiens GN=PMPCB PE=2	1.333	1.444	1.202	1.115	1.041	1.229	1.330	1.224
P17480	Nucleolar transcription factor 1 OS=Homo sapiens GN=UBTF PE=	1.349	1.721	1.487	1.687	1.184	1.215	1.425	1.964
P46821	Microtubule-associated protein 1B OS=Homo sapiens GN=MAP1B	1.387	1.032	1.032	0.746	1.100	1.035	1.010	0.924
J3QLB2	Zinc transporter ZIP11 (Fragment) OS=Homo sapiens GN=SLC39	1.696	1.557	1.638	1.524	1.167	1.329	1.702	1.317
Q9BRX8	Redox-regulatory protein FAM213A OS=Homo sapiens GN=FAM2	1.403	1.377	1.173	1.073	1.076	1.199	1.348	1.128
P42285	Superkiller viralicidic activity 2-like 2 OS=Homo sapiens GN=SKIV	1.550	1.605	1.298	0.966	1.563	1.348	1.633	1.129
Q14165	Malectin OS=Homo sapiens GN=MLEC PE=1 SV=1 - [MLEC_HUM	1.273	1.373	1.155	1.143	1.052	1.108	1.259	1.134
B2R6K4	cDNA, FLJ92996, highly similar to Homo sapiens guanine nucleoti	1.138	1.586	1.046	1.492	1.463	1.528	1.291	1.270
O75663	TIP41-like protein OS=Homo sapiens GN=TIPRL PE=1 SV=2 - [T	1.563	0.908	1.410	1.130	1.360	1.018	1.398	1.300
Q16740	ATP-dependent Clp protease proteolytic subunit, mitochondrial O	1.154	1.244	1.040	1.063	1.039	1.186	1.327	1.160
Q4G0J3	La-related protein 7 OS=Homo sapiens GN=LARP7 PE=1 SV=1 -	1.509	1.344	1.181	1.094	1.562	1.216	1.289	1.029
O15270	Serine palmitoyltransferase 2 OS=Homo sapiens GN=SPTLC2 PE=	1.966	1.703	1.635	1.381	1.315	1.383	2.023	1.397
Q8N1H4	cDNA FLJ40872 fis, clone TUTER2000283, highly similar to Homo	1.182	1.047	0.886	0.723	1.244	1.241	1.199	1.212
Q9BTU6	Phosphatidylinositol 4-kinase type 2-alpha OS=Homo sapiens GN	1.521	1.381	1.255	1.384	1.428	1.302	1.368	1.182
A8K885	cDNA FLJ77179, highly similar to Homo sapiens sorting nexin 6 (S	1.341	0.812	1.155	0.896	1.195	0.981	1.069	1.016
Q13907	Isopentenyl-diphosphate Delta-isomerase 1 OS=Homo sapiens GI	1.712	1.358	1.677	1.347	1.358	1.119	1.518	1.274
Q969Q0	60S ribosomal protein L36a-like OS=Homo sapiens GN=RPL36AL	1.167	0.997	1.048	0.758	1.227	1.114	1.027	0.889
O95478	Ribosome biogenesis protein NSA2 homolog OS=Homo sapiens G	1.774	2.175	1.667	1.042	2.152	1.501	2.233	1.173
P83881	60S ribosomal protein L36a OS=Homo sapiens GN=RPL36A PE=1	0.884	0.899	1.252	0.644	0.962	1.277	0.893	0.844
Q5JTV8	Torsin-1A-interacting protein 1 OS=Homo sapiens GN=TOR1AIP1	0.972	1.165	0.907	0.876	0.663	1.093	1.082	0.915
O95861	3'(2'),5'-bisphosphate nucleotidase 1 OS=Homo sapiens GN=BPN	1.112	0.995	1.335	1.070	1.012	1.001	1.201	1.323
Q9NPA8	Transcription and mRNA export factor ENY2 OS=Homo sapiens G	3.158	1.820	1.609	1.294	1.528	1.863	1.778	1.343
P15924	Desmoplakin OS=Homo sapiens GN=DSP PE=1 SV=3 - [DESP_HI	1.235	1.111	1.211	0.832	1.092	0.987	1.216	1.068
B5MCF9	Pescadillo homolog OS=Homo sapiens GN=PES1 PE=1 SV=1 - [B	1.768	1.868	1.595	1.309	1.673	1.530	1.728	1.243
P31937	3-hydroxyisobutyrate dehydrogenase, mitochondrial OS=Homo sa	1.013	1.094	0.917	0.919	0.854	1.056	0.967	0.891
C9JQV0	Uncharacterized protein C7orf50 (Fragment) OS=Homo sapiens G	1.543	2.058	1.673	1.401	1.548	1.551	1.541	1.423

Q7Z417	Nuclear fragile X mental retardation-interacting protein 2 OS=Hor	1.572	1.671	1.250	0.967	1.263	1.390	1.350	1.013
Q9BYN8	28S ribosomal protein S26, mitochondrial OS=Homo sapiens GN=	1.590	1.822	1.366	1.226	1.166	1.198	1.575	1.592
B4DH07	cDNA FLJ53321, highly similar to Homo sapiens pitrilysin metallop	1.271	1.311	1.105	1.125	1.034	1.122	1.173	1.179
D3DVL7	Transforming growth factor beta regulator 4, isoform CRA_b OS=	1.254	1.302	1.143	1.073	0.995	1.176	1.165	1.074
Q96DV4	39S ribosomal protein L38, mitochondrial OS=Homo sapiens GN=	1.325	1.425	1.110	1.096	0.981	1.208	1.229	1.155
Q99733	Nucleosome assembly protein 1-like 4 OS=Homo sapiens GN=NA	1.325	1.051	1.925	1.942	1.264	1.073	1.814	3.044
P35908	Keratin, type II cytoskeletal 2 epidermal OS=Homo sapiens GN=K	2.339	1.208	2.560	2.201	0.921	1.136	3.422	1.643
Q9NZW5	MAGUK p55 subfamily member 6 OS=Homo sapiens GN=MPP6 P	1.075	1.623	1.222	1.288	1.278	1.256	1.322	1.431
Q96KB5	Lymphokine-activated killer T-cell-originated protein kinase OS=H	1.243	0.841	1.169	0.915	1.065	1.018	1.108	1.180
P10155	60 kDa SS-A/Ro ribonucleoprotein OS=Homo sapiens GN=TROVE	1.151	0.938	0.948	0.756	1.210	1.019	0.976	0.888
P43246	DNA mismatch repair protein Msh2 OS=Homo sapiens GN=MSH2	1.275	1.120	1.248	1.077	1.352	1.160	1.174	1.151
P07311	Acylphosphatase-1 OS=Homo sapiens GN=ACYP1 PE=1 SV=2 - [	1.087	0.688	1.300	1.245	1.054	0.938	1.132	1.067
A8K9B9	cDNA FLJ77391, highly similar to Homo sapiens EH-domain conta	1.299	1.018	1.252	0.901	1.268	0.997	1.361	1.078
Q15645	Pachytene checkpoint protein 2 homolog OS=Homo sapiens GN=	1.494	0.962	1.319	1.044	1.279	1.088	1.267	1.195
A8K335	cDNA FLJ76254, highly similar to Homo sapiens gamma-glutamyl	1.287	1.259	1.635	1.875	1.349	1.183	1.732	1.800
A0MZ66	Shootin-1 OS=Homo sapiens GN=KIAA1598 PE=1 SV=4 - [SHOT	1.244	0.948	1.372	1.043	1.191	1.050	1.237	1.052
Q96SI9	Spermatid perinuclear RNA-binding protein OS=Homo sapiens GN	1.313	1.499	1.143	0.760	1.378	1.301	1.383	1.003
P51570	Galactokinase OS=Homo sapiens GN=GALK1 PE=1 SV=1 - [GALK	1.082	0.778	1.386	1.134	0.970	0.980	1.141	1.204
B3KWX6	cDNA FLJ44127 fis, clone THYMU2006420, highly similar to Morta	2.082	2.286	1.599	1.275	1.610	1.425	1.896	1.825
P48637	Glutathione synthetase OS=Homo sapiens GN=GSS PE=1 SV=1 -	1.145	0.780	1.185	0.959	1.123	0.928	1.104	1.000
P35659	Protein DEK OS=Homo sapiens GN=DEK PE=1 SV=1 - [DEK_HUN	1.878	2.027	1.774	1.419	1.427	1.235	1.643	1.646
Q9NRP4	Protein ACN9 homolog, mitochondrial OS=Homo sapiens GN=ACI	2.918	2.675	2.315	1.907	1.986	1.355	2.524	2.073
Q8N5A0	Eukaryotic translation initiation factor 5B OS=Homo sapiens GN=	1.599	1.643	1.615	1.210	1.300	1.143	1.332	1.249
Q15050	Ribosome biogenesis regulatory protein homolog OS=Homo sapie	1.693	1.884	1.422	1.358	1.684	1.436	1.532	1.502
F8W031	Uncharacterized protein (Fragment) OS=Homo sapiens PE=4 SV=	1.423	0.977	1.347	1.270	1.548	1.172	1.543	1.520
O75822	Eukaryotic translation initiation factor 3 subunit J OS=Homo sapie	1.814	1.322	1.664	1.173	1.722	1.300	1.506	1.325
Q9Y6D9	Mitotic spindle assembly checkpoint protein MAD1 OS=Homo sap	1.234	1.424	1.405	1.408	1.330	1.357	1.520	1.671
B4DXW4	Caseinolytic peptidase B protein homolog OS=Homo sapiens GN=	1.884	2.163	1.977	2.012	1.729	1.593	2.202	2.246
Q9Y570	Protein phosphatase methylesterase 1 OS=Homo sapiens GN=PP	1.336	1.001	1.376	1.056	1.418	1.053	1.171	1.030
K7EQG9	Tyrosine-protein phosphatase non-receptor type OS=Homo sapie	1.433	1.389	1.108	0.927	1.184	1.187	1.385	0.975
Q96F54	RELA protein OS=Homo sapiens GN=RELA PE=2 SV=1 - [Q96F54	0.975	0.921	0.925	0.715	1.504	1.190	1.476	0.739
Q9NYJ1	Cytochrome c oxidase assembly factor 4 homolog, mitochondrial	2.477	2.869	3.736	3.526	2.456	2.027	3.298	3.778
C9J2Y9	DNA-directed RNA polymerase OS=Homo sapiens GN=POLR2B PI	1.565	1.626	1.418	1.231	1.508	1.270	1.495	1.150
Q14008	Cytoskeleton-associated protein 5 OS=Homo sapiens GN=CKAP5	1.391	1.301	1.367	0.926	1.071	1.091	1.347	1.128
B4DJV2	Citrate synthase OS=Homo sapiens GN=CS PE=1 SV=1 - [B4DJV	1.465	1.357	1.210	1.266	1.025	1.191	1.387	1.265
B9A044	Eukaryotic translation initiation factor 4E type 2 OS=Homo sapier	1.628	1.539	1.675	1.257	1.547	1.283	1.509	1.288
P61201	COP9 signalosome complex subunit 2 OS=Homo sapiens GN=CO	1.306	1.065	1.155	0.988	1.275	1.240	1.063	1.086
Q13356	Peptidyl-prolyl cis-trans isomerase-like 2 OS=Homo sapiens GN=I	1.326	1.814	1.442	0.975	1.428	1.453	1.445	1.046
B4E2I4	cDNA FLJ58227, highly similar to Glutamate--cysteine ligase catal	1.686	0.975	1.340	1.112	1.338	1.096	1.291	1.063
Q9H936	Mitochondrial glutamate carrier 1 OS=Homo sapiens GN=SLC25A	1.243	1.568	1.306	1.481	0.941	1.269	1.453	1.499

O15144	Actin-related protein 2/3 complex subunit 2 OS=Homo sapiens GI	1.618	1.586	1.591	1.258	1.489	1.115	1.469	1.324
K7ES61	39S ribosomal protein L4, mitochondrial (Fragment) OS=Homo sa	1.163	1.335	1.012	1.112	0.851	1.092	1.166	1.139
B7ZLQ5	SMARCA1 protein OS=Homo sapiens GN=SMARCA1 PE=2 SV=1 -	1.256	1.329	1.105	0.827	1.149	1.135	1.158	1.035
X6RM59	Cytosolic 5'-nucleotidase 3A OS=Homo sapiens GN=NT5C3A PE=	1.279	1.873	1.455	1.312	1.242	1.592	1.464	1.519
Q14651	Plastin-1 OS=Homo sapiens GN=PLS1 PE=1 SV=2 - [PLSI_HUMA	1.340	1.027	1.183	1.001	1.202	1.054	1.251	1.099
Q7L2E3	Putative ATP-dependent RNA helicase DHX30 OS=Homo sapiens	1.421	1.554	1.254	1.026	1.056	1.187	1.405	1.198
B5BUI8	Dual specificity phosphatase 3 (Fragment) OS=Homo sapiens GN	1.798	1.018	1.478	1.180	1.697	1.042	1.464	1.319
P78345	Ribonuclease P protein subunit p38 OS=Homo sapiens GN=RPP3	1.342	1.328	1.337	1.030	1.199	1.375	1.268	1.228
P62330	ADP-ribosylation factor 6 OS=Homo sapiens GN=ARF6 PE=1 SV=	1.694	1.449	1.135	1.039	1.497	1.025	1.308	1.411
O75436	Vacuolar protein sorting-associated protein 26A OS=Homo sapien	1.480	1.056	1.311	0.924	1.437	1.105	1.219	1.064
B2R665	cDNA, FLJ92810, highly similar to Homo sapiens protein phospha	1.045	0.909	0.985	0.738	1.072	1.078	0.917	0.846
Q9UJA5	tRNA (adenine(58)-N(1))-methyltransferase non-catalytic subunit	1.356	1.258	1.013	0.822	1.337	1.230	1.050	0.823
P25788	Proteasome subunit alpha type-3 OS=Homo sapiens GN=PSMA3	1.097	0.908	1.011	0.761	1.252	1.062	0.957	0.827
Q567R0	UQCRH protein OS=Homo sapiens GN=UQCRH PE=2 SV=1 - [Q5	1.375	1.847	1.283	1.501	1.189	1.244	1.609	1.579
Q5T179	Cyclin-dependent kinases regulatory subunit OS=Homo sapiens G	1.103	0.804	1.090	0.789	1.212	1.163	1.182	1.052
Q1W6G4	LUC7-like (S. cerevisiae) OS=Homo sapiens GN=LUC7L PE=4 SV=	1.505	1.441	1.441	1.115	1.385	1.333	1.446	1.221
Q9NVX2	Notchless protein homolog 1 OS=Homo sapiens GN=NLE1 PE=1	1.533	1.471	1.394	1.028	1.598	1.418	1.612	1.164
Q9H0B6	Kinesin light chain 2 OS=Homo sapiens GN=KLC2 PE=1 SV=1 - [	1.393	1.014	1.342	0.975	1.366	1.183	1.524	1.124
A5YM53	ITGAV protein OS=Homo sapiens GN=ITGAV PE=2 SV=1 - [A5YN	1.120	1.325	0.956	1.196	1.013	1.009	1.108	1.086
A8K2I7	cDNA FLJ76072, highly similar to Homo sapiens GIPC PDZ domai	1.683	1.178	1.160	0.959	1.334	1.073	1.279	1.118
Q9BPW8	Protein NipSnap homolog 1 OS=Homo sapiens GN=NIPSNAP1 PE	1.235	1.215	1.030	0.996	1.096	1.141	1.237	1.121
Q14258	E3 ubiquitin/ISG15 ligase TRIM25 OS=Homo sapiens GN=TRIM2	1.533	1.219	1.345	0.989	1.284	1.174	1.313	1.144
Q53GE7	Tetratricopeptide repeat domain 1 variant (Fragment) OS=Homo	1.371	1.042	1.405	1.278	1.184	1.175	1.322	1.398
Q7Z5G4	Golgin subfamily A member 7 OS=Homo sapiens GN=GOLGA7 PE	0.952	1.582	0.992	0.980	1.102	1.072	1.130	1.049
P27824	Calnexin OS=Homo sapiens GN=CANX PE=1 SV=2 - [CALX_HUM	1.926	1.599	1.733	1.517	1.341	1.309	1.742	1.603
B3KQF0	cDNA FLJ90354 fis, clone NT2RP2003390, highly similar to Transl	1.597	1.812	2.152	1.792	1.182	1.365	1.327	1.593
Q13200	26S proteasome non-ATPase regulatory subunit 2 OS=Homo sapi	1.323	1.040	1.096	0.825	1.367	1.052	1.095	0.873
A8JZZ8	cDNA FLJ77826, highly similar to Homo sapiens BCS1-like (yeast)	1.245	1.421	1.244	1.291	1.060	1.119	1.342	1.418
Q13428	Treacle protein OS=Homo sapiens GN=TCOF1 PE=1 SV=3 - [TCC	1.244	1.647	1.100	0.981	1.067	1.298	1.125	1.170
O14828	Secretory carrier-associated membrane protein 3 OS=Homo sapie	1.264	1.519	1.290	1.150	1.192	1.292	1.504	1.373
Q15819	Ubiquitin-conjugating enzyme E2 variant 2 OS=Homo sapiens GN	1.224	0.877	1.289	1.067	1.094	1.069	1.270	1.050
Q8TED0	U3 small nucleolar RNA-associated protein 15 homolog OS=Homo	1.736	1.974	1.688	1.595	1.694	1.440	1.850	1.647
A8K097	cDNA FLJ78373, highly similar to Homo sapiens PCI domain conta	2.762	2.340	2.004	1.747	2.324	1.451	2.441	2.141
Q12888	Tumor suppressor p53-binding protein 1 OS=Homo sapiens GN=T	1.429	1.368	1.019	0.768	1.432	1.217	1.239	0.778
B2RBR9	cDNA, FLJ95650, highly similar to Homo sapiens karyopherin (im	1.574	1.189	1.524	1.328	1.360	1.119	1.439	1.231
B2RBM8	cDNA, FLJ95596, highly similar to Homo sapiens activity-depende	1.581	1.616	1.202	0.957	1.191	1.327	1.322	1.357
Q86YP4	Transcriptional repressor p66-alpha OS=Homo sapiens GN=GATA	1.097	1.382	0.933	0.845	0.971	1.295	1.266	1.053
Q9UBB4	Ataxin-10 OS=Homo sapiens GN=ATXN10 PE=1 SV=1 - [ATX10_	1.308	1.039	1.071	0.794	1.256	1.004	1.203	1.011
B4DQM6	cDNA FLJ55563, highly similar to Homo sapiens G1 to S phase tra	1.261	0.954	1.299	0.980	1.209	1.030	1.264	0.991
Q92769	Histone deacetylase 2 OS=Homo sapiens GN=HDAC2 PE=1 SV=2	1.743	1.274	1.385	1.118	1.887	1.365	1.463	1.227

Q7L2H7	Eukaryotic translation initiation factor 3 subunit M OS=Homo sapi	2.072	1.927	1.920	1.832	1.910	1.437	1.767	1.592
B4DJQ5	cDNA FLJ59211, highly similar to Glucosidase 2 subunit beta OS=	1.551	1.095	1.406	1.465	1.610	1.214	1.528	1.508
Q08945	FACT complex subunit SSRP1 OS=Homo sapiens GN=SSRP1 PE=	1.498	1.944	1.826	1.869	1.293	1.352	1.826	2.040
Q92665	28S ribosomal protein S31, mitochondrial OS=Homo sapiens GN=	1.297	1.374	1.040	1.157	0.959	0.991	1.137	1.270
P63272	Transcription elongation factor SPT4 OS=Homo sapiens GN=SUPT	3.021	2.627	2.511	1.948	2.486	1.786	2.265	2.232
B4DP27	cDNA FLJ52153, highly similar to Transmembrane emp24 domain	1.413	1.497	1.275	1.225	1.059	1.221	1.247	1.107
P55036	26S proteasome non-ATPase regulatory subunit 4 OS=Homo sapi	1.324	1.008	1.004	0.711	1.287	1.113	1.063	0.834
B2R6Z3	cDNA, FLJ93192, highly similar to Homo sapiens serine/threonine	1.064	1.178	1.182	0.838	1.035	1.408	1.420	1.322
O75394	39S ribosomal protein L33, mitochondrial OS=Homo sapiens GN=	1.287	1.552	1.019	0.950	1.024	1.023	1.171	1.133
Q9H7E9	UPF0488 protein C8orf33 OS=Homo sapiens GN=C8orf33 PE=1 S	1.608	1.475	1.641	1.110	1.317	1.332	1.637	1.320
P84103	Serine/arginine-rich splicing factor 3 OS=Homo sapiens GN=SRSI	2.611	2.203	2.367	2.231	2.271	1.144	2.241	2.243
Q9Y3D7	Mitochondrial import inner membrane translocase subunit TIM16	1.528	1.656	1.350	1.151	1.158	1.131	1.442	1.424
E1NZA1	Peroxisome proliferator activated receptor interacting complex pr	1.531	0.995	1.391	1.007	1.306	1.007	1.362	1.045
B2R9I9	cDNA, FLJ94417, highly similar to Homo sapiens WD repeat doma	1.482	1.628	1.289	1.073	1.589	1.435	1.529	1.360
O60830	Mitochondrial import inner membrane translocase subunit Tim17-	1.889	1.966	1.920	1.944	1.495	1.287	1.982	1.998
Q86VY4	Testis-specific Y-encoded-like protein 5 OS=Homo sapiens GN=TS	1.730	2.211	1.777	1.665	1.715	1.530	2.300	2.365
Q9BXP5	Serrate RNA effector molecule homolog OS=Homo sapiens GN=S	1.792	1.919	1.531	1.477	1.955	1.416	1.959	1.794
O94906	Pre-mRNA-processing factor 6 OS=Homo sapiens GN=PRPF6 PE=	1.284	1.475	1.140	0.881	1.219	1.290	1.332	1.065
Q92804	TATA-binding protein-associated factor 2N OS=Homo sapiens GN	1.207	1.236	0.795	0.698	1.451	1.222	1.206	0.969
Q53S33	BolA-like protein 3 OS=Homo sapiens GN=BOLA3 PE=1 SV=1 - [I	1.196	1.339	0.822	1.123	1.144	1.319	1.294	1.253
Q96AY3	Peptidyl-prolyl cis-trans isomerase FKBP10 OS=Homo sapiens GN	1.247	0.795	1.079	1.292	1.296	1.139	1.274	1.389
Q9NZL9	Methionine adenosyltransferase 2 subunit beta OS=Homo sapiens	1.102	0.860	1.061	0.837	1.073	1.007	0.882	0.819
Q96BS2	Calcineurin B homologous protein 3 OS=Homo sapiens GN=TESC	1.158	1.157	1.283	1.241	1.094	1.121	1.247	1.298
K7ESP4	Dephospho-CoA kinase domain-containing protein (Fragment) OS	1.678	1.643	1.440	1.521	1.557	1.281	1.884	1.801
Q9Y6C9	Mitochondrial carrier homolog 2 OS=Homo sapiens GN=MTCH2 P	1.253	1.688	1.145	1.248	0.891	1.136	1.240	1.209
Q9UBS4	DnaJ homolog subfamily B member 11 OS=Homo sapiens GN=DI	1.921	1.365	1.514	1.516	1.502	1.299	1.704	1.569
O60493	Sorting nexin-3 OS=Homo sapiens GN=SNX3 PE=1 SV=3 - [SNX3	1.363	0.784	1.376	0.936	1.336	1.218	1.701	1.432
B2RE68	cDNA, FLJ94243 OS=Homo sapiens PE=2 SV=1 - [B2RE68_HUM/	1.718	1.653	1.464	1.454	1.566	1.408	1.839	1.610
Q6P1M0	Long-chain fatty acid transport protein 4 OS=Homo sapiens GN=S	1.321	1.791	1.321	1.393	1.239	1.427	1.448	1.326
A0A024RBE8	Solute carrier family 25 (Mitochondrial carrier phosphate carrier),	1.272	1.413	1.053	1.196	1.081	1.221	1.258	1.264
B4DHN0	Mitogen-activated protein kinase OS=Homo sapiens PE=2 SV=1 -	1.486	0.981	1.396	1.060	1.296	1.340	1.317	1.182
Q12996	Cleavage stimulation factor subunit 3 OS=Homo sapiens GN=CST	1.245	1.404	1.042	0.877	1.218	1.312	1.351	1.075
Q9BRA2	Thioredoxin domain-containing protein 17 OS=Homo sapiens GN=	0.985	0.672	1.052	0.985	0.869	0.889	0.939	0.911
Q9BVG4	Protein PBDC1 OS=Homo sapiens GN=PBDC1 PE=1 SV=1 - [PBD	1.219	1.212	1.230	1.093	1.577	1.455	1.031	1.136
Q14126	Desmoglein-2 OS=Homo sapiens GN=DSG2 PE=1 SV=2 - [DSG2_	0.739	1.598	0.984	1.188	0.681	1.061	0.994	1.012
A0A024RBR1	Restin (Reed-Steinberg cell-expressed intermediate filament-asso	1.530	0.939	1.165	0.951	1.301	1.078	1.206	0.976
F5H365	Protein transport protein Sec23A OS=Homo sapiens GN=SEC23A	1.402	1.148	2.017	1.194	1.608	1.191	1.666	0.986
O15143	Actin-related protein 2/3 complex subunit 1B OS=Homo sapiens G	0.839	0.964	0.860	0.799	1.137	1.003	0.974	0.686
O60220	Mitochondrial import inner membrane translocase subunit Tim8 A	3.050	2.718	3.252	2.649	2.188	1.604	2.254	3.003
O43776	Asparagine--tRNA ligase, cytoplasmic OS=Homo sapiens GN=NAI	1.665	1.268	1.541	1.061	1.865	1.035	1.666	1.188

Q9BTZ2	Dehydrogenase/reductase SDR family member 4 OS=Homo sapie	1.474	1.367	1.240	1.326	1.279	1.203	1.430	1.416
B3KMA8	cDNA FLJ10612 fis, clone NT2RP2005358, highly similar to Methy	1.061	1.128	0.820	0.536	1.040	1.306	1.074	0.919
B4DMT5	Eukaryotic translation initiation factor 3 subunit F OS=Homo sapi	1.593	1.504	1.581	1.276	1.487	1.307	1.437	1.358
Q9BRT2	Ubiquinol-cytochrome-c reductase complex assembly factor 2 OS	1.828	1.835	1.903	1.951	1.469	1.294	2.331	1.525
A8K588	cDNA FLJ76823, highly similar to Homo sapiens splicing factor, ar	1.410	1.478	1.279	1.127	1.389	1.506	1.548	1.418
O00487	26S proteasome non-ATPase regulatory subunit 14 OS=Homo sap	1.342	1.042	1.031	0.795	1.162	1.160	1.011	1.021
Q9NQP4	Prefoldin subunit 4 OS=Homo sapiens GN=PFDN4 PE=1 SV=1 - [	1.029	0.801	1.039	0.825	1.080	0.959	1.007	0.953
B2R860	cDNA, FLJ93752, highly similar to Homo sapiens phosphoribosyl p	1.622	1.168	1.609	1.242	1.472	0.999	1.536	1.264
O43148	mRNA cap guanine-N7 methyltransferase OS=Homo sapiens GN=	1.517	1.582	1.121	0.839	1.490	1.322	1.139	0.829
H0YGR4	Oligoribonuclease, mitochondrial (Fragment) OS=Homo sapiens C	1.274	1.033	1.361	1.190	1.026	1.045	1.065	1.144
P20674	Cytochrome c oxidase subunit 5A, mitochondrial OS=Homo sapie	1.221	1.179	1.045	1.016	0.859	1.074	1.154	1.154
Q92896	Golgi apparatus protein 1 OS=Homo sapiens GN=GLG1 PE=1 SV=	1.337	1.500	1.271	1.271	1.188	1.242	1.501	1.135
F8VPI7	Double-stranded RNA-binding protein Staufen homolog 2 OS=Hor	1.162	1.150	1.012	0.593	1.182	1.156	1.016	0.789
B2R6J3	cDNA, FLJ92974, highly similar to Homo sapiens methylmalonic a	1.284	1.356	1.192	1.082	0.974	1.146	1.354	1.274
O14745	Na(+)/H(+) exchange regulatory cofactor NHE-RF1 OS=Homo sa	1.106	0.846	1.063	0.992	0.998	1.004	1.056	0.954
Q15257	Serine/threonine-protein phosphatase 2A activator OS=Homo sap	1.106	0.771	1.205	1.124	0.972	0.890	1.070	1.048
P82921	28S ribosomal protein S21, mitochondrial OS=Homo sapiens GN=	1.217	1.547	1.139	1.167	1.031	1.162	1.388	1.284
P12081	Histidine--tRNA ligase, cytoplasmic OS=Homo sapiens GN=HARS	1.432	0.950	1.517	1.179	1.367	1.049	1.358	1.254
Q53H12	Acylglycerol kinase, mitochondrial OS=Homo sapiens GN=AGK PE	1.651	1.806	1.547	1.562	1.198	1.254	1.764	1.758
P17812	CTP synthase 1 OS=Homo sapiens GN=CTPS1 PE=1 SV=2 - [PYI	1.465	0.971	1.433	1.095	1.403	1.111	1.325	1.292
B4DV94	cDNA FLJ58285, highly similar to Homo sapiens pre-B-cell leukem	1.168	1.170	1.027	0.872	1.072	1.116	1.012	0.936
Q9NPJ3	Acyl-coenzyme A thioesterase 13 OS=Homo sapiens GN=ACOT13	1.086	1.093	0.989	0.897	0.865	1.038	0.988	0.926
Q7Z478	ATP-dependent RNA helicase DHX29 OS=Homo sapiens GN=DHX	1.427	1.597	1.349	0.922	1.117	1.098	1.236	1.048
E7ETY2	Treacle protein OS=Homo sapiens GN=TCOF1 PE=1 SV=1 - [E7E	1.265	1.352	1.319	1.125	0.985	1.329	1.271	1.510
Q9C0C2	182 kDa tankyrase-1-binding protein OS=Homo sapiens GN=TNK	1.330	1.097	0.948	0.623	1.143	0.969	0.901	0.740
B4DG22	cDNA FLJ56618, highly similar to Ribosomal protein S6 kinase alp	1.110	0.835	0.901	0.760	0.963	0.915	0.852	0.746
W6MEN3	Beta-1,4-galactosyltransferase 1 OS=Homo sapiens GN=B4GALT1	1.571	1.542	1.141	0.769	1.566	1.273	1.396	0.781
O14949	Cytochrome b-c1 complex subunit 8 OS=Homo sapiens GN=UQC	1.253	1.420	1.262	1.216	0.844	1.040	1.208	1.260
A0A024R5K3	NADH dehydrogenase (Ubiquinone) Fe-S protein 8, 23kDa (NADH	1.269	1.378	1.303	1.271	1.151	1.201	1.430	1.519
O76031	ATP-dependent Clp protease ATP-binding subunit clpX-like, mitocl	1.584	1.409	1.171	1.083	1.277	1.255	1.420	1.040
P23921	Ribonucleoside-diphosphate reductase large subunit OS=Homo sa	1.355	0.976	1.449	1.098	1.139	1.084	1.450	1.377
I1VE18	SEC22 vesicle trafficking protein B (Fragment) OS=Homo sapiens	1.788	1.994	1.811	1.672	1.146	1.399	1.516	1.564
Q9Y2Z4	Tyrosine--tRNA ligase, mitochondrial OS=Homo sapiens GN=YARS	1.311	1.348	1.193	1.167	1.153	1.214	1.314	1.364
D3DTX6	Neurabin-2 OS=Homo sapiens GN=PPP1R9B PE=4 SV=1 - [D3DT	1.440	1.465	1.269	1.091	1.211	1.450	1.451	1.384
Q9NPA3	Mid1-interacting protein 1 OS=Homo sapiens GN=MID1IP1 PE=1	0.889	0.488	0.363	0.224	0.995	0.730	0.460	0.335
Q5J8M3	ER membrane protein complex subunit 4 OS=Homo sapiens GN=	1.166	1.175	0.978	0.976	0.950	1.178	1.069	0.997
Q9BS26	Endoplasmic reticulum resident protein 44 OS=Homo sapiens GN	1.829	1.235	1.390	1.414	1.320	1.199	1.498	1.437
B3KPU0	PDZ and LIM domain protein 2 OS=Homo sapiens GN=PDLIM2 PI	1.248	0.823	1.013	0.778	0.931	0.983	0.816	0.944
B4DUI8	cDNA FLJ52761, highly similar to Actin, aortic smooth muscle OS	1.264	1.265	1.032	0.885	1.145	1.249	1.111	1.110
A8K3X3	cDNA FLJ77685 OS=Homo sapiens PE=2 SV=1 - [A8K3X3_HUMA	1.948	2.412	2.146	1.742	2.470	1.670	2.221	1.462

Q9H2W6	39S ribosomal protein L46, mitochondrial OS=Homo sapiens GN=	1.216	1.288	1.062	1.101	1.013	1.248	1.280	1.182
E7D7X9	Pyrroline-5-carboxylate reductase OS=Homo sapiens PE=2 SV=1	1.344	1.356	1.194	1.199	1.250	1.028	1.389	1.199
E5RGS4	Prefoldin subunit 1 OS=Homo sapiens GN=PFDN1 PE=1 SV=1 - [	1.251	0.902	1.264	0.970	1.140	1.028	1.145	1.165
Q9Y4W2	Ribosomal biogenesis protein LAS1L OS=Homo sapiens GN=LAS1	1.460	1.605	1.459	1.025	1.520	1.503	1.511	1.121
Q99598	Translin-associated protein X OS=Homo sapiens GN=TSNAX PE=	1.833	1.547	1.746	1.412	1.821	1.257	1.396	1.570
F8W038	Chromatin complexes subunit BAP18 OS=Homo sapiens GN=C17	1.225	0.836	0.943	0.658	1.284	1.038	0.964	0.740
P51649	Succinate-semialdehyde dehydrogenase, mitochondrial OS=Homo	1.260	1.429	1.414	1.541	1.017	1.183	1.564	1.433
O15320	cTAGE family member 5 OS=Homo sapiens GN=CTAGE5 PE=1 SV	1.452	1.524	1.802	1.770	1.222	1.364	1.973	1.554
Q9H8H0	Nucleolar protein 11 OS=Homo sapiens GN=NOL11 PE=1 SV=1 -	1.923	2.082	1.779	1.824	1.840	1.698	2.193	2.324
A8K7V1	cDNA FLJ78053, highly similar to Homo sapiens splicing factor 3a	1.604	1.646	1.402	1.109	1.566	1.449	1.540	1.285
O43175	D-3-phosphoglycerate dehydrogenase OS=Homo sapiens GN=PH	1.176	0.678	1.101	0.834	1.049	0.860	1.033	1.070
M0R176	39S ribosomal protein L51, mitochondrial OS=Homo sapiens GN=	1.342	1.688	1.107	1.220	1.294	1.249	1.647	1.433
B2RDU2	cDNA, FLJ96768, Homo sapiens non-SMC (structural maintenance	2.081	1.835	1.622	1.440	2.045	1.314	1.960	1.848
Q05DA4	P4HA2 protein OS=Homo sapiens GN=P4HA2 PE=2 SV=1 - [Q05	1.197	0.825	0.912	0.800	1.286	1.019	1.213	0.980
Q6PJT7	Zinc finger CCCH domain-containing protein 14 OS=Homo sapien	1.583	1.850	1.262	1.104	1.323	1.546	1.805	1.773
Q09161	Nuclear cap-binding protein subunit 1 OS=Homo sapiens GN=NC	2.967	2.848	3.071	2.694	2.550	1.626	2.695	2.807
K7EJR3	26S proteasome non-ATPase regulatory subunit 8 (Fragment) OS	1.444	1.144	1.213	0.911	1.450	1.171	1.214	1.057
Q5QNY5	Peroxisomal biogenesis factor 19 (Fragment) OS=Homo sapiens (	1.727	1.288	1.818	1.629	1.726	1.141	1.610	1.512
Q9H6T3	RNA polymerase II-associated protein 3 OS=Homo sapiens GN=R	1.561	1.110	1.220	0.974	1.246	1.035	1.200	1.087
P49321	Nuclear autoantigenic sperm protein OS=Homo sapiens GN=NAS	1.284	1.049	0.944	0.800	1.203	1.081	1.016	0.925
P49755	Transmembrane emp24 domain-containing protein 10 OS=Homo	1.056	1.163	1.100	1.092	1.070	1.191	1.206	0.912
B2R4U3	cDNA, FLJ92217, highly similar to Homo sapiens ubiquitin-conjug	1.571	0.948	0.785	0.647	1.319	1.338	1.137	0.780
Q6IQ30	PABPC4 protein OS=Homo sapiens GN=PABPC4 PE=2 SV=1 - [Q	1.461	1.397	1.366	0.980	1.313	1.174	1.295	1.280
P31751	RAC-beta serine/threonine-protein kinase OS=Homo sapiens GN=	1.287	0.979	1.185	0.822	1.025	1.014	1.000	0.908
Q9UL40	Zinc finger protein 346 OS=Homo sapiens GN=ZNF346 PE=1 SV=	2.401	2.407	2.058	1.678	1.942	1.533	1.616	1.355
O75323	Protein NipSnap homolog 2 OS=Homo sapiens GN=GBAS PE=1 S	1.253	1.347	1.115	1.154	1.018	1.099	1.262	1.165
Q9BPW0	Serine/threonine-protein phosphatase (Fragment) OS=Homo sapi	1.644	1.030	1.649	1.228	1.479	1.159	1.480	1.406
Q9Y3C7	Mediator of RNA polymerase II transcription subunit 31 OS=Homo	2.089	1.721	1.433	1.152	1.921	1.437	1.232	1.299
Q96CM8	Acyl-CoA synthetase family member 2, mitochondrial OS=Homo s	1.188	1.437	1.360	1.421	0.968	1.145	1.377	1.353
Q96C57	Uncharacterized protein C12orf43 OS=Homo sapiens GN=C12orf4	1.057	1.249	1.051	1.300	1.152	1.390	1.284	1.379
P48729	Casein kinase I isoform alpha OS=Homo sapiens GN=CSNK1A1 P	1.504	1.289	0.980	0.905	1.244	1.180	1.369	1.036
O00743	Serine/threonine-protein phosphatase 6 catalytic subunit OS=Hor	1.832	1.133	1.443	1.187	1.379	1.115	1.546	1.242
P49841	Glycogen synthase kinase-3 beta OS=Homo sapiens GN=GSK3B I	1.387	0.924	1.076	0.774	1.193	1.050	1.096	0.902
P49642	DNA primase small subunit OS=Homo sapiens GN=PRIM1 PE=1 S	1.414	1.204	1.127	0.724	1.271	1.139	1.247	0.989
A0A087WXI1	3-hydroxyisobutyryl-CoA hydrolase, mitochondrial OS=Homo sapi	1.762	1.611	1.360	1.350	1.282	1.203	1.556	1.627
P21980	Protein-glutamine gamma-glutamyltransferase 2 OS=Homo sapie	1.444	0.780	1.401	1.232	1.120	0.892	1.116	1.163
B4DFL1	Dihydrolipoyl dehydrogenase OS=Homo sapiens PE=2 SV=1 - [B4	1.481	1.321	1.139	1.221	1.059	1.237	1.290	1.287
P41252	Isoleucine--tRNA ligase, cytoplasmic OS=Homo sapiens GN=IARS	1.428	1.240	1.245	0.996	1.176	1.029	1.224	1.053
Q9NXV6	CDKN2A-interacting protein OS=Homo sapiens GN=CDKN2AIP PE	1.128	1.262	0.809	0.574	1.225	1.347	0.892	0.814
Q14691	DNA replication complex GINS protein PSF1 OS=Homo sapiens G	1.083	0.867	0.817	0.666	1.123	1.235	0.785	0.722

Q5T6V5	UPF0553 protein C9orf64 OS=Homo sapiens GN=C9orf64 PE=1 S	1.472	1.017	1.485	1.346	1.254	1.193	1.240	1.315
P30085	UMP-CMP kinase OS=Homo sapiens GN=CMPK1 PE=1 SV=3 - [K	1.125	0.835	1.260	1.054	0.982	0.953	1.016	1.095
Q8TDD1	ATP-dependent RNA helicase DDX54 OS=Homo sapiens GN=DDX	1.631	1.613	1.290	0.958	1.613	1.305	1.624	1.299
P15428	15-hydroxyprostaglandin dehydrogenase [NAD(+)] OS=Homo sa	1.115	0.512	0.461	0.227	0.900	0.633	0.467	0.351
I3L0K1	Golgi SNAP receptor complex member 2 (Fragment) OS=Homo s	2.109	2.088	1.840	1.577	1.863	1.667	1.678	1.691
B3KUB6	cDNA FLJ39529 fis, clone PUAEN2004067, highly similar to Band	1.169	0.740	0.990	0.832	1.557	0.982	0.826	0.964
E9PLI6	Probable RNA-binding protein EIF1AD (Fragment) OS=Homo sapi	1.110	1.245	1.138	0.872	1.050	1.349	1.204	1.377
Q9NY61	Protein AATF OS=Homo sapiens GN=AATF PE=1 SV=1 - [AATF_H	1.539	1.633	1.391	1.315	1.265	1.452	1.520	1.319
B4DWC4	cDNA FLJ61180, highly similar to Chloride intracellular channel pr	1.307	1.048	1.326	0.965	1.080	0.953	1.048	1.089
P60510	Serine/threonine-protein phosphatase 4 catalytic subunit OS=Hor	1.540	1.389	1.225	0.827	1.730	1.276	1.322	1.085
H3BN98	Uncharacterized protein (Fragment) OS=Homo sapiens PE=4 SV=	1.835	1.888	1.135	1.488	1.672	1.425	1.665	1.275
Q9GZL7	Ribosome biogenesis protein WDR12 OS=Homo sapiens GN=WD	1.667	1.760	1.614	1.671	1.741	1.383	1.789	1.573
Q9UNS2	COP9 signalosome complex subunit 3 OS=Homo sapiens GN=CO	1.550	1.244	1.242	0.912	1.470	1.188	1.400	1.281
A0A087WZX2	NADH dehydrogenase [ubiquinone] 1 beta subcomplex subunit 6	1.305	1.575	1.422	1.421	1.262	1.307	1.367	1.095
Q9BW83	Intraflagellar transport protein 27 homolog OS=Homo sapiens GN	1.364	1.047	1.462	1.350	1.159	0.996	1.262	1.269
A0A087WUM0	Protein SYNJ2BP-COX16 (Fragment) OS=Homo sapiens GN=SYN	1.280	1.579	1.513	1.420	0.970	1.159	1.644	1.606
Q2KS26	Large T antigen GN=E1B_largeT PE=4 SV=1 - [Q2KS26_ADE05]	1.741	3.864	5.324	6.566	1.479	0.898	1.602	1.403
Q12849	G-rich sequence factor 1 OS=Homo sapiens GN=GRSF1 PE=1 SV	1.312	1.401	1.128	1.020	1.079	1.269	1.362	1.163
Q9UIA9	Exportin-7 OS=Homo sapiens GN=XPO7 PE=1 SV=3 - [XPO7_HU	1.682	1.087	1.896	1.304	1.428	1.143	1.489	1.480
P55789	FAD-linked sulfhydryl oxidase ALR OS=Homo sapiens GN=GFER F	1.215	1.198	1.158	1.083	1.352	1.318	1.358	1.646
Q6P1X6	UPF0598 protein C8orf82 OS=Homo sapiens GN=C8orf82 PE=1 S	1.444	1.411	0.886	1.029	0.891	1.220	0.936	0.950
P50579	Methionine aminopeptidase 2 OS=Homo sapiens GN=METAP2 PE	1.645	1.559	1.566	1.221	1.247	1.201	1.403	1.289
Q5JPE4	Putative uncharacterized protein DKFZp667O202 OS=Homo sapie	1.968	1.301	1.945	1.489	1.926	1.064	1.808	1.657
O76003	Glutaredoxin-3 OS=Homo sapiens GN=GLRX3 PE=1 SV=2 - [GLR	1.198	0.891	1.132	1.000	1.026	1.009	1.067	0.978
Q9BUN6	MRPS30 protein (Fragment) OS=Homo sapiens GN=MRPS30 PE=	1.204	1.181	1.062	0.893	0.970	1.210	1.034	1.027
O95571	Persulfide dioxygenase ETHE1, mitochondrial OS=Homo sapiens	1.666	1.562	2.037	1.929	1.401	1.383	1.374	1.716
I3L4Q4	PHD finger protein 23 (Fragment) OS=Homo sapiens GN=PHF23	0.934	0.892	0.662	0.337	1.070	0.985	0.780	0.539
D3DXC9	Serine hydroxymethyltransferase OS=Homo sapiens GN=SHMT1	1.668	1.059	1.697	1.098	1.480	1.177	1.597	1.845
Q8WXX5	DnaJ homolog subfamily C member 9 OS=Homo sapiens GN=DN	1.226	1.068	1.144	0.963	1.154	1.083	1.257	1.113
Q969S3	Zinc finger protein 622 OS=Homo sapiens GN=ZNF622 PE=1 SV=	1.274	1.413	1.425	0.889	1.464	1.267	1.422	1.096
P46013	Antigen KI-67 OS=Homo sapiens GN=MKI67 PE=1 SV=2 - [KI67_	1.509	2.048	1.341	1.113	1.267	1.153	1.337	1.151
P49257	Protein ERGIC-53 OS=Homo sapiens GN=LMAN1 PE=1 SV=2 - [L	1.250	1.342	1.496	1.282	1.042	1.185	1.299	1.254
J3KMX2	SWI/SNF-related matrix-associated actin-dependent regulator of	1.181	1.260	0.817	0.541	1.125	1.211	1.036	0.660
O00154	Cytosolic acyl coenzyme A thioester hydrolase OS=Homo sapiens	1.519	1.137	1.478	1.121	1.270	1.021	1.295	1.126
A8MWD9	Putative small nuclear ribonucleoprotein G-like protein 15 OS=Ho	2.115	1.637	1.224	1.001	1.033	1.046	1.158	1.096
Q9P2K5	Myelin expression factor 2 OS=Homo sapiens GN=MYEF2 PE=1 S	2.142	1.089	1.719	1.429	2.604	1.612	3.030	1.003
Q53GJ1	DEAD (Asp-Glu-Ala-Asp) box polypeptide 47 isoform 1 variant (Fr	1.777	1.819	1.398	1.253	1.430	1.354	1.634	1.689
Q13177	Serine/threonine-protein kinase PAK 2 OS=Homo sapiens GN=PA	1.375	0.871	1.163	0.941	1.260	1.038	1.152	1.000
B4DSW9	cDNA FLJ59415, highly similar to Beta-catenin OS=Homo sapiens	1.443	1.857	1.623	1.622	1.402	1.423	2.028	2.126
B4DL14	ATP synthase subunit gamma OS=Homo sapiens PE=2 SV=1 - [B	1.289	1.371	1.139	1.094	0.968	1.098	1.301	1.157

P17174	Aspartate aminotransferase, cytoplasmic OS=Homo sapiens GN=	1.463	0.970	1.290	1.136	1.242	0.984	1.317	1.268
Q9Y2Q5	Ragulator complex protein LAMTOR2 OS=Homo sapiens GN=LAM	1.070	1.099	1.254	1.025	1.273	0.927	1.124	1.148
P35754	Glutaredoxin-1 OS=Homo sapiens GN=GLRX PE=1 SV=2 - [GLRX	0.868	0.617	1.062	0.954	0.876	0.857	0.905	0.772
Q5T3I0	G patch domain-containing protein 4 OS=Homo sapiens GN=GPA	1.578	1.879	1.649	1.486	1.496	1.796	1.762	1.656
A0A024R869	Golgi autoantigen, golgin subfamily a, 1, isoform CRA_a OS=Hom	1.591	1.579	1.320	1.272	1.544	1.460	1.582	1.602
O15173	Membrane-associated progesterone receptor component 2 OS=H	1.140	1.061	1.072	0.852	1.069	1.129	1.077	0.847
Q96JB5	CDK5 regulatory subunit-associated protein 3 OS=Homo sapiens	1.783	1.721	1.758	1.462	1.670	1.524	1.770	1.940
Q16594	Transcription initiation factor TFIID subunit 9 OS=Homo sapiens	1.021	1.160	0.880	0.764	1.171	1.312	1.042	1.066
Q8N2F6	Armadillo repeat-containing protein 10 OS=Homo sapiens GN=AR	1.215	1.497	1.107	0.982	1.004	1.117	1.190	1.157
Q7Z5U5	CCDC25 protein (Fragment) OS=Homo sapiens GN=CCDC25 PE=	1.223	1.087	1.602	1.218	1.267	1.031	1.154	1.005
Q9H845	Acyl-CoA dehydrogenase family member 9, mitochondrial OS=Ho	1.425	1.391	1.490	1.615	1.130	1.113	1.650	1.375
Q6UB35	Monofunctional C1-tetrahydrofolate synthase, mitochondrial OS=	1.287	1.187	1.099	1.018	1.072	1.161	1.050	0.961
P48163	NADP-dependent malic enzyme OS=Homo sapiens GN=ME1 PE=	1.215	0.787	1.101	0.924	1.032	0.940	1.003	0.941
A0A024R529	Dihydroxyacetone kinase 2 homolog (Yeast), isoform CRA_a OS=	1.316	1.019	1.471	1.162	1.193	0.978	1.215	1.088
Q9Y4Z0	U6 snRNA-associated Sm-like protein LSm4 OS=Homo sapiens GN	2.120	1.932	1.487	1.272	1.580	1.194	1.824	1.283
P05204	Non-histone chromosomal protein HMG-17 OS=Homo sapiens GN	1.532	1.885	1.516	1.257	1.300	1.239	1.433	1.275
P41250	Glycine--tRNA ligase OS=Homo sapiens GN=GARS PE=1 SV=3 -	1.646	1.101	1.498	1.155	1.320	1.091	1.303	1.288
Q9NZB2	Constitutive coactivator of PPAR-gamma-like protein 1 OS=Homo	1.326	1.298	1.082	0.737	1.129	1.134	1.127	0.815
Q96A57	Transmembrane protein 230 OS=Homo sapiens GN=TMEM230 PE	1.378	1.453	1.149	0.935	1.068	1.276	1.377	1.006
A0A087X0M4	Kanadaptin OS=Homo sapiens GN=SLC4A1AP PE=4 SV=1 - [A0A	1.301	1.359	1.211	0.873	1.427	1.323	1.234	1.103
P49189	4-trimethylaminobutyraldehyde dehydrogenase OS=Homo sapien	1.277	1.181	1.161	1.072	1.158	1.131	1.344	1.353
P26447	Protein S100-A4 OS=Homo sapiens GN=S100A4 PE=1 SV=1 - [S	1.371	0.764	1.040	0.784	1.147	0.996	1.045	0.879
O95881	Thioredoxin domain-containing protein 12 OS=Homo sapiens GN=	1.625	1.021	1.393	1.439	1.595	1.169	1.507	1.508
Q9NP77	RNA polymerase II subunit A C-terminal domain phosphatase SSU	1.366	1.421	1.333	0.741	1.387	1.442	1.174	0.833
G3V203	60S ribosomal protein L18 OS=Homo sapiens GN=RPL18 PE=1 S	1.699	1.527	1.468	1.160	1.568	1.233	1.502	1.301
Q9NQ55	Suppressor of SWI4 1 homolog OS=Homo sapiens GN=PPAN PE=	1.754	2.016	1.438	1.310	1.424	1.331	1.799	1.448
Q12974	Protein tyrosine phosphatase type IVA 2 OS=Homo sapiens GN=I	2.069	2.147	1.945	1.695	2.026	1.329	1.960	1.822
P32321	Deoxycytidylate deaminase OS=Homo sapiens GN=DCTD PE=1 S	1.118	1.008	1.334	1.027	0.809	1.043	0.895	0.988
Q16763	Ubiquitin-conjugating enzyme E2 S OS=Homo sapiens GN=UBE2S	1.923	1.798	1.747	1.035	1.904	1.758	2.103	1.576
P51571	Translocon-associated protein subunit delta OS=Homo sapiens GI	1.221	1.258	1.047	1.031	0.972	1.061	1.294	1.101
B7Z7P8	Eukaryotic peptide chain release factor subunit 1 OS=Homo sapie	3.005	2.047	2.608	2.038	2.060	1.208	2.130	1.772
A0A024R2H7	tRNA nucleotidyl transferase, CCA-adding, 1, isoform CRA_b OS=	1.296	1.217	1.204	1.120	1.072	1.151	1.255	1.059
S4R3N1	Protein HSPE1-MOB4 OS=Homo sapiens GN=HSPE1-MOB4 PE=3	2.098	1.576	1.887	1.420	1.844	1.369	1.772	1.495
Q13895	Bystin OS=Homo sapiens GN=BYSL PE=1 SV=3 - [BYST_HUMAN	1.342	1.580	1.513	1.142	1.492	1.431	1.752	1.253
Q27J81	Inverted formin-2 OS=Homo sapiens GN=INF2 PE=1 SV=2 - [INI	1.086	1.153	0.974	0.785	1.036	1.191	1.036	0.890
B3KQ21	cDNA FLJ32640 fis, clone SYNOV2001033, highly similar to U3 sn	1.646	1.503	0.976	0.819	1.548	1.388	1.343	0.773
Q8NBJ5	Procollagen galactosyltransferase 1 OS=Homo sapiens GN=COLG	1.316	1.057	1.187	1.406	1.441	1.216	1.359	1.343
Q9UJ83	2-hydroxyacyl-CoA lyase 1 OS=Homo sapiens GN=HACL1 PE=1 S	1.063	1.130	1.025	1.076	0.965	1.106	1.023	1.053
Q92934	Bcl2-associated agonist of cell death OS=Homo sapiens GN=BAD	1.415	1.914	1.227	1.231	1.582	1.412	1.434	1.420
P18084	Integrin beta-5 OS=Homo sapiens GN=ITGB5 PE=1 SV=1 - [ITB	1.150	1.312	0.982	1.062	1.146	1.055	1.227	0.962

P46109	Crk-like protein OS=Homo sapiens GN=CRKL PE=1 SV=1 - [CRKL	1.515	1.064	1.509	1.132	1.334	1.116	1.390	1.583
Q9UG63	ATP-binding cassette sub-family F member 2 OS=Homo sapiens (	1.447	1.549	1.709	1.132	1.297	1.263	1.648	1.257
P62070	Ras-related protein R-Ras2 OS=Homo sapiens GN=RRAS2 PE=1 S	1.015	1.584	1.101	1.032	1.226	1.125	1.088	1.221
Q96C19	EF-hand domain-containing protein D2 OS=Homo sapiens GN=EF	1.751	1.594	1.934	1.089	1.413	0.900	1.213	1.528
Q15813	Tubulin-specific chaperone E OS=Homo sapiens GN=TBCE PE=1	1.303	0.841	1.135	0.935	1.108	1.037	1.030	1.019
B4DL66	cDNA FLJ56297, highly similar to Rattus norvegicus ubiquitin-conj	2.141	1.901	2.155	1.485	1.629	1.105	1.467	1.568
F6T1Q0	2',5'-phosphodiesterase 12 OS=Homo sapiens GN=PDE12 PE=1 S	1.323	1.297	1.141	1.032	1.137	1.292	1.387	1.155
Q0VGA5	SARS protein OS=Homo sapiens GN=SARS PE=2 SV=1 - [Q0VGA	1.372	1.049	1.365	1.112	1.282	1.090	1.324	1.267
A8K8U1	cDNA FLJ77762, highly similar to Homo sapiens cullin-associated	1.382	1.072	1.339	1.049	1.292	1.048	1.249	1.085
B2R802	cDNA, FLJ93681, highly similar to Homo sapiens small nuclear rib	1.086	1.237	1.030	0.755	1.162	1.232	1.079	0.992
Q9Y3B9	RRP15-like protein OS=Homo sapiens GN=RRP15 PE=1 SV=2 - [	1.241	1.460	1.528	1.391	1.326	1.355	1.507	1.116
Q92890	Ubiquitin fusion degradation protein 1 homolog OS=Homo sapien	1.172	0.824	0.999	0.719	1.272	1.073	0.975	0.827
A8K7Z3	cDNA FLJ77229, highly similar to Homo sapiens GRIP1 associated	1.450	0.962	2.028	2.309	1.227	1.009	1.430	1.799
P60866	40S ribosomal protein S20 OS=Homo sapiens GN=RPS20 PE=1 S	1.562	1.314	1.437	1.173	1.142	1.114	1.218	1.224
Q9NSI2	Protein FAM207A OS=Homo sapiens GN=FAM207A PE=1 SV=2 -	1.388	1.740	1.011	0.676	1.365	1.291	1.222	0.662
P61927	60S ribosomal protein L37 OS=Homo sapiens GN=RPL37 PE=1 S	1.274	1.388	1.269	0.971	1.507	1.280	1.475	1.193
Q14232	Translation initiation factor eIF-2B subunit alpha OS=Homo sapie	1.455	1.162	1.377	1.069	1.350	1.178	1.356	1.429
Q9Y676	28S ribosomal protein S18b, mitochondrial OS=Homo sapiens GN	1.655	1.776	1.403	1.358	1.159	1.073	1.599	1.430
C9J2P0	Ubiquitin-conjugating enzyme E2 E1 (Fragment) OS=Homo sapie	1.203	0.962	1.216	0.903	1.221	1.221	1.066	1.081
E5RH67	Centrin-3 OS=Homo sapiens GN=CETN3 PE=4 SV=1 - [E5RH67_	1.368	1.482	1.249	0.985	1.168	1.225	1.510	1.322
Q96A33	Coiled-coil domain-containing protein 47 OS=Homo sapiens GN=(	2.171	2.134	1.661	1.666	1.444	1.550	1.869	1.711
P53007	Tricarboxylate transport protein, mitochondrial OS=Homo sapiens	1.275	1.396	1.076	1.079	0.961	1.058	1.189	1.164
P26640	Valine--tRNA ligase OS=Homo sapiens GN=VARS PE=1 SV=4 - [S	1.302	0.945	1.124	0.885	1.183	0.999	1.113	0.937
A0A024QZW7	Nucleoporin 153kDa, isoform CRA_a OS=Homo sapiens GN=NUP	1.150	1.289	0.895	0.868	1.018	1.224	1.094	0.926
Q9UL25	Ras-related protein Rab-21 OS=Homo sapiens GN=RAB21 PE=1 S	1.318	1.539	1.310	1.409	1.116	1.270	1.356	1.353
Q9BYD2	39S ribosomal protein L9, mitochondrial OS=Homo sapiens GN=N	1.392	1.524	1.226	1.202	1.022	1.106	1.310	1.483
Q969Q5	Ras-related protein Rab-24 OS=Homo sapiens GN=RAB24 PE=1 S	1.067	1.583	1.529	1.515	1.025	1.243	1.714	1.652
E5KN59	Peptidyl-prolyl cis-trans isomerase D OS=Homo sapiens PE=4 SV=	1.339	1.061	1.233	1.007	1.417	1.114	1.342	1.215
P54727	UV excision repair protein RAD23 homolog B OS=Homo sapiens (	1.264	0.948	1.188	0.951	1.146	1.180	1.258	0.955
Q96C36	Pyrroline-5-carboxylate reductase 2 OS=Homo sapiens GN=PYCR	1.259	1.291	0.976	0.984	0.981	1.113	1.188	1.164
P0C0S5	Histone H2A.Z OS=Homo sapiens GN=H2AFZ PE=1 SV=2 - [H2A	1.480	1.727	1.491	1.369	1.404	1.208	1.515	1.635
Q8N2K0	Monoacylglycerol lipase ABHD12 OS=Homo sapiens GN=ABHD12	1.441	1.193	1.199	1.027	0.950	1.099	1.110	1.055
P83876	Thioredoxin-like protein 4A OS=Homo sapiens GN=TXNL4A PE=1	2.163	2.251	1.849	1.520	2.467	1.729	2.352	2.304
P53680	AP-2 complex subunit sigma OS=Homo sapiens GN=AP2S1 PE=1	0.987	1.102	0.991	0.883	1.035	1.045	1.198	1.080
A1L0T0	Acetolactate synthase-like protein OS=Homo sapiens GN=ILVBL F	1.424	1.399	1.199	1.176	1.148	1.235	1.358	1.217
P52435	DNA-directed RNA polymerase II subunit RPB11-a OS=Homo sap	0.677	0.779	0.635	0.558	0.915	1.253	0.955	0.583
Q6NZI2	Polymerase I and transcript release factor OS=Homo sapiens GN=	1.193	1.140	1.122	1.051	1.284	1.070	0.944	0.883
P40222	Alpha-taxilin OS=Homo sapiens GN=TXLNA PE=1 SV=3 - [TXLNA	1.507	1.266	1.311	0.950	1.445	1.437	1.371	1.179
Q9Y3B2	Exosome complex component CSL4 OS=Homo sapiens GN=EXOS	1.462	1.580	1.487	1.212	1.391	1.356	1.610	1.335
P61221	ATP-binding cassette sub-family E member 1 OS=Homo sapiens (	1.437	1.019	1.417	0.956	1.182	1.071	1.235	1.000

Q96B26	Exosome complex component RRP43 OS=Homo sapiens GN=EXO	2.986	3.175	2.463	2.998	2.696	1.557	2.629	2.845
P21283	V-type proton ATPase subunit C 1 OS=Homo sapiens GN=ATP6V1	1.890	1.281	1.582	1.345	1.692	1.174	1.606	1.574
Q7KZ85	Transcription elongation factor SPT6 OS=Homo sapiens GN=SUPT	1.440	1.670	1.477	1.234	1.492	1.507	1.580	1.389
A8KAQ6	cDNA FLJ76490, highly similar to Homo sapiens ancient ubiquitou	1.222	1.306	1.124	1.145	1.018	1.294	1.324	1.188
Q9Y2R0	Cytochrome c oxidase assembly factor 3 homolog, mitochondrial	1.502	1.648	1.188	1.227	1.101	1.134	1.501	1.458
F8W0J4	YEATS domain-containing protein 4 OS=Homo sapiens GN=YEAT	1.958	2.246	1.713	1.240	1.836	1.581	1.946	1.837
P11766	Alcohol dehydrogenase class-3 OS=Homo sapiens GN=ADH5 PE=	0.985	0.748	1.068	0.922	1.062	0.976	0.928	0.966
Q15435	Protein phosphatase 1 regulatory subunit 7 OS=Homo sapiens GN	1.158	0.922	1.253	1.207	1.070	1.030	1.311	1.076
Q99613	Eukaryotic translation initiation factor 3 subunit C OS=Homo sapi	1.489	1.479	1.384	1.045	1.357	1.181	1.271	1.125
Q3SYF1	Sorting nexin 12 OS=Homo sapiens GN=SNX12 PE=2 SV=1 - [Q3	1.063	0.730	1.065	0.801	1.051	1.014	1.011	0.919
Q9ULR0	Pre-mRNA-splicing factor ISY1 homolog OS=Homo sapiens GN=IS	1.511	1.784	1.484	1.195	1.413	1.446	1.445	0.986
O60763	General vesicular transport factor p115 OS=Homo sapiens GN=U	1.594	1.024	1.272	0.987	1.366	1.058	1.103	0.936
Q8NCW5	NAD(P)H-hydrate epimerase OS=Homo sapiens GN=APOA1BP PE	1.205	1.197	1.291	1.209	1.098	1.124	1.242	1.158
Q29RF7	Sister chromatid cohesion protein PDS5 homolog A OS=Homo sap	1.742	1.890	1.555	1.229	1.433	1.309	1.886	1.493
Q53FE5	Putative uncharacterized protein (Fragment) OS=Homo sapiens P	1.358	1.108	1.475	1.165	1.152	1.134	1.192	1.227
Q6FIA3	PACSIN2 protein OS=Homo sapiens GN=PACSIN2 PE=2 SV=1 - [	1.387	0.861	1.112	0.860	1.205	0.999	1.145	1.037
B4DRM3	cDNA FLJ54492, highly similar to Eukaryotic translation initiation	1.346	0.970	1.317	0.986	1.393	1.224	1.356	1.046
O43676	NADH dehydrogenase [ubiquinone] 1 beta subcomplex subunit 3	1.420	1.608	1.266	1.129	1.033	1.004	1.413	1.298
O75940	Survival of motor neuron-related-splicing factor 30 OS=Homo sap	1.132	1.444	1.013	0.823	1.555	1.259	1.633	1.131
Q02127	Dihydroorotate dehydrogenase (quinone), mitochondrial OS=Hom	1.135	1.585	1.263	1.342	0.981	1.186	1.371	1.257
B4DZJ7	Transcription elongation factor SPT5 OS=Homo sapiens PE=2 SV	1.429	1.354	1.418	1.077	1.523	1.403	1.391	1.244
Q9Y5K6	CD2-associated protein OS=Homo sapiens GN=CD2AP PE=1 SV=	1.189	0.801	1.288	0.894	1.116	0.968	1.092	0.996
Q8WVJ2	NudC domain-containing protein 2 OS=Homo sapiens GN=NUDC	1.538	0.932	1.865	1.233	1.154	0.935	1.168	1.025
B3LEU8	TMEM214 protein (Fragment) OS=Homo sapiens GN=TMEM214 F	1.461	1.711	1.344	1.145	1.200	1.187	1.503	1.293
Q15397	Pumilio domain-containing protein KIAA0020 OS=Homo sapiens (	1.591	1.740	1.466	1.090	1.406	1.332	1.451	1.171
B2RBP3	cDNA, FLJ95615, highly similar to Homo sapiens ubiquitin-activat	1.492	1.269	1.342	1.169	1.575	1.114	1.198	1.112
A6QKW0	SHINC3 OS=Homo sapiens GN=SHINC3 PE=2 SV=1 - [A6QKW0_	1.743	1.634	1.447	1.169	1.504	1.278	1.710	1.263
P61244	Protein max OS=Homo sapiens GN=MAX PE=1 SV=1 - [MAX_HU	1.342	1.653	1.529	1.465	1.370	1.393	1.619	1.861
O15400	Syntaxin-7 OS=Homo sapiens GN=STX7 PE=1 SV=4 - [STX7_HU	1.310	1.668	1.090	1.129	1.207	1.177	1.261	1.249
P23368	NAD-dependent malic enzyme, mitochondrial OS=Homo sapiens (	1.557	1.512	1.410	1.287	1.062	1.206	1.520	1.570
Q8NI22	Multiple coagulation factor deficiency protein 2 OS=Homo sapiens	4.477	4.397	5.107	4.872	4.132	1.584	4.884	5.193
Q7Z518	NADH dehydrogenase OS=Homo sapiens PE=2 SV=1 - [Q7Z518_	1.391	1.534	1.308	1.139	1.093	1.259	1.352	1.323
Q9UNK0	Syntaxin-8 OS=Homo sapiens GN=STX8 PE=1 SV=2 - [STX8_HU	1.237	1.457	1.160	1.043	1.249	1.288	1.418	1.360
O94973	AP-2 complex subunit alpha-2 OS=Homo sapiens GN=AP2A2 PE=	1.279	1.492	1.324	0.994	1.243	1.084	1.360	1.317
Q8TBT6	Putative uncharacterized protein (Fragment) OS=Homo sapiens P	1.175	1.860	1.155	1.535	1.503	1.315	1.809	1.077
Q2TSD2	Aging-associated gene 7 protein OS=Homo sapiens PE=2 SV=1 -	1.377	1.183	0.992	0.651	1.361	1.274	1.191	0.879
H3BV80	RNA-binding protein with serine-rich domain 1 OS=Homo sapiens	1.332	1.511	1.286	1.372	1.101	1.169	1.253	1.399
Q9NPD8	Ubiquitin-conjugating enzyme E2 T OS=Homo sapiens GN=UBE2T	1.304	1.222	1.421	0.901	1.266	1.286	1.301	1.259
Q9BWD1	Acetyl-CoA acetyltransferase, cytosolic OS=Homo sapiens GN=AC	1.736	0.750	1.595	1.048	1.164	0.843	1.141	1.423
P30038	Delta-1-pyrroline-5-carboxylate dehydrogenase, mitochondrial OS	1.091	1.180	0.950	0.990	0.959	1.188	1.131	1.064

B2R7R5	cDNA, FLJ93570, highly similar to Homo sapiens phosphoribosyl p	1.213	0.801	1.177	0.890	1.261	1.057	0.988	0.947
Q9BQP7	Mitochondrial genome maintenance exonuclease 1 OS=Homo sap	1.911	1.978	1.232	1.294	1.538	1.403	1.630	1.845
Q8WUY8	N-acetyltransferase 14 OS=Homo sapiens GN=NAT14 PE=1 SV=1	1.531	1.258	1.296	1.244	1.204	1.094	1.344	1.239
P0C7P0	CDGSH iron-sulfur domain-containing protein 3, mitochondrial OS	2.472	2.678	2.488	2.193	1.411	1.406	1.969	1.969
Q9UNX4	WD repeat-containing protein 3 OS=Homo sapiens GN=WDR3 PE	2.145	2.329	2.134	2.011	2.022	1.495	2.301	1.925
B3KMV5	cDNA FLJ12728 fis, clone NT2RP2000040, highly similar to Protei	1.557	1.778	1.650	1.615	1.590	1.439	1.853	1.656
Q99496	E3 ubiquitin-protein ligase RING2 OS=Homo sapiens GN=RNF2 P	1.484	1.520	1.402	1.353	1.352	1.497	1.755	1.557
Q13443	Disintegrin and metalloproteinase domain-containing protein 9 OS	1.447	1.503	1.273	1.061	1.013	1.086	1.015	0.965
O60826	Coiled-coil domain-containing protein 22 OS=Homo sapiens GN=(	1.463	1.060	1.311	1.014	1.314	1.085	1.315	1.315
Q53H96	Pyrroline-5-carboxylate reductase 3 OS=Homo sapiens GN=PYCR	1.724	1.078	1.479	1.355	1.458	1.279	1.330	1.099
Q14320	Protein FAM50A OS=Homo sapiens GN=FAM50A PE=1 SV=2 - [FA	1.228	1.087	1.158	0.848	1.212	1.213	1.148	1.060
B2RCM2	cDNA, FLJ96156, highly similar to Homo sapiens leucyl-tRNA synt	1.230	1.147	1.187	0.935	1.070	1.005	1.136	0.954
O15294	UDP-N-acetylglucosamine--peptide N-acetylglucosaminyltransfera	1.384	1.524	1.449	1.122	1.495	1.427	1.546	1.298
A0A087WY55	Chromosome 6 open reading frame 55, isoform CRA_b OS=Homo	1.523	0.940	1.616	1.406	1.452	1.057	1.409	1.235
Q9Y2S0	DNA-directed RNA polymerases I and III subunit RPAC2 OS=Hom	1.490	1.743	1.629	1.441	1.430	1.386	1.696	1.497
P52564	Dual specificity mitogen-activated protein kinase kinase 6 OS=Ho	1.035	0.695	0.979	0.736	0.806	0.839	1.050	0.599
Q86WX3	Active regulator of SIRT1 OS=Homo sapiens GN=RPS19BP1 PE=1	1.944	2.508	1.349	0.844	1.975	1.411	1.821	1.106
F1T0L5	ATP-dependent RNA helicase DDX51 OS=Homo sapiens GN=DDX	1.489	1.824	1.498	1.182	1.673	1.505	1.589	1.503
F5GXX5	Dolichyl-diphosphooligosaccharide--protein glycosyltransferase su	2.726	2.566	2.161	1.871	2.146	0.948	2.190	1.945
Q8WW59	SPRY domain-containing protein 4 OS=Homo sapiens GN=SPRYD	1.588	1.557	1.225	1.537	1.326	1.209	1.592	1.626
A0A087WSV8	DDB1- and CUL4-associated factor 5 OS=Homo sapiens GN=DCA	1.453	1.302	1.313	1.343	1.299	1.172	1.493	1.229
A0A024QZJ7	Coiled-coil domain containing 6, isoform CRA_a OS=Homo sapien	1.368	0.874	1.208	0.929	1.211	1.056	1.181	1.001
P49840	Glycogen synthase kinase-3 alpha OS=Homo sapiens GN=GSK3A	1.322	0.851	1.405	1.078	1.184	1.061	1.176	1.088
P62851	40S ribosomal protein S25 OS=Homo sapiens GN=RPS25 PE=1 S	1.389	1.399	1.448	0.977	1.385	1.230	1.243	1.088
B3KNS8	cDNA FLJ30322 fis, clone BRACE2006703, highly similar to Surfei	2.013	2.204	1.663	1.310	2.096	1.628	1.967	1.428
B4DNI0	cDNA FLJ51004, highly similar to WD repeat protein 46 OS=Homo	1.868	2.595	1.850	1.496	1.717	1.711	2.132	1.483
Q14690	Protein RRP5 homolog OS=Homo sapiens GN=PDCD11 PE=1 SV=	1.529	1.816	1.417	1.123	1.468	1.432	1.624	1.227
Q13823	Nucleolar GTP-binding protein 2 OS=Homo sapiens GN=GNL2 PE	1.622	1.991	1.437	1.226	1.652	1.666	1.970	1.482
Q52LJ0	Protein FAM98B OS=Homo sapiens GN=FAM98B PE=1 SV=1 - [FA	1.053	1.076	0.915	0.859	1.170	1.149	1.002	0.775
Q8WXV6	Plectin isoform 1a (Fragment) OS=Homo sapiens GN=PLEC1 PE=	0.767	0.664	0.632	0.516	2.070	1.277	2.206	1.120
B7Z4K8	cDNA FLJ52869, highly similar to Homo sapiens basic leucine zipp	1.632	1.367	1.337	1.046	1.338	1.090	1.296	1.157
P51843	Nuclear receptor subfamily 0 group B member 1 OS=Homo sapie	1.713	1.435	0.633	0.508	1.793	1.328	1.179	0.797
Q07021	Complement component 1 Q subcomponent-binding protein, mito	2.652	2.910	2.430	2.843	2.101	1.333	2.663	2.963
Q92888	Rho guanine nucleotide exchange factor 1 OS=Homo sapiens GN	1.400	0.946	1.217	0.904	1.188	0.978	1.193	1.018
A5D8W6	Mortality factor 4 like 1 OS=Homo sapiens GN=MORF4L1 PE=2 S	1.186	1.406	0.974	0.836	1.162	1.366	1.317	1.021
G8JLD5	Dynamin-1-like protein OS=Homo sapiens GN=DNM1L PE=1 SV=	0.750	0.498	0.676	0.592	0.980	0.942	0.830	0.740
H7C0E5	Zinc finger protein ZPR1 (Fragment) OS=Homo sapiens GN=ZPR1	1.373	0.935	1.484	1.224	1.379	1.226	1.191	1.298
A0A024RCB5	Chitinase domain containing 1, isoform CRA_a OS=Homo sapiens	1.571	1.409	1.803	1.685	1.526	1.263	1.678	1.719
P62266	40S ribosomal protein S23 OS=Homo sapiens GN=RPS23 PE=1 S	1.582	1.444	1.328	0.924	1.502	1.125	1.404	1.221
O15382	Branched-chain-amino-acid aminotransferase, mitochondrial OS=	1.594	1.428	1.662	1.555	1.118	1.324	1.478	1.456

B2RD19	cDNA, FLJ96419, highly similar to Homo sapiens fructosamine-3-k	1.374	0.850	1.342	1.094	1.154	1.049	1.504	1.092
Q96D53	AarF domain-containing protein kinase 4 OS=Homo sapiens GN=A	1.712	1.532	1.202	1.162	1.267	1.225	1.451	1.534
P13645	Keratin, type I cytoskeletal 10 OS=Homo sapiens GN=KRT10 PE=	1.944	1.519	1.826	3.380	1.465	1.487	3.850	1.790
Q08379	Golgin subfamily A member 2 OS=Homo sapiens GN=GOLGA2 PE	1.495	1.518	1.265	1.172	1.267	1.344	1.376	1.213
Q96QK1	Vacuolar protein sorting-associated protein 35 OS=Homo sapiens	1.874	1.363	1.745	1.377	1.521	1.242	1.582	1.297
P56182	Ribosomal RNA processing protein 1 homolog A OS=Homo sapien	1.715	1.799	1.369	1.383	1.419	1.447	1.767	1.439
A8K4T6	cDNA FLJ76282, highly similar to Homo sapiens proteasome (pro	1.195	0.860	1.187	0.949	1.240	1.017	1.027	0.933
B4DJI2	cDNA FLJ53342, highly similar to Granulins OS=Homo sapiens PE	1.559	1.418	1.075	0.884	1.408	1.162	1.159	1.074
B4DM78	cDNA FLJ58199, highly similar to Fragile X mental retardation syn					0.714	1.277	1.102	
Q12792	Twinfilin-1 OS=Homo sapiens GN=TWF1 PE=1 SV=3 - [TWF1_HU	1.104	0.868	1.005	0.855	1.015	0.988	0.888	0.892
Q9P032	NADH dehydrogenase [ubiquinone] 1 alpha subcomplex assembly	1.570	1.816	1.623	1.513	1.158	1.287	1.678	1.560
Q3ZCQ8	Mitochondrial import inner membrane translocase subunit TIM50	1.487	1.308	1.210	1.574	1.014	1.086	1.207	1.184
O00625	Pirin OS=Homo sapiens GN=PIR PE=1 SV=1 - [PIR_HUMAN]	1.127	0.784	1.111	0.930	1.087	0.895	0.933	0.974
P11177	Pyruvate dehydrogenase E1 component subunit beta, mitochondri	1.261	1.254	1.202	1.241	0.973	1.103	1.186	1.199
Q53FI8	DNA-directed RNA polymerase III 39 kDa polypeptide F variant (F	1.423	1.569	1.239	1.127	1.457	1.542	1.448	1.540
F5GWX5	Chromodomain-helicase-DNA-binding protein 4 OS=Homo sapien	1.567	1.830	1.219	0.989	1.373	1.254	1.636	1.245
O94919	Endonuclease domain-containing 1 protein OS=Homo sapiens GN	1.356	1.370	1.324	1.547	1.085	1.521	1.427	1.284
H0YDR3	Tetratricopeptide repeat protein 9C (Fragment) OS=Homo sapien	1.697	1.085	1.346	1.062	1.332	1.074	1.338	1.218
Q8N995	cDNA FLJ38173 fis, clone FCBBF1000053, highly similar to HYDR(	1.974	1.433	1.863	1.224	1.979	1.398	1.512	1.189
P63096	Guanine nucleotide-binding protein G(i) subunit alpha-1 OS=Hom	2.153	2.571	2.140	1.969	1.797	1.148	1.555	1.570
B2R5I8	cDNA, FLJ92490, highly similar to Homo sapiens RAB32, member	1.538	1.650	1.434	1.272	1.330	1.326	1.580	1.336
Q15404	Ras suppressor protein 1 OS=Homo sapiens GN=RSU1 PE=1 SV=	1.234	0.930	1.248	0.904	1.022	0.935	1.103	0.969
Q3ZAQ7	Vacuolar ATPase assembly integral membrane protein VMA21 OS=	3.237	3.192	3.068	2.436	2.239	1.854	2.838	2.574
H7C5G1	Isoamyl acetate-hydrolyzing esterase 1 homolog (Fragment) OS=	1.097	0.687	1.101	0.950	0.981	0.898	0.837	0.775
Q7Z426	Putative MAPK activating protein OS=Homo sapiens PE=2 SV=1 -	1.766	1.057	1.715	1.202	1.225	1.134	1.308	1.312
P05556	Integrin beta-1 OS=Homo sapiens GN=ITGB1 PE=1 SV=2 - [ITB1	1.223	1.353	1.006	0.936	1.109	1.155	1.074	0.889
Q15020	Squamous cell carcinoma antigen recognized by T-cells 3 OS=Hor	1.306	1.262	1.192	0.857	1.406	1.132	1.141	0.732
A6NDG6	Phosphoglycolate phosphatase OS=Homo sapiens GN=PGP PE=1	1.787	1.129	1.581	1.185	1.468	0.980	1.753	1.501
Q7Z5L9	Interferon regulatory factor 2-binding protein 2 OS=Homo sapien	0.923	0.758	0.520	0.349	0.948	0.933	0.569	0.354
Q9HC03	Dolichyl-phosphate beta-glucosyltransferase OS=Homo sapiens P	1.132	1.247	1.033	0.867	1.136	1.297	1.282	1.086
Q03169	Tumor necrosis factor alpha-induced protein 2 OS=Homo sapiens	1.768	1.435	1.237	0.964	1.575	1.134	1.169	0.999
P83111	Serine beta-lactamase-like protein LACTB, mitochondrial OS=Hon	1.759	1.819	1.295	1.254	1.689	1.229	1.557	1.254
O60568	Procollagen-lysine,2-oxoglutarate 5-dioxygenase 3 OS=Homo sap	1.413	1.132	1.154	1.253	1.328	1.107	1.241	1.459
P08621	U1 small nuclear ribonucleoprotein 70 kDa OS=Homo sapiens GN	1.327	1.486	1.086	0.807	1.278	1.225	1.364	1.216
P07686	Beta-hexosaminidase subunit beta OS=Homo sapiens GN=HEXB	1.349	1.244	1.305	1.320	1.461	1.177	1.403	1.554
O43865	Putative adenosylhomocysteinase 2 OS=Homo sapiens GN=AHCY	1.137	1.892	1.175	0.964	1.022	0.810	0.946	0.998
O75410	Transforming acidic coiled-coil-containing protein 1 OS=Homo sap	1.687	2.315	1.404	1.236	1.570	1.434	1.710	1.655
P51003	Poly(A) polymerase alpha OS=Homo sapiens GN=PAPOLA PE=1 S	1.335	1.045	1.126	0.891	1.380	1.245	1.122	0.874
Q9UNF1	Melanoma-associated antigen D2 OS=Homo sapiens GN=MAGED	1.358	1.072	1.370	1.067	1.199	1.058	1.417	1.497
O60884	DnaJ homolog subfamily A member 2 OS=Homo sapiens GN=DN	6.834	4.597	5.084	4.398	4.836	1.543	5.230	5.842

Q14318	Peptidyl-prolyl cis-trans isomerase FKBP8 OS=Homo sapiens GN=	1.051	1.089	0.865	0.758	0.863	1.125	1.104	0.901
Q9Y399	28S ribosomal protein S2, mitochondrial OS=Homo sapiens GN=M	1.622	1.724	1.514	1.443	1.005	1.190	1.644	1.352
F2Z3M0	tRNA-splicing endonuclease subunit Sen15 OS=Homo sapiens GN	0.954	0.828	0.772	0.604	1.004	0.948	0.728	0.606
Q03701	CCAAT/enhancer-binding protein zeta OS=Homo sapiens GN=CEE	0.729	0.866	0.698	0.747	1.204	1.477	1.218	0.871
A0A087WY54	Zinc finger HIT domain-containing protein 3 (Fragment) OS=Hom	3.224	3.528	2.884	1.889	2.426	2.500	3.170	2.299
P61964	WD repeat-containing protein 5 OS=Homo sapiens GN=WDR5 PE	1.990	2.799	2.140	1.638	1.935	1.396	2.248	2.426
P62256	Ubiquitin-conjugating enzyme E2 H OS=Homo sapiens GN=UBE2	1.443	0.855	1.396	1.008	1.174	1.126	1.231	1.140
A8KA74	cDNA FLJ76065 OS=Homo sapiens PE=2 SV=1 - [A8KA74_HUMA	1.456	1.645	1.157	1.026	1.315	1.332	1.378	1.079
Q5JPH6	Probable glutamate--tRNA ligase, mitochondrial OS=Homo sapier	1.307	1.467	1.219	1.249	1.054	1.222	1.373	1.352
Q5JPE7	Nodal modulator 2 OS=Homo sapiens GN=NOMO2 PE=1 SV=1 -	3.129	4.010	3.500	3.063	3.081	2.342	4.077	2.831
O00165	HCLS1-associated protein X-1 OS=Homo sapiens GN=HAX1 PE=1	1.260	1.234	1.120	1.073	1.325	1.238	1.305	1.347
A0A087X020	Ribosome maturation protein SBDS OS=Homo sapiens GN=SBDS	1.077	0.902	0.921	0.740	0.879	0.985	0.965	0.826
Q53F35	Acidic (Leucine-rich) nuclear phosphoprotein 32 family, member E	1.125	0.911	1.198	0.832	1.044	1.131	0.908	0.904
C9JFE4	COP9 signalosome complex subunit 1 OS=Homo sapiens GN=GPS	1.596	1.198	1.415	0.939	1.381	1.245	1.280	1.044
Q96I15	Selenocysteine lyase OS=Homo sapiens GN=SCLY PE=1 SV=4 - [	0.938	0.936	1.318	0.871	1.014	1.345	1.234	0.850
Q5VTR2	E3 ubiquitin-protein ligase BRE1A OS=Homo sapiens GN=RNF20	1.430	1.445	1.220	0.860	1.393	1.378	1.358	1.028
A0A024RC67	Protein regulator of cytokinesis 1, isoform CRA_e OS=Homo sapie	1.483	1.622	1.681	1.496	1.059	1.446	1.838	1.761
Q10567	AP-1 complex subunit beta-1 OS=Homo sapiens GN=AP1B1 PE=1	1.250	1.194	1.120	1.036	1.191	1.071	1.252	1.237
A0A087X1K9	Acyl-protein thioesterase 1 OS=Homo sapiens GN=LYPLA1 PE=4	1.221	0.953	1.155	1.001	0.921	0.990	1.079	1.072
Q9H6W3	Bifunctional lysine-specific demethylase and histidyl-hydroxylase I	1.475	1.589	1.408	1.255	1.417	1.577	1.709	1.526
Q9HD15	Steroid receptor RNA activator 1 OS=Homo sapiens GN=SRA1 PE	1.953	1.264	2.094	1.416	1.634	1.096	1.478	2.005
P50897	Palmitoyl-protein thioesterase 1 OS=Homo sapiens GN=PPT1 PE=	1.161	1.214	1.394	1.095	0.894	1.185	1.459	1.370
Q9BTE3	Mini-chromosome maintenance complex-binding protein OS=Hom	1.487	1.289	1.437	1.068	1.300	1.311	1.459	1.182
O95232	Luc7-like protein 3 OS=Homo sapiens GN=LUC7L3 PE=1 SV=2 -	2.107	2.180	2.073	1.869	2.025	1.730	2.244	1.814
Q9UKL0	REST corepressor 1 OS=Homo sapiens GN=RCOR1 PE=1 SV=1 -	1.302	1.241	0.806	0.610	1.316	1.143	1.099	0.973
Q9NXF1	Testis-expressed sequence 10 protein OS=Homo sapiens GN=TEX	1.911	1.850	1.442	1.003	1.701	1.306	1.645	1.056
Q9P016	Thymocyte nuclear protein 1 OS=Homo sapiens GN=THYN1 PE=	1.291	1.294	1.296	1.067	1.321	1.098	1.287	1.059
B2R9C5	cDNA, FLJ94330 OS=Homo sapiens PE=2 SV=1 - [B2R9C5_HUMA	0.932	1.147	0.887	0.741	1.125	1.434	1.175	0.790
Q9Y5U9	Immediate early response 3-interacting protein 1 OS=Homo sapie	1.685	1.964	1.791	1.791	1.432	1.375	1.781	1.435
Q86XZ4	Spermatogenesis-associated serine-rich protein 2 OS=Homo sapie	1.509	1.163	1.197	0.795	1.454	1.123	1.199	1.114
P49848	Transcription initiation factor TFIID subunit 6 OS=Homo sapiens G	1.241	1.500	1.169	1.106	1.278	1.264	1.494	1.398
A4D2P0	Ras-related C3 botulinum toxin substrate 1 (Rho family, small GTI	0.963	0.839	0.700	0.679	1.058	1.122	1.006	0.871
Q59F44	Cytochrome b-5 isoform 1 variant (Fragment) OS=Homo sapiens	1.203	1.107	1.024	0.934	1.071	1.136	1.127	0.999
Q9NUQ6	SPATS2-like protein OS=Homo sapiens GN=SPATS2L PE=1 SV=2	1.667	1.093	1.012	0.820	1.586	1.190	1.091	1.322
Q8NFH4	Nucleoporin Nup37 OS=Homo sapiens GN=NUP37 PE=1 SV=1 -	1.510	1.504	1.312	1.471	1.524	1.415	1.583	1.655
P45974	Ubiquitin carboxyl-terminal hydrolase 5 OS=Homo sapiens GN=U	1.192	0.856	1.174	0.965	1.155	0.927	1.063	0.953
B2RE34	cDNA, FLJ96901, highly similar to Homo sapiens Rac GTPase acti	1.316	1.381	0.826	0.412	1.134	1.073	1.144	0.714
Q5BKZ1	DBIRD complex subunit ZNF326 OS=Homo sapiens GN=ZNF326	1.277	1.311	1.152	0.898	1.256	1.211	1.214	1.040
Q5QP19	NFS1 nitrogen fixation 1 (S. cerevisiae), isoform CRA_b OS=Homo	1.141	1.301	1.026	1.011	0.991	1.207	1.062	0.982
B2R9J4	cDNA, FLJ94423, highly similar to Homo sapiens mitochondrial rib	1.667	1.802	1.582	1.774	1.362	1.120	1.412	1.383

P98175	RNA-binding protein 10 OS=Homo sapiens GN=RBM10 PE=1 SV=	1.395	1.541	1.285	1.045	1.393	1.328	1.549	1.205
Q562Z4	Actin-like protein (Fragment) OS=Homo sapiens GN=ACT PE=3 S	4.222	4.100	3.381	3.639	3.113	2.005	3.305	2.580
O96011	Peroxisomal membrane protein 11B OS=Homo sapiens GN=PEX1	2.037	1.875	1.683	1.628	1.448	1.464	1.774	1.674
Q99747	Gamma-soluble NSF attachment protein OS=Homo sapiens GN=N	1.329	1.194	1.208	1.095	1.163	1.124	1.373	1.426
Q86VR6	DDX10 protein (Fragment) OS=Homo sapiens GN=DDX10 PE=2 S	1.496	1.686	1.307	1.156	1.323	1.443	1.518	1.314
Q8N1G4	Leucine-rich repeat-containing protein 47 OS=Homo sapiens GN=	1.373	0.941	1.225	0.801	1.186	1.001	1.236	1.048
P49589	Cysteine--tRNA ligase, cytoplasmic OS=Homo sapiens GN=CARS	2.148	1.420	1.758	1.235	1.588	0.983	1.231	1.057
Q6DKK2	Tetratricopeptide repeat protein 19, mitochondrial OS=Homo sapi	1.402	1.479	1.239	1.177	1.424	1.310	1.430	1.492
Q8N766	ER membrane protein complex subunit 1 OS=Homo sapiens GN=	1.721	1.720	1.497	1.420	1.385	1.306	1.650	1.483
Q8NBJ4	Golgi membrane protein 1 OS=Homo sapiens GN=GOLM1 PE=1 S	1.313	1.269	1.650	1.336	1.085	1.060	1.459	1.211
Q96I25	Splicing factor 45 OS=Homo sapiens GN=RBM17 PE=1 SV=1 - [S	1.617	1.606	1.479	1.360	1.379	1.395	1.577	1.336
P78318	Immunoglobulin-binding protein 1 OS=Homo sapiens GN=IGBP1	1.363	0.999	1.493	1.190	1.408	1.092	1.226	1.252
Q9NZ63	Uncharacterized protein C9orf78 OS=Homo sapiens GN=C9orf78	1.787	1.551	1.780	1.322	1.594	1.431	1.403	1.635
Q5XKP0	Protein QIL1 OS=Homo sapiens GN=QIL1 PE=1 SV=1 - [QIL1_H	1.351	1.534	1.478	1.365	1.415	1.198	1.415	1.457
Q8NE86	Calcium uniporter protein, mitochondrial OS=Homo sapiens GN=I	2.249	1.768	1.748	1.961	1.090	1.148	1.370	1.607
Q9UNP9	Peptidyl-prolyl cis-trans isomerase E OS=Homo sapiens GN=PPIE	1.366	1.404	1.120	0.970	1.438	1.259	1.324	1.113
A0A024R248	Chromosome 9 open reading frame 89, isoform CRA_b OS=Homo	1.136	1.380	1.158	1.038	1.021	1.000	1.200	1.117
P51553	Isocitrate dehydrogenase [NAD] subunit gamma, mitochondrial O	1.159	1.317	1.131	1.067	1.068	1.250	1.374	1.083
O95295	SNARE-associated protein Snapin OS=Homo sapiens GN=SNAPIN	1.438	1.110	1.103	0.924	1.155	1.285	1.547	1.023
Q9Y2U8	Inner nuclear membrane protein Man1 OS=Homo sapiens GN=LE	1.428	1.645	1.276	1.049	1.110	1.272	1.453	1.226
P36871	Phosphoglucomutase-1 OS=Homo sapiens GN=PGM1 PE=1 SV=3	1.265	1.021	1.372	1.160	1.108	0.997	1.191	1.137
Q86XL3	Ankyrin repeat and LEM domain-containing protein 2 OS=Homo s	1.306	1.465	1.121	0.972	1.154	1.303	1.344	1.098
O95716	Ras-related protein Rab-3D OS=Homo sapiens GN=RAB3D PE=1	3.939	3.776	3.290	3.024	2.097	1.334	2.355	2.591
P63151	Serine/threonine-protein phosphatase 2A 55 kDa regulatory subu	1.216	0.940	1.466	1.043	1.087	1.118	1.320	1.205
A0A087X1L5	Transcription elongation factor A protein-like 1 OS=Homo sapiens	1.329	0.846	1.012	0.609	1.246	1.197	0.933	0.856
Q6I7N8	Lymphoid specific helicase variant9 OS=Homo sapiens GN=HELLS	1.561	1.911	1.611	1.153	1.325	1.379	1.816	1.182
Q6DKI1	60S ribosomal protein L7-like 1 OS=Homo sapiens GN=RPL7L1 P	1.752	2.453	1.419	1.169	1.767	1.780	1.785	1.188
O95101	Cytochrome c oxidase subunit 6A, mitochondrial OS=Homo sapie	0.934	1.003	0.909	1.016	0.560	0.971	0.637	0.866
G3V1L9	Tight junction protein 1 (Zona occludens 1), isoform CRA_a OS=H	1.174	1.301	0.947	0.740	1.110	1.152	1.209	0.976
B2R7E8	cDNA, FLJ93412, highly similar to Homo sapiens replication prote	1.394	1.774	2.258	2.183	1.464	1.499	1.584	1.908
Q6P1N9	Putative deoxyribonuclease TATDN1 OS=Homo sapiens GN=TATD	1.522	1.160	1.389	1.207	1.230	1.072	1.306	1.295
Q9BUL9	Ribonuclease P protein subunit p25 OS=Homo sapiens GN=RPP2	1.504	2.017	1.447	0.935	1.566	1.636	1.931	1.602
D6RAD4	Cyclin-dependent kinase 7 OS=Homo sapiens GN=CDK7 PE=1 SV	1.695	1.857	1.608	1.604	1.809	1.360	1.723	1.808
O95453	Poly(A)-specific ribonuclease PARN OS=Homo sapiens GN=PARN	1.616	1.634	1.317	1.005	1.551	1.339	1.540	1.272
Q7L1Q6	Basic leucine zipper and W2 domain-containing protein 1 OS=Hor	1.490	1.178	1.275	0.958	1.370	1.071	1.218	1.047
P06730	Eukaryotic translation initiation factor 4E OS=Homo sapiens GN=	1.517	1.411	1.363	1.017	1.493	1.216	1.353	1.123
Q6ZSJ8	Uncharacterized protein C1orf122 OS=Homo sapiens GN=C1orf12				1.434	0.516	0.977		1.518
E9PGT1	Translin OS=Homo sapiens GN=TSN PE=1 SV=1 - [E9PGT1_HUM	1.522	1.152	1.528	1.213	1.288	1.069	1.494	1.424
B4DM52	DNA ligase OS=Homo sapiens PE=2 SV=1 - [B4DM52_HUMAN]	1.142	1.106	1.063	0.915	1.204	1.189	1.055	0.953
Q7Z4W1	L-xylulose reductase OS=Homo sapiens GN=DCXR PE=1 SV=2 - [	1.408	1.455	1.407	1.684	1.161	1.287	1.635	1.770

P10644	cAMP-dependent protein kinase type I-alpha regulatory subunit O	1.218	1.095	1.044	0.836	1.186	1.141	1.106	0.891
Q9H0L4	Cleavage stimulation factor subunit 2 tau variant OS=Homo sapie	2.037	2.200	1.570	1.194	1.855	1.512	1.740	1.642
O14744	Protein arginine N-methyltransferase 5 OS=Homo sapiens GN=PR	1.726	1.128	1.421	1.137	1.516	1.097	1.376	1.367
Q92797	Symplekin OS=Homo sapiens GN=SYMPK PE=1 SV=2 - [SYMPK_	1.461	1.622	1.351	1.008	1.397	1.310	1.398	1.275
A0A087WYF7	MICOS complex subunit MIC27 OS=Homo sapiens GN=APOOL PE	1.026	1.299	0.949	1.069	0.836	1.126	1.133	1.119
Q5JVZ5	Engulfment and cell motility protein 2 OS=Homo sapiens GN=ELN	1.528	1.188	1.338	0.998	1.316	1.075	1.149	1.158
Q15155	Nodal modulator 1 OS=Homo sapiens GN=NOMO1 PE=1 SV=5 -	0.877	1.730	1.300	1.042	1.278	1.311	1.783	1.376
P23381	Tryptophan--tRNA ligase, cytoplasmic OS=Homo sapiens GN=WA	2.512	1.506	2.249	1.742	1.511	1.169	2.190	2.131
O75569	Interferon-inducible double-stranded RNA-dependent protein kina	1.254	1.060	1.373	0.975	1.265	1.158	1.236	1.030
C9JE12	Transmembrane and ubiquitin-like domain-containing protein 1 (F	1.554	1.452	1.430	1.234	1.295	1.237	1.609	1.328
B3KMR5	cDNA FLJ12434 fis, clone NT2RM1000037, highly similar to Homo	1.735	2.083	1.674	1.336	1.500	1.355	1.804	1.386
P82663	28S ribosomal protein S25, mitochondrial OS=Homo sapiens GN=	1.242	1.411	1.170	1.368	0.958	1.233	1.352	1.345
Q15061	WD repeat-containing protein 43 OS=Homo sapiens GN=WDR43	1.478	1.872	1.456	1.489	1.631	1.431	1.657	1.684
Q10713	Mitochondrial-processing peptidase subunit alpha OS=Homo sapi	1.656	1.697	1.648	1.451	1.182	1.218	1.597	1.601
B4DNR3	cDNA FLJ52710, highly similar to Abhydrolase domain-containing	1.158	0.872	1.305	1.052	0.916	0.943	1.163	1.064
Q5JPB8	Putative uncharacterized protein DKFZp686O1117 OS=Homo sap	1.159	0.785	1.166	0.970	1.090	0.918	1.033	1.016
P11233	Ras-related protein Ral-A OS=Homo sapiens GN=RALA PE=1 SV=	1.184	1.236	1.214	1.271	0.945	1.288	1.195	1.385
A8K7S0	cDNA FLJ75478, highly similar to Homo sapiens splicing factor 4 (	1.417	1.642	1.234	1.116	1.356	1.426	1.537	1.357
Q53F02	Cleavage and polyadenylation specific factor 3, 73kDa variant (Fr	1.412	2.071	1.729	1.509	1.610	1.280	1.680	1.678
O96005	Cleft lip and palate transmembrane protein 1 OS=Homo sapiens (	1.541	1.530	1.268	1.231	1.177	1.274	1.610	1.185
B7Z6U8	cDNA FLJ53665, highly similar to Four and a half LIM domains pr	7.389	5.788	8.445	6.816	2.389	1.194	2.333	4.085
Q5SSJ5	Heterochromatin protein 1-binding protein 3 OS=Homo sapiens G	1.491	1.837	1.190	1.195	1.182	1.147	1.177	1.097
O75911	Short-chain dehydrogenase/reductase 3 OS=Homo sapiens GN=D	1.832	1.505	1.308	1.121	1.592	1.020	1.563	1.178
Q9UBU9	Nuclear RNA export factor 1 OS=Homo sapiens GN=NXF1 PE=1 S	1.522	1.907	1.507	1.370	1.514	1.394	1.821	1.503
D3DR37	Centrosomal protein 55kDa, isoform CRA_a OS=Homo sapiens GI	1.172	0.917	0.792	0.584	1.029	1.020	1.062	0.724
Q99797	Mitochondrial intermediate peptidase OS=Homo sapiens GN=MIP	1.201	1.282	0.992	1.051	1.017	1.043	1.248	1.112
P11498	Pyruvate carboxylase, mitochondrial OS=Homo sapiens GN=PC P	1.347	1.289	1.132	1.141	1.060	1.185	1.197	1.202
P53992	Protein transport protein Sec24C OS=Homo sapiens GN=SEC24C	1.323	1.055	1.115	0.895	1.413	1.187	1.190	1.100
Q8NF45	FLJ00353 protein (Fragment) OS=Homo sapiens GN=FLJ00353 P	1.338	1.490	1.211	1.159	1.349	1.347	1.355	1.343
Q9NXW2	DnaJ homolog subfamily B member 12 OS=Homo sapiens GN=DI	1.416	1.626	1.501	1.409	1.170	1.208	1.512	1.264
Q00796	Sorbitol dehydrogenase OS=Homo sapiens GN=SORD PE=1 SV=4	1.152	0.906	1.265	1.136	1.361	0.981	1.483	1.085
Q13724	Mannosyl-oligosaccharide glucosidase OS=Homo sapiens GN=MO	1.167	1.138	0.949	0.972	0.980	1.146	1.135	0.970
O00764	Pyridoxal kinase OS=Homo sapiens GN=PDXK PE=1 SV=1 - [PDX	0.780	0.619	0.858	0.624	0.897	1.076	0.914	0.684
P08237	ATP-dependent 6-phosphofructokinase, muscle type OS=Homo sa	1.272	0.825	1.411	1.188	1.198	0.882	1.317	1.110
P50502	Hsc70-interacting protein OS=Homo sapiens GN=ST13 PE=1 SV=	1.353	0.906	1.156	0.936	1.207	1.011	1.157	1.002
Q9BPX5	Actin-related protein 2/3 complex subunit 5-like protein OS=Hom	1.168	1.537	1.117	0.982	1.360	1.307	1.181	0.871
Q8WWY3	U4/U6 small nuclear ribonucleoprotein Prp31 OS=Homo sapiens (	1.229	1.286	0.957	0.774	1.360	1.248	1.161	1.031
K7EIG1	Clustered mitochondria protein homolog (Fragment) OS=Homo sa	8.946	7.336	10.996	6.840	3.366	1.388	4.215	4.692
P35527	Keratin, type I cytoskeletal 9 OS=Homo sapiens GN=KRT9 PE=1	1.714	0.809	1.907	1.553	0.677	0.621	4.182	0.537
Q6PK04	Coiled-coil domain-containing protein 137 OS=Homo sapiens GN=	1.543	2.182	1.410	1.008	1.424	1.559	1.418	1.366

E7ETT4	Propionyl-CoA carboxylase beta chain, mitochondrial OS=Homo s	1.262	1.036	1.234	1.080	1.007	1.195	1.288	1.213
Q8NC56	LEM domain-containing protein 2 OS=Homo sapiens GN=LEMD2	1.050	1.271	0.917	0.882	1.001	1.025	1.077	0.903
O75616	GTPase Era, mitochondrial OS=Homo sapiens GN=ERAL1 PE=1 S	1.156	1.363	1.105	0.906	1.099	1.311	1.476	1.165
P14735	Insulin-degrading enzyme OS=Homo sapiens GN=IDE PE=1 SV=	1.400	1.032	1.370	1.059	1.168	1.071	1.220	1.207
P14649	Myosin light chain 6B OS=Homo sapiens GN=MYL6B PE=1 SV=1	1.224	0.817	0.963	0.741	1.026	0.897	0.955	0.971
Q9H204	Mediator of RNA polymerase II transcription subunit 28 OS=Hom	1.355	1.806	1.343	1.221	1.472	1.339	1.226	1.176
B3KN49	cDNA FLJ13562 fis, clone PLACE1008080, highly similar to Homo	1.539	1.303	1.059	0.528	1.282	1.154	0.993	0.892
P51116	Fragile X mental retardation syndrome-related protein 2 OS=Hom	1.848	1.554	1.260	1.037	1.708	1.203	1.343	1.202
E7ESY4	Metastasis-associated protein MTA1 OS=Homo sapiens GN=MTA1	1.133	1.282	0.930	0.767	1.143	1.308	1.329	1.085
B2R7M3	cDNA, FLJ93510, highly similar to Homo sapiens JTV1 gene (JTV1	2.397	2.489	2.549	1.671	1.719	1.156	2.039	2.143
Q6P2C8	Mediator of RNA polymerase II transcription subunit 27 OS=Hom	1.468	1.386	1.072	0.639	1.210	1.194	1.052	0.983
Q9Y320	Thioredoxin-related transmembrane protein 2 OS=Homo sapiens	2.547	2.042	2.199	1.680	1.612	1.447	1.875	1.528
Q14684	Ribosomal RNA processing protein 1 homolog B OS=Homo sapier	1.501	1.731	1.334	1.061	1.719	1.417	1.703	1.569
Q15424	Scaffold attachment factor B1 OS=Homo sapiens GN=SAFB PE=1	1.331	1.445	1.138	0.968	1.290	1.380	1.583	1.346
Q59EI3	Branched chain keto acid dehydrogenase E1, alpha polypeptide v	0.932	1.121	1.127	1.232	0.868	1.047	0.947	0.977
P30520	Adenylosuccinate synthetase isozyme 2 OS=Homo sapiens GN=A	1.336	0.944	1.343	0.995	1.215	1.002	1.229	1.086
C9JVN9	L-2-hydroxyglutarate dehydrogenase, mitochondrial OS=Homo sa	1.222	1.316	1.127	1.237	1.091	1.233	1.325	1.304
Q01105	Protein SET OS=Homo sapiens GN=SET PE=1 SV=3 - [SET_HUM	1.178	1.217	1.428	0.978	1.396	1.133	1.237	1.120
Q96ER9	Coiled-coil domain-containing protein 51 OS=Homo sapiens GN=(	1.573	1.689	1.530	1.360	1.323	1.241	1.515	1.424
H0YAL7	Eukaryotic translation elongation factor 1 epsilon-1 (Fragment) O	5.797	5.594	5.191	4.339	3.616	1.437	5.305	4.602
B4DEI4	cDNA FLJ54595, highly similar to Golgi reassembly-stacking prote	1.562	1.198	1.370	1.227	1.917	1.279	1.972	1.399
Q9Y285	Phenylalanine--tRNA ligase alpha subunit OS=Homo sapiens GN=	1.333	1.067	1.183	0.764	1.166	1.094	1.110	0.868
P20338	Ras-related protein Rab-4A OS=Homo sapiens GN=RAB4A PE=1	1.150	1.188	1.220	1.212	0.960	1.013	1.282	1.221
G5E9G6	E3 ubiquitin-protein ligase TRIP12 OS=Homo sapiens GN=TRIP12	1.920		1.200		1.976	1.503		
F8W0Q9	Periphilin-1 OS=Homo sapiens GN=PPHLN1 PE=1 SV=1 - [F8W0(	1.130	1.119	0.868	0.862	1.098	1.176	1.157	1.164
B4DWG1	cDNA FLJ55643, highly similar to SEC23-interacting protein OS=H	1.464	1.005	1.347	1.139	1.249	1.157	1.277	1.168
Q05048	Cleavage stimulation factor subunit 1 OS=Homo sapiens GN=CST	1.340	1.492	1.037	0.792	1.385	1.343	1.395	1.036
G3V1D4	Lin-7 homolog C (C. elegans), isoform CRA_b OS=Homo sapiens	1.253	1.668	1.283	1.287	1.540	1.307	1.392	1.533
Q5QJE6	Deoxynucleotidyltransferase terminal-interacting protein 2 OS=Ho	1.288	1.614	1.096	0.835	1.317	1.407	1.262	0.892
O43237	Cytoplasmic dynein 1 light intermediate chain 2 OS=Homo sapier	1.942	1.064	1.756	1.212	1.578	1.119	1.367	1.321
P33552	Cyclin-dependent kinases regulatory subunit 2 OS=Homo sapiens	3.306	2.431	2.267	1.605	3.030	2.288	3.846	3.708
O75976	Carboxypeptidase D OS=Homo sapiens GN=CPD PE=1 SV=2 - [C	1.303	1.517	1.513	1.537	1.074	1.252	1.408	1.287
Q9BYC9	39S ribosomal protein L20, mitochondrial OS=Homo sapiens GN=	1.096	1.357	1.086	1.177	0.846	1.189	1.233	1.263
Q9H3N1	Thioredoxin-related transmembrane protein 1 OS=Homo sapiens	1.313	1.213	1.193	1.098	1.045	1.336	1.329	1.175
Q9GZT9	Egl nine homolog 1 OS=Homo sapiens GN=EGLN1 PE=1 SV=1 -	1.534	0.984	1.201	0.639	1.077	0.952	1.087	0.673
Q12789	General transcription factor 3C polypeptide 1 OS=Homo sapiens (	1.505	1.748	1.176	1.012	1.411	1.421	1.551	1.696
Q9UQN3	Charged multivesicular body protein 2b OS=Homo sapiens GN=C	1.225	0.766	1.047	0.764	0.995	0.907	1.038	0.799
Q2M2R1	ANAPC7 protein (Fragment) OS=Homo sapiens GN=ANAPC7 PE=	1.430	1.375	1.197	0.885	1.289	1.344	1.430	1.133
O75152	Zinc finger CCCH domain-containing protein 11A OS=Homo sapie	1.252	1.656	1.221	1.111	0.990	1.309	1.554	1.452
Q9P000	COMM domain-containing protein 9 OS=Homo sapiens GN=COMI	1.930	1.835	2.463	1.910	2.168	1.481	2.578	1.904

B4DLM8	cDNA FLJ56105, highly similar to Nuclear valosin-containing prote	1.927	2.096	1.782	1.675	1.769	1.573	2.127	2.069
I3NI44	TOM1-like protein 1 OS=Homo sapiens GN=TOM1L1 PE=1 SV=1	1.856	1.022	1.694	1.511	1.630	1.069	1.534	1.632
Q6ZRP7	Sulfhydryl oxidase 2 OS=Homo sapiens GN=QSOX2 PE=1 SV=3 -	2.083	2.022	1.725	1.736	1.229	1.326	1.815	1.615
B7Z213	cDNA FLJ50130, highly similar to Heterogeneous nuclear ribonucl	1.381	1.372	1.034	0.873	0.950	1.078	0.998	0.765
Q6QN92	Mitochondrial glycine cleavage system H-protein (Fragment) OS=	1.663	1.570	1.301	1.234	0.953	1.220	1.138	1.335
Q99426	Tubulin-folding cofactor B OS=Homo sapiens GN=TBCB PE=1 SV	1.437	1.005	1.412	1.312	1.417	1.037	1.329	1.226
B2RA56	Nicalin OS=Homo sapiens PE=2 SV=1 - [B2RA56_HUMAN]	1.437	1.416	1.304	1.180	1.067	1.337	1.320	1.214
B2RE59	cDNA, FLJ93148, highly similar to Homo sapiens RCD1 required f	1.857	2.044	1.918	1.438	1.763	1.518	1.854	1.333
A0PJ79	MRPL1 protein (Fragment) OS=Homo sapiens GN=MRPL1 PE=2 S	1.290	1.478	1.127	1.195	0.979	1.112	1.259	1.199
O43805	Sjoegren syndrome nuclear autoantigen 1 OS=Homo sapiens GN	1.458	1.213	1.947	1.575	1.403	1.241	1.381	1.331
Q5TFE4	5'-nucleotidase domain-containing protein 1 OS=Homo sapiens G	1.464	0.963	1.392	1.150	1.253	1.055	1.325	1.226
B2RWN5	HEAT repeat containing 1 OS=Homo sapiens GN=HEATR1 PE=2 S	1.854	2.093	1.828	1.739	1.802	1.535	1.933	1.807
Q9NR50	Translation initiation factor eIF-2B subunit gamma OS=Homo sap	1.730	1.318	1.708	1.387	1.478	1.141	1.653	1.593
B3KRC6	cDNA FLJ34004 fis, clone FCBBF1000232, highly similar to Cytoch	1.170	1.137	0.803	0.664	0.927	1.063	1.113	0.763
F5H8D7	Forkhead box protein H1 OS=Homo sapiens GN=FOXH1 PE=4 SV	1.149	1.294	1.251	1.125	1.111	1.158	1.173	1.260
Q16576	Histone-binding protein RBBP7 OS=Homo sapiens GN=RBBP7 PE	1.260	1.158	0.938	0.788	1.225	1.275	1.307	1.046
D6RGI3	Septin 11, isoform CRA_b OS=Homo sapiens GN=SEPT11 PE=1 S	1.398	0.989	1.284	0.904	1.297	1.050	1.185	1.160
G3XAG1	Zinc finger protein 512 OS=Homo sapiens GN=ZNF512 PE=4 SV=	1.595	2.048	1.348	1.286	1.002	1.097	1.297	1.746
Q5U5X0	Complex III assembly factor LYRM7 OS=Homo sapiens GN=LYRM	1.441	1.298	0.943	1.074	1.270	1.155	1.465	1.246
C9J0I9	Nuclear-interacting partner of ALK OS=Homo sapiens GN=ZC3HC	1.707	1.989	1.621	2.458	1.700	1.350	1.659	1.782
B3KPI5	cDNA FLJ31840 fis, clone NT2RP7000109, highly similar to Tudor	1.235	1.423	1.123	1.189	1.036	1.182	1.400	1.314
J3QRZ6	Homeobox protein MOX-1 (Fragment) OS=Homo sapiens GN=ME	1.030	0.795	0.851	0.767	1.204	0.945	0.911	0.834
Q15554	Telomeric repeat-binding factor 2 OS=Homo sapiens GN=TERF2 I	1.176	1.548	1.119	0.841	1.129	1.321	1.257	1.275
I7GPQ7	cDNA FLJ75793 OS=Homo sapiens PE=2 SV=1 - [I7GPQ7_HUMA	1.331	1.152	1.053	0.829	1.322	1.221	0.998	0.866
Q2TNB3	Cell migration-inducing protein 22 OS=Homo sapiens PE=2 SV=1	1.099	0.608	1.295	0.927	1.090	0.982	1.056	0.907
Q2NLD4	PURA protein (Fragment) OS=Homo sapiens GN=PURA PE=2 SV=	1.901	1.945	1.669	1.245	1.803	1.286	1.707	1.280
Q9UBP6	tRNA (guanine-N(7)-)-methyltransferase OS=Homo sapiens GN=I	2.694	2.232	2.561	1.620	1.507	1.176	1.326	1.139
A8K005	cDNA FLJ77896, highly similar to Homo sapiens Ras homolog enr	1.729	1.198	1.683	1.242	1.288	1.154	1.294	1.580
Q9H9B4	Sideroflexin-1 OS=Homo sapiens GN=SFXN1 PE=1 SV=4 - [SFXN	1.360	1.464	1.150	1.221	1.010	1.164	1.462	1.378
Q9UBB5	Methyl-CpG-binding domain protein 2 OS=Homo sapiens GN=MB	1.204	1.707	1.113	0.927	1.043	1.098	1.362	1.270
Q8IY81	pre-rRNA processing protein FTSJ3 OS=Homo sapiens GN=FTSJ3	1.498	1.575	1.253	1.076	1.479	1.359	1.488	1.255
Q9NYB0	Telomeric repeat-binding factor 2-interacting protein 1 OS=Homo	1.416	1.698	1.379	1.065	1.419	1.508	1.671	1.704
H7C1U8	Apolipoprotein O (Fragment) OS=Homo sapiens GN=APOO PE=1	1.849	2.036	1.856	1.553	1.390	1.205	1.835	1.950
O43181	NADH dehydrogenase [ubiquinone] iron-sulfur protein 4, mitocho	1.764	1.492	1.548	1.473	1.062	0.957	1.273	1.300
P17655	Calpain-2 catalytic subunit OS=Homo sapiens GN=CAPN2 PE=1 S	1.584	0.880	1.300	0.932	1.311	1.013	1.198	1.031
O95400	CD2 antigen cytoplasmic tail-binding protein 2 OS=Homo sapiens	1.254	1.140	0.942	0.790	1.285	1.299	1.433	1.471
Q496C9	D-tyrosyl-tRNA(Tyr) deacylase OS=Homo sapiens GN=DTD1 PE=	3.352	2.794	3.528	3.238	2.307	1.247	2.524	2.799
B4DKP8	cDNA FLJ52317, highly similar to tRNA-dihydrouridine synthase 2-	1.224	1.121	1.111	0.816	1.174	1.185	1.068	1.031
P48507	Glutamate--cysteine ligase regulatory subunit OS=Homo sapiens	1.546	1.006	1.244	1.004	1.232	1.109	1.168	0.976
A8K4D5	Kynureninase OS=Homo sapiens GN=KYNU PE=2 SV=1 - [A8K4D	1.873	1.156	1.794	1.336	1.478	1.033	1.555	1.253

P56556	NADH dehydrogenase [ubiquinone] 1 alpha subcomplex subunit 6	0.977	1.065	0.857	0.893	0.919	1.143	1.043	0.937
M0R3D4	Prenylated Rab acceptor protein 1 OS=Homo sapiens GN=RABAC	2.440	2.719	1.739	1.204	1.376	1.167	1.519	1.813
A8K3B6	cDNA FLJ78579, highly similar to Homo sapiens c-src tyrosine kin	1.245	0.896	1.273	0.998	1.146	0.954	1.243	1.079
A1L188	Uncharacterized protein C17orf89 OS=Homo sapiens GN=C17orf8	3.950	3.274	2.744	2.765	1.722	1.629	2.130	2.209
A0A024RAC0	Leucine zipper protein 1, isoform CRA_a OS=Homo sapiens GN=L	1.696	1.601	1.388	0.966	1.404	1.253	1.279	1.122
Q9BZG1	Ras-related protein Rab-34 OS=Homo sapiens GN=RAB34 PE=1 S	1.707	1.828	1.487	1.317	1.512	1.196	1.532	1.389
J3KRC4	5'(3')-deoxyribonucleotidase, cytosolic type OS=Homo sapiens GN	1.897	1.044	1.372	1.380	0.989	1.077	1.550	1.358
A0A087WZH7	Myristoylated alanine-rich C-kinase substrate OS=Homo sapiens G	1.736	1.011	1.139	0.935	1.296	0.897	1.110	0.949
E9PJ81	UBX domain-containing protein 1 (Fragment) OS=Homo sapiens G	1.184	0.867	1.070	0.783	1.020	1.064	0.928	0.980
Q9Y383	Putative RNA-binding protein Luc7-like 2 OS=Homo sapiens GN=L	1.464	1.394	1.377	0.938	1.392	1.158	1.351	1.029
H0YLF3	Beta-2-microglobulin form pI 5.3 (Fragment) OS=Homo sapiens G	2.046	1.681	1.485	1.694	1.319	1.585	1.848	1.829
Q6NZY4	Zinc finger CCHC domain-containing protein 8 OS=Homo sapiens	1.318	1.245	1.002	0.753	1.222	1.374	1.160	0.921
P59768	Guanine nucleotide-binding protein G(I)/G(S)/G(O) subunit gamm		1.003			0.951	1.362	0.999	0.812
Q5SWX8	Protein odr-4 homolog OS=Homo sapiens GN=ODR4 PE=2 SV=1	1.911	1.827	1.442	1.304	1.344	1.292	1.613	1.535
Q93096	Protein tyrosine phosphatase type IVA 1 OS=Homo sapiens GN=P	2.585	2.688	1.733	1.347	2.084	1.483	1.882	1.686
J3KPP0	39S ribosomal protein L42, mitochondrial OS=Homo sapiens GN=	4.649	4.398	3.490	3.713	2.904	1.540	4.474	3.993
B7Z653	cDNA FLJ61657, highly similar to Band 4.1-like protein 1 OS=Hom	1.537	1.355	1.146	0.819	1.513	1.071	1.236	1.080
E7EQT4	Apoptotic chromatin condensation inducer in the nucleus OS=Hor	1.695	1.535	1.446	1.262	1.541	1.333	1.542	1.546
D6R613	ATP synthase protein 8 OS=Homo sapiens GN=ATP8 PE=3 SV=1	4.227	5.287	4.074	4.456	2.402	1.227	3.966	4.652
B2RC50	cDNA, FLJ95853, highly similar to Homo sapiens exosome compo	1.531	2.253	1.635	1.691	1.701	1.323	1.654	1.569
Q9BXY0	Protein MAK16 homolog OS=Homo sapiens GN=MAK16 PE=1 SV	1.754	1.690	1.250	1.102	1.565	1.384	1.544	1.377
O00273	DNA fragmentation factor subunit alpha OS=Homo sapiens GN=D	1.491	1.077	1.504	1.524	1.436	1.211	1.422	1.315
Q16543	Hsp90 co-chaperone Cdc37 OS=Homo sapiens GN=CDC37 PE=1	1.657	1.304	1.395	1.153	1.491	1.044	1.618	1.487
A0A024R837	Haloacid dehalogenase-like hydrolase domain containing 3, isofor	1.343	1.370	1.223	1.309	1.105	1.158	1.270	1.428
Q15056	Eukaryotic translation initiation factor 4H OS=Homo sapiens GN=	1.211	1.079	1.513	1.162	1.136	0.904	1.162	0.990
Q9NY12	H/ACA ribonucleoprotein complex subunit 1 OS=Homo sapiens GI	2.277	2.466	1.980	1.913	2.074	1.568	2.168	2.406
H9E7F7	Cytochrome c oxidase subunit 2 (Fragment) OS=Homo sapiens G	0.975	1.123	1.015	0.988	0.535	0.958	1.090	1.217
A0A087X0I4	Coatomer subunit epsilon OS=Homo sapiens GN=COPE PE=4 SV	1.705	1.188	1.569	1.034	1.778	1.195	1.341	1.060
B4DGF8	cDNA FLJ57877, highly similar to Cleavage and polyadenylation sp	1.288	1.504	1.001	0.935	1.309	1.371	1.290	1.179
E7EVH7	Kinesin light chain 1 OS=Homo sapiens GN=KLC1 PE=4 SV=1 - [	1.635	1.259	1.394	1.062	1.388	1.051	1.475	1.307
Q9UPT5	Exocyst complex component 7 OS=Homo sapiens GN=EXOC7 PE	1.401	0.989	1.414	1.020	1.292	1.180	1.302	1.095
B2RAH5	Protein phosphatase 1 regulatory subunit 12 OS=Homo sapiens F	1.805	1.954	1.373	1.014	1.613	1.270	1.310	1.167
Q96GM5	SWI/SNF-related matrix-associated actin-dependent regulator of c	1.497	1.584	0.923	0.702	1.358	1.312	1.170	1.052
B2RBE5	cDNA, FLJ95468, highly similar to Homo sapiens transcriptional co	1.568	1.086	1.347	0.989	1.359	1.188	1.413	1.004
P62861	40S ribosomal protein S30 OS=Homo sapiens GN=FAU PE=1 SV=	1.696	1.533	1.570	0.993	1.500	1.250	1.343	1.215
O75821	Eukaryotic translation initiation factor 3 subunit G OS=Homo sapi	0.929	0.812	0.838	0.625	1.075	1.164	0.896	0.739
Q9NZ01	Very-long-chain enoyl-CoA reductase OS=Homo sapiens GN=TEC	2.324	2.425	2.049	1.886	1.824	1.485	2.299	1.952
D6R9Z7	Cytochrome c oxidase subunit 7C, mitochondrial OS=Homo sapie	1.293	1.468	1.196	1.217	0.781	1.010	1.132	1.155
F8VYY9	5'-AMP-activated protein kinase subunit gamma-1 OS=Homo sapi	1.543	0.989	1.350	1.002	1.294	0.991	1.321	1.131
P62745	Rho-related GTP-binding protein RhoB OS=Homo sapiens GN=RH	2.005	2.444	1.139	1.056	2.117	1.847	1.730	1.238

C9JYM0	Ribonuclease P protein subunit p20 (Fragment) OS=Homo sapien	1.055	1.006	0.985	0.664	1.098	1.294	0.980	0.744
B4DHE8	Protein turtle homolog B OS=Homo sapiens GN=IGSF9B PE=2 SV	1.301	1.308	1.013	0.884	1.292	1.143	1.162	1.187
Q8TC12	Retinol dehydrogenase 11 OS=Homo sapiens GN=RDH11 PE=1 S	1.345	1.202	0.963	1.089	1.153	1.183	1.204	1.057
Q5T1C6	Acyl-coenzyme A thioesterase THEM4 OS=Homo sapiens GN=TH	1.667	2.080	1.745	1.685	1.295	1.506	1.959	2.243
Q53HS1	Achalasia, adrenocortical insufficiency, alacrimia (Allgrove, triple-A	1.369	1.404	1.302	1.295	1.369	1.332	1.434	1.578
F5H442	Tumor susceptibility gene 101 protein OS=Homo sapiens GN=TS(	1.829	1.256	1.432	1.193	1.595	1.140	1.498	1.302
Q9Y2W1	Thyroid hormone receptor-associated protein 3 OS=Homo sapien	1.393	1.406	1.158	1.131	1.272	1.260	1.196	1.366
Q9P035	Very-long-chain (3R)-3-hydroxyacyl-CoA dehydratase 3 OS=Homo	1.458	1.421	1.445	1.453	1.166	1.372	1.709	1.479
I3L2B0	Clustered mitochondria protein homolog (Fragment) OS=Homo sa	1.598	1.414	1.860	1.374	1.571	1.195	1.596	1.361
O43491	Band 4.1-like protein 2 OS=Homo sapiens GN=EPB41L2 PE=1 SV	1.361	1.420	1.081	0.958	1.309	1.107	1.136	1.322
Q5JQ44	Putative uncharacterized protein DKFZp547A0616 (Fragment) OS	1.242	1.447	1.027	1.060	1.244	1.091	0.965	1.201
Q9NX62	Inositol monophosphatase 3 OS=Homo sapiens GN=IMPAD1 PE=	1.327	1.369	1.514	1.594	1.093	1.423	1.435	1.230
Q9H9L3	Interferon-stimulated 20 kDa exonuclease-like 2 OS=Homo sapie	1.564	2.075	1.529	1.251	1.270	1.563	1.592	1.298
Q8IY37	Probable ATP-dependent RNA helicase DHX37 OS=Homo sapiens	1.481	1.763	1.326	1.043	1.375	1.393	1.445	1.253
Q92785	Zinc finger protein ubi-d4 OS=Homo sapiens GN=DPF2 PE=1 SV=	2.772	2.492	1.538	1.945	2.648	1.428	2.557	1.509
Q16864	V-type proton ATPase subunit F OS=Homo sapiens GN=ATP6V1F	3.825	2.859	3.282	3.083	3.204	1.850	3.600	3.788
O14980	Exportin-1 OS=Homo sapiens GN=XPO1 PE=1 SV=1 - [XPO1_HU	1.531	1.284	1.672	1.375	1.649	1.262	1.518	1.485
Q59FM5	Guanine nucleotide-binding protein G, alpha subunit variant (Frag	1.165	1.679	1.267	1.196	1.013	1.263	1.379	1.351
Q9Y3C6	Peptidyl-prolyl cis-trans isomerase-like 1 OS=Homo sapiens GN=I	1.483	1.350	1.229	0.995	1.415	1.236	1.324	1.090
B4DMD3	cDNA FLJ58174, highly similar to WW domain-binding protein 11	1.372	1.388	1.009	0.816	1.405	1.278	1.069	0.959
P08754	Guanine nucleotide-binding protein G(k) subunit alpha OS=Homo	2.607	3.019	2.160	2.487	2.297	1.438	2.247	2.526
Q13618	Cullin-3 OS=Homo sapiens GN=CUL3 PE=1 SV=2 - [CUL3_HUMA	1.417	1.218	1.150	0.905	1.365	1.111	1.196	1.006
Q4G0N4	NAD kinase 2, mitochondrial OS=Homo sapiens GN=NADK2 PE=1	1.626	1.663	1.459	1.387	1.169	1.408	1.560	1.520
B1AJY5	26S proteasome non-ATPase regulatory subunit 10 OS=Homo sap	5.348	3.017	8.648	6.930	4.202	1.258	4.394	3.811
B3KUZ7	cDNA FLJ40986 fis, clone UTERU2014898, highly similar to Vacuo	1.595	1.215	1.554	1.313	1.475	1.340	1.800	1.598
Q9NX20	39S ribosomal protein L16, mitochondrial OS=Homo sapiens GN=	1.252	1.491	1.080	0.928	0.865	1.089	1.159	1.055
Q9P2I0	Cleavage and polyadenylation specificity factor subunit 2 OS=Hor	1.631	2.120	1.767	1.422	1.635	1.606	1.733	1.719
Q9NPD3	Exosome complex component RRP41 OS=Homo sapiens GN=EXC	1.239	1.492	1.158	1.100	1.266	1.230	1.346	1.271
B3KXX5	Structural maintenance of chromosomes protein OS=Homo sapie	1.341	1.015	1.036	0.734	1.147	1.104	1.103	0.890
Q8IX12	Cell division cycle and apoptosis regulator protein 1 OS=Homo sa	1.589	1.636	1.122	0.790	1.314	1.346	1.220	0.981
P49247	Ribose-5-phosphate isomerase OS=Homo sapiens GN=RPIA PE=	3.523	2.493	2.862	2.001	1.315	1.365	1.581	1.934
P50416	Carnitine O-palmitoyltransferase 1, liver isoform OS=Homo sapier	2.248	2.148	1.980	1.976	1.255	1.506	1.765	1.726
Q9H061	Transmembrane protein 126A OS=Homo sapiens GN=TMEM126A	1.798	1.842	1.821	1.800	1.003	1.130	1.889	1.837
Q9H3U1	Protein unc-45 homolog A OS=Homo sapiens GN=UNC45A PE=1	1.475	1.105	1.353	1.133	1.348	1.160	1.365	1.185
Q15291	Retinoblastoma-binding protein 5 OS=Homo sapiens GN=RBBP5	1.345	1.511	1.050	0.797	1.412	1.336	1.383	1.294
Q71RC2	La-related protein 4 OS=Homo sapiens GN=LARP4 PE=1 SV=3 -	1.427	1.636	1.349	0.991	1.425	1.296	1.391	1.057
O75792	Ribonuclease H2 subunit A OS=Homo sapiens GN=RNASEH2A PE	1.594	1.488	2.377	1.586	1.594	1.447	1.618	1.624
Q14978	Nucleolar and coiled-body phosphoprotein 1 OS=Homo sapiens G	1.436	1.718	1.333	1.538	1.172	1.275	1.327	1.755
B4DNL8	cDNA FLJ58851, highly similar to Galactosylgalactosylxylosyl prote	1.226	1.106	1.338	1.149	0.882	1.135	1.247	1.045
B7Z2R7	Acyl-CoA-binding domain-containing protein 5 OS=Homo sapiens	1.310	1.304	1.162	1.318	1.063	1.281	1.542	1.327

P28340	DNA polymerase delta catalytic subunit OS=Homo sapiens GN=P	1.120	1.044	1.134	0.823	1.137	1.041	0.939	0.793
Q53SY7	Putative uncharacterized protein CAD (Fragment) OS=Homo sapi	1.376	0.922	1.398	1.187	1.216	0.962	1.249	1.137
Q9UM54	Unconventional myosin-VI OS=Homo sapiens GN=MYO6 PE=1 S\	1.421	1.216	1.421	1.278	1.312	1.213	1.453	1.309
H7C0N4	Splicing factor 1 (Fragment) OS=Homo sapiens GN=SF1 PE=1 SV	1.583	1.749	1.115	0.780	1.530	1.400	1.379	0.924
B7Z8G2	cDNA FLJ58213, highly similar to FK506-binding protein 5 (EC 5.2	1.090	1.070	1.074	0.704	1.336	1.039	1.042	0.932
I6L9E8	Family with sequence similarity 98, member A OS=Homo sapiens	1.199	1.400	1.094	0.877	1.173	1.173	1.266	1.001
P40123	Adenylyl cyclase-associated protein 2 OS=Homo sapiens GN=CAF	1.121	0.782	1.073	0.814	1.126	0.892	0.956	0.899
Q12846	Syntaxin-4 OS=Homo sapiens GN=STX4 PE=1 SV=2 - [STX4_HU	0.854	1.141	0.953	1.120	0.935	1.246	1.126	0.935
P52948	Nuclear pore complex protein Nup98-Nup96 OS=Homo sapiens G	1.525	1.661	1.493	1.568	1.561	1.460	1.695	1.763
B4DEA6	cDNA FLJ56566, highly similar to Small glutamine-rich tetratricope	2.103	1.570	1.766	1.486	2.140	1.466	1.854	1.788
F5GZ97	WASH complex subunit CCDC53 OS=Homo sapiens GN=CCDC53	1.441	1.052	1.369	1.131	1.268	1.089	1.249	1.176
P50570	Dynamin-2 OS=Homo sapiens GN=DNM2 PE=1 SV=2 - [DYN2_H	1.613	1.015	1.381	1.147	1.340	0.985	1.159	1.039
O14925	Mitochondrial import inner membrane translocase subunit Tim23	1.168	1.369	1.043	0.929	0.908	1.112	1.210	1.063
Q53RD8	Putative uncharacterized protein LOC84524 (Fragment) OS=Hom	1.455	1.834	1.154	0.801	1.460	1.614	1.316	0.908
Q9Y394	Dehydrogenase/reductase SDR family member 7 OS=Homo sapie	1.274	1.311	1.434	1.197	1.157	1.162	1.562	1.181
B2RBI2	cDNA, FLJ95525, highly similar to Homo sapiens synapse associa	1.490	1.078	1.237	1.027	1.346	1.200	1.267	1.177
Q96GK7	Fumarylacetoacetate hydrolase domain-containing protein 2A OS	1.413	1.439	1.495	1.414	1.057	1.203	1.316	1.399
Q8WWM7	Ataxin-2-like protein OS=Homo sapiens GN=ATXN2L PE=1 SV=2	1.538	1.602	1.396	1.057	1.402	1.266	1.250	1.256
Q8IWJ2	GRIP and coiled-coil domain-containing protein 2 OS=Homo sapie	1.681	1.478	1.211	1.066	1.614	1.339	1.225	1.070
A8K4W7	cDNA FLJ76284, highly similar to Homo sapiens succinate-CoA lig	1.377	1.471	1.317	1.250	1.260	1.245	1.404	1.360
Q96EK6	Glucosamine 6-phosphate N-acetyltransferase OS=Homo sapiens	1.792	1.923	2.276	2.416	1.049	1.216	1.254	2.087
H7BXL1	Transmembrane protein 41A OS=Homo sapiens GN=TMEM41A P	1.515	1.838	1.469	1.503	1.188	1.261	1.560	1.413
Q5TG80	Voltage-gated potassium channel subunit beta-2 (Fragment) OS=	1.086	0.958	1.473	0.964	1.149	0.975	1.206	0.876
Q6UN15	Pre-mRNA 3'-end-processing factor FIP1 OS=Homo sapiens GN=	1.575	1.558	1.234	1.100	1.565	1.484	1.612	1.523
H0Y2W2	ATPase family AAA domain-containing protein 3A (Fragment) OS=						1.693		
P42126	Enoyl-CoA delta isomerase 1, mitochondrial OS=Homo sapiens G	1.357	1.476	1.285	1.565	1.009	1.248	1.251	1.297
L0R588	Alternative protein C11orf48 OS=Homo sapiens GN=C11orf48 PE	1.329	1.288	1.076	0.746	1.338	1.173	1.170	0.882
Q9H2U1	ATP-dependent RNA helicase DHX36 OS=Homo sapiens GN=DH	1.525	1.589	1.332	0.973	1.283	1.167	1.264	1.137
H7BXI1	Extended synaptotagmin-2 (Fragment) OS=Homo sapiens GN=E	1.282	1.580	1.227	1.243	1.145	1.242	1.326	1.226
C9JY28	LYR motif-containing protein 4 OS=Homo sapiens GN=LYRM4 PE	1.062	1.273	1.024	1.017	0.859	1.161	1.248	1.087
Q8TAT6	Nuclear protein localization protein 4 homolog OS=Homo sapiens	1.423	1.073	1.101	0.732	1.332	1.118	1.061	1.004
B3KML1	cDNA FLJ11308 fis, clone PLACE1010074, highly similar to Sortin	1.237	0.891	1.282	0.957	1.127	0.995	1.106	1.080
Q9NX58	Cell growth-regulating nucleolar protein OS=Homo sapiens GN=L	1.628	1.880	1.115	1.130	1.110	1.324	1.463	1.072
Q8IZ81	ELMO domain-containing protein 2 OS=Homo sapiens GN=ELMO	1.271	1.191	1.577	1.449	1.366	1.412	1.915	1.175
A0A024R7P3	ATPase type 13A1, isoform CRA_f OS=Homo sapiens GN=ATP13	1.522	1.459	1.316	1.213	1.129	1.184	1.469	1.174
P55795	Heterogeneous nuclear ribonucleoprotein H2 OS=Homo sapiens	1.227	1.252	1.011	0.711	1.302	1.162	1.100	0.869
H7C608	Geminin (Fragment) OS=Homo sapiens GN=GMNN PE=1 SV=1 -	1.723	3.376	3.136	1.679	1.672	2.913	3.637	2.317
O75348	V-type proton ATPase subunit G 1 OS=Homo sapiens GN=ATP6V	0.650	0.590	0.551	0.623	1.019	1.562	0.977	0.775
O43570	Carbonic anhydrase 12 OS=Homo sapiens GN=CA12 PE=1 SV=1	0.846	1.080	0.796	0.860	0.852	0.976	0.777	0.744
P57740	Nuclear pore complex protein Nup107 OS=Homo sapiens GN=NU	1.658	1.763	1.588	1.644	1.597	1.470	1.794	1.875

P52732	Kinesin-like protein KIF11 OS=Homo sapiens GN=KIF11 PE=1 SV	1.550	0.980	1.236	1.073	1.347	1.097	1.458	1.380
Q06787	Fragile X mental retardation protein 1 OS=Homo sapiens GN=FM	1.216	1.256	1.080	0.521	1.066	0.868	1.152	0.814
Q69YL0	Uncharacterized protein NCBP2-AS2 OS=Homo sapiens GN=NCBI		1.993				1.323		
Q9NVM9	Protein asunder homolog OS=Homo sapiens GN=ASUN PE=1 SV=	1.430	1.430	1.463	0.952	1.296	1.341	1.450	1.247
P62854	40S ribosomal protein S26 OS=Homo sapiens GN=RPS26 PE=1 S	1.460	1.277	1.158	0.810	1.363	1.176	1.141	1.008
Q9NRG0	Chromatin accessibility complex protein 1 OS=Homo sapiens GN=	1.536	1.448	1.633	1.111	0.978	1.385	1.595	1.799
Q9NRF9	DNA polymerase epsilon subunit 3 OS=Homo sapiens GN=POLE3	1.179	1.117	1.251	0.715	1.076	1.087	1.177	1.276
Q9NR12	PDZ and LIM domain protein 7 OS=Homo sapiens GN=PDLIM7 P	1.146	0.994	0.940	0.719	1.076	1.105	0.892	0.774
O14929	Histone acetyltransferase type B catalytic subunit OS=Homo sapi	1.110	0.941	1.045	0.833	1.209	1.098	1.087	0.893
Q9BRG1	Vacuolar protein-sorting-associated protein 25 OS=Homo sapiens	1.400	1.243	1.301	1.196	1.071	1.238	1.390	1.081
B2R921	cDNA, FLJ94171, highly similar to Homo sapiens solute carrier fa	1.490	1.550	1.475	1.497	1.046	1.352	1.532	1.444
Q9NW64	Pre-mRNA-splicing factor RBM22 OS=Homo sapiens GN=RBM22	1.458	1.429	1.322	0.998	1.331	1.484	1.261	0.915
B4DH12	cDNA FLJ60514, highly similar to Fructosamine-3-kinase (EC 2.7.	1.389	1.016	1.327	1.108	1.083	1.163	1.301	1.222
Q9HC36	rRNA methyltransferase 3, mitochondrial OS=Homo sapiens GN=	1.289	1.593	1.343	1.045	1.032	1.381	1.497	1.331
Q3ZCR7	NEIL2 protein (Fragment) OS=Homo sapiens GN=NEIL2 PE=2 SV	1.640	2.041	1.885	1.476	1.188	1.359	1.316	1.292
B4DII5	Importin subunit alpha OS=Homo sapiens PE=2 SV=1 - [B4DII5_	1.471	1.287	1.323	0.948	1.252	1.171	1.417	1.203
A6NHR9	Structural maintenance of chromosomes flexible hinge domain-co	1.293	1.396	1.043	0.802	1.142	1.271	1.304	0.973
Q8NFW8	N-acylneuraminate cytidylyltransferase OS=Homo sapiens GN=C	1.346	1.771	1.504	1.255	1.474	1.379	1.632	1.739
Q8NEJ9	Neuroguidin OS=Homo sapiens GN=NGDN PE=1 SV=1 - [NGDN_	1.285	1.465	1.264	1.080	1.380	1.405	1.550	1.392
Q9Y6I3	Epsin-1 OS=Homo sapiens GN=EPN1 PE=1 SV=2 - [EPN1_HUMA	1.603	1.171	1.177	0.879	1.168	1.006	1.158	1.217
O43633	Charged multivesicular body protein 2a OS=Homo sapiens GN=C	1.381	1.009	1.306	1.042	1.310	1.074	1.259	0.986
A0A024QZG0	Ring finger protein 40, isoform CRA_a OS=Homo sapiens GN=RN	1.506	1.671	1.298	0.860	1.500	1.289	1.450	1.072
Q8WUM0	Nuclear pore complex protein Nup133 OS=Homo sapiens GN=NU	1.777	1.932	1.759	1.925	1.615	1.438	2.016	2.133
B4DGM3	SWI/SNF-related matrix-associated actin-dependent regulator of	1.388	1.246	0.916	0.713	1.317	1.323	1.075	1.056
B3KPX5	cDNA FLJ32418 fis, clone SKMUS2000863, weakly similar to PRO	1.315	1.392	1.322	1.253	1.090	1.168	1.283	1.329
Q9NX55	Huntingtin-interacting protein K OS=Homo sapiens GN=HYPK PE	3.089	2.143	2.740	2.259	2.518	1.392	2.805	2.575
Q02952	A-kinase anchor protein 12 OS=Homo sapiens GN=AKAP12 PE=1	1.390	0.986	1.013	0.732	1.236	0.899	1.014	0.925
Q9NQH7	Probable Xaa-Pro aminopeptidase 3 OS=Homo sapiens GN=XPNF	1.253	1.217	1.023	1.181	1.006	1.175	1.246	1.179
Q9NXH8	Torsin-4A OS=Homo sapiens GN=TOR4A PE=1 SV=2 - [TOR4A_H	1.284	1.471	0.910	0.768	1.142	1.281	1.185	0.993
B7Z7Y3	cDNA FLJ61618, highly similar to Discoidin, CUB and LCCL domai	1.616	2.162	1.281	1.187	1.571	1.098	1.202	1.141
A0A024RD07	Trinucleotide repeat containing 5, isoform CRA_c OS=Homo sapie	1.416	1.004	1.304	1.207	1.420	1.150	1.349	1.189
Q9BXF6	Rab11 family-interacting protein 5 OS=Homo sapiens GN=RAB11	1.606	1.938	1.658	1.521	1.186	1.363	1.792	1.554
P13984	General transcription factor IIF subunit 2 OS=Homo sapiens GN=	1.631	1.634	1.446	1.014	1.698	1.279	1.365	1.091
C9JPH1	Golgi apparatus membrane protein TVP23 homolog OS=Homo sa	2.516	2.780	2.600	2.268	1.834	1.636	2.268	2.012
B4DZZ0	cDNA FLJ52128, highly similar to PRA1 family protein 3 OS=Hom	1.097	1.090	0.858	0.838	0.948	0.996	0.971	0.833
Q5SY16	Polynucleotide 5'-hydroxyl-kinase NOL9 OS=Homo sapiens GN=N	1.738	2.009	1.376	1.076	1.870	1.446	1.805	1.222
Q96JM3	Chromosome alignment-maintaining phosphoprotein 1 OS=Homo	1.208	1.653	1.103	0.948	1.054	1.480	1.413	1.256
Q86SF2	N-acetylgalactosaminyltransferase 7 OS=Homo sapiens GN=GAL	1.264	1.440	1.246	1.308	1.098	1.300	1.371	1.263
Q9NZ43	Vesicle transport protein USE1 OS=Homo sapiens GN=USE1 PE=	1.370	1.464	1.335	1.391	1.224	1.241	1.492	1.203
Q9NYF8	Bcl-2-associated transcription factor 1 OS=Homo sapiens GN=BC	1.372	1.619	1.123	1.179	1.096	0.972	1.547	1.623

D6RJ90	COMM domain containing 10, isoform CRA_b OS=Homo sapiens (	1.384	1.043	1.334	1.054	1.229	1.342	1.277	1.170
B2R8X4	cDNA, FLJ94105, highly similar to Homo sapiens GA binding prote	1.839	2.029	2.143	1.621	1.621	1.616	2.038	1.873
A2IDC6	39S ribosomal protein L28, mitochondrial (Fragment) OS=Homo s	1.274	1.475	1.229	1.104	0.958	1.133	1.239	1.237
Q15075	Early endosome antigen 1 OS=Homo sapiens GN=EEA1 PE=1 SV	1.439	1.603	1.277	1.105	1.307	1.102	1.301	1.236
P23258	Tubulin gamma-1 chain OS=Homo sapiens GN=TUBG1 PE=1 SV=	2.051	1.403	1.878	1.282	1.648	1.190	1.754	1.807
B4DLM0	cDNA FLJ60296, highly similar to RNA-binding region-containing p	1.388	1.449	1.414	1.136	1.314	1.231	1.378	1.238
P01034	Cystatin-C OS=Homo sapiens GN=CST3 PE=1 SV=1 - [CYTC_HU	1.585	1.658	1.045	0.660	1.354	1.365	1.590	0.954
E9PPB5	Ester hydrolase C11orf54 (Fragment) OS=Homo sapiens GN=C11	1.478	1.202	1.766	1.513	1.429	0.944	1.376	1.472
P52298	Nuclear cap-binding protein subunit 2 OS=Homo sapiens GN=NC	1.442	1.553	1.524	1.301	1.392	1.338	1.479	1.430
P27707	Deoxycytidine kinase OS=Homo sapiens GN=DCK PE=1 SV=1 - [	0.924	0.746	0.867	0.719	0.995	0.981	1.024	0.795
Q15102	Platelet-activating factor acetylhydrolase IB subunit gamma OS=H	1.203	0.854	1.220	1.071	1.142	0.951	1.270	1.089
G8JLB3	tRNA pseudouridine synthase (Fragment) OS=Homo sapiens GN=	1.684	1.437	1.467	1.147	1.163	1.113	1.322	1.290
P22059	Oxysterol-binding protein 1 OS=Homo sapiens GN=OSBP PE=1 S	1.546	1.210	1.546	1.214	1.641	1.040	1.651	1.309
P38432	Coilin OS=Homo sapiens GN=COIL PE=1 SV=1 - [COIL_HUMAN]	2.050	2.171	1.891	1.786	1.292	1.395	1.788	1.982
B2RB99	cDNA, FLJ95387 OS=Homo sapiens PE=2 SV=1 - [B2RB99_HUM/	1.723	1.450	1.619	1.119	1.793	1.533	1.634	1.286
P63092	Guanine nucleotide-binding protein G(s) subunit alpha isoforms sl	1.333	1.868	1.417	1.557	1.543	1.244	1.325	1.522
Q13601	KRR1 small subunit processome component homolog OS=Homo s	1.549	1.971	1.299	1.055	1.350	1.314	1.493	1.300
A0A087WVB0	ATP-dependent RNA helicase DDX50 OS=Homo sapiens GN=DDX	1.329	1.473	1.056	0.981	1.426	1.263	1.329	1.259
B4DLE7	cDNA FLJ50983, highly similar to Homo sapiens lysocardiolipin ac	1.316	1.426	1.231	1.346	1.150	1.269	1.349	1.295
O75909	Cyclin-K OS=Homo sapiens GN=CCNK PE=1 SV=2 - [CCNK_HUM	1.427	1.541	1.130	0.877	1.293	1.238	1.438	1.218
H3BSM5	Gamma-aminobutyric acid receptor-associated protein-like 2 OS=	1.351	1.103	0.976	0.676	1.533	1.315	1.233	0.969
A0A087WZU5	Tetraspanin-6 OS=Homo sapiens GN=TSPAN6 PE=4 SV=1 - [A0A	6.008	5.832	5.270	4.487	3.992	1.450	4.458	4.810
Q9UHA4	Ragulator complex protein LAMTOR3 OS=Homo sapiens GN=LAM	1.450	1.453	1.281	1.291	1.361	1.165	1.538	1.412
O00411	DNA-directed RNA polymerase, mitochondrial OS=Homo sapiens	1.357	1.605	1.252	1.121	1.165	1.200	1.356	1.168
B7ZMF2	Fanconi anemia, complementation group I OS=Homo sapiens GN	1.566	1.746	1.744	1.741	1.372	1.374	1.982	2.196
Q9HCS7	Pre-mRNA-splicing factor SYF1 OS=Homo sapiens GN=XAB2 PE=	1.375	1.392	1.071	0.902	1.318	1.367	1.304	0.988
P11717	Cation-independent mannose-6-phosphate receptor OS=Homo sa	1.519	1.492	1.261	1.321	1.283	1.085	1.413	1.387
B4DP77	cDNA FLJ57413, highly similar to Mitochondrial 28S ribosomal pro	1.098	1.170	1.700	1.423	1.299	1.327	1.032	1.421
Q96JJ7	Protein disulfide-isomerase TMX3 OS=Homo sapiens GN=TMX3 P	2.517	2.455	2.145	1.949	1.747	1.453	1.728	2.024
Q9Y2V2	Calcium-regulated heat stable protein 1 OS=Homo sapiens GN=C	1.631	0.932	1.996	1.891	1.154	1.025	1.405	1.457
O14672	Disintegrin and metalloproteinase domain-containing protein 10 (	1.309	1.532	1.093	1.008	1.110	1.212	1.026	0.955
B9A018	U4/U6.U5 tri-snRNP-associated protein 2 OS=Homo sapiens GN=	1.338	1.607	1.306	1.058	1.317	1.248	1.460	1.405
P50453	Serpin B9 OS=Homo sapiens GN=SERPINB9 PE=1 SV=1 - [SPB9_	1.422	0.929	1.396	1.181	1.338	1.069	1.168	1.149
Q9Y6A4	Cilia- and flagella-associated protein 20 OS=Homo sapiens GN=C	1.524	1.756	1.562	1.105	1.504	1.285	1.359	1.212
A8KAE0	cDNA FLJ78476, highly similar to Homo sapiens WD repeat and H	1.209	1.027	0.939	0.659	1.197	1.139	1.000	0.818
F8VU51	YLP motif-containing protein 1 OS=Homo sapiens GN=YLPM1 PE=	1.083	1.362	0.976	0.892	1.065	1.325	1.241	1.069
B2R7C2	cDNA, FLJ93375, highly similar to Homo sapiens ZW10, kinetocho	2.194	1.814	1.907	2.134	1.869	1.491	2.257	2.160
O95747	Serine/threonine-protein kinase OSR1 OS=Homo sapiens GN=OX	1.345	1.018	1.541	1.662	1.164	1.107	1.218	1.107
O00566	U3 small nucleolar ribonucleoprotein protein MPP10 OS=Homo sa	1.377	1.416	1.097	0.950	1.412	1.447	1.379	1.223
Q8N4V1	Membrane magnesium transporter 1 OS=Homo sapiens GN=MM(	2.354	2.453	2.000	1.876	1.899	1.794	2.438	1.921

Q96AT1	Uncharacterized protein KIAA1143 OS=Homo sapiens GN=KIAA1	1.559	1.336	1.349	1.048	1.525	1.220	1.145	1.079
O95602	DNA-directed RNA polymerase I subunit RPA1 OS=Homo sapiens	1.559	1.903	1.591	1.213	1.378	1.499	1.793	1.307
H3BPB8	Mannose-6-phosphate isomerase OS=Homo sapiens GN=MPI PE=	0.978	0.770	1.324	1.009	0.880	0.968	1.175	1.048
O15155	BET1 homolog OS=Homo sapiens GN=BET1 PE=1 SV=1 - [BET1_	1.259		1.148	1.077	0.184	1.210	0.939	1.160
Q9BTL3	RNMT-activating mini protein OS=Homo sapiens GN=FAM103A1 I	1.024	1.110	0.965	0.562	1.158	1.276	0.873	0.616
B7Z4S4	cDNA FLJ52567, highly similar to Renin receptor OS=Homo sapie	1.163	1.445	1.078	0.843	0.906	1.444	1.538	1.245
Q96PU8	Protein quaking OS=Homo sapiens GN=QKI PE=1 SV=1 - [QKI_H	1.285	1.301	0.882	0.534	1.458	1.188	0.906	0.649
Q5T653	39S ribosomal protein L2, mitochondrial OS=Homo sapiens GN=M	2.120	2.195	1.444	1.714	1.412	1.127	1.729	1.847
Q9NTJ5	Phosphatidylinositide phosphatase SAC1 OS=Homo sapiens GN=S	1.325	1.325	1.098	1.038	1.023	1.289	1.199	1.142
Q9BRT9	DNA replication complex GINS protein SLD5 OS=Homo sapiens G	1.172	1.035	1.119	0.852	1.390	1.199	1.076	1.100
A0A024R2G1	Cysteine-rich with EGF-like domains 1, isoform CRA_b OS=Homo	1.232	1.339	1.114	1.138	1.068	1.188	1.427	1.191
H3BNF1	Ceroid-lipofuscinosis neuronal protein 6 OS=Homo sapiens GN=C	0.938	1.144	0.892	0.627	0.805	1.242	0.715	
P49454	Centromere protein F OS=Homo sapiens GN=CENPF PE=1 SV=2	1.420	2.011	1.957	1.688	1.252	1.549	2.213	2.099
Q7Z7F7	39S ribosomal protein L55, mitochondrial OS=Homo sapiens GN=	1.270	1.475	1.078	1.057	0.949	1.100	1.171	1.179
B4DZF1	cDNA FLJ56541, highly similar to Ubiquilin-2 OS=Homo sapiens P	1.643	1.275	1.598	1.400	1.502	1.077	1.597	1.605
C9JW94	Kinetochore protein Spc25 (Fragment) OS=Homo sapiens GN=SP	1.722		1.331		1.307	1.107	1.061	1.026
B2R713	cDNA, FLJ93224 OS=Homo sapiens PE=2 SV=1 - [B2R713_HUM/	1.606	1.312	1.150	0.839	1.422	1.218	1.348	0.876
A8K8N7	Phosphoribosylformylglycinamidine synthase (FGAR amidotransfe	1.276	0.942	1.272	1.082	1.174	1.017	1.238	1.184
Q9UL26	Ras-related protein Rab-22A OS=Homo sapiens GN=RAB22A PE=	1.311	1.596	1.149	1.028	1.109	1.194	1.260	1.134
P13929	Beta-enolase OS=Homo sapiens GN=ENO3 PE=1 SV=5 - [ENOB_	1.001	0.922	1.931	1.321	1.249	1.039	1.197	1.303
B4E290	cDNA FLJ50039, highly similar to Homo sapiens solute carrier fan	1.087	1.218	1.028	1.088	0.866	0.988	0.985	0.997
B3KNI2	cDNA FLJ14650 fis, clone NT2RP2002185, highly similar to Ubiqu	1.557	1.107	1.320	1.288	1.434	1.260	1.237	1.245
Q7L4X7	Glioma tumor suppressor candidate region gene 2 (Fragment) OS	1.829	1.981	1.053	0.576	1.892	1.775	1.694	0.996
Q9NQG5	Regulation of nuclear pre-mRNA domain-containing protein 1B OS	1.301	1.347	1.222	0.940	1.347	1.316	1.382	1.181
Q6IQ22	Ras-related protein Rab-12 OS=Homo sapiens GN=RAB12 PE=1 S	1.489	1.884	1.679	1.484	1.217	1.400	1.505	1.517
B3KQK4	cDNA FLJ90619 fis, clone PLACE1002374, highly similar to Cathe	1.250	1.193	0.868	0.756	1.420	1.133	1.087	0.844
P10620	Microsomal glutathione S-transferase 1 OS=Homo sapiens GN=M	2.048	1.989	1.858	1.776	1.677	1.286	1.929	1.560
Q9UMS0	NFU1 iron-sulfur cluster scaffold homolog, mitochondrial OS=Hon	1.956	1.823	1.303	1.475	1.400	1.240	1.686	1.535
Q9Y2P8	RNA 3'-terminal phosphate cyclase-like protein OS=Homo sapiens	1.509	1.901	1.303	1.121	1.370	1.401	1.645	1.289
Q08426	Peroxisomal bifunctional enzyme OS=Homo sapiens GN=EHHADH	1.122	1.263	0.885	0.850	1.019	1.149	1.262	0.983
Q9NRX2	39S ribosomal protein L17, mitochondrial OS=Homo sapiens GN=	1.405	1.527	1.157	1.093	1.305	1.253	1.297	1.286
E7EQZ4	Survival motor neuron protein OS=Homo sapiens GN=SMN1 PE=	1.337	1.434	1.293	1.116	1.491	1.231	1.710	1.555
Q9HB72	PNAS-106 OS=Homo sapiens PE=2 SV=1 - [Q9HB72_HUMAN]	0.510	0.616	0.417	0.262	0.836	0.825	0.672	0.520
Q0VDF9	Heat shock 70 kDa protein 14 OS=Homo sapiens GN=HSPA14 PE	1.666	1.008	1.486	1.039	1.431	1.046	1.197	1.269
Q3MHD2	Protein LSM12 homolog OS=Homo sapiens GN=LSM12 PE=1 SV=	1.589	1.203	1.071	0.955	1.379	1.262	1.091	1.073
Q07666	KH domain-containing, RNA-binding, signal transduction-associate	1.126	1.541	0.906	0.804	1.005	1.207	0.948	0.656
Q9BRT6	Protein LLP homolog OS=Homo sapiens GN=LLPH PE=2 SV=1 -	1.535	1.683	1.291	0.977	1.549	1.496	1.625	1.335
A0A024R943	Torsin family 3, member A, isoform CRA_b OS=Homo sapiens GN	1.633	1.795	1.633	1.574	1.368	1.436	1.737	1.967
O43772	Mitochondrial carnitine/acylcarnitine carrier protein OS=Homo sa	1.521	1.863	1.500	1.664	1.103	1.207	1.580	1.622
A8K2S7	cDNA FLJ77865 OS=Homo sapiens PE=2 SV=1 - [A8K2S7_HUMA	1.105	1.140	1.140	0.896	0.854	1.180	1.220	1.077

B1AJQ6	Syntaxin-12 (Fragment) OS=Homo sapiens GN=STX12 PE=1 SV=	1.343	1.433	1.257	1.205	1.131	1.385	1.289	1.320
A0A024R8B6	Nucleoporin 214kDa, isoform CRA_b OS=Homo sapiens GN=NUP	1.499	1.369	1.412	1.428	1.432	1.328	1.558	1.620
Q8IZV5	Retinol dehydrogenase 10 OS=Homo sapiens GN=RDH10 PE=1 S	1.189	1.474	1.030	1.070	0.920	0.978	1.044	0.917
P60468	Protein transport protein Sec61 subunit beta OS=Homo sapiens G	1.852	2.213	1.887	1.359	1.470	1.907	2.083	1.558
Q9UIG0	Tyrosine-protein kinase BAZ1B OS=Homo sapiens GN=BAZ1B PE=	1.317	1.875	1.457	1.240	1.108	1.298	1.382	1.456
C9J5C3	Programmed cell death protein 10 (Fragment) OS=Homo sapiens	1.414	0.833	1.039	0.786	1.107	1.145	1.192	0.755
P09234	U1 small nuclear ribonucleoprotein C OS=Homo sapiens GN=SNR	1.248	1.333	0.958	0.628	1.283	1.311	1.093	0.876
P13798	Acylamino-acid-releasing enzyme OS=Homo sapiens GN=APEH P	1.278	0.750	1.104	0.855	1.193	0.851	0.982	0.982
P47712	Cytosolic phospholipase A2 OS=Homo sapiens GN=PLA2G4A PE=	1.641	0.958	1.326	0.998	1.275	0.984	1.122	0.925
Q9UFC0	Leucine-rich repeat and WD repeat-containing protein 1 OS=Hom	1.186	1.110	1.224	1.133	1.398	1.342	1.485	1.348
O15344	E3 ubiquitin-protein ligase Midline-1 OS=Homo sapiens GN=MID1	1.937	1.113	1.171	0.909	1.512	0.945	1.199	1.021
P14406	Cytochrome c oxidase subunit 7A2, mitochondrial OS=Homo sapi	1.588	1.840	1.542	1.508	0.875	0.928	1.225	0.987
Q9H3P7	Golgi resident protein GCP60 OS=Homo sapiens GN=ACBD3 PE=	1.567	1.289	1.243	0.860	1.523	1.196	1.157	1.035
Q9NRN7	L-aminoadipate-semialdehyde dehydrogenase-phosphopantethein	1.130	0.797	1.153	0.821	1.062	1.168	1.006	0.955
Q13085	Acetyl-CoA carboxylase 1 OS=Homo sapiens GN=ACACA PE=1 SV	1.374	1.010	1.261	0.986	1.232	0.995	1.152	1.003
P53004	Biliverdin reductase A OS=Homo sapiens GN=BLVRA PE=1 SV=2	1.186	0.914	1.409	1.140	1.087	0.844	1.172	0.999
A8K245	cDNA FLJ75441, highly similar to Homo sapiens vaccinia related k	1.411	1.795	1.285	0.779	1.344	1.305	1.369	1.012
P52888	Thimet oligopeptidase OS=Homo sapiens GN=THOP1 PE=1 SV=2	1.242	0.897	1.252	1.099	1.160	1.054	1.159	1.287
B4DLT2	cDNA FLJ56637, highly similar to Nuclear pore complex protein N	1.622	1.520	1.569	1.563	1.530	1.408	1.757	1.897
P15374	Ubiquitin carboxyl-terminal hydrolase isozyme L3 OS=Homo sapie	1.489	1.500	1.521	1.398	1.328	0.952	1.604	1.713
Q9ULD2	Microtubule-associated tumor suppressor 1 OS=Homo sapiens GN	1.394	0.948	0.961	0.755	1.028	0.810	0.933	0.717
Q9NUL7	Probable ATP-dependent RNA helicase DDX28 OS=Homo sapiens	1.326	1.543	1.015	0.761	1.119	1.241	1.180	0.938
K7ELQ9	Transmembrane protein 205 (Fragment) OS=Homo sapiens GN=T	1.084	1.037	1.000	0.918	0.922	1.121	1.183	0.909
Q96IJ6	Mannose-1-phosphate guanyltransferase alpha OS=Homo sapiens	1.101	0.763	1.285	0.858	1.055	1.107	1.044	0.921
Q9NY93	Probable ATP-dependent RNA helicase DDX56 OS=Homo sapiens	1.533	1.604	1.252	0.793	1.449	1.471	1.527	0.922
Q9NVI7	ATPase family AAA domain-containing protein 3A OS=Homo sapie	0.907	1.025	0.753	0.794	0.771	1.042	0.943	0.899
Q6DEN2	DPYSL3 protein OS=Homo sapiens GN=DPYSL3 PE=2 SV=1 - [Q	1.115	0.692	1.018	0.838	0.994	0.993	1.049	1.004
A8K5S3	cDNA FLJ78449 OS=Homo sapiens PE=2 SV=1 - [A8K5S3_HUMA	1.495	0.923	1.456	1.187	1.158	1.097	1.360	1.232
P43490	Nicotinamide phosphoribosyltransferase OS=Homo sapiens GN=N	1.284	0.989	1.318	0.988	1.117	1.091	1.153	0.979
B2R9Y1	cDNA, FLJ94608, Homo sapiens reticulon 4 interacting protein 1 (	1.227	1.321	0.951	1.003	1.104	1.054	1.187	1.144
A0A024QZY0	Biphenyl hydrolase-like (Serine hydrolase breast epithelial mucin-	1.417	1.268	1.279	1.359	1.132	1.194	1.336	1.299
Q9Y3D3	28S ribosomal protein S16, mitochondrial OS=Homo sapiens GN=	1.014	1.169	0.868	0.844	0.846	1.037	1.001	0.977
Q96J01	THO complex subunit 3 OS=Homo sapiens GN=THOC3 PE=1 SV=	1.795	1.823	1.475	1.389	1.574	1.138	1.680	1.535
A8K5Q1	cDNA FLJ77548, highly similar to Homo sapiens bin3, bicoid-inter	1.506	1.513	1.060	0.778	1.436	1.500	1.162	0.868
Q96BP3	Peptidylprolyl isomerase domain and WD repeat-containing protei	1.570	1.504	1.487	1.030	1.558	1.373	1.401	1.140
Q9HD26	Golgi-associated PDZ and coiled-coil motif-containing protein OS=	1.707	1.479	1.415	1.323	2.127	1.405	1.381	1.450
I3L4W0	CTD nuclear envelope phosphatase 1 OS=Homo sapiens GN=CTD	1.723	1.557	1.471	1.371	1.183	1.560	1.658	1.480
B7Z1N6	Fructose-bisphosphate aldolase OS=Homo sapiens PE=2 SV=1 -	1.421	1.098	1.320	1.191	1.075	0.949	1.215	1.170
P62875	DNA-directed RNA polymerases I, II, and III subunit RPABC5 OS=	1.518	1.689	1.395	1.239	1.523	1.450	1.598	1.286
B3KX16	cDNA FLJ44469 fis, clone UTERU2026090, highly similar to Cartila	3.197	2.061	2.194	1.907	2.871	1.516	2.263	2.305

B4DJQ8	cDNA FLJ55694, highly similar to Dipeptidyl-peptidase 1 (EC 3.4.1	1.392	1.404	1.654	1.669	1.639	1.169	1.836	1.790
A8K5B0	cDNA FLJ75682, highly similar to Homo sapiens armadillo repeat	2.544	1.783	2.036	2.019	1.668	1.403	2.365	2.571
F5H7R9	Parathymosin (Fragment) OS=Homo sapiens GN=PTMS PE=1 SV	0.996	0.722	1.064	0.803	0.896	0.949	0.788	0.748
P19387	DNA-directed RNA polymerase II subunit RPB3 OS=Homo sapiens	1.265	1.407	1.204	0.857	1.227	1.238	1.163	0.982
A8K894	cDNA FLJ77927 OS=Homo sapiens PE=2 SV=1 - [A8K894_HUMA	1.034	1.276	0.872	0.567	1.036	1.107	0.876	0.740
B3KN29	cDNA FLJ13371 fis, clone PLACE1000656, highly similar to PRA1 f	2.528	2.523	1.620	2.125	2.209	1.438	2.358	1.679
Q9UEE9	Craniofacial development protein 1 OS=Homo sapiens GN=CFDP1	1.488	1.113	1.247	1.074	1.251	1.059	1.134	1.194
Q9H6Y2	WD repeat-containing protein 55 OS=Homo sapiens GN=WDR55	1.716	1.659	1.577	1.457	1.806	1.419	1.517	1.274
Q9Y657	Spindlin-1 OS=Homo sapiens GN=SPIN1 PE=1 SV=3 - [SPIN1_HU	1.064	1.187	0.905	0.899	1.122	1.194	0.997	0.894
Q08378	Golgin subfamily A member 3 OS=Homo sapiens GN=GOLGA3 PE	1.473	1.668	1.556	1.482	1.300	1.319	1.527	1.396
Q05BW9	PAPSS1 protein (Fragment) OS=Homo sapiens GN=PAPSS1 PE=2	1.088	1.189	1.350	0.975	1.092	1.033	1.138	0.819
B4DHL7	cDNA FLJ59515 OS=Homo sapiens PE=2 SV=1 - [B4DHL7_HUMA	1.996	1.466	2.215	1.486	1.691	1.250	1.638	1.625
Q15059	Bromodomain-containing protein 3 OS=Homo sapiens GN=BRD3	1.035	1.456	1.068	1.195	0.765	1.001	1.123	1.188
Q9NPA0	ER membrane protein complex subunit 7 OS=Homo sapiens GN=	1.230	1.354	1.016	0.919	0.966	1.261	1.145	0.961
B3KTT0	cDNA FLJ38675 fis, clone IMR322000243, highly similar to Nucleo	1.685	1.392	1.370	1.691	1.631	1.248	1.919	1.788
Q92747	Actin-related protein 2/3 complex subunit 1A OS=Homo sapiens G	1.172	1.191	1.139	0.803	1.369	0.950	1.181	1.132
Q8IYU8	Calcium uptake protein 2, mitochondrial OS=Homo sapiens GN=M	1.785	2.017	1.675	1.405	1.392	1.245	1.688	1.517
Q6UXN9	WD repeat-containing protein 82 OS=Homo sapiens GN=WDR82	1.203	1.303	1.031	0.585	1.182	1.417	1.183	1.036
P28072	Proteasome subunit beta type-6 OS=Homo sapiens GN=PSMB6 P	1.303	0.926	1.015	0.843	1.192	0.999	1.059	0.809
Q6P161	39S ribosomal protein L54, mitochondrial OS=Homo sapiens GN=	1.169	1.345	1.131	0.981	0.928	1.080	1.110	1.144
Q9H6R0	Putative ATP-dependent RNA helicase DHX33 OS=Homo sapiens	1.541	1.754	1.411	1.165	1.353	1.502	1.473	1.283
Q9Y296	Trafficking protein particle complex subunit 4 OS=Homo sapiens G	1.730	1.379	1.475	1.252	1.415	1.285	1.650	0.994
F5GX58	Cell division cycle-associated protein 3 OS=Homo sapiens GN=CD	2.015	1.084	1.332	1.594	1.188	1.262	1.292	1.455
Q13867	Bleomycin hydrolase OS=Homo sapiens GN=BLMH PE=1 SV=1 -	1.279	0.962	1.369	1.361	1.389	1.243	1.505	1.339
B3KY51	cDNA FLJ46863 fis, clone UTERU3011558 OS=Homo sapiens PE=	1.546	1.784	1.263	1.137	1.365	1.198	1.517	1.331
B3KY42	cDNA FLJ46788 fis, clone TRACH3028855, highly similar to Pseud	1.084	0.986	1.040	0.744	1.085	1.124	0.892	0.778
Q09028	Histone-binding protein RBBP4 OS=Homo sapiens GN=RBBP4 PE	1.497	1.382	1.111	0.944	1.513	1.446	1.707	1.525
Q99543	DnaJ homolog subfamily C member 2 OS=Homo sapiens GN=DN	1.325	1.223	1.419	0.982	1.167	1.063	1.094	1.135
E9PNW4	CD59 glycoprotein OS=Homo sapiens GN=CD59 PE=1 SV=1 - [E9	1.940	1.912	1.550	1.365	1.546	1.082	1.014	1.088
P56181	NADH dehydrogenase [ubiquinone] flavoprotein 3, mitochondrial	1.438	1.387	1.154	1.384	1.054	1.105	1.215	1.269
A8K0P8	cDNA FLJ78223 OS=Homo sapiens PE=2 SV=1 - [A8K0P8_HUMA	1.615	1.788	1.396	0.972	1.576	1.504	1.520	0.846
H3BLU7	Aflatoxin B1 aldehyde reductase member 2 (Fragment) OS=Homo	1.049	0.886	1.084	1.109	0.966	0.985	1.117	1.132
P07942	Laminin subunit beta-1 OS=Homo sapiens GN=LAMB1 PE=1 SV=	1.387	1.321	1.232	1.190	1.365	1.224	1.334	1.254
Q9BPX6	Calcium uptake protein 1, mitochondrial OS=Homo sapiens GN=M	1.283	1.450	1.015	0.906	1.040	1.134	1.178	1.152
P11234	Ras-related protein Ral-B OS=Homo sapiens GN=RALB PE=1 SV=	2.251	2.732	2.102	1.767	1.516	1.185	1.779	2.283
B4DR80	cDNA FLJ61159, highly similar to Serine/threonine-protein kinase	1.982	1.542	1.526	1.292	1.561	1.028	1.510	1.387
O15269	Serine palmitoyltransferase 1 OS=Homo sapiens GN=SPTLC1 PE=	1.198	1.262	1.325	1.104	0.967	1.367	1.325	1.224
Q9BW27	Nuclear pore complex protein Nup85 OS=Homo sapiens GN=NUP	1.641	1.593	1.531	1.492	1.485	1.431	1.623	1.663
Q86U79	Adenosine kinase OS=Homo sapiens PE=2 SV=1 - [Q86U79_HUM	1.505	1.146	1.580	1.358	1.264	0.947	1.299	1.426
P35052	Glypican-1 OS=Homo sapiens GN=GPC1 PE=1 SV=2 - [GPC1_HU	0.944	1.779	0.761	0.490	1.045	1.164	0.919	0.527

Q9Y3U8	60S ribosomal protein L36 OS=Homo sapiens GN=RPL36 PE=1 S	1.384	1.242	1.209	1.086	1.262	1.309	1.263	1.135
Q7Z7H5	Transmembrane emp24 domain-containing protein 4 OS=Homo s	1.408	1.330	1.255	1.318	1.131	1.254	1.229	1.319
Q6UX53	Methyltransferase-like protein 7B OS=Homo sapiens GN=METTL7	1.116	1.177	1.106	0.981	0.856	1.214	1.212	1.070
Q92973	Transportin-1 OS=Homo sapiens GN=TNPO1 PE=1 SV=2 - [TNPO	1.809	1.311	1.690	1.183	1.708	1.305	1.391	1.371
Q53GG0	Epithelial protein lost in neoplasm beta variant (Fragment) OS=H	1.448	1.694	1.615	1.617	1.288	1.373	1.958	1.805
O00291	Huntingtin-interacting protein 1 OS=Homo sapiens GN=HIP1 PE=	1.077	1.210	1.040	0.945	1.251	1.173	1.171	1.039
B2R694	cDNA, FLJ92849, highly similar to Homo sapiens lanosterol syntha	1.049	1.027	0.839	0.858	0.914	1.114	0.928	0.830
Q9H7C9	Mth938 domain-containing protein OS=Homo sapiens GN=AAMD	0.978	0.913	1.273	1.145	0.931	0.832	1.023	1.032
O75400	Pre-mRNA-processing factor 40 homolog A OS=Homo sapiens GN	1.329	1.513	1.357	1.120	1.397	1.397	1.574	1.442
Q96CB8	Integrator complex subunit 12 OS=Homo sapiens GN=INTS12 PE	0.970	1.228	0.988	0.451	1.014	1.314	0.934	0.699
P10768	S-formylglutathione hydrolase OS=Homo sapiens GN=ESD PE=1	1.627	1.361	1.885	1.638	1.558	1.089	1.458	1.726
Q8IW76	EIF2AK2 protein (Fragment) OS=Homo sapiens GN=EIF2AK2 PE=	1.621	1.064	1.163	0.795	1.445	1.051	1.219	1.054
E9PL57	Protein NEDD8-MDP1 (Fragment) OS=Homo sapiens GN=NEDD8	1.842	1.591	1.562	1.590	1.996	1.021	1.765	2.078
H3BQZ5	UPF0235 protein C15orf40 OS=Homo sapiens GN=C15orf40 PE=	1.654	2.772	1.603	2.139	0.954	1.322	1.169	1.339
Q5STZ8	ATP-binding cassette sub-family F member 1 (Fragment) OS=Hor	1.389	1.260	1.273	0.906	1.257	1.335	0.972	0.910
Q6FHU0	Proteasome subunit beta type (Fragment) OS=Homo sapiens GN	1.286	1.055	0.948	0.864	1.179	1.261	1.029	0.971
O95298	NADH dehydrogenase [ubiquinone] 1 subunit C2 OS=Homo sapie	2.224	2.580	1.951	2.021	1.495	1.409	2.218	2.172
B4DRS4	cDNA FLJ60139, highly similar to Homo sapiens HIV TAT specific	1.494	1.589	1.109	0.857	1.509	1.193	1.235	0.931
H3BSM7	UPF0420 protein C16orf58 OS=Homo sapiens GN=C16orf58 PE=	1.630	1.455	1.347	1.159	1.387	1.086	1.596	1.233
Q8TCZ2	CD99 antigen-like protein 2 OS=Homo sapiens GN=CD99L2 PE=1	3.545	3.980	3.951	3.216	2.587	1.550	1.136	2.940
O75116	Rho-associated protein kinase 2 OS=Homo sapiens GN=ROCK2 P	1.840	1.264	1.690	1.434	1.665	1.152	1.507	1.410
P51151	Ras-related protein Rab-9A OS=Homo sapiens GN=RAB9A PE=1	2.403	2.333	2.490	3.563	1.361	1.317	1.675	2.484
Q13042	Cell division cycle protein 16 homolog OS=Homo sapiens GN=CD	3.295	2.799	2.570	2.131	2.857	1.496	2.726	2.421
Q96EX1	Small integral membrane protein 12 OS=Homo sapiens GN=SMIN	2.018	2.530	1.544	1.626	1.759	1.659	2.141	1.754
I3L2L5	Protein FAM195B OS=Homo sapiens GN=FAM195B PE=1 SV=1 -	1.798	1.423	1.462	1.002	1.575	1.195	1.497	1.160
P33527	Multidrug resistance-associated protein 1 OS=Homo sapiens GN=	1.356	1.641	1.298	1.365	1.404	1.253	1.375	1.338
C9JP00	Muscleblind-like protein 1 OS=Homo sapiens GN=MBNL1 PE=1 S	1.754	1.751	1.434	0.974	1.754	1.122	1.284	1.115
P11441	Ubiquitin-like protein 4A OS=Homo sapiens GN=UBL4A PE=1 SV=	1.295	1.122	1.179	0.908	1.174	1.154	1.152	1.013
Q9H307	Pinin OS=Homo sapiens GN=PNN PE=1 SV=4 - [PININ_HUMAN]	1.509	1.576	1.357	1.335	1.357	1.346	1.452	1.574
Q14534	Squalene monooxygenase OS=Homo sapiens GN=SQLE PE=1 SV	1.224	1.301	1.019	0.796	0.902	1.303	1.112	0.818
P55060	Exportin-2 OS=Homo sapiens GN=CSE1L PE=1 SV=3 - [XPO2_HU	1.689	1.099	1.465	1.118	1.524	1.072	1.413	1.149
Q8WTT2	Nucleolar complex protein 3 homolog OS=Homo sapiens GN=NO	1.400	1.774	1.411	1.190	1.246	1.323	1.483	1.377
B7Z9F3	cDNA, FLJ78817, highly similar to Metaxin-2 OS=Homo sapiens P	1.461	1.813	1.371	1.425	1.517	1.189	1.469	1.526
Q16854	Deoxyguanosine kinase, mitochondrial OS=Homo sapiens GN=DC	0.223	0.258	0.243	0.241	0.329	1.011	0.287	0.344
O14656	Torsin-1A OS=Homo sapiens GN=TOR1A PE=1 SV=1 - [TOR1A_H	1.520	1.474	1.450	1.561	1.441	1.293	1.656	1.803
P35269	General transcription factor IIF subunit 1 OS=Homo sapiens GN=	1.178	1.405	1.253	0.861	1.288	1.182	0.908	0.985
P56545	C-terminal-binding protein 2 OS=Homo sapiens GN=CTBP2 PE=1	1.242	1.041	0.936	0.545	1.289	1.213	0.959	0.844
Q9NS00	Glycoprotein-N-acetylgalactosamine 3-beta-galactosyltransferase	2.171	1.989	1.475	1.529	1.679	1.692	2.510	1.894
Q14694	Ubiquitin carboxyl-terminal hydrolase 10 OS=Homo sapiens GN=U	1.331	1.469	1.302	0.948	1.213	1.137	1.288	1.016
Q9UHQ9	NADH-cytochrome b5 reductase 1 OS=Homo sapiens GN=CYB5R	1.180	1.454	1.178	1.112	1.233	1.203	1.380	1.364

Q96QR8	Transcriptional activator protein Pur-beta OS=Homo sapiens GN=	1.589	1.616	1.262	1.075	1.552	1.278	1.390	1.136
Q9H3H0	Vacuolar ATP synthase subunit D homolog OS=Homo sapiens GN	1.242	1.181	1.092	1.000	1.243	1.169	1.441	1.117
K7ENV7	Isochorismatase domain-containing protein 2, mitochondrial (Frag	2.008	1.985	2.036	2.020	1.201	1.301	1.913	1.705
P55011	Solute carrier family 12 member 2 OS=Homo sapiens GN=SLC12/	1.365	1.592	1.291	1.545	1.082	1.186	1.220	1.334
Q9NQ50	39S ribosomal protein L40, mitochondrial OS=Homo sapiens GN=	1.134	1.228	0.891	0.804	0.801	1.029	1.027	0.992
Q9NPL8	Complex I assembly factor TIMMDC1, mitochondrial OS=Homo sa	1.194	1.216	0.812	1.045	0.913	1.130	1.152	1.117
Q00765	Receptor expression-enhancing protein 5 OS=Homo sapiens GN=	1.465	1.629	1.356	1.246	1.272	1.286	1.434	1.412
Q2NL82	Pre-rRNA-processing protein TSR1 homolog OS=Homo sapiens G	1.407	1.541	1.481	1.109	1.394	1.379	1.293	1.099
Q8NBY1	RNA-binding protein 4B OS=Homo sapiens GN=RBM4B PE=2 SV=	1.347	0.948	1.414	0.936	1.297	1.036	1.146	1.059
B7ZA10	cDNA, FLJ79024, highly similar to Arfaptin-1 OS=Homo sapiens P	1.495	1.199	1.222	0.966	1.207	1.103	1.211	1.150
B4E1E2	cDNA FLJ61530, highly similar to Hepatocyte growth factor-regul	1.547	1.195	1.397	1.165	1.459	1.098	1.386	1.242
P09496	Clathrin light chain A OS=Homo sapiens GN=CLTA PE=1 SV=1 - [	1.320	1.225	1.124	0.997	1.270	1.028	1.226	1.187
E5RIP4	Zinc transporter ZIP14 (Fragment) OS=Homo sapiens GN=SLC39	1.456	2.474	1.731	1.465	1.326	1.787	2.322	2.045
B4DG57	cDNA FLJ61230, highly similar to PHD finger protein 14 OS=Hom	1.412	1.832	1.392	1.535	1.338	1.597	1.745	1.831
Q9Y3B3	Transmembrane emp24 domain-containing protein 7 OS=Homo s	1.312	1.377	1.314	1.161	1.258	1.216	1.291	1.155
B2R4I8	cDNA, FLJ92106, highly similar to Homo sapiens adaptor-related	1.248	0.654	1.080	0.877	1.008	0.833	1.053	0.838
Q8IYB8	ATP-dependent RNA helicase SUPV3L1, mitochondrial OS=Homo	1.574	1.604	1.321	1.278	1.218	1.213	1.369	1.328
B7Z2X0	cDNA FLJ60543, highly similar to Active breakpoint cluster region	1.403	0.899	1.215	1.011	1.180	0.940	1.063	1.092
Q9UKA9	Polypyrimidine tract-binding protein 2 OS=Homo sapiens GN=PTE	1.118	1.429	0.852	0.589	1.086	1.200	1.029	0.700
E9PL10	Transcription factor BTF3 homolog 4 OS=Homo sapiens GN=BTF3	4.921	3.385	4.610	3.058	1.842	1.000	3.362	3.531
Q86XN0	MRPL43 protein (Fragment) OS=Homo sapiens GN=MRPL43 PE=	1.301	1.281	0.939	1.087	0.991	1.386	0.993	1.077
B3KUM4	cDNA FLJ40239 fis, clone TESTI2023436, highly similar to Peptidy	2.335	1.173	2.047	2.268	1.424	1.193	1.469	1.525
Q6ZNJ9	FLJ00319 protein (Fragment) OS=Homo sapiens GN=FLJ00319 P	1.349	1.186	1.342	1.525	1.201	1.084	1.382	1.552
Q9Y3D6	Mitochondrial fission 1 protein OS=Homo sapiens GN=FIS1 PE=1	1.224	1.107	1.060	0.890	1.190	1.293	1.111	1.099
B7Z896	Kinesin-like protein KIFC3 OS=Homo sapiens GN=KIFC3 PE=2 SV	1.422	1.092	0.919	0.580	1.216	1.043	0.975	0.626
Q9HBB9	HC56 OS=Homo sapiens PE=4 SV=1 - [Q9HBB9_HUMAN]	1.996	2.297	2.332	1.910	1.616	1.750	2.425	2.301
P30566	Adenylosuccinate lyase OS=Homo sapiens GN=ADSL PE=1 SV=2	1.361	0.861	1.184	0.975	1.083	0.968	1.123	1.006
B7ZLC9	GEMIN5 protein OS=Homo sapiens GN=GEMIN5 PE=2 SV=1 - [B	1.695	1.545	1.824	1.340	1.419	1.218	1.544	1.471
Q9GZP4	PITH domain-containing protein 1 OS=Homo sapiens GN=PITHD	1.424	1.263	1.380	1.116	1.237	1.157	1.520	1.409
Q86Y56	HEAT repeat-containing protein 2 OS=Homo sapiens GN=HEATR2	1.512	1.076	1.353	0.964	1.319	1.115	1.361	1.149
O15231	Zinc finger protein 185 OS=Homo sapiens GN=ZNF185 PE=1 SV=	1.340	1.101	0.981	0.647	1.248	1.322	1.091	0.886
Q14166	Tubulin--tyrosine ligase-like protein 12 OS=Homo sapiens GN=TT	1.201	0.989	1.248	1.028	1.004	0.953	1.155	1.098
Q8N4Q1	Mitochondrial intermembrane space import and assembly protein	2.181	2.431	2.237	2.013	2.110	2.045	2.424	2.331
P82912	28S ribosomal protein S11, mitochondrial OS=Homo sapiens GN=	1.430	1.387	1.162	1.059	1.120	1.150	1.497	1.197
O00268	Transcription initiation factor TFIID subunit 4 OS=Homo sapiens (	1.063	1.385	1.255	1.046	1.149	1.309	1.334	1.330
O60427	Fatty acid desaturase 1 OS=Homo sapiens GN=FADS1 PE=1 SV=	1.042	0.844	0.692	0.507	0.576	0.832	0.696	0.510
O95373	Importin-7 OS=Homo sapiens GN=IPO7 PE=1 SV=1 - [IPO7_HUI	1.656	1.276	1.745	1.551	1.638	1.291	1.426	1.479
P30046	D-dopachrome decarboxylase OS=Homo sapiens GN=DDT PE=1	1.293	0.855	1.422	1.214	1.021	1.142	1.200	1.227
A8K6V6	cDNA FLJ75883, highly similar to Homo sapiens glucosamine (N-a	1.129	0.989	1.188	1.239	1.197	1.014	1.230	1.253
Q12965	Unconventional myosin-Ie OS=Homo sapiens GN=MYO1E PE=1 S	1.632	1.113	0.939	0.668	1.260	1.055	0.942	0.586

E9PS06	Ras-related protein Rab-30 (Fragment) OS=Homo sapiens GN=R/	1.284	1.362	1.460	0.924	1.369	1.213	1.158	
Q8N3U4	Cohesin subunit SA-2 OS=Homo sapiens GN=STAG2 PE=1 SV=3	0.497	0.560	0.541	0.446	0.941	1.208	0.928	0.619
Q9H4L4	Sentrin-specific protease 3 OS=Homo sapiens GN=SENP3 PE=1 S	1.833	1.718	1.091	0.862	1.982	1.232	1.758	1.139
O43598	2'-deoxynucleoside 5'-phosphate N-hydrolase 1 OS=Homo sapien	1.844	1.200	2.126	1.555	1.661	0.981	1.727	1.562
B2RDR4	cDNA, FLJ96732, highly similar to Homo sapiens testis derived tra	1.017	0.756	0.978	0.761	1.025	0.918	0.938	0.884
Q5JVM0	Unconventional myosin-VI (Fragment) OS=Homo sapiens GN=MY	1.179	1.043	1.094	1.092	1.068	1.105	1.338	1.132
Q13423	NAD(P) transhydrogenase, mitochondrial OS=Homo sapiens GN=	1.321	1.682	1.461	1.625	0.984	1.123	1.351	1.446
Q9Y2A7	Nck-associated protein 1 OS=Homo sapiens GN=NCKAP1 PE=1 S	1.518	0.980	1.217	0.880	1.381	1.065	1.288	0.994
B3KSJ3	cDNA FLJ36409 fis, clone THYMU2010448, highly similar to Regu	1.804	1.916	1.947	1.454	1.500	1.344	1.770	1.834
Q13619	Cullin-4A OS=Homo sapiens GN=CUL4A PE=1 SV=3 - [CUL4A_HU	1.929	1.869	1.467	1.109	1.886	1.218	1.831	1.287
Q9BSV6	tRNA-splicing endonuclease subunit Sen34 OS=Homo sapiens GN	1.367	0.990	0.934	0.613	1.372	0.967	0.997	0.782
Q96HT3	POLR1C protein (Fragment) OS=Homo sapiens GN=POLR1C PE=	1.405	1.693	1.446	1.372	1.483	1.475	1.859	1.610
A8K2G0	cDNA FLJ76605, highly similar to Homo sapiens secretory carrier	1.107	1.071	0.998	0.994	0.954	1.050	1.012	1.103
C9JTE9	B-cell receptor-associated protein 29 (Fragment) OS=Homo sapie	1.205	1.167	1.080	1.042	0.787	1.240	1.221	1.099
Q8NBZ0	INO80 complex subunit E OS=Homo sapiens GN=INO80E PE=1 S	1.216	1.379	1.013	0.798	1.047	1.318	1.127	1.215
Q8N4T8	Carbonyl reductase family member 4 OS=Homo sapiens GN=CBR	1.163	1.177	1.043	0.802	0.967	1.020	0.957	0.803
B2R880	cDNA, FLJ93778, highly similar to Homo sapiens CD38 antigen (p	1.271	1.575	1.140	1.410	1.020	1.174	1.207	1.185
A8K6T5	cDNA FLJ75144, highly similar to Homo sapiens ash2 (absent, sm	1.452	1.727	1.262	0.989	1.633	1.378	1.696	1.343
Q9H2G2	STE20-like serine/threonine-protein kinase OS=Homo sapiens GN	1.547	0.947	1.410	1.012	1.232	1.016	1.319	1.098
Q9H9Y2	Ribosome production factor 1 OS=Homo sapiens GN=RPF1 PE=1	1.451	1.774	1.123	0.644	1.530	1.373	1.340	0.833
H3BN55	Ras-related protein Rab-27A (Fragment) OS=Homo sapiens GN=I	0.936	1.198	1.112	1.261	0.845	1.194	1.393	1.357
P51809	Vesicle-associated membrane protein 7 OS=Homo sapiens GN=V/	1.675	1.771	1.355	1.564	1.309	1.392	1.883	1.582
O95067	G2/mitotic-specific cyclin-B2 OS=Homo sapiens GN=CCNB2 PE=1	1.340	1.993	1.945	1.705	1.002	1.721	3.566	3.918
O43818	U3 small nucleolar RNA-interacting protein 2 OS=Homo sapiens G	1.756	1.942	1.678	1.702	1.700	1.371	1.688	1.601
Q9P031	Thyroid transcription factor 1-associated protein 26 OS=Homo sa	1.089	1.338	0.866	0.620	1.185	1.330	1.088	0.813
O95758	Polypyrimidine tract-binding protein 3 OS=Homo sapiens GN=PTE	2.128	1.994	1.743	1.359	2.160	1.274	1.946	1.614
Q92879	CUGBP Elav-like family member 1 OS=Homo sapiens GN=CELF1 I	1.271	1.331	0.734	0.579	1.204	1.340	0.924	0.780
B3KR70	cDNA FLJ33764 fis, clone BRCOC2000360, highly similar to Vacuc	2.519	2.916	2.123	2.196	2.125	1.765	2.938	2.308
P49756	RNA-binding protein 25 OS=Homo sapiens GN=RBM25 PE=1 SV=	1.866	1.908	1.639	1.304	1.549	1.471	1.872	1.574
Q5R3B4	Mitochondrial pyruvate carrier 2 (Fragment) OS=Homo sapiens G	1.589	1.726	0.976	1.517	1.134	1.040	1.422	1.468
L0R8F8	Alternative protein SMCR7L OS=Homo sapiens GN=SMCR7L PE=4	0.685	0.932	0.684	0.713	0.759	1.168	0.854	0.702
F8W8R3	DNA polymerase delta subunit 2 OS=Homo sapiens GN=POLD2 P	1.515	1.501	1.234	1.054	1.371	1.183	1.139	1.078
E9PNN3	6-pyruvoyl tetrahydrobiopterin synthase OS=Homo sapiens GN=F	1.289	0.956	1.271	1.237	1.247	1.158	1.364	1.401
Q8TCC3	39S ribosomal protein L30, mitochondrial OS=Homo sapiens GN=	1.219	1.389	1.114	1.083	0.902	1.132	1.187	1.168
A6PVJ6	RanBP-type and C3HC4-type zinc finger-containing protein 1 OS=	1.181	0.688	1.113	0.776	1.215	0.787	0.794	0.824
Q14151	Scaffold attachment factor B2 OS=Homo sapiens GN=SAFB2 PE=	1.317	1.449	1.082	0.842	1.231	1.433	1.590	1.407
Q96E29	Transcription termination factor 3, mitochondrial OS=Homo sapie	1.188	1.044	0.813	0.630	0.863	0.995	0.901	0.759
Q13045	Protein flightless-1 homolog OS=Homo sapiens GN=FLII PE=1 SV	1.356	1.080	1.085	0.800	1.170	1.156	1.118	0.859
Q9H6S3	Epidermal growth factor receptor kinase substrate 8-like protein 2	1.260	0.909	1.171	0.904	1.223	0.958	1.234	1.014
Q9NP66	High mobility group protein 20A OS=Homo sapiens GN=HMG20A	1.429	1.467	1.250	1.114	1.194	1.378	1.254	1.362

P29372	DNA-3-methyladenine glycosylase OS=Homo sapiens GN=MPG PI	1.288	1.397	1.286	1.013	1.355	1.178	1.293	1.537
Q6JHV3	Ubiquitin carboxyl-terminal hydrolase OS=Homo sapiens GN=USF	1.330	1.329	0.947	0.938	1.154	1.180	1.018	0.735
A0A024R872	Chromosome 9 open reading frame 88, isoform CRA_a OS=Homo	1.376	1.167	1.343	1.203	1.205	0.952	1.169	1.078
B4DIH2	cDNA FLJ60563, highly similar to Ganglioside-induceddifferentiati	1.456	1.773	1.416	1.376	1.081	1.316	1.632	1.592
O75319	RNA/RNP complex-1-interacting phosphatase OS=Homo sapiens (	1.483	1.812	1.227	0.827	1.638	1.648	1.580	1.112
B4DXN3	cDNA FLJ57657, highly similar to Charged multivesicular body pro	1.298	0.918	1.266	1.028	1.000	1.085	1.237	1.043
B2R7G6	cDNA, FLJ93437, highly similar to Homo sapiens histidyl-tRNA syr	1.228	1.225	0.940	0.810	1.007	1.102	1.093	0.873
B4DRA5	cDNA FLJ61346, highly similar to Protein transport protein Sec23I	2.388	1.891	2.166	1.701	1.699	1.800	2.095	2.116
Q99470	Stromal cell-derived factor 2 OS=Homo sapiens GN=SDF2 PE=1 S	0.930	1.043	0.680	0.869	0.968	1.282	1.068	1.202
Q969X6	Cirhin OS=Homo sapiens GN=CIRH1A PE=1 SV=1 - [CIR1A_HUM	1.897	2.374	2.013	1.702	1.796	1.447	2.052	1.747
A0A087X2D8	C-Jun-amino-terminal kinase-interacting protein 4 OS=Homo sapi	1.346	0.894	1.146	0.939	1.182	0.968	1.063	0.971
B3KQQ0	cDNA PSEC0007 fis, clone NT2RM1000634, highly similar to FK50	1.702	1.326	1.507	1.546	1.635	1.253	1.672	1.779
H0YNH8	Uveal autoantigen with coiled-coil domains and ankyrin repeats C	1.416	1.162	0.984	0.848	1.227	1.042	1.047	1.001
Q9BW92	Threonine--tRNA ligase, mitochondrial OS=Homo sapiens GN=TA	1.164	1.123	0.907	0.916	0.894	1.002	1.153	1.055
F5H5X6	Probable 28S rRNA (cytosine(4447)-C(5))-methyltransferase (Frag	1.145	1.716	1.268	0.777	0.860	1.068	1.161	1.089
Q5JPT2	SH3 domain-containing kinase-binding protein 1 (Fragment) OS=	1.394	0.842	1.041	0.725	1.130	0.924	0.919	0.737
Q5TC12	ATP synthase mitochondrial F1 complex assembly factor 1 OS=Hc	1.565	1.675	1.371	1.344	1.317	1.188	1.395	1.683
Q9P0I2	ER membrane protein complex subunit 3 OS=Homo sapiens GN=	1.906	1.915	1.752	1.536	1.548	1.432	2.011	1.644
B2RBA0	cDNA, FLJ95388, highly similar to Homo sapiens step II splicing f	1.852	1.992	1.748	1.344	1.732	1.714	1.970	1.806
P17858	ATP-dependent 6-phosphofructokinase, liver type OS=Homo sapi	1.796	0.902	1.627	1.114	1.292	1.015	1.461	1.283
B3KU60	cDNA FLJ39235 fis, clone OCBBF2007829, highly similar to Mus n	1.573	1.482	1.793	1.380	1.580	1.186	1.251	1.393
Q14677	Clathrin interactor 1 OS=Homo sapiens GN=CLINT1 PE=1 SV=1 -	1.142	1.096	1.020	0.811	1.051	1.028	1.015	0.848
A0A024RAD5	Dolichyl-diphosphooligosaccharide--protein glycosyltransferase 48	1.194	1.250	1.134	1.031	0.987	1.111	1.229	1.018
B2R6H6	cDNA, FLJ92954, highly similar to Homo sapiens TAF9-like RNA p	1.263	1.721	1.474	1.188	1.350	1.529	1.805	1.563
A0A024R588	Splicing factor 1, isoform CRA_d OS=Homo sapiens GN=SF1 PE=	1.098	1.363	0.939	0.683	1.232	1.362	1.149	0.867
Q9H6R4	Nucleolar protein 6 OS=Homo sapiens GN=NOL6 PE=1 SV=2 - [N	1.244	1.472	1.296	1.230	1.308	1.309	1.410	1.221
X5D7T7	C-terminal binding protein 1 isoform D OS=Homo sapiens GN=CT	1.386	1.306	0.983	0.618	1.542	1.171	1.185	0.917
B4DXV9	cDNA FLJ53678 OS=Homo sapiens PE=2 SV=1 - [B4DXV9_HUMA	1.255	1.313	1.005	1.071	1.080	1.090	1.151	1.130
H7C5V3	28S ribosomal protein S28, mitochondrial (Fragment) OS=Homo s	1.499	1.493	1.199	1.382	0.965	1.162	1.466	1.456
Q96GG9	DCN1-like protein 1 OS=Homo sapiens GN=DCUN1D1 PE=1 SV=	2.157	1.758	1.931	1.522	1.940	1.362	1.789	1.863
Q59ET7	Thioredoxin reductase 2 isoform 1 variant (Fragment) OS=Homo	1.650	1.604	1.383	1.599	1.166	1.297	1.635	1.811
B9EIS5	PCM1 protein OS=Homo sapiens GN=PCM1 PE=2 SV=1 - [B9EIS	1.058	1.226	0.944	0.772	0.880	1.165	0.925	0.836
Q7Z7M4	Superoxide dismutase (Fragment) OS=Homo sapiens GN=SOD2 I	2.193	1.998	1.907	1.944	1.479	1.351	1.345	1.757
Q6IBT1	Proteasome subunit beta type OS=Homo sapiens GN=PSMB7 PE=	1.858	1.337	1.222	1.002	1.695	1.142	1.233	1.074
P20645	Cation-dependent mannose-6-phosphate receptor OS=Homo sap	1.365	1.324	1.299	1.204	1.405	1.345	1.293	1.161
Q96SK2	Transmembrane protein 209 OS=Homo sapiens GN=TMEM209 PE	1.194	1.496	1.192	1.034	0.930	1.409	1.184	1.056
A0A087WZ13	Ribonucleoprotein PTB-binding 1 OS=Homo sapiens GN=RAVER1	0.893	1.136	0.711	0.517	1.045	1.281	0.925	0.838
Q13425	Beta-2-syntrophin OS=Homo sapiens GN=SNTB2 PE=1 SV=1 - [S	0.979	1.411	0.962	0.749	0.914	0.997	0.854	0.701
Q96NC0	Zinc finger matrin-type protein 2 OS=Homo sapiens GN=ZMAT2 F	0.479	0.429	0.842	0.330	0.782	1.279	0.677	0.395
Q8NBT6	cDNA FLJ90758 fis, clone SKNMC1000082, highly similar to Mitoc	1.693	2.556	2.365	2.452	1.039	1.442	2.074	2.450

Q9BSE5	Agmatinase, mitochondrial OS=Homo sapiens GN=AGMAT PE=1	1.264	1.347	1.202	1.198	1.061	1.352	1.422	1.260
Q02241	Kinesin-like protein KIF23 OS=Homo sapiens GN=KIF23 PE=1 SV	1.086	1.236	0.651	0.465	1.114	1.128	0.967	0.608
O75063	Glycosaminoglycan xylosylkinase OS=Homo sapiens GN=FAM20B	1.239	1.551	1.321	1.368	1.261	1.279	1.410	1.253
Q96ST3	Paired amphipathic helix protein Sin3a OS=Homo sapiens GN=SI	1.193	1.232	0.809	0.552	1.100	1.051	1.042	0.704
P43155	Carnitine O-acetyltransferase OS=Homo sapiens GN=CRAT PE=1	1.203	1.288	1.030	1.000	0.918	1.024	1.111	1.062
P52594	Arf-GAP domain and FG repeat-containing protein 1 OS=Homo sa	1.229	0.915	1.162	0.959	1.139	0.998	1.110	0.932
Q8TBF4	Zinc finger CCHC-type and RNA-binding motif-containing protein 1	1.347	1.584	0.966	0.874	1.258	1.292	1.223	0.969
Q05CR6	VRK2 protein (Fragment) OS=Homo sapiens GN=VRK2 PE=2 SV=	1.343	1.452	1.635	0.755	1.425	1.648	1.383	1.112
A0A087X106	Keratin, type II cuticular Hb1 OS=Homo sapiens GN=KRT81 PE=4	2.117	1.043	1.089	0.788	1.738	1.199	1.006	0.714
Q96I36	Cytochrome c oxidase assembly protein COX14 OS=Homo sapien	2.926	3.083	2.337	2.069	1.665	0.964	2.716	2.366
Q6FI30	Nuclear factor 1 OS=Homo sapiens GN=NFIC PE=2 SV=1 - [Q6F	1.293	1.417	1.117	0.780	1.247	1.254	1.322	1.230
A0A087WXM8	Basal cell adhesion molecule OS=Homo sapiens GN=BCAM PE=4	0.976	1.286	1.195	1.254	0.945	0.985	1.265	1.337
A2ACR1	Proteasome subunit beta type OS=Homo sapiens GN=PSMB9 PE=	1.130	0.579	0.580	0.372	1.173	0.867	0.812	0.586
Q8NE71	ATP-binding cassette sub-family F member 1 OS=Homo sapiens G	1.211	1.203	1.417	0.971	1.126	1.091	1.189	1.005
Q9H2J4	Phosducin-like protein 3 OS=Homo sapiens GN=PDCL3 PE=1 SV=	2.252	1.790	2.325	1.705	1.852	1.309	2.243	2.069
F8VXD2	Cyclin-dependent kinase 4 (Fragment) OS=Homo sapiens GN=CD	1.126	0.860	0.789	0.480	1.288	1.170	0.852	0.624
A8K2T7	cDNA FLJ76780, highly similar to Homo sapiens epidermal growth	1.456	0.929	0.348	0.219	1.455	1.324	1.113	1.359
Q59F20	Neuropilin-1 variant (Fragment) OS=Homo sapiens PE=2 SV=1 -	1.071	0.996	0.520	0.599	1.063	0.916	0.756	0.624
P58546	Myotrophin OS=Homo sapiens GN=MTPN PE=1 SV=2 - [MTPN_H	4.013	3.052	4.299	3.812	2.627	1.298	3.163	4.040
O96019	Actin-like protein 6A OS=Homo sapiens GN=ACTL6A PE=1 SV=1	2.242	2.177	1.679	1.544	2.113	1.450	2.474	2.168
B3KS18	cDNA FLJ35285 fis, clone PROST2008079, highly similar to Golgi	1.538	1.797	1.425	1.142	1.692	1.662	1.572	1.390
Q8NEF9	Serum response factor-binding protein 1 OS=Homo sapiens GN=S	1.831	2.256	1.538	1.336	1.727	1.588	1.890	1.349
Q9HBH0	Rho-related GTP-binding protein RhoF OS=Homo sapiens GN=RH	2.104	2.415	1.249	1.234	1.835	1.528	1.401	1.356
A0A024R7S3	Clathrin, light polypeptide (Lcb), isoform CRA_c OS=Homo sapier	1.265	1.048	0.989	0.892	1.151	0.961	0.989	1.061
Q9Y282	Endoplasmic reticulum-Golgi intermediate compartment protein 3	1.488	1.585	1.370	1.299	1.246	1.327	1.584	1.296
O43716	Glutamyl-tRNA(Gln) amidotransferase subunit C, mitochondrial O	0.888	1.195	0.555	0.735	0.722	1.054		
Q96HI4	Glycylpeptide N-tetradecanoyltransferase (Fragment) OS=Homo s	1.272	1.192	1.138	0.819	1.112	1.085	1.091	0.951
F5GX99	Caseinolytic peptidase B protein homolog OS=Homo sapiens GN=	1.737	1.848	2.108	2.041	1.812	1.540	2.097	2.384
B4DPZ3	cDNA FLJ53290, highly similar to Cytoplasmic dynein 1 intermedi	2.096	1.512	1.978	1.470	1.739	1.063	1.939	1.431
Q9H6K4	Optic atrophy 3 protein OS=Homo sapiens GN=OPA3 PE=1 SV=1	1.202	1.537	1.199	1.262	0.950	0.880	1.291	1.018
Q96F88	Processing of 1, ribonuclease P/MRP subunit (S. cerevisiae) OS=H	1.411	1.603	1.155	0.745	1.380	1.333	1.272	0.998
Q9NTI5	Sister chromatid cohesion protein PDS5 homolog B OS=Homo sap	1.210	1.585	1.238	1.042	1.094	1.234	1.318	1.244
Q9H4G4	Golgi-associated plant pathogenesis-related protein 1 OS=Homo s					0.591	1.335	0.661	
F5H8H2	Mevalonate kinase OS=Homo sapiens GN=MVK PE=1 SV=1 - [F5	1.142	0.761	1.028	1.012	0.974	0.898	0.993	0.789
Q15599	Na(+)/H(+) exchange regulatory cofactor NHE-RF2 OS=Homo sa	1.674	1.696	1.174	1.015	1.886	1.323	1.598	1.187
O95218	Zinc finger Ran-binding domain-containing protein 2 OS=Homo sa	3.239	2.715	2.182	2.168	1.846	1.215	1.128	1.706
Q92466	DNA damage-binding protein 2 OS=Homo sapiens GN=DDB2 PE=	0.947	1.140	0.669	0.581	0.957	0.967	0.923	0.964
B4DHG4	cDNA FLJ54619, highly similar to Exocyst complex component 5 (	1.587	1.169	1.540	1.385	1.409	1.079	1.430	1.215
B3KSG3	cDNA FLJ36152 fis, clone TESTI2025403, highly similar to Actin-b	1.533	1.731	1.438	1.391	1.319	1.616	1.480	1.380
P20337	Ras-related protein Rab-3B OS=Homo sapiens GN=RAB3B PE=1	2.947	3.368	2.569	1.765	1.869	1.279	1.428	2.096

Q9H0R6	Glutamyl-tRNA(Gln) amidotransferase subunit A, mitochondrial OS	1.324	1.546	0.978	1.045	1.098	1.333	1.398	1.136
Q15021	Condensin complex subunit 1 OS=Homo sapiens GN=NCAPD2 PE	1.512	1.148	1.200	0.884	1.352	1.031	1.133	0.905
O15042	U2 snRNP-associated SURP motif-containing protein OS=Homo sa	1.548	1.751	1.353	1.216	1.404	1.445	1.594	1.331
G5E9L0	ADP-ribosylation factor GTPase-activating protein 2 OS=Homo sa	1.329	0.888	1.062	0.822	1.159	0.896	1.068	0.918
Q6NUR1	Non-SMC condensin I complex, subunit G OS=Homo sapiens GN=	1.615	1.405	1.508	1.001	1.688	1.146	1.341	1.133
Q9BQA1	Methylosome protein 50 OS=Homo sapiens GN=WDR77 PE=1 SV	2.060	1.476	1.813	1.679	1.443	1.271	1.744	1.769
P43353	Aldehyde dehydrogenase family 3 member B1 OS=Homo sapiens	1.215	1.383	1.069	0.934	1.005	0.997	0.890	0.963
D5L9I5	Methyl CpG binding protein 2 transcript 1 OS=Homo sapiens GN=	1.001	1.181	0.933	0.859	0.792	1.177	1.026	0.939
P25490	Transcriptional repressor protein YY1 OS=Homo sapiens GN=YY1	1.112	1.760	1.459	1.277	0.904	1.248	1.494	1.683
O95810	Serum deprivation-response protein OS=Homo sapiens GN=SDPF	1.546	1.506	1.179	1.148	1.565	1.132	1.034	1.086
A0A087WYQ5	Putative hydrolase RBBP9 OS=Homo sapiens GN=RBBP9 PE=4 SV	1.239	1.042	1.611	1.344	1.067	1.038	1.393	1.407
Q9BY49	Peroxisomal trans-2-enoyl-CoA reductase OS=Homo sapiens GN=	1.825	1.927	2.117	1.796	1.332	1.332	1.751	1.496
Q8IYI6	Exocyst complex component 8 OS=Homo sapiens GN=EXOC8 PE	1.442	0.944	1.188	0.907	1.307	1.010	1.157	1.064
Q9BVI4	Nucleolar complex protein 4 homolog OS=Homo sapiens GN=NO	1.383	1.672	1.332	1.150	1.503	1.307	1.792	1.230
P52294	Importin subunit alpha-5 OS=Homo sapiens GN=KPNA1 PE=1 SV	1.725	1.181	1.956	1.347	1.463	1.264	1.635	1.832
Q69YN2	CWF19-like protein 1 OS=Homo sapiens GN=CWF19L1 PE=1 SV=	1.312	1.215	1.137	0.834	1.289	1.183	1.120	0.921
Q92667	A-kinase anchor protein 1, mitochondrial OS=Homo sapiens GN=	1.678	1.983	1.710	1.575	1.267	1.439	1.784	1.433
Q9ULZ3	Apoptosis-associated speck-like protein containing a CARD OS=H	1.476	1.006	1.654	1.273	1.326	0.966	1.198	1.376
O60762	Dolichol-phosphate mannosyltransferase subunit 1 OS=Homo sap	2.184	2.256	1.835	1.609	1.833	1.119	2.115	1.881
Q9Y244	Proteasome maturation protein OS=Homo sapiens GN=POMP PE=	1.688	1.010	0.788	0.463	1.512	0.870	1.018	0.512
Q9UJX2	Cell division cycle protein 23 homolog OS=Homo sapiens GN=CD	2.140	2.058	1.956	1.618	2.024	1.355	2.153	1.930
Q05DK5	ADD2 protein (Fragment) OS=Homo sapiens GN=ADD2 PE=2 SV	1.169	1.087	1.289	0.984	1.198	0.888	1.175	1.306
Q9H0A8	COMM domain-containing protein 4 OS=Homo sapiens GN=COMI	1.822	1.636	2.143	1.761	1.688	1.317	1.974	1.737
A0A087WZF1	Lipoma-preferred partner OS=Homo sapiens GN=LPP PE=4 SV=1	1.324	0.776	0.979	0.752	1.150	0.892	0.915	0.852
Q9NWU2	Glucose-induced degradation protein 8 homolog OS=Homo sapiei	2.321	1.922	1.630	1.560	2.013	1.639	1.835	1.519
O14975	Very long-chain acyl-CoA synthetase OS=Homo sapiens GN=SLC2	2.022	2.196	1.738	1.715	1.386	1.296	1.745	1.730
Q9HCU5	Prolactin regulatory element-binding protein OS=Homo sapiens G	1.329	1.435	1.301	1.172	1.055	1.185	1.342	1.153
Q9NR45	Sialic acid synthase OS=Homo sapiens GN=NANS PE=1 SV=2 - [	1.092	0.812	1.086	0.936	0.969	0.917	1.033	0.879
Q16643	Drebrin OS=Homo sapiens GN=DBN1 PE=1 SV=4 - [DREB_HUMA	1.474	1.572	1.287	1.207	1.344	1.330	1.392	1.451
P29144	Tripeptidyl-peptidase 2 OS=Homo sapiens GN=TPP2 PE=1 SV=4	1.463	1.150	1.488	1.069	1.340	1.170	1.320	1.310
I6L9H2	Cytosine-specific methyltransferase OS=Homo sapiens GN=DNMT		1.282	0.744		0.945	1.561	1.387	0.740
P22692	Insulin-like growth factor-binding protein 4 OS=Homo sapiens GN	1.392	0.761	0.481	0.205	1.404	0.962	0.420	0.202
F8WCT1	ADP-ribosylation factor-like protein 6-interacting protein 4 OS=Ho	1.195	0.884	1.311	0.610	1.555	1.236	0.886	0.638
P36507	Dual specificity mitogen-activated protein kinase kinase 2 OS=Ho	1.999	1.713	2.322	1.524	2.006	1.447	2.142	1.792
A0A087WXK9	Thyroid receptor-interacting protein 11 OS=Homo sapiens GN=TI	1.193	1.635	1.251	1.103	1.264	1.364	1.325	1.276
Q9BZE9	Tether containing UBX domain for GLUT4 OS=Homo sapiens GN=	1.369	1.189	1.370	2.039	1.770	1.257	1.700	1.024
Q9C0J8	pre-mRNA 3' end processing protein WDR33 OS=Homo sapiens G	1.163	1.420	1.025	0.797	1.141	1.350	1.270	0.977
A0A024RBC7	Calcium-transporting ATPase OS=Homo sapiens GN=ATP2B1 PE=	1.260	1.533	1.228	1.211	1.105	1.116	1.195	1.116
J3KNF4	Copper chaperone for superoxide dismutase OS=Homo sapiens G	1.392	1.788	1.849	1.950	1.474	1.189	1.988	1.911
Q9BW61	DET1- and DDB1-associated protein 1 OS=Homo sapiens GN=DD	1.312	1.338	0.989	0.642	1.308	1.292	1.146	0.928

Q9BUH6	Uncharacterized protein C9orf142 OS=Homo sapiens GN=C9orf14	1.010	0.839	1.351	1.192	1.150	0.928	0.936	0.860
Q96BK5	PIN2/TERF1-interacting telomerase inhibitor 1 OS=Homo sapiens	1.629	2.302	2.160	1.790	1.665	1.719	1.972	1.971
P28290	Sperm-specific antigen 2 OS=Homo sapiens GN=SSFA2 PE=1 SV=	1.234	1.331	0.978	1.094	1.325	1.244	1.332	1.208
Q5VYK3	Proteasome-associated protein ECM29 homolog OS=Homo sapier	1.338	1.011	1.198	0.921	1.253	1.029	1.119	0.872
F5H0U5	Glycolipid transfer protein OS=Homo sapiens GN=GLTP PE=1 SV=	1.249	1.056	1.468	1.435	1.080	0.890	1.371	1.384
Q96QD9	UAP56-interacting factor OS=Homo sapiens GN=FYTTD1 PE=1 S\	1.583	2.415	2.000	1.571	1.530	1.582	2.311	2.266
J3QRU7	Biogenesis of lysosome-related organelles complex 1 subunit 2 (F	2.133	1.957	1.452	1.294	1.841	1.867	1.867	2.076
B3KMB8	cDNA FLJ10654 fis, clone NT2RP2005901, highly similar to FAST	2.132	2.319	1.811	1.778	1.670	1.443	1.923	1.894
Q8NI27	THO complex subunit 2 OS=Homo sapiens GN=THOC2 PE=1 SV=	1.361	1.609	1.397	1.002	1.181	1.282	1.291	1.194
A0A024R0A9	Stromal cell derived factor 4, isoform CRA_b OS=Homo sapiens G	1.400	1.322	1.321	1.325	1.063	1.114	1.262	1.253
B2R5N4	cDNA, FLJ92544, highly similar to Homo sapiens lectin, mannose-	1.901	2.008	1.779	1.645	1.447	1.354	1.559	1.544
Q13188	Serine/threonine-protein kinase 3 OS=Homo sapiens GN=STK3 P	1.869	1.398	1.604	1.172	1.473	1.172	1.801	1.648
A8K6X9	cDNA FLJ76427, highly similar to Homo sapiens SH2 domain bind	1.583	1.926	1.455	1.201	1.399	1.494	1.460	1.338
B5MC22	Mitochondrial fission process protein 1 OS=Homo sapiens GN=MT	1.387	1.627	1.117	1.104	0.997	1.114	1.312	1.219
C9JBI3	Phosphoserine phosphatase (Fragment) OS=Homo sapiens GN=F	1.404	0.878	1.249	0.969	1.204	0.889	1.107	1.088
A0A087X0G7	NF-kappa-B essential modulator OS=Homo sapiens GN=IKBKG PI	1.532	1.077	1.285	0.998	1.509	1.275	1.158	1.219
Q05BM8	GALNT1 protein OS=Homo sapiens GN=GALNT1 PE=2 SV=1 - [Q	1.270	1.353	1.134	1.091	1.002	1.291	1.132	1.090
Q00403	Transcription initiation factor IIB OS=Homo sapiens GN=GTF2B P	1.342	1.244	1.188	0.811	1.241	1.327	1.158	0.973
A0A024R972	Laminin, gamma 1 (Formerly LAMB2), isoform CRA_a OS=Homo	1.602	1.654	1.326	1.217	1.378	1.294	1.681	1.502
Q15269	Periodic tryptophan protein 2 homolog OS=Homo sapiens GN=PV	1.724	1.963	1.735	1.781	1.537	1.370	1.982	1.711
Q9UQ35	Serine/arginine repetitive matrix protein 2 OS=Homo sapiens GN=	1.546	1.558	1.271	0.941	1.548	1.508	1.502	1.203
Q9NWX6	Probable tRNA(His) guanylyltransferase OS=Homo sapiens GN=T	1.625	1.395	1.519	1.463	1.384	1.285	1.710	1.601
Q969H8	UPF0556 protein C19orf10 OS=Homo sapiens GN=C19orf10 PE=	2.543	1.557	1.644	1.484	1.873	1.180	1.634	2.514
Q8TDQ7	Glucosamine-6-phosphate isomerase 2 OS=Homo sapiens GN=GI	1.407	1.093	1.612	1.238	1.044	1.194	1.266	1.197
X5DR54	NudE neurodevelopment protein 1 isoform B (Fragment) OS=Hor	1.135	1.019	0.796	0.607	1.023	1.009	0.863	0.623
P23141	Liver carboxylesterase 1 OS=Homo sapiens GN=CES1 PE=1 SV=2	1.689	1.273	1.736	1.878	1.688	1.334	1.726	1.754
B7Z695	cDNA FLJ52742, highly similar to Periphilin-1 OS=Homo sapiens I		1.353				1.003	0.918	
Q6DKJ4	Nucleoredoxin OS=Homo sapiens GN=NXN PE=1 SV=2 - [NXN_H	1.218	0.868	1.035	0.823	1.293	1.165	1.032	0.938
Q86UA3	Chromosome 12 open reading frame 10 OS=Homo sapiens GN=C	1.193	0.912	1.271	1.012	1.116	1.026	1.059	1.100
Q9H7L9	Sin3 histone deacetylase corepressor complex component SDS3 C	1.604	1.759	1.081	0.722	1.604	1.224	1.412	1.019
O95721	Synaptosomal-associated protein 29 OS=Homo sapiens GN=SNAI	1.244	1.159	1.404	1.246	1.148	1.208	1.436	1.409
Q14353	Guanidinoacetate N-methyltransferase OS=Homo sapiens GN=GA	1.284	1.111	1.539	1.390	1.297	0.884	1.354	1.202
Q8N7H5	RNA polymerase II-associated factor 1 homolog OS=Homo sapier	1.845	1.802	1.574	1.133	1.810	1.559	1.739	1.355
Q8IWA0	WD repeat-containing protein 75 OS=Homo sapiens GN=WDR75	1.567	1.716	1.425	1.510	1.589	1.404	1.798	1.708
Q14669	E3 ubiquitin-protein ligase TRIP12 OS=Homo sapiens GN=TRIP1	1.196	1.230	1.018	0.779	0.987	1.135	1.123	0.858
Q6PKG0	La-related protein 1 OS=Homo sapiens GN=LARP1 PE=1 SV=2 -	1.436	1.541	1.362	1.018	1.384	1.173	1.344	1.054
Q8NHH9	Atlastin-2 OS=Homo sapiens GN=ATL2 PE=1 SV=2 - [ATLA2_HUI	1.512	1.737	1.350	1.312	1.281	1.454	1.515	1.411
Q13769	THO complex subunit 5 homolog OS=Homo sapiens GN=THOC5	1.634	1.559	1.363	1.287	1.480	1.315	1.633	1.608
Q96EY4	Translation machinery-associated protein 16 OS=Homo sapiens G	1.303	1.554	1.233	1.066	1.379	1.329	1.350	1.598
J3KQ45	Trans-Golgi network integral membrane protein 2 OS=Homo sapi	1.528	1.513	1.002	0.978	1.427	1.281	1.460	1.095

O14776	Transcription elongation regulator 1 OS=Homo sapiens GN=TCER	1.270	1.451	1.388	0.862	1.108	1.247	1.101	0.978
P40763	Signal transducer and activator of transcription 3 OS=Homo sapie	1.289	0.902	1.074	0.780	1.295	0.924	1.034	0.943
Q9UN37	Vacuolar protein sorting-associated protein 4A OS=Homo sapiens	1.858	1.291	2.004	1.660	1.577	1.292	2.185	1.616
Q587I9	Vesicle transport protein SFT2C OS=Homo sapiens GN=SFT2D3 F	1.902	1.903	1.552	1.307	1.682	1.590	1.431	1.312
Q58EY4	SWI/SNF related, matrix associated, actin dependent regulator of	1.322	1.347	1.044	0.735	1.565	1.443	1.266	1.020
Q6P392	PPIL4 protein (Fragment) OS=Homo sapiens GN=PPIL4 PE=2 SV	1.472	1.703	1.395	1.089	1.608	1.302	1.423	1.168
Q9UI10	Translation initiation factor eIF-2B subunit delta OS=Homo sapier	1.321	1.067	1.139	0.862	1.051	1.107	1.169	0.991
Q149P0	GBF1 protein OS=Homo sapiens GN=GBF1 PE=2 SV=1 - [Q149P(	1.594	1.203	1.324	1.085	1.394	1.212	1.268	1.049
E9PMI6	Methylosome subunit pICln OS=Homo sapiens GN=CLNS1A PE=1	1.354	0.958	0.857	1.038	1.483	1.347	1.609	1.332
A7MD96	SYNPO protein (Fragment) OS=Homo sapiens GN=SYNPO PE=2 S	2.097	2.165	1.777	1.905	1.651	1.689	1.306	2.278
B7Z1U1	cDNA FLJ60457, highly similar to NADH-ubiquinone oxidoreducta:	1.127	1.325	1.058	1.014	0.871	1.064	1.099	0.990
Q9H501	ESF1 homolog OS=Homo sapiens GN=ESF1 PE=1 SV=1 - [ESF1_	1.476	1.660	1.369	1.144	1.486	1.454	1.479	1.276
Q6IN84	rRNA methyltransferase 1, mitochondrial OS=Homo sapiens GN=	1.433	1.564	1.390	1.309	1.004	1.199	1.436	1.518
Q8NBJ7	Sulfatase-modifying factor 2 OS=Homo sapiens GN=SUMF2 PE=1	1.195	0.835	0.973	1.032	1.081	1.043	0.991	1.004
B4DEY8	cDNA FLJ60452, highly similar to Cytochrome c oxidase assembly	11.871	11.915	10.237	10.013	4.883	2.248	7.884	11.169
H3BUT5	Nuclear envelope phosphatase-regulatory subunit 1 OS=Homo sa	1.040	0.935	0.577	0.539	0.972	1.219	0.830	0.557
Q8ND82	Zinc finger protein 280C OS=Homo sapiens GN=ZNF280C PE=1 S	1.418	1.649	1.314	1.185	1.180	1.429	1.368	1.416
O15379	Histone deacetylase 3 OS=Homo sapiens GN=HDAC3 PE=1 SV=2	1.158	1.255	1.001	0.818	1.156	1.130	1.174	1.171
Q6MZZ4	Putative uncharacterized protein DKFZp686H0575 OS=Homo sapi	0.919	0.775	0.323	0.346	0.836	0.832	0.683	0.520
Q06587	E3 ubiquitin-protein ligase RING1 OS=Homo sapiens GN=RING1	1.637	1.830	1.924	1.216	1.555	1.842	1.879	1.618
Q68CZ2	Tensin-3 OS=Homo sapiens GN=TNS3 PE=1 SV=2 - [TENS3_HUI	1.392	0.794	0.873	0.650	1.125	0.834	0.724	0.656
C9JLU1	DNA-directed RNA polymerases I, II, and III subunit RPABC3 (Fra	1.593	1.716	1.604	1.484	1.755	1.180	1.785	1.686
B2RDJ6	Probable cytosolic iron-sulfur protein assembly protein CIAO1 OS	1.776	1.428	1.670	1.305	1.637	1.252	1.684	1.396
Q6P1K2	Polyamine-modulated factor 1 OS=Homo sapiens GN=PMF1 PE=1	2.440	2.728	2.587	2.140	2.358	1.644	2.841	2.331
Q7L576	Cytoplasmic FMR1-interacting protein 1 OS=Homo sapiens GN=C	1.885	1.519	1.609	1.344	1.608	1.125	1.461	1.459
C9J4X5	Inhibitor of growth protein 2 (Fragment) OS=Homo sapiens GN=	2.578	2.124	2.096	1.418	1.805	1.413	1.741	1.455
Q9BYV8	Centrosomal protein of 41 kDa OS=Homo sapiens GN=CEP41 PE=	1.103	1.066	1.031	0.776	1.187	1.293	1.220	0.898
B2RDG1	cDNA, FLJ96593 OS=Homo sapiens PE=2 SV=1 - [B2RDG1_HUM	1.237	1.307	0.937	0.834	1.056	1.096	1.114	0.814
Q8IUD2	ELKS/Rab6-interacting/CAST family member 1 OS=Homo sapiens	1.617	1.208	1.596	1.439	1.383	1.147	1.478	1.512
B2R6S4	cDNA, FLJ93089, highly similar to Homo sapiens NCK adaptor prc	1.646	0.980	1.205	1.067	1.362	1.062	1.205	1.075
Q4VXZ8	Peroxisomal 2,4-dienoyl-CoA reductase OS=Homo sapiens GN=D	1.567	1.832	1.703	1.491	1.531	1.236	1.462	1.491
Q04446	1,4-alpha-glucan-branching enzyme OS=Homo sapiens GN=GBE1	1.335	1.103	1.534	1.326	1.140	0.937	1.330	1.316
Q86TU7	Histone-lysine N-methyltransferase setd3 OS=Homo sapiens GN=	1.750	1.506	1.618	0.977	1.243	1.157	1.454	1.218
Q53F37	SAR1a gene homolog 2 variant (Fragment) OS=Homo sapiens PE	1.576	1.117	1.383	1.060	1.487	1.168	1.469	1.218
O95470	Sphingosine-1-phosphate lyase 1 OS=Homo sapiens GN=SGPL1 I	1.257	1.229	1.014	0.995	0.982	1.152	1.284	0.965
P60983	Glia maturation factor beta OS=Homo sapiens GN=GMFB PE=1 S	1.029	0.714	1.098	0.954	0.913	0.932	0.929	0.888
Q96DP0	cDNA FLJ31479 fis, clone NT2NE2001634, moderately similar to I	1.170	1.453	0.959	1.075	0.913	1.049	1.116	1.116
Q12769	Nuclear pore complex protein Nup160 OS=Homo sapiens GN=NU	1.631	1.622	1.584	1.698	1.600	1.386	1.820	1.838
Q9H9A6	Leucine-rich repeat-containing protein 40 OS=Homo sapiens GN=	1.266	0.941	1.305	1.029	1.192	1.014	1.276	1.103
Q9UJW0	Dynactin subunit 4 OS=Homo sapiens GN=DCTN4 PE=1 SV=1 - [	1.328	1.044	1.262	0.966	1.241	1.192	1.358	1.078

P63165	Small ubiquitin-related modifier 1 OS=Homo sapiens GN=SUMO1	1.214	1.056	0.762	0.822	1.338	0.961	0.975	1.154
Q9H074	Polyadenylate-binding protein-interacting protein 1 OS=Homo sap	1.644	1.197	1.664	1.147	1.425	0.943	1.335	1.276
Q9UBV2	Protein sel-1 homolog 1 OS=Homo sapiens GN=SEL1L PE=1 SV=	1.414	1.220	1.039	1.165	1.215	1.241	1.312	0.960
Q16630	Cleavage and polyadenylation specificity factor subunit 6 OS=Hor	1.186	1.332	0.946	0.734	1.231	1.259	1.075	0.873
O43752	Syntaxin-6 OS=Homo sapiens GN=STX6 PE=1 SV=1 - [STX6_HU	2.174	2.103	1.460	1.918	1.550	0.970	1.754	1.769
O00479	High mobility group nucleosome-binding domain-containing prote	1.061	1.090	0.971	0.705	1.009	0.950	0.934	0.897
I6L975	Hydroxysteroid dehydrogenase like 1 OS=Homo sapiens GN=HSD	1.317	1.469	1.068	1.072	1.108	1.234	1.346	1.165
A8K5B6	cDNA FLJ76830, highly similar to Homo sapiens SWI/SNF related,	1.211	1.335	0.657	0.305	1.177	1.108	0.931	0.490
Q8IV48	3'-5' exoribonuclease 1 OS=Homo sapiens GN=ERI1 PE=1 SV=3	0.914	0.789	0.803	0.679	1.032	1.102	0.852	0.789
B7Z5P4	cDNA FLJ55542, highly similar to Ran-binding protein 3 OS=Hom	1.342	1.435	1.423	0.994	1.519	1.400	1.283	1.120
P46108	Adapter molecule crk OS=Homo sapiens GN=CRK PE=1 SV=2 - [	1.320	0.917	1.168	1.164	1.227	0.926	1.191	1.084
Q96FX7	tRNA (adenine(58)-N(1))-methyltransferase catalytic subunit TRM	0.612	0.582	0.821	0.447	0.941	1.018	0.627	0.515
O75691	Small subunit processome component 20 homolog OS=Homo sap	1.798	2.209	1.744	1.547	1.598	1.509	1.969	1.462
A0A024RD08	Mitochondrial carrier homolog 1 (C. elegans), isoform CRA_d OS=	2.565	2.774	2.394	2.265	1.143	1.220	1.431	1.902
B4DUI2	cDNA FLJ61415, highly similar to Protein kinase C and casein kina	1.243	1.378	1.023	0.870	1.124	1.135	1.318	1.173
Q8N806	Putative E3 ubiquitin-protein ligase UBR7 OS=Homo sapiens GN=	1.279	1.274	1.253	0.876	1.525	1.278	1.264	1.134
Q9NRP2	COX assembly mitochondrial protein 2 homolog OS=Homo sapier	1.561	1.356	1.317	1.365	1.395	1.298	1.441	1.302
Q9BV20	Methylthioribose-1-phosphate isomerase OS=Homo sapiens GN=	0.905	0.867	1.095	1.097	1.192	0.814	1.092	1.172
B6VEX5	Abl-interactor 1 variant 72 OS=Homo sapiens PE=2 SV=1 - [B6VI	1.351	0.889	1.141	0.974	1.122	1.027	1.147	1.156
Q13610	Periodic tryptophan protein 1 homolog OS=Homo sapiens GN=PV	2.522	3.003	2.003	1.478	2.405	1.737	1.770	1.682
Q96T51	RUN and FYVE domain-containing protein 1 OS=Homo sapiens GI	1.434	1.316	1.934	1.480	1.530	1.207	1.604	1.674
Q6P2E9	Enhancer of mRNA-decapping protein 4 OS=Homo sapiens GN=E	1.647	1.882	1.799	1.641	1.581	1.528	1.853	1.640
B2R673	cDNA, FLJ92818, highly similar to Homo sapiens pyruvate dehydr	1.157	1.193	0.935	0.995	0.957	1.119	1.124	1.061
Q9BSF4	Uncharacterized protein C19orf52 OS=Homo sapiens GN=C19orf!	1.488	1.804	0.973	0.862	1.020	1.160	1.244	1.017
P51580	Thiopurine S-methyltransferase OS=Homo sapiens GN=TPMT PE=	1.814	1.081	1.799	1.544	1.782	1.159	1.527	1.429
Q9BXS6	Nucleolar and spindle-associated protein 1 OS=Homo sapiens GN	1.096	1.601	0.871	0.680	0.983	1.005	1.049	1.176
O00186	Syntaxin-binding protein 3 OS=Homo sapiens GN=STXBP3 PE=1	1.424	1.614	1.349	1.210	1.264	1.385	1.439	1.419
B3KPJ4	Polyhomeotic-like protein 2 OS=Homo sapiens GN=PHC2 PE=1 S	1.621	1.728	1.158	1.032	1.636	1.310	1.588	1.396
P46734	Dual specificity mitogen-activated protein kinase kinase 3 OS=Ho	1.372	0.834	1.187	0.750	1.079	1.089	1.021	0.909
Q9NVH1	DnaJ homolog subfamily C member 11 OS=Homo sapiens GN=DI	1.548	1.502	1.242	1.363	1.335	1.276	1.535	1.311
B2R7X3	cDNA, FLJ93645, highly similar to Homo sapiens chromatin assen	1.278	1.800	1.493	1.369	1.304	1.628	2.096	1.853
D6RIY6	Exosome complex component RRP45 OS=Homo sapiens GN=EXC	1.630	1.662	1.353	1.180	1.606	1.336	1.662	1.458
Q8NFH9	MLL/SEPTIN6 fusion protein (Fragment) OS=Homo sapiens PE=2	1.301	0.822	0.841	0.642	1.144	0.830	0.846	0.706
B7Z731	Glucosylceramidase OS=Homo sapiens PE=2 SV=1 - [B7Z731_HU	1.309	1.145	1.190	1.244	1.423	1.194	1.310	1.318
Q13243	Serine/arginine-rich splicing factor 5 OS=Homo sapiens GN=SRSI	2.544	2.860	5.252	1.734	2.216	1.669	2.047	2.153
P62253	Ubiquitin-conjugating enzyme E2 G1 OS=Homo sapiens GN=UBE	1.992	1.498	2.139	1.543	1.936	1.290	1.744	1.574
E7EVA0	Microtubule-associated protein OS=Homo sapiens GN=MAP4 PE=	1.321	1.010	1.070	0.801	1.021	0.948	1.070	0.949
A8K548	cDNA FLJ75008, highly similar to Homo sapiens proline-, glutamic	1.341	1.158	1.024	0.790	1.305	1.271	1.188	0.899
Q99666	RANBP2-like and GRIP domain-containing protein 5/6 OS=Homo	1.347	2.105	1.391	1.460	1.536	1.400	1.642	1.664
P78362	SRSF protein kinase 2 OS=Homo sapiens GN=SRPK2 PE=1 SV=3	1.600	1.482	1.597	1.063	1.873	1.238	1.782	1.396

Q96DB5	Regulator of microtubule dynamics protein 1 OS=Homo sapiens (	1.316	1.346	1.217	1.293	1.371	1.409	1.306	1.388
Q9UII2	ATPase inhibitor, mitochondrial OS=Homo sapiens GN=ATPIF1 PE	2.365	2.141	1.996	2.452	1.696	1.172	2.176	1.879
Q63HN8	E3 ubiquitin-protein ligase RNF213 OS=Homo sapiens GN=RNF21	1.370	1.044	1.088	0.777	1.306	1.026	1.043	0.843
Q7Z2E3	Aprataxin OS=Homo sapiens GN=APTX PE=1 SV=2 - [APTX_HUM	1.470	1.684	1.368	1.214	1.562	1.269	1.566	1.614
B7Z307	cDNA FLJ55028, highly similar to Transmembrane protein 66 OS=	1.211	1.263	0.818	0.579	1.022	1.210	1.178	0.753
B7Z6C9	cDNA FLJ61658, highly similar to Transmembrane 9 superfamily p	1.329	1.357	1.354	1.371	1.248	1.179	1.467	1.209
P05165	Propionyl-CoA carboxylase alpha chain, mitochondrial OS=Homo	1.347	1.438	1.305	1.371	0.999	1.174	1.396	1.256
A8K9T3	cDNA FLJ76467, highly similar to Homo sapiens acyl-CoA syntheta	1.566	1.960	1.909	1.798	1.403	1.369	1.712	1.786
B3KN09	cDNA FLJ13173 fis, clone NT2RP3003831, highly similar to Endor	1.889	3.164	2.316	2.529	1.768	2.026	2.464	2.289
Q8NFV4	Alpha/beta hydrolase domain-containing protein 11 OS=Homo sa	1.196	1.424	1.103	1.104	1.392	1.033	1.223	1.180
K7EIJ0	WW domain-binding protein 2 (Fragment) OS=Homo sapiens GN=	1.612	1.073	1.418	1.103	1.435	1.067	1.365	1.214
B4E321	Olfactory receptor 7D2 OS=Homo sapiens GN=OR7D2 PE=2 SV=	1.155	0.927	0.778	0.653	0.868	0.992	1.065	0.989
B2RA70	cDNA, FLJ94729, highly similar to Homo sapiens v-yes-1 Yamagu	1.241	1.411	1.313	1.151	1.124	1.458	1.139	1.262
B3KN52	cDNA FLJ13606 fis, clone PLACE1010579, highly similar to Homo	1.422	1.130	1.149	0.877	1.032	0.970	1.005	1.072
O00399	Dynactin subunit 6 OS=Homo sapiens GN=DCTN6 PE=1 SV=1 -	1.883	1.123	1.473	1.268	1.368	1.325	1.613	1.415
Q9NUG6	p53 and DNA damage-regulated protein 1 OS=Homo sapiens GN=	1.369	1.089	1.244	1.074	1.087	1.138	1.193	1.205
Q8WZA0	Protein LZIC OS=Homo sapiens GN=LZIC PE=1 SV=1 - [LZIC_HU	1.271	0.986	1.379	1.192	1.104	1.083	1.243	1.297
A8K6J9	cDNA FLJ76879, highly similar to Homo sapiens DEAH (Asp-Glu-A	1.536	1.449	1.242	0.940	1.458	1.425	1.296	1.070
A8K0B9	cDNA FLJ75988, highly similar to Homo sapiens transcription fact	1.184	1.233	0.874	0.852	0.985	1.069	1.023	1.131
Q53T59	HCLS1-binding protein 3 OS=Homo sapiens GN=HS1BP3 PE=1 S	1.161	0.858	1.263	0.996	0.981	0.941	0.985	1.122
Q59FC2	Acyl-Coenzyme A dehydrogenase family, member 8 variant (Fragr	0.770	1.007	0.972	0.437	0.721	1.092	0.733	0.845
P50440	Glycine amidinotransferase, mitochondrial OS=Homo sapiens GN=	1.480	1.185	1.384	1.702	0.957	1.207	2.039	1.563
Q9UK41	Vacuolar protein sorting-associated protein 28 homolog OS=Hom	1.450	0.844	1.048	0.999	1.264	0.983	1.167	1.009
Q8WUA4	General transcription factor 3C polypeptide 2 OS=Homo sapiens (	1.313	1.551	1.256	1.048	1.349	1.320	1.498	1.554
Q49A26	Putative oxidoreductase GLYR1 OS=Homo sapiens GN=GLYR1 PE	1.683	2.039	1.795	1.559	1.472	1.323	1.749	1.711
Q9BZF1	Oxysterol-binding protein-related protein 8 OS=Homo sapiens GN	1.455	1.404	1.376	1.128	1.168	1.192	1.460	1.435
Q8NCC3	Group XV phospholipase A2 OS=Homo sapiens GN=PLA2G15 PE=	2.021	1.695	2.475	2.511	1.716	1.230	1.758	2.456
Q14692	Ribosome biogenesis protein BMS1 homolog OS=Homo sapiens G	1.333	1.544	1.195	1.129	1.214	1.331	1.507	1.127
Q7Z5G3	Acetyl-coenzyme A synthetase OS=Homo sapiens GN=ACSS1 PE=	1.288	1.204	1.397	1.229	1.058	1.238	1.253	1.179
Q9GZS1	DNA-directed RNA polymerase I subunit RPA49 OS=Homo sapien	1.337	1.822	1.510	1.305	1.301	1.375	1.462	1.265
P46937	Transcriptional coactivator YAP1 OS=Homo sapiens GN=YAP1 PE=	1.309	0.691	1.066	0.773	1.152	0.938	0.891	0.854
B2RAG5	cDNA, FLJ94903, highly similar to Homo sapiens glyceronephospl	1.767	2.105	1.834	1.511	1.486	1.285	1.592	1.524
Q8NI36	WD repeat-containing protein 36 OS=Homo sapiens GN=WDR36	1.628	1.821	1.555	1.425	1.432	1.358	1.572	1.515
B3KNC3	cDNA FLJ14222 fis, clone NT2RP3003992, highly similar to Nucle	1.509	1.698	1.517	1.613	1.379	1.302	1.695	1.698
P00813	Adenosine deaminase OS=Homo sapiens GN=ADA PE=1 SV=3 -	1.027	0.749	1.016	0.904	0.932	0.859	0.903	0.914
E7EW49	CLIP-associating protein 2 OS=Homo sapiens GN=CLASP2 PE=1	1.379	1.029	1.402	1.026	1.136	0.818	1.175	1.137
A8K0F7	cDNA FLJ76587, highly similar to Homo sapiens vitamin K epoxide	2.060	2.098	1.759	1.780	1.400	1.337	1.775	1.686
Q8N0Z6	Tetratricopeptide repeat protein 5 OS=Homo sapiens GN=TTC5 P	1.298	1.013	1.773	1.051	1.250	1.042	1.444	1.281
Q5UIP0	Telomere-associated protein RIF1 OS=Homo sapiens GN=RIF1 PE	1.489	1.865	1.510	1.182	1.106	1.463	1.618	1.263
X5CF57	BRD2 OS=Homo sapiens GN=BRD2 PE=4 SV=1 - [X5CF57_HUM/	1.417	2.667	2.067	2.156	1.285	2.113	2.291	2.273

B3KU62	cDNA FLJ39243 fis, clone OCBBF2008283, highly similar to Protei	1.172	0.909	1.214	1.025	1.091	0.952	1.012	0.864
Q9NT62	Ubiquitin-like-conjugating enzyme ATG3 OS=Homo sapiens GN=A	1.447	0.965	1.451	1.250	1.203	1.090	1.424	1.462
Q9Y3E0	Vesicle transport protein GOT1B OS=Homo sapiens GN=GOLT1B		0.242			0.200	0.844	0.221	0.178
Q9UMY1	Nucleolar protein 7 OS=Homo sapiens GN=NOL7 PE=1 SV=2 - [N	1.170	1.414	0.556	0.376	1.208	1.183	0.952	0.540
E7EW18	DNA polymerase beta (Fragment) OS=Homo sapiens GN=POLB P	1.561	2.063	2.018	1.871	1.463	1.416	1.924	1.955
Q8NFF5	FAD synthase OS=Homo sapiens GN=FLAD1 PE=1 SV=1 - [FAD1	1.924	1.674	1.696	1.683	1.340	1.239	1.557	1.807
O15392	Baculoviral IAP repeat-containing protein 5 OS=Homo sapiens GN	1.715	1.694	1.182	1.207	1.616	1.491	1.629	1.520
Q9H0C8	Integrin-linked kinase-associated serine/threonine phosphatase 2	1.112	0.949	1.099	0.939	1.146	1.190	1.038	0.906
Q8TCT9	Minor histocompatibility antigen H13 OS=Homo sapiens GN=HM1	1.903	1.970	1.787	1.876	1.572	1.393	2.054	1.650
Q10570	Cleavage and polyadenylation specificity factor subunit 1 OS=Hor	1.425	1.539	1.273	1.069	1.427	1.348	1.544	1.333
Q32MZ4	Leucine-rich repeat flightless-interacting protein 1 OS=Homo sapi	1.646	1.324	1.520	1.053	1.366	1.252	1.227	1.307
Q9NVV4	Poly(A) RNA polymerase, mitochondrial OS=Homo sapiens GN=M	1.767	1.976	1.457	1.420	1.472	1.263	1.605	1.627
Q14966	Zinc finger protein 638 OS=Homo sapiens GN=ZNF638 PE=1 SV=	1.157	1.680	1.170	1.016	1.023	1.299	1.366	1.156
Q7L9L4	MOB kinase activator 1B OS=Homo sapiens GN=MOB1B PE=1 SV	1.593	1.164	1.707	1.295	1.395	1.104	1.594	1.317
P49770	Translation initiation factor eIF-2B subunit beta OS=Homo sapien	1.081	0.988	1.314	0.805	0.970	1.115	1.152	1.003
Q14249	Endonuclease G, mitochondrial OS=Homo sapiens GN=ENDOG PI	1.264	1.429	0.999	0.635	1.062	1.296	1.172	0.851
A8K7H4	cDNA FLJ75764, highly similar to Homo sapiens origin recognitior	1.440	1.708	1.579	1.226	1.436	1.390	1.547	1.565
M0R0B4	KxDL motif-containing protein 1 (Fragment) OS=Homo sapiens G	1.185	0.946	1.147	1.180	1.219		0.882	1.350
A8MXV4	Nucleoside diphosphate-linked moiety X motif 19, mitochondrial C	1.742	1.697	1.556	1.637	1.027	1.415	1.996	1.582
O00203	AP-3 complex subunit beta-1 OS=Homo sapiens GN=AP3B1 PE=1	1.469	0.981	1.355	1.006	1.468	1.011	1.303	1.051
A8K6N9	cDNA FLJ77153, highly similar to Homo sapiens calcium/calmodu	1.266	1.158	0.883	0.601	1.444	1.283	1.121	1.150
Q9HBL7	Plasminogen receptor (KT) OS=Homo sapiens GN=PLGRKT PE=1	0.921	1.232	0.967	1.061	0.918	1.086	1.181	1.059
Q6PL18	ATPase family AAA domain-containing protein 2 OS=Homo sapier	1.632	2.590	1.480	0.684	1.581	1.845	2.530	1.322
B3KMH8	Autophagy protein 5 OS=Homo sapiens PE=2 SV=1 - [B3KMH8_I	1.370	0.878	1.235	0.883	1.071	1.051	1.150	1.013
F8VU90	Peptidyl-prolyl cis-trans isomerase OS=Homo sapiens GN=FKBP1	2.486	2.015	2.649	2.577	1.332	1.328	1.811	1.727
P41134	DNA-binding protein inhibitor ID-1 OS=Homo sapiens GN=ID1 PE	1.229	0.505			1.175	0.910	0.440	
O95169	NADH dehydrogenase [ubiquinone] 1 beta subcomplex subunit 8,	1.270	1.383	1.068	1.108	0.897	1.104	1.227	1.337
Q15042	Rab3 GTPase-activating protein catalytic subunit OS=Homo sapie	1.359	1.189	1.467	1.037	1.355	1.163	1.459	1.217
Q2KRZ4	E3 14.7 kDa protein GN=E3_14.7 - [Q2KRZ4_ADE05]	3.792	6.077	13.083	13.134	1.925	0.919	1.906	
C9J384	Protein CMSS1 (Fragment) OS=Homo sapiens GN=CMSS1 PE=1 S	1.479	1.752	1.475	1.299	1.605	1.522	1.576	1.525
P07305	Histone H1.0 OS=Homo sapiens GN=H1F0 PE=1 SV=3 - [H10_HU	1.177	2.178	1.130	1.165	1.044	0.895	0.858	1.249
Q9Y324	rRNA-processing protein FCF1 homolog OS=Homo sapiens GN=F(	0.983	1.167	0.749	0.523	0.984	1.136	1.046	0.636
Q8N9T8	Protein KRI1 homolog OS=Homo sapiens GN=KRI1 PE=1 SV=3 -	1.401	1.440	1.234	1.110	1.245	1.295	1.374	1.197
P17301	Integrin alpha-2 OS=Homo sapiens GN=ITGA2 PE=1 SV=1 - [ITA	1.057	1.427	1.073	1.104	1.239	1.170	0.997	1.099
B2RDU9	cDNA, FLJ96775 OS=Homo sapiens PE=2 SV=1 - [B2RDU9_HUM	2.332	2.754	2.022	2.592	2.114	2.786	3.131	2.637
O95239	Chromosome-associated kinesin KIF4A OS=Homo sapiens GN=KI	1.279	1.256	1.043	0.938	1.087	1.136	1.189	1.129
Q86UY8	5'-nucleotidase domain-containing protein 3 OS=Homo sapiens G	1.711	1.796	1.403	1.412	1.407	1.313	1.574	1.487
A6NEM2	HCF N-terminal chain 5 OS=Homo sapiens GN=HCFC1 PE=1 SV=	1.193	1.223	0.904	0.656	1.198	1.208	1.134	0.940
Q9UQR0	Sex comb on midleg-like protein 2 OS=Homo sapiens GN=SCML2	1.353	1.721	1.433	1.245	1.463	1.489	1.670	1.233
Q92621	Nuclear pore complex protein Nup205 OS=Homo sapiens GN=NU	1.720	1.763	1.714	1.754	1.582	1.479	1.905	1.859

Q8ND56	Protein LSM14 homolog A OS=Homo sapiens GN=LSM14A PE=1	1.753	1.747	1.689	1.398	1.815	1.432	1.980	1.713
Q6P158	Putative ATP-dependent RNA helicase DHX57 OS=Homo sapiens	1.490	1.399	1.649	1.159	1.432	1.320	1.712	1.745
A0A024R7K0	Splicing factor, arginine/serine-rich 14, isoform CRA_a OS=Homo	1.417	1.356	1.011	0.668	1.372	1.267	1.231	1.053
K7EQ02	DAZ-associated protein 1 (Fragment) OS=Homo sapiens GN=DAZ	0.806	0.913	0.718	0.452	0.968	0.962	0.658	0.714
F8W7Q4	Protein FAM162A OS=Homo sapiens GN=FAM162A PE=1 SV=1 -	1.623	1.523	1.494	1.550	1.226	0.836	1.477	1.867
Q9BZX2	Uridine-cytidine kinase 2 OS=Homo sapiens GN=UCK2 PE=1 SV=	1.271	1.162	1.421	0.900	1.191	1.424	1.357	0.924
Q9H6S0	Probable ATP-dependent RNA helicase YTHDC2 OS=Homo sapien	1.317	1.264	1.210	1.089	1.227	1.071	1.285	1.091
J3KQ34	COP9 signalosome complex subunit 7b OS=Homo sapiens GN=C(	2.989	1.976	2.933	1.909	1.630	1.380	1.939	1.626
Q96HW7	Integrator complex subunit 4 OS=Homo sapiens GN=INTS4 PE=1	1.663	1.906	1.326	1.189	1.793	1.400	1.685	1.324
Q14WI3	NADH-ubiquinone oxidoreductase chain 1 (Fragment) OS=Homo	1.375	1.188	1.297	0.980	1.030	0.950	1.224	1.163
Q76FK4	Nucleolar protein 8 OS=Homo sapiens GN=NOL8 PE=1 SV=1 - [N	1.661	1.806	1.389	1.284	1.465	1.421	1.751	1.443
Q5T8P6	RNA-binding protein 26 OS=Homo sapiens GN=RBM26 PE=1 SV=	1.195	1.471	1.042	0.785	1.186	1.228	1.232	1.015
Q96LZ7	Regulator of microtubule dynamics protein 2 OS=Homo sapiens (	1.953	2.289	2.065	1.778	1.589	1.660	2.317	1.932
O60292	Signal-induced proliferation-associated 1-like protein 3 OS=Homo	1.570	1.811	1.072	0.849	1.349	1.319	1.296	0.987
Q13148	TAR DNA-binding protein 43 OS=Homo sapiens GN=TARDBP PE=	1.053	1.371	0.791	0.583	1.156	1.170	0.920	0.698
O95340	Bifunctional 3'-phosphoadenosine 5'-phosphosulfate synthase 2 C	1.259	1.078	1.388	0.876	1.214	1.060	1.136	0.954
Q7L523	Ras-related GTP-binding protein A OS=Homo sapiens GN=RRAGA	2.154	1.778	1.540	1.385	1.994	1.243	1.948	1.768
A8CDT9	MYO5A variant protein OS=Homo sapiens PE=2 SV=1 - [A8CDT9	1.370	1.314	1.251	1.075	1.320	1.316	1.479	1.435
B2R841	Serine/threonine-protein kinase PLK OS=Homo sapiens PE=2 SV=	1.564	1.785	1.382	0.903	1.201	1.369	1.640	1.243
Q8NFQ8	Torsin-1A-interacting protein 2 OS=Homo sapiens GN=TOR1AIP2	1.349	1.263	1.103	1.101	1.105	1.289	1.538	1.331
Q9HA77	Probable cysteine--tRNA ligase, mitochondrial OS=Homo sapiens	1.175	1.029	1.008	1.039	0.969	1.215	1.120	1.131
Q6P1L8	39S ribosomal protein L14, mitochondrial OS=Homo sapiens GN=	1.700	1.692	1.332	1.038	1.106	1.115	1.353	1.330
Q4G176	Acyl-CoA synthetase family member 3, mitochondrial OS=Homo s	1.597	1.588	1.457	1.419	1.056	1.192	1.426	1.508
Q15942	Zyxin OS=Homo sapiens GN=ZYX PE=1 SV=1 - [ZYX_HUMAN]	1.161	0.799	0.922	0.775	1.022	0.944	0.971	0.827
Q96L92	Sorting nexin-27 OS=Homo sapiens GN=SNX27 PE=1 SV=2 - [SN	1.254	0.879	0.980	0.865	1.286	1.076	1.123	1.045
Q9Y376	Calcium-binding protein 39 OS=Homo sapiens GN=CAB39 PE=1 S	1.526	1.616	1.771	1.646	1.388	1.187	1.536	1.731
Q8NHQ9	ATP-dependent RNA helicase DDX55 OS=Homo sapiens GN=DDX	1.336	1.468	1.199	0.919	1.348	1.346	1.257	1.026
B4DJ73	cDNA FLJ59546 OS=Homo sapiens PE=2 SV=1 - [B4DJ73_HUMA	1.166	1.348	0.726	0.600	1.289	1.319	1.163	0.743
Q96GM8	Target of EGR1 protein 1 OS=Homo sapiens GN=TOE1 PE=1 SV=	1.408	1.432	0.958	0.931	1.115	1.275	1.289	0.981
Q9NVH2	Integrator complex subunit 7 OS=Homo sapiens GN=INTS7 PE=1	1.914	1.936	1.821	1.614	1.719	1.508	2.027	1.738
Q5M775	Cytospin-B OS=Homo sapiens GN=SPECC1 PE=1 SV=1 - [CYTSB	1.217	1.257	0.835	0.575	1.160	1.188	0.997	0.776
Q9BRQ8	Apoptosis-inducing factor 2 OS=Homo sapiens GN=AIFM2 PE=1 S	2.083	1.758	1.656	1.051	1.775	1.324	1.961	1.656
Q6NX51	Exocyst complex component 4 OS=Homo sapiens GN=EXOC4 PE	1.562	1.059	1.495	1.203	1.328	1.103	1.273	1.106
Q5VT52	Regulation of nuclear pre-mRNA domain-containing protein 2 OS=	1.432	1.462	1.141	1.109	1.302	1.346	1.454	1.240
Q68D91	Metallo-beta-lactamase domain-containing protein 2 OS=Homo sa	1.433	1.125	0.995	0.919	1.179	1.102	1.165	1.054
H0Y6Y8	39S ribosomal protein L43, mitochondrial (Fragment) OS=Homo s	1.065	1.270	1.107	0.866	0.926	1.034	1.066	1.020
Q9H9T3	Elongator complex protein 3 OS=Homo sapiens GN=ELP3 PE=1 S	1.299	1.074	1.143	0.789	1.154	1.069	1.256	0.953
Q96CP6	GRAM domain-containing protein 1A OS=Homo sapiens GN=GRA	1.409	1.243	0.981	0.827	1.049	1.223	1.153	0.799
Q15633	RISC-loading complex subunit TARBP2 OS=Homo sapiens GN=TA	1.435	0.865	0.984	0.839	1.232	1.160	1.156	1.086
P40261	Nicotinamide N-methyltransferase OS=Homo sapiens GN=NNMT	1.261	0.684	1.242	1.025	0.982	0.854	0.872	0.896

Q9H089	Large subunit GTPase 1 homolog OS=Homo sapiens GN=LSG1 PE	1.278	1.592	1.362	0.958	1.251	1.337	1.479	1.160
H0YBU4	Protein-L-isoaspartate O-methyltransferase domain-containing pro	2.473	1.484	2.140	1.820	2.036	0.843	0.871	1.381
A8K321	cDNA FLJ78524, highly similar to Homo sapiens SMILE protein (S	1.672	1.792	1.487	1.422	1.165	1.244	1.642	1.254
Q9BXK5	Bcl-2-like protein 13 OS=Homo sapiens GN=BCL2L13 PE=1 SV=1	1.277	1.264	0.992	0.922	1.140	1.232	1.259	1.027
I1VZV6	Hemoglobin alpha 1 OS=Homo sapiens GN=HBA1 PE=3 SV=1 - [	2.187	1.225	1.332	3.584	2.112	2.215	1.156	1.947
Q86VS8	Protein Hook homolog 3 OS=Homo sapiens GN=HOOK3 PE=1 SV	1.776	1.048	1.294	1.205	1.414	1.130	1.354	1.320
Q92615	La-related protein 4B OS=Homo sapiens GN=LARP4B PE=1 SV=3	1.056	1.053	0.747	0.562	0.973	1.113	0.896	0.634
Q9P013	Spliceosome-associated protein CWC15 homolog OS=Homo sapie	1.411	1.577	1.250	1.078	1.797	1.466	1.427	1.249
Q15526	Surfeit locus protein 1 OS=Homo sapiens GN=SURF1 PE=1 SV=1	0.995	0.961	0.826	0.696	0.811	0.958	0.992	0.904
V9GYH4	ADP-ribosylation factor-related protein 1 OS=Homo sapiens GN=/	1.647	1.757	1.944	1.564	1.540	1.316	2.025	1.492
A8K359	cDNA FLJ76102, highly similar to Homo sapiens excision repair cr	1.461	1.877	1.663	1.491	1.363	1.341	1.472	1.290
Q9Y4C8	Probable RNA-binding protein 19 OS=Homo sapiens GN=RBM19	1.654	1.901	1.573	1.554	1.392	1.436	1.563	1.462
A0A024QZ63	HCG27198, isoform CRA_c OS=Homo sapiens GN=hCG_27198 PE	1.375	1.403	1.168	0.878	1.216	1.215	1.222	1.073
B4DZF6	cDNA FLJ54206, highly similar to Ubiquilin-4 OS=Homo sapiens P	1.172	1.186	0.981	0.965	1.199	1.180	1.083	0.918
A0AV96	RNA-binding protein 47 OS=Homo sapiens GN=RBM47 PE=1 SV=	1.331	1.184	0.752	0.499	1.184	0.933	0.855	0.551
Q9ULU4	Protein kinase C-binding protein 1 OS=Homo sapiens GN=ZMYND	1.896	1.869	1.225	1.189	1.841	1.195	1.685	1.693
F8WA11	CLIP-associating protein 1 OS=Homo sapiens GN=CLASP1 PE=1	1.527	1.226	1.547	1.207	1.036	1.118	1.167	1.126
Q8N884	Cyclic GMP-AMP synthase OS=Homo sapiens GN=MB21D1 PE=1	1.512	2.139	1.763	1.304	1.418	1.210	1.505	1.804
Q9Y4E8	Ubiquitin carboxyl-terminal hydrolase 15 OS=Homo sapiens GN=U	1.493	1.127	1.372	1.048	1.252	1.048	1.312	1.214
A8K5H7	cDNA FLJ77542, highly similar to Homo sapiens YME1-like 1 (S. c	1.298	1.405	1.070	1.119	0.941	1.083	1.264	1.128
E2GIM7	Interferon regulatory factor 3c OS=Homo sapiens GN=IRF3 PE=2	1.205	1.122	2.200	1.454	1.422	0.994	1.316	1.338
Q7Z6U0	TATA box-binding protein-like protein 1 (Fragment) OS=Homo sa	1.646	1.119	1.170	0.727	1.521	1.099	0.858	
Q16595	Frataxin, mitochondrial OS=Homo sapiens GN=FXN PE=1 SV=2 -	3.066	3.776	3.534	3.491	1.931	1.975	3.408	4.034
B4DTU7	10-formyltetrahydrofolate dehydrogenase OS=Homo sapiens PE=	1.198	1.091	0.798	1.019	0.909	1.087	0.814	1.155
O95630	STAM-binding protein OS=Homo sapiens GN=STAMBP PE=1 SV=	1.227	0.949	1.326	1.063	1.271	1.019	1.243	1.139
Q14554	Protein disulfide-isomerase A5 OS=Homo sapiens GN=PDIA5 PE=	1.385	1.177	1.244	1.194	1.281	1.095	1.166	1.220
Q9H5V9	UPF0428 protein CXorf56 OS=Homo sapiens GN=CXorf56 PE=1 S	1.404	1.586	1.554	1.105	1.393	1.640	1.475	1.128
Q9HB40	Retinoid-inducible serine carboxypeptidase OS=Homo sapiens GN	1.523	1.484	1.739	1.985	1.326	1.295	1.670	1.751
Q9Y2R4	Probable ATP-dependent RNA helicase DDX52 OS=Homo sapiens	1.545	1.641	1.341	0.922	1.534	1.353	1.393	1.304
B4DU97	cDNA FLJ57596, highly similar to Peroxisomal membrane protein	5.588	5.007	4.270	4.097	3.190	1.528	4.879	4.339
B3KXN9	cDNA FLJ45793 fis, clone NT2RI2009233 OS=Homo sapiens PE=	1.212	1.048	1.309	1.149	1.113	1.129	1.230	1.127
A8K5Y7	cDNA FLJ78655, highly similar to Homo sapiens exportin 5 (XPO5	2.023	1.632	2.152	1.542	1.941	1.308	1.788	1.402
Q9Y6H1	Coiled-coil-helix-coiled-coil-helix domain-containing protein 2, mit					0.487	1.241	0.487	
D9ZGF5	Fibroblast growth factor OS=Homo sapiens GN=FGF2 PE=3 SV=1	0.945	1.186	0.847	0.760	0.977	1.098	0.926	0.846
M0QY97	Zinc finger CCCH domain-containing protein 4 (Fragment) OS=Ho	1.135	1.156	0.809	0.577	1.135	1.182	0.965	0.708
H0YJ34	Fermitin family homolog 2 (Fragment) OS=Homo sapiens GN=FE	1.355	0.989	1.213	0.929	1.101	0.954	1.097	1.065
A8K1J3	cDNA FLJ78534, highly similar to Homo sapiens RAD23 homolog	2.310	1.852	1.966	1.940	1.565	1.202	1.682	1.889
Q08209	Serine/threonine-protein phosphatase 2B catalytic subunit alpha i	1.343	0.998	1.194	0.925	1.257	1.039	1.020	0.881
A5YKK6	CCR4-NOT transcription complex subunit 1 OS=Homo sapiens GN	1.665	1.702	1.484	1.196	1.384	1.340	1.428	1.145
Q9Y487	V-type proton ATPase 116 kDa subunit a isoform 2 OS=Homo sap	1.450	1.431	1.288	1.205	1.380	1.493	1.654	1.494

K7EP90	RNA-binding protein 42 OS=Homo sapiens GN=RBM42 PE=1 SV=	1.605	1.857	1.428	0.974	1.571	1.479	1.866	1.607
O75391	Sperm-associated antigen 7 OS=Homo sapiens GN=SPAG7 PE=1	1.027	0.706	0.821	0.629	1.070	1.150	0.911	0.696
Q92947	Glutaryl-CoA dehydrogenase, mitochondrial OS=Homo sapiens GI	1.087	1.394	1.166	1.069	0.894	1.018	1.212	1.261
Q14157	Ubiquitin-associated protein 2-like OS=Homo sapiens GN=UBAP2	1.298	1.423	1.192	0.946	1.108	1.237	1.273	1.104
Q9H1E3	Nuclear ubiquitous casein and cyclin-dependent kinase substrate	1.454	0.510	0.550	0.378	1.346	0.654	0.757	0.586
E5RGS2	Receptor expression-enhancing protein 4 OS=Homo sapiens GN=	1.415	1.396	1.163	1.013	1.068	1.263	1.185	1.153
A6XND5	Ribonuclease T2 OS=Homo sapiens PE=2 SV=1 - [A6XND5_HUM	1.016	0.874	1.044	1.355	1.254	1.131	1.431	1.551
Q6NUM9	All-trans-retinol 13,14-reductase OS=Homo sapiens GN=RETSAT	1.461	1.445	1.136	1.017	1.322	1.248	1.313	1.203
Q14746	Conserved oligomeric Golgi complex subunit 2 OS=Homo sapiens	1.739	1.769	1.508	1.046	1.518	1.345	1.814	1.441
B6CGU5	Caspase 8 splice variant OS=Homo sapiens GN=CASP8 PE=2 SV=	1.972	1.338	1.653	1.202	1.563	1.129	1.458	1.185
B3KT11	cDNA FLJ37430 fis, clone BRAWH2001679, highly similar to Hom	2.291	2.155	2.024	1.686	2.417	1.340	2.618	1.910
P48723	Heat shock 70 kDa protein 13 OS=Homo sapiens GN=HSPA13 PE	1.427	1.321	1.044	1.078	1.362	1.528	1.519	1.213
P07199	Major centromere autoantigen B OS=Homo sapiens GN=CENPB P	1.119	1.373	1.247	1.120	1.297	1.293	1.355	1.514
B2R4A5	cDNA, FLJ92019, highly similar to Homo sapiens mitochondrial rib	1.358	1.388	1.072	1.071	1.077	1.201	1.272	1.305
Q8TCD1	UPF0729 protein C18orf32 OS=Homo sapiens GN=C18orf32 PE=	3.414	2.604	2.444	2.102	2.408	1.842	2.413	2.235
O75446	Histone deacetylase complex subunit SAP30 OS=Homo sapiens G	1.099	1.215	0.740	0.428	1.025	0.955	1.017	0.571
Q9BYC8	39S ribosomal protein L32, mitochondrial OS=Homo sapiens GN=	0.697	0.770	0.602	0.505	0.781	1.134	0.826	0.640
Q00535	Cyclin-dependent-like kinase 5 OS=Homo sapiens GN=CDK5 PE=	1.748	1.208	2.819	3.069	1.768	1.218	2.206	3.438
B3KN15	cDNA FLJ13198 fis, clone NT2RP3004454, highly similar to Hepar	1.215	1.524	1.170	1.337	1.121	1.307	1.484	1.204
O95864	Fatty acid desaturase 2 OS=Homo sapiens GN=FADS2 PE=1 SV=	1.267	1.214	0.805	0.550	1.126	1.067	1.118	0.683
Q96IZ0	PRKC apoptosis WT1 regulator protein OS=Homo sapiens GN=PA	1.535	1.190	1.198	0.844	1.675	1.161	1.111	0.959
P49903	Selenide, water dikinase 1 OS=Homo sapiens GN=SEPHS1 PE=1	1.778	1.396	1.565	0.932	1.536	1.269	1.619	1.017
A8K930	cDNA FLJ75500, highly similar to Homo sapiens EH domain bindir	1.200	0.777	0.927	0.616	1.054	0.925	1.072	0.904
J3KT68	Transmembrane protein 97 OS=Homo sapiens GN=TMEM97 PE=	3.281	3.802	3.529	3.129	2.724	1.482	3.520	3.182
P51970	NADH dehydrogenase [ubiquinone] 1 alpha subcomplex subunit 8	1.618	1.604	1.323	1.663	1.406	1.174	1.666	1.972
Q9UHR5	SAP30-binding protein OS=Homo sapiens GN=SAP30BP PE=1 SV	1.712	1.730	1.300	1.071	1.452	1.394	1.676	1.202
Q9ULW3	Activator of basal transcription 1 OS=Homo sapiens GN=ABT1 PE	1.616	1.710	1.146	1.030	1.531	1.494	1.527	1.110
Q8TBQ9	Protein kish-A OS=Homo sapiens GN=TMEM167A PE=1 SV=1 - [	3.787	4.759	4.192	3.871	2.972	1.691	3.751	3.560
Q5HYI8	Rab-like protein 3 OS=Homo sapiens GN=RABL3 PE=1 SV=1 - [R	1.253	1.339	1.194	1.059	1.257	1.193	1.354	0.964
Q9H553	Alpha-1,3/1,6-mannosyltransferase ALG2 OS=Homo sapiens GN=	1.586	1.904	1.580	1.583	1.384	1.362	1.565	1.596
Q9BVV7	Mitochondrial import inner membrane translocase subunit Tim21	1.420	1.416	1.261	1.333	1.059	1.096	1.456	1.422
Q9BQ48	39S ribosomal protein L34, mitochondrial OS=Homo sapiens GN=	1.427	1.956	1.418	1.234	1.115	1.079	1.399	1.513
Q13586	Stromal interaction molecule 1 OS=Homo sapiens GN=STIM1 PE=	1.470	1.460	1.271	1.213	1.168	1.223	1.308	1.102
Q8WWH5	Probable tRNA pseudouridine synthase 1 OS=Homo sapiens GN=	1.657	1.668	1.478	0.537	1.424	1.325	1.321	0.883
F5H0F9	Anaphase-promoting complex subunit 5 OS=Homo sapiens GN=A	1.588	1.334	1.130	0.918	1.492	1.218	1.262	1.181
B1ALH6	Phytanoyl-CoA dioxygenase, peroxisomal OS=Homo sapiens GN=	1.427	1.247	0.986	0.752	1.178	1.092	1.059	0.945
P07992	DNA excision repair protein ERCC-1 OS=Homo sapiens GN=ERCC	1.827	1.713	1.831	1.279	2.089	1.544	1.752	1.616
A0A024RDY9	Rho guanine nucleotide exchange factor (GEF) 7, isoform CRA_b	1.308	0.954	1.363	0.991	1.470	1.085	1.463	1.277
Q13630	GDP-L-fucose synthase OS=Homo sapiens GN=TSTA3 PE=1 SV=	1.129	0.999	1.259	1.123	1.262	0.847	1.240	1.153
I0CE67	Four-and-a-half LIM domains 2 OS=Homo sapiens GN=FHL2 PE=	2.819	2.309	2.306	1.715	2.214	1.368	1.744	1.748

Q9NWB6	Arginine and glutamate-rich protein 1 OS=Homo sapiens GN=AR(	1.536	1.545	1.455	1.259	1.217	1.182	1.343	1.350
D6RHI7	Uncharacterized protein OS=Homo sapiens GN=CCNH PE=1 SV=	2.073	2.370	2.148	1.856	1.724	1.301	2.175	2.280
Q9NRG1	Phosphoribosyltransferase domain-containing protein 1 OS=Homo	1.360	0.975	1.313	1.154	1.294	0.873	1.139	1.271
A0A024RDH6	SEC31-like 1 (S. cerevisiae), isoform CRA_b OS=Homo sapiens GI	1.374	0.949	1.255	0.923	1.190	1.027	1.136	0.958
Q13257	Mitotic spindle assembly checkpoint protein MAD2A OS=Homo sa	1.825	1.376	1.742	1.568	1.471	1.148	1.487	1.400
E7EWP0	NADH dehydrogenase [ubiquinone] 1 beta subcomplex subunit 5,	1.818	1.900	1.706	1.814	1.431	1.261	1.667	1.669
P61923	Coatomer subunit zeta-1 OS=Homo sapiens GN=COPZ1 PE=1 SV	1.689	1.108	1.599	1.276	1.539	1.076	1.399	1.188
F5H345	Porphobilinogen deaminase OS=Homo sapiens GN=HMBS PE=1 S	1.341	1.110	1.391	1.104	1.343	1.020	1.196	1.126
Q9BTY2	Plasma alpha-L-fucosidase OS=Homo sapiens GN=FUCA2 PE=1 S	1.435	1.161	1.406	1.510	1.432	1.247	1.667	2.083
B2R704	cDNA, FLJ93207, highly similar to Homo sapiens microtubule-asso	1.387	1.085	1.240	0.867	1.156	1.045	1.132	0.948
C9JG97	Angio-associated migratory cell protein OS=Homo sapiens GN=A/	1.310	1.022	1.709	1.351	1.147	1.240	1.561	1.282
Q14444	Caprin-1 OS=Homo sapiens GN=CAPRIN1 PE=1 SV=2 - [CAPR1_	1.727	1.551	1.319	0.951	1.327	1.272	1.514	1.135
B2RB95	cDNA, FLJ95381, Homo sapiens tripartite motif-containing 16 (TR	1.589	1.044	1.266	0.880	1.434	0.994	1.107	0.957
Q53EY9	F-box only protein 22 isoform a variant (Fragment) OS=Homo sap	1.485	1.171	1.578	1.147	1.311	1.209	1.372	1.574
P47895	Aldehyde dehydrogenase family 1 member A3 OS=Homo sapiens	1.356	0.788	1.046	0.923	1.309	1.038	1.195	0.897
Q9UL03	Integrator complex subunit 6 OS=Homo sapiens GN=INTS6 PE=1	1.577	1.581	1.166	0.961	1.339	1.248	1.448	1.065
Q7L5D6	Golgi to ER traffic protein 4 homolog OS=Homo sapiens GN=GET	1.215	1.103	1.173	0.886	1.138	1.526	1.333	1.014
Q8N5Y3	GYG1 protein (Fragment) OS=Homo sapiens GN=GYG1 PE=2 SV=	1.616	1.161	2.270	1.211	1.349	1.217	1.931	1.874
Q3B874	STRN protein (Fragment) OS=Homo sapiens GN=STRN PE=2 SV=	1.718	1.644	1.490	1.348	1.651	1.244	1.528	1.267
B4DWZ7	cDNA FLJ56802, highly similar to LanC-like protein 2 OS=Homo s	2.052	1.829	2.163	1.864	1.557	1.503	1.945	2.155
Q06203	Amidophosphoribosyltransferase OS=Homo sapiens GN=PPAT PE	1.081	0.905	1.231	1.088	1.075	0.948	1.162	1.053
Q9H5Q4	Dimethyladenosine transferase 2, mitochondrial OS=Homo sapier	1.797	2.045	1.598	1.494	1.275	1.440	1.631	1.345
A0A024R725	Inosine-5'-monophosphate dehydrogenase OS=Homo sapiens GN	2.028	1.510	2.377	1.796	1.310	1.114	1.923	2.006
Q49A71	COQ7 protein OS=Homo sapiens GN=COQ7 PE=2 SV=1 - [Q49A7	2.155	2.430	2.051	2.008	2.175	2.014	2.200	2.355
Q96H55	Unconventional myosin-XIX OS=Homo sapiens GN=MYO19 PE=2	1.361	1.353	1.349	1.207	1.330	1.345	1.464	1.326
A0A087WTW0	E3 ubiquitin-protein ligase UHRF1 OS=Homo sapiens GN=UHRF1	1.420	2.846	2.262	1.582	1.242	2.010	1.943	1.296
Q8IUF8	Bifunctional lysine-specific demethylase and histidyl-hydroxylase I	1.240	1.597	1.330	1.185	1.214	1.427	1.392	1.362
Q9P2N5	RNA-binding protein 27 OS=Homo sapiens GN=RBM27 PE=1 SV=	1.385	1.591	1.046	0.911	1.249	1.305	1.253	1.037
Q96HR3	Mediator of RNA polymerase II transcription subunit 30 OS=Homo	1.801	1.986	1.221	1.238	1.413	1.178	1.615	1.413
B3KM35	cDNA FLJ10144 fis, clone HEMBA1003286, highly similar to Beta-	1.470	1.388	1.093	0.905	1.226	1.134	1.090	0.910
Q15031	Probable leucine--tRNA ligase, mitochondrial OS=Homo sapiens G	1.456	1.596	1.358	1.369	1.047	1.341	1.486	1.544
Q5HYL4	Putative uncharacterized protein DKFZp686E1893 OS=Homo sapi	1.342	1.594	1.307	1.197	1.365	1.343	1.403	1.163
I3L4A2	Leucine carboxyl methyltransferase 1 (Fragment) OS=Homo sapie	2.263	2.067	2.944	2.477	1.499	1.563	2.063	2.105
A0A087WZY4	Metaxin 1, isoform CRA_b OS=Homo sapiens GN=MTX1 PE=4 SV	1.395	1.612	1.367	1.378	1.066	1.387	1.326	1.406
Q8NBI6	Xyloside xylosyltransferase 1 OS=Homo sapiens GN=XXYLT1 PE=	1.526	1.667	1.434	1.668	1.083	1.485	1.811	1.626
E7EV99	Alpha-adducin OS=Homo sapiens GN=ADD1 PE=1 SV=1 - [E7EV	1.152	1.103	1.143	1.018	1.160	1.021	1.126	1.068
A0A024R7G6	Epidermal growth factor receptor pathway substrate 15-like 1, isc	2.042	1.751	1.914	1.810	1.928	1.194	1.778	1.731
Q8IWX8	Calcium homeostasis endoplasmic reticulum protein OS=Homo sa	1.548	1.727	1.354	1.317	1.361	1.374	1.562	1.751
Q71F23	Centromere protein U OS=Homo sapiens GN=CENPU PE=1 SV=1	1.355	1.570	1.424	1.174	1.164	1.339	1.790	1.522
Q9UH17	DNA dC->dU-editing enzyme APOBEC-3B OS=Homo sapiens GN=	2.894	2.371	1.236	0.928	2.564	1.317	2.123	1.832

P42830	C-X-C motif chemokine 5 OS=Homo sapiens GN=CXCL5 PE=1 SV	1.484	0.385			1.082	0.273		
Q96AT9	Ribulose-phosphate 3-epimerase OS=Homo sapiens GN=RPE PE=	0.895	0.734	1.193	1.034	0.965	0.929	0.957	0.994
C9J8T4	E3 ubiquitin-protein ligase RNF13 (Fragment) OS=Homo sapiens	1.785	1.566	1.000	0.825	1.676	1.196	1.183	0.924
B7Z7A4	cDNA FLJ55148, highly similar to Thioredoxin domain-containing	1.342	1.011	1.183	0.861	1.247	1.130	1.223	1.004
O75818	Ribonuclease P protein subunit p40 OS=Homo sapiens GN=RPP4	1.636	1.844	1.349	1.044	1.782	1.380	1.503	1.400
Q96S66	Chloride channel CLIC-like protein 1 OS=Homo sapiens GN=CLCC	1.116	1.095	1.145	0.988	0.989	1.345	1.207	1.103
B2R8K8	cDNA, FLJ93949, highly similar to Homo sapiens NIMA (never in	1.122	0.683	0.905	0.956	1.001	0.989	0.834	0.772
H3BQM0	Non-canonical poly(A) RNA polymerase PAPD5 OS=Homo sapiens	1.482	1.660	1.476	1.185	1.374	1.371	1.690	1.422
F8VV52	CCR4-NOT transcription complex subunit 2 (Fragment) OS=Homo	1.663	2.100	1.291	1.068	1.404	1.346	1.483	1.272
Q9NPF4	Probable tRNA N6-adenosine threonylcarbamoyltransferase OS=H	1.354	0.918	1.055	1.012	1.181	0.966	0.942	1.014
P35219	Carbonic anhydrase-related protein OS=Homo sapiens GN=CA8 P	1.118		1.437		1.457	1.122	1.061	
Q7L8L6	FAST kinase domain-containing protein 5 OS=Homo sapiens GN=	1.235	1.453	1.082	1.001	1.089	1.239	1.259	1.096
Q53Q75	Putative uncharacterized protein C2orf5 (Fragment) OS=Homo sa	1.031	0.766	1.174	1.066	1.043	1.077	0.981	1.361
Q8TD30	Alanine aminotransferase 2 OS=Homo sapiens GN=GPT2 PE=1 S	1.403	1.703	1.427	1.091	1.089	1.473	1.639	1.435
Q8IXB1	DnaJ homolog subfamily C member 10 OS=Homo sapiens GN=D	2.014	1.620	1.971	1.584	1.882	1.375	1.858	1.578
Q9Y6M5	Zinc transporter 1 OS=Homo sapiens GN=SLC30A1 PE=1 SV=3 -	2.864	3.451	2.097	2.326	2.175	1.694	1.884	1.530
Q9NVU7	Protein SDA1 homolog OS=Homo sapiens GN=SDAD1 PE=1 SV=	1.491	1.735	1.493	1.232	1.595	1.513	1.583	1.343
Q96NT0	Coiled-coil domain-containing protein 115 OS=Homo sapiens GN=	1.283	1.463	1.267	0.697	1.345	1.341	1.275	1.257
Q6PID6	Tetratricopeptide repeat protein 33 OS=Homo sapiens GN=TTC33	3.385	1.209	1.200	2.070	1.362	1.481	0.941	2.148
O60870	DNA/RNA-binding protein KIN17 OS=Homo sapiens GN=KIN PE=	1.073	1.292	1.082	0.728	1.013	1.337	1.037	0.826
Q9HAV0	Guanine nucleotide-binding protein subunit beta-4 OS=Homo sap	1.304	2.192	2.268	2.413	1.403	1.156	3.348	3.296
I3L4C2	Coiled-coil domain-containing protein 43 OS=Homo sapiens GN=	1.493	1.783	1.991	1.606	1.358	1.250	1.777	1.475
E9PCX8	Tensin-3 (Fragment) OS=Homo sapiens GN=TNS3 PE=1 SV=3 -	1.794	0.970	0.692	0.414	1.316	0.729	0.648	0.431
Q13415	Origin recognition complex subunit 1 OS=Homo sapiens GN=ORC	1.370	2.640	1.983	1.821	1.317	1.880	2.260	2.164
H0Y5D5	Cip1-interacting zinc finger protein (Fragment) OS=Homo sapiens	1.059	1.575	1.440	1.041	1.334	1.626	1.874	1.777
Q86SR1	Polypeptide N-acetylgalactosaminyltransferase 10 OS=Homo sapi	1.326	1.466	1.009	0.892	1.322	1.274	1.259	0.960
B2RD27	cDNA, FLJ96428, highly similar to Homo sapiens proteasome (pro	1.066	0.829	0.856	0.547	1.040	0.935	0.778	0.646
P54709	Sodium/potassium-transporting ATPase subunit beta-3 OS=Homo	1.759	2.732	2.303	2.063	1.493	1.701	2.599	2.456
Q9BRR6	ADP-dependent glucokinase OS=Homo sapiens GN=ADPGK PE=1	1.370	1.470	1.221	1.319	1.070	1.241	1.272	1.345
Q01968	Inositol polyphosphate 5-phosphatase OCRL-1 OS=Homo sapiens	1.446	1.252	1.751	1.292	1.453	1.033	1.391	1.371
Q6IAN0	Dehydrogenase/reductase SDR family member 7B OS=Homo sap	1.608	1.594	1.284	1.328	1.255	1.219	1.684	1.296
P24928	DNA-directed RNA polymerase II subunit RPB1 OS=Homo sapiens	1.181	1.438	1.020	0.851	1.132	1.261	1.270	0.837
K7ESK0	Ubiquitin carboxyl-terminal hydrolase 22 (Fragment) OS=Homo s	1.813	1.679	1.248	0.730	1.831	1.388	1.733	1.191
O60231	Putative pre-mRNA-splicing factor ATP-dependent RNA helicase D	1.193	1.366	0.995	0.682	1.196	1.120	0.979	0.821
P55854	Small ubiquitin-related modifier 3 OS=Homo sapiens GN=SUMO3	0.500	0.462	0.379	0.370	1.101	1.137	0.703	0.599
P51948	CDK-activating kinase assembly factor MAT1 OS=Homo sapiens G	1.253	1.503	1.225	1.166	1.232	1.413	1.407	1.643
Q08380	Galectin-3-binding protein OS=Homo sapiens GN=LGALS3BP PE=	1.336	1.209	0.931	1.019	1.204	1.109	1.176	1.070
H7BXF4	Sphingomyelin phosphodiesterase 4 OS=Homo sapiens GN=SMPI	1.584	1.885	1.896	1.735	1.322	1.435	1.894	1.640
P23229	Integrin alpha-6 OS=Homo sapiens GN=ITGA6 PE=1 SV=5 - [IT	1.384	1.519	1.215	1.299	1.289	1.223	1.370	1.150
Q5GJ64	Hypothetical rhabdomyosarcoma antigen MU-RMS-40.5 (Fragmer	1.396	1.108	1.086	0.815	1.244	1.092	1.169	0.888

B7ZMD6	IRGQ protein OS=Homo sapiens GN=IRGQ PE=2 SV=1 - [B7ZMD	1.520	0.946	1.189	1.103	1.213	1.026	1.345	1.283
O60306	Intron-binding protein aquarius OS=Homo sapiens GN=AQR PE=	1.466	1.542	1.291	1.005	1.417	1.511	1.452	1.173
Q12972	Nuclear inhibitor of protein phosphatase 1 OS=Homo sapiens GN	1.517	1.639	1.215	0.795	1.507	1.282	1.224	1.008
Q9UK54	Hemoglobin beta subunit variant (Fragment) OS=Homo sapiens (	0.203	0.227	0.455	0.544	0.395	0.401	0.335	0.370
P25815	Protein S100-P OS=Homo sapiens GN=S100P PE=1 SV=2 - [S10(	1.621	1.158	2.509	2.034	1.425	1.292	1.835	1.754
Q9UBK9	Protein UXT OS=Homo sapiens GN=UXT PE=1 SV=1 - [UXT_HUN	1.582	1.301	1.868	1.322	1.686	1.091	1.504	1.417
A0A024R326	Ribosomal protein L29, isoform CRA_a OS=Homo sapiens GN=RF	1.259	1.176	1.343	0.966	1.151	1.203	1.197	1.203
Q53X12	V-type proton ATPase subunit a OS=Homo sapiens PE=1 SV=1 -	1.558	1.570	1.472	1.500	1.322	1.361	1.542	1.477
Q9NVD7	Alpha-parvin OS=Homo sapiens GN=PARVA PE=1 SV=1 - [PARVA	1.732	1.319	1.808	1.506	1.756	1.044	1.353	1.376
Q9Y3Y2	Chromatin target of PRMT1 protein OS=Homo sapiens GN=CHTO	1.308	1.625	1.278	0.859	1.264	1.288	1.417	1.382
A8K7A0	cDNA FLJ75753, highly similar to Homo sapiens primase, polypep	0.961	0.761	0.817	0.619	1.067	1.125	1.020	0.797
Q96QE5	Transcription elongation factor, mitochondrial OS=Homo sapiens (	1.594	1.662	1.264	1.145	1.239	1.171	1.354	1.290
B4DJ38	cDNA FLJ56092, highly similar to Pentatricopeptide repeat protei	1.350	1.531	1.143	1.051	1.062	1.206	1.271	1.104
Q12824	SWI/SNF-related matrix-associated actin-dependent regulator of (	2.040	2.292	1.441	0.973	1.930	1.507	1.822	1.194
P61599	N-alpha-acetyltransferase 20 OS=Homo sapiens GN=NAA20 PE=1	1.619	1.107	1.758	1.249	1.486	1.448	1.552	1.338
B4DI01	cDNA FLJ53994, highly similar to CDK5 regulatory subunit-associa	1.255	1.512	1.296	1.055	1.001	1.266	1.347	0.965
Q13228	Selenium-binding protein 1 OS=Homo sapiens GN=SELENBP1 PE	1.254	0.984	1.625	1.294	0.994	1.024	1.392	1.260
Q8WX92	Negative elongation factor B OS=Homo sapiens GN=NELFB PE=1	1.322	1.321	1.179	0.791	1.364	1.233	1.136	0.879
A8K6M4	cDNA FLJ75725, highly similar to Homo sapiens vesicle transport	1.395	1.327	1.013	0.920	1.384	1.296	1.477	1.393
Q7Z6Z7	E3 ubiquitin-protein ligase HUWE1 OS=Homo sapiens GN=HUWE	1.322	1.047	1.133	0.912	1.253	1.035	1.102	0.973
A6NIR2	Chromosome 1 open reading frame 41, isoform CRA_b OS=Homo	0.674	0.446	0.626	0.570	0.994	1.100	1.018	0.667
Q9NX02	NACHT, LRR and PYD domains-containing protein 2 OS=Homo sa	1.774	1.301	1.854	1.466	1.379	1.131	1.944	1.722
A0A024R4A5	Trinucleotide repeat containing 15, isoform CRA_a OS=Homo sap	1.147	1.505	1.216	0.953	1.135	1.180	1.226	1.032
Q96SB8	Structural maintenance of chromosomes protein 6 OS=Homo sap	1.305	1.380	1.073	1.123	1.432	1.215	1.322	1.285
Q8TEM1	Nuclear pore membrane glycoprotein 210 OS=Homo sapiens GN=	1.252	1.402	1.210	1.235	0.936	1.140	1.431	1.299
Q8NBU5	ATPase family AAA domain-containing protein 1 OS=Homo sapien	1.599	2.160	2.174	2.200	1.813	1.449	2.358	1.686
Q8IXT5	RNA-binding protein 12B OS=Homo sapiens GN=RBM12B PE=1 S	1.412	1.554	1.384	1.242	1.461	1.458	1.627	1.425
P11182	Lipoamide acyltransferase component of branched-chain alpha-ke	1.195	1.381	1.215	1.060	0.910	1.212	1.173	1.093
Q8TB61	Adenosine 3'-phospho 5'-phosphosulfate transporter 1 OS=Homo	1.652	1.857	1.437	1.312	1.465	1.288	1.612	1.559
Q9H8V3	Protein ECT2 OS=Homo sapiens GN=ECT2 PE=1 SV=4 - [ECT2_H	1.059	0.985	0.714	0.590	1.018	0.899	0.987	0.819
Q14807	Kinesin-like protein KIF22 OS=Homo sapiens GN=KIF22 PE=1 SV	1.378	1.696	1.381	1.157	1.372	1.359	1.625	1.985
Q96GC5	39S ribosomal protein L48, mitochondrial OS=Homo sapiens GN=	1.236	1.497	1.124	1.035	0.932	1.107	1.178	1.180
Q5HYI7	Metaxin-3 OS=Homo sapiens GN=MTX3 PE=1 SV=2 - [MTX3_HU	2.090	2.524	2.202	2.137	1.548	1.542	1.936	1.897
A8K4H1	cDNA FLJ78268, highly similar to Homo sapiens fusion (involved i	3.056	3.084	2.015	2.184	2.788	1.827	2.785	1.725
Q9NXE8	Pre-mRNA-splicing factor CWC25 homolog OS=Homo sapiens GN	1.759	1.996	1.331	1.089	1.209	1.333	1.290	1.463
P49069	Calcium signal-modulating cyclophilin ligand OS=Homo sapiens G	2.041	2.385	1.841	1.463	1.526	1.426	1.839	1.496
P39880	Homeobox protein cut-like 1 OS=Homo sapiens GN=CUX1 PE=1	1.015	1.233	0.762	0.568	1.143	1.109	1.019	1.278
P82094	TATA element modulatory factor OS=Homo sapiens GN=TMF1 PE	1.512	1.644	1.476	1.301	1.399	1.491	1.607	1.493
P62699	Protein yippee-like 5 OS=Homo sapiens GN=YPEL5 PE=1 SV=1 -	1.230	1.124	0.883	0.562	1.153	1.090	0.960	0.683
B4E324	cDNA FLJ60397, highly similar to Lysosomal protective protein (E(	1.034	0.938	1.099	0.996	1.102	1.069	1.154	1.114

Q12933	TNF receptor-associated factor 2 OS=Homo sapiens GN=TRAF2 P	3.467	2.114	2.802	2.165	1.773	1.093	1.170	1.466
Q9UL15	BAG family molecular chaperone regulator 5 OS=Homo sapiens G	1.286	1.343	1.385	1.476	1.196	1.380	1.171	1.515
Q9Y5Q9	General transcription factor 3C polypeptide 3 OS=Homo sapiens (	1.503	1.783	1.045	1.003	1.393	1.329	1.510	1.859
A8K7A2	cDNA FLJ78084, highly similar to Homo sapiens cell division cycle	1.051	1.047	0.558	1.045	0.803	1.463	0.987	0.541
O43913	Origin recognition complex subunit 5 OS=Homo sapiens GN=ORC	1.449	1.664	1.406	1.201	1.373	1.348	1.396	1.411
Q9P2X0	Dolichol-phosphate mannosyltransferase subunit 3 OS=Homo sap	1.484	1.447	1.191	1.285	1.029	1.164	1.439	1.227
Q14919	Dr1-associated corepressor OS=Homo sapiens GN=DRAP1 PE=1	1.209	1.051	1.175	1.008	1.152	1.041	1.221	1.062
Q96HN2	Putative adenosylhomocysteinase 3 OS=Homo sapiens GN=AHCY	0.764	1.349	0.961	0.792	0.800	1.018	1.007	0.913
Q7Z6K5	Arpin OS=Homo sapiens GN=ARPIN PE=1 SV=1 - [ARPIN_HUMA	1.069	0.869	1.220	1.003	1.058	1.022	1.115	1.042
Q9BRF8	Serine/threonine-protein phosphatase CPPED1 OS=Homo sapiens	1.583	1.105	1.606	1.476	1.249	0.951	1.236	1.208
Q969G6	Riboflavin kinase OS=Homo sapiens GN=RFK PE=1 SV=2 - [RIFK	1.584	1.088	1.384	1.092	1.154	1.056	1.000	0.952
Q59E88	DnaJ (Hsp40) homolog, subfamily A, member 3 variant (Fragmen	1.802	2.079	1.694	1.616	1.388	1.557	2.424	1.934
B4DQG8	cDNA FLJ54124, highly similar to DNA methyltransferase 1-associ	0.931	1.673	1.263	0.893	1.073	1.452	1.460	0.948
O15091	Mitochondrial ribonuclease P protein 3 OS=Homo sapiens GN=KI/	1.395	1.636	1.011	0.945	0.992	0.959	1.176	1.257
B2RDV2	cDNA, FLJ96778, highly similar to Homo sapiens aminopeptidase-	1.151	1.001	1.126	0.790	1.241	1.075	0.997	0.899
Q9BSC4	Nucleolar protein 10 OS=Homo sapiens GN=NOL10 PE=1 SV=1 -	1.721	1.885	1.613	1.495	1.543	1.327	1.811	1.613
A8K9E1	cDNA FLJ77957, highly similar to Homo sapiens HBS1-like (S. cer	1.438	1.000	1.352	1.150	1.256	1.130	1.128	0.999
P28288	ATP-binding cassette sub-family D member 3 OS=Homo sapiens (	1.151	1.307	1.168	1.193	1.055	1.191	1.334	1.172
Q53G08	DNA replication complex GINS protein PSF2 variant (Fragment) O	1.131	1.066	1.272	0.964	1.215	1.076	1.023	1.082
P52788	Spermine synthase OS=Homo sapiens GN=SMS PE=1 SV=2 - [SI	2.758	1.775	2.522	1.717	1.674	1.137	1.709	1.941
Q9NRY5	Protein FAM114A2 OS=Homo sapiens GN=FAM114A2 PE=1 SV=4	2.103	1.647	2.031	1.420	1.598	1.324	1.801	1.769
J3QS48	Mannose-P-dolichol utilization defect 1 protein OS=Homo sapiens	2.810	3.034	2.640	2.898	2.492	1.654	3.347	2.850
Q13617	Cullin-2 OS=Homo sapiens GN=CUL2 PE=1 SV=2 - [CUL2_HUMA	1.675	1.340	1.243	0.835	1.505	1.293	1.391	1.070
Q8WYP5	Protein ELYS OS=Homo sapiens GN=AHCTF1 PE=1 SV=3 - [ELYS	1.533	1.738	1.225	1.156	1.035	1.297	1.465	1.346
Q5RI15	Cytochrome c oxidase protein 20 homolog OS=Homo sapiens GN	0.973	1.168	1.005	0.896	0.948	1.005	1.251	0.898
O60678	Protein arginine N-methyltransferase 3 OS=Homo sapiens GN=PF	1.595	1.106	1.603	1.260	1.241	1.155	1.446	1.317
Q9H0E2	Toll-interacting protein OS=Homo sapiens GN=TOLLIP PE=1 SV=	1.335	1.245	1.074	0.989	1.319	1.172	1.252	0.966
Q8IY18	Structural maintenance of chromosomes protein 5 OS=Homo sap	1.195	1.686	1.307	0.970	1.046	1.121	1.353	1.219
M0R116	Sodium/potassium-transporting ATPase subunit alpha-3 OS=Hom	1.725	2.592	1.873	2.168	1.710	1.727	1.949	2.197
X5DP57	Receptor accessory protein 3 isoform A OS=Homo sapiens GN=R	1.770	2.060	1.754	1.660	1.424	1.525	1.856	1.591
Q8N523	Tuftelin-interacting protein 11 OS=Homo sapiens GN=TFIP11 PE=	1.383	1.472	1.231	0.965	1.434	1.392	1.439	1.240
Q6P087	RNA pseudouridylate synthase domain-containing protein 3 OS=H	1.767	1.535	1.272	0.904	1.320	1.119	1.374	1.088
Q15120	[Pyruvate dehydrogenase (acetyl-transferring)] kinase isozyme 3,	1.331	1.435	0.889	1.013	0.866	1.300	1.268	1.249
Q7Z7K6	Centromere protein V OS=Homo sapiens GN=CENPV PE=1 SV=1	2.004	2.332	1.771	1.515	1.766	1.378	1.976	1.640
B7Z3I9	Delta-aminolevulinic acid dehydratase OS=Homo sapiens PE=2 S\	1.115		0.731	0.860	0.993	1.087	1.064	0.691
P41214	Eukaryotic translation initiation factor 2D OS=Homo sapiens GN=	1.265	1.394	1.252	1.038	0.957	0.984	1.165	0.945
Q9BW19	Kinesin-like protein KIFC1 OS=Homo sapiens GN=KIFC1 PE=1 SV	1.122	1.063	0.814	0.607	1.172	1.080	1.079	0.954
P16930	Fumarylacetoacetase OS=Homo sapiens GN=FAH PE=1 SV=2 - [	1.460	1.126	1.567	1.168	1.219	1.142	1.248	1.164
P46100	Transcriptional regulator ATRX OS=Homo sapiens GN=ATRX PE=	1.357	1.509	1.207	0.929	1.321	1.300	1.273	1.146
Q8NBN7	Retinol dehydrogenase 13 OS=Homo sapiens GN=RDH13 PE=1 S	1.333	1.418	1.242	1.053	1.205	1.384	1.347	1.257

P49674	Casein kinase I isoform epsilon OS=Homo sapiens GN=CSNK1E P		0.721			0.987	1.463	1.019	
D6RDV0	Tumor suppressor candidate 3 OS=Homo sapiens GN=TUSC3 PE=	1.043	1.222	1.090	0.972	0.905	1.147	1.326	0.850
O95159	Zinc finger protein-like 1 OS=Homo sapiens GN=ZFPL1 PE=1 SV=	2.218	3.132	2.067	2.140	1.481	1.448	1.741	1.798
Q5T063	Hexaprenyldihydroxybenzoate methyltransferase, mitochondrial C	2.463	2.465	2.276	2.231	2.119	1.481	2.927	2.857
B2RAR2	cDNA, FLJ95064, highly similar to Homo sapiens nin one binding	1.706	1.444	1.781	1.125	1.382	1.272	1.515	1.223
Q13438	Protein OS-9 OS=Homo sapiens GN=OS9 PE=1 SV=1 - [OS9_HU	5.692	4.546	4.591	4.195	3.238	1.327	4.905	4.756
Q5JSK8	High mobility group nucleosome-binding domain-containing prote	2.178	2.594	2.377	2.301	2.007	1.427	2.582	2.411
P21399	Cytoplasmic aconitate hydratase OS=Homo sapiens GN=ACO1 PE	1.026	0.776	1.277	1.007	0.983	0.963	1.098	1.200
I3L4X3	NF-kappa-B inhibitor beta (Fragment) OS=Homo sapiens GN=NFI	2.644	2.180	2.666	1.734	2.075	1.418	2.182	2.204
A8MT40	Pyruvate dehydrogenase phosphatase regulatory subunit, mitoch	1.207	1.669	1.430	1.401	1.127	1.353	1.196	1.145
Q12873	Chromodomain-helicase-DNA-binding protein 3 OS=Homo sapien	1.746	1.735	1.322	1.179	1.621	1.153	1.573	1.519
Q5TBB1	Ribonuclease H2 subunit B OS=Homo sapiens GN=RNASEH2B PE	1.077	1.120	1.436	1.105	1.037	1.060	1.027	0.947
Q06055	ATP synthase F(0) complex subunit C2, mitochondrial OS=Homo	1.096	1.298	1.026	1.011	0.821	0.847	1.161	1.094
Q8WUK0	Phosphatidylglycerophosphatase and protein-tyrosine phosphatas	1.381	1.738	1.180	1.215	1.199	0.991	1.708	1.285
Q9H1B7	Interferon regulatory factor 2-binding protein-like OS=Homo sapi	1.226	1.012	0.655	0.419	1.077	1.227	0.883	0.646
Q15018	BRISC complex subunit Abro1 OS=Homo sapiens GN=FAM175B F	1.211	0.884	1.169	0.770	1.077	0.955	1.041	0.906
B8ZZQ6	Uncharacterized protein OS=Homo sapiens GN=PTMA PE=1 SV=	1.393	1.202	1.575	1.320	1.325	1.282	1.311	1.284
P08240	Signal recognition particle receptor subunit alpha OS=Homo sapie	1.312	1.454	1.161	1.089	1.222	1.212	1.364	1.321
P35354	Prostaglandin G/H synthase 2 OS=Homo sapiens GN=PTGS2 PE=	1.082	0.500	0.271	0.358	0.989	0.584	0.371	0.181
Q5SRE5	Nucleoporin NUP188 homolog OS=Homo sapiens GN=NUP188 PE	1.888	1.985	2.026	2.055	1.830	1.663	2.157	2.127
P53582	Methionine aminopeptidase 1 OS=Homo sapiens GN=METAP1 PE	2.799	2.270	2.892	1.843	2.627	1.442	2.829	2.878
Q5VV42	Threonylcarbamoyladenosine tRNA methylthiotransferase OS=Ho	1.106	1.242	1.085	0.959	1.010	1.268	1.443	1.129
O15439	Multidrug resistance-associated protein 4 OS=Homo sapiens GN=	1.349	1.535	1.282	1.322	1.265	1.098	1.164	1.248
B4DJ39	cDNA FLJ54357, highly similar to Epidermal Langerhans cell prote	1.018	0.967	0.733	0.697	0.938	1.291	0.925	0.570
Q53F20	Acidic (Leucine-rich) nuclear phosphoprotein 32 family, member E	2.812	1.684	2.386	2.023	2.441	0.906	1.890	1.434
H0YNG3	Signal peptidase complex catalytic subunit SEC11A OS=Homo sa	1.604	1.540	1.318	1.233	1.326	1.176	1.586	1.318
A0A087WXU0	Required for meiotic nuclear division protein 1 homolog OS=Hom	1.827	2.168	1.657	1.663	1.578	1.119	1.867	1.798
Q8IYV2	DEAD (Asp-Glu-Ala-Asp) box polypeptide 20 OS=Homo sapiens G	1.297	1.524	1.571	1.243	1.153	1.384	1.498	1.299
Q9NR46	Endophilin-B2 OS=Homo sapiens GN=SH3GLB2 PE=1 SV=1 - [SH	1.210	0.807	1.214	0.971	1.066	0.943	1.129	0.915
Q9NZZ3	Charged multivesicular body protein 5 OS=Homo sapiens GN=CH	1.295	0.774	1.059	0.895	1.113	0.873	1.022	0.941
Q9BWH2	FUN14 domain-containing protein 2 OS=Homo sapiens GN=FUND	2.533	2.937	2.331	2.045	1.449	1.538	2.113	2.739
G3V5T0	Maleylacetoacetate isomerase OS=Homo sapiens GN=GSTZ1 PE=	1.549	1.370	1.321	1.242	1.514	1.209	1.070	1.389
Q9UPQ0	LIM and calponin homology domains-containing protein 1 OS=Ho	1.933	1.205	1.571	1.032	1.648	1.187	1.163	1.072
Q8IV08	Phospholipase D3 OS=Homo sapiens GN=PLD3 PE=1 SV=1 - [PL	1.277	1.072	0.803	0.654	1.227	1.035	1.090	0.826
Q6QNY5	UDP-N-acetylglucosamine-2-epimerase / N-acetylmannosamine ki	1.378	0.802	1.073	0.801	1.138	0.854	0.909	0.825
Q96H20	Vacuolar-sorting protein SNF8 OS=Homo sapiens GN=SNF8 PE=1	1.398	1.148	1.524	0.977	1.195	1.213	1.394	1.039
Q9NUQ7	Ufm1-specific protease 2 OS=Homo sapiens GN=UFSP2 PE=1 SV	1.154	1.311	1.283	1.167	1.218	1.456	1.648	1.426
E7EPN9	Protein PRRC2C OS=Homo sapiens GN=PRRC2C PE=1 SV=1 - [E	1.256	1.507	1.109	0.910	1.010	1.226	1.265	0.931
O15164	Transcription intermediary factor 1-alpha OS=Homo sapiens GN=	1.047	0.996	0.691	0.543	1.226	1.040	0.945	0.701
A0A024RAI6	Bridging integrator 1, isoform CRA_g OS=Homo sapiens GN=BIN	1.233	0.850	1.005	0.862	1.162	0.990	0.985	0.992

Q96MW1	Coiled-coil domain-containing protein 43 OS=Homo sapiens GN=(	3.295	2.664	3.583	2.908	2.049	1.218	2.523	2.603
P17252	Protein kinase C alpha type OS=Homo sapiens GN=PRKCA PE=1	1.138	1.333	1.033	1.081	1.022	1.125	1.147	1.081
C9J050	Choline-phosphate cytidylyltransferase A (Fragment) OS=Homo s	1.162	0.975	0.888	0.777	1.263	1.165	0.994	0.814
Q5QPM7	Proteasome inhibitor PI31 subunit OS=Homo sapiens GN=PSMF1	2.824	2.073	3.005	2.302	2.487	1.172	2.736	2.629
E9PEZ3	Protein diaphanous homolog 1 OS=Homo sapiens GN=DIAPH1 PI	1.579	1.005	1.299	0.960	1.312	1.101	1.363	1.142
Q9NRW7	Vacuolar protein sorting-associated protein 45 OS=Homo sapiens	1.241	1.073	1.199	0.813	1.225	1.330	1.126	1.143
H7BZT4	Small ubiquitin-related modifier OS=Homo sapiens GN=SUMO2 P	1.714	1.613	1.585	1.559	1.748	1.551	1.809	2.135
Q96CN7	Isochorismatase domain-containing protein 1 OS=Homo sapiens (	1.269	0.976	1.539	1.529	1.149	1.140	1.208	1.135
A0A087WWP4	Putative RNA-binding protein 15 OS=Homo sapiens GN=RBM15 F	1.143	1.582	1.222	0.877	1.123	1.324	1.448	1.104
A0A024R684	Numb homolog (Drosophila), isoform CRA_e OS=Homo sapiens G	1.207	1.085	1.036	1.049	0.987	0.986	1.029	1.182
Q86W50	Methyltransferase-like protein 16 OS=Homo sapiens GN=METTL1	1.645	1.264	1.418	0.883	1.500	1.101	1.112	1.030
Q9Y2X9	Zinc finger protein 281 OS=Homo sapiens GN=ZNF281 PE=1 SV=	1.210	1.056	0.759	0.667	0.987	1.009	0.957	0.920
Q9H9B1	Histone-lysine N-methyltransferase EHMT1 OS=Homo sapiens GN	1.524	1.767	1.220	1.305	1.501	1.484	1.491	1.541
Q9NRY2	SOSS complex subunit C OS=Homo sapiens GN=INIP PE=1 SV=1	1.705	1.600	1.348	1.167	1.877	1.414	1.678	1.249
B7Z410	cDNA FLJ50791, highly similar to Nitrilase homolog 1 (EC 3.5.-.-)	1.184	0.922	1.032	1.050	1.037	0.934	1.019	0.953
B4DZQ5	cDNA FLJ51417, highly similar to Serine/threonine-protein kinase	1.404	1.587	1.398	1.159	1.472	1.359	1.473	1.473
Q96LD4	Tripartite motif-containing protein 47 OS=Homo sapiens GN=TRI	2.241	1.554	1.876	1.485	1.884	1.262	1.895	1.576
H3BS02	[3-methyl-2-oxobutanoate dehydrogenase [lipoamide]] kinase, m	0.781	0.656	0.566		0.611	0.973	0.929	0.640
Q86TW5	Full-length cDNA clone CS0DC006YI13 of Neuroblastoma of Hom	3.946	4.005	3.112	3.753	2.218	1.326	3.724	2.760
Q13043	Serine/threonine-protein kinase 4 OS=Homo sapiens GN=STK4 P	1.434	0.909	1.297	0.924	1.388	1.015	1.004	1.019
Q15773	Myeloid leukemia factor 2 OS=Homo sapiens GN=MLF2 PE=1 SV	1.283	1.194	1.337	1.569	1.519	1.387	1.723	1.955
P46199	Translation initiation factor IF-2, mitochondrial OS=Homo sapiens	1.189	1.335	1.065	0.955	0.906	1.127	1.150	1.059
Q8TF74	WAS/WASL-interacting protein family member 2 OS=Homo sapier	1.214	1.016	0.995	0.430	1.131	1.139	1.073	0.556
Q9BT73	Proteasome assembly chaperone 3 OS=Homo sapiens GN=PSMG	0.955	0.794	1.341	0.924	1.020	1.030	0.458	0.798
P26440	Isovaleryl-CoA dehydrogenase, mitochondrial OS=Homo sapiens (	1.318	1.436	1.204	1.343	1.037	1.202	1.368	1.425
B4E3D9	cDNA FLJ59152, highly similar to PITSLRE serine/threonine-prote	1.274	1.510	1.101	0.756	1.381	1.361	1.181	0.960
Q15126	Phosphomevalonate kinase OS=Homo sapiens GN=PMVK PE=1 S	1.106	0.541	0.825	0.966	0.919	0.938	0.977	0.868
O14657	Torsin-1B OS=Homo sapiens GN=TOR1B PE=1 SV=2 - [TOR1B_H	1.372	1.497	1.610	1.595	1.360	1.400	1.797	1.777
Q9H788	SH2 domain-containing protein 4A OS=Homo sapiens GN=SH2D4	1.384	0.934	1.356	0.874	0.979	1.071	1.265	0.960
Q8WYA6	Beta-catenin-like protein 1 OS=Homo sapiens GN=CTNNBL1 PE=	1.288	1.404	1.197	0.979	1.294	1.330	1.351	1.295
Q92544	Transmembrane 9 superfamily member 4 OS=Homo sapiens GN=	1.323	1.506	1.344	1.388	1.215	1.367	1.492	1.317
Q8IY95	Transmembrane protein 192 OS=Homo sapiens GN=TMEM192 PE	2.520	2.804	2.717	2.628	2.144	1.463	2.485	2.459
Q8TB37	Iron-sulfur protein NUBPL OS=Homo sapiens GN=NUBPL PE=1 S	1.312	1.532	1.271	1.021	0.891	1.177	1.281	1.134
Q9H0W8	Protein SMG9 OS=Homo sapiens GN=SMG9 PE=1 SV=1 - [SMG9_	1.606	1.137	1.227	1.158	1.224	1.256	1.306	1.032
Q96BP2	Coiled-coil-helix-coiled-coil-helix domain-containing protein 1 OS=	2.205	2.444	1.919	1.798	1.374	1.298	1.488	1.915
Q53F76	Polymerase (RNA) III (DNA directed) polypeptide C (62kD) variar	1.589	1.726	1.609	1.020	1.937	1.366	1.568	1.362
Q6P4A7	Sideroflexin-4 OS=Homo sapiens GN=SFXN4 PE=1 SV=1 - [SFXN	1.119	1.531	1.214	1.260	1.027	1.099	1.445	1.415
B4DZJ6	cDNA FLJ54706, highly similar to Ubiquitin-associated protein 2-li	1.170	1.903	1.379	0.987	1.080	1.375	1.434	1.323
Q9H814	Phosphorylated adapter RNA export protein OS=Homo sapiens G	1.596	1.564	1.756	1.171	1.692	1.409	1.418	1.431
Q53EP0	Fibronectin type III domain-containing protein 3B OS=Homo sapi	1.303	1.108	0.765	0.568	0.964	0.988	0.924	0.717

B2R758	cDNA, FLJ93295, highly similar to Homo sapiens glucosaminyl (N-	2.174	0.658	0.143	0.333	1.993	0.661	0.468	0.189
O95163	Elongator complex protein 1 OS=Homo sapiens GN=IKBKAP PE=	1.626	1.141	1.689	1.243	1.376	1.088	1.503	1.328
Q5JRA6	Melanoma inhibitory activity protein 3 OS=Homo sapiens GN=MI/	1.420	1.640	1.546	1.650	1.085	1.298	1.773	1.604
P37198	Nuclear pore glycoprotein p62 OS=Homo sapiens GN=NUP62 PE=	1.467	1.319	1.370	1.530	1.371	1.343	1.485	1.597
Q14573	Inositol 1,4,5-trisphosphate receptor type 3 OS=Homo sapiens GI	1.415	1.394	1.141	1.135	1.203	1.181	1.383	1.268
H0Y2S9	Myosin phosphatase Rho-interacting protein (Fragment) OS=Hom	1.625	1.301	1.095	0.914	1.202	1.242	1.121	1.018
D3DU05	Tousled-like kinase 2, isoform CRA_a OS=Homo sapiens GN=TLK	1.221	1.366	1.338	1.208	1.207	1.184	1.417	1.992
J3KNL6	Protein transport protein Sec16A OS=Homo sapiens GN=SEC16A	1.385	1.264	1.441	1.284	1.228	1.287	1.506	1.416
Q92643	GPI-anchor transamidase OS=Homo sapiens GN=PIGK PE=1 SV=	1.278	1.392	1.128	1.336	1.009	1.244	1.462	1.169
P18074	TFIIH basal transcription factor complex helicase XPD subunit OS	1.569	1.714	1.544	1.517	1.357	1.206	1.612	1.633
Q96G25	Mediator of RNA polymerase II transcription subunit 8 OS=Homo	1.210	1.338	0.907	0.555	1.242	1.130	1.038	0.886
P47224	Guanine nucleotide exchange factor MSS4 OS=Homo sapiens GN	2.294	1.854	2.642	2.151	1.739	1.202	1.841	2.124
A8K5D8	cDNA FLJ75934, highly similar to Homo sapiens vacuolar protein	1.165	0.929	1.206	0.991	1.156	1.102	1.196	1.078
Q9UH65	Switch-associated protein 70 OS=Homo sapiens GN=SWAP70 PE=	1.986	1.373	1.767	1.332	1.581	1.093	1.179	1.266
O95707	Ribonuclease P protein subunit p29 OS=Homo sapiens GN=POP4	2.399	2.913	1.698	1.284	1.561	1.337	1.352	1.885
Q9NU23	LYR motif-containing protein 2 OS=Homo sapiens GN=LYRM2 PE=	1.586	1.584	1.120	1.034	1.160	1.264	1.471	1.345
Q15654	Thyroid receptor-interacting protein 6 OS=Homo sapiens GN=TRI	1.749	1.426	1.218	1.024	1.672	1.090	1.433	1.523
Q9H2M9	Rab3 GTPase-activating protein non-catalytic subunit OS=Homo s	1.571	1.028	1.530	1.098	1.370	1.226	1.473	1.350
Q7Z5J4	Retinoic acid-induced protein 1 OS=Homo sapiens GN=RAI1 PE=	1.324	1.984	1.147	1.127	1.247	1.374	1.548	1.415
P19440	Gamma-glutamyltranspeptidase 1 OS=Homo sapiens GN=GGT1 F	1.268	1.444	1.170	1.305	1.171	1.049	1.222	1.257
Q9NWU1	3-oxoacyl-[acyl-carrier-protein] synthase, mitochondrial OS=Hom	1.087	1.200	1.095	0.951	0.936	1.297	1.088	1.067
O75208	Ubiquinone biosynthesis protein COQ9, mitochondrial OS=Homo s	1.328	1.231	0.916	0.980	1.041	1.062	1.205	1.115
Q9Y3E2	BolA-like protein 1 OS=Homo sapiens GN=BOLA1 PE=1 SV=1 - [I	1.012	0.913	0.982	0.920	0.917	0.996	0.913	0.861
B4DT01	cDNA FLJ60347, highly similar to Homo sapiens hydroxyacylglutat	1.801	1.871	1.782	1.858	1.583	1.210	1.784	2.191
P17676	CCAAT/enhancer-binding protein beta OS=Homo sapiens GN=CEI	0.902	0.761	0.365	0.174	0.806	0.847	0.532	0.346
C9J2P9	E3 ubiquitin-protein ligase Hakai (Fragment) OS=Homo sapiens G	1.364	1.535	1.245	0.928	1.382	1.295	1.524	1.374
B1AHQ6	Centromere protein M OS=Homo sapiens GN=CENPM PE=1 SV=1	1.317	1.295	1.829	1.500	1.087	1.244	1.298	1.330
Q9UKM7	Endoplasmic reticulum mannosyl-oligosaccharide 1,2-alpha-mann	1.228	1.191	0.916	0.825	1.238	1.306	1.434	1.106
A0A024R5F3	SAPS domain family, member 3, isoform CRA_c OS=Homo sapien	1.560	1.344	1.528	1.426	1.414	1.209	1.379	1.583
B7Z5Y7	cDNA FLJ53090, moderately similar to RNA-binding motif, single-s	1.216	1.039	0.736	0.529	1.237	1.037	0.842	0.641
Q9BSH4	Translational activator of cytochrome c oxidase 1 OS=Homo sapie	1.362	1.566	1.241	0.974	1.217	1.506	1.178	1.308
Q15758	Neutral amino acid transporter B(0) OS=Homo sapiens GN=SLC1	2.678	3.398	2.700	2.813	2.221	1.563	3.041	3.253
A0A024RBQ5	2'-5'-oligoadenylate synthetase 3, 100kDa, isoform CRA_a OS=Ho	1.385	1.138	1.383	0.933	1.216	1.087	1.268	1.033
Q9BTC0	Death-inducer obliterator 1 OS=Homo sapiens GN=DIDO1 PE=1	1.243	1.464	1.173	0.905	1.042	1.249	1.384	1.122
B4DV31	cDNA FLJ57497, highly similar to Peroxisomal biogenesis factor 3	1.359	1.715	1.524	1.452	1.306	1.385	1.744	1.438
B4E1G1	Derlin-1 OS=Homo sapiens GN=DERL1 PE=1 SV=1 - [B4E1G1_H	1.512	1.346	0.973	0.943	1.031	1.379	1.273	0.994
Q08AM6	Protein VAC14 homolog OS=Homo sapiens GN=VAC14 PE=1 SV=	1.861	1.416	1.771	1.324	1.520	1.047	1.351	1.312
Q9BRU9	rRNA-processing protein UTP23 homolog OS=Homo sapiens GN=	1.658	1.941	1.185	1.207	1.737	1.448	1.716	1.497
E9PQP6	Protein farnesyltransferase/geranylgeranyltransferase type-1 subu	1.255	1.048	1.252	1.330	1.310	0.985	1.239	1.220
A0A024R3J7	HCG2032701, isoform CRA_a OS=Homo sapiens GN=hCG_20327	1.697	1.612	1.483	1.446	1.402	1.304	1.763	1.387

Q12765	Secernin-1 OS=Homo sapiens GN=SCRN1 PE=1 SV=2 - [SCRN1_	1.405	0.845	1.590	1.279	1.523	0.885	1.590	1.700
B2R7I1	cDNA, FLJ93452, highly similar to Homo sapiens nucleoporin like	1.476	1.494	1.449	1.366	1.522	1.482	1.749	1.687
Q24K26	C9orf125 protein (Fragment) OS=Homo sapiens GN=C9orf125 PE	2.489	2.835	2.111	2.083	2.018	1.619	2.742	2.733
Q15648	Mediator of RNA polymerase II transcription subunit 1 OS=Homo	1.326	1.536	1.037	0.726	1.193	1.338	1.243	1.064
Q96F86	Enhancer of mRNA-decapping protein 3 OS=Homo sapiens GN=E	1.239	1.064	1.008	0.997	1.109	1.319	1.413	1.147
Q5U000	Cathepsin Z OS=Homo sapiens PE=2 SV=1 - [Q5U000_HUMAN]	1.389	1.230	1.334	1.660	1.505	1.043	1.461	1.632
Q15428	Splicing factor 3A subunit 2 OS=Homo sapiens GN=SF3A2 PE=1	1.325	1.337	1.011	0.859	1.311	1.241	1.198	1.030
H0YNH6	ER membrane protein complex subunit 9 OS=Homo sapiens GN=	3.640	3.706	3.714	3.247	3.333	1.454	3.574	3.494
D6R9Y1	Polyadenylate-binding protein-interacting protein 2 OS=Homo sa	1.412	0.957	0.969	0.492	1.138	1.161	1.272	0.914
Q5H9A7	Metalloproteinase inhibitor 1 OS=Homo sapiens GN=TIMP1 PE=1	1.715	1.803	0.987	1.096	1.529	1.724	1.679	1.076
A8K9A5	cDNA FLJ78114, highly similar to Homo sapiens thymidylate synt	1.654	1.424	1.406	0.755	1.206	1.485	1.794	1.458
M0R2T8	Histone H2AX (Fragment) OS=Homo sapiens GN=H2AFX PE=4 S	1.848	1.478	1.536	0.928	1.577	1.084	1.501	1.514
A8K9D2	Mitochondrial ribosomal protein L18, isoform CRA_b OS=Homo sa	1.522	2.063	1.412	1.508	1.230	1.261	1.587	1.549
Q59E90	Alpha-mannosidase (Fragment) OS=Homo sapiens PE=2 SV=1 -	1.431	1.326	1.343	1.414	1.500	1.311	1.545	1.520
Q9UK45	U6 snRNA-associated Sm-like protein LSm7 OS=Homo sapiens G	1.577	1.611	1.309	1.105	1.587	1.400	1.456	1.388
P55081	Microfibrillar-associated protein 1 OS=Homo sapiens GN=MFAP1	1.460	1.489	1.246	1.096	1.365	1.483	1.426	1.179
J3K000	PEPD protein OS=Homo sapiens GN=PEPD PE=2 SV=1 - [J3K000	1.584	1.252	1.845	1.589	1.147	0.948	1.323	1.390
V9GY01	Chromosome 15 open reading frame 23, isoform CRA_d OS=Hom	1.973	1.213	1.438	1.091	1.150	1.295	1.076	1.182
O14734	Acyl-coenzyme A thioesterase 8 OS=Homo sapiens GN=ACOT8 P	2.114	1.890	1.716	1.819	1.842	1.642	1.696	1.869
Q9BV23	Monoacylglycerol lipase ABHD6 OS=Homo sapiens GN=ABHD6 P	1.341	1.507	1.162	1.188	1.236	1.056	1.379	1.022
A0A087X105	Cell adhesion molecule 1 OS=Homo sapiens GN=CADM1 PE=4 S	1.316	2.176	1.442	1.117	1.429	1.268	1.743	1.740
A8QI98	DIS3 OS=Homo sapiens PE=2 SV=1 - [A8QI98_HUMAN]	1.309	1.378	1.186	0.758	1.220	1.186	1.049	0.796
Q9C005	Protein dpy-30 homolog OS=Homo sapiens GN=DPY30 PE=1 SV=	0.265	0.368	1.171	0.219	0.647	1.187	0.584	0.386
I6L976	Chromosome 6 open reading frame 57 OS=Homo sapiens GN=C6	1.415	0.803	1.220	0.548	1.327	0.789	1.073	0.650
Q9NVH0	Exonuclease 3'-5' domain-containing protein 2 OS=Homo sapiens	1.217	1.718	1.455	1.362	1.041	1.284	1.583	1.378
O43291	Kunitz-type protease inhibitor 2 OS=Homo sapiens GN=SPINT2 P	1.145	1.246	0.789	0.697	0.979	1.337	1.204	0.832
B7Z8A0	cDNA FLJ54727, highly similar to 6-phosphofructo-2-kinase/fructo	1.987	0.861	0.752	0.457	2.028	1.208	0.874	0.570
B2R7D2	cDNA, FLJ93389, highly similar to Homo sapiens multiple inositol	2.070	1.315	1.906	2.083	1.780	1.411	1.915	1.919
Q5JR04	Mov10, Moloney leukemia virus 10, homolog (Mouse), isoform CR	1.473	1.327	1.360	0.983	1.270	1.085	1.161	1.148
H7BXK9	ATP-binding cassette sub-family B member 6, mitochondrial (Frag	1.413	1.361	1.994	1.101	1.488	1.404	1.363	1.145
Q96IZ7	Serine/Arginine-related protein 53 OS=Homo sapiens GN=RSRC1	1.410	1.566	1.278	1.192	1.366	1.409	1.949	1.632
B7Z591	Transmembrane and coiled-coil domains 1, isoform CRA_a OS=H	1.355	1.354	1.211	1.095	1.020	1.192	1.380	1.175
Q96PE7	Methylmalonyl-CoA epimerase, mitochondrial OS=Homo sapiens	1.229	1.185	1.414	1.192	1.059	1.119	1.324	1.290
Q969V5	Mitochondrial ubiquitin ligase activator of NFKB 1 OS=Homo sapi	1.628	1.605	1.309	1.229	1.355	1.203	1.757	1.209
Q5T160	Probable arginine--tRNA ligase, mitochondrial OS=Homo sapiens	1.180	1.315	0.997	1.002	0.945	1.056	0.999	1.014
Q10469	Alpha-1,6-mannosyl-glycoprotein 2-beta-N-acetylglucosaminyltran	1.859	2.309	1.546	1.650	1.634	1.741	1.896	1.423
Q9UL33	Trafficking protein particle complex subunit 2-like protein OS=Ho	1.615	1.120	1.526	1.173	1.412	1.073	1.073	1.139
Q9Y312	Protein AAR2 homolog OS=Homo sapiens GN=AAR2 PE=1 SV=2	1.301	1.386	1.492	1.149	1.336	1.459	1.600	1.409
Q69YP1	Putative uncharacterized protein DKFZp762M013 (Fragment) OS=	1.400	1.550	1.317	1.026	1.250	1.352	1.378	1.045
Q13418	Integrin-linked protein kinase OS=Homo sapiens GN=ILK PE=1 S	1.317	0.909	1.077	0.752	1.202	0.955	1.019	0.796

Q96K19	E3 ubiquitin-protein ligase RNF170 OS=Homo sapiens GN=RNF17	1.762	1.785	1.456	1.478	1.353	1.280	1.745	1.602
D6RF48	Syntaxin-18 OS=Homo sapiens GN=STX18 PE=1 SV=1 - [D6RF48	2.952	3.364	2.419	2.460	1.818	1.669	2.029	2.011
P05067	Amyloid beta A4 protein OS=Homo sapiens GN=APP PE=1 SV=3	1.468	1.552	0.865	0.483	1.355	1.408	1.371	0.696
Q15388	Mitochondrial import receptor subunit TOM20 homolog OS=Homo	3.017	1.499	1.260	1.590	0.940	1.464	1.408	1.366
Q9NVA1	Ubiquinol-cytochrome-c reductase complex assembly factor 1 OS=	1.875	1.901	1.768	1.585	1.578	1.310	2.071	1.694
Q53GS7	Nucleoporin GLE1 OS=Homo sapiens GN=GLE1 PE=1 SV=2 - [GL	1.683	1.732	1.494	1.497	1.664	1.588	1.738	1.907
Q9NV56	MRG/MORF4L-binding protein OS=Homo sapiens GN=MRGBP PE=	0.985	0.769	0.685	0.552	0.983	1.285	0.828	0.747
Q92575	UBX domain-containing protein 4 OS=Homo sapiens GN=UBXN4	1.248	1.268	1.004	0.846	1.052	1.221	1.222	0.954
Q9Y3Z3	Deoxynucleoside triphosphate triphosphohydrolase SAMHD1 OS=	1.659	1.570	1.570	1.278	1.481	1.250	1.502	1.375
B4DW33	cDNA FLJ54187, highly similar to Homo sapiens polymerase (RNA	1.667	2.656	1.737	1.520	1.296	1.475	2.000	1.755
F8VXG7	Protein SCAF11 OS=Homo sapiens GN=SCAF11 PE=1 SV=1 - [F8	1.406	1.659	1.134	1.033	1.206	1.364	1.637	1.337
Q16706	Alpha-mannosidase 2 OS=Homo sapiens GN=MAN2A1 PE=1 SV=	1.419	1.611	1.378	1.398	1.258	1.347	1.582	1.393
B3KQ33	cDNA FLJ32715 fis, clone TESTI2000784, highly similar to Import	2.087	1.398	2.251	1.427	1.759	1.141	1.507	1.801
Q13153	Serine/threonine-protein kinase PAK 1 OS=Homo sapiens GN=PA	1.068	1.030	1.413	1.506	1.054	1.136	2.589	1.322
Q2KRZ2	E4 17 kDa protein GN=E4_17K PE=4 SV=1 - [Q2KRZ2_ADE05]	0.924	1.075	1.702	1.324	0.520	1.191	1.607	2.025
C9JQB1	Nucleoside diphosphate kinase OS=Homo sapiens GN=NME6 PE=	0.913	1.131	1.123	0.938	0.758	1.010	0.946	1.006
Q658J0	Putative GTP cyclohydrolase 1 type 2 (Fragment) OS=Homo sapie	1.265	0.995	1.182	1.102	1.115	1.078	1.175	0.955
Q53FA7	Quinone oxidoreductase PIG3 OS=Homo sapiens GN=TP53I3 PE=	1.038	0.831	1.024	0.811	0.938	0.979	1.106	0.904
O43665	Regulator of G-protein signaling 10 OS=Homo sapiens GN=RGS1	0.815	0.616	0.807	0.661	1.176	1.152	1.153	0.850
J9JIC5	Protein Njmu-R1 OS=Homo sapiens GN=C17orf75 PE=1 SV=1 - [	1.769	1.280	1.822	1.224	0.939	1.004	1.689	1.060
Q86Y79	Probable peptidyl-tRNA hydrolase OS=Homo sapiens GN=PTRH1	1.014	1.260	0.808	0.924	1.104	1.158	1.173	1.000
Q96RS6	NudC domain-containing protein 1 OS=Homo sapiens GN=NUDCI	1.933	1.425	2.088	1.283	1.456	1.156	1.794	1.586
Q96C86	m7GpppX diphosphatase OS=Homo sapiens GN=DCPS PE=1 SV=	1.591	1.277	1.562	1.313	1.375	1.209	1.465	1.498
O15438	Canalicular multispecific organic anion transporter 2 OS=Homo sa	1.328	1.794	1.475	1.499	1.091	1.319	1.255	1.323
O15258	Protein RER1 OS=Homo sapiens GN=RER1 PE=1 SV=1 - [RER1_	2.848	3.215	2.602	2.221	2.313	1.426	2.725	2.336
B4DLW4	cDNA FLJ60300, highly similar to Homo sapiens protein phosphat	1.325	1.263	1.213	0.994	1.187	1.337	1.135	1.036
Q6SPF0	Atherin OS=Homo sapiens GN=SAMD1 PE=1 SV=1 - [SAMD1_HU	1.073	1.130	1.295	1.101	1.280	1.426	1.536	1.562
H0Y2S1	Putative GTP-binding protein 6 OS=Homo sapiens GN=GTPBP6 P	1.321	1.378	1.086	0.968	1.072	1.202	1.171	0.981
C9JBY7	28S ribosomal protein S33, mitochondrial OS=Homo sapiens GN=	1.291	1.738	1.192	1.081	0.992	0.977	1.252	1.380
O14646	Chromodomain-helicase-DNA-binding protein 1 OS=Homo sapien	1.662	1.906	1.300	1.288	1.477	1.430	1.472	1.424
Q8NB16	Mixed lineage kinase domain-like protein OS=Homo sapiens GN=	1.207	0.931	1.191	0.805	1.088	1.062	1.103	0.855
Q9UGV2	Protein NDRG3 OS=Homo sapiens GN=NDRG3 PE=1 SV=2 - [ND	1.296	0.855	1.284	0.893	1.265	1.060	1.277	1.090
B4DRU9	cDNA FLJ57179, highly similar to Homo sapiens ATP-binding cass	1.552	1.040	1.454	1.099	1.261	1.101	1.375	1.120
A8K4R1	cDNA FLJ77420, highly similar to Homo sapiens cancer susceptibi	2.485	2.719	2.198	2.152	1.858	1.278	2.284	2.318
B4DPC0	cDNA FLJ52713, moderately similar to Mus musculus leucine rich	2.090	1.657	1.847	1.406	1.984	1.226	1.865	1.660
Q13131	5'-AMP-activated protein kinase catalytic subunit alpha-1 OS=Hor	1.343	0.969	1.286	1.121	1.202	0.902	0.986	0.969
Q86YQ8	Copine-8 OS=Homo sapiens GN=CPNE8 PE=1 SV=2 - [CPNE8_HU	1.630	2.383	1.870	2.133	1.538	1.239	1.522	1.983
H3BN86	Methyltransferase-like protein 9 (Fragment) OS=Homo sapiens GI	1.417	1.492	1.137	1.029	1.331	1.373	1.516	0.964
Q8IUR0	Trafficking protein particle complex subunit 5 OS=Homo sapiens (	1.645	1.569	1.464	1.218	1.455	1.180	1.515	1.581
B2RBB2	cDNA, FLJ95416, highly similar to Homo sapiens phosphatidylinos	2.474	2.255	3.131	2.378	2.423	1.442	2.260	2.518

Q8N4A0	Polypeptide N-acetylgalactosaminyltransferase 4 OS=Homo sapie	1.181	1.334	0.999	1.136	1.229	1.291	1.271	1.024
H0YHG0	Uncharacterized protein (Fragment) OS=Homo sapiens PE=4 SV=	1.183	1.547	0.946	0.783	1.294	1.230	1.274	1.031
Q9UBD5	Origin recognition complex subunit 3 OS=Homo sapiens GN=ORC	1.760	1.972	1.724	1.615	1.470	1.329	1.201	1.687
Q6IRX3	RNA binding motif protein 7 OS=Homo sapiens GN=RBM7 PE=2 S	1.410	1.314	0.962	0.645	1.207	1.191	1.101	0.825
O00257	E3 SUMO-protein ligase CBX4 OS=Homo sapiens GN=CBX4 PE=1	1.476	1.682	0.731	0.638	1.171	1.269	1.051	0.724
A0A024R819	Nudix (Nucleoside diphosphate linked moiety X)-type motif 1, isof	1.217	0.860	1.346	1.269	1.088	1.004	1.212	1.292
Q8IYN9	Focal adhesion kinase 1 OS=Homo sapiens GN=PTK2 PE=1 SV=1	1.882	1.230	1.767	1.311	1.583	1.213	1.602	1.112
O95487	Protein transport protein Sec24B OS=Homo sapiens GN=SEC24B	1.777	2.125	1.970	1.945	1.640	2.068	2.105	2.037
Q9BT22	Chitobiosyldiphosphodolichol beta-mannosyltransferase OS=Homo	1.373	1.428	1.305	1.317	1.206	1.265	1.482	1.309
C9JE98	Nuclear receptor corepressor 2 OS=Homo sapiens GN=NCOR2 PE	1.220	1.414	1.054	0.758	1.223	1.560	1.360	1.399
Q96I51	Williams-Beuren syndrome chromosomal region 16 protein OS=H	1.372	1.629	1.245	1.073	1.197	1.321	1.327	1.109
B4DEQ0	cDNA FLJ59482, highly similar to Electron transfer flavoprotein-ub	1.705	2.122	1.863	1.678	1.300	1.141	1.595	1.693
Q9H9Y6	DNA-directed RNA polymerase I subunit RPA2 OS=Homo sapiens	1.582	1.795	1.775	1.550	1.643	1.542	1.907	1.559
Q9NZD8	Maspardin OS=Homo sapiens GN=SPG21 PE=1 SV=1 - [SPG21_H	1.823	1.953	2.408	1.268	1.730	1.560	1.765	1.458
B4DG42	cDNA FLJ53753, highly similar to Myeloid-associated differentiatio	2.783	4.226	2.563	2.945	2.314	1.624	1.646	2.348
Q9UHB7	AF4/FMR2 family member 4 OS=Homo sapiens GN=AFF4 PE=1 S	1.841	2.567	1.914	1.888	1.448	1.950	2.034	2.385
B2R8U0	cDNA, FLJ94063, highly similar to Homo sapiens activating transc	1.092	1.311	1.228	0.866	1.037	1.215	1.132	1.010
O75937	DnaJ homolog subfamily C member 8 OS=Homo sapiens GN=DN	1.228	1.030	1.089	0.772	1.217	1.217	1.034	0.849
B3KS62	cDNA FLJ35573 fis, clone SPLEN2005927, highly similar to Tripart	1.503	1.589	1.235	0.889	1.503	1.425	1.525	1.166
Q9Y375	Complex I intermediate-associated protein 30, mitochondrial OS=	0.662	0.940	0.898	1.095	0.662	1.263	1.020	0.956
Q8IYQ7	Threonine synthase-like 1 OS=Homo sapiens GN=THNSL1 PE=1	1.164	1.213	1.052	1.058	1.038	1.153	1.033	1.030
A0A087WUK0	AP2-associated protein kinase 1 OS=Homo sapiens GN=AAK1 PE=	1.042	0.826	1.042	0.811	1.236	0.914	1.079	0.915
Q8IY57	YY1-associated factor 2 OS=Homo sapiens GN=YAF2 PE=1 SV=3	0.285	0.322	0.206	0.274	0.578	1.195	0.670	0.336
D3DP46	Signal peptidase complex subunit 3 homolog (S. cerevisiae), isofo	1.501	1.351	1.295	1.194	1.130	1.218	1.337	1.106
Q9P0U4	CXXC-type zinc finger protein 1 OS=Homo sapiens GN=CXXC1 PE	1.437	1.743	1.109	0.644	1.410	1.271	1.338	1.024
Q53HH3	General transcription factor IIH, polypeptide 4, 52kDa variant (Fra	1.313	1.513	1.357	1.311	1.374	1.276	1.464	1.470
B3KSN3	cDNA FLJ36686 fis, clone UTERU2008027, highly similar to ATP-b	1.351	1.298	1.153	1.183	1.067	1.103	1.332	1.194
Q15642	Cdc42-interacting protein 4 OS=Homo sapiens GN=TRIP10 PE=1	1.355	0.862	1.104	0.876	1.096	0.894	0.996	0.892
Q8IWA4	Mitofusin-1 OS=Homo sapiens GN=MFN1 PE=1 SV=2 - [MFN1_H	1.303	1.621	1.323	1.151	1.197	1.471	1.319	1.144
F8VQD9	Autophagy-related protein 101 (Fragment) OS=Homo sapiens GN	1.889	1.447	1.237	1.058	1.501	1.224	1.031	1.262
Q96F63	Coiled-coil domain-containing protein 97 OS=Homo sapiens GN=(	1.721	1.528	1.789	1.267	1.482	1.581	1.748	1.457
O60443	Non-syndromic hearing impairment protein 5 OS=Homo sapiens (	1.081	0.821	1.187	0.832	1.020	0.981	0.863	0.870
Q86UK7	Zinc finger protein 598 OS=Homo sapiens GN=ZNF598 PE=1 SV=	1.355	1.635	1.267	1.001	1.315	1.216	1.579	1.157
Q53F51	FGF intracellular binding protein isoform b variant (Fragment) OS	1.051	1.060	1.075	1.106	1.036	1.043	1.178	1.029
Q12962	Transcription initiation factor TFIID subunit 10 OS=Homo sapiens	1.009	1.185	0.940	0.864	1.008	1.444	1.071	1.095
E9PRI4	Ribosomal protein S6 kinase OS=Homo sapiens GN=RPS6KA1 PE	1.317	1.121	1.424	1.202	1.230	1.013	1.383	1.254
B4DFY5	Mitogen-activated protein kinase kinase 1, isoform CRA_d OS=Ho	1.513	1.151	1.501	1.296	1.391	1.029	1.459	1.296
Q96EL2	28S ribosomal protein S24, mitochondrial OS=Homo sapiens GN=	1.348	1.545	1.143	1.131	1.074	1.032	1.439	1.276
A8K8A4	cDNA FLJ77640, highly similar to Homo sapiens copine II (CPNE2	1.093	1.564	1.201	1.427	1.008	1.149	1.311	1.423
P27216	Annexin A13 OS=Homo sapiens GN=ANXA13 PE=1 SV=3 - [ANX	1.637	2.353	1.978	1.974	2.075	1.805	2.486	2.477

Q5VWZ2	Lysophospholipase-like protein 1 OS=Homo sapiens GN=LYPLAL1	1.272	1.184	1.266	1.217	1.096	1.016	1.203	1.226
Q9UK59	Lariat debranching enzyme OS=Homo sapiens GN=DBR1 PE=1 S	1.376	1.327	1.145	0.888	1.598	1.315	1.332	1.088
Q5T8U5	Surfeit 4 OS=Homo sapiens GN=SURF4 PE=1 SV=1 - [Q5T8U5_H	1.385	1.463	1.359	1.259	1.277	1.175	1.461	1.198
Q86VM9	Zinc finger CCCH domain-containing protein 18 OS=Homo sapien	1.357	1.607	1.201	1.213	1.328	1.344	1.524	1.507
H0Y984	ADP-ribosyl cyclase/cyclic ADP-ribose hydrolase 2 (Fragment) OS=	1.162	1.174	1.093	1.390	1.086	1.103	1.070	1.067
Q96QC0	Serine/threonine-protein phosphatase 1 regulatory subunit 10 OS	1.186	1.455	0.795	0.555	1.098	1.261	1.074	0.903
B7ZKW4	PDDC1 protein OS=Homo sapiens GN=PDDC1 PE=2 SV=1 - [B7Z	1.323	0.962	1.397	1.115	1.263	1.445	1.102	1.190
Q9Y446	Plakophilin-3 OS=Homo sapiens GN=PKP3 PE=1 SV=1 - [PKP3_H	1.476	1.483	1.117	0.929	1.298	1.020	1.613	1.497
A0A024RAJ7	G elongation factor, mitochondrial 2, isoform CRA_c OS=Homo sa	1.059	1.162	1.097	0.952	0.956	1.121	1.137	1.305
Q8N1G2	Cap-specific mRNA (nucleoside-2'-O-)-methyltransferase 1 OS=H	1.228	1.070	1.197	0.929	1.122	1.184	1.167	0.954
Q56P03	E2F-associated phosphoprotein OS=Homo sapiens GN=EAPP PE=	1.974	2.428	1.663	1.389	2.146	1.452	2.073	1.557
K7EMY9	Cold-inducible RNA-binding protein OS=Homo sapiens GN=CIRBF	3.674	3.597	1.958	1.802	3.289	1.572	2.477	1.788
E9PK80	NAD-dependent protein deacetylase sirtuin-3, mitochondrial OS=	0.806	0.943	0.790	0.758	0.839	1.015	0.934	0.697
P61764	Syntaxin-binding protein 1 OS=Homo sapiens GN=STXBP1 PE=1	1.142	1.192	1.058	0.830	1.159	1.254	1.116	0.944
B2RAK1	cDNA, FLJ94965, highly similar to Homo sapiens leucyl/cystinyl ar	1.666	1.860	1.581	1.459	1.702	1.142	1.774	1.325
Q8TBM8	DnaJ homolog subfamily B member 14 OS=Homo sapiens GN=DI	1.206	1.278	1.246	1.334	1.190	1.403	1.376	1.237
Q96C23	Aldose 1-epimerase OS=Homo sapiens GN=GALM PE=1 SV=1 - [	1.051	0.709	1.236	0.947	1.026	0.938	0.921	0.920
O00629	Importin subunit alpha-3 OS=Homo sapiens GN=KPNA4 PE=1 SV	1.646	1.253	1.592	1.399	1.309	1.054	1.609	1.544
B3KVB0	cDNA FLJ16322 fis, clone SPLEN2037678, highly similar to Proteir	1.113	1.306	1.187	1.146	0.971	1.172	1.381	1.222
O00505	Importin subunit alpha-4 OS=Homo sapiens GN=KPNA3 PE=1 SV	1.891	1.348	1.712	1.231	1.528	1.243	1.634	1.636
P83436	Conserved oligomeric Golgi complex subunit 7 OS=Homo sapiens	2.170	2.055	1.797	1.548	1.456	1.208	1.405	1.484
O43847	Nardilysin OS=Homo sapiens GN=NRD1 PE=1 SV=2 - [NRDC_HU	1.666	0.989	1.515	1.217	1.359	1.186	1.437	1.250
A0AVT1	Ubiquitin-like modifier-activating enzyme 6 OS=Homo sapiens GN	1.338	0.992	1.384	1.172	1.139	1.035	1.276	1.173
Q96EK5	KIF1-binding protein OS=Homo sapiens GN=KIAA1279 PE=1 SV=	1.559	1.334	1.697	1.325	1.459	1.454	1.648	1.536
P14373	Zinc finger protein RFP OS=Homo sapiens GN=TRIM27 PE=1 SV=	1.515	1.870	1.389	1.559	1.505	1.503	1.853	1.783
Q9H5X1	MIP18 family protein FAM96A OS=Homo sapiens GN=FAM96A PE	1.456	1.073	1.156	1.317	1.232	0.600	1.936	1.195
Q8WY36	HMG box transcription factor BBX OS=Homo sapiens GN=BBX PE	1.767	2.420	1.498	1.307	1.528	1.376	1.880	2.118
B4E1N6	cDNA FLJ50355, highly similar to RAF proto-oncogene serine/thre	1.816	1.356	1.492	1.116	1.111	1.079	1.722	0.899
Q8NDX6	Zinc finger protein 740 OS=Homo sapiens GN=ZNF740 PE=1 SV=	1.486	1.888	1.555	1.221	0.959	1.147	1.315	1.231
A4D0Y1	Wiskott-Aldrich syndrome-like OS=Homo sapiens GN=WASL PE=4	1.478	0.815	1.192	0.913	1.100	0.863	1.098	0.844
Q96P70	Importin-9 OS=Homo sapiens GN=IPO9 PE=1 SV=3 - [IPO9_HUI	1.571	1.239	1.588	1.447	1.357	1.189	1.392	1.300
D6W5R0	HCG1741805, isoform CRA_a OS=Homo sapiens GN=hCG_17418	1.466	1.826	1.401	1.285	1.286	1.460	1.657	1.473
P60033	CD81 antigen OS=Homo sapiens GN=CD81 PE=1 SV=1 - [CD81_	3.343	3.708	3.855	4.362	2.764	1.897	3.489	2.631
Q9UHN6	Transmembrane protein 2 OS=Homo sapiens GN=TMEM2 PE=1 S	1.318	1.204	0.922	0.920	1.136	1.083	0.908	0.773
A0A024R2M8	Xeroderma pigmentosum, complementation group C, isoform CR/	1.014	1.595	1.062	1.189	1.154	1.219	1.350	1.445
P50336	Protoporphyrinogen oxidase OS=Homo sapiens GN=PPOX PE=1 S	1.157	1.236	0.787	0.868	0.858	1.004	0.946	0.796
B2R9Y2	cDNA, FLJ94609 OS=Homo sapiens PE=2 SV=1 - [B2R9Y2_HUM/	1.325	2.045	1.470	1.035	1.205	1.499	1.396	1.449
O14561	Acyl carrier protein, mitochondrial OS=Homo sapiens GN=NDUFA	2.797	2.960	2.365	2.754	2.787	1.782	3.494	2.889
Q9H857	5'-nucleotidase domain-containing protein 2 OS=Homo sapiens G	0.969	0.994	0.840	0.817	0.872	1.043	1.027	1.034
P52943	Cysteine-rich protein 2 OS=Homo sapiens GN=CRIP2 PE=1 SV=1	1.059	0.761	1.113	0.959	0.960	0.922	0.966	0.830

Q53F19	Uncharacterized protein C17orf85 OS=Homo sapiens GN=C17orf8	1.267	1.496	1.302	1.044	1.295	1.398	1.483	1.220
Q969N2	GPI transamidase component PIG-T OS=Homo sapiens GN=PIGT	1.483	1.527	1.307	1.239	1.122	1.175	1.346	1.324
Q8N5M1	ATP synthase mitochondrial F1 complex assembly factor 2 OS=Ho	2.062	2.248	2.200	2.180	1.590	1.271	1.915	1.909
A0A024R6M2	KIAA2010, isoform CRA_a OS=Homo sapiens GN=KIAA2010 PE=	2.991	2.843	2.022	1.588	2.468	1.402	1.785	1.349
B4DU58	cDNA FLJ51488, highly similar to Macrophage capping protein OS	1.539	0.936	1.342	0.956	1.313	0.936	1.202	1.009
B2R506	Transcription initiation factor IIA subunit 2 OS=Homo sapiens PE=	1.396	1.274	1.287	1.021	1.207	1.257	1.322	1.316
Q96DP5	Methionyl-tRNA formyltransferase, mitochondrial OS=Homo sapie	1.239	1.497	1.198	1.057	0.944	0.935	1.346	1.206
Q8IXI2	Mitochondrial Rho GTPase 1 OS=Homo sapiens GN=RHOT1 PE=1	1.698	1.954	1.777	1.915	1.265	1.256	2.041	1.890
Q9NXX6	Non-structural maintenance of chromosomes element 4 homolog	1.781	2.043	1.703	1.597	1.837	1.412	1.816	1.681
O00264	Membrane-associated progesterone receptor component 1 OS=H	1.620	1.637	1.477	1.448	1.121	1.273	1.436	1.334
Q7Z4A2	Ankyrin repeat and SOCS box-containing 9 OS=Homo sapiens GN	1.087	0.751	1.059	0.978	1.092	1.030	1.132	1.003
B4E0H8	cDNA FLJ60385, highly similar to Integrin alpha-3 OS=Homo sapi	1.148	1.432	1.079	1.085	1.076	1.142	1.000	0.909
A8K4P8	cDNA FLJ75337 OS=Homo sapiens PE=2 SV=1 - [A8K4P8_HUMA	1.632	1.902	1.610	1.533	1.504	1.378	1.752	1.654
O15254	Peroxisomal acyl-coenzyme A oxidase 3 OS=Homo sapiens GN=A	2.063	1.939	1.677	1.686	1.406	1.290	1.653	2.066
P54278	Mismatch repair endonuclease PMS2 OS=Homo sapiens GN=PMS	1.024	0.944	1.035	0.756	1.105	1.078	0.784	0.661
B4E396	cDNA FLJ59612, highly similar to Lactadherin OS=Homo sapiens	1.430	1.425	0.955	0.930	1.211	1.289	1.192	0.951
P80217	Interferon-induced 35 kDa protein OS=Homo sapiens GN=IFI35 I	1.425	1.151	1.318	1.056	1.022	0.919	0.776	0.985
Q9Y5Z4	Heme-binding protein 2 OS=Homo sapiens GN=HEBP2 PE=1 SV=	0.991	0.444	1.483	0.887	1.127	0.949	0.789	1.015
B2R6N9	cDNA, FLJ93042, highly similar to Homo sapiens signal sequence	1.078	1.131	0.917	0.941	0.851	1.216	0.982	0.933
A8K874	cDNA FLJ77588, highly similar to Homo sapiens MAK10 homolog,	1.864	2.075	2.202	1.612	2.076	1.277	1.945	1.852
Q96RQ3	Methylcrotonoyl-CoA carboxylase subunit alpha, mitochondrial OS	1.451	1.309	1.076	1.276	1.094	1.114	1.360	1.222
P58557	Putative ribonuclease OS=Homo sapiens GN=YBEY PE=1 SV=2 -	2.091	2.152	2.054	1.795	1.676	1.279	2.161	1.904
Q8WWC4	Uncharacterized protein C2orf47, mitochondrial OS=Homo sapien	1.201	1.357	1.040	0.981	0.998	1.060	1.279	1.242
Q9Y3C8	Ubiquitin-fold modifier-conjugating enzyme 1 OS=Homo sapiens (	1.733	1.291	2.102	1.824	1.715	1.226	1.705	1.639
P48509	CD151 antigen OS=Homo sapiens GN=CD151 PE=1 SV=3 - [CD1	2.708	3.597	2.506	3.342	2.023	1.366	2.559	2.312
M0QXT0	Upstream stimulatory factor 2 (Fragment) OS=Homo sapiens GN=	2.052	2.392	2.002	2.304	1.372	1.559	2.361	2.745
Q9NXV2	BTB/POZ domain-containing protein KCTD5 OS=Homo sapiens GI	2.844	1.960	1.733	0.999	2.473	1.866	2.421	1.759
Q96G03	Phosphoglucomutase-2 OS=Homo sapiens GN=PGM2 PE=1 SV=4	1.288	1.365	1.739	1.375	1.192	1.123	1.382	1.210
Q9Y5S2	Serine/threonine-protein kinase MRCK beta OS=Homo sapiens GI	1.244	0.909	1.047	0.667	1.107	0.982	0.974	0.783
Q6UW63	KDEL motif-containing protein 1 OS=Homo sapiens GN=KDELC1	1.818	1.490	2.112	2.487	2.043	1.574	2.359	2.621
Q9BV81	ER membrane protein complex subunit 6 OS=Homo sapiens GN=	1.528	1.386	3.008	2.605	1.054	1.085	1.401	1.776
B4DLL7	cDNA FLJ57319, highly similar to Sorting nexin-17 OS=Homo sap	1.254	0.797	0.900	0.542	1.257	1.136	0.984	0.795
P12532	Creatine kinase U-type, mitochondrial OS=Homo sapiens GN=CKI	1.184	1.294	1.488	1.647	1.019	1.402	1.639	1.903
Q96TC7	Regulator of microtubule dynamics protein 3 OS=Homo sapiens (	1.167	1.480	1.453	1.356	1.046	1.099	1.543	1.459
B2R7B1	cDNA, FLJ93361, highly similar to Homo sapiens membrane intera	3.755	3.295	3.349	3.127	2.609	1.743	2.621	2.857
A8K654	cDNA FLJ75178, highly similar to Homo sapiens methyl-CpG bind	1.895	1.949	1.571	2.018	1.394	1.525	1.955	2.119
P55212	Caspase-6 OS=Homo sapiens GN=CASP6 PE=1 SV=2 - [CASP6_H	2.494	1.748	2.464	1.970	2.068	1.310	1.771	2.136
K7ERI9	Truncated apolipoprotein C-I (Fragment) OS=Homo sapiens GN=/	4.372	5.347	4.150	3.445	3.858	2.252	4.739	3.938
B4DRG7	Condensin complex subunit 2 OS=Homo sapiens PE=2 SV=1 - [B	1.344	0.932	1.049	0.786	1.210	0.984	0.995	0.865
Q92733	Proline-rich protein PRCC OS=Homo sapiens GN=PRCC PE=1 SV=	1.330	1.466	1.285	1.040	1.172	1.359	1.109	0.978

A8K1E1	cDNA FLJ75589, highly similar to Homo sapiens mutS homolog 3	1.048	1.378	1.408	1.149	0.944	1.118	1.210	1.320
P54802	Alpha-N-acetylglucosaminidase OS=Homo sapiens GN=NAGLU PE	1.384	1.244	1.416	1.070	1.288	1.131	1.566	1.182
Q5QPQ0	Acyl-protein thioesterase 2 OS=Homo sapiens GN=LYPLA2 PE=1	1.169	0.806	1.174	0.996	1.161	1.022	1.195	1.053
Q9Y5B6	PAX3- and PAX7-binding protein 1 OS=Homo sapiens GN=PAXBP	1.458	1.578	1.382	1.353	1.433	1.406	1.619	1.621
Q12800	Alpha-globin transcription factor CP2 OS=Homo sapiens GN=TFC	0.996	0.885	1.010	1.012	0.986	1.414	1.308	1.227
F5GZ78	Paxillin OS=Homo sapiens GN=PXN PE=1 SV=1 - [F5GZ78_HUM/	1.564	1.448	1.075	0.630	1.261	0.860	1.056	0.938
Q9H8N9	cDNA FLJ13353 fis, clone OVARC1002182, weakly similar to BETA	1.808	2.468	1.512	1.478	2.055	1.311	1.417	2.046
P0DI81	Trafficking protein particle complex subunit 2 OS=Homo sapiens (	1.981	1.493	1.388	1.171	0.963	1.168	1.129	1.256
B4E0N1	cDNA FLJ58735, highly similar to Helicase SKI2W (EC 3.6.1.-) OS	1.441	0.915	1.185	0.953	1.236	1.011	1.171	1.061
Q9NWH9	SAFB-like transcription modulator OS=Homo sapiens GN=SLTM P	1.350	1.427	1.051	0.857	1.227	1.308	1.360	1.159
Q9HCG8	Pre-mRNA-splicing factor CWC22 homolog OS=Homo sapiens GN	1.604	1.757	1.519	1.265	1.790	1.639	1.599	1.406
Q8WVQ1	Soluble calcium-activated nucleotidase 1 OS=Homo sapiens GN=(	1.196	1.606	0.992	0.941	1.054	1.266	1.350	1.065
O43747	AP-1 complex subunit gamma-1 OS=Homo sapiens GN=AP1G1 P	1.709	1.612	1.755	1.282	1.495	1.156	1.493	1.476
Q6P3W7	SCY1-like protein 2 OS=Homo sapiens GN=SCYL2 PE=1 SV=1 - [	1.452	1.215	1.650	1.081	1.283	1.101	1.673	1.850
C9JJV1	Protein TSSC4 (Fragment) OS=Homo sapiens GN=TSSC4 PE=1 S	0.999	1.263	1.108	0.944	1.000	1.243	1.002	1.018
Q8N567	Zinc finger CCHC domain-containing protein 9 OS=Homo sapiens	0.969	1.181	0.713	0.493	1.017	1.256	0.899	0.629
A0A024R3R5	Lamin B receptor, isoform CRA_a OS=Homo sapiens GN=LBR PE=	1.903	1.558	1.171	1.151	1.378	1.211	1.787	1.138
B3KSI7	cDNA FLJ36374 fis, clone THYMU2008185, highly similar to Xaa-F	1.223	0.951	1.301	1.124	1.120	0.932	1.258	1.250
Q15208	Serine/threonine-protein kinase 38 OS=Homo sapiens GN=STK38	1.352	0.885	1.072	0.745	0.921	1.079	1.119	1.022
B2RC06	cDNA, FLJ95791, highly similar to Homo sapiens aurora kinase B	1.100	1.647	1.002	0.818	1.246	1.438	1.366	0.980
O75844	CAAX prenyl protease 1 homolog OS=Homo sapiens GN=ZMPSTE	1.694	1.582	1.518	1.538	1.216	1.247	1.502	1.558
Q9NY27	Serine/threonine-protein phosphatase 4 regulatory subunit 2 OS=	1.310	1.200	1.035	0.595	1.249	1.136	0.905	0.703
Q6ZWJ1	Syntaxin-binding protein 4 OS=Homo sapiens GN=STXBP4 PE=1	1.577	1.426	1.713	1.308	1.534	1.289	1.674	1.308
Q9Y508	E3 ubiquitin-protein ligase RNF114 OS=Homo sapiens GN=RNF11	1.646	1.742	2.748	1.900	2.308	1.408	1.961	2.773
P42568	Protein AF-9 OS=Homo sapiens GN=MLLT3 PE=1 SV=2 - [AF9_H	1.160	1.289	0.973	0.895	1.043	1.233	0.999	1.054
Q9UEL4	Putative uncharacterized protein (Fragment) OS=Homo sapiens P	1.470	1.350	0.777	0.973	1.814	1.215	1.193	0.972
Q6IQ43	PTPN9 protein OS=Homo sapiens GN=PTPN9 PE=2 SV=1 - [Q6I(	1.588	1.721	1.332	1.138	1.635	1.225	1.576	1.508
Q96CU9	FAD-dependent oxidoreductase domain-containing protein 1 OS=	1.772	1.794	1.452	1.532	1.351	1.180	1.691	1.722
P46736	Lys-63-specific deubiquitinase BRCC36 OS=Homo sapiens GN=BF	1.411	1.181	1.327	1.017	1.375	1.047	1.328	1.070
B8ZZU6	Cyclic AMP-dependent transcription factor ATF-2 OS=Homo sapie	0.907	1.225	1.024	1.047	1.021	1.114	1.134	1.167
F8WB06	Ataxin-2 OS=Homo sapiens GN=ATXN2 PE=1 SV=1 - [F8WB06_H	1.490	1.347	1.220	0.912	1.360	1.107	1.332	0.978
P35251	Replication factor C subunit 1 OS=Homo sapiens GN=RFC1 PE=1	1.374	2.089	1.810	1.773	1.330	1.352	1.576	1.948
H0YIA8	Protein lin-7 homolog A (Fragment) OS=Homo sapiens GN=LIN7/	1.226	1.423	1.226	1.293	1.116	1.248	1.164	1.337
Q9HB90	Ras-related GTP-binding protein C OS=Homo sapiens GN=RRAG(	1.948	1.755	1.572	1.346	1.931	1.278	1.706	2.121
Q1RLN5	ARHGAP12 protein OS=Homo sapiens GN=ARHGAP12 PE=2 SV=	1.478	1.098	1.306	0.962	1.477	1.064	1.263	0.965
A8K2B4	cDNA FLJ77490, highly similar to Homo sapiens enabled homolog	1.714	1.200	1.634	1.263	1.483	1.172	1.372	1.333
Q9H6E4	Coiled-coil domain-containing protein 134 OS=Homo sapiens GN=	1.585	1.605	1.409	1.434	1.204	1.267	1.412	1.542
B3KT25	cDNA FLJ37504 fis, clone BRAWH2017103, highly similar to ATPa	1.227	1.353	1.193	0.893	1.284	1.318	1.030	0.764
P17612	cAMP-dependent protein kinase catalytic subunit alpha OS=Homo	2.702	2.113	1.930	1.576	2.209	1.181	2.116	1.903
B4E0Y8	cDNA FLJ58970, highly similar to Homo sapiens SYF2 homolog, R	1.679	1.935	1.555	0.852	1.573	1.522	1.701	1.186

Q8TD16	Protein bicaudal D homolog 2 OS=Homo sapiens GN=BICD2 PE=	1.662	1.246	1.410	1.301	1.595	1.414	1.774	1.390
P22694	cAMP-dependent protein kinase catalytic subunit beta OS=Homo	1.712	1.431	1.658	1.110	1.341	1.218	1.818	1.137
P49711	Transcriptional repressor CTCF OS=Homo sapiens GN=CTCF PE=	3.070	2.309	2.019	1.869	1.317	1.345	1.687	1.532
J3KQQ5	Polypeptide N-acetylgalactosaminyltransferase 14 (Fragment) OS=	2.369	2.427	2.322	2.006	1.883	1.470	2.460	2.665
Q9H9C1	Spermatogenesis-defective protein 39 homolog OS=Homo sapien	1.363	1.042	1.112	0.893	1.053	0.979	1.053	0.930
Q9NTZ6	RNA-binding protein 12 OS=Homo sapiens GN=RBM12 PE=1 SV=	1.345	1.212	1.070	0.771	1.410	1.238	0.957	0.885
O00560	Syntenin-1 OS=Homo sapiens GN=SDCBP PE=1 SV=1 - [SDCB1_	0.557	0.466	0.804	0.702	0.655	0.866	0.417	0.367
Q9UDY4	DnaJ homolog subfamily B member 4 OS=Homo sapiens GN=DN.	1.698	1.027	1.519	1.113	1.279	1.337	1.412	1.058
P61962	DDB1- and CUL4-associated factor 7 OS=Homo sapiens GN=DCA	1.282	1.465	1.224	1.050	1.139	1.906	1.334	1.121
Q15750	TGF-beta-activated kinase 1 and MAP3K7-binding protein 1 OS=H	1.588	1.026	1.395	1.050	1.248	0.987	1.245	0.815
Q9UBF2	Coatomer subunit gamma-2 OS=Homo sapiens GN=COPG2 PE=1	1.397	0.998	1.320	1.048	1.277	1.061	1.321	1.158
H3BPE1	Microtubule-actin cross-linking factor 1, isoforms 1/2/3/5 OS=Hor	1.599	1.379	1.211	0.898	1.331	1.172	1.182	1.024
Q3B726	DNA-directed RNA polymerase I subunit RPA43 OS=Homo sapien	2.188	3.085	2.464	2.522	2.188	1.802	2.846	2.649
Q13564	NEDD8-activating enzyme E1 regulatory subunit OS=Homo sapier	1.262	0.900	1.147	0.786	1.113	1.023	1.080	0.775
H3BRU1	Protein FAM219B (Fragment) OS=Homo sapiens GN=FAM219B PI					0.927	0.924		
Q9NQ48	Leucine zipper transcription factor-like protein 1 OS=Homo sapier	1.178	0.978	1.158	1.105	0.851	0.970	1.593	0.678
B7Z1M0	cDNA FLJ50116, highly similar to Arylsulfatase E (EC 3.1.6.-) OS=	1.264	1.400	1.161	0.964	1.296	1.060	1.304	1.002
Q5SW79	Centrosomal protein of 170 kDa OS=Homo sapiens GN=CEP170 I	1.351	1.125	1.181	0.903	1.041	0.928	1.076	0.824
B3KRS1	cDNA FLJ34794 fis, clone NT2NE2005676, highly similar to Gamn	1.246	0.916	1.134	0.825	1.140	1.149	1.163	1.207
Q9H9E3	Conserved oligomeric Golgi complex subunit 4 OS=Homo sapiens	2.309	2.087	1.973	1.361	1.978	1.188	1.739	1.735
Q8NE01	Metal transporter CNNM3 OS=Homo sapiens GN=CNNM3 PE=1 S	1.195	1.654	1.612	1.434	1.141	1.530	1.649	1.150
B4E3V1	cDNA FLJ61209, highly similar to NG,NG-dimethylarginine dimeth	2.191	1.974	2.151	2.207	2.090	1.449	2.396	2.127
C9JA07	Cytochrome c oxidase assembly factor 1 homolog (Fragment) OS	0.480	0.619	0.501	0.594	0.589	1.045	0.704	0.564
Q8IXH7	Negative elongation factor C/D OS=Homo sapiens GN=NELFCD P	1.940	1.702	1.984	1.297	1.968	1.298	1.399	1.253
A0A024RCR6	HLA-B associated transcript 3, isoform CRA_a OS=Homo sapiens	1.584	1.123	1.152	0.936	1.480	1.183	1.255	0.980
Q6QWC0	TAP1 OS=Homo sapiens GN=TAP1 PE=2 SV=1 - [Q6QWC0_HUM	1.520	0.944	0.544	0.446	1.173	0.935	0.724	0.616
Q9UHY7	Enolase-phosphatase E1 OS=Homo sapiens GN=ENOPH1 PE=1 S	1.386	1.067	1.715	1.325	1.455	1.046	0.651	1.253
P53384	Cytosolic Fe-S cluster assembly factor NUBP1 OS=Homo sapiens	1.240	0.980	1.303	1.192	1.037	1.019	1.296	1.260
Q96KB3	cDNA FLJ14389 fis, clone HEMBA1002876 OS=Homo sapiens PE=	1.530	1.295	1.373	1.419	1.110	1.152	1.425	1.180
A0A024R3N3	Amyloid beta (A4)-like protein 2, isoform CRA_a OS=Homo sapier	1.407	1.861	1.228	0.928	1.152	1.645	1.631	1.074
Q9HAB8	Phosphopantothenate--cysteine ligase OS=Homo sapiens GN=PP	1.450	1.284	1.641	1.470	1.512	1.099	1.450	1.612
Q86YQ0	HZGJ OS=Homo sapiens GN=HZGJ PE=2 SV=1 - [Q86YQ0_HUM/	1.127	0.785	0.987	0.674	1.138	0.906	1.030	0.872
Q96FF9	Sororin OS=Homo sapiens GN=CDCA5 PE=1 SV=1 - [CDCA5_HU	1.536	2.095	1.724	1.609	1.441	1.429	2.359	1.919
A8K644	Splicing factor, arginine/serine-rich 4, isoform CRA_b OS=Homo s	2.177	2.132	1.973	1.488	2.095	1.466	1.970	2.020
M0QZ22	Protein Smaug homolog 2 OS=Homo sapiens GN=SAMD4B PE=1	2.048	1.893	1.991	1.485	2.028	1.323	2.102	1.610
P56937	3-keto-steroid reductase OS=Homo sapiens GN=HSD17B7 PE=1	1.353	1.314	1.166	1.133	1.016	1.162	1.073	1.106
Q9HAJ7	Histone deacetylase complex subunit SAP30L OS=Homo sapiens	1.122	1.883	1.724	1.049	1.084	1.153	1.333	1.704
Q5T440	Putative transferase CAF17, mitochondrial OS=Homo sapiens GN=	1.568	1.667	1.524	1.450	1.246	1.558	1.777	1.795
P48426	Phosphatidylinositol 5-phosphate 4-kinase type-2 alpha OS=Hom	2.195	1.062	1.660	1.244	1.765	1.211	1.952	1.797
Q96P16	Regulation of nuclear pre-mRNA domain-containing protein 1A OS	1.457	1.238	1.427	1.043	1.310	1.390	1.552	0.954

Q9BUE0	Mediator of RNA polymerase II transcription subunit 18 OS=Hom	1.791	1.697	1.416	1.204	1.938	1.420	1.704	1.507
P17275	Transcription factor jun-B OS=Homo sapiens GN=JUNB PE=1 SV=	1.146	1.077	0.474	0.526	1.160	1.056	0.805	0.593
P62314	Small nuclear ribonucleoprotein Sm D1 OS=Homo sapiens GN=SI	1.894	1.414	1.456	1.015	1.784	1.274	1.685	1.230
Q86TI2	Dipeptidyl peptidase 9 OS=Homo sapiens GN=DPP9 PE=1 SV=3	1.464	1.092	1.173	0.942	1.316	0.905	1.219	1.187
A0A024R5L7	Phosphatidylinositol binding clathrin assembly protein, isoform CF	1.346	1.546	1.000	0.906	1.372	1.170	1.307	1.096
Q14331	Protein FRG1 OS=Homo sapiens GN=FRG1 PE=1 SV=1 - [FRG1_	1.498	1.978	2.084	1.554	1.448	1.638	1.757	1.821
Q14641	Early placenta insulin-like peptide OS=Homo sapiens GN=INSL4 I	1.503	0.965	0.434		1.375	0.911	0.791	0.390
Q86TB9	Protein PAT1 homolog 1 OS=Homo sapiens GN=PATL1 PE=1 SV=	1.354	1.147	1.537	0.985	1.194	1.114	1.224	0.981
B7Z5X1	cDNA FLJ60843, highly similar to Serine/threonine-protein phospl	1.601	0.976	1.365	0.899	1.093	1.060	1.365	0.936
Q96HR9	Receptor expression-enhancing protein 6 OS=Homo sapiens GN=	1.185	1.127	1.224	1.046	1.068	1.439	1.411	1.327
A8K8Z3	cDNA FLJ77613, highly similar to Homo sapiens tousled-like kinas	1.169	1.156	1.429	1.301	1.318	1.356	1.388	1.967
Q9NXR7	BRCA1-A complex subunit BRE OS=Homo sapiens GN=BRE PE=1	1.868	1.831	1.544	1.084	1.645	1.175	1.795	1.418
Q9UM22	Mammalian ependymin-related protein 1 OS=Homo sapiens GN=	1.340	1.160	1.109	1.374	1.076	0.957	0.939	1.308
B2R602	cDNA, FLJ92709, highly similar to Homo sapiens LanC lantibiotic s	1.422	1.009	1.623	1.232	1.300	0.937	1.210	1.188
B4DDH8	cDNA FLJ55184, highly similar to Homo sapiens leukocyte recepto	1.492	1.277	1.036	1.118	1.024	1.167	1.005	1.042
D3DVH1	Succinate dehydrogenase complex, subunit C, integral membrane	9.381	10.262	9.152	8.483	5.243	2.316	7.987	8.927
Q9H0U3	Magnesium transporter protein 1 OS=Homo sapiens GN=MAGT1	1.455	1.378	1.577	1.378	1.178	1.220	1.375	1.445
O60828	Polyglutamine-binding protein 1 OS=Homo sapiens GN=PQBP1 P	1.116	1.160	0.954	0.720	1.213	1.263	1.034	0.962
H7C285	Tetraspanin-15 (Fragment) OS=Homo sapiens GN=TSPAN15 PE=	1.133	1.620	1.028	1.079	1.188	1.112	1.062	1.082
O94903	Proline synthase co-transcribed bacterial homolog protein OS=Ho	1.434	1.045	1.352	1.314	1.166	1.085	1.293	1.359
Q9NZN3	EH domain-containing protein 3 OS=Homo sapiens GN=EHD3 PE	0.667				0.513	0.720	0.616	0.516
P48730	Casein kinase I isoform delta OS=Homo sapiens GN=CSNK1D PE	1.561	1.476	0.977	0.621	1.092	1.101	1.247	1.040
A2RRP1	Neuroblastoma-amplified sequence OS=Homo sapiens GN=NBAS	1.437	1.478	1.474	1.446	1.266	1.330	1.576	1.569
O75794	Cell division cycle protein 123 homolog OS=Homo sapiens GN=CI	1.831	1.570	1.921	1.018	1.837	1.436	1.863	1.203
Q9Y4X5	E3 ubiquitin-protein ligase ARIH1 OS=Homo sapiens GN=ARIH1 I	1.542	1.161	1.400	1.055	1.315	1.267	1.369	1.284
Q53X93	CREB1 protein (Fragment) OS=Homo sapiens GN=CREB1 PE=2 S	4.084	2.595	1.914	1.879	1.531	1.046	2.194	2.487
E9PNK6	Tumor protein D53 OS=Homo sapiens GN=TPD52L1 PE=1 SV=1	0.992	0.780	0.902	0.933	1.199	0.944	1.241	1.021
O14497	AT-rich interactive domain-containing protein 1A OS=Homo sapie	1.226	1.100	0.752	0.609	1.264	1.145	1.024	0.960
A8K5C5	cDNA FLJ77534, highly similar to Homo sapiens membrane protei	1.378	1.344	1.089	0.885	1.252	1.219	1.043	1.245
Q9Y639	Neuroplastin OS=Homo sapiens GN=NPTN PE=1 SV=2 - [NPTN_	1.217	1.526	0.982	1.033	1.130	1.328	1.329	1.046
Q9H7D7	WD repeat-containing protein 26 OS=Homo sapiens GN=WDR26	1.585	1.391	1.142	0.862	1.470	1.316	1.244	1.126
Q9H944	Mediator of RNA polymerase II transcription subunit 20 OS=Hom	0.878	0.976	0.813	0.492	1.128	1.021	0.863	0.941
Q5C9Z4	Nucleolar MIF4G domain-containing protein 1 OS=Homo sapiens	1.574	1.774	1.362	1.183	1.438	1.538	1.494	1.213
A0A024R4D3	Melanophilin, isoform CRA_b OS=Homo sapiens GN=MLPH PE=4	1.599	1.287	0.590	0.356	1.511	1.400	0.914	0.540
Q9C0C9	E2/E3 hybrid ubiquitin-protein ligase UBE2O OS=Homo sapiens G	1.570	1.195	1.739	1.383	1.326	1.201	1.548	1.535
Q499Z2	B3GALT6 protein (Fragment) OS=Homo sapiens GN=B3GALT6 PE	1.346	1.685	1.428	1.519	1.134	1.563	1.579	1.354
B4DYP7	cDNA FLJ55435, highly similar to Gamma-tubulin complex compo	1.774	1.274	1.477	1.289	1.622	1.174	1.566	1.288
Q9BV86	N-terminal Xaa-Pro-Lys N-methyltransferase 1 OS=Homo sapiens	2.630	1.862	2.251	1.957	2.047	1.116	2.040	1.859
Q0P607	Splicing factor, arginine/serine-rich 15 OS=Homo sapiens GN=SFI	1.491	2.063	1.449	1.379	1.487	1.413	1.814	1.502
H0Y9V7	Calcium-transporting ATPase type 2C member 1 (Fragment) OS=I	1.543	1.829	1.540	1.369	1.549	1.373	1.694	1.496

Q53GL1	Nicotinamide nucleotide adenylyltransferase 1 variant (Fragment)	1.244	1.371	1.118	0.662	1.340	0.973	1.404	1.333
H0YNP1	D-glucuronyl C5-epimerase OS=Homo sapiens GN=GLCE PE=4 S	0.877	1.080	0.895	0.942	0.959	1.144	0.987	0.905
K7ER46	Beclin-1 (Fragment) OS=Homo sapiens GN=BECN1 PE=4 SV=1 -	0.534	0.441	0.524	0.453	0.889	1.130	0.673	0.503
Q14160	Protein scribble homolog OS=Homo sapiens GN=SCRIB PE=1 SV	1.049	1.551	1.209	1.267	1.117	1.138	1.142	1.233
A8K9X5	cDNA FLJ76472, highly similar to Homo sapiens Fas (TNFRSF6) a	1.235	1.157	1.178	1.058	1.390	1.172	1.382	1.193
A8K6Q4	cDNA FLJ76888, highly similar to Homo sapiens RNA binding mot	1.525	1.712	1.650	1.307	1.487	1.448	1.499	1.413
Q6PD74	Alpha- and gamma-adaptin-binding protein p34 OS=Homo sapier	1.472	0.893	1.368	1.346	1.293	0.915	1.321	1.190
A8K7A1	cDNA FLJ77346, highly similar to Homo sapiens DEAD (Asp-Glu-A	1.881	2.108	2.046	1.626	1.389	1.607	1.565	1.288
B7Z4M5	cDNA FLJ56114, highly similar to Rap1 GTPase-GDP dissociation s	1.718	1.081	1.603	1.298	1.392	1.099	1.530	1.622
H7C4K8	Very-long-chain (3R)-3-hydroxyacyl-CoA dehydratase 2 (Fragmen	5.124	4.724	4.950	4.231	2.995	1.534	4.528	3.557
Q643R3	Lysophospholipid acyltransferase LPCAT4 OS=Homo sapiens GN=	1.111	0.919	0.882	0.762	0.890	0.914	1.100	0.961
I3L4G8	Charged multivesicular body protein 6 (Fragment) OS=Homo sap	2.540	2.859	2.510	2.263	1.976	1.680	2.286	2.617
B4DP84	DCN1-like protein OS=Homo sapiens PE=2 SV=1 - [B4DP84_HUM	2.859	2.227	2.393	1.714	2.013	1.374		2.227
Q9BRP4	Proteasomal ATPase-associated factor 1 OS=Homo sapiens GN=F	1.189	0.737	1.113	0.774	1.197	0.797	1.074	0.851
Q96FK6	WD repeat-containing protein 89 OS=Homo sapiens GN=WDR89	2.132	2.226	2.043	2.047	1.923	1.659	2.168	2.766
P30154	Serine/threonine-protein phosphatase 2A 65 kDa regulatory subu	2.196	1.419	1.525	0.997	1.904	1.206	1.855	1.253
B2R8E4	cDNA, FLJ93859, highly similar to Homo sapiens protein phospha	1.515	0.972	1.378	0.938	1.238	0.981	1.149	1.047
I3L1R7	Ethanolamine-phosphate cytidylyltransferase OS=Homo sapiens (	2.503	1.153	2.225	1.357	1.739	1.455	1.578	1.789
Q7L5A8	Fatty acid 2-hydroxylase OS=Homo sapiens GN=FA2H PE=1 SV=	2.428	2.003	1.219	1.044	1.647	1.286	1.748	1.288
A0A087WUW9	ADP-ribosylation factor-like protein 15 OS=Homo sapiens GN=AR	1.120	1.635	1.235	1.491	1.303	1.634	1.784	1.803
P62891	60S ribosomal protein L39 OS=Homo sapiens GN=RPL39 PE=1 S	1.213	1.101	1.193	1.100	1.178	1.115	1.164	1.068
Q9BQC6	Ribosomal protein 63, mitochondrial OS=Homo sapiens GN=MRP	1.483	1.702	1.328	1.235	1.155	1.169	1.444	1.397
B2R5H5	cDNA, FLJ92476, highly similar to Homo sapiens LSM3 homolog,	1.107	1.025	0.891	0.756	1.101	1.094	1.016	0.876
P17535	Transcription factor jun-D OS=Homo sapiens GN=JUND PE=1 SV	1.080	1.086	0.651	0.437	1.317	1.002	0.796	0.827
Q9NPE2	Neugrin OS=Homo sapiens GN=NGRN PE=1 SV=2 - [NGRN_HUM	2.486	2.422	1.920	1.632	1.518	1.223	1.808	1.527
Q6PI78	Transmembrane protein 65 OS=Homo sapiens GN=TMEM65 PE=	1.820	1.869	1.366	1.504	1.271	1.048	1.521	1.402
Q8NBX0	Saccharopine dehydrogenase-like oxidoreductase OS=Homo sapi	1.263	1.441	1.254	1.115	0.816	1.103	1.223	1.446
O95071	E3 ubiquitin-protein ligase UBR5 OS=Homo sapiens GN=UBR5 PE	1.273	1.112	0.831	0.699	1.248	1.064	0.939	0.729
Q15555	Microtubule-associated protein RP/EB family member 2 OS=Hom	1.258	0.910	1.351	0.586	1.135	0.963	0.971	0.923
P14324	Farnesyl pyrophosphate synthase OS=Homo sapiens GN=FDPS P	1.218	0.855	1.168	1.073	1.119	0.970	1.170	1.030
B2R5W6	cDNA, FLJ92661, highly similar to Homo sapiens microtubule-ass	2.009	1.648	1.807	1.186	1.289	1.270	1.641	1.231
Q8N543	Prolyl 3-hydroxylase OGFOD1 OS=Homo sapiens GN=OGFOD1 PE	1.303	1.266	1.552	1.157	1.229	1.334	1.192	1.285
Q8NEC6	MTMR1 protein OS=Homo sapiens GN=MTMR1 PE=1 SV=1 - [Q8	1.630	1.057	1.598	1.380	1.204	1.184	1.630	1.417
Q9H6D7	HAUS augmin-like complex subunit 4 OS=Homo sapiens GN=HAU	1.726	1.419	1.773	1.532	1.466	1.229	1.737	1.800
P78549	Endonuclease III-like protein 1 OS=Homo sapiens GN=NTHL1 PE	1.131	1.018	0.890	0.758	1.001	0.965	0.998	0.790
Q96KC2	ADP-ribosylation factor-like protein 5B OS=Homo sapiens GN=AR	2.708	2.304	1.488	1.539	2.181	1.535	2.049	1.586
Q9NSK0	Kinesin light chain 4 OS=Homo sapiens GN=KLC4 PE=1 SV=3 - [	1.758	1.151	1.503	1.229	1.211	0.830	1.140	1.248
P27361	Mitogen-activated protein kinase 3 OS=Homo sapiens GN=MAPK3	1.298	0.866	1.154	0.991	1.088	0.720	1.057	0.896
A0A024RAB0	Endothelin converting enzyme 1, isoform CRA_a OS=Homo sapie	1.284	1.217	0.926	0.817	1.157	1.240	1.127	0.911
Q8NHP8	Putative phospholipase B-like 2 OS=Homo sapiens GN=PLBD2 PE	1.442	1.051	1.266	1.126	1.283	0.944	1.222	1.143

Q9H974	Queuine tRNA-ribosyltransferase subunit QTRTD1 OS=Homo sapi	1.549	1.215	1.562	1.135	1.463	1.112	1.278	1.167
Q9NXN4	Ganglioside-induced differentiation-associated protein 2 OS=Hom	1.444	1.875	1.500	1.170	2.028	1.490	1.909	1.470
A8K6Q9	cDNA FLJ75882, highly similar to Homo sapiens spastic paraplegi	1.274	0.989	1.471	1.209	1.193	1.163	1.257	1.211
B3KPK9	cDNA FLJ31905 fis, clone NT2RP7004358, highly similar to Homo	1.212	0.944	1.368	1.193	1.066	1.185	1.185	1.056
P23378	Glycine dehydrogenase (decarboxylating), mitochondrial OS=Hon	1.899	1.877	1.610	1.509	1.451	1.303	1.789	1.667
P85037	Forkhead box protein K1 OS=Homo sapiens GN=FOXK1 PE=1 SV	1.292	1.167	1.194	0.914	1.123	1.020	1.125	0.859
Q9NVT9	Armadillo repeat-containing protein 1 OS=Homo sapiens GN=ARI	2.138	2.006	1.923	1.747	2.099	1.282	2.085	2.407
B4DYK6	cDNA FLJ56887, highly similar to Homo sapiens guanine nucleotic	1.235	0.975	1.600	1.710	1.400	0.964	1.635	1.332
Q96EU7	C1GALT1-specific chaperone 1 OS=Homo sapiens GN=C1GALT1C	2.077	2.316	1.578	1.751	1.782	1.514	2.610	1.912
Q6UWE0	E3 ubiquitin-protein ligase LRSAM1 OS=Homo sapiens GN=LRSAI	2.212	1.463	1.669	1.435	1.611	0.909	1.512	1.299
Q86T03	Type 1 phosphatidylinositol 4,5-bisphosphate 4-phosphatase OS=	1.623	1.733	1.480	1.018	1.989	1.449	1.899	1.545
I3VM54	N-terminus deleted lysine-specific demethylase 2A OS=Homo sap	1.392	1.714	1.428	1.420	1.392	1.347	1.430	1.276
P50238	Cysteine-rich protein 1 OS=Homo sapiens GN=CRIP1 PE=1 SV=3	1.216	0.816	1.342	1.251	1.083	1.022	1.078	1.145
Q53GP2	Thioredoxin-like 4B variant (Fragment) OS=Homo sapiens PE=2 S	1.136	1.061	1.193	0.727	1.118	1.485	1.230	1.169
B3KRJ9	cDNA FLJ34439 fis, clone HLUNG2001146, highly similar to Splicii	1.640	1.756	1.367	1.179	1.676	1.267	1.471	1.366
A8K6K2	cDNA FLJ75877, highly similar to Homo sapiens 5'-nucleotidase, c	1.543	0.979	1.411	1.158	1.448	0.955	1.274	1.084
Q93008	Probable ubiquitin carboxyl-terminal hydrolase FAF-X OS=Homo s	1.735	0.982	1.464	1.096	1.527	1.102	1.244	1.172
Q5TE63	BCL2-like 1, isoform CRA_c OS=Homo sapiens GN=BCL2L1 PE=2	1.031	1.111	0.853	0.790	1.158	1.147	0.905	0.891
E7EQI7	WASH complex subunit strumpellin OS=Homo sapiens GN=KIAA(	1.402	1.058	1.339	1.049	1.224	1.186	1.395	1.126
Q8N335	Glycerol-3-phosphate dehydrogenase 1-like protein OS=Homo sa	2.594	2.621	3.267	3.055	2.131	1.220	2.737	2.982
D3DQS4	Formin binding protein 4, isoform CRA_d OS=Homo sapiens GN=	1.198	1.226	1.097	0.924	1.167	1.168	1.182	1.158
Q9UKJ3	G patch domain-containing protein 8 OS=Homo sapiens GN=GPA	1.637	1.430	1.364	1.012	1.505	1.445	1.368	0.948
D3DS96	Bromodomain adjacent to zinc finger domain, 1A, isoform CRA_c	1.294	1.684	1.302	1.152	1.111	1.434	1.520	1.478
Q9BV44	THUMP domain-containing protein 3 OS=Homo sapiens GN=THU	1.407	0.949	1.373	1.067	1.229	1.114	1.383	1.228
Q9Y2X7	ARF GTPase-activating protein GIT1 OS=Homo sapiens GN=GIT1	1.139	0.833	1.107	0.934	0.958	1.035	1.159	0.918
P10398	Serine/threonine-protein kinase A-Raf OS=Homo sapiens GN=AR	1.147	0.904	1.204	0.880	1.127	1.027	1.174	1.045
Q15910	Histone-lysine N-methyltransferase EZH2 OS=Homo sapiens GN=	1.640	1.780	1.325	1.217	1.728	1.720	1.595	1.539
B7ZKY2	Calcium/calmodulin-dependent serine protein kinase (MAGUK fan	1.008	1.474	1.177	1.050	1.077	1.168	1.076	1.035
B4DTN0	cDNA FLJ51085, highly similar to Retinoblastoma-associated prot	2.074	1.471	1.187	0.689	1.607	1.009	0.984	0.616
A0A024R1M8	Apolipoprotein L, 2, isoform CRA_a OS=Homo sapiens GN=APOL	1.403	1.403	1.105	1.012	1.252	1.344	1.259	1.169
O00443	Phosphatidylinositol 4-phosphate 3-kinase C2 domain-containing	1.486	1.417	1.411	1.283	1.288	1.152	1.363	1.194
A8MTY9	Down syndrome critical region protein 3 OS=Homo sapiens GN=I	1.142			0.778	1.054	0.902	0.854	
P52756	RNA-binding protein 5 OS=Homo sapiens GN=RBM5 PE=1 SV=2	1.638	1.985	1.771	1.191	1.689	1.778	2.070	1.648
H0Y9E6	Centromere protein H (Fragment) OS=Homo sapiens GN=CENPH	1.227	1.294	1.081	1.254	1.342	1.269	1.482	1.424
Q16514	Transcription initiation factor TFIID subunit 12 OS=Homo sapiens	1.067	1.293	1.118	0.972	1.245	1.336	1.328	1.251
B3KP31	cDNA FLJ31047 fis, clone HSYRA2000424, highly similar to Carbo	1.396	0.893	1.185	1.257	1.279	1.042	1.289	1.306
Q13888	General transcription factor IIH subunit 2 OS=Homo sapiens GN=	1.431	1.644	1.316	1.269	1.492	1.273	1.631	1.692
E9PQY3	Lysosomal acid phosphatase OS=Homo sapiens GN=ACP2 PE=4 S	1.587	1.602	1.515	1.585	1.712	1.215	1.456	1.381
R4GN18	Membrane cofactor protein (Fragment) OS=Homo sapiens GN=CI	0.452	0.668	0.538	0.518	0.752	1.385	0.958	0.573
Q01664	Transcription factor AP-4 OS=Homo sapiens GN=TFAP4 PE=1 SV	1.444	1.960	1.838	1.434	1.353	1.385	1.176	1.947

Q92791	Synaptonemal complex protein SC65 OS=Homo sapiens GN=LEPI	0.997	0.794	0.953	0.842	0.785	0.998	1.033	0.879
P18583	Protein SON OS=Homo sapiens GN=SON PE=1 SV=4 - [SON_HU	1.527	1.699	1.390	1.247	1.499	1.508	1.598	1.626
Q969E2	Secretory carrier-associated membrane protein 4 OS=Homo sapie	1.272	1.516	1.123	1.409	1.063	1.206	1.346	1.271
Q96ER3	Protein SAAL1 OS=Homo sapiens GN=SAAL1 PE=1 SV=2 - [SAAL	2.500	1.895	2.256	1.692	1.779	1.545	2.186	1.818
Q9ULX6	A-kinase anchor protein 8-like OS=Homo sapiens GN=AKAP8L PE	1.290	1.613	1.192	0.797	1.474	1.351	1.498	0.989
Q9HD45	Transmembrane 9 superfamily member 3 OS=Homo sapiens GN=	3.388	3.199	2.644	2.841	2.632	1.831	3.524	3.227
Q9H3Z4	DnaJ homolog subfamily C member 5 OS=Homo sapiens GN=DN	6.501	6.675	5.005	5.517	3.936	2.110	5.291	5.490
Q15651	High mobility group nucleosome-binding domain-containing prote	1.290	1.626	1.429	1.219	0.998	1.087	1.232	1.240
M0QYH2	Bifunctional polynucleotide phosphatase/kinase OS=Homo sapien	1.591	1.635	1.559	1.387	1.319	1.287	1.371	1.558
A7E2Y5	DnaJ (Hsp40) homolog, subfamily C, member 13 OS=Homo sapie	1.451	1.254	1.228	1.071	1.224	1.110	1.242	1.298
Q8WXD5	Gem-associated protein 6 OS=Homo sapiens GN=GEMIN6 PE=1					0.659	1.426	0.828	0.358
E7EV10	Metastasis-associated protein MTA3 OS=Homo sapiens GN=MTA3	1.613	1.638	1.244	1.170	1.610	1.294	1.649	1.405
A8K8X0	cDNA FLJ75187, highly similar to Homo sapiens nap1 P120 OS=H	1.871	1.402	1.630	1.143	1.518	1.268	1.533	1.202
Q96IR7	4-hydroxyphenylpyruvate dioxygenase-like protein OS=Homo sap	1.109	1.331	1.451	1.513	0.964	1.370	1.453	1.349
P21266	Glutathione S-transferase Mu 3 OS=Homo sapiens GN=GSTM3 PE	2.335	2.137	3.195	3.376	1.790	1.730	2.612	2.891
A3KMH1	von Willebrand factor A domain-containing protein 8 OS=Homo s	1.270	1.444	1.113	1.131	1.039	1.226	1.253	1.246
B2R4D5	Actin-related protein 2/3 complex subunit 3 OS=Homo sapiens PE	1.598	1.078	0.827	0.638	0.848	1.078	0.823	0.855
P34913	Bifunctional epoxide hydrolase 2 OS=Homo sapiens GN=EPHX2 P	1.611	1.386	1.818	1.514	1.406	1.280	1.489	1.641
Q8N5C6	S1 RNA-binding domain-containing protein 1 OS=Homo sapiens G	1.221	1.766	1.229	1.386	1.126	1.236	1.262	1.155
Q6NUM7	AGPAT5 protein (Fragment) OS=Homo sapiens GN=AGPAT5 PE=2	1.597	1.717	1.533	1.515	1.291	1.308	1.727	2.012
D3DSS6	Dedicator of cytokinesis 5, isoform CRA_a OS=Homo sapiens GN=	1.359	1.100	1.148	0.825	1.134	0.961	1.023	0.827
Q96T23	Remodeling and spacing factor 1 OS=Homo sapiens GN=RSF1 PE	1.702	1.620	1.194	0.991	1.616	1.333	1.657	1.227
O43823	A-kinase anchor protein 8 OS=Homo sapiens GN=AKAP8 PE=1 SV	1.385	1.480	1.129	0.912	1.440	1.451	1.417	1.109
B2RMQ4	Cytoskeleton associated protein 2 OS=Homo sapiens GN=CKAP2	1.429	2.096	1.767	1.416	1.042	1.694	1.992	2.353
A0A024R6R1	SHC SH2-domain binding protein 1, isoform CRA_a OS=Homo sap	1.673	1.592	1.325	0.938	1.779	1.708	1.853	1.554
D3DVW9	Protein tyrosine phosphatase, non-receptor type substrate 1, isof	0.972	1.428	1.068	1.041	0.954	1.354	1.561	1.607
Q6P6C2	RNA demethylase ALKBH5 OS=Homo sapiens GN=ALKBH5 PE=1	1.228	1.352	1.263	0.943	1.392	1.197	1.419	1.014
Q8WUX2	Cation transport regulator-like protein 2 OS=Homo sapiens GN=C	1.441	1.145	1.822	1.374	1.187	1.199	1.549	1.746
E7EWE1	Ubiquitin-like modifier-activating enzyme 5 OS=Homo sapiens GN	0.826	0.661	1.017	0.993	0.957	0.916	1.192	1.113
B3KP47	cDNA FLJ31151 fis, clone IMR322001541, highly similar to Probal	2.089	2.159	1.642	1.217	2.099	1.677	1.854	1.388
D6R9U7	DNA-directed RNA polymerase III subunit RPC7 (Fragment) OS=H	1.816	1.981	1.684	1.282	1.580	1.583	1.633	1.847
O15014	Zinc finger protein 609 OS=Homo sapiens GN=ZNF609 PE=1 SV=	0.984	1.046	0.682	0.618	0.947	1.115	1.016	0.989
Q9BV38	WD repeat-containing protein 18 OS=Homo sapiens GN=WDR18	1.455	1.741	1.235	1.099	1.493	1.328	1.397	1.110
B2R6H7	cDNA, FLJ92955, highly similar to Homo sapiens transportin-SR (T	2.531	1.468	1.883	1.471	2.339	1.534	2.217	1.913
B3KM57	cDNA FLJ10347 fis, clone NT2RM2001035, highly similar to CCR4	1.872	2.005	1.621	1.297	1.642	1.285	1.602	1.240
A0A024RA66	Cell division cycle 2-like 5 (Cholinesterase-related cell division con	1.521	1.370	1.353	0.820	1.607	1.563	1.717	1.316
Q659A9	Putative uncharacterized protein DKFZp547I0910 (Fragment) OS=	1.697	1.705	1.240	1.335	1.320	1.060	1.442	1.197
P16278	Beta-galactosidase OS=Homo sapiens GN=GLB1 PE=1 SV=2 - [B	1.748	1.610	1.980	2.076	1.665	1.206	1.813	2.011
A7E2F7	CAP-GLY domain containing linker protein 2 OS=Homo sapiens GI	1.338	0.773	0.944	0.720	1.026	1.110	0.895	0.791
L0R804	Alternative protein AAK1 OS=Homo sapiens GN=AAK1 PE=4 SV=	0.982	0.734	0.970	0.760	0.944	0.962	1.111	0.715

O95822	Malonyl-CoA decarboxylase, mitochondrial OS=Homo sapiens GN	1.491	1.482	1.737	1.585	1.110	1.204	1.475	1.646
Q8WVV4	Protein POF1B OS=Homo sapiens GN=POF1B PE=1 SV=3 - [POF	1.082	0.984	1.139	0.842	1.183	0.902	1.074	1.013
Q02040	A-kinase anchor protein 17A OS=Homo sapiens GN=AKAP17A PE	1.548	1.594	1.196	1.095	1.388	1.348	1.356	1.370
B7Z588	cDNA FLJ52718, highly similar to Homo sapiens tumor necrosis fa	1.259	1.160	0.856	0.445	1.337	1.387	1.300	1.071
Q5JSH3	WD repeat-containing protein 44 OS=Homo sapiens GN=WDR44	1.666	1.059	1.452	1.241	1.591	1.075	1.438	1.383
A0A024QZH6	Serine arginine-rich pre-mRNA splicing factor SR-A1, isoform CRA	1.528	1.817	1.129	1.313	1.390	1.276	1.547	1.362
I3L2J8	Centrosomal protein of 131 kDa OS=Homo sapiens GN=CEP131 I	1.380	1.695	1.482	1.393	0.992	1.315	1.532	1.434
Q8NHZ8	Anaphase-promoting complex subunit CDC26 OS=Homo sapiens	1.368	1.359	0.939	0.742	1.294	1.357	1.286	0.967
Q7Z6E9	E3 ubiquitin-protein ligase RBBP6 OS=Homo sapiens GN=RBBP6	1.355	1.607	1.331	1.103	1.348	1.401	1.609	1.487
Q2PZI1	Probable C-mannosyltransferase DPY19L1 OS=Homo sapiens GN=	1.259	1.736	1.103	0.982	1.166	1.337	1.286	0.947
Q05519	Serine/arginine-rich splicing factor 11 OS=Homo sapiens GN=SRS	2.052	1.784	1.589	1.128	1.516	1.365	1.609	1.212
Q8TDX6	Chondroitin sulfate N-acetylgalactosaminyltransferase 1 OS=Hom	1.456	1.316	1.073	0.934	1.315	1.062	1.125	0.915
A0A024R4Z4	HCG2039447, isoform CRA_d OS=Homo sapiens GN=hCG_20394	1.549	1.383	1.273	1.076	1.325	1.292	1.424	1.312
O75648	Mitochondrial tRNA-specific 2-thiouridylase 1 OS=Homo sapiens (	1.986	1.870	1.954	1.857	1.283	1.414	1.910	2.202
Q8TBX8	Phosphatidylinositol 5-phosphate 4-kinase type-2 gamma OS=Ho	1.886	1.423	1.615	1.438	1.491	1.141	1.841	1.831
C9JDZ2	Fas apoptotic inhibitory molecule 1 (Fragment) OS=Homo sapiens	0.533	0.538	0.441	0.601		0.902	0.749	0.741
A0A024R4R9	Leukocyte receptor cluster (LRC) member 8, isoform CRA_a OS=I	1.264	1.301	1.031	0.925	1.240	1.179	1.113	1.003
A8K603	cDNA FLJ75870, highly similar to Homo sapiens neuro-oncologica	1.103	0.911	0.967	0.659	1.196	1.419	0.922	0.754
Q9NRL3	Striatin-4 OS=Homo sapiens GN=STRN4 PE=1 SV=2 - [STRN4_H	1.368	1.072	1.099	0.888	1.263	1.165	1.209	1.034
Q9BTY7	Protein HGH1 homolog OS=Homo sapiens GN=HGH1 PE=1 SV=1	1.553	1.329	1.643	1.303	1.425	1.198	1.674	1.372
Q8N9Z2	Coiled-coil domain-containing protein 71L OS=Homo sapiens GN=	0.877	0.995	1.043	0.935	0.886	1.212	1.162	1.434
Q96CS2	HAUS augmin-like complex subunit 1 OS=Homo sapiens GN=HAU	1.309	1.337	1.389	1.572	1.477	1.164	1.360	1.340
P14635	G2/mitotic-specific cyclin-B1 OS=Homo sapiens GN=CCNB1 PE=1	1.352	1.632	2.307	1.965	1.047	1.347	2.406	2.701
Q8TAA9	Vang-like protein 1 OS=Homo sapiens GN=VANGL1 PE=1 SV=1 -	1.405	1.581	1.638	1.358	1.467	1.317	1.518	1.287
A0A024QZR3	HCG2002731, isoform CRA_a OS=Homo sapiens GN=hCG_20027	1.695	1.357	1.414	1.141	1.571	1.310	1.234	1.011
B4DYU9	cDNA FLJ60060, highly similar to Origin recognition complex subu	1.106	1.213	1.039	0.941	1.140	1.126	1.100	1.209
B4E1V6	cDNA FLJ54235, highly similar to Ceroid-lipofuscinosis neuronal p	1.625	1.594	1.784	1.418	1.738	1.338	1.668	1.749
Q9UBV8	Peflin OS=Homo sapiens GN=PEF1 PE=1 SV=1 - [PEF1_HUMAN]	1.448	1.388	1.527	1.574	1.424	1.356	1.560	1.460
Q92974	Rho guanine nucleotide exchange factor 2 OS=Homo sapiens GN	1.382	1.064	1.205	0.760	1.133	1.015	1.146	0.819
O60885	Bromodomain-containing protein 4 OS=Homo sapiens GN=BRD4	2.003	1.985	1.741	1.699	1.622	1.188	1.902	1.796
Q59F45	Mothers against decapentaplegic homolog (Fragment) OS=Homo	1.414	1.030	0.936	0.652	1.222	0.856	0.757	0.631
P26572	Alpha-1,3-mannosyl-glycoprotein 2-beta-N-acetylglucosaminyltrar	1.664	1.957	1.711	1.749	1.667	1.460	1.854	1.800
P17900	Ganglioside GM2 activator OS=Homo sapiens GN=GM2A PE=1 SV	1.994	1.622	1.602	1.714	1.539	1.044	1.823	1.870
B7Z7F5	cDNA FLJ51581 OS=Homo sapiens PE=2 SV=1 - [B7Z7F5_HUMA	1.547	1.762	1.258	1.519	1.443	1.571	1.736	1.540
Q2NKX8	DNA excision repair protein ERCC-6-like OS=Homo sapiens GN=E	1.294	0.938	1.325	1.010	1.278	1.084	1.287	1.166
F5GX09	Protein FAM76B OS=Homo sapiens GN=FAM76B PE=1 SV=1 - [F!	1.430	1.441	1.302	1.071	1.651	1.323	1.451	1.316
Q9NWT6	Hypoxia-inducible factor 1-alpha inhibitor OS=Homo sapiens GN=	1.077	0.946	1.212	0.898	1.193	1.159	1.219	0.962
B4DWT6	cDNA FLJ51703, highly similar to Ubiquitin-conjugating enzyme E	1.363	1.104	1.248	0.925	1.171	1.094	1.162	1.023
Q9BUR4	Telomerase Cajal body protein 1 OS=Homo sapiens GN=WRAP53	1.375	1.233	1.334	1.090	1.523	1.113	1.319	1.215
A8K724	cDNA FLJ76899, highly similar to Homo sapiens p21(CDKN1A)-ac	1.075	0.831	0.950	0.593	0.867	0.953	0.909	0.611

H3BSE3	Phosphopantothenoylcysteine decarboxylase OS=Homo sapiens (	0.644	0.520	0.604	1.010	1.013	0.893	1.106	0.916
P62306	Small nuclear ribonucleoprotein F OS=Homo sapiens GN=SNRPF		0.530	0.305	0.280	0.702	1.369	0.513	0.445
Q8IY17	Neuropathy target esterase OS=Homo sapiens GN=PNPLA6 PE=1	1.496	1.276	1.299	1.148	1.270	1.198	1.287	1.089
Q9BRH2	ASRGL1 protein (Fragment) OS=Homo sapiens GN=ASRGL1 PE=2	0.980	0.761	1.210	1.080	0.881	1.045	1.007	1.327
Q9NX08	COMM domain-containing protein 8 OS=Homo sapiens GN=COMM	1.363	1.082	1.264	1.056	1.258	1.169	1.261	1.288
Q9UJX5	Anaphase-promoting complex subunit 4 OS=Homo sapiens GN=A	1.687	1.350	1.175	0.942	1.450	1.346	1.349	1.355
Q68D10	Protein SPT2 homolog OS=Homo sapiens GN=SPTY2D1 PE=1 SV	1.677	2.778	1.683	1.314	1.289	1.542	1.468	1.524
Q9H2G0	CTCL tumor antigen se37-2 OS=Homo sapiens GN=UBE3A PE=2	1.365	1.039	1.138	0.890	1.331	1.196	1.200	1.049
P20248	Cyclin-A2 OS=Homo sapiens GN=CCNA2 PE=1 SV=2 - [CCNA2_H	1.264	2.188	1.342	1.367	1.508	1.873	2.762	2.053
Q9UKV8	Protein argonaute-2 OS=Homo sapiens GN=AGO2 PE=1 SV=3 - [	1.450	1.533	1.351	0.962	1.370	1.299	1.346	1.243
B4DRF3	cDNA FLJ53020, highly similar to Homo sapiens timeless-interacti	1.132	1.820	1.118	2.615	1.388	1.742	1.180	1.114
B4DXH2	cDNA FLJ51138, highly similar to Arfaptin-2 OS=Homo sapiens PI	1.304	1.670	1.307	1.168	1.211	1.431	1.723	1.508
Q8IUH3	RNA-binding protein 45 OS=Homo sapiens GN=RBM45 PE=2 SV=	1.342	1.882	1.330	0.967	1.656	1.531	1.756	1.808
A0A087WUI6	Progesterone-induced-blocking factor 1 OS=Homo sapiens GN=P	1.588	1.956	1.897	1.641	1.622	1.240	1.854	1.784
O94808	Glutamine--fructose-6-phosphate aminotransferase [isomerizing]	2.218	0.990	1.467	0.897	1.809	0.922	1.423	1.212
Q7Z2K6	Endoplasmic reticulum metallopeptidase 1 OS=Homo sapiens GN	1.577	1.619	1.368	1.322	1.367	1.326	1.546	1.472
Q32P41	tRNA (guanine(37)-N1)-methyltransferase OS=Homo sapiens GN=	1.275	1.236	1.160	0.845	1.166	1.061	1.213	0.858
A9X3U0	BSK65-TEST2 OS=Homo sapiens GN=RNF185 PE=2 SV=1 - [A9X	2.411	2.509	2.346	2.117	1.496	1.523	2.231	2.057
B7Z7D5	cDNA FLJ53110 OS=Homo sapiens PE=2 SV=1 - [B7Z7D5_HUMA	1.012	0.927	1.022	0.780	0.970	0.966	0.989	0.750
O76024	Wolframin OS=Homo sapiens GN=WFS1 PE=1 SV=2 - [WFS1_HU	1.341	1.232	1.110	1.065	1.129	1.245	1.414	1.117
Q8TB40	Abhydrolase domain-containing protein 4 OS=Homo sapiens GN=	1.614	1.310	0.842	0.644	1.291	1.158	1.086	0.797
Q04941	Proteolipid protein 2 OS=Homo sapiens GN=PLP2 PE=1 SV=1 - [	0.989	1.180	0.992	1.084	1.104	1.220	0.957	1.046
H3BLV0	Complement decay-accelerating factor (Fragment) OS=Homo sap	1.476	1.361	0.817	0.984	1.144	0.882	0.983	0.793
A0A024RAJ5	Collagen, type IV, alpha 3 (Goodpasture antigen) binding protein,	1.705	1.401	1.756	1.480	1.611	1.122	1.541	1.400
Q13049	E3 ubiquitin-protein ligase TRIM32 OS=Homo sapiens GN=TRIM3	1.379	1.398	1.138	1.018	1.323	1.586	1.371	1.200
Q9UQ90	Paraplegin OS=Homo sapiens GN=SPG7 PE=1 SV=2 - [SPG7_HU	1.198	1.291	0.988	0.963	1.209	1.144	1.155	1.103
Q9Y6X4	Soluble lamin-associated protein of 75 kDa OS=Homo sapiens GN	1.668	1.625	1.505	1.303	1.581	1.587	1.757	1.534
Q96B36	Proline-rich AKT1 substrate 1 OS=Homo sapiens GN=AKT1S1 PE=	1.396	0.891	1.471	0.929	1.070	0.983	1.369	1.260
Q96EF6	F-box only protein 17 OS=Homo sapiens GN=FBXO17 PE=1 SV=	2.058	1.188	1.612	1.558	1.340	1.410	1.602	1.171
Q8TBP6	Solute carrier family 25 member 40 OS=Homo sapiens GN=SLC25	1.255	1.340	1.006	1.146	0.721	1.080	1.087	0.928
Q9NQW6	Actin-binding protein anillin OS=Homo sapiens GN=ANLN PE=1 S	1.508	1.578	1.052	0.795	1.401	1.249	1.601	1.268
B3KVH4	cDNA FLJ16549 fis, clone PLACE7003657, highly similar to RAC-al	1.336	0.846	1.222	0.871	1.043	1.123	1.174	1.096
Q6WKZ4	Rab11 family-interacting protein 1 OS=Homo sapiens GN=RAB11	1.324	1.712	1.228	1.268	1.021	1.258	1.321	1.459
P54289	Voltage-dependent calcium channel subunit alpha-2/delta-1 OS=I	1.253	1.520	1.292	1.388	1.068	1.197	1.267	0.994
Q7Z2Z2	Elongation factor Tu GTP-binding domain-containing protein 1 OS	1.580	1.166	1.519	1.097	1.350	1.044	1.357	1.183
E5RJS5	Uncharacterized protein OS=Homo sapiens GN=MTFR1 PE=1 SV=	1.178	1.244	1.042	0.994	0.971	1.196	1.237	1.156
B4DNZ2	cDNA FLJ57132, highly similar to Exostosin-like 2 (EC 2.4.1.223) (	1.617	1.806	1.460	1.748	1.458	1.565	1.741	1.802
Q9H9F9	Actin-related protein 5 OS=Homo sapiens GN=ACTR5 PE=1 SV=2	1.235	1.399	1.271	1.187	1.252	1.307	1.325	1.396
Q8NB88	cDNA FLJ34075 fis, clone FCBBF3003216 OS=Homo sapiens PE=	1.180	1.148	1.391	0.992	0.902	1.034	1.269	1.132
B0FTY2	NudC-like protein OS=Homo sapiens PE=2 SV=1 - [B0FTY2_HUM	1.116	0.922	1.466	1.023	0.975	1.030	1.244	1.169

Q96JC9	ELL-associated factor 1 OS=Homo sapiens GN=EAF1 PE=1 SV=1	1.051	1.200	0.811	0.616	0.925	1.252	0.967	0.879
Q9BRZ2	E3 ubiquitin-protein ligase TRIM56 OS=Homo sapiens GN=TRIM5	1.920	1.755	1.967	1.554	1.884	1.533	2.003	2.826
Q9BW85	Coiled-coil domain-containing protein 94 OS=Homo sapiens GN=(	1.656	1.592	1.419	1.008	1.367	1.378	1.428	1.054
P14174	Macrophage migration inhibitory factor OS=Homo sapiens GN=M	3.310	2.442	3.810	3.419	2.620	1.663	2.974	2.726
Q9NRF8	CTP synthase 2 OS=Homo sapiens GN=CTPS2 PE=1 SV=1 - [PYF	1.380	1.068	1.391	1.153	1.222	1.012	1.353	1.162
O95865	N(G),N(G)-dimethylarginine dimethylaminohydrolase 2 OS=Homo	1.095	0.699	0.985	0.861	1.046	0.767	0.835	0.863
H3BSW0	Leucine-rich repeat-containing protein 57 (Fragment) OS=Homo s	1.898	1.115	2.171	0.894	1.805	1.704	1.710	1.606
Q9UP83	Conserved oligomeric Golgi complex subunit 5 OS=Homo sapiens	1.259	1.391	1.177	0.982	1.214	1.235	1.287	1.166
Q9UNI6	Dual specificity protein phosphatase 12 OS=Homo sapiens GN=D	1.755	1.063	1.568	1.061	1.351	1.057	1.041	1.263
Q7L8P3	Putative uncharacterized protein DKFZp586I1023 (Fragment) OS=	0.921	0.932	0.898	0.861	1.087	1.109	1.065	0.946
Q8TC07	TBC1 domain family member 15 OS=Homo sapiens GN=TBC1D15	1.755	1.303	1.766	1.724	1.413	1.120	1.590	1.499
O75027	ATP-binding cassette sub-family B member 7, mitochondrial OS=I	1.427	1.662	1.386	1.280	1.127	1.089	1.474	1.315
P25445	Tumor necrosis factor receptor superfamily member 6 OS=Homo	1.302	0.667			1.379	1.538	1.992	2.438
P01137	Transforming growth factor beta-1 OS=Homo sapiens GN=TGFB1	1.810	1.257	1.280	1.001	1.522	1.060	1.560	1.435
E7EN32	Menin OS=Homo sapiens GN=MEN1 PE=1 SV=1 - [E7EN32_HUM	1.391	1.199	1.436	1.322	1.211	1.507	1.424	1.417
O75448	Mediator of RNA polymerase II transcription subunit 24 OS=Hom	1.434	1.533	1.111	0.757	1.455	1.370	1.381	1.051
A0A087WXM0	Forkhead box protein K2 OS=Homo sapiens GN=FOXK2 PE=4 SV	1.234	1.156	0.921	0.572	1.016	1.051	1.064	0.690
Q9NQS7	Inner centromere protein OS=Homo sapiens GN=INCENP PE=1 S	1.188	1.853	1.202	0.992	1.084	1.307	1.411	1.265
Q96MH2	Protein HEXIM2 OS=Homo sapiens GN=HEXIM2 PE=1 SV=1 - [H	2.073	1.809	1.658	0.828	2.163	1.055	1.944	1.209
M0R2P6	SH3KBP1 binding protein 1, isoform CRA_c OS=Homo sapiens GN	1.038	1.224	0.999	0.819	1.022	1.190	1.210	1.068
Q92783	Signal transducing adapter molecule 1 OS=Homo sapiens GN=ST	3.588	2.323	2.886	2.471	3.653	1.311	2.601	2.836
J3QL56	Protein SCO1 homolog, mitochondrial OS=Homo sapiens GN=SC(	1.277	1.327	1.227	1.223	0.982	1.209	1.327	1.346
Q8NHP6	Motile sperm domain-containing protein 2 OS=Homo sapiens GN=	2.112	1.837	1.767	1.648	1.656	1.298	1.961	1.590
K7ER15	Haloacid dehalogenase-like hydrolase domain-containing protein :	1.033	0.821	1.154	1.053	0.865	0.972	0.992	1.128
A0A024QYZ6	Exocyst complex component 3, isoform CRA_a OS=Homo sapiens	1.837	1.374	1.672	1.454	1.420	1.137	1.696	1.302
Q9NYV4	Cyclin-dependent kinase 12 OS=Homo sapiens GN=CDK12 PE=1	1.375	1.762	1.025	0.663	1.207	1.321	1.256	0.842
Q8IXI1	Mitochondrial Rho GTPase 2 OS=Homo sapiens GN=RHOT2 PE=1	1.778	1.418	1.253	1.941	1.095	1.503	1.745	1.817
G3V3Y1	Uncharacterized protein OS=Homo sapiens PE=4 SV=1 - [G3V3Y	1.450	1.002	1.734	1.564	1.288	0.950	1.329	1.330
P50749	Ras association domain-containing protein 2 OS=Homo sapiens G	1.276	0.923	1.358	0.955	1.121	0.964	1.185	1.048
Q7Z4Q2	HEAT repeat-containing protein 3 OS=Homo sapiens GN=HEATR3	1.578	1.413	1.863	1.027	1.270	1.282	1.413	1.296
B7ZKQ9	SCARB1 protein OS=Homo sapiens GN=SCARB1 PE=1 SV=1 - [B	1.708	1.928	1.485	1.276	1.679	1.141	1.774	1.523
Q96EM0	Trans-L-3-hydroxyproline dehydratase OS=Homo sapiens GN=L3H	1.150	0.759	0.846	0.682	0.937	0.955	0.883	0.763
A0A024R8Q1	Glucosidase, alpha acid (Pompe disease, glycogen storage diseas	1.185	1.179	1.280	1.403	0.838	1.153	1.136	1.372
Q5T4S7	E3 ubiquitin-protein ligase UBR4 OS=Homo sapiens GN=UBR4 PE	1.495	0.974	1.293	0.996	1.348	0.989	1.244	1.038
B7ZLI5	Family with sequence similarity 98, member C OS=Homo sapiens	1.323	0.593	1.162	0.994	1.171	1.335	1.198	0.781
Q9Y5A7	NEDD8 ultimate buster 1 OS=Homo sapiens GN=NUB1 PE=1 SV=	1.128	0.953	1.153	0.804	1.075	1.078	1.228	0.796
Q59F95	TBC1 domain family, member 13 variant (Fragment) OS=Homo sa	1.652	1.008	1.341	1.359	1.267	1.138	1.244	1.248
B4DUQ5	cDNA FLJ53610, highly similar to Mitochondrial folate transporter	1.169	1.213	0.896	0.755	1.050	0.960	1.276	0.910
A8K8B2	cDNA FLJ78127, highly similar to Homo sapiens SFRS protein kina	1.218	0.941	1.093	0.853	1.131	1.259	1.225	1.158
A8K5N3	Palmitoyltransferase OS=Homo sapiens PE=2 SV=1 - [A8K5N3_H	1.516	1.808	1.551	1.490	1.533	1.456	1.726	1.714

Q14139	Ubiquitin conjugation factor E4 A OS=Homo sapiens GN=UBE4A	1.608	1.238	1.375	0.981	1.571	1.466	1.480	1.264
Q86VX2	COMM domain-containing protein 7 OS=Homo sapiens GN=COMI	2.214	1.906	2.300	1.500	2.096	1.652	2.152	1.906
Q9NX47	E3 ubiquitin-protein ligase MARCH5 OS=Homo sapiens GN=MAR(	4.320	5.240	3.731	6.349	1.741	1.249	3.164	1.976
Q92696	Geranylgeranyl transferase type-2 subunit alpha OS=Homo sapie	1.707	1.341	1.791	1.475	1.264	1.222	1.816	1.727
Q06609	DNA repair protein RAD51 homolog 1 OS=Homo sapiens GN=RAI	1.259	1.226	1.242	0.846	1.137	1.492	1.501	1.160
C9JNM8	Transcription termination factor 1, mitochondrial (Fragment) OS=	1.180	1.437	0.946	0.754	1.031	1.419	1.057	0.924
B7Z6H4	DNA-directed RNA polymerase OS=Homo sapiens PE=2 SV=1 - [I	1.282	1.526	1.160	0.985	1.248	1.404	1.393	1.229
O00461	Golgi integral membrane protein 4 OS=Homo sapiens GN=GOLIM	1.212	1.567	1.336	1.423	1.096	1.454	1.570	1.393
Q96SY0	von Willebrand factor A domain-containing protein 9 OS=Homo s	1.672	1.764	1.290	1.196	1.625	1.572	1.359	1.406
S4R469	RNA-binding protein fox-1 homolog 2 (Fragment) OS=Homo sapi	1.186	1.126	0.812	0.490	1.284	1.031	0.937	0.812
P49006	MARCKS-related protein OS=Homo sapiens GN=MARCKSL1 PE=1	1.270	1.167	1.237	0.994	1.126	1.116	1.190	1.130
Q96EH3	Mitochondrial assembly of ribosomal large subunit protein 1 OS=I	1.402	1.400	1.055	0.990	1.048	1.269	1.313	1.158
Q6PRX3	Transducin-like enhancer of split 3 splice variant 1 OS=Homo sap	1.772	2.261	2.284	1.646	1.917	2.345	1.735	1.636
Q96GS4	Uncharacterized protein C17orf59 OS=Homo sapiens GN=C17orf!	1.120	1.066	1.004	1.042	1.076	1.119	0.938	0.947
Q8N490	Probable hydrolase PNKD OS=Homo sapiens GN=PNKD PE=1 SV	1.854	1.593	1.317	1.355	1.592	1.213	1.572	1.387
J3KNL5	GRAM domain-containing protein 1B OS=Homo sapiens GN=GRA	1.273	1.336	0.931	0.778	1.277	1.193	1.114	0.644
Q9P2R3	Rabankyrin-5 OS=Homo sapiens GN=ANKFY1 PE=1 SV=2 - [ANF	1.750	1.462	1.736	1.398	1.441	1.226	1.614	1.506
A0A087WYV3	Nucleolar protein of 40 kDa OS=Homo sapiens GN=ZCCHC17 PE=	1.972	2.397	1.518	1.496	1.773	1.370	1.628	1.630
P32121	Beta-arrestin-2 OS=Homo sapiens GN=ARRB2 PE=1 SV=2 - [ARI	3.109	2.285	3.380	2.343	2.132	1.571	2.500	2.244
B7Z2P6	cDNA FLJ55296, highly similar to Homo sapiens WD repeat doma	1.408	1.147	1.508	1.388	1.209	1.068	1.429	1.649
I3L0A0	HCG2044781 OS=Homo sapiens GN=TMEM189-UBE2V1 PE=4 SV	1.386	1.049	1.400	1.210	1.203	0.984	1.269	1.305
K7ENP1	tRNA (guanine(10)-N2)-methyltransferase homolog OS=Homo sa	1.349	0.932	1.164	1.135	1.067	1.021	1.234	1.375
Q96HY6	DDRGK domain-containing protein 1 OS=Homo sapiens GN=DDR	1.087	1.754	1.104	0.987	0.881	1.268	1.171	0.941
P10909	Clusterin OS=Homo sapiens GN=CLU PE=1 SV=1 - [CLUS_HUMA	1.836	1.355	0.823	0.617	1.591	1.198	0.995	0.739
Q9NWA0	Mediator of RNA polymerase II transcription subunit 9 OS=Homo	1.046	1.234	0.795	0.662	1.211	1.304	1.123	1.015
P49427	Ubiquitin-conjugating enzyme E2 R1 OS=Homo sapiens GN=CDC	1.341	1.113	1.700	1.354	1.259	1.288	1.677	1.428
Q9BTW9	Tubulin-specific chaperone D OS=Homo sapiens GN=TBCD PE=1	1.464	0.968	1.466	1.134	1.277	0.994	1.242	1.294
Q6PJG6	BRCA1-associated ATM activator 1 OS=Homo sapiens GN=BRAT1	1.485	1.120	1.581	1.084	1.366	1.282	1.528	1.429
Q9P2K3	REST corepressor 3 OS=Homo sapiens GN=RCOR3 PE=1 SV=2 -	1.314	1.085	0.790	0.681	1.482	1.345	1.119	0.931
Q8N9M1	Uncharacterized protein C19orf47 OS=Homo sapiens GN=C19orf4	1.158	1.693	0.885	0.445	1.037	1.299	1.278	0.749
Q6P1N0	Coiled-coil and C2 domain-containing protein 1A OS=Homo sapie	1.325	0.917	1.329	0.933	1.049	1.035	1.313	1.052
Q9NV66	S-adenosyl-L-methionine-dependent tRNA 4-demethylwyosine syr	1.688	1.734	1.221	1.085	1.259	1.492	1.441	0.848
Q8NI37	Protein phosphatase PTC7 homolog OS=Homo sapiens GN=PPTC	1.500	1.508	1.421	1.332	1.402	1.211	1.692	1.265
Q13901	Nuclear nucleic acid-binding protein C1D OS=Homo sapiens GN=	1.467	1.764	0.905	1.014	1.203	1.280	1.554	0.985
P22033	Methylmalonyl-CoA mutase, mitochondrial OS=Homo sapiens GN	1.196	1.415	1.248	0.984	1.007	1.240	1.265	1.196
Q9H8G2	Caspase activity and apoptosis inhibitor 1 OS=Homo sapiens GN=	1.018	1.351	0.855	0.625	0.936	1.189	1.078	0.947
B3KW23	cDNA FLJ41961 fis, clone PUAEN2004083, highly similar to Nucle	1.740	1.667	1.527	1.712	1.400	1.495	1.845	2.054
B2R6F5	cDNA, FLJ92928, highly similar to Homo sapiens retinitis pigment	0.980	1.447	1.030	1.073	1.051	1.282	1.051	1.038
Q16186	Proteasomal ubiquitin receptor ADRM1 OS=Homo sapiens GN=AI	1.545	1.278	1.384	1.017	1.400	1.377	1.263	1.066
Q5BKU9	Oxidoreductase-like domain-containing protein 1 OS=Homo sapie	1.056	1.141	0.861	0.835	0.774	1.032	1.076	0.942

Q5JVS0	Intracellular hyaluronan-binding protein 4 OS=Homo sapiens GN=	1.034	0.930	0.995	0.584	0.857	0.869	0.745	0.588
P46939	Utrophin OS=Homo sapiens GN=UTRN PE=1 SV=2 - [UTRO_HUM	1.365	1.497	1.188	0.993	1.236	1.148	1.146	1.091
Q00653	Nuclear factor NF-kappa-B p100 subunit OS=Homo sapiens GN=N	1.563	1.018	1.028	0.764	1.329	1.219	1.120	0.855
Q86WC4	Osteopetrosis-associated transmembrane protein 1 OS=Homo sap	1.288	1.221	1.118	1.024	1.300	1.205	1.191	1.099
Q9UK61	Protein FAM208A OS=Homo sapiens GN=FAM208A PE=1 SV=3 -	1.268	1.384	1.116	0.936	1.326	1.380	1.407	1.380
Q96SQ9	Cytochrome P450 2S1 OS=Homo sapiens GN=CYP2S1 PE=2 SV=	1.255	1.245	1.221	1.139	1.100	1.181	1.322	1.087
A9UK01	Rho GTPase activating protein OS=Homo sapiens PE=2 SV=1 - [A	0.962	0.863	0.934	0.847	0.944	0.747	0.918	0.824
Q712K3	Ubiquitin-conjugating enzyme E2 R2 OS=Homo sapiens GN=UBE	1.493	1.286	1.379	1.247	1.289	1.099	1.270	1.018
C9JNJ7	Transcription termination factor 4, mitochondrial (Fragment) OS=	1.272	1.520	1.236	1.162	0.781	1.184	1.112	1.147
Q13427	Peptidyl-prolyl cis-trans isomerase G OS=Homo sapiens GN=PPIG	1.912	1.935	1.810	1.509	1.624	1.339	1.740	1.576
B4DDR0	cDNA FLJ55674, highly similar to DNA-directed RNA polymerases	1.553	1.281	1.630	1.338	1.342	1.448	1.504	1.840
Q5XKL0	ALKBH1 protein (Fragment) OS=Homo sapiens GN=ALKBH1 PE=	1.499	1.482	1.322	1.197	1.112	1.313	1.452	1.393
A8K732	cDNA FLJ78771, highly similar to Homo sapiens discs, large homo	1.385	1.106	1.124	0.831	1.084	1.105	1.228	1.143
E9PL33	Etoposide-induced protein 2.4 homolog (Fragment) OS=Homo sa	3.202	3.343	3.063	2.807	2.288	1.294	2.815	2.922
Q9ULT8	E3 ubiquitin-protein ligase HECTD1 OS=Homo sapiens GN=HECT	1.566	1.170	1.461	0.858	1.384	1.279	1.422	0.969
Q96PU4	E3 ubiquitin-protein ligase UHRF2 OS=Homo sapiens GN=UHRF2	1.182	1.732	1.138	0.997	1.404	1.376	1.320	1.232
Q69YR0	Putative uncharacterized protein DKFZp667K0918 (Fragment) OS	1.678	1.891	1.882	1.562	1.663	1.415	1.786	1.911
P29590	Protein PML OS=Homo sapiens GN=PML PE=1 SV=3 - [PML_HUM	1.211	1.092	1.121	1.003	1.095	1.318	1.116	1.007
Q9BVC3	Sister chromatid cohesion protein DCC1 OS=Homo sapiens GN=D	1.476	1.191	1.173	0.813	1.404	1.310	1.092	0.883
M0QXA7	Protein Wiz OS=Homo sapiens GN=WIZ PE=1 SV=1 - [M0QXA7_	1.414	1.565	1.456	1.081	1.261	1.441	1.586	1.215
F6PQP6	Epsin-2 (Fragment) OS=Homo sapiens GN=EPN2 PE=1 SV=3 - [F	1.241	1.541	1.003	1.017	1.165	1.265	0.990	1.022
Q9P0U3	Sentrin-specific protease 1 OS=Homo sapiens GN=SENP1 PE=1 S	1.141	1.376	0.946	1.109	1.182	1.251	1.501	1.231
Q9NZE8	39S ribosomal protein L35, mitochondrial OS=Homo sapiens GN=	1.557	1.644	1.230	1.070	1.082	1.023	1.366	1.323
P78356	Phosphatidylinositol 5-phosphate 4-kinase type-2 beta OS=Homo	1.681	1.396	1.348	1.190	1.498	1.158	1.556	1.479
B4DVN1	cDNA FLJ52214, highly similar to DnaJ homolog subfamily B mem	1.671	1.984	1.719	1.685	1.436	1.688	1.611	1.634
Q92887	Canalicular multispecific organic anion transporter 1 OS=Homo sa	1.445	1.819	1.230	1.196	1.487	1.286	1.266	1.285
Q9H3H3	UPF0696 protein C11orf68 OS=Homo sapiens GN=C11orf68 PE=	1.767	1.063	1.376	1.712	1.308	0.998	0.832	1.073
Q96M27	Protein PRRC1 OS=Homo sapiens GN=PRRC1 PE=1 SV=1 - [PRR	1.205	1.050	1.023	0.886	1.178	1.293	1.134	1.092
Q9UID3	Vacuolar protein sorting-associated protein 51 homolog OS=Hom	2.498	2.366	2.082	1.918	1.856	1.339	2.119	1.844
Q9NUM4	Transmembrane protein 106B OS=Homo sapiens GN=TMEM106B	2.674	1.637	2.098	1.734	2.504	0.996	2.331	1.908
O15031	Plexin-B2 OS=Homo sapiens GN=PLXNB2 PE=1 SV=3 - [PLXB2_H	1.413	1.736	1.349	1.448	1.444	1.242	1.533	1.525
Q9Y3Q3	Transmembrane emp24 domain-containing protein 3 OS=Homo s	1.553	1.466	1.225	1.169	1.272	1.155	1.334	1.249
Q59FY9	LEPREL2 protein variant (Fragment) OS=Homo sapiens PE=4 SV=	0.950	0.738	0.901	1.006	1.107	0.995	1.002	1.091
Q16678	Cytochrome P450 1B1 OS=Homo sapiens GN=CYP1B1 PE=1 SV=	6.817	4.275	2.023	1.391	5.115	3.528	1.969	1.336
Q96RT1	Protein LAP2 OS=Homo sapiens GN=ERBB2IP PE=1 SV=2 - [LAP	1.223	1.816	1.193	0.980	0.982	1.296	1.253	1.039
Q9UHL4	Dipeptidyl peptidase 2 OS=Homo sapiens GN=DPP7 PE=1 SV=3	1.234	1.071	1.441	1.382	1.270	1.054	1.323	1.459
P78536	Disintegrin and metalloproteinase domain-containing protein 17 O	0.917	1.017	0.728	0.756	0.942	0.987	0.772	0.771
E5KSE7	Mitochondrial short-chain specific acyl-CoA dehydrogenase OS=H	1.111	1.024	0.935	1.190	0.869	1.083	1.012	0.951
B4DJ65	Major prion protein OS=Homo sapiens PE=2 SV=1 - [B4DJ65_HU	1.649	2.922	0.990	0.642	1.383	1.492	1.382	0.824
Q9Y3A6	Transmembrane emp24 domain-containing protein 5 OS=Homo s	2.055	2.019	1.824	1.792	1.713	1.246	1.912	1.556

Q06136	3-ketodihydrosphingosine reductase OS=Homo sapiens GN=KDSI	1.529	1.326	1.271	1.148	1.196	1.058	1.378	1.181
C9J6V2	Retinitis pigmentosa 9 protein (Fragment) OS=Homo sapiens GN=	1.430	2.353	1.052	1.155	1.357	1.322	1.257	1.269
A8KA19	cDNA FLJ75831, highly similar to Homo sapiens exportin, tRNA (r	2.768	2.022	2.619	1.816	2.014	1.244	2.050	1.946
B4DI81	Gap junction protein OS=Homo sapiens PE=2 SV=1 - [B4DI81_H	1.682	1.037	0.602	0.463	1.669	0.774	1.091	0.889
F8W0R1	Relaxin-3 (Fragment) OS=Homo sapiens GN=RLN3 PE=4 SV=1 -	1.103	1.144	1.065	1.067	1.020	1.039	1.104	1.071
Q3YEC7	Rab-like protein 6 OS=Homo sapiens GN=RABL6 PE=1 SV=2 - [R	1.247	0.827	1.036	0.773	0.995	0.964	1.034	0.834
Q8WVK2	U4/U6.U5 small nuclear ribonucleoprotein 27 kDa protein OS=Ho	0.719	0.752	0.641	0.657	0.737	0.850	0.753	0.802
B3KST9	cDNA FLJ36938 fis, clone BRACE2005434, highly similar to Histor	1.245	1.371	1.254	0.965	1.349	1.797	1.512	1.194
P27448	MAP/microtubule affinity-regulating kinase 3 OS=Homo sapiens G	1.840	1.181	1.833	1.211	1.424	1.243	1.556	1.431
B3KRW4	cDNA FLJ34980 fis, clone OCBBF2000522, highly similar to Rab G	1.255	1.083	1.240	1.084	1.075	1.177	1.431	1.143
P10109	Adrenodoxin, mitochondrial OS=Homo sapiens GN=FDX1 PE=1 S	0.654	0.667	0.612	0.715	0.928	1.139	1.039	1.059
C9J494	Probable E3 ubiquitin-protein ligase makorin-2 OS=Homo sapiens	2.851	2.665	2.827	1.852	1.647	1.420	2.091	2.389
A6NND8	Quinone oxidoreductase-like protein 1 OS=Homo sapiens GN=CR	1.724	1.487	1.794	1.355	1.694	1.116	1.585	1.244
Q15650	Activating signal cointegrator 1 OS=Homo sapiens GN=TRIP4 PE=	1.297	1.290	1.095	0.812	1.055	1.001	1.115	1.004
Q8NBZ7	UDP-glucuronic acid decarboxylase 1 OS=Homo sapiens GN=UXS	1.344	1.401	1.095	1.104	1.197	1.394	1.212	1.028
A0A087X0P4	Pre-B-cell leukemia transcription factor 1 OS=Homo sapiens GN=	1.110	1.086	0.979	0.872	1.209	1.051	1.197	0.902
Q53F88	General transcription factor IIE, polypeptide 1 (Alpha subunit, 56l	1.455	1.561	1.409	1.027	2.006	1.345	1.451	1.099
O95090	Battenin (Fragment) OS=Homo sapiens GN=CLN3 PE=1 SV=1 - [	1.503	1.489	1.381	1.330	1.638	1.287	1.730	1.458
Q8N954	G patch domain-containing protein 11 OS=Homo sapiens GN=GP	2.042	1.788	2.090	1.630	1.658	1.522	2.038	1.690
Q7Z4H8	KDEL motif-containing protein 2 OS=Homo sapiens GN=KDELC2	1.299	1.116	1.440	1.727	1.225	1.206	1.936	1.797
O96028	Histone-lysine N-methyltransferase NSD2 OS=Homo sapiens GN=	1.549	1.820	1.273	1.181	1.324	1.313	1.671	1.553
Q13144	Translation initiation factor eIF-2B subunit epsilon OS=Homo sapi	1.458	1.059	1.398	1.107	1.361	1.086	1.284	1.314
A8K274	cDNA FLJ78227, highly similar to Homo sapiens pituitary tumor-tr	2.812	2.548	2.046	1.772	2.037	1.475	2.194	1.910
Q9BT25	HAUS augmin-like complex subunit 8 OS=Homo sapiens GN=HAU	1.261	1.142	1.223	0.946	1.172	1.050	1.002	0.959
J3KS17	Beta-2-glycoprotein 1 (Fragment) OS=Homo sapiens GN=APOH F	1.895	1.331	3.553	4.192	2.414	1.699	2.196	1.887
Q7Z7E8	Ubiquitin-conjugating enzyme E2 Q1 OS=Homo sapiens GN=UBE	1.527	1.249	1.329	1.236	1.288	1.076	1.576	1.359
O15116	U6 snRNA-associated Sm-like protein LSm1 OS=Homo sapiens GI		0.805				1.277		
B4E205	cDNA FLJ61651, highly similar to Protein transport protein Sec24/	1.501	1.166	1.457	1.220	1.386	1.220	1.634	1.295
A0A024RDQ7	Mitochondrial translational initiation factor 3, isoform CRA_a OS=	1.263	1.543	1.210	1.108	1.166	1.319	1.294	1.139
B8ZZL5	MIT domain-containing protein 1 (Fragment) OS=Homo sapiens (	1.167	1.341	0.992	0.810	1.273	1.087	1.142	1.084
B4E355	cDNA FLJ60398, highly similar to Transforming acidic coiled-coil-c	2.345	1.849	2.887	1.627	1.597	1.523	3.669	3.902
P36941	Tumor necrosis factor receptor superfamily member 3 OS=Homo	2.343	2.383	1.652	1.336	1.871	1.441	1.582	1.286
Q9NWK9	Box C/D snoRNA protein 1 OS=Homo sapiens GN=ZNHIT6 PE=1	1.527	2.077	1.453	0.907	1.461	1.517	1.438	1.209
Q9P275	Ubiquitin carboxyl-terminal hydrolase 36 OS=Homo sapiens GN=l	1.421	1.603	1.345	1.180	1.317	1.347	1.390	1.434
Q8IUC4	Rhophilin-2 OS=Homo sapiens GN=RHPN2 PE=1 SV=1 - [RHPN2	1.240	0.594	0.297	0.491	1.176	0.726	0.467	0.312
P0CG12	Chromosome transmission fidelity protein 8 homolog isoform 2 OS	0.675	0.507	0.269		0.643	0.538	0.345	
G5E9A6	Ubiquitin carboxyl-terminal hydrolase OS=Homo sapiens GN=USF	1.561	1.156	0.975	0.682	1.540	1.217	1.027	0.977
Q9UKK3	Poly [ADP-ribose] polymerase 4 OS=Homo sapiens GN=PARP4 PE	1.504	1.088	1.153	0.900	1.345	1.035	1.165	1.024
Q5TA45	Integrator complex subunit 11 OS=Homo sapiens GN=CPSF3L PE	1.292	1.350	1.201	0.874	1.401	1.350	1.304	1.133
Q9GZP9	Derlin-2 OS=Homo sapiens GN=DERL2 PE=1 SV=1 - [DERL2_HU	1.375	1.434	1.698	1.797	1.037	1.604	1.615	1.091

D6W625	Chromatin assembly factor 1, subunit A (P150), isoform CRA_a O	1.513	2.045	1.470	1.577	1.256	1.641	1.950	1.811
O14893	Gem-associated protein 2 OS=Homo sapiens GN=GEMIN2 PE=1	1.405	1.196	1.400	1.086	1.267	1.177	1.424	1.233
Q56NI9	N-acetyltransferase ESCO2 OS=Homo sapiens GN=ESCO2 PE=1 S	1.653	2.772	2.058	1.786	1.846	2.002	2.670	2.513
Q5SVL2	Caspase-7 (Fragment) OS=Homo sapiens GN=CASP7 PE=1 SV=1	1.388	0.982	1.481	1.144	1.234	1.070	1.265	1.086
Q8IVL6	Prolyl 3-hydroxylase 3 OS=Homo sapiens GN=LEPREL2 PE=1 SV=			1.117	0.954	0.722	0.983	0.720	1.153
Q13825	Methylglutaconyl-CoA hydratase, mitochondrial OS=Homo sapien	1.495	1.414	1.312	1.311	1.032	1.094	1.303	1.243
B7ZVZ1	SEPT8 protein OS=Homo sapiens GN=SEPT8 PE=2 SV=1 - [B7ZV	2.460	1.991	3.183	2.277	1.966	1.182	2.274	2.356
A0A024RAP0	X-ray repair complementing defective repair in Chinese hamster c	1.550	1.087	0.986	0.924	1.596	1.436	1.459	1.349
Q9NX46	Poly(ADP-ribose) glycohydrolase ARH3 OS=Homo sapiens GN=AI	1.286	1.120	1.285	1.391	1.246	0.995	1.264	1.390
Q53HC9	Protein TSSC1 OS=Homo sapiens GN=TSSC1 PE=1 SV=2 - [TSS	1.510	1.034	1.474	1.182	1.396	1.134	1.296	1.326
Q8NG11	Tetraspanin-14 OS=Homo sapiens GN=TSPAN14 PE=1 SV=1 - [T	1.883	1.671	0.972	0.882	1.431	1.068	1.116	0.719
Q8NF37	Lysophosphatidylcholine acyltransferase 1 OS=Homo sapiens GN=	1.477	1.545	1.382	1.377	1.308	1.285	1.446	1.279
P68402	Platelet-activating factor acetylhydrolase IB subunit beta OS=Hon	1.905		1.311			1.231		1.395
B4DPP0	cDNA FLJ51032, highly similar to CD9 antigen OS=Homo sapiens	3.134	3.557	2.691	2.656	2.811	1.621	3.116	2.723
Q9NUY8	TBC1 domain family member 23 OS=Homo sapiens GN=TBC1D2	1.819	2.308	1.675	1.373	1.634	1.336	1.594	2.043
Q9BT40	Inositol polyphosphate 5-phosphatase K OS=Homo sapiens GN=I	1.663	1.955	1.664	1.553	1.663	1.428	1.819	1.633
A0A024RE20	Uncharacterized protein OS=Homo sapiens GN=FLJ11193 PE=4 S	1.071	1.118	1.254	1.070	1.220	1.082	1.235	0.803
B3KP18	cDNA FLJ30946 fis, clone FEBRA2007622, highly similar to RAD5	1.759	1.918	2.112	1.881	2.035	1.569	2.176	2.188
Q9H6V9	UPF0554 protein C2orf43 OS=Homo sapiens GN=C2orf43 PE=1 S	1.703	1.913	1.837	1.755	1.822	1.471	2.110	2.083
B7Z5X7	cDNA FLJ50844, highly similar to Tryptophanyl-tRNA synthetase,	1.199	1.409	1.636	1.142	0.861	1.116	1.415	1.282
O95900	Probable tRNA pseudouridine synthase 2 OS=Homo sapiens GN=	1.546	1.565	1.323	1.213	1.184	1.184	1.442	1.279
Q59GZ8	Urokinase plasminogen activator preproprotein variant (Fragment	1.125	0.524	0.201	0.161	1.113	0.529	0.246	
A0A024RDV5	Fibronectin type III domain containing 3A, isoform CRA_b OS=Ho	1.431	1.679	1.394	1.277	1.270	1.377	1.460	1.439
B4DVB7	cDNA FLJ55470, highly similar to Chaperone-activity of bc1 comp	1.161	1.764	1.632	1.554	1.459	1.291	1.832	1.804
Q9BV79	Trans-2-enoyl-CoA reductase, mitochondrial OS=Homo sapiens G	1.396	1.564	1.281	1.277	1.167	1.241	1.438	1.504
A0A024R8K7	Integrin beta OS=Homo sapiens GN=ITGB4 PE=3 SV=1 - [A0A02	1.043	0.958	0.698	0.708	0.920	0.915	0.753	0.661
Q8IWE2	Protein NOXP20 OS=Homo sapiens GN=FAM114A1 PE=1 SV=2 -	2.531	2.024	2.146	1.607	2.052	1.184	1.956	2.288
Q8WVT3	Trafficking protein particle complex subunit 12 OS=Homo sapiens	1.222	1.035	1.236	0.875	1.206	1.003	1.157	0.906
Q9NUQ3	Gamma-taxilin OS=Homo sapiens GN=TXLNG PE=1 SV=2 - [TXL	1.568	1.315	1.523	1.158	1.360	1.325	1.573	1.220
Q7Z3R8	DNA-directed RNA polymerase OS=Homo sapiens GN=DKFZp686	1.363	1.599	1.475	1.044	1.246	1.356	1.563	1.331
Q53GS8	Wolf-Hirschhorn syndrome candidate 2 protein variant (Fragment	0.955	1.087	0.793	0.566	1.125	1.196	0.939	0.598
S5FMB0	PHF10 isoform Ps OS=Homo sapiens PE=2 SV=1 - [S5FMB0_HUI	1.568	1.895	1.486	1.319	1.535	1.389	1.680	1.445
H7C128	Bromodomain-containing protein 8 (Fragment) OS=Homo sapiens	1.566	1.727	1.189	0.985	1.281	1.302	1.498	1.273
Q59GJ0	Eukaryotic translation initiation factor 4 gamma, 3 variant (Fragm	1.250	1.133	1.179	0.888	1.070	1.132	1.113	0.849
Q86WA8	Lon protease homolog 2, peroxisomal OS=Homo sapiens GN=LOI	1.270	1.820	1.048	0.811	1.304	1.540	1.619	0.885
B7Z5W0	cDNA FLJ53174, highly similar to Numb-like protein OS=Homo sa	1.142	1.037	0.939	0.547	1.425	0.798	0.811	0.628
E9PEI0	Cell division cycle-associated protein 2 OS=Homo sapiens GN=CI	1.404	1.836	1.023	0.888	1.253	1.369	1.436	1.046
B5BU41	Calcium/calmodulin-dependent protein kinase I OS=Homo sapien	0.883		0.840	0.603	0.792		0.977	0.970
Q9NVR2	Integrator complex subunit 10 OS=Homo sapiens GN=INTS10 PE	1.552	1.808	1.280	1.196	1.688	1.619	1.450	1.474
Q8NCH0	Carbohydrate sulfotransferase 14 OS=Homo sapiens GN=CHST14	1.442	1.931	1.492	1.261	1.204	1.562	1.685	1.545

E7EVH9	Pseudouridine-5'-monophosphatase (Fragment) OS=Homo sapien	2.756	2.180	2.718	2.535	2.407	1.421	2.100	2.289
Q8NDZ4	Deleted in autism protein 1 OS=Homo sapiens GN=C3orf58 PE=1	1.634	2.018	1.614	1.644	1.472	1.625	1.835	1.800
Q8WY22	BRI3-binding protein OS=Homo sapiens GN=BRI3BP PE=1 SV=1	1.613	2.005	1.771	2.858	1.445	1.298	1.831	1.502
B4DIQ0	cDNA FLJ51692, highly similar to mRNA capping enzyme OS=Hor	1.069	1.043	0.962	0.616	1.270	1.182	1.020	0.878
Q9NV88	Integrator complex subunit 9 OS=Homo sapiens GN=INTS9 PE=1	1.762	1.981	1.679	1.412	2.055	1.521	2.137	1.642
P15848	Arylsulfatase B OS=Homo sapiens GN=ARSB PE=1 SV=1 - [ARSB	0.999	0.915	1.132	1.196	1.123	1.084	1.354	1.319
O75427	Leucine-rich repeat and calponin homology domain-containing pro	1.423	1.812	1.069	1.133	1.430	1.420	1.396	1.231
A0A087WZA9	Transmembrane protein 120A (Fragment) OS=Homo sapiens GN=	1.617	1.631	1.360	1.220	1.301	1.199	1.495	1.322
Q9Y294	Histone chaperone ASF1A OS=Homo sapiens GN=ASF1A PE=1 SV	1.699	1.053	0.810	0.461	1.661	1.203	1.312	0.960
Q9ULK4	Mediator of RNA polymerase II transcription subunit 23 OS=Homo	1.630	1.561	1.403	0.908	1.616	1.359	1.558	1.045
Q86Y91	Kinesin-like protein KIF18B OS=Homo sapiens GN=KIF18B PE=1	1.800	2.261	1.233	0.815	2.051	2.070	2.197	1.032
Q99808	Equilibrative nucleoside transporter 1 OS=Homo sapiens GN=SLC	1.301	1.688	1.647	1.477	1.393	1.536	1.437	1.646
Q9NQ34	Transmembrane protein 9B OS=Homo sapiens GN=TMEM9B PE=	3.874	4.715	4.002	3.717	2.302	1.757	3.265	2.826
B4DE67	cDNA FLJ57259, highly similar to Lysosomal acid lipase/cholester	2.594	1.981	1.387	1.268	2.942	1.298	2.477	1.737
H3BRL3	Ubiquitin domain-containing protein UBFD1 OS=Homo sapiens GN	1.379	0.984	1.409	1.186	1.303	1.194	1.372	1.341
Q9BS40	Latexin OS=Homo sapiens GN=LXN PE=1 SV=2 - [LXN_HUMAN]	1.111	0.955	1.324	1.230	1.094	0.990	1.208	0.989
O95140	Mitofusin-2 OS=Homo sapiens GN=MFN2 PE=1 SV=3 - [MFN2_H	1.869	2.545	1.736	1.664	1.655	1.696	1.981	1.712
B7ZAV4	cDNA, FLJ79318, highly similar to 24-dehydrocholesterol reductas	1.690	1.684	2.101	1.869	1.551	1.165	1.874	1.323
B4E2X3	cDNA FLJ56024 OS=Homo sapiens PE=2 SV=1 - [B4E2X3_HUMA	1.621	1.038	1.418	1.090	1.451	1.153	1.250	0.876
Q96SI1	BTB/POZ domain-containing protein KCTD15 OS=Homo sapiens G	1.420	1.450	1.419	0.953	1.144	1.460	1.508	1.131
J3QSH4	Vascular endothelial zinc finger 1 OS=Homo sapiens GN=VEZF1 P	2.266	2.335	2.005	1.742	1.623	1.218	1.868	1.938
O60287	Nucleolar pre-ribosomal-associated protein 1 OS=Homo sapiens G	1.624	1.731	1.360	1.242	1.358	1.376	1.722	1.359
Q9BVQ7	Spermatogenesis-associated protein 5-like protein 1 OS=Homo sa	1.477	1.243	1.388	0.875	1.358	1.198	1.311	1.267
B3KVN0	cDNA FLJ16785 fis, clone NT2RI2015342, highly similar to Solute	1.318	1.679	1.151	1.164	1.292	1.262	1.234	1.132
O00468	Agrin OS=Homo sapiens GN=AGRN PE=1 SV=5 - [AGRIN_HUMA	1.206	1.120	0.968	0.835	1.045	0.934	1.170	0.995
O43464	Serine protease HTRA2, mitochondrial OS=Homo sapiens GN=HT	1.758	1.638	1.382	1.324	1.521	1.308	1.499	1.489
P15529	Membrane cofactor protein OS=Homo sapiens GN=CD46 PE=1 SV	2.112	2.552	2.245	2.251	1.845	1.670	2.106	2.290
Q14676	Mediator of DNA damage checkpoint protein 1 OS=Homo sapiens	1.120	1.488	1.205	1.006	1.071	1.332	1.346	1.172
P16422	Epithelial cell adhesion molecule OS=Homo sapiens GN=EPCAM P	1.088	3.292	3.298	3.858	0.956	1.685	3.508	3.745
Q00534	Cyclin-dependent kinase 6 OS=Homo sapiens GN=CDK6 PE=1 SV	1.376	0.665	0.784	0.731	1.311	0.787	0.857	0.556
J3KNN7	BRCA1-associated protein OS=Homo sapiens GN=BRAP PE=1 SV	0.813	0.591	0.983	0.747	0.848	1.412	1.205	0.739
A0A024R6C8	ATP-binding cassette, sub-family D (ALD), member 4, isoform CR	1.383	1.311	1.089	1.358	1.118	1.129	1.186	1.084
Q7L5N7	Lysophosphatidylcholine acyltransferase 2 OS=Homo sapiens GN=	1.359	1.789	1.933	2.140	1.098	1.465	2.321	2.149
P08582	Melanotransferrin OS=Homo sapiens GN=MFI2 PE=1 SV=2 - [TR	1.580	1.958	1.108	1.080	1.586	1.021	1.428	1.229
Q567U8	COPS7A protein OS=Homo sapiens GN=COPS7A PE=2 SV=1 - [Q	1.015	1.010	0.798	0.612	1.211	1.008	0.862	0.769
Q9BYB4	Guanine nucleotide-binding protein subunit beta-like protein 1 OS	0.871	0.560	0.885	0.998	1.257	1.041	0.956	0.717
Q8IYS1	Peptidase M20 domain-containing protein 2 OS=Homo sapiens GN	1.413	2.391	1.389	1.784	1.209	1.066	1.323	1.731
Q969E4	Transcription elongation factor A protein-like 3 OS=Homo sapiens	0.811	0.597	0.959	0.664	0.927	1.074	0.835	0.884
A0A024R5E8	Histone acetyltransferase OS=Homo sapiens GN=HTATIP PE=3 S	0.670	0.807	0.707	0.579	0.690	0.946	0.849	0.825
B4DJ23	Myotubularin-related protein 14 OS=Homo sapiens GN=MTMR14	2.694	1.984	3.027	2.261	2.109	1.250	2.443	2.110

Q68E01	Integrator complex subunit 3 OS=Homo sapiens GN=INTS3 PE=1	1.623	1.631	1.250	0.962	1.719	1.463	1.672	1.270
Q9H6X2	Anthrax toxin receptor 1 OS=Homo sapiens GN=ANTXR1 PE=1 S	1.239	1.257	0.968	0.760	1.207	1.359	1.021	0.848
E5RHU1	Transmembrane protein 68 (Fragment) OS=Homo sapiens GN=TI	1.608	1.453	1.472	1.274	0.786	1.168	1.529	1.248
B2RMV2	CYTSA protein OS=Homo sapiens GN=CYTSA PE=2 SV=1 - [B2RI	1.365	1.022	0.588	0.299	1.022	1.146	0.756	0.440
B4DY46	cDNA FLJ53447, highly similar to Syntaxin-binding protein 2 OS=I	1.133	0.998	1.188	1.149	1.061	1.146	0.985	0.906
Q96G74	OTU domain-containing protein 5 OS=Homo sapiens GN=OTUD5	1.625	1.614	1.684	1.278	1.719	1.858	1.870	1.524
B2RAQ5	cDNA, FLJ95054 OS=Homo sapiens PE=2 SV=1 - [B2RAQ5_HUM	0.991	1.415	1.128	1.115	1.420	1.302	1.337	1.389
Q12834	Cell division cycle protein 20 homolog OS=Homo sapiens GN=CD	0.957	1.166	1.200	1.137	1.158	1.826	1.627	1.269
Q9P2B4	CTTNBP2 N-terminal-like protein OS=Homo sapiens GN=CTTNBP2	1.238	0.858	0.801	0.507	1.034	1.086	0.857	0.884
Q9UBW7	Zinc finger MYM-type protein 2 OS=Homo sapiens GN=ZMYM2 PI	1.262	1.320	0.955	0.740	1.156	1.355	1.067	0.706
B3KM95	Phosphatidate cytidylyltransferase OS=Homo sapiens PE=2 SV=1	1.780	1.717	1.252	1.363	1.390	1.100	1.587	1.238
Q96N67	Dedicator of cytokinesis protein 7 OS=Homo sapiens GN=DOCK7	1.855	1.459	1.672	1.372	1.424	1.286	1.903	1.560
B1AUU8	Epidermal growth factor receptor substrate 15 OS=Homo sapiens	2.660	1.904	3.117	2.364	2.021	1.218	1.999	2.348
B2R9X3	cDNA, FLJ94599, highly similar to Homo sapiens GDP-mannose 4	1.457	1.010	1.486	1.222	1.157	1.019	1.476	1.318
Q75ME3	Putative uncharacterized protein WBSCR22 OS=Homo sapiens GN	1.354	1.452	1.185	0.925	1.420	1.332	1.359	1.141
Q7Z406	Myosin-14 OS=Homo sapiens GN=MYH14 PE=1 SV=2 - [MYH14_	1.750	1.376	1.397	1.006	1.297	1.146	1.176	1.053
O14617	AP-3 complex subunit delta-1 OS=Homo sapiens GN=AP3D1 PE=	2.357	1.458	1.915	1.289	1.831	1.116	1.623	1.304
Q8WTS6	Histone-lysine N-methyltransferase SETD7 OS=Homo sapiens GN	1.391	0.831	0.971	0.915	1.263	1.117	1.069	0.859
Q9UNN8	Endothelial protein C receptor OS=Homo sapiens GN=PROCR PE=		1.176	1.326			1.362	1.061	
Q15345	Leucine-rich repeat-containing protein 41 OS=Homo sapiens GN=	1.128	1.411	1.430	0.940	0.920	1.212	1.516	1.289
A0A087X239	Round spermatid basic protein 1-like protein OS=Homo sapiens G	1.773	1.744	1.633	1.248	1.659	1.399	2.067	1.590
O60524	Nuclear export mediator factor NEMF OS=Homo sapiens GN=NEM	1.334	1.054	1.267	0.898	1.164	0.981	1.039	0.970
Q8N5W9	Protein FAM101B OS=Homo sapiens GN=FAM101B PE=1 SV=1 -	1.285	1.308	1.019	0.944	1.251	0.980	1.170	1.388
B4DDG9	cDNA FLJ53856, highly similar to 5-aminolevulinate synthase, nor	2.119	2.418	1.353	1.067	1.734	1.990	1.896	1.206
Q15628	Tumor necrosis factor receptor type 1-associated DEATH domain	1.117	0.797	1.302	0.955	1.197	1.113	0.995	1.027
A0A024R5B2	MAP/microtubule affinity-regulating kinase 2, isoform CRA_c OS=	1.744	1.385	1.521	1.076	1.442	1.474	1.513	1.476
B2RDT8	cDNA, FLJ96764, highly similar to Homo sapiens sorting nexin 8 (	1.919	1.131	1.506	0.548	1.182	0.933	1.161	0.643
O14773	Tripeptidyl-peptidase 1 OS=Homo sapiens GN=TPP1 PE=1 SV=2	1.219	1.077	1.388	1.413	1.270	1.096	1.437	1.506
Q8IWZ3	Ankyrin repeat and KH domain-containing protein 1 OS=Homo sa	2.057	2.013	1.974	1.182	1.756	1.124	1.963	1.784
Q96DG6	Carboxymethylenebutenolidase homolog OS=Homo sapiens GN=	2.049	1.307	1.857	1.557	1.423	1.016	1.765	1.687
Q9UJY1	Heat shock protein beta-8 OS=Homo sapiens GN=HSPB8 PE=1 S	1.208	0.742	0.983	0.620	1.197	1.043	1.012	0.821
Q12899	Tripartite motif-containing protein 26 OS=Homo sapiens GN=TRI	1.784	1.681	2.219	2.274	1.620	1.305	1.981	2.306
P25774	Cathepsin S OS=Homo sapiens GN=CTSS PE=1 SV=3 - [CATS_HU	1.564	1.094	1.766	1.266	1.622	1.242	1.382	1.346
Q5VT94	Growth hormone-inducible transmembrane protein OS=Homo sap	1.591	1.697	1.234	1.191	1.121	1.098	1.521	1.430
H3BQV3	Conserved oligomeric Golgi complex subunit 8 OS=Homo sapiens	1.783	1.603	1.671	1.475	1.164	1.053	1.258	1.143
Q99805	Transmembrane 9 superfamily member 2 OS=Homo sapiens GN=	1.641	1.617	1.269	1.397	1.252	1.265	1.560	1.377
Q86X55	Histone-arginine methyltransferase CARM1 OS=Homo sapiens GN	2.404	1.980	2.512	1.276	2.041	1.462	2.255	1.604
F5GWD3	General transcription factor IIH subunit 3 (Fragment) OS=Homo s	1.226	1.247	1.117	1.180	1.238	1.221	1.346	1.358
Q9Y5N6	Origin recognition complex subunit 6 OS=Homo sapiens GN=ORC	0.871	1.143	0.889	0.605	1.061	1.569	1.205	0.842
Q9BQ70	Transcription factor 25 OS=Homo sapiens GN=TCF25 PE=1 SV=1	2.111	2.060	1.848	1.468	2.289	1.533	2.123	1.769

Q9Y3E1	Hepatoma-derived growth factor-related protein 3 OS=Homo sap	3.611	3.626	2.946	2.271	2.862	1.375	2.927	2.624
Q53EN0	Zinc finger protein 330 variant (Fragment) OS=Homo sapiens PE=	1.192	1.116	1.143	1.083	1.053	0.983	1.081	0.999
Q96EZ8	Microspherule protein 1 OS=Homo sapiens GN=MCRS1 PE=1 SV=	1.007	1.177	0.900	0.520	1.117	1.406	1.000	1.035
Q8WUQ7	Cactin OS=Homo sapiens GN=CACTIN PE=1 SV=3 - [CATIN_HUM	1.495	1.518	1.397	0.973	1.563	1.391	1.345	1.128
Q6UW02	Cytochrome P450 20A1 OS=Homo sapiens GN=CYP20A1 PE=2 S	1.512	1.424	1.083	1.059	1.091	1.263	1.356	0.952
Q8WTW3	Conserved oligomeric Golgi complex subunit 1 OS=Homo sapiens	1.076	1.626	1.276	0.687	1.205	1.262	1.173	1.013
Q8TCJ2	Dolichyl-diphosphooligosaccharide--protein glycosyltransferase su	1.451	1.468	1.405	1.282	1.187	1.211	1.548	1.203
X5CMH5	TAP2 OS=Homo sapiens GN=TAP2 PE=3 SV=1 - [X5CMH5_HUMA	1.275	0.598	0.377	0.377	0.955	0.703	0.516	0.466
J3QQW9	Polycomb protein SUZ12 OS=Homo sapiens GN=SUZ12 PE=1 SV	1.329	1.725	1.247	0.939	1.415	1.587	1.545	1.418
Q2KS23	DNA polymerase OS=Human adenovirus C serotype 5 GN=E2B P	0.879	0.774	1.061	1.330	0.666	1.170	2.135	1.049
B4DJN0	cDNA FLJ51641, highly similar to Nicastrin OS=Homo sapiens PE=	1.393	1.051	1.029	0.815	1.187	1.269	1.168	1.091
Q9H981	Actin-related protein 8 OS=Homo sapiens GN=ACTR8 PE=1 SV=2	1.517	1.466	1.378	1.720	1.487	1.514	1.404	1.349
D3DR40	Chromosome 10 open reading frame 4, isoform CRA_b OS=Homo	2.458	1.991	2.572	1.991	2.313	1.607	2.028	1.879
A8K8N5	Ubiquitin carboxyl-terminal hydrolase OS=Homo sapiens PE=2 S	1.498	0.986	1.350	1.035	1.335	1.155	1.343	1.116
Q96Q45	Transmembrane protein 237 OS=Homo sapiens GN=TMEM237 P	1.676	1.957	1.577	1.094	1.146	1.294	1.632	1.358
Q01650	Large neutral amino acids transporter small subunit 1 OS=Homo	2.000	2.930	1.894	2.334	1.779	1.635	1.756	1.958
A8K1C7	cDNA FLJ75179, highly similar to Homo sapiens leucine rich repe	1.478	1.845	1.385	1.357	1.496	1.273	1.493	1.377
P78545	ETS-related transcription factor Elf-3 OS=Homo sapiens GN=ELF	0.742	0.486	0.226	0.217	0.767	0.688	0.398	0.245
A0A087WYU1	Sorting nexin-9 OS=Homo sapiens GN=SNX9 PE=4 SV=1 - [A0A	2.027	1.736	2.022	1.511	1.644	1.183	1.985	1.940
A0PJB6	C10orf118 protein (Fragment) OS=Homo sapiens GN=C10orf118	2.404					1.551		
Q9BR76	Coronin-1B OS=Homo sapiens GN=CORO1B PE=1 SV=1 - [COR1	1.414	1.007	1.243	0.931	1.503	1.128	1.267	1.349
A1L4K2	Mitogen-activated protein kinase 8 OS=Homo sapiens GN=MAPK	1.967	1.169	2.024		2.077	1.952	1.972	1.826
Q99661	Kinesin-like protein KIF2C OS=Homo sapiens GN=KIF2C PE=1 S	1.581	1.309	1.056	0.997	1.280	0.967	1.050	0.890
F2Z2V0	Copine-1 (Fragment) OS=Homo sapiens GN=CPNE1 PE=1 SV=1	1.936	1.142	1.515	1.213	1.928	1.045	1.451	1.308
Q8TCG1	Protein CIP2A OS=Homo sapiens GN=KIAA1524 PE=1 SV=2 - [C	1.522	1.053	1.368	1.090	1.325	1.073	1.253	1.293
X6RI37	Leucine-rich repeat protein SHOC-2 (Fragment) OS=Homo sapien	1.076	1.531	1.037	0.973	1.200	0.894	1.127	0.831
X5D289	Acid phosphatase 6, lysophosphatidic (Fragment) OS=Homo sapi	1.394	1.319	1.602	1.087	1.311	1.587	1.042	1.635
Q96GX9	Methylthioribulose-1-phosphate dehydratase OS=Homo sapiens	2.988	2.361	3.358	1.727	2.066	1.233	2.123	2.105
B4DP60	cDNA FLJ54324 OS=Homo sapiens PE=2 SV=1 - [B4DP60_HUMA	2.050	1.392	2.175	1.387	1.601	1.202	1.697	1.571
B2ZGL7	Monoacylglycerol lipase isoform 2 OS=Homo sapiens GN=MGLL	1.108	0.847	0.736	0.489	0.990	0.969	0.730	0.618
A0A024QZN8	Hect domain and RLD 4, isoform CRA_b OS=Homo sapiens GN=	1.666	1.290	1.315	0.915	1.712	1.050	1.296	1.173
Q9NWV8	BRISC and BRCA1-A complex member 1 OS=Homo sapiens GN=	1.251	0.897	1.099	0.912	1.129	1.010	1.066	1.035
E9PIF4	Sialidase-1 OS=Homo sapiens GN=NEU1 PE=1 SV=1 - [E9PIF4_	1.533	1.415	1.434	1.061	1.037	1.202	1.140	1.188
A0A024R7W5	YTH domain family, member 3, isoform CRA_a OS=Homo sapiens	1.206	1.350	1.239	0.885	1.072	1.303	1.231	1.056
Q9Y217	Myotubularin-related protein 6 OS=Homo sapiens GN=MTMR6 P	1.264	0.915	1.064	0.956	1.445	1.061	1.148	0.991
Q5T1M5	FK506-binding protein 15 OS=Homo sapiens GN=FKBP15 PE=1	1.284	1.169	1.384	0.858	1.290	0.951	1.361	1.121
Q6NUQ2	Calmin (Calponin-like, transmembrane) OS=Homo sapiens GN=C	1.725	1.945	1.410	1.185	1.483	1.324	1.414	1.258
Q9NW15	Anoctamin-10 OS=Homo sapiens GN=ANO10 PE=1 SV=2 - [ANO	1.665	1.743	1.554	1.619	1.230	1.273	1.460	1.337
Q13190	Syntaxin-5 OS=Homo sapiens GN=STX5 PE=1 SV=2 - [STX5_HU	1.438	1.480	1.237	1.236	1.236	1.160	1.369	1.412
B4DSN8	cDNA FLJ60863, highly similar to High mobility group protein 2-li	1.597	2.052	1.055	0.940	1.166	1.351	2.038	1.427

Q96EB6	NAD-dependent protein deacetylase sirtuin-1 OS=Homo sapiens (	1.007	1.111	0.898	0.647	1.190	1.117	0.886	0.754
A0A024QZ48	Integrator complex subunit 2, isoform CRA_a OS=Homo sapiens (	1.454	1.774	1.272	1.032	1.656	1.590	1.499	1.267
Q99442	Translocation protein SEC62 OS=Homo sapiens GN=SEC62 PE=1	1.594	1.606	1.438	1.363	1.317	1.472	1.739	1.542
Q9BQA9	Uncharacterized protein C17orf62 OS=Homo sapiens GN=C17orf(	3.046	3.147	2.343	2.938	2.694	1.174	3.681	2.975
Q8N6M0	OTU domain-containing protein 6B OS=Homo sapiens GN=OTUD	1.398	1.248	1.413	1.035	1.388	1.044	1.149	1.371
Q8N3C0	Activating signal cointegrator 1 complex subunit 3 OS=Homo sap	1.496	1.356	1.318	1.005	1.253	1.043	1.301	1.237
F8VSL3	Nuclear transcription factor Y subunit beta (Fragment) OS=Homo	1.524	1.668	1.513	1.373	1.626	1.346	1.573	1.710
Q9BTX1	Nucleoporin NDC1 OS=Homo sapiens GN=NDC1 PE=1 SV=2 - [N	1.286	1.242	1.022	0.990	0.905	1.053	1.334	1.090
B2RDB1	cDNA, FLJ96533, highly similar to Homo sapiens golgi coiled-coil	1.249	1.340	1.015	0.918	1.183	1.469	0.965	0.897
B3KMT5	cDNA FLJ12554 fis, clone NT2RM4000741, highly similar to SGT1	2.357	1.495	2.008	1.643	1.285	1.092	1.666	1.491
Q92990	Glomulin OS=Homo sapiens GN=GLMN PE=1 SV=2 - [GLMN_HU	1.338	1.114	1.645	1.276	1.383	0.948	1.207	1.131
A8K4Z6	cDNA FLJ76180 OS=Homo sapiens PE=2 SV=1 - [A8K4Z6_HUMA	1.364	1.517	1.000	0.826	1.298	1.343	1.378	1.355
Q96T17	MAP7 domain-containing protein 2 OS=Homo sapiens GN=MAP7I	1.387	1.554	1.663	1.267	1.025	1.235	1.808	1.779
Q13287	N-myc-interactor OS=Homo sapiens GN=NMI PE=1 SV=2 - [NMI_	1.014	0.779	1.082	0.742	0.972	1.005	0.939	0.684
Q93099	Homogentisate 1,2-dioxygenase OS=Homo sapiens GN=HGD PE=	1.384	1.430	1.497	1.200	1.360	0.977	1.279	1.300
A0A087WTY1	DNA-directed RNA polymerases I and III subunit RPAC2 OS=Hom	1.013	1.416	1.081	0.485	1.172	1.333	1.100	0.829
Q5VY60	HAUS augmin-like complex subunit 6 OS=Homo sapiens GN=HAU	1.395	1.101	1.306	1.049	1.300	1.152	1.342	1.140
B0AZV9	Trafficking protein particle complex 6B, isoform CRA_a OS=Homo	1.816	1.193	1.585	1.259	1.490	1.135	1.603	1.554
Q6ZRV2	Protein FAM83H OS=Homo sapiens GN=FAM83H PE=1 SV=3 - [F	1.480	1.768	1.099	0.894	1.110	1.186	1.336	0.954
B4DSE0	cDNA FLJ60851, highly similar to Ephrin type-B receptor 2 (EC 2.7	1.448	1.605	1.132	1.158	1.437	1.185	1.363	1.394
O15230	Laminin subunit alpha-5 OS=Homo sapiens GN=LAMA5 PE=1 SV=	1.247	1.101	0.929	0.796	1.154	1.059	1.142	0.821
B4E1N1	Armadillo repeat-containing protein 6 OS=Homo sapiens GN=ARI	1.713	1.491	2.050	2.053	1.553	1.175	1.624	1.591
Q5BJH7	Protein YIF1B OS=Homo sapiens GN=YIF1B PE=1 SV=1 - [YIF1E	2.917	2.775	3.033	2.091	2.471	1.463	2.008	2.350
Q9H2F5	Enhancer of polycomb homolog 1 OS=Homo sapiens GN=EPC1 P	1.140	1.737	0.907	0.746	1.263	1.698	1.748	0.904
Q01658	Protein Dr1 OS=Homo sapiens GN=DR1 PE=1 SV=1 - [NC2B_HU	1.021	0.878	0.984	0.726	0.970	1.136	0.962	1.033
Q12841	Follistatin-related protein 1 OS=Homo sapiens GN=FSTL1 PE=1 S	1.286	1.528	1.475	1.157	1.319	1.756	1.779	1.567
Q9NW82	WD repeat-containing protein 70 OS=Homo sapiens GN=WDR70	1.601	1.330	1.309	1.139	1.435	1.268	1.335	0.979
Q8WVM7	Cohesin subunit SA-1 OS=Homo sapiens GN=STAG1 PE=1 SV=3	1.359	1.683	1.432	1.394	1.389	1.448	1.811	1.646
Q53EU6	Glycerol-3-phosphate acyltransferase 3 OS=Homo sapiens GN=A(	1.295	1.115	0.841	0.789	1.484	1.153	1.469	0.985
Q68CQ7	Glycosyltransferase 8 domain-containing protein 1 OS=Homo sap	1.122	1.084	0.907	0.854	0.983	1.072	1.069	0.880
H0YCU9	Transgelin (Fragment) OS=Homo sapiens GN=TAGLN PE=1 SV=1				0.483	1.175	0.939	0.633	0.774
P50148	Guanine nucleotide-binding protein G(q) subunit alpha OS=Homo	1.543	2.047	1.459	1.299	1.220	1.187	1.380	1.311
B7Z2F7	cDNA FLJ54655, highly similar to Heat shock 70 kDa protein 12A	1.240	1.120	1.277	1.025	1.203	1.180	1.138	1.185
Q56A86	AKT3 protein (Fragment) OS=Homo sapiens GN=AKT3 PE=2 SV=	1.366	1.152	4.499	3.447	0.897	0.758	0.920	0.704
J3KSG1	BTB/POZ domain-containing protein KCTD1 (Fragment) OS=Hom	0.684	0.683	0.452		0.974	1.521	0.610	0.610
Q8NEZ2	Vacuolar protein sorting-associated protein 37A OS=Homo sapien	1.155	0.745	0.813	0.732	0.989	0.798	0.886	0.664
Q9H173	Nucleotide exchange factor SIL1 OS=Homo sapiens GN=SIL1 PE=	1.672	1.259	1.303	1.207	1.683	1.239	1.485	1.671
B3KS95	cDNA FLJ35792 fis, clone TESTI2005759, highly similar to Amphc	1.567	0.569	0.311	0.202	1.233	0.696	0.416	
Q5VW32	BRO1 domain-containing protein BROX OS=Homo sapiens GN=BI	0.912	0.588	0.888	0.763	0.919	0.892	0.856	0.707
O95479	GDH/6PGL endoplasmic bifunctional protein OS=Homo sapiens G	1.177	0.801	1.317	1.183	1.204	1.067	1.297	1.314

D3DUR9	Zinc finger protein 384, isoform CRA_a OS=Homo sapiens GN=Zl	1.107	1.350	1.052	1.051	0.934	1.478	1.388	1.135
Q9C0B5	Palmitoyltransferase ZDHHC5 OS=Homo sapiens GN=ZDHHC5 PE	0.904	1.454	0.798	0.859	0.949	1.082	1.010	0.837
Q96AG3	Solute carrier family 25 member 46 OS=Homo sapiens GN=SLC2!	1.752	2.016	1.781	1.813	1.636	1.371	2.109	2.053
Q9Y6X9	MORC family CW-type zinc finger protein 2 OS=Homo sapiens GN	1.268	1.618	1.185	0.825	0.924	1.191	1.346	1.231
P15289	Arylsulfatase A OS=Homo sapiens GN=ARSA PE=1 SV=3 - [ARSA	1.422	0.828	1.077	1.098	1.155	1.063	1.260	1.101
O75376	Nuclear receptor corepressor 1 OS=Homo sapiens GN=NCOR1 PE	1.132	1.334	0.886	0.704	1.032	1.256	1.134	1.053
Q4G0F5	Vacuolar protein sorting-associated protein 26B OS=Homo sapien	1.369	1.418	1.259	1.446	1.153	1.025	1.384	1.030
Q14202	Zinc finger MYM-type protein 3 OS=Homo sapiens GN=ZMYM3 PI	1.151	1.175	0.835	0.731	1.260	1.186	1.306	0.920
Q9BUB7	Transmembrane protein 70, mitochondrial OS=Homo sapiens GN=	1.183	1.417	1.179	1.155	1.054	1.089	1.290	1.194
A0A087WYU2	UPF0585 protein C16orf13 OS=Homo sapiens GN=C16orf13 PE=4	1.089	0.661	1.041	0.868	0.976	0.876	1.035	0.755
Q15545	Transcription initiation factor TFIID subunit 7 OS=Homo sapiens (	1.370	1.414	1.194	0.951	1.358	1.663	1.502	1.372
Q5VTL8	Pre-mRNA-splicing factor 38B OS=Homo sapiens GN=PRPF38B PI	1.186	1.470	1.151	0.884	1.113	1.156	1.147	0.969
Q9H9A5	CCR4-NOT transcription complex subunit 10 OS=Homo sapiens G	2.169	2.363	1.887	2.047	2.044	1.556	2.026	1.820
Q8IWB1	Inositol 1,4,5-trisphosphate receptor-interacting protein OS=Hom	1.221	1.517	0.836	1.041	0.999	1.029	1.053	1.006
Q14181	DNA polymerase alpha subunit B OS=Homo sapiens GN=POLA2 F	1.134	1.030	0.908	0.698	1.141	1.210	0.874	0.813
Q9HAF1	Chromatin modification-related protein MEAF6 OS=Homo sapiens	1.510	1.906	1.066	0.989	1.241	1.309	1.602	1.516
H3BSD1	Methenyltetrahydrofolate synthase domain-containing protein (Fra	1.288	1.445	1.653	1.147	1.256	1.278	1.557	1.173
Q99549	M-phase phosphoprotein 8 OS=Homo sapiens GN=MPHOSPH8 PE	1.128	1.369	0.938	0.948	1.078	1.161	1.260	1.147
F8VSE4	WD repeat domain-containing protein 83 OS=Homo sapiens GN=	0.983	1.594	1.297	1.157	1.427	0.843	1.273	1.216
H0Y360	AMP deaminase 2 (Fragment) OS=Homo sapiens GN=AMPD2 PE=	1.260	0.823	1.215	1.060	1.100	1.049	1.175	1.120
Q92604	Acyl-CoA:lysophosphatidylglycerol acyltransferase 1 OS=Homo sa	1.197	1.426	1.073	1.134	0.932	1.208	1.125	0.909
Q96AJ9	Vesicle transport through interaction with t-SNAREs homolog 1A (	1.156	1.370	1.481	1.256	1.031	1.193	1.409	1.468
B3KQQ7	cDNA PSEC0037 fis, clone NT2RP1000800, highly similar to N-ace	0.941	1.483	1.165	1.178	0.864	1.575	1.745	1.657
B3KP90	cDNA FLJ31447 fis, clone NT2NE2000913, highly similar to Protei	1.482	0.813	1.303	1.027	1.184	0.915	0.783	1.072
A0A024R3K2	RNA pseudouridylate synthase domain containing 4, isoform CRA_	1.189	1.125	1.059	0.821	0.780	1.238	1.005	0.905
B4DMI5	cDNA FLJ56027, highly similar to Glutamate-rich protein 1 OS=Hc	1.432	1.483	0.921	0.764	1.294	1.201	1.246	0.958
E5KQF5	Glucocorticoid receptor OS=Homo sapiens GN=NR3C1 PE=3 SV=	1.284	0.942	0.902	0.660	1.128	1.024	0.797	0.581
B3KQ68	cDNA FLJ32970 fis, clone TESTI2008840, highly similar to Proteir	2.687	2.527	2.329	2.241	1.807	1.444	2.452	2.211
P20933	N(4)-(beta-N-acetylglucosaminyl)-L-asparaginase OS=Homo sapie	2.249	1.688	1.750	2.049	1.587	0.954	2.096	2.104
Q15814	Tubulin-specific chaperone C OS=Homo sapiens GN=TBCC PE=1	1.208	1.024	1.347	0.821	1.007	1.033	1.113	0.995
Q9NPH2	Inositol-3-phosphate synthase 1 OS=Homo sapiens GN=ISYNA1	1.438	0.818	1.485	1.072	1.232	0.863	1.261	1.114
Q7L3T8	Probable proline--tRNA ligase, mitochondrial OS=Homo sapiens G	1.977	1.799	1.238	0.971	1.056	1.120	1.736	1.629
A8K594	cDNA FLJ77256, highly similar to Homo sapiens SH3-domain binc	1.635	1.232	0.811	0.595	1.446	0.974	1.023	0.793
B4DVR4	cDNA FLJ60912, highly similar to Vinexin OS=Homo sapiens PE=:	1.068	0.785	0.902	0.664	0.962	0.990	0.971	0.676
F8W717	Echinoderm microtubule-associated protein-like 1 OS=Homo sapi	1.185	0.884	1.043	0.827	1.048	0.885	1.042	0.843
Q0VDG4	Secernin-3 OS=Homo sapiens GN=SCRN3 PE=1 SV=1 - [SCRN3_	1.390	1.041	1.603	1.425	1.127	1.379	1.464	1.611
Q8NBF2	NHL repeat-containing protein 2 OS=Homo sapiens GN=NHLRC2	1.722	1.472	1.907	1.579	1.450	1.134	1.479	1.498
I3L413	G1/S-specific cyclin-E1 (Fragment) OS=Homo sapiens GN=CCNE:	1.376	2.602	2.131	1.296	1.544	1.945	2.146	1.778
A6NIH7	Protein unc-119 homolog B OS=Homo sapiens GN=UNC119B PE=	1.758	1.367	1.475	1.590	1.461	1.253	1.512	1.176
Q99836	Myeloid differentiation primary response protein MyD88 OS=Hom	1.306	0.982	1.145	0.976	1.323	0.839	1.065	1.072

Q96FV2	Secernin-2 OS=Homo sapiens GN=SCRN2 PE=1 SV=3 - [SCRN2_	2.644	1.495	2.509	2.960	1.830	1.281	2.076	2.798
P42574	Caspase-3 OS=Homo sapiens GN=CASP3 PE=1 SV=2 - [CASP3_H	1.265	0.892	1.149	1.058	1.288	1.186	1.518	1.303
B2RCJ6	cDNA, FLJ96114, highly similar to Homo sapiens bromodomain ar	1.321	1.372	1.193	1.000	1.205	1.214	1.316	1.149
Q6YHK3	CD109 antigen OS=Homo sapiens GN=CD109 PE=1 SV=2 - [CD1	1.176	1.906	1.038	1.076	1.090	1.018	1.223	0.804
A2ABF8	Histone-lysine N-methyltransferase EHMT2 OS=Homo sapiens GN	1.381	1.422	1.021	0.974	1.361	1.454	1.663	1.640
Q4KMP7	TBC1 domain family member 10B OS=Homo sapiens GN=TBC1D	1.356	0.894	1.163	0.961	1.128	1.019	1.246	1.373
Q9NVM6	DnaJ homolog subfamily C member 17 OS=Homo sapiens GN=DI	1.070	0.973	1.014	0.626	1.091	1.252	1.053	0.772
H7C3U7	LisH domain-containing protein ARMC9 (Fragment) OS=Homo sa	1.462	1.066	1.063	1.381	0.877	1.098	1.602	1.458
Q8WV60	Pentatricopeptide repeat-containing protein 2, mitochondrial OS=	1.452	1.446	1.098	1.020	1.117	1.077	1.195	1.145
Q9Y6W5	Wiskott-Aldrich syndrome protein family member 2 OS=Homo sa	1.267	0.841	0.925	0.676	0.998	0.909	0.799	0.842
Q5THR1	Probable ATP-dependent RNA helicase DHX35 OS=Homo sapiens	1.240	1.279	1.003	0.829	1.160	1.129	1.117	0.837
O14578	Citron Rho-interacting kinase OS=Homo sapiens GN=CIT PE=1 S	1.441	1.053	1.058	0.931	1.362	1.004	1.117	1.189
A8KAH1	cDNA FLJ75839, highly similar to Homo sapiens phosphatidylserir	0.977	1.116	1.128	1.001	0.790	1.163	1.107	0.963
A8K4M5	cDNA FLJ76043, highly similar to Homo sapiens cyclin T1 (CCNT1	1.457	1.777	1.234	0.836	1.431	1.352	1.515	1.302
Q15276	Rab GTPase-binding effector protein 1 OS=Homo sapiens GN=RA	1.332	1.107	1.210	1.193	1.087	1.126	1.178	1.285
Q8WZ82	Ovarian cancer-associated gene 2 protein OS=Homo sapiens GN=	1.306	1.014	1.311	1.157	0.930	1.020	1.135	1.091
B4DS44	cDNA FLJ55499, highly similar to Vacuolar protein sorting protein	1.711	1.132	1.523	1.162	1.252	1.277	1.393	1.511
E5KRP6	Spastin OS=Homo sapiens GN=SPAST PE=3 SV=1 - [E5KRP6_HU	1.117	0.836	0.829	0.741	0.931	1.028	0.863	0.764
Q15542	Transcription initiation factor TFIID subunit 5 OS=Homo sapiens (	1.289	1.578	1.438	1.528	1.359	1.365	1.559	1.608
Q9HBL8	NmrA-like family domain-containing protein 1 OS=Homo sapiens	1.157	0.821	1.509	1.021	1.089	1.075	1.156	1.107
Q68CP9	AT-rich interactive domain-containing protein 2 OS=Homo sapiens	1.257	1.362	0.960	0.858	1.254	1.260	1.301	1.046
Q9ULR3	Protein phosphatase 1H OS=Homo sapiens GN=PPM1H PE=1 SV	1.192	0.841	1.024	0.963	1.048	1.033	1.054	0.705
Q86U44	N6-adenosine-methyltransferase 70 kDa subunit OS=Homo sapie	1.115	1.006	0.913	0.634	1.154	1.155	1.177	0.733
Q53FP0	Pyridoxine 5'-phosphate oxidase variant (Fragment) OS=Homo sa	1.106	1.018	1.257	1.266	1.092	1.007	1.302	1.210
Q9NWY4	UPF0609 protein C4orf27 OS=Homo sapiens GN=C4orf27 PE=1 S	1.228	1.297	1.454	0.969	1.148	1.241	1.370	1.161
C9JRJ5	LIM domain-containing protein 1 OS=Homo sapiens GN=LIMD1 F	1.328	0.910	1.080	0.864	1.174	1.076	1.041	0.966
Q9UNS1	Protein timeless homolog OS=Homo sapiens GN=TIMELESS PE=1	1.375	1.355	1.165	0.825	1.437	1.171	1.312	1.016
O95425	Supervillin OS=Homo sapiens GN=SVIL PE=1 SV=2 - [SVIL_HUM	1.310	1.619	1.254	0.902	1.154	1.180	1.230	0.989
Q99487	Platelet-activating factor acetylhydrolase 2, cytoplasmic OS=Hom	1.375	1.270	1.428	1.231	1.163	1.134	1.116	1.174
K7EK00	Protein FAM210A (Fragment) OS=Homo sapiens GN=FAM210A PI	1.006	1.382	1.131	1.170	0.919	1.127	1.053	1.110
Q5W0B1	RING finger protein 219 OS=Homo sapiens GN=RNF219 PE=1 SV	1.612	1.594	1.677	1.121	1.299	1.452	1.380	1.087
A6PWM2	Cysteine-rich with EGF-like domain protein 2 (Fragment) OS=Hor	0.404	0.393	0.492	0.500	0.927	1.315	0.866	0.630
B0YJ81	Very-long-chain (3R)-3-hydroxyacyl-CoA dehydratase 1 OS=Homo	1.475	1.192	1.021	0.940	1.224	1.281	1.431	1.224
Q96BW9	Phosphatidate cytidylyltransferase, mitochondrial OS=Homo sapie	1.440	1.643	1.281	1.107	1.059	1.093	1.365	1.301
P48634	Protein PRRC2A OS=Homo sapiens GN=PRRC2A PE=1 SV=3 - [P	1.188	1.800	0.982	1.082	1.123	1.292	1.215	1.003
Q7Z3K3	Pogo transposable element with ZNF domain OS=Homo sapiens (	1.156	1.333	1.055	0.933	1.028	1.209	1.173	0.963
Q5T5I6	Golgi phosphoprotein 3-like (Fragment) OS=Homo sapiens GN=G	1.486	1.365	1.171	1.319	1.494	1.383	1.528	1.224
Q8WUU5	GATA zinc finger domain-containing protein 1 OS=Homo sapiens	1.740	2.092	1.105	0.981	1.353	1.768	1.587	1.047
A0A024R2G2	Fanconi anemia, complementation group D2, isoform CRA_b OS=	1.713	1.908	2.263	2.109	1.693	1.397	2.148	2.987
Q969Z3	Mitochondrial amidoxime reducing component 2 OS=Homo sapier	2.492	3.137	2.758	2.734	2.023	1.757	2.769	2.794

Q1JUQ5	Peptidyl-prolyl cis-trans isomerase OS=Homo sapiens GN=FKBP1	1.132	0.825	1.233	0.888	1.098	1.008	0.965	0.841
D3VVD6	Ataxin 3 variant ref (Fragment) OS=Homo sapiens GN=ATXN3 PE	2.943	2.152	2.488	1.926	2.633	1.261	2.153	2.107
P98173	Protein FAM3A OS=Homo sapiens GN=FAM3A PE=1 SV=2 - [FAM	1.082	1.112	0.934	0.878	0.927	1.213	1.209	0.945
Q5T447	E3 ubiquitin-protein ligase HECTD3 OS=Homo sapiens GN=HECT	1.414	1.035	0.797	1.009	1.266	1.148	1.070	0.939
Q9NVH6	Trimethyllysine dioxygenase, mitochondrial OS=Homo sapiens GN	0.992	1.283	1.160	1.105	0.839	1.193	1.375	1.268
Q9NQS1	Cell death regulator Aven OS=Homo sapiens GN=AVEN PE=1 SV=	1.719	1.565	1.658	1.289	1.428	1.196	1.623	1.552
B3KRY3	cDNA FLJ35079 fis, clone PLACE6005283, highly similar to Lysoso	1.505	1.176	1.313	1.203	1.467	1.137	1.601	1.500
Q9Y4B5	Microtubule cross-linking factor 1 OS=Homo sapiens GN=MTCL1	1.332	1.234	1.159	1.040	1.193	1.122	1.155	1.021
O60502	Bifunctional protein NCOAT OS=Homo sapiens GN=MGEA5 PE=1	1.321	0.986	1.215	0.928	1.351	0.985	1.136	1.088
B4DG30	cDNA FLJ58802, highly similar to Muskelin OS=Homo sapiens PE=	1.623	1.669	1.721	1.253	1.526	1.651	1.691	1.222
Q9NXA8	NAD-dependent protein deacylase sirtuin-5, mitochondrial OS=Ho	1.063	1.185	0.926	0.923	0.890	0.845	0.921	0.917
Q96RL1	BRCA1-A complex subunit RAP80 OS=Homo sapiens GN=UIMC1	1.753	1.596	1.247	0.869	1.640	1.231	1.400	1.386
Q8WWQ0	PH-interacting protein OS=Homo sapiens GN=PHIP PE=1 SV=2 -	1.828	2.164	1.450	1.292	1.518	1.180	1.721	1.650
P18827	Syndecan-1 OS=Homo sapiens GN=SDC1 PE=1 SV=3 - [SDC1_H	1.480	2.098	1.785	1.617	1.166	1.203	1.745	1.635
O43502	DNA repair protein RAD51 homolog 3 OS=Homo sapiens GN=RAI	1.718	1.526	1.617	1.120	1.659	1.308	1.764	1.313
A0JLT2	Mediator of RNA polymerase II transcription subunit 19 OS=Hom	1.826	1.776	1.809	1.580	1.611	1.628	1.306	1.627
B3KV69	cDNA FLJ16196 fis, clone COLON2006417, highly similar to Homo	1.429	1.160	1.366	0.981	1.266	1.039	1.216	1.328
Q9H0X9	Oxysterol-binding protein-related protein 5 OS=Homo sapiens GN	1.433	1.420	1.081	1.096	0.847	1.069	1.012	1.582
O14880	Microsomal glutathione S-transferase 3 OS=Homo sapiens GN=M	1.431	1.707	1.245	1.151	1.656	1.553	1.427	1.518
Q4J6C6	Prolyl endopeptidase-like OS=Homo sapiens GN=PREPL PE=1 SV	1.132	0.992	1.472	1.126	0.849	1.351	1.202	1.353
Q9HCE5	N6-adenosine-methyltransferase subunit METTL14 OS=Homo sap	1.217	1.259	1.071	0.775	1.165	1.164	1.128	0.835
B4DS52	cDNA FLJ56237, highly similar to Homo sapiens thioredoxin doma	2.294	2.482	2.176	1.493	1.814	1.723	2.440	1.688
A0A024R6D1	NIMA (Never in mitosis gene a)-related kinase 9, isoform CRA_a (	1.337	1.158	1.468	1.117	1.243	1.118	1.326	1.285
B2RAI2	cDNA, FLJ94931, highly similar to Homo sapiens zinc finger prote	1.015	1.372	1.064	0.893	0.898	1.203	1.198	1.041
B4E1V1	Anoctamin (Fragment) OS=Homo sapiens PE=2 SV=1 - [B4E1V1_	1.256	1.551	1.207	1.221	1.113	1.212	1.211	1.244
A0A087WTT6	40S ribosomal protein S29 OS=Homo sapiens GN=RPS29 PE=4 S	6.826	7.065	6.278	4.102	2.569	1.622	2.738	2.989
Q9UHD2	Serine/threonine-protein kinase TBK1 OS=Homo sapiens GN=TBI	1.255	0.872	1.106	0.856	1.094	0.967	1.127	0.832
Q5TEU4	NADH dehydrogenase [ubiquinone] 1 alpha subcomplex assembly	1.043	1.191	1.038	1.124	0.893	1.142	0.965	1.067
Q7Z4V5	Hepatoma-derived growth factor-related protein 2 OS=Homo sap	1.592	1.395	1.321	1.221	1.292	1.159	1.339	1.191
Q86WJ1	Chromodomain-helicase-DNA-binding protein 1-like OS=Homo sa	1.368	1.072	1.047	0.905	1.172	0.987	0.983	0.921
O95870	Abhydrolase domain-containing protein 16A OS=Homo sapiens G	1.414	1.433	1.214	1.284	1.074	1.157	1.393	1.022
A0A087WSZ7	Dihydropyrimidinase-related protein 3 OS=Homo sapiens GN=DP	1.094	0.501	0.767	0.568	0.986	0.808	0.681	0.684
Q6KB66	Keratin, type II cytoskeletal 80 OS=Homo sapiens GN=KRT80 PE	2.015	1.749	1.082	0.968	1.777	1.154	1.348	0.923
Q5TDH0	Protein DDI1 homolog 2 OS=Homo sapiens GN=DDI2 PE=1 SV=	1.120	0.903	1.151	0.862	1.273	1.116	0.971	0.918
Q658Y4	Protein FAM91A1 OS=Homo sapiens GN=FAM91A1 PE=1 SV=3 -	1.247	1.334	1.127	0.947	1.318	1.196	1.262	1.122
Q9H3F6	BTB/POZ domain-containing adapter for CUL3-mediated RhoA de	2.874	1.596	2.003	1.452	1.732	1.618	1.733	1.293
A0A024RDA1	Exocyst complex component 1, isoform CRA_a OS=Homo sapiens	1.175	1.163	1.283	1.090	1.177	1.031	1.150	1.005
Q96ES7	SAGA-associated factor 29 homolog OS=Homo sapiens GN=CCDC	1.275	1.603	1.285	0.953	1.136	1.610	1.457	1.185
B4DQA5	cDNA FLJ58037, highly similar to Mus musculus RAB GTPase activ	1.392	1.360	1.192	1.396	1.336	1.301	1.463	1.532
Q6P9B9	Integrator complex subunit 5 OS=Homo sapiens GN=INTS5 PE=1	2.038	2.076	2.036	1.737	2.082	1.503	1.916	1.828

A0AV58	Striatin, calmodulin binding protein 3 OS=Homo sapiens GN=STR	1.436	1.035	1.285	0.877	1.408	1.142	1.289	1.040
Q2KS10	Protein X OS=Human adenovirus C serotype 5 GN=L2 PE=4 SV=	0.645	0.729			0.712	1.178	1.221	1.334
Q96KC8	DnaJ homolog subfamily C member 1 OS=Homo sapiens GN=DN	1.197	0.753	0.459	0.634	0.998	0.823	0.625	0.478
P51531	Probable global transcription activator SNF2L2 OS=Homo sapiens	1.146	1.369	0.973	0.849	1.251	1.158	1.356	0.961
B1AH87	Putative peripheral benzodiazepine receptor-related protein (Frag	1.883	1.921	1.633	1.735	1.341	1.124	2.004	1.590
Q9NQX4	Unconventional myosin-Vc OS=Homo sapiens GN=MYO5C PE=1 S	1.617	1.325	1.465	1.351	1.503	1.311	1.348	1.499
E7ES96	Presenilin OS=Homo sapiens GN=PSEN1 PE=1 SV=1 - [E7ES96_	1.903	2.666	1.998	2.959	2.050	1.152	1.979	2.233
Q5VZL5	Zinc finger MYM-type protein 4 OS=Homo sapiens GN=ZMYM4 PI	1.443	1.909	1.250	1.093	1.426	1.365	1.575	1.298
Q147X3	N-alpha-acetyltransferase 30 OS=Homo sapiens GN=NAA30 PE=1	1.709	1.544	1.665	0.990	1.290	1.471	1.478	1.431
C9J8T0	Selenocysteine-specific elongation factor OS=Homo sapiens GN=	1.643	1.401	1.529	0.964	1.318	1.070	1.593	1.275
Q9BU89	Deoxyhypusine hydroxylase OS=Homo sapiens GN=DOHH PE=1	1.387	1.119	1.605	1.442	1.181	1.215	1.674	1.341
Q9UJ68	Mitochondrial peptide methionine sulfoxide reductase OS=Homo s	1.238	1.073	1.182	0.972	1.081	1.142	1.295	1.210
B4DWW6	cDNA FLJ53934, highly similar to Homo sapiens RNA pseudouridy	1.802	1.573	1.441	1.046	1.588	1.150	1.243	1.232
B4DH44	cDNA FLJ52538, highly similar to Dual specificity mitogen-activate					0.246	1.038		
Q9Y5Y5	Peroxisomal membrane protein PEX16 OS=Homo sapiens GN=PE	0.724	1.049	0.753	0.798	0.991	1.296	0.976	0.970
B7Z4S8	cDNA FLJ53066, highly similar to Legumain (EC 3.4.22.34) OS=H	1.116	1.233	1.130	1.000	1.185	1.058	1.092	1.134
Q9NPQ8	Synembryn-A OS=Homo sapiens GN=RIC8A PE=1 SV=3 - [RIC8A	1.131	1.466	1.146	1.069	1.123	1.141	1.294	0.916
Q9Y232	Chromodomain Y-like protein OS=Homo sapiens GN=CDYL PE=1	1.276	1.325	0.770	0.485	1.222	1.110	0.930	0.810
B4DEK8	Tetraspanin OS=Homo sapiens PE=2 SV=1 - [B4DEK8_HUMAN]	1.239	1.150	0.838	0.709	1.502	1.159	1.288	0.993
K7EJB8	Protein phosphatase 1 regulatory subunit 14A OS=Homo sapiens	0.944	0.524	0.651	0.617	0.859	0.795	0.948	0.674
B4DQE4	cDNA FLJ51763, highly similar to Orphan nuclear receptor EAR-2	1.034	0.995	1.032	0.837	0.751	0.951	1.100	1.122
Q12929	Epidermal growth factor receptor kinase substrate 8 OS=Homo sa	1.140	1.164	1.031	0.650	1.132	0.789	0.939	0.696
P09884	DNA polymerase alpha catalytic subunit OS=Homo sapiens GN=P	1.606	1.424	1.310	0.878	1.378	1.213	1.390	1.063
Q12872	Splicing factor, suppressor of white-apricot homolog OS=Homo sa	1.931	2.341	2.050	1.498	1.703	1.553	2.194	2.161
Q8NEN9	PDZ domain-containing protein 8 OS=Homo sapiens GN=PDZD8	1.240	1.075	0.982	0.937	1.033	1.043	1.185	0.917
Q5JTZ9	Alanine--tRNA ligase, mitochondrial OS=Homo sapiens GN=AARS	0.953	1.047	1.085	0.912	0.859	1.111	1.169	0.933
Q0JRZ9	FCH domain only protein 2 OS=Homo sapiens GN=FCHO2 PE=1	1.600	1.231	1.630	1.309	1.465	1.217	1.617	1.332
Q9BTE6	Alanyl-tRNA editing protein Aarsd1 OS=Homo sapiens GN=AARSD	1.300	0.861	1.156	0.931	1.107	0.905	1.091	0.971
O95210	Starch-binding domain-containing protein 1 OS=Homo sapiens GI	2.236	2.115	1.800	1.654	1.629	1.383	1.601	1.557
Q66LE6	Serine/threonine-protein phosphatase 2A 55 kDa regulatory subu	1.052	0.726	0.877	0.644	0.934	1.163	1.058	1.084
B4DVF9	cDNA FLJ54388, highly similar to Tax1-binding protein 1 OS=Hom	1.597	1.330	1.034	0.690	1.741	1.324	1.321	0.928
O14966	Ras-related protein Rab-7L1 OS=Homo sapiens GN=RAB29 PE=1	1.563	1.659	1.596	1.532	1.421	1.398	1.836	1.518
B2R9B8	cDNA, FLJ94320 OS=Homo sapiens PE=2 SV=1 - [B2R9B8_HUM	2.457	2.951	3.434	1.776	2.339	1.244	2.080	2.423
B3KMF1	cDNA FLJ10840 fis, clone NT2RP4001315, highly similar to Rab5	1.844	1.517	1.933	1.549	1.493	1.191	1.681	1.401
Q6NUL1	Exostoses (Multiple) 2 OS=Homo sapiens GN=EXT2 PE=2 SV=1 -	1.426	1.531	1.158	0.853	1.180	1.414	1.699	1.156
Q02083	N-acylethanolamine-hydrolyzing acid amidase OS=Homo sapiens	2.024	1.773	2.187	1.808	1.866	1.277	2.227	1.952
Q709C8	Vacuolar protein sorting-associated protein 13C OS=Homo sapien	1.577	1.192	1.469	1.098	1.347	1.027	1.455	1.202
B3KNJ3	cDNA FLJ14684 fis, clone NT2RP2004933, highly similar to Death	2.045	1.787	1.465	1.148	1.550	1.302	1.456	1.247
B4DLE4	cDNA FLJ54419, highly similar to Synaptopodin-2 OS=Homo sapi	1.364	1.296	0.785	0.612	1.020	1.161	0.973	0.628
B4DDM0	Ribosomal protein S6 kinase OS=Homo sapiens PE=2 SV=1 - [B4	1.733	1.176	1.281	0.958	1.018	0.971	1.139	0.970

B4DNM0	cDNA FLJ60738, highly similar to RNA polymerase-associated pro	1.426	1.456	1.140	0.875	1.363	1.239	1.321	1.037
Q8NAV1	Pre-mRNA-splicing factor 38A OS=Homo sapiens GN=PRPF38A Pl	2.658	3.359	2.401	2.141	2.553	1.795	2.532	2.341
Q86U86	Protein polybromo-1 OS=Homo sapiens GN=PBRM1 PE=1 SV=1 -	1.211	1.494	1.205	1.167	1.163	1.299	1.383	1.308
X5D7P8	Serine threonine kinase 39 isoform D (Fragment) OS=Homo sapie	1.729	1.161	1.658	1.198	1.339	1.098	1.372	1.475
Q13057	Bifunctional coenzyme A synthase OS=Homo sapiens GN=COASY	1.209	1.112	1.518	1.107	1.379	1.055	1.439	1.192
B1Q2B0	URCC5 OS=Homo sapiens GN=URCC5 PE=2 SV=1 - [B1Q2B0_HU	5.184	4.252	5.523	3.667	1.714	1.618	2.484	3.152
P61619	Protein transport protein Sec61 subunit alpha isoform 1 OS=Hom	2.407	2.554	2.485	2.095	2.122	1.328	2.538	1.878
Q5TA58	Protein argonaute OS=Homo sapiens GN=AGO1 PE=1 SV=1 - [Q	1.210	1.124	1.104	0.792	1.237	1.072	0.895	0.917
Q92576	PHD finger protein 3 OS=Homo sapiens GN=PHF3 PE=1 SV=3 - [	1.009	1.463	1.020	0.772	0.895	1.158	1.124	0.918
A8K1Z3	cDNA FLJ75002, highly similar to Homo sapiens, neural cell expre	1.550	1.318	1.482	1.130	1.413	1.355	1.645	1.150
H0Y485	Insulin-like growth factor-binding protein 3 (Fragment) OS=Homo	1.407	0.611	0.175		1.277	0.593	0.577	
Q9Y3P9	Rab GTPase-activating protein 1 OS=Homo sapiens GN=RABGAP	1.174	0.904	1.107	0.769	1.084	0.818	1.119	0.954
Q5H9S0	Putative uncharacterized protein DKFZp781N1974 OS=Homo sapi	1.260	1.422	1.190	1.068	1.166	1.327	1.571	1.323
Q96IF1	LIM domain-containing protein ajuba OS=Homo sapiens GN=AJU	1.738	0.991	1.053	0.726	1.971	1.193	0.923	0.968
E7EW69	Septin-10 OS=Homo sapiens GN=SEPT10 PE=1 SV=1 - [E7EW69	1.679	0.943	1.278	0.856	1.316	1.212	1.108	1.385
Q9Y680	Peptidyl-prolyl cis-trans isomerase FKBP7 OS=Homo sapiens GN=	2.086	1.502	1.895	2.151	1.850	1.271	2.130	2.135
B3KTL8	cDNA FLJ38476 fis, clone FEBRA2022504, highly similar to YTH d	1.195	1.610	1.180	1.103	1.057	1.133	1.276	1.166
O95671	N-acetylserotonin O-methyltransferase-like protein OS=Homo sap	1.662	1.729	1.697	1.326	1.591	1.204	1.624	1.470
E9PFR3	Serine/threonine-protein phosphatase 2A 56 kDa regulatory subu	1.128	0.795	1.089	0.934	0.958	1.038	1.224	0.862
B4DWW0	cDNA FLJ50721, highly similar to Peroxisomal targeting signal 1 r	2.064		1.192	1.809	0.807	0.952	1.015	1.296
H0Y714	U3 small nucleolar ribonucleoprotein protein IMP4 (Fragment) OS	1.301	1.367	1.118	1.018	1.223	1.346	1.287	1.075
Q6P5R1	TMEM59 protein (Fragment) OS=Homo sapiens GN=TMEM59 PE=	1.513	1.307	1.107	0.843	1.490	1.236	1.263	0.946
Q5T280	Putative methyltransferase C9orf114 OS=Homo sapiens GN=C9or	2.293	1.764	1.985	2.034	1.601	1.463	1.744	2.018
O95363	Phenylalanine--tRNA ligase, mitochondrial OS=Homo sapiens GN=	1.950	1.349	1.493	1.743	1.370	1.244	1.656	1.233
Q6UWP2	Dehydrogenase/reductase SDR family member 11 OS=Homo sap	1.232		1.613	1.171	1.156		1.635	
Q93034	Cullin-5 OS=Homo sapiens GN=CUL5 PE=1 SV=4 - [CUL5_HUMA	1.255	1.701	1.549	1.073	1.367	1.159	1.364	1.177
B4DL02	cDNA FLJ56101, highly similar to SHC-transforming protein 1 OS=	1.223	0.843	1.083	1.023	1.567	1.011	1.029	0.849
B2RCM6	cDNA, FLJ96161 OS=Homo sapiens PE=2 SV=1 - [B2RCM6_HUM	1.113	1.050	1.075	0.723	1.182	1.064	1.098	0.907
Q93074	Mediator of RNA polymerase II transcription subunit 12 OS=Home	1.367	1.294	0.818	0.534	1.033	1.235	1.232	0.827
Q8WX93	Palladin OS=Homo sapiens GN=PALLD PE=1 SV=3 - [PALLD_HUI	1.703	1.058	1.038	0.748	1.473	1.142	1.189	1.016
Q8N5Y8	Mono [ADP-ribose] polymerase PARP16 OS=Homo sapiens GN=P	1.330	2.020	1.653	1.700	1.250	1.911	1.548	1.549
A6NLH6	Protein cornichon homolog 4 OS=Homo sapiens GN=CNIH4 PE=1	1.343	1.274	1.249	1.234	1.385	1.297	1.531	1.057
A8K0M6	cDNA FLJ76697 OS=Homo sapiens PE=2 SV=1 - [A8K0M6_HUMA	1.470	1.952	1.762	1.991	1.366	1.672	1.913	1.892
Q9NQZ5	StAR-related lipid transfer protein 7, mitochondrial OS=Homo sap	1.009	1.216	1.080	0.872	1.179	1.509	1.334	0.994
Q05D32	CTD small phosphatase-like protein 2 OS=Homo sapiens GN=CTI	1.239	1.318	1.082	0.964	1.146	1.214	1.311	1.059
Q13503	Mediator of RNA polymerase II transcription subunit 21 OS=Home	0.803	0.729	0.620	0.540	0.821	1.061	0.843	0.680
P17152	Transmembrane protein 11, mitochondrial OS=Homo sapiens GN=	1.363	1.513	1.235	1.191	1.230	1.075	1.337	1.283
E7EWE8	Filamin-binding LIM protein 1 (Fragment) OS=Homo sapiens GN=	1.732	0.959	1.140	0.863	1.521	0.839	1.253	1.224
Q32MN6	TATA-box-binding protein OS=Homo sapiens GN=TBP PE=2 SV=1	1.228	1.488	1.314	1.252	1.157	1.223	1.618	1.455
O60318	Germinal-center associated nuclear protein OS=Homo sapiens GN	1.409	1.347	1.235	1.280	1.113	1.329	1.439	1.400

A0A087WUI1	RNA-binding motif protein, X-linked 2 OS=Homo sapiens GN=RBI	1.579	2.042	1.354	1.241	1.329	1.200	1.404	1.724
O14920	Inhibitor of nuclear factor kappa-B kinase subunit beta OS=Homo	1.429	0.922	0.814	1.057	1.257	1.018	1.112	0.944
B2RE48	cDNA, FLJ96928, highly similar to Homo sapiens candidate tumor	1.167	1.199	1.050	0.947	0.969	1.207	1.243	1.163
B4DZH5	cDNA FLJ55753, highly similar to Transcription factor 12 OS=Hom	1.199	1.599	1.309	0.913	1.258	1.330	1.553	1.604
Q6PGP7	Tetratricopeptide repeat protein 37 OS=Homo sapiens GN=TTC37	1.260	1.066	1.299	0.982	1.119	1.075	1.161	1.022
A0A024RAC6	Transcription elongation factor B (SIII), polypeptide 3 (110kDa, e	1.275	1.752	1.103	0.863	1.046	1.301	1.064	0.891
Q8ND87	Putative uncharacterized protein DKFZp434K0835 OS=Homo sapi	1.785	2.334	3.855	3.648	1.247	1.891	3.107	4.148
Q08AF3	Schlafen family member 5 OS=Homo sapiens GN=SLFN5 PE=1 S	1.134	1.087	1.150	0.772	1.078	0.925	0.896	0.764
Q9NZV1	Cysteine-rich motor neuron 1 protein OS=Homo sapiens GN=CRI	1.582	0.947	0.387	0.349	1.962	0.787	0.788	0.602
K7EIU8	Mothers against decapentaplegic homolog OS=Homo sapiens GN	1.677	1.396	1.642	1.388	1.664	1.256	1.937	1.969
Q6PCE3	Glucose 1,6-bisphosphate synthase OS=Homo sapiens GN=PGM2	0.997	0.928	1.279	0.830	1.406	1.049	1.250	0.958
A0A087WUD3	Oligosaccharyltransferase complex subunit OSTC OS=Homo sapie	1.830	2.058	1.495	1.557	1.580	1.078	1.748	1.505
P48059	LIM and senescent cell antigen-like-containing domain protein 1 (	1.550	1.265	1.408	1.066	1.098	1.005	1.194	1.256
P19838	Nuclear factor NF-kappa-B p105 subunit OS=Homo sapiens GN=I	1.638	1.450	1.646	1.164	1.508	0.984	1.335	1.121
B4DP56	cDNA FLJ52237, highly similar to Creatine kinase B-type (EC 2.7.3	0.574	1.143	1.595	1.365	0.575	1.289	1.594	1.086
K7ELY2	Syntaxin-10 (Fragment) OS=Homo sapiens GN=STX10 PE=1 SV=	1.336	1.465	1.088	0.984	1.497	1.432	1.488	1.307
Q0IJ49	PKMYT1 protein (Fragment) OS=Homo sapiens GN=PKMYT1 PE=	0.729	1.068	0.709	0.546	0.607	1.466	1.055	0.698
Q96A73	Putative monooxygenase p33MONOX OS=Homo sapiens GN=KIA	1.205	1.187	1.120	0.920	1.008	1.093	1.236	1.120
A0A087WXZ3	YTH domain-containing family protein 2 OS=Homo sapiens GN=Y	1.709	2.367	1.778	1.025	1.360	1.219	1.548	2.107
Q9ULG6	Cell cycle progression protein 1 OS=Homo sapiens GN=CCPG1 PE	2.310	3.015	1.691	0.855	2.534	2.153	2.043	1.530
Q9BY89	Uncharacterized protein KIAA1671 OS=Homo sapiens GN=KIAA1	1.265	1.683	1.210	0.939	1.070	1.431	1.410	1.200
Q96S59	Ran-binding protein 9 OS=Homo sapiens GN=RANBP9 PE=1 SV=	1.040	0.814	0.851	0.673	0.905	1.150	0.840	0.574
A0A024RC47	Zinc finger protein 24 (KOX 17), isoform CRA_b OS=Homo sapier	1.460	2.030	1.517	1.116	1.328	1.443	1.751	1.624
B3KPZ7	cDNA FLJ32517 fis, clone SMINT1000117, highly similar to Pyruva	1.441	1.309	1.032	1.192	1.138	1.072	1.136	1.194
Q9HBU6	Ethanolamine kinase 1 OS=Homo sapiens GN=ETNK1 PE=1 SV=	1.393	1.051	1.055	1.345	1.410	0.933	1.078	1.082
Q9UJY4	ADP-ribosylation factor-binding protein GGA2 OS=Homo sapiens	2.291	1.512	2.283	1.684	1.570	1.174	1.927	1.586
B3KN55	cDNA FLJ13636 fis, clone PLACE1011160, highly similar to RING f	1.259	1.219	1.193	1.270	1.131	0.985	1.167	0.948
O14976	Cyclin-G-associated kinase OS=Homo sapiens GN=GAK PE=1 SV=	1.375	0.941	1.174	0.935	1.208	1.113	1.271	1.126
O75175	CCR4-NOT transcription complex subunit 3 OS=Homo sapiens GN	1.498	1.507	1.416	1.030	1.433	1.156	1.374	1.210
B2RDR2	cDNA, FLJ96730, highly similar to Homo sapiens sorting nexin 15	1.465	0.877	1.006	0.917	1.268	1.240	1.284	1.133
A8K0D2	cDNA FLJ77740, highly similar to Homo sapiens 7-dehydrocholest	2.094	1.848	1.792	1.914	1.660	1.496	2.046	1.373
Q8WXH0	Nesprin-2 OS=Homo sapiens GN=SYNE2 PE=1 SV=3 - [SYNE2_H	2.445	2.087	1.886	1.897	2.267	1.411	2.132	2.087
Q7L4I2	Arginine/serine-rich coiled-coil protein 2 OS=Homo sapiens GN=F	1.112	1.358	1.226	0.830	1.283	1.289	1.089	0.853
Q13608	Peroxisome assembly factor 2 OS=Homo sapiens GN=PEX6 PE=1	1.387	1.416	1.352	1.443	1.423	1.165	1.467	1.439
U3KPY1	DNA-directed RNA polymerases I, II, and III subunit RPABC2 (Fra	1.128	1.256	1.099	0.972	1.089	1.329	1.164	1.173
Q8NDT2	Putative RNA-binding protein 15B OS=Homo sapiens GN=RBM15I	1.358	1.746	1.369	1.016	1.303	1.419	1.535	1.154
Q9BVS5	tRNA (adenine(58)-N(1))-methyltransferase, mitochondrial OS=H	1.168	1.327	1.108	1.066	1.041	1.154	1.214	1.186
B3KNJ9	cDNA FLJ14720 fis, clone NT2RP3001495, highly similar to WW d	1.291	1.254	1.297	1.440	1.128	1.156	1.471	1.577
Q8IUI8	Cytokine receptor-like factor 3 OS=Homo sapiens GN=CRLF3 PE=	2.146	1.654	1.973	1.747	2.027	1.692	1.776	2.013
Q9Y371	Endophilin-B1 OS=Homo sapiens GN=SH3GLB1 PE=1 SV=1 - [SH	1.293	0.916	1.367	0.999	1.203	1.030	1.236	1.104

Q7LG56	Ribonucleoside-diphosphate reductase subunit M2 B OS=Homo sa	1.623	1.100	1.564	1.223	1.395	0.992	1.481	1.448
G3XAN4	Translocating chain-associated membrane protein 1 OS=Homo sa	4.124	4.821	4.073	3.689	2.759	1.622	3.800	3.392
P84157	Matrix-remodeling-associated protein 7 OS=Homo sapiens GN=M	2.859	3.309	2.869	2.744	2.498	1.274	3.028	3.340
A0A087WTN3	Selenoprotein T OS=Homo sapiens GN=SELT PE=4 SV=1 - [A0A0	2.350	3.397	3.272	3.659	1.588	2.549	3.732	3.866
A0A024R2W4	Dystroglycan 1 (Dystrophin-associated glycoprotein 1), isoform C	1.082	2.106	1.447	1.434	0.921	1.256	1.197	0.976
Q9H0U9	Testis-specific Y-encoded-like protein 1 OS=Homo sapiens GN=TS	1.463	1.627	0.985	0.847	1.311	1.758	1.464	1.019
Q86WL1	Hemochromatosis (Fragment) OS=Homo sapiens GN=HFE PE=2	1.011	0.902	0.810	0.825	0.859	1.038	0.882	0.705
Q8N4J0	UPF0586 protein C9orf41 OS=Homo sapiens GN=C9orf41 PE=1 S	1.674	1.451	1.440	0.992	1.547	1.233	1.359	1.182
D6REL5	BRCA1-A complex subunit Abraxas OS=Homo sapiens GN=FAM17	1.411	1.364	1.603	1.374	1.283	1.218	1.278	1.067
Q12830	Nucleosome-remodeling factor subunit BPTF OS=Homo sapiens G	1.496	1.812	1.437	1.170	1.202	1.435	1.710	1.290
E9PHM6	Dystonin OS=Homo sapiens GN=DST PE=1 SV=3 - [E9PHM6_HU	1.934	1.444			1.761	1.545		1.406
Q7KZN9	Cytochrome c oxidase assembly protein COX15 homolog OS=Hon	1.490	1.615	1.271	1.131	0.977	1.072	1.285	1.214
Q5SZE1	Ceramide synthase 2 (Fragment) OS=Homo sapiens GN=CERS2 F	2.054	1.935	1.802	1.602	1.860	1.491	1.968	1.930
Q9NXS2	Glutaminyl-peptide cyclotransferase-like protein OS=Homo sapier	1.002	1.031	0.903	0.602	0.906	1.161	0.981	0.547
B4DQ98	cDNA FLJ55135, highly similar to Di-N-acetylchitobiase (EC 3.2.1.	1.027	0.956	1.109	1.169	1.154	1.109	1.349	1.494
Q658P3	Metalloreductase STEAP3 OS=Homo sapiens GN=STEAP3 PE=1 S	1.108	1.541	1.332	1.564	1.199	1.259	1.692	1.921
Q3SY17	Solute carrier family 25 member 52 OS=Homo sapiens GN=SLC25	1.202	1.485	1.107	1.052	1.013	1.272	1.208	1.043
Q2T9J0	Peroxisomal leader peptide-processing protease OS=Homo sapier	2.451	2.729	2.044	1.197	2.046	1.553	2.530	1.464
Q9H875	PRKR-interacting protein 1 OS=Homo sapiens GN=PRKRIP1 PE=1	1.571	1.365	1.305	0.939	1.399	1.169	1.089	1.119
Q9NU22	Midasin OS=Homo sapiens GN=MDN1 PE=1 SV=2 - [MDN1_HUM	1.395	1.550	1.285	0.993	1.442	1.339	1.410	0.944
Q92989	Polyribonucleotide 5'-hydroxyl-kinase Clp1 OS=Homo sapiens GN	1.463	1.516	1.278	0.910	1.436	1.324	1.298	0.992
H7C5K2	Stromal interaction molecule 2 (Fragment) OS=Homo sapiens GN	1.177	1.503	1.344	1.175	0.990	1.166	1.581	1.080
Q8N129	Protein canopy homolog 4 OS=Homo sapiens GN=CNPY4 PE=2 S	1.651	1.358	1.543	1.575	1.586	1.778	1.254	1.396
B7ZM73	MON2 protein OS=Homo sapiens GN=MON2 PE=2 SV=1 - [B7ZM	1.992	1.237	1.801	1.273	1.699	1.056	1.471	1.426
O75330	Hyaluronan mediated motility receptor OS=Homo sapiens GN=HM	1.118	0.734	0.695	0.404	0.847	0.736	0.938	0.738
D3YTC9	Ankyrin repeat domain-containing protein 54 OS=Homo sapiens G	1.282	0.972	1.081	0.668	1.268	1.218	1.234	0.791
Q9UBL6	Copine-7 OS=Homo sapiens GN=CPNE7 PE=2 SV=1 - [CPNE7_HU	1.928	1.636	1.781	1.561	1.806	1.057	1.816	1.810
Q96T58	Msx2-interacting protein OS=Homo sapiens GN=SPEN PE=1 SV=	1.307	1.940	1.453	1.354	1.249	1.763	1.863	1.730
Q5TBH9	Chromosome 1 open reading frame 131, isoform CRA_a OS=Hom	1.948	2.910	1.835	1.335	1.680	1.631	2.116	1.600
B1AKM8	Phosphatidylserine decarboxylase alpha chain (Fragment) OS=Ho	1.929	1.537	1.165	1.059	1.130	1.275	1.330	1.141
D3DUL8	KIAA1609 protein, isoform CRA_a OS=Homo sapiens GN=KIAA16	1.332	1.080	1.287	1.065	1.372	1.115	1.029	0.971
Q9BX40	Protein LSM14 homolog B OS=Homo sapiens GN=LSM14B PE=1	1.068	1.272	0.867	0.421	1.201	1.219	1.059	1.000
Q9BT30	Alpha-ketoglutarate-dependent dioxygenase alkB homolog 7, mito	1.733	1.438	1.201	1.022	1.440	1.236	1.249	0.965
B4E2M1	cDNA FLJ58478, highly similar to Glypican-6 OS=Homo sapiens P	0.891	1.114	0.767	0.500	1.123	0.790	0.813	0.602
Q9Y6M1	Insulin-like growth factor 2 mRNA-binding protein 2 OS=Homo sa	1.411	1.145	1.119	0.774	1.146	0.972	0.972	1.008
Q8N465	D-2-hydroxyglutarate dehydrogenase, mitochondrial OS=Homo sa	1.562	1.661	1.448	1.524	1.180	1.277	1.594	1.627
O95219	Sorting nexin-4 OS=Homo sapiens GN=SNX4 PE=1 SV=1 - [SNX4	1.139	1.212	1.320	1.039	1.429	1.015	1.277	1.098
Q9BYE7	Polycomb group RING finger protein 6 OS=Homo sapiens GN=PC	0.974	1.451	1.035	0.878	1.032	1.417	1.339	1.159
A2RRC9	IQ motif containing GTPase activating protein 3 OS=Homo sapier	1.492	1.047	1.042	0.711	1.398	1.029	0.979	0.645
Q7Z7L1	Schlafen family member 11 OS=Homo sapiens GN=SLFN11 PE=1	1.518	1.611	1.450	1.279	1.247	1.267	1.464	1.328

Q9H7Z6	Histone acetyltransferase KAT8 OS=Homo sapiens GN=KAT8 PE=	2.178	2.259	1.719	1.227	1.520	1.341	1.925	1.605
Q9BVG9	Phosphatidylserine synthase 2 OS=Homo sapiens GN=PTDSS2 PE	3.500	3.723	3.400	3.244	2.872	1.702	3.499	3.401
Q9H269	Vacuolar protein sorting-associated protein 16 homolog OS=Hom	1.292	1.239	1.297	0.981	1.240	1.081	1.251	1.055
F5GZ90	Denticleless protein homolog OS=Homo sapiens GN=DTL PE=1 S	1.968	4.138	3.226	1.902	1.910	3.412	3.148	2.236
Q8IV50	LysM and putative peptidoglycan-binding domain-containing prote	1.702	1.537	1.735	1.375	1.252	1.397	1.601	1.846
B4DFJ1	cDNA FLJ53728, highly similar to TERF1-interacting nuclear facto	1.733	1.677	1.382	0.716	1.545	1.342	1.612	1.155
B7Z561	Amino acid transporter OS=Homo sapiens PE=2 SV=1 - [B7Z561_	1.365	2.406	1.801	1.788	1.336	1.652	1.853	2.110
B4DET0	cDNA FLJ55900, highly similar to Pre-mRNA-splicing factor ATP-d	1.166	1.241	1.141	0.973	1.184	1.288	1.233	0.982
E9PKJ0	Protein wntless homolog (Fragment) OS=Homo sapiens GN=WLS	1.323	1.583	1.477	1.304	1.118	1.249	1.450	1.313
Q14781	Chromobox protein homolog 2 OS=Homo sapiens GN=CBX2 PE=	1.427	2.741	1.377	1.476	1.063	2.119	2.064	2.011
Q9Y5P6	Mannose-1-phosphate guanyltransferase beta OS=Homo sapiens	1.602	1.219	1.288	1.079	1.448	1.036	1.478	1.410
Q96BQ5	Coiled-coil domain-containing protein 127 OS=Homo sapiens GN=	1.310	1.381	1.427	1.216	1.279	1.379	1.476	1.182
O14684	Prostaglandin E synthase OS=Homo sapiens GN=PTGES PE=1 SV	2.074	1.725	1.677	1.317	1.515	1.309	1.697	1.347
A0A087WY88	Protein jagunal homolog 1 OS=Homo sapiens GN=JAGN1 PE=4 S	1.227	1.029	0.995	1.013	1.330	1.317	1.356	0.925
Q969G5	Protein kinase C delta-binding protein OS=Homo sapiens GN=PR	3.310	3.613	2.863	2.532	2.635	1.577	2.464	2.838
H7C1J2	Deoxynucleotidyltransferase terminal-interacting protein 1 (Fragm	1.399	1.858	1.178	1.211	1.200	1.294	1.390	1.120
B4DW31	cDNA FLJ54186, highly similar to Nuclear receptor-binding protei	1.787	1.068	1.279	0.809	1.579	1.140	1.441	1.112
A0A024R6Q8	Bromodomain containing 7, isoform CRA_b OS=Homo sapiens GN	1.583	1.637	1.341	1.300	1.309	1.437	1.648	1.429
Q6VMQ6	Activating transcription factor 7-interacting protein 1 OS=Homo s	1.272	1.602	1.213	0.943	1.283	1.218	1.338	0.942
Q14BN4	Sarcolemmal membrane-associated protein OS=Homo sapiens GN	1.376	1.372	1.371	1.491	1.184	1.315	1.464	1.194
Q15582	Transforming growth factor-beta-induced protein ig-h3 OS=Homo	1.999	1.455	0.982	0.416	1.380	1.454	1.531	0.660
Q8IZ69	tRNA (uracil-5-)-methyltransferase homolog A OS=Homo sapiens	3.644	3.138	3.285	2.608	3.124	1.696	3.740	2.599
Q9H694	Protein bicaudal C homolog 1 OS=Homo sapiens GN=BICC1 PE=	1.227	0.911	0.824	0.611	1.115	0.880	0.760	0.597
Q7Z5Q1	Cytoplasmic polyadenylation element-binding protein 2 OS=Homo	1.430	1.319	0.664	0.761	1.434	1.124	0.837	0.696
E9PMQ6	Heat shock factor protein 1 OS=Homo sapiens GN=HSF1 PE=1 S	1.165	1.416	1.301	1.133	1.276	1.472	1.155	1.061
Q9Y4R8	Telomere length regulation protein TEL2 homolog OS=Homo sapi	1.344	1.081	1.555	0.974	1.293	1.075	1.361	1.218
A8K3J5	cDNA FLJ75054, highly similar to Homo sapiens misato homolog	1.143	0.721	0.991	0.831	1.204	1.004	1.030	0.757
U3KQP8	Mitochondrial calcium uniporter regulator 1 (Fragment) OS=Homo	1.392	1.639	1.418	1.657	1.062	1.166	1.306	1.319
O95834	Echinoderm microtubule-associated protein-like 2 OS=Homo sapi	1.159	0.828	1.388	1.145	1.037	0.912	1.058	1.008
B7Z4H7	cDNA FLJ55340, highly similar to Kelch-like protein 7 OS=Homo s	1.971	1.586	1.260	1.193	2.166	1.227	1.540	1.366
Q8N201	Integrator complex subunit 1 OS=Homo sapiens GN=INTS1 PE=1	1.239	1.255	1.140	0.840	1.162	1.274	1.338	0.999
Q8N556	Actin filament-associated protein 1 OS=Homo sapiens GN=AFAP1	1.466	1.453	0.996	0.736	1.167	1.239	0.989	0.866
O76095	Protein JTB OS=Homo sapiens GN=JTB PE=1 SV=1 - [JTB_HUMA	1.463	1.522	1.038	1.182	1.029	1.392	1.453	1.293
A0A024R2J0	Upstream binding protein 1 (LBP-1a), isoform CRA_b OS=Homo s	1.646	1.529	1.716	1.431	0.705	1.164	1.679	2.107
P30049	ATP synthase subunit delta, mitochondrial OS=Homo sapiens GN=	1.134	1.167	0.953	1.022	0.973	1.089	1.097	1.102
Q96L91	E1A-binding protein p400 OS=Homo sapiens GN=EP400 PE=1 SV	1.069	1.422	0.941	0.623	0.941	1.250	1.134	0.800
Q13137	Calcium-binding and coiled-coil domain-containing protein 2 OS=	1.843	2.105	2.077	1.224	2.171	1.514	2.094	1.668
Q96GA7	Serine dehydratase-like OS=Homo sapiens GN=SDSL PE=1 SV=1	1.099	0.674	1.335	1.047	0.950	0.903	1.027	1.007
F8W9S7	GTPase-activating protein and VPS9 domain-containing protein 1	1.707	1.158	1.635	1.344	1.425	1.035	1.520	1.413
Q8N3P5	Putative uncharacterized protein DKFZp761K058 OS=Homo sapie	1.513	1.450	1.266	1.294	1.139	1.204	1.185	1.020

C0LQF2	Deoxyribonuclease II (Fragment) OS=Homo sapiens PE=2 SV=1	1.408	1.270	1.283	1.227	1.141	1.029	1.283	1.247
Q8WV44	E3 ubiquitin-protein ligase TRIM41 OS=Homo sapiens GN=TRIM4	1.911	1.409	1.093	1.086	1.572	1.502	1.546	1.148
P35610	Sterol O-acyltransferase 1 OS=Homo sapiens GN=SOAT1 PE=1 S	1.640	1.514	1.313	1.115	1.026	1.300	1.101	1.187
A8MQ02	Afadin OS=Homo sapiens GN=MLLT4 PE=1 SV=2 - [A8MQ02_HU	1.406	1.354	1.200	0.853	1.249	0.964	1.136	0.940
Q9NWZ8	Gem-associated protein 8 OS=Homo sapiens GN=GEMIN8 PE=1	1.150	1.000	1.090	0.830	0.926	1.116	0.951	0.972
Q7Z6B0	Coiled-coil domain-containing protein 91 OS=Homo sapiens GN=	1.377	0.882	1.398	1.045	1.316	0.968	1.170	1.041
Q9NQ84	G-protein coupled receptor family C group 5 member C OS=Hom	1.149	1.632	1.204	1.136	1.165	1.292	1.057	1.225
G3V0G1	Golgi reassembly stacking protein 1, 65kDa, isoform CRA_d OS=H		0.449			0.505	1.323	0.378	0.400
Q96HY7	Probable 2-oxoglutarate dehydrogenase E1 component DHKTD1,	1.769	1.700	1.193	1.669	0.897	1.125	1.107	1.617
Q92547	DNA topoisomerase 2-binding protein 1 OS=Homo sapiens GN=T	1.519	1.925	1.651	1.449	1.130	1.570	1.923	1.994
Q2VPK5	Cytoplasmic tRNA 2-thiolation protein 2 OS=Homo sapiens GN=C	1.879	1.064	2.017	1.433	1.336	1.394	1.973	1.663
Q9UBG0	C-type mannose receptor 2 OS=Homo sapiens GN=MRC2 PE=1 S	1.008	1.006	0.858	0.755	0.962	1.123	1.133	0.995
Q08495	Dematin OS=Homo sapiens GN=DMTN PE=1 SV=3 - [DEMA_HUI	1.080	0.760		0.445	0.855	1.406	1.369	0.910
Q8NFC6	Biorientation of chromosomes in cell division protein 1-like 1 OS=	1.208	1.246	0.771	0.548	1.051	1.237	1.088	0.776
A6NKE1	Trafficking protein particle complex subunit 3 OS=Homo sapiens	1.112	0.826	1.176	0.990	1.084	0.750	1.177	1.162
Q13136	Liprin-alpha-1 OS=Homo sapiens GN=PPFIA1 PE=1 SV=1 - [LIPA	1.284	1.019	1.165	0.932	1.076	0.991	1.204	1.002
B4DR71	cDNA FLJ57078, highly similar to Homo sapiens opioid receptor, s	1.263	1.316	1.256	1.173	0.893	1.131	1.114	1.074
B2RXH1	Polyhomeotic homolog 1 (Drosophila) OS=Homo sapiens GN=PH	1.152	1.445	1.263	1.160	1.133	1.072	1.296	1.483
Q9NZ09	Ubiquitin-associated protein 1 OS=Homo sapiens GN=UBAP1 PE=	1.802	1.636	2.033	1.688	1.670	1.302	1.626	1.479
B3KSS4	cDNA FLJ36858 fis, clone ASTRO2015185, highly similar to POLIC	1.213	1.453	1.238	1.239	1.122	1.460	1.403	1.285
Q96CB9	5-methylcytosine rRNA methyltransferase NSUN4 OS=Homo sapie	1.404	1.763	1.698	1.207	1.288	1.142	1.471	1.483
Q8TF01	Arginine/serine-rich protein PNISR OS=Homo sapiens GN=PNISR	1.734	1.590	1.337	0.926	1.694	1.154	1.303	0.983
Q9H9S5	Fukutin-related protein OS=Homo sapiens GN=FKRP PE=1 SV=1	1.354	1.816	1.432	1.488	1.088	1.709	1.690	1.766
Q9P2M7	Cingulin OS=Homo sapiens GN=CGN PE=1 SV=2 - [CING_HUMA	1.586	1.907	1.802	1.637	1.084	1.344	1.757	1.750
Q8IXQ4	GPALPP motifs-containing protein 1 OS=Homo sapiens GN=GPAL	1.246	1.179	1.048	0.827	1.204	1.246	1.218	1.077
B3KPN7	cDNA FLJ32002 fis, clone NT2RP7009394, highly similar to Exocy	1.614	1.145	1.754	1.236	1.153	1.218	1.318	1.289
F8VNT9	CD63 antigen (Fragment) OS=Homo sapiens GN=CD63 PE=1 SV	2.687	2.368	1.793	1.608	2.903	1.712	2.011	1.360
O75886	Signal transducing adapter molecule 2 OS=Homo sapiens GN=ST	1.113	0.940	1.054	0.938	0.981	1.144	1.035	0.889
Q53SV6	Putative uncharacterized protein LOC284996 (Fragment) OS=Hor	3.211	3.346			2.030	1.833	2.388	2.630
U3KQU8	Oxidoreductase NAD-binding domain-containing protein 1 OS=Ho	1.339	1.533	1.140	1.073	1.116	1.245	1.234	1.186
A8K923	cDNA FLJ78690, highly similar to Homo sapiens protein phosphat	1.469	0.771	1.227	0.933	1.270	1.097	1.302	1.120
Q8IYB3	Serine/arginine repetitive matrix protein 1 OS=Homo sapiens GN=	1.387	1.538	1.194	0.876	1.388	1.244	1.245	1.186
Q6MZP7	Protein lin-54 homolog OS=Homo sapiens GN=LIN54 PE=1 SV=3	1.233	1.538	1.794	0.885	1.139	1.299	1.371	1.309
H7C1Z9	Peptidyl-prolyl cis-trans isomerase (Fragment) OS=Homo sapiens	1.247	1.036	1.423	1.429	1.256	1.228	1.401	1.434
Q5SXM8	DNL-type zinc finger protein OS=Homo sapiens GN=DNLZ PE=1 S	0.891	1.080	0.903	0.873	0.590	1.151	0.865	1.134
Q9BQ95	Evolutionarily conserved signaling intermediate in Toll pathway, m	1.167	1.362	1.253	1.128	0.881	1.171	1.181	1.180
B4DMK2	cDNA FLJ57398 OS=Homo sapiens PE=2 SV=1 - [B4DMK2_HUM		0.228			0.206	0.927		0.198
Q5TDG9	DnaJ (Hsp40) homolog, subfamily C, member 16, isoform CRA_a	2.163	1.789	1.795	2.615	1.657	1.190	1.654	1.647
Q9BTA9	WW domain-containing adapter protein with coiled-coil OS=Homo	0.975	0.927	0.794	0.429	0.952	1.335	1.124	0.627
A8K556	cDNA FLJ78217 OS=Homo sapiens PE=2 SV=1 - [A8K556_HUMA	1.890	2.375	1.479	1.490	2.091	1.557	1.737	1.538

Q8TAF3	WD repeat-containing protein 48 OS=Homo sapiens GN=WDR48	1.806	2.028	1.809	1.615	1.723	1.370	1.698	1.936
O60784	Target of Myb protein 1 OS=Homo sapiens GN=TOM1 PE=1 SV=.	1.322	0.956	1.391	1.036	1.972	1.433	1.255	1.282
Q969Y2	tRNA modification GTPase GTPBP3, mitochondrial OS=Homo sapi	1.256	1.198	1.163	1.085	0.891	1.039	1.238	1.119
Q4LE39	AT-rich interactive domain-containing protein 4B OS=Homo sapie	1.442	1.633	0.936	0.658	1.481	1.293	1.373	1.047
Q59GF2	ADAM15 isoform 6a variant (Fragment) OS=Homo sapiens PE=2	1.232	1.195	0.980	0.860	1.014	1.195	1.159	0.906
Q9HBM0	Vezatin OS=Homo sapiens GN=VEZT PE=1 SV=3 - [VEZA_HUMA	1.377	1.711	1.503					
Q8IX90	Spindle and kinetochore-associated protein 3 OS=Homo sapiens (	1.829	1.462	1.759	1.442	1.486	1.254	1.733	1.630
D3DUP2	WNK lysine deficient protein kinase 1, isoform CRA_d OS=Homo s	1.476	1.083	1.362	1.033	1.186	1.077	1.268	1.182
A8K4S9	Signal transducer and activator of transcription OS=Homo sapiens	1.409	0.856	0.945	0.695	1.220	0.897	0.965	0.767
E7EW77	Abl interactor 2 OS=Homo sapiens GN=ABI2 PE=1 SV=1 - [E7EW	1.589	1.162	1.072	0.801	1.152	1.253	1.426	
U3KQN9	Uncharacterized protein (Fragment) OS=Homo sapiens PE=3 SV=	1.414	2.430	1.146	1.190	1.234	1.626	1.550	1.703
O00712	Nuclear factor 1 B-type OS=Homo sapiens GN=NFIB PE=1 SV=2	1.445	1.321	1.562	0.945	1.309	1.695	1.679	1.928
A8K5G6	cDNA FLJ78646, highly similar to Homo sapiens polymerase (DNA	1.304	1.205	0.873	0.949	0.928	1.055	1.113	0.917
Q9NQX3	Gephyrin OS=Homo sapiens GN=GPHN PE=1 SV=1 - [GEPH_HUI	1.726	1.349	2.145	1.545	1.649	0.979	1.431	1.667
Q9Y608	Leucine-rich repeat flightless-interacting protein 2 OS=Homo sapi	1.157	0.760	0.872	0.536	0.850	1.023	0.956	0.738
O15084	Serine/threonine-protein phosphatase 6 regulatory ankyrin repeat	1.300	0.844	1.197	1.013	1.164	1.070	1.158	0.905
Q9H871	Protein RMD5 homolog A OS=Homo sapiens GN=RMND5A PE=1	1.647	1.537	1.510	1.103	1.502	1.547	1.438	1.408
Q6P582	Mitotic-spindle organizing protein 2A OS=Homo sapiens GN=MZT	1.246	1.203	1.196	0.969	1.507	1.334	1.457	1.516
Q5NDL2	EGF domain-specific O-linked N-acetylglucosamine transferase OS	1.681	1.381	1.492	1.541	1.506	1.380	1.596	1.581
Q8NDD5	Putative uncharacterized protein DKFZp586L2318 OS=Homo sapi	1.670	1.654	1.302	1.421	1.744	1.487	1.710	1.798
Q9H4L7	SWI/SNF-related matrix-associated actin-dependent regulator of c	1.694	1.490	1.422	1.030	1.692	1.346	1.309	1.053
Q96GA3	Protein LTV1 homolog OS=Homo sapiens GN=LTV1 PE=1 SV=1 -	1.435	1.247	1.610	1.060	1.255	1.158	1.501	1.361
A8KAD6	cDNA FLJ77616, highly similar to Homo sapiens CWF19-like 2, ce	1.265	1.374	1.149	0.941	1.318	1.337	1.427	1.227
Q13445	Transmembrane emp24 domain-containing protein 1 OS=Homo s	2.583	2.235	2.138	2.112	1.688	1.435	1.958	2.100
B4E257	cDNA FLJ50362, highly similar to Hepatocyte nuclear factor 3-alp	1.141	1.739	1.242	1.015	1.029	1.254	1.639	1.406
P54687	Branched-chain-amino-acid aminotransferase, cytosolic OS=Homo	2.404	1.563	2.400	2.223	1.726	1.139	1.945	1.848
P31644	Gamma-aminobutyric acid receptor subunit alpha-5 OS=Homo sa	1.020	1.162	0.669	0.649	0.906	0.998	0.769	0.669
Q8N6N3	UPF0690 protein C1orf52 OS=Homo sapiens GN=C1orf52 PE=1 S	0.388	0.408	0.404	0.193	0.595	1.043	0.446	0.397
B7ZM87	SRGAP2 protein OS=Homo sapiens GN=SRGAP2 PE=2 SV=1 - [B	1.266	0.835	1.135	0.775	1.180	0.973	1.161	0.827
P62166	Neuronal calcium sensor 1 OS=Homo sapiens GN=NCS1 PE=1 SV	1.104	1.316	1.096	0.920	0.874	0.962	1.097	1.249
Q8IX04	Ubiquitin-conjugating enzyme E2 variant 3 OS=Homo sapiens GN	0.847	0.633	0.899	0.534	0.871	1.080	0.804	0.692
Q7Z422	SUZ domain-containing protein 1 OS=Homo sapiens GN=SZRD1 I	1.518	1.073	1.642	1.144	1.467	1.076	1.270	1.233
Q96H35	Probable RNA-binding protein 18 OS=Homo sapiens GN=RBM18 I	1.773	1.911	1.324	0.839	2.108	1.764	1.490	0.912
Q92968	Peroxisomal membrane protein PEX13 OS=Homo sapiens GN=PE	0.902	1.043	0.983	0.643	0.774	1.090	1.082	0.674
Q4G1C4	ZADH2 protein (Fragment) OS=Homo sapiens GN=ZADH2 PE=2 S	0.970	1.053	0.643	1.001	0.950	0.981	0.949	0.849
Q9H967	WD repeat-containing protein 76 OS=Homo sapiens GN=WDR76	1.273	1.975	1.913	1.464	1.188	1.734	2.185	2.134
Q96RE7	Nucleus accumbens-associated protein 1 OS=Homo sapiens GN=	1.049	1.139	0.970	0.622	1.309	1.405	1.081	0.911
A0A087WUD1	Zinc finger protein 787 OS=Homo sapiens GN=ZNF787 PE=4 SV=	1.364	1.399	1.270	1.113	1.181	1.294	1.278	1.204
P05423	DNA-directed RNA polymerase III subunit RPC4 OS=Homo sapien	1.345	1.616	1.236	0.937	1.273	1.356	1.329	1.377
Q9BUT1	3-hydroxybutyrate dehydrogenase type 2 OS=Homo sapiens GN=	1.307	1.172	1.486	1.425	1.059	1.016	1.579	1.418

Q969R5	Lethal(3)malignant brain tumor-like protein 2 OS=Homo sapiens	1.764	2.837	1.695	1.684	2.023	1.732	2.521	1.726
Q96SU4	Oxysterol-binding protein-related protein 9 OS=Homo sapiens GN	1.395	0.963	1.215	0.946	1.261	1.114	1.230	0.907
B4DSE1	cDNA FLJ55364, highly similar to CRSP complex subunit 6 OS=Ho	1.810	1.777	1.573	1.201	1.796	1.518	1.724	1.486
Q6AI12	Ankyrin repeat domain-containing protein 40 OS=Homo sapiens	1.190	1.322	0.757	0.758	1.053	1.229	1.024	0.955
A8K8P8	Alpha-(1,6)-fucosyltransferase OS=Homo sapiens PE=2 SV=1 - [	1.441	1.471	1.319	1.348	1.373	1.117	1.550	1.506
A0A024QZX9	KCCR13L, isoform CRA_a OS=Homo sapiens GN=LOC221955 PE=	1.136	0.966	1.075	1.037	1.045	1.221	1.186	0.940
P10176	Cytochrome c oxidase subunit 8A, mitochondrial OS=Homo sapie	1.600	1.994	1.657	1.969	1.289	0.917	1.455	1.912
E7EXA6	Chromosome transmission fidelity protein 18 homolog OS=Homo	1.266	1.156	1.942	0.940	0.994	1.096	0.988	1.015
B7Z4V7	cDNA FLJ50087 OS=Homo sapiens PE=2 SV=1 - [B7Z4V7_HUMA	1.304	0.942	0.438	0.315	1.200	1.457	1.432	0.957
A0A087WVD4	Arsenite methyltransferase OS=Homo sapiens GN=AS3MT PE=4	1.180	1.152	1.648	1.295	1.160	1.069	1.507	1.596
O94851	Protein-methionine sulfoxide oxidase MICAL2 OS=Homo sapiens	1.429	1.585	1.121	0.812	1.182	1.092	0.825	0.766
O75054	Immunoglobulin superfamily member 3 OS=Homo sapiens GN=I	1.077	1.393	1.200	1.156	1.057	1.160	1.157	1.016
A0PJM7	IFT74 protein (Fragment) OS=Homo sapiens GN=IFT74 PE=2 SV	1.101	0.892	0.941	0.668	0.979	1.119	0.977	0.935
O95376	E3 ubiquitin-protein ligase ARIH2 OS=Homo sapiens GN=ARIH2	1.014	0.811	1.039	0.873	1.131	1.037	0.976	1.071
Q14997	Proteasome activator complex subunit 4 OS=Homo sapiens GN=	1.540	1.391	1.518	1.116	1.417	1.068	1.375	1.168
B4DHS5	cDNA FLJ54678, highly similar to N(2),N(2)-dimethylguanosine tR	1.103	1.181	0.964	0.703	1.005	1.106	0.992	0.850
O60507	Protein-tyrosine sulfotransferase 1 OS=Homo sapiens GN=TPST1	1.161	1.515	1.265	1.361	1.075	1.415	1.533	1.569
Q6P1Q9	Methyltransferase-like protein 2B OS=Homo sapiens GN=METTL2	1.635	1.460	1.861	1.566	1.425	1.347	1.610	1.457
Q96LT9	RNA-binding protein 40 OS=Homo sapiens GN=RNPC3 PE=1 SV=	1.317	1.658	1.195	0.949	1.414	1.338	1.416	1.139
Q86V21	Acetoacetyl-CoA synthetase OS=Homo sapiens GN=AACS PE=1 S	0.992	0.824	1.357	1.230	0.967	1.084	1.055	1.138
H0YAA3	WD repeat-containing protein 41 (Fragment) OS=Homo sapiens	2.324	1.621	2.015	1.457	1.980	1.516	1.965	1.415
Q9P0W2	SWI/SNF-related matrix-associated actin-dependent regulator of	1.359	1.166	0.954	0.810	1.218	1.045	1.047	1.099
A0A024R2I7	RAD18 homolog (S. cerevisiae), isoform CRA_a OS=Homo sapien	1.735	1.891	1.541	0.947	1.548	1.623	1.488	1.246
Q9H300	Presenilins-associated rhomboid-like protein, mitochondrial OS=H	1.421	1.709	1.353	1.470	1.116	1.178	1.521	1.637
P19022	Cadherin-2 OS=Homo sapiens GN=CDH2 PE=1 SV=4 - [CADH2_	0.823	1.490	0.984	1.114	0.900	1.289	0.918	0.926
Q96EV2	RNA-binding protein 33 OS=Homo sapiens GN=RBM33 PE=1 SV=	1.169	1.194	0.913	0.620	1.132	1.164	1.042	0.750
J3KQA0	Synaptotagmin I, isoform CRA_b OS=Homo sapiens GN=SYT1 PE	1.251	1.887	1.128	1.323	1.300	1.132	1.267	1.161
Q8TBB5	Kelch domain-containing protein 4 OS=Homo sapiens GN=KLHDC	1.705	1.143	1.300	1.021	1.425	1.287	1.190	1.246
Q9BQB6	Vitamin K epoxide reductase complex subunit 1 OS=Homo sapier	1.350	1.540	1.493	1.529	1.172	1.118	1.536	1.473
Q9NRK6	ATP-binding cassette sub-family B member 10, mitochondrial OS=	1.138	1.130	0.769	1.029	1.013	1.043	1.115	1.044
Q6IQ49	Protein SDE2 homolog OS=Homo sapiens GN=SDE2 PE=1 SV=1	1.154	1.047	1.122		1.278	1.438	1.001	0.819
Q96N64	PWWP domain-containing protein 2A OS=Homo sapiens GN=PW	0.718	0.701	0.793	0.413	0.853	1.186	0.920	0.609
A0A024R1H7	Zinc finger protein 278, isoform CRA_b OS=Homo sapiens GN=Z	2.140	2.282	1.474	1.202	1.690	1.475	1.897	1.437
Q96BH1	E3 ubiquitin-protein ligase RNF25 OS=Homo sapiens GN=RNF25	1.504	1.389	1.514	1.092	1.615	1.393	1.268	1.394
O95456	Proteasome assembly chaperone 1 OS=Homo sapiens GN=PSMG	2.208	3.261	3.035	2.081	2.167	1.648	2.376	1.942
O14524	Transmembrane protein 194A OS=Homo sapiens GN=TMEM194A	0.873	0.998	0.759	0.693	0.749	1.115	0.877	0.706
B3KS69	cDNA FLJ35627 fis, clone SPLEN2011139, highly similar to AMSH-	1.443	0.785	1.087	0.829	1.240	1.135	1.183	1.249
Q96T76	MMS19 nucleotide excision repair protein homolog OS=Homo sap	1.677	0.997	1.228	0.887	1.566	1.219	1.080	1.167
Q9P086	Mediator of RNA polymerase II transcription subunit 11 OS=Hom	1.678	1.493	1.199	0.866	1.520	1.194	1.779	1.194
A5YM55	RECQL5 protein OS=Homo sapiens GN=RECQL5 PE=2 SV=1 - [A	1.367	1.973	1.480	1.030	1.373	1.372	1.477	1.164

Q9UMP7	G4 protein OS=Homo sapiens GN=G4 PE=2 SV=1 - [Q9UMP7_HU	1.595	2.208	1.074	1.168	1.201	1.389	1.629	1.547
Q8NDX5	Polyhomeotic-like protein 3 OS=Homo sapiens GN=PHC3 PE=1 S	1.303	1.863	1.438	1.528	1.352	1.401	1.788	1.927
Q63ZY3	KN motif and ankyrin repeat domain-containing protein 2 OS=Ho	1.635	1.505	1.348	0.954	1.245	1.194	1.312	1.048
E9PJM3	F-box only protein 3 OS=Homo sapiens GN=FBXO3 PE=1 SV=1 -	1.198	1.102	1.133	0.895	1.537	1.070	1.512	1.034
I3L1H5	Diphthamide biosynthesis protein 1 (Fragment) OS=Homo sapien	1.825	1.021	1.238	1.144	1.357	1.158	1.131	1.011
Q14108	Lysosome membrane protein 2 OS=Homo sapiens GN=SCARB2 P	1.283	1.274	1.442	1.371	1.407	1.179	1.425	1.409
B2R9T9	cDNA, FLJ94551 OS=Homo sapiens PE=2 SV=1 - [B2R9T9_HUM/	1.643	1.615	1.509	1.615	1.179	1.380	1.784	1.632
P24390	ER lumen protein-retaining receptor 1 OS=Homo sapiens GN=KD	5.847	6.373	6.305	6.073	4.479	2.210	6.009	5.174
Q86YS6	Ras-related protein Rab-43 OS=Homo sapiens GN=RAB43 PE=1 S	1.006	1.508			1.295			
Q9H7H0	Methyltransferase-like protein 17, mitochondrial OS=Homo sapier	1.280	1.523	1.408	1.154	1.075	1.201	1.406	1.396
Q5JSB5	Transcription factor Dp-1 (Fragment) OS=Homo sapiens GN=TFD	1.102	2.179	2.058	1.790	1.767	0.991	2.128	2.807
Q6UX04	Peptidyl-prolyl cis-trans isomerase CWC27 homolog OS=Homo sa	1.518	1.396	1.253	1.074	1.369	1.340	1.368	1.258
H7C0M7	Methyltransferase-like protein 5 (Fragment) OS=Homo sapiens GI	1.625	1.306	2.020	1.577	1.583	1.027	1.627	1.544
Q15746	Myosin light chain kinase, smooth muscle OS=Homo sapiens GN=	1.493	1.550	1.308	0.788	1.398	1.200	1.239	1.021
Q9BSR8	Protein YIPF4 OS=Homo sapiens GN=YIPF4 PE=1 SV=1 - [YIPF4	3.034	2.139	2.775	2.464	1.433	1.434	1.754	2.491
E7ETU5	RNA-binding motif, single-stranded-interacting protein 1 OS=Hom	1.403	1.297	1.094	0.764	1.253	1.292	1.103	0.923
B2RA34	cDNA, FLJ94678, highly similar to Homo sapiens BCL2-associated	1.202	1.370	0.818	0.994	1.200	1.599	1.340	1.361
Q9UHE8	Metalloreductase STEAP1 OS=Homo sapiens GN=STEAP1 PE=1 S	2.461	2.831	2.050	1.721	2.191	1.360	1.999	1.641
P22670	MHC class II regulatory factor RFX1 OS=Homo sapiens GN=RFX1	1.024	1.364	1.105	0.960	1.039	1.134	1.499	1.315
Q6PK21	OGFR protein OS=Homo sapiens PE=2 SV=1 - [Q6PK21_HUMAN]	1.759	1.386	1.522	1.274	1.601	1.544	1.524	1.464
Q13325	Interferon-induced protein with tetratricopeptide repeats 5 OS=H	1.345	1.017	1.713	1.319	0.992	0.879	1.080	1.142
A0A024RC75	Chromodomain helicase DNA binding protein 2, isoform CRA_a OS	1.473	1.563	1.105	0.802	1.070	1.346	1.243	0.967
Q96L52	PP2A B56 gamma 2 OS=Homo sapiens PE=4 SV=1 - [Q96L52_HU	3.391	3.515	3.807	1.923	2.981	2.207	2.146	2.908
Q15800	Methylsterol monooxygenase 1 OS=Homo sapiens GN=MSMO1 P	1.906	1.903	1.423	1.035	1.565	1.212	1.705	1.179
A1X283	SH3 and PX domain-containing protein 2B OS=Homo sapiens GN=	1.099	0.797	0.886	0.656	0.880	0.854	0.749	0.756
Q5UCC4	ER membrane protein complex subunit 10 OS=Homo sapiens GN	0.926	1.117	0.853	0.782	0.688	1.133	0.996	0.908
Q99959	Plakophilin-2 OS=Homo sapiens GN=PKP2 PE=1 SV=2 - [PKP2_H	1.297	1.576	1.284	1.089	0.992	1.012	1.539	1.248
O95251	Histone acetyltransferase KAT7 OS=Homo sapiens GN=KAT7 PE=	1.547	1.544	1.121	0.900	1.366	1.244	1.320	1.012
D6REA0	Glutamyl-tRNA(Gln) amidotransferase subunit B, mitochondrial OS	1.174	1.213	1.108	1.048	1.063	1.162	1.331	1.096
B3KM89	cDNA FLJ10528 fis, clone NT2RP2000943, highly similar to Protei	1.297	1.040	1.350	0.973	1.488	1.222	1.413	0.999
Q8NFD5	AT-rich interactive domain-containing protein 1B OS=Homo sapie	1.156	1.391	0.995	0.638	1.284	1.308	1.078	0.826
B7ZLK1	KIAA0528 protein OS=Homo sapiens GN=KIAA0528 PE=2 SV=1	1.450	1.516	1.566	1.455	1.591	1.101	1.711	1.545
Q96RN5	Mediator of RNA polymerase II transcription subunit 15 OS=Homo	1.156	1.315	1.008	0.877	1.392	1.376	1.285	1.144
Q54A15	DTGCU2 OS=Homo sapiens GN=DTGCU2 PE=2 SV=1 - [Q54A15_	1.319	0.862	1.409	0.928	1.088	1.061	1.302	1.223
A0A087WZX0	B-cell CLL/lymphoma 9-like protein OS=Homo sapiens GN=BCL9L	1.448	1.075	0.682	0.415	1.450	1.241	1.077	0.722
E5RH64	7-methylguanosine phosphate-specific 5'-nucleotidase OS=Homo	0.444	0.860	0.756	0.844	0.484	0.742	0.891	1.055
Q2TAL8	Glutamine-rich protein 1 OS=Homo sapiens GN=QRICH1 PE=1 SV	0.877	1.077	0.796	0.488	1.021	1.086	0.872	0.733
O43760	Synaptogyrin-2 OS=Homo sapiens GN=SYNGR2 PE=1 SV=1 - [SI	1.058	1.069	1.012	1.227	0.637	0.707	0.606	0.587
Q86UL3	Glycerol-3-phosphate acyltransferase 4 OS=Homo sapiens GN=A(	1.247	1.335	1.182	1.149	1.031	1.161	1.286	1.244
Q9NRZ7	1-acyl-sn-glycerol-3-phosphate acyltransferase gamma OS=Homo	1.408	1.378	1.407	1.102	1.062	1.147	1.364	1.304

B4DGM5	cDNA FLJ53983, highly similar to IWS1 homolog OS=Homo sapie	1.776	1.728	1.232	0.919	1.828	1.336	1.541	1.223
H7C3C4	Sodium bicarbonate cotransporter 3 (Fragment) OS=Homo sapier	0.977	1.225	0.796	0.569	0.847	0.962	0.778	0.620
A0M8Q2	Phosphatase 2C motif OS=Homo sapiens PE=3 SV=1 - [A0M8Q2_	1.708	1.782	2.354	2.076	1.971	1.363	2.367	1.049
B4DYN3	cDNA FLJ55634, highly similar to Autophagy-related protein 9A O	1.898	1.847	2.097	1.863	1.665	1.313	2.252	2.016
A8K8Y8	cDNA FLJ77796, highly similar to Homo sapiens evolutionarily cor	1.744	1.704	1.472	1.209	1.762	1.326	1.620	1.353
Q59FZ8	Nebulette non-muscle isoform variant (Fragment) OS=Homo sapi	1.173	1.065	1.057	0.744	0.980	0.912	1.049	0.888
B4DGE7	cDNA FLJ61235, weakly similar to WD repeat protein YBL104C OS	1.510	1.387	1.312	1.148	1.403	1.153	1.331	1.429
B2RDV7	tRNA-dihydrouridine(47) synthase [NAD(P)(+)] OS=Homo sapien	1.431	1.145	1.002	0.965	1.242	1.069	1.053	0.702
H3BMM5	Uncharacterized protein OS=Homo sapiens PE=4 SV=1 - [H3BMN	1.319	1.154	1.333	1.020	1.232	1.212	1.322	0.948
B7Z954	cDNA FLJ61560, highly similar to Tight junction protein ZO-2 OS=	1.431	1.378	1.179	0.833	1.383	1.125	1.428	1.264
B4DVP2	cDNA FLJ57208, highly similar to 5'-AMP-activated protein kinase	1.284	0.902	1.187	0.857	0.981	1.151	1.247	1.188
A0A024R751	HSPC049 protein, isoform CRA_a OS=Homo sapiens GN=HSPC04	1.572	1.208	1.419	0.944	1.401	1.057	1.620	1.295
P51692	Signal transducer and activator of transcription 5B OS=Homo sap	1.019	0.792	1.006	0.745	1.094	0.940	1.093	1.047
Q9HCK8	Chromodomain-helicase-DNA-binding protein 8 OS=Homo sapien	1.093	1.260	0.941	0.658	1.050	1.305	1.229	0.845
V9GZ55	Proteasome assembly chaperone 2 (Fragment) OS=Homo sapiens	1.286	0.891	1.339	0.762	1.158	1.253	1.196	1.007
B7ZKT9	KIAA1033 protein OS=Homo sapiens GN=KIAA1033 PE=2 SV=1	1.419	0.987	1.127	0.946	1.210	0.989	1.245	0.990
Q59E97	Galectin (Fragment) OS=Homo sapiens PE=2 SV=1 - [Q59E97_H	3.197	1.598	3.065	2.100	2.293	0.841	1.736	1.271
Q658U3	Putative uncharacterized protein DKFZp666D023 (Fragment) OS=	0.972	0.717	0.890	1.063	0.950	0.836	0.936	0.923
O75179	Ankyrin repeat domain-containing protein 17 OS=Homo sapiens (	1.744	1.180	1.636	1.156	1.235	1.328	1.604	1.162
Q7Z589	Protein EMSY OS=Homo sapiens GN=EMSY PE=1 SV=2 - [EMSY_	1.018	1.061	0.537	0.468	0.891	1.112	0.839	0.703
B4DRY5	cDNA FLJ54538, highly similar to Transportin-2 OS=Homo sapien	2.583	1.739	2.958	1.898	1.665	1.161	1.800	1.841
Q59HG4	Elongation factor RNA polymerase II variant (Fragment) OS=Hom	2.507	2.539	0.927	2.122	1.703	1.500	2.612	0.956
Q14CS0	UBX domain-containing protein 2B OS=Homo sapiens GN=UBXN2	1.161	0.566	0.804	0.633	1.106	1.301	1.199	0.700
A8K0D4	cDNA FLJ78693, highly similar to Homo sapiens TAF6-like RNA po	1.721	1.035	1.620		1.620	1.439	2.271	1.169
Q96MU7	YTH domain-containing protein 1 OS=Homo sapiens GN=YTHDC1	1.519	1.497	1.242	1.088	1.402	1.337	1.522	1.537
Q15906	Vacuolar protein sorting-associated protein 72 homolog OS=Hom	1.189	1.394	0.895	0.711	1.291	1.435	1.344	1.064
B3KR81	cDNA FLJ33833 fis, clone CTONG2004126, highly similar to Homo	1.149	1.363	1.338	1.470	0.916	1.270	1.419	1.191
Q53TD0	Putative uncharacterized protein SP100 (Fragment) OS=Homo sa	1.285	1.218	1.104	0.828	1.362	1.216	1.036	1.024
Q8IWL3	Iron-sulfur cluster co-chaperone protein HscB, mitochondrial OS=	1.306	1.302	1.252	1.357	1.139	1.227	1.203	1.386
Q9NVX0	HAUS augmin-like complex subunit 2 OS=Homo sapiens GN=HAU	1.263	0.990	1.590	1.125	1.060	0.887	1.033	1.252
Q7L590	Protein MCM10 homolog OS=Homo sapiens GN=MCM10 PE=1 SV	1.150	2.849	2.967	1.808	0.952	2.374	3.101	2.639
Q6P3X3	Tetratricopeptide repeat protein 27 OS=Homo sapiens GN=TTC27	1.797	1.241	1.865	1.431	1.445	1.106	1.573	1.364
Q9Y6D6	Brefeldin A-inhibited guanine nucleotide-exchange protein 1 OS=I	1.458	1.262	1.483	1.099	1.146	1.168	1.292	1.139
A8K124	cDNA FLJ76213, highly similar to Homo sapiens suppressor of ha	1.022	1.032	0.712	0.735	0.951	1.052	1.205	0.924
D6W5Z8	Kruppel-like factor 16, isoform CRA_a OS=Homo sapiens GN=KLF	1.512	1.609			1.156	1.929		
O60518	Ran-binding protein 6 OS=Homo sapiens GN=RANBP6 PE=1 SV=	2.110	2.579	2.864	2.773	2.170	1.695	2.312	2.569
Q05209	Tyrosine-protein phosphatase non-receptor type 12 OS=Homo sa	1.092	0.688	0.926	0.657	0.994	0.877	0.773	0.860
B3KX15	cDNA FLJ44468 fis, clone UTERU2026025, moderately similar to S	1.686	1.594	1.210	0.846	1.986	1.270	1.375	1.226
Q9H1A4	Anaphase-promoting complex subunit 1 OS=Homo sapiens GN=A	1.582	1.552	1.351	1.167	1.642	1.315	1.604	1.329
Q9BRP1	Programmed cell death protein 2-like OS=Homo sapiens GN=PDC	1.896	1.464	2.043	1.891	1.637	1.267	2.039	1.931

Q9BVC4	Target of rapamycin complex subunit LST8 OS=Homo sapiens GN	2.411	2.091	2.158	1.850	1.756	1.368	2.153	2.170
E9PS17	N-terminal kinase-like protein OS=Homo sapiens GN=SCYL1 PE=	1.297	1.016	1.197	0.777	1.080	1.035	1.221	0.922
Q9NYL2	Mitogen-activated protein kinase kinase kinase MLT OS=Homo sa	1.309	1.181	1.260	0.938	1.106	0.997	1.040	0.731
Q53SV2	Putative uncharacterized protein FLJ10035 OS=Homo sapiens GN	1.765	1.090	1.592	1.242	1.593	1.126	1.461	1.304
A0A024R9Q8	Recombining binding protein suppressor of hairless (Drosophila),	1.505	1.459	1.002	0.898	1.219	0.965	0.909	1.019
P61077	Ubiquitin-conjugating enzyme E2 D3 OS=Homo sapiens GN=UBE	1.437	0.995	1.445	1.012	1.201	1.031	1.276	1.142
H0Y8J2	Centromere protein C (Fragment) OS=Homo sapiens GN=CENPC	0.699	0.968	0.874	0.866	0.514	1.186	0.896	0.940
B4DTF2	Annexin OS=Homo sapiens GN=ANXA8L1 PE=2 SV=1 - [B4DTF2_	1.133	0.802	1.079	1.268	1.155	0.757	1.081	1.243
E9PMB9	Malic enzyme (Fragment) OS=Homo sapiens GN=ME3 PE=3 SV=	1.102	1.134	1.008	1.003	0.947	1.106	0.987	0.988
Q9Y4B6	Protein VPRBP OS=Homo sapiens GN=VPRBP PE=1 SV=3 - [VPRI	1.381	1.363	1.050	0.849	1.288	1.230	1.165	0.768
Q16513	Serine/threonine-protein kinase N2 OS=Homo sapiens GN=PKN2	1.541	1.206	1.457	1.106	1.284	1.119	1.476	1.146
A8K3E7	cDNA FLJ76257, highly similar to Homo sapiens DIP13 beta (DIP1	1.069	0.722	1.106	0.815	1.227	1.410	0.951	0.671
Q7L7X3	Serine/threonine-protein kinase TAO1 OS=Homo sapiens GN=TAO	1.275	1.092	1.048	0.875	1.053	1.080	1.088	1.080
P49759	Dual specificity protein kinase CLK1 OS=Homo sapiens GN=CLK1	1.537	1.703	1.102	0.868	1.388	1.276	1.483	1.170
B4DP12	cDNA FLJ55119, highly similar to Homo sapiens ZW10 interactor	1.345	1.408	1.345	0.850	1.224	1.456	1.887	1.486
B2RDW9	cDNA, FLJ96803, highly similar to Homo sapiens GTP binding pro	1.076	1.054	1.191	0.863	1.099	0.903	1.250	0.950
B4DQX8	cDNA FLJ51723, highly similar to DCC-interacting protein 13 alpha	1.687	1.131	1.457	1.365	1.063	1.279	1.607	1.875
Q5T6F2	Ubiquitin-associated protein 2 OS=Homo sapiens GN=UBAP2 PE=	1.368	1.237	1.282	0.855	1.282	1.136	1.415	1.047
Q9NUD5	Zinc finger CCHC domain-containing protein 3 OS=Homo sapiens	1.555	1.407	1.309	0.697	0.996	1.249	0.742	0.820
Q8WU76	Sec1 family domain-containing protein 2 OS=Homo sapiens GN=S	1.782	1.886	1.913	2.045	1.757	1.504	1.775	1.998
A8K454	cDNA FLJ78187, highly similar to Homo sapiens calmegin, mRNA	1.669	1.627	1.746	1.773	1.297	1.269	1.867	1.538
Q9UKV5	E3 ubiquitin-protein ligase AMFR OS=Homo sapiens GN=AMFR PI	1.093	1.154	0.787	0.580	0.899	1.118	0.945	0.720
Q7Z6M1	Rab9 effector protein with kelch motifs OS=Homo sapiens GN=RA	1.298	1.085	1.440	1.532	1.342	1.096	1.737	1.277
B4E1P2	cDNA FLJ59223, highly similar to C-Rel proto-oncogene protein O	1.159	0.799	1.299	0.992	1.140	1.009	1.193	1.053
Q5U5Z3	Paraneoplastic antigen MA2 OS=Homo sapiens GN=PNMA2 PE=2	0.801	0.718	0.854	0.542	0.659	1.127	0.605	0.645
Q8IVM0	Coiled-coil domain-containing protein 50 OS=Homo sapiens GN=(	1.612	1.131	1.368	1.345	1.499	1.199	1.492	1.590
B2R6X8	cDNA, FLJ93169, highly similar to Homo sapiens GPAA1P anchor	0.965	1.060	0.921	0.866	0.974	1.084	1.186	0.938
Q9Y5B0	RNA polymerase II subunit A C-terminal domain phosphatase OS=	1.252	1.033	0.911	0.844	1.671	1.164	0.875	0.751
Q9NX05	Constitutive coactivator of PPAR-gamma-like protein 2 OS=Homo	1.089	0.843	0.973	0.771	1.025	1.052	0.750	0.718
Q7Z528	E3-16 OS=Homo sapiens PE=2 SV=1 - [Q7Z528_HUMAN]	1.995	1.523	0.933	0.701	1.981	1.152	1.794	1.082
Q9NVV0	Trimeric intracellular cation channel type B OS=Homo sapiens GN	2.375	2.802	2.638	2.592	1.986	1.422	2.894	2.669
O00400	Acetyl-coenzyme A transporter 1 OS=Homo sapiens GN=SLC33A1	2.964	2.718	2.662	2.648	1.950	1.711	2.775	2.221
C9J5X1	Tyrosine-protein kinase receptor OS=Homo sapiens GN=IGF1R PI	1.479	1.127	0.942	0.894	1.108	0.984	1.049	0.897
B4DTL8	cDNA FLJ61389, highly similar to Ras GTPase-activating protein 1	1.464	0.900	1.436	1.038	0.999	1.082	1.336	0.950
Q15386	Ubiquitin-protein ligase E3C OS=Homo sapiens GN=UBE3C PE=1	1.057	0.906	0.994	0.790	1.175	1.138	1.166	0.825
Q6JQN1	Acyl-CoA dehydrogenase family member 10 OS=Homo sapiens G	1.434	1.468	1.102	1.032	1.202	1.098	1.260	1.399
A0A087X207	Breast carcinoma-amplified sequence 3 OS=Homo sapiens GN=B	1.386	1.022	1.442	1.395	1.265	0.956	1.442	1.223
Q8IYS2	Uncharacterized protein KIAA2013 OS=Homo sapiens GN=KIAA2(	1.190	1.373	1.141	1.326	1.047	1.291	1.176	1.336
B4DH70	cDNA FLJ54601, highly similar to F-box/WD repeat protein 11 OS	0.773	0.682	0.594	0.357	0.996	0.912	0.851	0.489
B4DSG6	cDNA FLJ55520, highly similar to TFIIH basal transcription factor	1.712	2.053	1.712	1.293	1.548	1.320	1.625	1.475

P50851	Lipopolysaccharide-responsive and beige-like anchor protein OS=	1.389	0.943	1.203	0.910	1.356	0.956	1.121	1.025
Q92889	DNA repair endonuclease XPF OS=Homo sapiens GN=ERCC4 PE=	1.141	1.320	1.045	0.796	1.240	1.233	1.248	1.008
B4DFW2	Metalloproteinase inhibitor 2 OS=Homo sapiens GN=TIMP2 PE=2	4.430	3.550	1.468	1.099	2.893	2.087	2.317	1.952
Q9P270	SLAIN motif-containing protein 2 OS=Homo sapiens GN=SLAIN2	0.949	0.918	1.131			0.906		0.937
B4DR12	G-protein-coupled receptor 84 OS=Homo sapiens GN=GPR84 PE=	0.955	1.164	0.976	1.117	0.515	1.097	0.828	0.744
A8K2T9	cDNA FLJ78705, highly similar to Homo sapiens oral-facial-digital	1.299	1.821	1.253	1.241	1.291	1.583	1.483	1.509
Q9Y2D5	A-kinase anchor protein 2 OS=Homo sapiens GN=AKAP2 PE=1 SV	1.679	2.065	1.659	1.350	1.551	1.580	1.747	1.722
Q9Y4G6	Talin-2 OS=Homo sapiens GN=TLN2 PE=1 SV=4 - [TLN2_HUMAN	1.256	1.011	1.045	1.095	1.048	0.885	1.092	0.909
Q53GA4	Pleckstrin homology-like domain family A member 2 OS=Homo sa	2.657	1.474	0.651		2.215	1.979	1.718	1.224
D6RDW4	Macrophage erythroblast attacher OS=Homo sapiens GN=MAEA I	1.411	1.310	1.175	0.688	1.259	1.367	1.153	0.815
Q9UK23	N-acetylglucosamine-1-phosphodiester alpha-N-acetylglucosamini	2.154	2.202	1.783	2.009	1.868	1.515	2.429	2.417
Q9P253	Vacuolar protein sorting-associated protein 18 homolog OS=Hom	1.436	1.316	1.354	1.106	1.323	1.246	1.138	0.960
Q9UEY4	Anion exchanger 2 type b1 OS=Homo sapiens GN=SLC4A2 PE=4	1.139	2.045	1.435	1.276	1.483	1.910	1.546	1.329
P54803	Galactocerebrosidase OS=Homo sapiens GN=GALC PE=1 SV=2 -	1.665	1.403	1.505	1.885	1.569	1.210	1.690	1.895
O15234	Protein CASC3 OS=Homo sapiens GN=CASC3 PE=1 SV=2 - [CAS	2.825	2.421	2.263	1.730	2.166	1.514	2.294	2.348
A8K7S5	Katanin p60 ATPase-containing subunit A1 OS=Homo sapiens GN	1.374	1.210	1.336	0.869	1.122	1.146	1.403	1.033
Q9P265	Disco-interacting protein 2 homolog B OS=Homo sapiens GN=DII	1.337	1.169	1.242	0.915	1.205	1.100	1.198	0.985
P42345	Serine/threonine-protein kinase mTOR OS=Homo sapiens GN=M	1.515	1.322	1.466	1.127	1.364	1.319	1.474	1.302
Q8NBK3	Sulfatase-modifying factor 1 OS=Homo sapiens GN=SUMF1 PE=1	1.233	1.108	2.464	2.016	1.742	1.211	1.711	1.693
Q7Z7F0	UPF0469 protein KIAA0907 OS=Homo sapiens GN=KIAA0907 PE	1.184	0.861	0.848	0.642	1.160	1.291	0.942	0.699
O43670	BUB3-interacting and GLEBS motif-containing protein ZNF207 OS	1.314	1.408	1.049	0.740	1.338	1.388	1.251	0.984
Q8IZ83	Aldehyde dehydrogenase family 16 member A1 OS=Homo sapien	1.412	0.975	1.357	1.039	1.300	1.017	1.188	1.007
Q59GT1	Conserved helix-loop-helix ubiquitous kinase variant (Fragment) (	2.900	1.778	2.117	1.901	2.130	1.375	2.077	2.134
Q5T5X7	BEN domain-containing protein 3 OS=Homo sapiens GN=BEND3	1.571	2.611	3.274	2.541	1.609	1.615	2.324	3.266
B7Z670	cDNA FLJ61703, highly similar to Neuronal cell adhesion molecule	0.927	1.603	1.018	1.020	1.007	0.961	0.871	0.559
B3KU43	cDNA FLJ39177 fis, clone OCBBF2003925, highly similar to Homo	2.039	1.783	1.311	1.066	2.028	1.221	1.838	1.612
O60671	Cell cycle checkpoint protein RAD1 OS=Homo sapiens GN=RAD1	1.463	1.243	1.286	1.016	1.358	1.449	1.302	1.369
B4DE07	cDNA FLJ52472 OS=Homo sapiens PE=2 SV=1 - [B4DE07_HUMA	1.017	1.244	1.118	0.772	1.029	1.370	1.048	0.968
Q99996	A-kinase anchor protein 9 OS=Homo sapiens GN=AKAP9 PE=1 SV	1.217	1.329	1.240	1.066	1.160	1.185	1.240	1.177
Q9H267	Vacuolar protein sorting-associated protein 33B OS=Homo sapien	1.309	1.097	1.445	0.891	1.059	0.917	1.010	0.897
Q9ULF5	Zinc transporter ZIP10 OS=Homo sapiens GN=SLC39A10 PE=1 S	1.199	1.560	1.048	1.073	1.063	1.210	1.060	1.126
Q01970	1-phosphatidylinositol 4,5-bisphosphate phosphodiesterase beta-	1.571	1.161	1.547	1.188	1.301	1.191	1.337	1.345
Q6YP21	Kynurenine--oxoglutarate transaminase 3 OS=Homo sapiens GN=	1.127	0.969	1.280	1.093	0.929	1.031	0.989	1.213
Q5EBL4	RILP-like protein 1 OS=Homo sapiens GN=RILPL1 PE=1 SV=1 - [	1.295	0.704	1.369	1.116	1.226	0.901	1.162	0.944
Q96BN8	Ubiquitin thioesterase otulin OS=Homo sapiens GN=OTULIN PE=	1.552	1.169	1.248	1.139	1.262	1.346	1.338	1.426
Q6XYC5	LP6054 OS=Homo sapiens GN=RP3-402G11.12 PE=2 SV=1 - [Q6	1.283	1.390	1.194	1.082	1.018	1.312	1.465	1.359
E9PM35	Beta-arrestin-1 (Fragment) OS=Homo sapiens GN=ARRB1 PE=1	1.116		1.246			0.889	1.209	1.048
E7EN20	E3 ubiquitin-protein ligase TRIM33 OS=Homo sapiens GN=TRIM3	1.623	1.437	0.965	0.774	1.559	1.277	1.254	1.090
Q9UBV7	Beta-1,4-galactosyltransferase 7 OS=Homo sapiens GN=B4GALT7	1.146	0.938	0.807		0.990	1.051	1.134	
C9JYN0	Synaptophysin-like protein 1 OS=Homo sapiens GN=SYPL1 PE=1	1.333	1.346	1.230	1.210	1.133	1.242	1.287	1.231

Q12913	Receptor-type tyrosine-protein phosphatase eta OS=Homo sapier	1.536	1.727	1.071	1.217	1.265	1.123	1.234	1.252
P68543	UBX domain-containing protein 2A OS=Homo sapiens GN=UBXN2	2.535	2.349	1.516		1.716	1.648	2.088	2.163
B3KUJ0	cDNA FLJ39996 fis, clone STOMA2002166, highly similar to Splicir	1.500	1.362	1.130	1.017	1.300	1.240	1.211	0.903
O95466	Formin-like protein 1 OS=Homo sapiens GN=FMNL1 PE=1 SV=3	1.138	0.732	1.178	0.902	1.117	1.041	1.114	
Q8N6T7	NAD-dependent protein deacetylase sirtuin-6 OS=Homo sapiens (	1.874	2.014	1.561	1.169	1.503	1.773	1.456	1.399
A0A087WWW9	B-cell lymphoma/leukemia 10 OS=Homo sapiens GN=BCL10 PE=	0.159	0.142	0.111	0.096	0.325	0.831	0.208	0.187
B4DHI4	cDNA FLJ60536, highly similar to Death-associated protein kinase	1.449	1.343	1.098	0.814	1.474	1.192	1.212	0.936
Q9UBB6	Neurochondrin OS=Homo sapiens GN=NCDN PE=1 SV=1 - [NCD	2.882	3.772	3.916	4.376	1.465	1.420	2.309	3.146
Q6P1A2	Lysophospholipid acyltransferase 5 OS=Homo sapiens GN=LPCAT	3.207	2.843	2.503	2.101	2.146	1.489	2.542	2.365
Q9NQT8	Kinesin-like protein KIF13B OS=Homo sapiens GN=KIF13B PE=1	1.691	1.126	1.523	1.228	1.547	1.032	1.497	0.919
A8K3T2	cDNA FLJ77961, highly similar to Homo sapiens occludin (OCLN),	1.464	2.143	1.683	1.400	1.741	1.325	2.017	1.596
Q69YN4	Protein virilizer homolog OS=Homo sapiens GN=KIAA1429 PE=1	2.248	2.621	1.648	1.622	1.654	1.851	2.201	1.945
P49754	Vacuolar protein sorting-associated protein 41 homolog OS=Hom	1.834	1.878	2.149	1.610	1.618	1.348	1.798	1.772
A0A024R442	Aspartyl aminopeptidase, isoform CRA_b OS=Homo sapiens GN=	1.549	1.130	1.310	1.229	1.318	1.007	1.205	1.263
B2RAW0	cDNA, FLJ95154, highly similar to Homo sapiens disabled homolo	1.376	1.029	1.139	1.054	1.177	1.013	1.237	1.217
O94817	Ubiquitin-like protein ATG12 OS=Homo sapiens GN=ATG12 PE=1	1.174	0.745	1.038	0.840	0.939	0.835	0.939	0.915
Q8NB90	Spermatogenesis-associated protein 5 OS=Homo sapiens GN=SP/	1.351	0.966	1.153	0.839	1.074	1.033	1.154	0.977
O75381	Peroxisomal membrane protein PEX14 OS=Homo sapiens GN=PE	1.237	1.378	1.138	1.235	0.890	1.210	1.403	1.214
O14981	TATA-binding protein-associated factor 172 OS=Homo sapiens GN	1.254	1.286	1.122	0.814	1.183	1.095	1.076	0.920
Q53SN6	Enhancer of polycomb homolog (Fragment) OS=Homo sapiens G	1.342	1.928	1.179	1.179	1.554	1.731	1.532	1.272
P19012	Keratin, type I cytoskeletal 15 OS=Homo sapiens GN=KRT15 PE=	1.425	1.819	1.376	0.851	1.218	1.090	1.280	1.246
Q6QNY0	Biogenesis of lysosome-related organelles complex 1 subunit 3 O	1.261	1.121	1.242	0.840	1.059	1.019	1.106	0.968
P61966	AP-1 complex subunit sigma-1A OS=Homo sapiens GN=AP1S1 PE	1.290	1.245	0.963	0.875	1.145	1.053	1.066	1.013
O75529	TAF5-like RNA polymerase II p300/CBP-associated factor-associat	2.465	2.474	2.226	1.700	2.273	1.674	2.180	2.398
P17707	S-adenosylmethionine decarboxylase proenzyme OS=Homo sapie	1.764	1.476	1.396	0.629	1.515	1.516	1.454	0.689
Q86W92	Liprin-beta-1 OS=Homo sapiens GN=PPFIBP1 PE=1 SV=2 - [LIPE	0.928	0.849	1.175	0.799	0.974	1.009	0.966	0.756
J3KP52	Transcriptional enhancer factor TEF-1 OS=Homo sapiens GN=TE/	1.098	1.243	0.620	0.656	1.114	0.907	1.077	0.966
E9PN41	Tetraspanin-4 (Fragment) OS=Homo sapiens GN=TSPAN4 PE=4 S	2.655	3.103	1.811	1.365	2.094	1.261	2.060	1.422
D3DR32	M-phase phosphoprotein 1, isoform CRA_a OS=Homo sapiens GN	1.208	1.830	1.255	0.956	0.780	1.525	1.928	1.405
B2RAA8	cDNA, FLJ94802 OS=Homo sapiens PE=2 SV=1 - [B2RAA8_HUM/	1.665	1.176	1.592	1.258	1.243	1.210	1.955	1.568
B3KSW0	CDP-diacylglycerol--inositol 3-phosphatidyltransferase (Phosphatic	1.335	1.313	1.102	0.985	1.031	1.091	1.229	1.107
I3L419	Protein FAM64A OS=Homo sapiens GN=FAM64A PE=1 SV=1 - [I3		1.427	1.942				2.169	1.336
Q96EB1	Elongator complex protein 4 OS=Homo sapiens GN=ELP4 PE=1 S	1.477	0.707	1.634	1.014	1.329	0.749	1.168	0.766
Q16222	UDP-N-acetylhexosamine pyrophosphorylase OS=Homo sapiens (	1.433	1.109	1.667	1.388	1.211	1.190	1.520	1.530
D3DPC4	FAST kinase domain-containing protein 1 OS=Homo sapiens GN=	1.360	1.632	1.380	1.585	0.957	1.049	1.672	1.275
Q96JH7	Deubiquitinating protein VCIP135 OS=Homo sapiens GN=VCPIP1	1.416	1.032	1.274	0.880	1.224	1.049	1.155	1.019
B4DW43	cDNA FLJ56162, highly similar to N-acetylgalactosamine kinase (E	1.170	0.848	1.277	1.008	1.055	0.920	1.011	1.058
D6RFW1	Small glutamine-rich tetratricopeptide repeat-containing protein b	2.978	2.175	3.132	2.836	2.861	1.503	2.690	2.469
Q6ZMI0	Protein phosphatase 1 regulatory subunit 21 OS=Homo sapiens (	1.627	1.092	1.370	0.947	1.300	1.147	1.370	1.034
E7ENQ1	Mitogen-activated protein kinase kinase kinase kinase 4 OS=Hom	1.481	0.894	1.452	0.892	1.073	1.030	1.034	1.165

Q9UKG9	Peroxisomal carnitine O-octanoyltransferase OS=Homo sapiens G	1.367	1.550	1.293	1.449	0.992	1.325	1.252	1.263
J3QS47	Cytochrome b561 OS=Homo sapiens GN=CYB561 PE=4 SV=1 - [	1.358	2.027	1.595	1.581	0.901	1.418	2.300	1.733
K7EMK9	Lipid phosphate phosphohydrolase 2 (Fragment) OS=Homo sapie	1.828	2.161	1.358	1.419	1.779	1.396	1.679	1.508
Q9H0X4	Protein ITFG3 OS=Homo sapiens GN=ITFG3 PE=1 SV=1 - [ITFG	2.177	3.089	2.355	2.783	2.107	1.269	2.712	2.624
A8K8N3	cDNA FLJ78740, highly similar to Homo sapiens sperm associated	1.575	1.183	1.068	0.819	1.447	1.432	1.213	1.061
D9ZGF8	Rho-associated protein kinase OS=Homo sapiens GN=ROCK1 PE	1.408	0.962	1.434	1.078	1.130	0.974	1.364	0.971
Q9UGU0	Transcription factor 20 OS=Homo sapiens GN=TCF20 PE=1 SV=3	1.145	1.320	1.143	0.965	0.973	1.094	1.146	1.256
B2RA72	cDNA, FLJ94734, Homo sapiens CHMP1.5 protein (CHMP1.5), mR	1.240	0.878	1.074	0.775	1.027	0.892	0.978	0.716
A6NKD9	Coiled-coil domain-containing protein 85C OS=Homo sapiens GN=						0.903	0.546	
Q6VY07	Phosphofurin acidic cluster sorting protein 1 OS=Homo sapiens G	1.186	0.692	0.788	0.661	1.012	0.925	1.019	0.863
P33981	Dual specificity protein kinase TTK OS=Homo sapiens GN=TTK P	1.851	1.357	1.281	0.824	0.908	0.990	1.458	1.507
Q9UNY4	Transcription termination factor 2 OS=Homo sapiens GN=TTF2 P	1.937	1.814	2.207	1.466	1.574	1.305	2.677	2.062
Q5JQQ4	Protein DPCD OS=Homo sapiens GN=DPCD PE=1 SV=1 - [Q5JQ	1.511	0.878	1.317	0.964	1.183	1.071	0.963	0.856
Q96LJ7	Dehydrogenase/reductase SDR family member 1 OS=Homo sapie	1.719	1.832	1.887	1.714	1.769	1.792	1.820	1.595
Q9UGR2	Zinc finger CCCH domain-containing protein 7B OS=Homo sapien	1.136	1.275	1.150	0.761	1.352	1.290	1.162	1.008
C9JPX5	MLN64 N-terminal domain homolog (Fragment) OS=Homo sapier	2.267	2.075	1.692	1.626	1.324	1.070	1.290	1.400
Q6P444	Mitochondrial fission regulator 2 OS=Homo sapiens GN=MTFR2 P	1.195	1.318	1.016	0.895	1.138	0.956	1.132	1.157
Q9Y597	BTB/POZ domain-containing protein KCTD3 OS=Homo sapiens G	1.438	1.900	1.680	1.300	1.336	1.566	1.778	1.718
Q96BW5	Phosphotriesterase-related protein OS=Homo sapiens GN=PTER	1.189	0.799	1.106	0.923	1.081	0.974	1.056	0.754
Q9Y6D5	Brefeldin A-inhibited guanine nucleotide-exchange protein 2 OS=	1.405	1.003	1.368	0.895	1.223	1.020	1.319	1.036
H0Y329	WD repeat domain phosphoinositide-interacting protein 4 (Fragm	2.057	1.181	1.394	1.294	1.623	1.030	1.375	1.309
Q96EK4	THAP domain-containing protein 11 OS=Homo sapiens GN=THAP	1.318	1.561	1.188	1.018	1.169	1.541	1.367	1.287
Q8IUR7	Armadillo repeat-containing protein 8 OS=Homo sapiens GN=ARI	0.934	1.176	0.977	0.818	1.124	1.074	1.125	0.858
A0A024R857	F-box and leucine-rich repeat protein 18, isoform CRA_b OS=Hon	0.880	0.891	1.001	0.541	1.159	0.882	0.965	0.939
Q32P28	Prolyl 3-hydroxylase 1 OS=Homo sapiens GN=LEPRE1 PE=1 SV=	1.594	1.299	1.519	1.550	1.811	1.273	1.580	1.450
O00767	Acyl-CoA desaturase OS=Homo sapiens GN=SCD PE=1 SV=2 - [/	1.374	1.029	0.397	0.164	1.216	0.944	0.781	0.315
Q6UX07	Dehydrogenase/reductase SDR family member 13 OS=Homo sap	1.207	1.486	1.318	1.093	1.161	1.185	1.440	1.103
B4DTK6	cDNA FLJ50670, highly similar to RNA polymerase I-specific trans	2.085	2.690	3.500	3.977	1.863	2.200	4.025	4.079
O15066	Kinesin-like protein KIF3B OS=Homo sapiens GN=KIF3B PE=1 SV	1.036	1.020	1.061	0.808	0.990	0.987	0.823	0.760
H0YG27	Tripartite motif-containing protein 65 (Fragment) OS=Homo sapie						0.975		
Q659A1	Little elongation complex subunit 2 OS=Homo sapiens GN=ICE2	1.336	1.319	1.134	0.622	1.196	1.096	1.234	1.061
P08047	Transcription factor Sp1 OS=Homo sapiens GN=SP1 PE=1 SV=3	3.055	3.330	2.603	2.229	2.232	1.363	3.189	2.583
Q9BQ69	O-acetyl-ADP-ribose deacetylase MACROD1 OS=Homo sapiens G	0.655		0.785	0.608		0.635	0.667	0.857
Q9UGT4	Sushi domain-containing protein 2 OS=Homo sapiens GN=SUSD2	1.469	1.584	1.107	1.241	1.276	1.034	1.336	1.135
Q8N6M3	Fat storage-inducing transmembrane protein 2 OS=Homo sapiens	1.502	1.382	1.590	1.625	2.219	1.370	1.093	2.689
Q86SY9	Full-length cDNA clone CS0DJ011YD14 of T cells (Jurkat cell line)	1.129	1.227	1.152	1.147	0.935	1.194	1.230	1.243
A9QQ14	Actin nucleation promoting factor (Fragment) OS=Homo sapiens	1.410	0.985	1.145	0.842	1.327	1.019	1.118	0.886
Q709F0	Acyl-CoA dehydrogenase family member 11 OS=Homo sapiens G	1.194	1.442	1.206	1.194	1.141	1.101	1.275	1.227
Q7Z6J9	tRNA-splicing endonuclease subunit Sen54 OS=Homo sapiens GN	1.366	1.054	1.097	0.800	1.447	1.328	1.093	0.785
Q68EM7	Rho GTPase-activating protein 17 OS=Homo sapiens GN=ARHGA	1.444	1.235	1.331	1.056	1.258	1.042	1.311	1.159

B2R6K2	cDNA, FLJ92993, highly similar to Homo sapiens F-box and leucir	1.497	1.851	1.528	1.534	1.231	1.251	1.297	1.489
H0YA55	Serum albumin (Fragment) OS=Homo sapiens GN=ALB PE=1 SV	2.288	0.369	1.400	0.458	2.205	1.307	1.442	1.264
Q6ZR87	cDNA FLJ46554 fis, clone THYMU3038970 OS=Homo sapiens PE=	1.185	1.600		1.249	1.457	1.386		1.440
B4DF62	cDNA FLJ58794, highly similar to Pogo transposable element with	1.754	2.008			1.135	1.800	1.548	
Q6GMV2	SET and MYND domain-containing protein 5 OS=Homo sapiens G	1.361	1.066	1.633	1.472	1.182	1.083	1.356	1.374
A8K6K7	cDNA FLJ76881, highly similar to Homo sapiens glycogen synthas	1.701	1.271	1.406	1.244	1.537	1.242	2.065	1.466
B2RB38	cDNA, FLJ95288, highly similar to Homo sapiens feline leukemia v	1.649	2.347	1.997	1.844	1.610	1.368	2.257	1.460
Q96BZ8	Leukocyte receptor cluster member 1 OS=Homo sapiens GN=LEN	1.200	1.200	1.478	1.128	1.299	1.038	1.162	1.016
Q9UKZ1	CCR4-NOT transcription complex subunit 11 OS=Homo sapiens G	1.766	1.842	1.324	0.801	1.502	1.301	1.226	1.059
Q9H5I1	Histone-lysine N-methyltransferase SUV39H2 OS=Homo sapiens	1.116	1.432	1.265	1.039	1.048	1.391	1.268	1.383
B4DL81	cDNA FLJ56369, highly similar to Homo sapiens phosphatidylglyc	0.609	0.596	0.608	0.810	0.400	1.003	1.125	0.474
Q5T200	Zinc finger CCCH domain-containing protein 13 OS=Homo sapien	1.250	1.669	1.277	0.968	1.302	1.498	1.532	1.208
B2R728	cDNA, FLJ93255, highly similar to Homo sapiens solute carrier far	1.439	1.625	1.045	1.231	1.387	1.185	1.132	1.345
Q9UHQ1	Nuclear prelamin A recognition factor OS=Homo sapiens GN=NAI	1.750	1.320	1.517	1.148	1.694	1.454	1.562	1.770
J3QQN7	Glycerophosphodiester phosphodiesterase domain-containing pro	2.179	3.055	2.851	2.566	1.699	1.919	2.806	2.997
F8W9X7	Coiled-coil domain-containing protein 93 OS=Homo sapiens GN=	1.109	1.260	1.082	0.910	1.053	1.156	1.097	0.916
B7Z7J5	cDNA FLJ61673, highly similar to Retinoic acid receptor RXR-beta	1.001	1.708	0.996	0.581	1.046	1.380	1.102	0.997
B4DZG2	cDNA FLJ56521, highly similar to Homo sapiens transcription term	2.060	2.400	1.938	1.776	2.000	1.889	2.478	2.089
Q8NBM8	Prenylcysteine oxidase-like OS=Homo sapiens GN=PCYOX1L PE=	1.466	1.700	1.846	1.934	1.391	1.317	1.618	1.975
A0A024R254	Melanoma antigen family D, 1, isoform CRA_a OS=Homo sapiens	1.100	1.156	0.859	0.650	1.243	1.103	1.210	0.746
O14495	Lipid phosphate phosphohydrolase 3 OS=Homo sapiens GN=PPA					1.763			
D6RBJ7	Vitamin D-binding protein OS=Homo sapiens GN=GC PE=1 SV=1	3.395	0.815	1.713	0.982	3.336	1.190	1.504	1.641
Q53FD1	Zinc finger, HIT domain containing 2 variant (Fragment) OS=Hon	1.729	0.937	1.122	1.022	1.268	1.240	1.353	0.894
H7C0S8	Uncharacterized protein (Fragment) OS=Homo sapiens PE=4 SV=	1.582	1.599	1.710	1.213	1.633	1.613	1.575	1.667
Q6PJG2	ELM2 and SANT domain-containing protein 1 OS=Homo sapiens	1.146	1.471	1.125	0.998	0.904	1.338	1.164	1.001
B3KN28	cDNA FLJ13370 fis, clone PLACE1000653, highly similar to Phospl	1.492	1.318	1.949	1.424	1.581	1.068	1.751	1.406
O75151	Lysine-specific demethylase PHF2 OS=Homo sapiens GN=PHF2 P	0.961	1.459	1.022	0.850	0.957	1.175	1.029	0.999
Q9Y4C2	Protein FAM115A OS=Homo sapiens GN=FAM115A PE=1 SV=3 -	2.142	2.203	2.032	1.201	1.810	1.697	2.083	1.345
B4DPS7	cDNA FLJ58519, highly similar to Hermansky-Pudlak syndrome 3	1.378	0.872	1.105	0.979	0.949	1.073	0.993	0.961
H0Y613	Genetic suppressor element 1 (Fragment) OS=Homo sapiens GN=	1.357	1.183	0.743	0.529	1.194	1.058	1.004	0.942
A0A087X0D5	Pro-cathepsin H OS=Homo sapiens GN=CTSH PE=4 SV=1 - [A0A	1.745	1.551	1.735	2.052	1.489	1.360	1.951	2.500
A0A024R776	CCR4-NOT transcription complex, subunit 4, isoform CRA_a OS=I	2.143	1.777	2.110	1.495	1.334	1.516	1.816	1.526
Q9H1I8	Activating signal cointegrator 1 complex subunit 2 OS=Homo sap	2.098	2.068	2.086	1.542	1.735	1.169	1.811	1.764
Q9Y2X0	Mediator of RNA polymerase II transcription subunit 16 OS=Hom	1.452	1.495	1.031	0.784	1.413	1.211	1.277	1.062
B2RTX8	WAPAL protein OS=Homo sapiens GN=WAPAL PE=2 SV=1 - [B2R	1.439	1.565	1.153	1.055	1.335	1.133	1.451	1.252
Q5SNT2	Transmembrane protein 201 OS=Homo sapiens GN=TMEM201 PE	3.250	4.175	3.596	3.558	2.485	1.900	3.640	3.271
Q5JSZ5	Protein PRRC2B OS=Homo sapiens GN=PRRC2B PE=1 SV=2 - [P	1.569	1.943	1.341	1.232	1.186	1.348	1.247	0.992
Q86WR7	Proline and serine-rich protein 2 OS=Homo sapiens GN=PROSER	1.404	1.196	1.559	1.178	1.541	1.266	1.385	1.075
A8K0J3	cDNA FLJ76732, highly similar to Homo sapiens TAO kinase 3 (TA	1.099	0.764	0.938	0.744	0.878	0.984	0.950	0.880
B4DDK7	cDNA FLJ51792, highly similar to Smad nuclear-interacting protei	0.902	0.708	0.619	0.512	1.062	1.029	1.089	

V9GY48	Uncharacterized protein (Fragment) OS=Homo sapiens PE=4 SV=	1.267	1.235	0.954	0.652	1.198	1.285	1.101	0.788
Q9UPN7	Serine/threonine-protein phosphatase 6 regulatory subunit 1 OS=	2.009	1.852	2.688	2.202	2.053	1.257	2.245	1.908
H0Y934	Radical S-adenosyl methionine domain-containing protein 1, mito	0.629	1.152	0.932	0.784	0.852	1.021	1.346	0.875
A0A087WUU9	Zinc finger protein 36, C3H1 type-like 2 OS=Homo sapiens GN=Z	1.571	1.595	1.171	0.884	1.234	1.856	1.199	0.853
Q8TEW0	Partitioning defective 3 homolog OS=Homo sapiens GN=PARD3 P	1.417	1.078	1.160	1.072	1.148	1.126	1.465	1.183
Q6IA86	Elongator complex protein 2 OS=Homo sapiens GN=ELP2 PE=1 S	1.172	0.875	0.879	0.961	1.186	1.021	1.093	0.932
F5GZK9	mRNA-decapping enzyme 1B OS=Homo sapiens GN=DCP1B PE=	1.724	1.406	1.330	1.350	1.479	1.526	1.837	1.369
B7ZLB7	C5orf44 protein (Fragment) OS=Homo sapiens GN=C5orf44 PE=:	1.213	1.061	1.206	0.885	1.321	1.023	1.277	1.165
B7Z7Q6	cDNA FLJ57232, highly similar to Lysosomal Pro-X carboxypeptida	1.897	1.560	1.930	2.124	1.802	1.212	1.973	1.908
Q9Y613	FH1/FH2 domain-containing protein 1 OS=Homo sapiens GN=FH	1.352	0.773	1.037	0.739	1.116	0.942	1.189	0.873
Q96CF2	Charged multivesicular body protein 4c OS=Homo sapiens GN=C	1.314	1.470	1.964	0.729	1.271	1.651	1.967	1.710
Q13488	V-type proton ATPase 116 kDa subunit a isoform 3 OS=Homo sa	1.599	1.158	0.985	1.036	1.305	1.119	0.968	1.238
Q9HBI1	Beta-parvin OS=Homo sapiens GN=PARVB PE=1 SV=1 - [PARVB_	1.450	1.179	1.263	1.072	1.130	1.570	0.928	1.191
G3V2I3	RCC1 domain-containing protein 1 OS=Homo sapiens GN=RCCD1	1.036	1.075	1.576	0.868	1.080	0.974	1.096	0.887
Q13952	Nuclear transcription factor Y subunit gamma OS=Homo sapiens	1.152	1.345	1.360	1.107	1.219	1.163	1.244	1.434
Q8WVY7	Ubiquitin-like domain-containing CTD phosphatase 1 OS=Homo s	2.799	2.259	2.404	1.661	1.613	1.002	2.070	1.896
Q59FQ8	Spinster variant (Fragment) OS=Homo sapiens PE=2 SV=1 - [Q5	1.993	1.893	1.988	1.622	2.301	1.404	2.046	1.970
D6RJF0	Centromere protein K OS=Homo sapiens GN=CENPK PE=4 SV=1	1.671	1.998	1.985	1.649	1.444	1.427	2.043	1.970
Q9NQY0	Bridging integrator 3 OS=Homo sapiens GN=BIN3 PE=1 SV=1 - [	1.102	0.829	0.818	0.615	1.116	1.093	1.056	0.738
H0YED9	Wilms tumor protein (Fragment) OS=Homo sapiens GN=WT1 PE	0.818	1.307	1.122	0.774	0.945	1.665	1.286	1.339
I6L9K0	TRIT1 protein OS=Homo sapiens GN=TRIT1 PE=2 SV=1 - [I6L9K	1.156						0.835	
Q6YHU6	Thyroid adenoma-associated protein OS=Homo sapiens GN=THA	1.499	0.899	1.497	1.182	1.276	1.007	1.231	1.127
Q9NX00	Transmembrane protein 160 OS=Homo sapiens GN=TMEM160 PE	2.050	2.295	1.799	1.867	1.411	1.110	1.904	1.847
B4E0K6	cDNA FLJ56191, highly similar to Homo sapiens valyl-tRNA synthe	0.977	0.963	0.827	0.809	0.703	0.960	0.857	0.854
B4DKS0	cDNA FLJ53381, highly similar to Monocarboxylate transporter 1 (	1.478	2.523	2.257	2.490	1.193	1.666	2.055	1.962
P49715	CCAAT/enhancer-binding protein alpha OS=Homo sapiens GN=CE	1.405	3.973	2.933	1.371	1.119	3.086	3.095	2.333
Q96L35	EPH receptor B4, isoform CRA_b OS=Homo sapiens GN=EPHB4 P	0.645	0.694	0.213	0.188	0.734	1.009	0.677	0.597
Q92610	Zinc finger protein 592 OS=Homo sapiens GN=ZNF592 PE=1 SV=	1.346	1.511	1.292	0.958	1.232	1.274	1.319	1.283
E9PNU1	Nucleoside diphosphate kinase 7 OS=Homo sapiens GN=NME7 P	1.500	1.257	1.511		1.398	1.515	1.570	1.325
Q16656	Nuclear respiratory factor 1 OS=Homo sapiens GN=NRF1 PE=1 S	1.255	1.141	1.490	1.349	1.114	1.216	1.281	1.486
B7Z2X6	cDNA FLJ52145, highly similar to G-protein coupled receptor fami	0.904	1.134	0.832	0.746	0.866	1.327	0.910	0.728
Q5TZT0	Three prime repair exonuclease 1 OS=Homo sapiens GN=TREX1	1.504	1.720			0.917	1.014		
I7JB59	ABCG2 protein OS=Homo sapiens GN=ABCG2 PE=4 SV=1 - [I7JB	1.107	1.219	0.845	0.940	1.073	0.993	1.048	0.904
B2RAN0	cDNA, FLJ95010, highly similar to Homo sapiens Bloom syndrome	1.259	0.768	0.959	0.952	1.161	1.598	1.693	1.339
Q05BL1	TP53BP2 protein OS=Homo sapiens GN=TP53BP2 PE=2 SV=1 - [	1.685	1.581	1.413	0.732	0.993	1.153	1.287	1.014
Q8NCF5	NFATC2-interacting protein OS=Homo sapiens GN=NFATC2IP PE=	1.029	1.017	1.103	0.632	0.687	0.707	1.044	1.018
E5RFX8	Cyclin-C (Fragment) OS=Homo sapiens GN=CCNC PE=1 SV=1 - [	1.579	1.387	0.966	0.581	1.431	1.201	1.345	0.881
Q5MIZ7	Serine/threonine-protein phosphatase 4 regulatory subunit 3B OS	1.666	1.632	1.390	0.894	1.549	1.659	1.378	1.230
Q6P4R8	Nuclear factor related to kappa-B-binding protein OS=Homo sapi	1.140	1.450	1.059	1.075	1.088	1.352	1.503	1.245
B4E2D6	cDNA FLJ61066, highly similar to Segment polarity protein dishev	1.505	1.327	1.263	1.220	1.167	0.947	1.163	1.419

O43463	Histone-lysine N-methyltransferase SUV39H1 OS=Homo sapiens (	1.932	3.750	3.692	3.661	1.408	2.015	2.420	2.330
Q5VIR6	Vacuolar protein sorting-associated protein 53 homolog OS=Hom	1.540	1.231	1.562	1.043	1.191	1.014	1.374	1.101
Q5JTV1	Glucocorticoid modulatory element binding protein 2, isoform CR/	1.247	1.653	1.345	0.862	1.207	1.446	1.665	1.789
Q8NDV7	Trinucleotide repeat-containing gene 6A protein OS=Homo sapier	1.379	1.402	1.094	0.943	0.962	1.103	1.355	0.925
Q9NQS3	Nectin-3 OS=Homo sapiens GN=PVRL3 PE=1 SV=1 - [PVRL3_HU	1.383	1.804	1.330	1.302	1.388	1.222	1.483	1.444
Q8N3D4	EH domain-binding protein 1-like protein 1 OS=Homo sapiens GN	1.498	1.300	1.243	1.136	1.387	1.091	1.115	0.945
D3DPS3	Arginine/proline rich coiled-coil 1, isoform CRA_b OS=Homo sapie	1.553	1.351	1.566	1.070	1.031	0.960	0.945	1.005
Q9Y3E7	Charged multivesicular body protein 3 OS=Homo sapiens GN=CH	1.366	0.778	1.238	0.988	1.111	1.105	1.259	1.102
Q9NXG6	Transmembrane prolyl 4-hydroxylase OS=Homo sapiens GN=P4H	1.346	1.219	1.118	1.434	1.182	1.364	1.486	1.383
Q9Y2D4	Exocyst complex component 6B OS=Homo sapiens GN=EXOC6B	1.414	1.088	1.546	1.041	1.057	1.167	1.208	1.028
P15144	Aminopeptidase N OS=Homo sapiens GN=ANPEP PE=1 SV=4 - [/	0.767	1.178	0.775	0.843	1.248	1.118	0.823	0.813
Q12802	A-kinase anchor protein 13 OS=Homo sapiens GN=AKAP13 PE=1	1.280	1.064	1.094	0.909	1.061	0.969	1.005	0.821
A8K2F4	cDNA FLJ75620 OS=Homo sapiens PE=2 SV=1 - [A8K2F4_HUMA	1.070	1.265	0.888	1.005	0.899	1.005	1.559	1.456
B4DXW2	cDNA FLJ60947, highly similar to Coiled-coil domain-containing pi	1.704	1.438	1.445	1.189	1.609	1.118	1.744	1.753
Q92674	Centromere protein I OS=Homo sapiens GN=CENPI PE=1 SV=2 -	1.503	1.319	1.086	0.971	1.119	1.266	1.402	1.135
Q8WTS1	1-acylglycerol-3-phosphate O-acyltransferase ABHD5 OS=Homo s	1.495	1.417	1.786	1.374	1.133	1.748	1.632	1.586
Q9H270	Vacuolar protein sorting-associated protein 11 homolog OS=Hom	0.981	0.920	1.149	0.731	1.116	0.976	1.125	1.046
A6H8W8	Intersectin 2 OS=Homo sapiens GN=ITSN2 PE=2 SV=1 - [A6H8V	1.162	1.239	1.800	1.504	1.294	1.364	1.157	1.061
Q9H1C7	Cysteine-rich and transmembrane domain-containing protein 1 O!	1.731	2.313	10.405	4.222	2.190	1.809	2.497	2.122
A0A024R2D7	Transcriptional adaptor 3 (NGG1 homolog, yeast)-like, isoform CR	2.147	2.554	1.979	1.628	1.699	1.329	2.281	1.990
F2Z357	Rap1 GTPase-activating protein 1 OS=Homo sapiens GN=RAP1G/	2.005	1.299	1.653	1.109	1.568	1.320	1.658	1.472
Q86VR2	Protein FAM134C OS=Homo sapiens GN=FAM134C PE=1 SV=1 -	1.515	1.477	1.436	1.314	1.152	1.226	1.531	1.380
Q8NCD3	Holliday junction recognition protein OS=Homo sapiens GN=HJUI	1.101	0.751	0.205	0.089	1.215	0.902	0.549	0.247
I3L1H3	Lipopolysaccharide-induced tumor necrosis factor-alpha factor OS	2.352	1.702	0.731	0.573	2.037	1.069	1.038	0.618
B4DL56	cDNA FLJ55709, highly similar to Polypeptide N-acetylgalactosam	0.910	1.505	1.395	1.306	1.252	1.299	1.704	1.734
H7C1U3	Coiled-coil and C2 domain-containing protein 1B (Fragment) OS=	0.979	0.712	0.810	0.715	1.166	1.080	0.933	0.732
E7EMD6	A-kinase anchor protein 10, mitochondrial OS=Homo sapiens GN=	1.810	1.165	1.107	0.992	1.308	1.163	1.423	0.733
Q75QN2	Integrator complex subunit 8 OS=Homo sapiens GN=INTS8 PE=1	2.755	2.937	2.500	1.800	2.538	1.831	2.986	2.332
B2R6Y2	cDNA, FLJ93173, Homo sapiens sulfite oxidase (SUOX), nuclear g	0.917	0.991	0.887	0.903	1.107	1.161	0.968	0.987
B2RE36	cDNA, FLJ96903 OS=Homo sapiens PE=2 SV=1 - [B2RE36_HUM/	1.153	1.144	0.786	0.775	0.938	1.001	1.160	1.012
A0A024R4V8	G-2 and S-phase expressed 1, isoform CRA_b OS=Homo sapiens	1.517	1.606	1.788	1.182	1.039	1.608	1.531	1.628
Q4G148	Glucoside xylosyltransferase 1 OS=Homo sapiens GN=GXYLT1 PE	1.583	1.536	1.228	1.106	1.419	1.427	1.704	1.302
P50548	ETS domain-containing transcription factor ERF OS=Homo sapien	1.434	1.089	1.027	0.602	1.227	1.038	1.167	0.839
Q9H8M2	Bromodomain-containing protein 9 OS=Homo sapiens GN=BRD9	1.401	1.200	1.732	1.888	1.281	1.408	1.293	1.373
Q9NVE7	Pantothenate kinase 4 OS=Homo sapiens GN=PANK4 PE=1 SV=1	0.971	0.675	1.083	0.943	1.010	0.956	1.082	0.890
D3XNU5	E-cadherin 1 OS=Homo sapiens GN=CDH1 PE=4 SV=1 - [D3XNU	0.596	1.085	0.905	0.952	0.583	0.910	0.688	0.895
O95081	Arf-GAP domain and FG repeat-containing protein 2 OS=Homo sa	1.822	1.095	1.469	2.144	1.713	1.149		
A3KN83	Protein strawberry notch homolog 1 OS=Homo sapiens GN=SBN(	1.105	1.076	0.985	0.660	1.126	0.903	0.887	0.676
H0YDD3	Probable dolichyl pyrophosphate Glc1Man9GlcNAc2 alpha-1,3-glu	3.922	4.411	4.001	3.769	2.489	1.483	3.360	3.262
Q3BDU3	PMS1 protein homolog 1 OS=Homo sapiens GN=PMS1 PE=1 SV=	1.249	1.314	1.376	1.090	1.401	0.970	1.094	1.006

Q9UBU6	Protein FAM8A1 OS=Homo sapiens GN=FAM8A1 PE=1 SV=1 - [F/	0.936	1.368	0.681	1.190	0.829	1.313	1.059	1.092
B2RAR3	Queuine tRNA-ribosyltransferase OS=Homo sapiens PE=2 SV=1 -	1.141	0.791	0.984	1.125	1.191	0.886	0.970	1.177
Q9ULH0	Kinase D-interacting substrate of 220 kDa OS=Homo sapiens GN=	1.421	1.734	1.365	1.267	1.333	1.295	1.542	1.275
Q6IEH8	Transcriptional regulator OS=Homo sapiens GN=NIPBL PE=2 SV=	1.059	1.483	1.197	0.993	0.955	1.154	1.386	1.227
Q2TAA5	GDP-Man:Man(3)GlcNAc(2)-PP-Dol alpha-1,2-mannosyltransferas	1.496	1.283	1.202	1.457	0.721	1.255	1.021	1.044
B4E3L9	cDNA FLJ61578, highly similar to Bromodomain-containing protei	1.391	1.706	1.395	1.683	1.460	1.475	1.777	1.816
Q9NRY4	Rho GTPase-activating protein 35 OS=Homo sapiens GN=ARHGA	1.684	1.261	1.777	1.002	1.595	1.081	1.384	1.189
Q86SQ0	Pleckstrin homology-like domain family B member 2 OS=Homo sa	1.154	1.061	0.856	0.705	0.974	0.915	0.871	0.793
Q8IV63	Inactive serine/threonine-protein kinase VRK3 OS=Homo sapiens	0.967	1.137	1.423	0.723		1.120	0.870	
B4DQK1	cDNA FLJ53475, highly similar to Autophagy-related protein 7 OS	1.315	0.858	1.195	0.914	1.394	1.214	1.323	1.028
H0YAN8	Rho guanine nucleotide exchange factor 10 (Fragment) OS=Hom	1.732	1.040	1.512	1.108	1.479	1.064	1.455	1.051
A7Y9J9	Mucin 5AC, oligomeric mucus/gel-forming OS=Homo sapiens GN=	1.177	0.901	0.944	0.848	1.077	0.980	1.036	0.706
B2RB72	cDNA, FLJ95344, highly similar to Homo sapiens WW domain bin	3.414	2.954	2.418	1.852	2.501	1.372	2.213	1.730
J3QRV5	Spermatogenesis-associated serine-rich protein 2 OS=Homo sapi	1.366	1.329	1.026	0.988	1.119	1.125	1.335	1.031
A8K3Y8	cDNA FLJ76141 OS=Homo sapiens PE=2 SV=1 - [A8K3Y8_HUMA	1.257	0.805	1.139	0.982	0.989	0.827	1.024	0.962
Q9H3S7	Tyrosine-protein phosphatase non-receptor type 23 OS=Homo sa	1.596	1.042	1.362	0.887	1.518	0.975	1.198	1.010
A0A024QZM0	Activating signal cointegrator 1 complex subunit 1, isoform CRA_a	2.034	1.923	1.938	1.431	1.781	1.307	1.836	1.886
Q15172	Serine/threonine-protein phosphatase 2A 56 kDa regulatory subu	1.062	0.430	1.034		0.974	0.898	0.711	0.432
O14732	Inositol monophosphatase 2 OS=Homo sapiens GN=IMPA2 PE=1	1.456	1.301	1.487	1.386	1.392	1.173	1.219	1.399
E7EWC2	Ras GTPase-activating-like protein IQGAP2 (Fragment) OS=Homo	1.005	1.140	1.513	1.132	1.347	1.184	1.408	1.157
G3XA86	Ubiquinone biosynthesis monooxygenase COQ6 OS=Homo sapier	1.410	1.616	1.527	1.618	1.126	1.165	1.228	1.324
D3DU39	Chromosome 3 open reading frame 38, isoform CRA_b OS=Homo	0.525	0.543	0.616	0.418	0.800	0.951	0.736	0.589
Q86UT6	NLR family member X1 OS=Homo sapiens GN=NLRX1 PE=1 SV=	1.446	1.862	1.601	1.256	1.133	1.236	1.704	1.266
B4DER6	cDNA FLJ51800, highly similar to Peroxisome biogenesis factor 1	1.273	1.066	1.008	1.079	1.205	1.061	1.099	1.429
Q92830	Histone acetyltransferase KAT2A OS=Homo sapiens GN=KAT2A P	1.373	2.050	1.213	1.198	1.396	1.792	1.722	1.273
A0A087WVA8	Testis-expressed sequence 2 protein OS=Homo sapiens GN=TEX:	1.180	1.567	1.329	1.191	0.997	1.331	1.433	1.489
Q8IX18	Probable ATP-dependent RNA helicase DHX40 OS=Homo sapiens	1.667	1.864	1.680	0.972	1.573	1.152	1.607	1.559
A8K822	cDNA FLJ77778, highly similar to Homo sapiens death-associated	0.785	0.791	0.423	0.392	0.769	1.048	0.659	0.527
O95749	Geranylgeranyl pyrophosphate synthase OS=Homo sapiens GN=(	1.232	0.867	1.213	0.975	1.193	0.959	1.041	1.077
Q96JP2	Unconventional myosin-XVB OS=Homo sapiens GN=MYO15B PE=	1.292	1.150	0.923	0.654	1.163	1.166	1.023	0.683
Q9H993	UPF0364 protein C6orf211 OS=Homo sapiens GN=C6orf211 PE=	1.214	0.813	1.324	1.179	1.002	1.038	1.014	1.146
Q8IWI9	MAX gene-associated protein OS=Homo sapiens GN=MGA PE=1	1.270	1.735	1.149	0.935	1.110	1.475	1.370	0.981
Q16769	Glutaminyl-peptide cyclotransferase OS=Homo sapiens GN=QPCT	1.464	1.535	1.338		1.504	1.550	2.054	0.830
H3BNF0	Cytosolic Fe-S cluster assembly factor NUBP2 OS=Homo sapiens	1.105	0.769	1.173	1.045	1.100	0.860	1.165	1.023
P57081	tRNA (guanine-N(7)-)-methyltransferase non-catalytic subunit WE	1.590	1.201	1.323	1.053	1.740	0.963	1.003	0.949
O15357	Phosphatidylinositol 3,4,5-trisphosphate 5-phosphatase 2 OS=Ho	1.382	1.003	1.289	0.759	1.258	1.029	1.096	0.951
A8K313	cDNA FLJ78249, highly similar to Homo sapiens RAD51 associate	1.368	1.273	0.943	0.710	0.908	1.043	0.773	0.858
B4E272	cDNA FLJ56417, highly similar to F-box only protein 11 OS=Homo	1.465	1.326	1.175	1.042	1.290	1.290	1.484	1.140
B1AQP1	Upstream stimulatory factor 1 OS=Homo sapiens GN=USF1 PE=4	3.327	5.177	4.947	4.510	2.254	1.751	3.592	5.317
Q16635	Tafazzin OS=Homo sapiens GN=TAZ PE=1 SV=1 - [TAZ_HUMAN]	2.272	2.570	1.553	1.659	1.880	1.524	1.917	1.920

P42356	Phosphatidylinositol 4-kinase alpha OS=Homo sapiens GN=PI4KA	1.205	1.288	1.180	1.333	1.187	1.097	0.968	1.138
Q8IZH2	5'-3' exoribonuclease 1 OS=Homo sapiens GN=XRN1 PE=1 SV=1	0.903	1.189	1.101	0.837	1.019	1.053	1.120	0.992
O15541	RING finger protein 113A OS=Homo sapiens GN=RNF113A PE=1	1.276	1.144	1.017	0.776	1.291	1.273	1.269	0.949
O94864	STAGA complex 65 subunit gamma OS=Homo sapiens GN=SUPT	1.812	2.101			1.823	2.225	1.968	1.887
Q96JB2	Conserved oligomeric Golgi complex subunit 3 OS=Homo sapiens	1.507	1.342	1.238	0.918	1.234	1.101	1.038	1.008
B4DUF8	cDNA FLJ52165, highly similar to Ubiquitin-conjugating enzyme E	1.515	1.876	1.908	1.567	1.392	2.112	1.826	2.017
H7C1I0	Solute carrier family 35 member E1 (Fragment) OS=Homo sapien	1.718	2.302	1.843	1.617	1.386	1.240	1.873	1.897
A0PJ54	PEX12 protein (Fragment) OS=Homo sapiens GN=PEX12 PE=2 S	1.201	1.554	1.251	1.006	0.943	1.449	1.327	1.202
Q16512	Serine/threonine-protein kinase N1 OS=Homo sapiens GN=PKN1	1.513	1.036	1.507	1.148	1.128	1.078	1.287	1.271
B4DKZ1	cDNA FLJ55090 OS=Homo sapiens PE=2 SV=1 - [B4DKZ1_HUMA	0.961	0.922	0.877	0.768	0.889	1.107	0.905	0.752
P13647	Keratin, type II cytoskeletal 5 OS=Homo sapiens GN=KRT5 PE=1	2.117	2.407	2.432	2.074	1.455	1.760	3.514	1.477
A7MD03	CCDC132 protein OS=Homo sapiens GN=CCDC132 PE=2 SV=1 -	1.997	1.547	2.039	1.501	1.686	1.273	1.883	1.906
A8K6A5	cDNA FLJ77742, highly similar to Homo sapiens integrin, alpha 5	0.970	1.643	1.279	1.512	1.176	1.230	1.253	1.170
A1L3A9	TBC1 domain family, member 9B (With GRAM domain) OS=Homo	2.296	1.073	1.612	1.693	1.938	1.068	2.003	1.115
O14933	Ubiquitin/ISG15-conjugating enzyme E2 L6 OS=Homo sapiens GN	1.150	0.775	1.420	1.131	1.100	0.922	1.060	1.180
Q8N2M8	CLK4-associating serine/arginine rich protein OS=Homo sapiens G	1.670	1.713	1.490	0.916	1.796	1.345	1.450	1.126
P23458	Tyrosine-protein kinase JAK1 OS=Homo sapiens GN=JAK1 PE=1 S	1.214	1.280	0.667	0.466	1.321	1.128	0.809	0.597
F8VRE8	Transmembrane protein 19 (Fragment) OS=Homo sapiens GN=T	1.376	1.571	1.145	1.155	1.144	1.215	1.320	1.187
B3KXX3	Solute carrier family 12 member 6 OS=Homo sapiens GN=SLC12	1.838	2.314	1.813	2.142	1.129	1.496	2.114	1.710
E7EST9	Ubiquitin carboxyl-terminal hydrolase OS=Homo sapiens GN=USP	1.092	1.105	1.088	0.921	1.038	1.129	1.018	0.985
Q9BXB4	Oxysterol-binding protein-related protein 11 OS=Homo sapiens G	1.132	0.869	1.160	0.876	1.082	0.986	1.154	1.212
Q9P206	Uncharacterized protein KIAA1522 OS=Homo sapiens GN=KIAA1	1.450	1.070	1.048	0.669	1.324	0.948	0.874	0.951
B2RBH2	cDNA, FLJ95508, highly similar to Homo sapiens 5'-nucleotidase,	0.739	1.011	0.825	0.818	0.847	0.972	0.628	0.614
A0AVG7	Breast carcinoma amplified sequence 1 OS=Homo sapiens GN=B	1.422	1.061	1.249	1.014	1.189	1.192	1.248	0.968
Q8WV74	Nucleoside diphosphate-linked moiety X motif 8, mitochondrial O	0.795		1.327	1.203	0.812	1.112	1.434	1.225
B5M450	Solute carrier family 4 sodium bicarbonate cotransporter member	1.896	2.000	1.340	1.200	1.530	1.026	1.328	1.230
F8WDV0	Importin-11 OS=Homo sapiens GN=IPO11 PE=1 SV=1 - [F8WDV	3.133	2.481	2.642	2.126	2.462	1.422	2.480	2.542
Q12959	Disks large homolog 1 OS=Homo sapiens GN=DLG1 PE=1 SV=2	1.581	2.068	1.832	1.718	1.311	1.299	1.592	2.049
Q8TBR7	Protein FAM57A OS=Homo sapiens GN=FAM57A PE=1 SV=2 - [F	3.525	5.017	4.101	3.993	2.492	1.549	3.857	3.252
Q8IVQ8	XPO4 protein (Fragment) OS=Homo sapiens GN=XPO4 PE=2 SV=	2.094	1.399	1.934	1.446	1.863	1.169	1.811	1.641
Q6ZN55	Zinc finger protein 574 OS=Homo sapiens GN=ZNF574 PE=1 SV=	1.293	1.285	1.019	0.729	1.134	1.337	1.334	1.267
Q9Y666	Solute carrier family 12 member 7 OS=Homo sapiens GN=SLC12	1.071	1.701	1.275	1.409	1.007	1.257	1.403	1.353
Q8ND04	Protein SMG8 OS=Homo sapiens GN=SMG8 PE=1 SV=1 - [SMG8	1.353	1.844	1.675	1.822	1.125	1.274	1.353	0.827
A8K8X5	cDNA FLJ76447, highly similar to Homo sapiens cell division cycle	4.394	5.354	3.007	1.450	3.270	2.149	4.488	2.786
Q9UQ53	Alpha-1,3-mannosyl-glycoprotein 4-beta-N-acetylglucosaminyltrar	0.930	1.182	1.430	1.025	0.849	1.085	0.986	1.099
Q9H8K7	Uncharacterized protein C10orf88 OS=Homo sapiens GN=C10orf8	1.824	2.183	1.755	1.596	1.808	1.610	1.716	2.010
Q03111	Protein ENL OS=Homo sapiens GN=MLLT1 PE=1 SV=2 - [ENL_HU		0.740	0.537	0.560	0.871	1.760	1.014	0.806
Q8TB96	T-cell immunomodulatory protein OS=Homo sapiens GN=ITFG1 F	1.161	1.236	1.119	1.127	0.833	1.122	1.045	1.058
H3BS42	Zinc finger protein 768 OS=Homo sapiens GN=ZNF768 PE=1 SV=						1.120		
Q9NR09	Baculoviral IAP repeat-containing protein 6 OS=Homo sapiens GN	1.375	1.036	1.339	1.040	1.223	1.090	1.314	0.958

Q6N069	N-alpha-acetyltransferase 16, NatA auxiliary subunit OS=Homo sa	1.568	1.888	1.807	1.266	1.354	1.505	1.131	1.582
W6I206	Lysyl oxidase-like 2 delta e13 OS=Homo sapiens GN=LOXL2 PE=:	1.765	0.948	1.012	0.681	1.453	1.217	1.343	0.635
Q8TDB6	E3 ubiquitin-protein ligase DTX3L OS=Homo sapiens GN=DTX3L	1.237	1.141	1.059	0.828	1.047	1.307	1.212	1.006
B4DRN8	Palmitoyltransferase OS=Homo sapiens GN=ZDHHC20 PE=1 SV=	0.973	1.315	0.897	0.799	0.945	1.164	1.168	1.056
B7Z168	Neuregulin 1 isoform HRG-alpha (Fragment) OS=Homo sapiens G	1.242	0.896	0.488	0.272	0.823	0.830	0.517	0.387
P54274	Telomeric repeat-binding factor 1 OS=Homo sapiens GN=TERF1 I	1.670	2.814	2.346	1.769	1.520	1.738	2.364	1.652
J3QKD2	Zinc finger MYND domain-containing protein 11 OS=Homo sapier	1.214	1.891	1.438	1.077	1.404	1.388	1.800	1.429
Q8IY22	C-Maf-inducing protein OS=Homo sapiens GN=CMIP PE=1 SV=3	1.522	0.988	0.937	0.585	1.370	1.014	0.877	0.741
Q9HBR0	Putative sodium-coupled neutral amino acid transporter 10 OS=H	1.198	1.529	1.170	1.135	1.141	1.144	1.224	1.042
P53609	Geranylgeranyl transferase type-1 subunit beta OS=Homo sapien	1.260	0.851	1.114	0.907	1.212	1.150	1.232	1.026
B4DSW6	cDNA FLJ58422, highly similar to HIRA protein OS=Homo sapiens	0.897	1.444	0.958	0.667	1.371	1.416	1.461	1.259
A2AAT0	Zinc transporter SLC39A7 (Fragment) OS=Homo sapiens GN=SLC	1.503	1.027	1.132	0.987	1.149	1.112	1.286	0.973
O15047	Histone-lysine N-methyltransferase SETD1A OS=Homo sapiens G	1.234	1.181	0.793	0.625	1.116	1.219	1.102	0.979
B2RCZ4	Protein kinase C OS=Homo sapiens PE=2 SV=1 - [B2RCZ4_HUMA	1.938	1.204	1.509	1.144	1.780	0.995	1.552	1.384
A0A024R412	Neuropilin 2, isoform CRA_c OS=Homo sapiens GN=NRP2 PE=4 S	1.005	1.206	0.893	0.548	1.138	1.040	0.831	0.669
E9PRK7	ARL14 effector protein (Fragment) OS=Homo sapiens GN=ARL14	1.640	1.774	1.252	1.199	2.063	1.468	1.735	1.092
Q5JTD5	cDNA FLJ50818, highly similar to Protein YIPF3 OS=Homo sapien	1.333	1.357	1.048	1.337	1.141	1.235	1.085	1.353
Q2KHR3	Glutamine and serine-rich protein 1 OS=Homo sapiens GN=QSER	1.140	1.034	0.704	0.447	1.265	1.093	0.802	0.688
Q9HAU5	Regulator of nonsense transcripts 2 OS=Homo sapiens GN=UPF2	1.353	1.232	1.364	0.882	1.173	1.169	1.256	1.135
Q6NSC7	STX17 protein (Fragment) OS=Homo sapiens GN=STX17 PE=2 S	1.056	1.196	1.043	1.021	1.061	1.125	1.050	1.044
B4DMI6	WDR45-like, isoform CRA_f OS=Homo sapiens GN=WDR45L PE=	2.770	2.203	2.968	2.234	1.964	1.662	2.268	1.681
P20749	B-cell lymphoma 3 protein OS=Homo sapiens GN=BCL3 PE=1 SV	1.296	0.736			1.130	0.802		
O43422	52 kDa repressor of the inhibitor of the protein kinase OS=Homo	1.157	1.195	1.259	1.059	0.917	1.326	1.304	1.579
O94822	E3 ubiquitin-protein ligase listerin OS=Homo sapiens GN=LTN1 P	1.939	1.653	1.917	1.356	1.789	1.392	1.875	1.530
Q14147	Probable ATP-dependent RNA helicase DHX34 OS=Homo sapiens	1.630	1.595	1.203	1.240	1.428	1.278	1.746	1.143
P04066	Tissue alpha-L-fucosidase OS=Homo sapiens GN=FUCA1 PE=1 SV	1.387	1.495	1.618	1.783	1.672	1.321	1.824	1.744
F8WF49	Disks large-associated protein 4 OS=Homo sapiens GN=DLGAP4	1.675	1.094	1.235	0.779	1.658	0.960	1.205	1.008
Q8IVQ6	Palmitoyltransferase ZDHHC21 OS=Homo sapiens GN=ZDHHC21	1.563	2.254	1.644	1.834	1.727	1.482	2.444	2.011
Q9NYR9	NF-kappa-B inhibitor-interacting Ras-like protein 2 OS=Homo sap	1.315	0.873	0.742	0.621	1.215	1.118	1.066	0.944
Q9UK73	Protein fem-1 homolog B OS=Homo sapiens GN=FEM1B PE=1 SV	2.675	2.351	1.678	1.168	1.837	1.651	1.266	1.078
C9JCN8	Fos-related antigen 2 (Fragment) OS=Homo sapiens GN=FOSL2 I	1.197	1.196	0.744	0.615	1.147	0.919	1.053	0.970
Q9H4H8	Protein FAM83D OS=Homo sapiens GN=FAM83D PE=1 SV=3 - [F	1.545	1.048	0.756	0.543	1.223	1.002	1.102	0.812
Q96PY5	Formin-like protein 2 OS=Homo sapiens GN=FMNL2 PE=1 SV=3	1.182	1.305	1.102	0.938	0.898	1.136	1.041	0.917
Q8ND24	RING finger protein 214 OS=Homo sapiens GN=RNF214 PE=1 SV	1.161	1.025	1.053	1.091	1.193	1.068	1.347	1.205
B4DEQ6	cDNA FLJ58501, highly similar to Homo sapiens PAX interacting (	1.048	1.291	1.117	0.941	1.270	1.454	1.499	1.391
Q9NPB8	Glycerophosphocholine phosphodiesterase GPCPD1 OS=Homo sa	1.366	1.034	0.838	0.944	1.197	0.690	0.703	0.757
Q96K76	Ubiquitin carboxyl-terminal hydrolase 47 OS=Homo sapiens GN=U	1.570	1.029	1.629	1.347	1.459	1.060	1.429	1.392
Q9H7E2	Tudor domain-containing protein 3 OS=Homo sapiens GN=TDRD:	1.182	1.076	1.139	0.739	0.976	1.051	1.001	0.922
Q9UNW9	RNA-binding protein Nova-2 OS=Homo sapiens GN=NOVA2 PE=1	1.182	1.605	1.268	1.315	0.917	1.295	1.479	1.712
Q59F54	Solute carrier family 2 (Facilitated glucose transporter), member 3	1.376	1.413	1.241	1.238	1.305	1.087	1.200	1.156

Q9UBH6	Xenotropic and polytropic retrovirus receptor 1 OS=Homo sapiens	2.200	2.524	2.513	2.346	2.227	1.327	2.283	2.629
A0A024R223	Vacuolar protein sorting 13A (Yeast), isoform CRA_c OS=Homo sa	1.231	1.279	1.341	1.338	1.182	1.217	1.533	1.567
Q86V59	PNMA-like protein 1 OS=Homo sapiens GN=PNMAL1 PE=1 SV=2	1.348	2.895	2.671	2.411	1.349	2.466	2.151	2.138
B1Q3L0	Tripartite motif-containing protein 5 alpha OS=Homo sapiens GN=	0.949	1.074	0.743	0.969	1.196	1.486	0.865	0.954
Q7Z4S6	Kinesin-like protein KIF21A OS=Homo sapiens GN=KIF21A PE=1	1.573	1.111	1.191	1.122	1.196	1.039	1.219	0.909
O94927	HAUS augmin-like complex subunit 5 OS=Homo sapiens GN=HAU	1.231	0.875	0.946	0.899	1.094	1.108	1.026	0.906
P78310	Coxsackievirus and adenovirus receptor OS=Homo sapiens GN=C	1.255	1.569	0.978	1.171	1.267	1.201	1.111	1.287
B4DSF4	cDNA FLJ58382, highly similar to Zinc finger protein 8 OS=Homo	0.982	1.232	1.022	0.742	1.093	1.453	1.465	1.171
Q03154	Aminoacylase-1 OS=Homo sapiens GN=ACY1 PE=1 SV=1 - [ACY	1.040	0.781	1.220	0.981	0.942	0.926	0.877	0.938
Q99766	ATP synthase subunit s, mitochondrial OS=Homo sapiens GN=AT	0.680	0.745	0.723	0.755	0.711	1.151	0.838	0.799
Q96J02	E3 ubiquitin-protein ligase Itchy homolog OS=Homo sapiens GN=	1.324	1.260	0.677	0.459	1.340	1.164	0.892	0.344
P42695	Condensin-2 complex subunit D3 OS=Homo sapiens GN=NCAPD3	1.519	1.828	1.305	0.910	1.374	1.681	1.347	1.286
A0A024R1D9	Neurofibromin 2 (Bilateral acoustic neuroma), isoform CRA_d OS=	0.831	0.867	0.684	0.462	0.836	1.176	0.833	1.241
Q9NXW9	Alpha-ketoglutarate-dependent dioxygenase alkB homolog 4 OS=	1.358	1.295	1.586	1.105	1.335	1.577	1.689	1.444
A5YP36	SEC15-like protein 3 OS=Homo sapiens GN=SEC15L3 PE=2 SV=1	1.648	1.280	1.742	1.241	1.582	1.236	1.506	1.294
X6R717	Putative pre-mRNA-splicing factor ATP-dependent RNA helicase D	1.181	0.699	0.911	0.677	1.127	1.009	0.943	0.822
Q6IE81	Protein Jade-1 OS=Homo sapiens GN=JADE1 PE=1 SV=1 - [JADE	0.835	0.882	0.496	0.327	0.917	0.883	0.723	0.528
Q6I9T1	Mothers against decapentaplegic homolog OS=Homo sapiens GN	1.217	0.995	0.918	0.681	1.010	1.515	1.226	1.394
Q8N3E9	1-phosphatidylinositol 4,5-bisphosphate phosphodiesterase delta-	1.193	1.467	0.913	0.949	1.227	1.104	1.072	1.042
O14757	Serine/threonine-protein kinase Chk1 OS=Homo sapiens GN=CHE	0.577	0.430	0.458	0.285	0.544	1.009	0.532	0.307
Q9H2H9	Sodium-coupled neutral amino acid transporter 1 OS=Homo sapie	3.279	4.097	2.743	2.785	2.677	1.695	2.772	3.016
Q5H9P2	Intraflagellar transport protein 43 homolog (Fragment) OS=Homo	2.137	2.492	2.569	1.360	1.542	2.249	2.062	1.886
Q96GX5	Serine/threonine-protein kinase greatwall OS=Homo sapiens GN=	0.974	0.780	0.919	0.597	1.059	0.939	0.878	0.625
Q9P2B2	Prostaglandin F2 receptor negative regulator OS=Homo sapiens G	1.104	1.569	1.103	1.633	0.994	1.018	1.272	1.531
A0A024R1H0	Uncharacterized protein OS=Homo sapiens GN=RP5-1119A7.4 PE	1.479	1.612	1.294	0.997	1.465	1.268	1.541	1.409
B2RDI0	cDNA, FLJ96618, highly similar to Homo sapiens tyrosyl-DNA pho	1.206	1.073	1.341	0.840	1.283	1.230	0.885	0.878
B3KNH6	cDNA FLJ14631 fis, clone NT2RP2000660, highly similar to Homo	2.053	1.842	1.869	1.119	1.554	1.288	1.795	1.815
Q08AD1	Calmodulin-regulated spectrin-associated protein 2 OS=Homo sap	1.301	1.058	1.115	0.951	1.044	0.986	1.155	1.032
B2R932	cDNA, FLJ94187, highly similar to Homo sapiens CD99 antigen (C	2.679	4.989	3.846	3.361	1.827	2.039	3.542	3.631
Q68CS2	Putative uncharacterized protein DKFZp781P1796 OS=Homo sapi	1.648	1.503	1.778	1.319	1.516	1.534	1.715	1.724
Q9UNF3	DNA polymerase OS=Homo sapiens GN=POLE1 PE=3 SV=1 - [Q9	1.545	1.471	1.789	1.297	1.207	1.402	1.462	1.419
A0A024R680	AT rich interactive domain 4A (RBP1-like), isoform CRA_b OS=Ho	1.263	1.180	0.688	0.458	1.199	0.929	1.083	0.654
Q8NBL1	Protein O-glucosyltransferase 1 OS=Homo sapiens GN=POGLUT1	1.580	1.179	1.465	1.171	1.674	1.236	1.636	1.552
Q95IC0	MHC class I chain-related protein A (Fragment) OS=Homo sapien	1.702	2.445	2.126	2.053	1.639	1.750	2.794	2.455
Q9HD47	Ran guanine nucleotide release factor OS=Homo sapiens GN=RA	1.141	1.118	1.831	1.456	1.044	1.063	1.255	1.241
A6NGW1	Protein YIF1A OS=Homo sapiens GN=YIF1A PE=1 SV=1 - [A6NG	2.527	2.669	2.296	1.893	1.771	1.478	2.172	2.048
Q8NEB9	Phosphatidylinositol 3-kinase catalytic subunit type 3 OS=Homo s	1.258	0.925	1.065	0.805	1.301	1.070	1.152	0.965
O95396	Adenylyltransferase and sulfurtransferase MOCS3 OS=Homo sapi	1.178	0.899	1.021	1.027	1.178	1.051	0.993	0.955
Q9BRK4	Leucine zipper putative tumor suppressor 2 OS=Homo sapiens GI	1.469	1.778	0.853	0.865	1.256	1.290	1.347	1.156
Q8IZ21	Phosphatase and actin regulator 4 OS=Homo sapiens GN=PHACT	1.458	1.031	1.275	0.842	1.160	0.976	1.315	0.813

Q9H7Z3	Protein NRDE2 homolog OS=Homo sapiens GN=NRDE2 PE=1 SV	1.066	1.383	1.075	0.877	1.263	1.349	1.446	0.709
A8K3W7	cDNA FLJ76271, highly similar to Homo sapiens protein O-fucosyl	2.057	1.373	2.135	1.809	1.560	1.316	1.773	1.997
Q9BVS4	Serine/threonine-protein kinase RIO2 OS=Homo sapiens GN=RIC	2.014	1.279	1.365	1.270	1.287	1.369	1.662	1.097
Q93100	Phosphorylase b kinase regulatory subunit beta OS=Homo sapier	1.158	0.838	1.154	0.841	1.219	0.753	0.887	0.754
O15397	Importin-8 OS=Homo sapiens GN=IPO8 PE=1 SV=2 - [IPO8_HUI	1.806	1.266	1.587	1.269	1.436	1.322	1.792	1.429
Q8N9N5	Protein BANP OS=Homo sapiens GN=BANP PE=1 SV=3 - [BANP_	1.081	1.725	1.108	1.246	1.234	1.379	1.297	1.439
J3QQJ5	Trafficking protein particle complex subunit 8 OS=Homo sapiens (	1.259	0.975	1.159	1.134	0.971	1.109	1.258	1.109
Q86TJ2	Transcriptional adapter 2-beta OS=Homo sapiens GN=TADA2B PE	1.390	1.099	1.217	1.092	1.431	1.426	1.276	1.247
Q8IYL3	UPF0688 protein C1orf174 OS=Homo sapiens GN=C1orf174 PE=	1.345	1.485	1.012	0.686	1.237	1.317	1.306	0.998
G3V167	Poly (ADP-ribose) polymerase family, member 2, isoform CRA_a (	1.299	1.708	1.213	0.978	1.333	1.426	1.374	1.122
A0A024RE13	Ribonuclease III, nuclear, isoform CRA_a OS=Homo sapiens GN=	1.053	1.432	1.185	1.070	1.200	1.464	1.703	1.206
Q9BSK2	Solute carrier family 25 member 33 OS=Homo sapiens GN=SLC2!					2.083	1.315		1.198
Q8IWC1	MAP7 domain-containing protein 3 OS=Homo sapiens GN=MAP7I	1.520	1.726	1.763	1.330	1.197	1.446	1.418	1.804
Q5T7W0	Zinc finger protein 618 OS=Homo sapiens GN=ZNF618 PE=1 SV=	2.434	3.119	2.061	1.122	2.207	1.016	2.163	0.925
Q8N4C8	Misshapen-like kinase 1 OS=Homo sapiens GN=MINK1 PE=1 SV=	1.125	1.320	1.150	0.871	0.963	1.070	1.116	1.228
B3KRQ2	cDNA FLJ34689 fis, clone MESAN2000815, highly similar to Homo	1.317	1.582	1.186	0.819	0.981	1.001	1.578	1.117
Q96AP0	Adrenocortical dysplasia protein homolog OS=Homo sapiens GN=	1.271	1.286	1.313	0.892	1.224	1.265	1.275	0.870
Q9H6U8	Alpha-1,2-mannosyltransferase ALG9 OS=Homo sapiens GN=ALG	0.692	0.718	0.583	0.600	1.147	1.256	0.963	0.715
Q3KQZ1	Solute carrier family 25 member 35 OS=Homo sapiens GN=SLC2!	1.148	1.266	0.923	0.752	0.886	0.953	0.944	0.724
Q6NXT6	Transmembrane anterior posterior transformation protein 1 homo	1.212	1.319	1.144	0.649	1.015	1.219	1.127	1.132
Q96J84	Kin of IRRE-like protein 1 OS=Homo sapiens GN=KIRREL PE=1 S	1.035	1.508	0.952	0.880	1.199	1.470	1.300	1.356
Q9Y6X3	MAU2 chromatid cohesion factor homolog OS=Homo sapiens GN=	1.259	1.793	1.392	1.053	1.134	1.314	1.365	1.443
Q14571	Inositol 1,4,5-trisphosphate receptor type 2 OS=Homo sapiens GI	1.375	1.625	1.651	1.274	1.152	1.196	1.829	1.628
A8K2Q6	Peptidyl-prolyl cis-trans isomerase OS=Homo sapiens PE=2 SV=1	1.438	0.866	1.184	1.035	1.714	1.184	1.212	1.276
B4E0M5	cDNA FLJ58823, highly similar to Thromboxane-A synthase (EC 5	2.240	3.592	1.743	1.847	1.522	1.659	2.218	3.207
A0A024RCT1	TAP binding protein (Tapasin), isoform CRA_c OS=Homo sapiens	1.293	0.817	0.529	0.468	1.093	0.861	0.702	0.492
Q5T0N5	Formin-binding protein 1-like OS=Homo sapiens GN=FNBP1L PE=	1.558	1.258	1.278	0.960	1.553	1.096	1.353	1.272
Q8N5F7	NF-kappa-B-activating protein OS=Homo sapiens GN=NKAP PE=1	2.589	3.243	1.976	1.907	2.290	1.291	1.889	1.823
Q92995	Ubiquitin carboxyl-terminal hydrolase 13 OS=Homo sapiens GN=U	1.370	1.107	1.124	0.745	1.091	0.876	1.118	1.019
O95155	Ubiquitin conjugation factor E4 B OS=Homo sapiens GN=UBE4B I	1.705	1.240	1.242	1.120	1.574	1.207	1.387	1.091
B4DF30	cDNA FLJ60065, highly similar to Solute carrier family 12 membe	0.989	0.978	0.985	0.895	0.888	1.164	0.999	1.009
B2R7D3	cDNA, FLJ93390, highly similar to Homo sapiens RALBP1 associat	1.230	0.894	1.204	0.979	1.433	0.923	1.397	1.033
H0YK36	Dipeptidyl peptidase 8 (Fragment) OS=Homo sapiens GN=DPP8 I	2.107	1.517	1.811	1.368	1.778	1.266	1.681	1.451
B4DHI5	cDNA FLJ51844, highly similar to Serine incorporator 3 OS=Homo	2.374	2.597	1.399	1.203	2.121	1.654	2.063	1.514
Q96PZ2	Protein FAM111A OS=Homo sapiens GN=FAM111A PE=1 SV=2 -	1.659	1.338	1.110	1.627	1.400	1.590	1.629	1.551
Q2TAK2	PHD finger protein 12 OS=Homo sapiens GN=PHF12 PE=1 SV=1	0.675	1.096	0.921	0.668	0.714	0.848	0.889	0.696
Q92484	Acid sphingomyelinase-like phosphodiesterase 3a OS=Homo sapi	1.178	1.053	0.796	0.907	1.070	1.276	1.193	1.423
D3DPA6	WD repeat, sterile alpha motif and U-box domain containing 1, is	1.495	1.338	1.683	1.689	1.546	1.366	2.017	1.697
Q8N573	Oxidation resistance protein 1 OS=Homo sapiens GN=OXR1 PE=1	2.647	1.731	1.928	1.216	2.192	1.905	2.223	2.236
B4E1W4	cDNA FLJ55383 OS=Homo sapiens PE=2 SV=1 - [B4E1W4_HUM/	1.316	1.396	0.663	0.591	1.144	1.291	1.252	0.977

A1L390	Pleckstrin homology domain-containing family G member 3 OS=H	2.080	2.386	1.680	1.321	1.921	1.540	1.751	1.568
Q16773	Kynurenine--oxoglutarate transaminase 1 OS=Homo sapiens GN=					1.461		0.908	0.732
P24311	Cytochrome c oxidase subunit 7B, mitochondrial OS=Homo sapie	1.604	1.826	1.556	1.541	1.212	1.198	1.724	1.575
H0Y6H0	Lysine-specific histone demethylase 1B (Fragment) OS=Homo sap						1.070		
Q9Y496	Kinesin-like protein KIF3A OS=Homo sapiens GN=KIF3A PE=1 SV	1.342	0.838	0.996	0.745	1.026	1.038	0.988	0.799
Q96L93	Kinesin-like protein KIF16B OS=Homo sapiens GN=KIF16B PE=1	1.178	1.269	1.324	1.087	1.177	1.022	1.133	1.059
Q8TDW0	Volume-regulated anion channel subunit LRRC8C OS=Homo sapie	1.303	1.891	1.493	1.630	1.130	1.408	1.429	1.559
Q96MY1	Nucleolar protein 4-like OS=Homo sapiens GN=NOL4L PE=1 SV=	1.379	1.229	0.794	0.421	1.223	1.076	1.118	0.911
Q5MJ32	Protection of telomeres protein 1 variant 5 OS=Homo sapiens PE=	0.254		0.418		0.655	1.140	0.519	0.325
A8K6H9	cDNA FLJ75876, highly similar to Homo sapiens solute carrier fam	1.877	1.412	0.863	0.808	1.913	0.892	1.105	0.972
Q96NB2	Sideroflexin-2 OS=Homo sapiens GN=SFXN2 PE=1 SV=2 - [SFXN	2.137	1.966	2.308	2.157	1.738	1.174	2.205	2.145
O15020	Spectrin beta chain, non-erythrocytic 2 OS=Homo sapiens GN=SI	1.211	1.447	1.242	1.077	1.087	1.022	1.678	1.673
Q9UMN6	Histone-lysine N-methyltransferase 2B OS=Homo sapiens GN=KM	1.526	1.810	1.488	1.334	1.231	1.372	1.479	1.661
Q5H8A4	GPI ethanolamine phosphate transferase 2 OS=Homo sapiens GN	1.915	1.840	1.831	1.532	1.254	1.704	1.927	1.082
B4DSC2	Elongation of very long chain fatty acids protein OS=Homo sapier	3.298	3.635	3.116	2.792	2.354	1.448	3.339	2.714
B4DJK7	cDNA FLJ56648, highly similar to Homo sapiens acyl-CoA synthet	1.103	1.112	0.942	1.055	0.865	1.056	0.885	0.870
Q96S06	Lipase maturation factor 1 OS=Homo sapiens GN=LMF1 PE=1 SV	1.490	1.551	1.540	1.411	1.051	1.145	1.371	1.279
Q14814	Myocyte-specific enhancer factor 2D OS=Homo sapiens GN=MEF	1.288	0.952	0.528	0.304	1.406	1.029	0.764	0.504
B7ZM65	PHRF1 protein OS=Homo sapiens GN=PHRF1 PE=2 SV=1 - [B7ZI	1.642	2.090	1.124	0.848	1.431	1.573	1.413	1.097
A0A075B7F8	Nuclear envelope pore membrane protein POM 121C OS=Homo s	0.980	1.135	0.878	0.704	0.823	1.244	1.101	0.850
A8K7F7	cDNA FLJ76913, highly similar to Homo sapiens F-box protein 7 (	1.107	0.773	0.888	0.586	1.040	0.909	0.831	0.656
O15118	Niemann-Pick C1 protein OS=Homo sapiens GN=NPC1 PE=1 SV=	1.653	1.266	1.020	1.001	1.809	1.106	1.278	1.001
O43896	Kinesin-like protein KIF1C OS=Homo sapiens GN=KIF1C PE=1 SV	1.143	1.060	0.869	0.701	0.991	1.162	1.450	0.721
A6NJ78	Probable methyltransferase-like protein 15 OS=Homo sapiens GN	0.577	0.603	0.550	0.827	0.709	1.064	0.948	0.960
O75970	Multiple PDZ domain protein OS=Homo sapiens GN=MPDZ PE=1	1.247	1.402	1.168	1.034	1.301	1.282	1.206	1.133
A8K2T5	cDNA FLJ77047, highly similar to Homo sapiens zinc finger protei	0.911	0.798	1.119	2.678	0.986	0.754	0.532	1.047
Q69YS0	Putative uncharacterized protein DKFZp313K2110 (Fragment) OS	1.728	2.264	1.873	1.703	1.761	2.093	2.109	1.818
O14545	TRAF-type zinc finger domain-containing protein 1 OS=Homo sap						1.050	1.188	
Q96BU1	S100P-binding protein OS=Homo sapiens GN=S100PBP PE=1 SV	1.538	1.576	0.565		1.616	1.591	1.065	0.570
Q13563	Polycystin-2 OS=Homo sapiens GN=PKD2 PE=1 SV=3 - [PKD2_H	2.759	2.488	2.132	1.810	1.873	1.183	2.440	1.958
A0A024R8A5	Outer dense fiber of sperm tails 2, isoform CRA_a OS=Homo sapi					1.026	1.166	1.001	0.825
Q9H330	Transmembrane protein 245 OS=Homo sapiens GN=TMEM245 PE	2.516	2.695	2.375	2.355	1.980	1.446	2.254	2.286
O75882	Attractin OS=Homo sapiens GN=ATRN PE=1 SV=2 - [ATRN_HUM	1.316	1.616	1.562	1.429	1.422	1.426	1.820	1.450
Q8TD22	Sideroflexin-5 OS=Homo sapiens GN=SFXN5 PE=2 SV=1 - [SFXN	1.238	1.107	1.135	0.904	0.882	1.200	1.139	1.072
Q8WWI5	Choline transporter-like protein 1 OS=Homo sapiens GN=SLC44A	1.104	1.207	0.965	0.809	1.162	1.146	1.044	0.881
A0A024R0X0	AP1 gamma subunit binding protein 1, isoform CRA_d OS=Homo	1.600	1.155	1.519	1.167	1.527	1.189	1.357	1.150
Q9GZX9	Twisted gastrulation protein homolog 1 OS=Homo sapiens GN=T	2.747	2.194	3.049	3.031	2.576	2.064	3.004	3.506
Q9ULM3	YEATS domain-containing protein 2 OS=Homo sapiens GN=YEAT	1.473	1.586	1.260	0.765	1.151	1.546	1.428	1.216
B3KQL5	cDNA FLJ90678 fis, clone PLACE1005736, highly similar to Plecks	0.990	0.965	1.129		0.994	1.490	1.573	1.134
H3BMF7	Stathmin domain-containing protein 1 (Fragment) OS=Homo sapi	1.821	1.668	1.991	1.628	1.407	1.452	1.669	1.434

A0A024RD22	Nuclear transcription factor Y, alpha, isoform CRA_a OS=Homo sa	1.893	1.788	1.762	1.455	1.130	1.293	1.386	1.625
R4GNB2	DENN domain-containing protein 4C OS=Homo sapiens GN=DEN	1.304	0.912	1.153	0.778	1.101	0.978	0.865	0.884
Q5VYS8	Terminal uridylyltransferase 7 OS=Homo sapiens GN=ZCCHC6 PE	1.355	0.916	1.086	0.769	1.200	1.084	1.105	0.962
A8K8K1	cDNA FLJ76936, highly similar to Homo sapiens RNA terminal pho	1.287	0.879	1.307	0.867	1.172	1.033	1.071	1.031
P05549	Transcription factor AP-2-alpha OS=Homo sapiens GN=TFAP2A P	1.228	1.194	0.974	0.838	1.024	1.186	1.189	0.854
P06132	Uroporphyrinogen decarboxylase OS=Homo sapiens GN=UROD P	1.379	1.039	1.819	1.380	1.276	1.073	1.205	1.311
Q8N0V3	Putative ribosome-binding factor A, mitochondrial OS=Homo sapi	1.145	1.195	1.085	1.009	0.903	1.120	1.126	1.171
B2RBN3	cDNA, FLJ95601, highly similar to Homo sapiens WD repeat doma	1.574	1.447	1.282	1.040	1.523	1.287	1.366	1.245
A0A087WYB2	RUN and SH3 domain-containing protein 1 (Fragment) OS=Homo	1.083	1.239	1.076	0.578	0.956	0.950	1.090	0.932
O95714	E3 ubiquitin-protein ligase HERC2 OS=Homo sapiens GN=HERC2	1.120	1.188	1.281	1.096	1.104	1.123	1.392	1.122
Q6N075	Molybdate-anion transporter OS=Homo sapiens GN=MFSD5 PE=1	1.859	2.279	1.265	2.453	1.105		1.291	1.428
P52735	Guanine nucleotide exchange factor VAV2 OS=Homo sapiens GN=	2.026	1.163	2.061	1.812	1.863	1.115	2.100	1.742
Q99871	HAUS augmin-like complex subunit 7 OS=Homo sapiens GN=HAU	2.261	1.841	2.241	1.335	1.750	1.264	1.690	1.828
Q9HAU0	Pleckstrin homology domain-containing family A member 5 OS=H	1.992	1.771	1.727	1.437	1.382	1.169	1.739	1.682
P35573	Glycogen debranching enzyme OS=Homo sapiens GN=AGL PE=1	1.272	0.933	1.265	1.099	1.165	0.985	1.341	1.110
O94763	Unconventional prefoldin RPB5 interactor 1 OS=Homo sapiens GN	1.910	1.318	2.091	1.298	1.743	1.764	1.629	1.588
Q96HA1	Nuclear envelope pore membrane protein POM 121 OS=Homo sa						1.440		1.474
Q9Y2U5	Mitogen-activated protein kinase kinase kinase 2 OS=Homo sapie	1.162	1.262	1.237	1.149	0.827	1.055	1.416	0.671
Q86Y37	CDK2-associated and cullin domain-containing protein 1 OS=Hom	1.776	1.387	1.513	1.054	1.715	1.343	1.498	1.269
P29350	Tyrosine-protein phosphatase non-receptor type 6 OS=Homo sap	0.985	0.735	0.965	0.844	0.871	0.782	1.009	0.797
Q86US8	Telomerase-binding protein EST1A OS=Homo sapiens GN=SMG6	1.527	1.120	1.428	0.749	1.191	1.155	1.581	1.014
Q9H9G7	Protein argonaute-3 OS=Homo sapiens GN=AGO3 PE=1 SV=2 - [	0.654	1.149	1.058		0.770	1.184	1.176	1.033
Q08722	Leukocyte surface antigen CD47 OS=Homo sapiens GN=CD47 PE	2.830	3.176	2.355	2.596	2.771	1.321	3.195	3.153
Q13884	Beta-1-syntrophin OS=Homo sapiens GN=SNTB1 PE=1 SV=3 - [S	1.037	1.280				1.084	1.539	
O94913	Pre-mRNA cleavage complex 2 protein Pcf11 OS=Homo sapiens G	1.672	1.719	1.261	1.046	1.470	1.535	1.533	1.169
Q9BYG5	Partitioning defective 6 homolog beta OS=Homo sapiens GN=PAR	1.448	1.289	1.169	0.894	1.300	1.112	1.326	1.207
Q9UPU5	Ubiquitin carboxyl-terminal hydrolase 24 OS=Homo sapiens GN=U	2.015	1.208	1.808	1.227	1.573	1.279	1.755	1.469
Q9BYH2	Cystine/glutamate exchanger OS=Homo sapiens GN=hxCT PE=2	1.535	1.742	0.972	0.969	1.338	1.435	0.986	0.868
Q92629	Delta-sarcoglycan OS=Homo sapiens GN=SGCD PE=1 SV=2 - [SG	1.434	1.804	1.120	1.195	1.092	1.612	1.208	1.125
Q68CZ6	HAUS augmin-like complex subunit 3 OS=Homo sapiens GN=HAU	0.682	0.653	0.648	0.535	0.905	1.406	0.851	0.817
C9JFB2	Protein YIPF1 (Fragment) OS=Homo sapiens GN=YIPF1 PE=4 SV	2.284	2.237	2.280	2.273	1.908	1.510	2.657	2.489
Q9H0J9	Poly [ADP-ribose] polymerase 12 OS=Homo sapiens GN=PARP12	1.593	2.433	2.541	2.111	1.521	2.130	2.539	2.461
Q9C0K1	Zinc transporter ZIP8 OS=Homo sapiens GN=SLC39A8 PE=2 SV=	2.372	2.546	2.461	2.520	2.378	1.679	2.667	2.898
A0A087WYW3	NK-tumor recognition protein OS=Homo sapiens GN=NKTR PE=4	1.763	2.001	1.603	1.489	1.786	1.816	2.011	1.957
B4E2S7	cDNA FLJ58780, highly similar to Homo sapiens lysosomal-associa	2.139	2.121	1.830	1.884	2.229	1.372	1.972	1.870
Q9BS48	HSF2 protein OS=Homo sapiens GN=HSF2 PE=2 SV=1 - [Q9BS48	1.293	2.809	2.136	1.334	1.316	2.092	2.726	1.861
A0A087WW32	Interleukin-1 receptor-associated kinase 4 OS=Homo sapiens GN=	1.381	0.973	1.449	1.225	1.270	0.978	1.275	1.221
Q86YV9	Hermansky-Pudlak syndrome 6 protein OS=Homo sapiens GN=HI	1.612	1.066	1.180	0.916	1.150	1.192	1.202	0.964
A0A024RDD6	Uncharacterized protein OS=Homo sapiens GN=LOC285513 PE=4	1.404	1.165	0.934	0.675	1.261	0.932	0.724	0.486
Q92558	Wiskott-Aldrich syndrome protein family member 1 OS=Homo sap	2.077	1.377	2.140	1.387	1.964	1.106	1.702	1.856

Q8IXZ2	Zinc finger CCCH domain-containing protein 3 OS=Homo sapiens	1.220	2.062	1.280	1.022	1.364	1.770	1.654	1.246
Q86UA1	Pre-mRNA-processing factor 39 OS=Homo sapiens GN=PRPF39 P	0.729	0.702	0.950	1.091	1.011	1.065	1.003	0.569
H0Y544	Solute carrier family 23 member 2 (Fragment) OS=Homo sapiens	2.926	3.421	2.455	2.393	2.621		2.627	2.461
B7Z5S3	cDNA FLJ61654, highly similar to Homo sapiens cyclin M1 (CNNM	1.433	1.742	1.336	1.267	1.241	1.322	1.419	1.081
B2R7T2	cDNA, FLJ93591, highly similar to Homo sapiens transforming gro	1.095	0.354	0.314	0.136	1.283	0.642	0.457	0.147
P08581	Hepatocyte growth factor receptor OS=Homo sapiens GN=MET P	1.276	1.447	0.995	0.930	1.144	1.296	1.293	0.938
Q9Y289	Sodium-dependent multivitamin transporter OS=Homo sapiens G	1.922	2.844	2.522	2.611	1.579	1.750	1.955	2.444
B3KW34	cDNA FLJ42071 fis, clone SYNOV2014157, highly similar to Protei	1.341	1.442	1.081	1.161	1.134	1.305	1.425	1.077
B3KVF3	cDNA FLJ16485 fis, clone BRTHA3004307, highly similar to Dual s	1.744	2.236	1.219	1.075	1.610	1.174	1.696	1.298
F5H1F6	Vacuolar protein sorting-associated protein 37B (Fragment) OS=H	3.506	2.711	2.719	2.014	2.327	1.604	2.465	2.262
Q5JXJ0	Mitochondrial ribosome-associated GTPase 2 (Fragment) OS=Hor	1.298	1.940	1.459	1.102	1.132	1.463	1.604	1.064
G3V148	RING finger protein 121 OS=Homo sapiens GN=RNF121 PE=4 SV	9.471	8.571	7.314	6.740	2.332	1.348	2.340	2.611
F8W9Y0	Syntaxin-3 OS=Homo sapiens GN=STX3 PE=1 SV=1 - [F8W9Y0_	1.426	1.388	1.236	1.208	1.297	1.249	1.233	1.140
A6ND36	Protein FAM83G OS=Homo sapiens GN=FAM83G PE=1 SV=2 - [F	1.350	1.322	1.197	1.316	1.102	1.113	1.465	1.186
B4DZ84	MAGUK p55 subfamily member 2 OS=Homo sapiens GN=MPP2 P	0.977	1.110	0.830	1.035	1.642	1.469	0.835	1.199
Q7Z333	Probable helicase senataxin OS=Homo sapiens GN=SETX PE=1 S	1.257	1.174	0.871	0.729	1.130	1.180	0.926	0.882
Q8N3Z3	GTP-binding protein 8 OS=Homo sapiens GN=GTPBP8 PE=2 SV=	1.518	1.962	1.692		1.469	0.862	1.234	
D6RIS5	Methylmalonic aciduria type A protein, mitochondrial OS=Homo s	1.078	1.330		1.139		0.614		
H9ZYI9	GA binding protein transcription factor beta subunit 1 transcript v	1.436	1.519	1.399	1.102	1.422	1.273	1.674	1.570
Q96HA7	Tonsoku-like protein OS=Homo sapiens GN=TONSL PE=1 SV=2 -	1.365	1.021	1.116	0.800	1.283	1.065	1.514	1.362
B4DKU9	Coronin OS=Homo sapiens PE=2 SV=1 - [B4DKU9_HUMAN]	1.142	0.896	1.287	0.950	1.099	0.932	1.173	1.103
B2RBL9	cDNA, FLJ95582, highly similar to Homo sapiens breast cancer ar	2.149	1.217	1.407	0.942	1.857	1.214	1.424	1.010
Q14161	ARF GTPase-activating protein GIT2 OS=Homo sapiens GN=GIT2	1.371	1.072	1.121	0.771	1.208	1.270	1.334	0.955
B4DPL8	cDNA FLJ51721, highly similar to Glucose-6-phosphate translocas	3.064	3.220	2.887	2.933	2.254	1.499	2.801	2.942
B3KNH9	cDNA FLJ14643 fis, clone NT2RP2001597, weakly similar to RYAN	0.442	0.848	0.655	0.477	0.750	1.476	0.813	0.642
F5H837	Retinoblastoma-like protein 2 (Fragment) OS=Homo sapiens GN=			2.210					
P49336	Cyclin-dependent kinase 8 OS=Homo sapiens GN=CDK8 PE=1 SV	2.063	2.363	1.517	0.949	1.996	1.795	1.730	1.330
Q96AA3	Protein RFT1 homolog OS=Homo sapiens GN=RFT1 PE=1 SV=1	1.541	1.242	1.348	1.015	1.098	1.232	1.652	1.401
Q6P4I2	WD repeat-containing protein 73 OS=Homo sapiens GN=WDR73	0.997	0.957	1.128	1.102	1.104	1.397	1.305	1.246
C9JPL0	Cyclin-L1 OS=Homo sapiens GN=CCNL1 PE=1 SV=1 - [C9JPL0_H	1.189	1.204	0.961	0.639	1.226	1.333	1.019	0.688
Q9BZV1	UBX domain-containing protein 6 OS=Homo sapiens GN=UBXN6	1.197	0.965	1.344	1.002	1.429	1.094	1.080	1.042
B5MC02	Metalloreductase STEAP2 OS=Homo sapiens GN=STEAP2 PE=4 S	2.728	2.673	1.991	2.247	1.728	0.921	1.922	1.828
Q9Y6Y0	Influenza virus NS1A-binding protein OS=Homo sapiens GN=IVNS					0.501	0.802	0.636	0.542
A0PJW6	Transmembrane protein 223 OS=Homo sapiens GN=TMEM223 PE	2.404	2.161	2.146	2.321	1.601	1.075	2.044	2.060
B7ZLE0	IBTK protein OS=Homo sapiens GN=IBTK PE=2 SV=1 - [B7ZLE0_	1.559	0.960	0.854	0.610	1.265	0.885	1.140	1.155
Q8IYB7	DIS3-like exonuclease 2 OS=Homo sapiens GN=DIS3L2 PE=1 SV	1.207	0.790	1.556	1.250	1.117	1.111	1.204	1.310
Q5VTE6	Protein angel homolog 2 OS=Homo sapiens GN=ANGEL2 PE=1 S	1.671	1.549	1.198	1.051	1.367	1.564	1.353	1.205
Q15334	Lethal(2) giant larvae protein homolog 1 OS=Homo sapiens GN=	0.949	0.813	1.119	0.848	0.964	0.838	0.989	1.093
Q96RR1	Twinkle protein, mitochondrial OS=Homo sapiens GN=PEO1 PE=	1.637	1.986	1.153	0.867	1.320	1.209	1.662	1.252
Q6P4Q7	Metal transporter CNNM4 OS=Homo sapiens GN=CNNM4 PE=1 S	1.227	1.699	1.027	1.236	0.895	1.025	1.325	1.243

B4DGP5	cDNA FLJ57365, highly similar to Serine/threonine-protein kinase	1.641	0.942	1.367	0.819	1.613	1.458	1.662	1.179
O75146	Huntingtin-interacting protein 1-related protein OS=Homo sapien	1.928	1.826	1.778	1.451	1.834	1.318	2.165	1.877
Q9UPN6	Protein SCAF8 OS=Homo sapiens GN=SCAF8 PE=1 SV=1 - [SCAF	1.397	1.545	1.153	0.869	1.472	1.229	1.195	0.930
Q8NBF6	Late secretory pathway protein AVL9 homolog OS=Homo sapiens	1.283	0.951	0.907	0.644	1.215	1.072	1.103	0.857
Q9C0H2	Protein tweety homolog 3 OS=Homo sapiens GN=TTYH3 PE=1 S\	1.115	0.979	0.763	0.609	1.161	0.959	0.921	0.714
O43909	Exostosin-like 3 OS=Homo sapiens GN=EXTL3 PE=1 SV=1 - [EXT	1.034	1.334	1.048	1.256	1.138	1.296	1.363	1.062
Q14191	Werner syndrome ATP-dependent helicase OS=Homo sapiens GN	1.243	1.667	1.256	0.780	1.120	1.264	1.343	1.198
B2R928	Mitogen-activated protein kinase kinase kinase kinase OS=Homo	1.578	1.159	1.819	1.483	1.501	1.242	1.843	1.228
Q04656	Copper-transporting ATPase 1 OS=Homo sapiens GN=ATP7A PE=	1.234	1.312	0.942	1.013	0.979	1.116	1.045	1.016
B3KQM3	cDNA FLJ90732 fis, clone PLACE1010081, highly similar to Recept	1.213	0.795	1.067	0.759	0.719	1.052	1.229	0.863
Q96EA4	Protein Spindly OS=Homo sapiens GN=SPDL1 PE=1 SV=2 - [SPD	1.474	1.390	0.869	0.601	1.316	1.429	1.378	1.005
F8W9L8	PHD finger protein 20-like protein 1 OS=Homo sapiens GN=PHF2	1.291	1.472	1.086	0.788	1.348	1.055	1.238	1.288
Q14643	Inositol 1,4,5-trisphosphate receptor type 1 OS=Homo sapiens GI	1.305	1.356	1.303	1.383	0.958	1.118	1.444	1.321
A8K940	cDNA FLJ77630, highly similar to Homo sapiens BPY2 interacting	0.994	0.780	0.949	0.554	0.779	0.903	0.830	0.699
Q6ZRS2	Helicase SRCAP OS=Homo sapiens GN=SRCAP PE=1 SV=3 - [SR(	0.982	1.105	0.988	0.712	0.930	1.190	0.993	0.749
Q7RTP6	Protein-methionine sulfoxide oxidase MICAL3 OS=Homo sapiens (	1.180	1.153	1.136	0.871	1.247	0.959	1.168	1.038
Q6PHZ7	NR2C2 protein OS=Homo sapiens GN=NR2C2 PE=2 SV=1 - [Q6P	0.927	1.150	0.881	0.630	0.731	1.072	1.120	1.099
Q8NC44	Protein FAM134A OS=Homo sapiens GN=FAM134A PE=1 SV=3 -	3.527	2.898	2.362	2.739	2.699	2.027	2.568	2.965
B7Z3Q1	cDNA FLJ55638 OS=Homo sapiens PE=2 SV=1 - [B7Z3Q1_HUMA	1.365	1.426	1.121	1.107	1.369	1.188	1.276	1.120
Q8TDY2	RB1-inducible coiled-coil protein 1 OS=Homo sapiens GN=RB1CC	1.145	1.093	0.950	0.792	1.309	1.334	1.422	1.242
A0A024R468	Chondroitin polymerizing factor, isoform CRA_d OS=Homo sapien	1.496	1.628	1.384	1.249	1.279	1.187	1.722	1.680
M0R2L2	TBC1 domain family member 17 (Fragment) OS=Homo sapiens G	1.282	1.045	1.764	1.073	1.373	1.267	1.218	0.985
Q9NR77	Peroxisomal membrane protein 2 OS=Homo sapiens GN=PXMP2		3.483	2.477	2.857		1.986	3.268	2.565
C9J7Z4	tRNA-splicing endonuclease subunit Sen2 (Fragment) OS=Homo s	1.200	1.301	1.098	0.802	1.345	1.103	1.058	0.908
B7Z7T1	cDNA FLJ58708, weakly similar to Mus musculus GTPase, very lar	2.923	1.935	2.834	2.186	2.518	1.349	2.620	1.960
B4DR18	cDNA FLJ60816, highly similar to Probable phospholipid-transport	1.107	1.036	1.260	1.004	1.202	0.966	1.273	1.070
Q8NHU6	Tudor domain-containing protein 7 OS=Homo sapiens GN=TDRD	1.183	0.892	1.018	0.802	0.982	1.011	1.088	0.864
Q8N4V6	TMEM181 protein (Fragment) OS=Homo sapiens GN=TMEM181 F	2.040	2.329	1.710	1.530	1.498	1.323	1.584	1.621
O95235	Kinesin-like protein KIF20A OS=Homo sapiens GN=KIF20A PE=1	1.987	2.012	0.771	0.603	1.236	1.262	0.931	0.362
Q8IVL5	Prolyl 3-hydroxylase 2 OS=Homo sapiens GN=LEPREL1 PE=1 SV=	1.282	1.040		0.691	1.540	1.366	1.012	1.124
Q86TM6	E3 ubiquitin-protein ligase synoviolin OS=Homo sapiens GN=SYV	5.205	4.233	3.543	2.852	1.661	1.373	2.120	1.573
A7LFP5	Beta-mannosidase OS=Homo sapiens GN=MANBA PE=2 SV=1 - [	1.257	1.002	1.266	1.074	1.346	1.140	1.363	1.112
B3KRD8	SEC14-like 2 (S. cerevisiae), isoform CRA_c OS=Homo sapiens GI	1.149	1.009	0.987	0.844	0.968	0.886	0.906	0.993
B3KPG6	cDNA FLJ31769 fis, clone NT2RI2007956, highly similar to Chond	2.232	2.415	1.798	1.698	1.862	1.615	1.966	1.773
Q0MQR4	Poly (ADP-ribose) glycohydrolase OS=Homo sapiens GN=PARG P	1.694	1.662	1.500	1.495	1.743	1.420	1.698	1.619
Q9UKB3	DnaJ homolog subfamily C member 12 OS=Homo sapiens GN=DI	1.019	0.515	0.826	0.736	0.906	0.682	0.599	0.589
K7EKI8	Periplakin OS=Homo sapiens GN=PPL PE=1 SV=1 - [K7EKI8_HUI	1.016	1.076	0.929	0.889	1.085	0.933	0.847	0.955
A0A087X2C2	Protein FAM69A OS=Homo sapiens GN=FAM69A PE=4 SV=1 - [A	2.502	2.591	2.049	2.311	1.693	1.354	2.671	2.323
B4DHN9	cDNA FLJ53884, highly similar to mRNA decapping enzyme 1A (E	1.200	1.364	1.662	1.493	1.241	1.945	2.091	1.194
O15427	Monocarboxylate transporter 4 OS=Homo sapiens GN=SLC16A3 I	1.359	1.632	1.295	1.540	1.160	1.389	1.458	1.329

Q86X02	Cerebellar degeneration-related protein 2-like OS=Homo sapiens	1.351	0.949	0.710	0.659	0.866	0.895	0.965	0.558
O43542	DNA repair protein XRCC3 OS=Homo sapiens GN=XRCC3 PE=1 S	1.934	1.123	1.344	1.261	1.210	1.609	1.549	1.495
A0A087X0C8	Ankyrin repeat and SOCS box protein 2 OS=Homo sapiens GN=A	0.965	1.185	1.091	1.052	0.968	0.979	1.229	1.092
B3KUH0	cDNA FLJ39883 fis, clone SPLEN2016268, highly similar to Protei	1.954	1.184	1.672	0.972	1.459	1.031	1.333	1.019
A8KAK5	cDNA FLJ77399, highly similar to Homo sapiens cofactor required	1.546	1.860	1.158	0.785	1.343	1.290	1.186	1.026
I3L2K5	Zinc finger CCCH domain-containing protein 7A (Fragment) OS=H	1.261	1.492	0.969	0.768	1.072	1.190	0.961	1.022
B4DQI5	cDNA FLJ52686, highly similar to Homo sapiens LysM, peptidogly	1.375	0.943	0.979	0.993	0.772	1.264	1.013	1.034
O95801	Tetratricopeptide repeat protein 4 OS=Homo sapiens GN=TTC4 P	1.633	1.127	1.340	1.174	1.220	1.306	1.364	1.584
H7C270	Carboxy-terminal domain RNA polymerase II polypeptide A small	1.665	2.438	1.699	1.819	1.480	1.492	1.780	1.612
Q9NUP7	tRNA:m(4)X modification enzyme TRM13 homolog OS=Homo sap	0.952	0.933	1.166	0.739	1.003	1.015	1.082	0.878
A0A087X1H5	Arf-GAP with coiled-coil, ANK repeat and PH domain-containing p	1.213	0.930	1.136	0.876	1.201	0.958	1.087	0.941
Q8TDM6	Disks large homolog 5 OS=Homo sapiens GN=DLG5 PE=1 SV=4	0.983	1.184	1.155	0.989	1.028	1.200	1.360	1.182
B4DIA5	cDNA FLJ59531, highly similar to Transcription factor E3 OS=Hom	1.020	0.859	0.871	0.650	0.979	1.024	1.065	1.047
Q9Y2B1	Transmembrane protein 5 OS=Homo sapiens GN=TMEM5 PE=1 S	1.228	1.839	1.283	1.437	1.063	1.256	1.515	1.383
O43156	TELO2-interacting protein 1 homolog OS=Homo sapiens GN=TTI	1.221	1.517	1.128	1.142	1.809	1.096	1.516	1.210
B4DEL2	Inhibitor of growth protein OS=Homo sapiens PE=2 SV=1 - [B4D	2.262	2.815	2.027	1.462	1.726	2.804	1.879	2.353
B7ZL14	FNBP1 protein OS=Homo sapiens GN=FNBP1 PE=1 SV=1 - [B7Z	1.635	1.277	2.377	2.595	1.246	1.076	1.638	2.087
Q8N122	Regulatory-associated protein of mTOR OS=Homo sapiens GN=R	1.224	1.218	1.102	1.103	1.261	1.212	1.009	1.094
J3QK86	Bromodomain adjacent to zinc finger domain, 2A, isoform CRA_a	1.418	1.626	1.133	1.019	1.282	1.154	1.207	1.183
A0A024R301	Choline dehydrogenase, isoform CRA_a OS=Homo sapiens GN=C	1.563	1.622	1.660	1.586	1.092	1.174	1.599	1.716
B8XCX8	EPC1/ASXL2b fusion protein OS=Homo sapiens PE=2 SV=1 - [B8	0.936	1.330	0.977	0.602	1.069	1.064	1.239	1.179
E9PRF4	Histone-lysine N-methyltransferase SETDB1 (Fragment) OS=Hom	1.473	1.779	1.193	1.003	1.597	1.522	1.500	1.061
Q59EA4	Phospholipase D1 variant (Fragment) OS=Homo sapiens PE=2 S	1.534	1.231	0.976	1.083	1.802	0.924	1.077	1.006
E9PMH5	Baculoviral IAP repeat-containing protein 2 OS=Homo sapiens G	1.284	0.561	0.569	0.587	1.352	0.854	1.250	
E9PRM7	Solute carrier family 22 member 18 OS=Homo sapiens GN=SLC2	1.415	1.538	1.326	1.016	1.031	1.036	1.233	1.136
Q86TW2	Uncharacterized aarF domain-containing protein kinase 1 OS=Ho	1.590	1.746	1.536	1.506	1.667	1.527	1.622	2.356
Q96BD5	PHD finger protein 21A OS=Homo sapiens GN=PHF21A PE=1 SV	0.915	0.894	0.785	0.776	0.755	1.057	0.869	0.787
Q5HYK7	SH3 domain-containing protein 19 OS=Homo sapiens GN=SH3D1	1.511	0.971	1.206	0.972	1.126	1.421	1.259	0.833
B4DJ22	cDNA FLJ52945, highly similar to Zinc finger HIT domain-containi	1.589	2.400	1.433	1.153	1.765	1.252	1.840	1.816
P49356	Protein farnesyltransferase subunit beta OS=Homo sapiens GN=F	1.879	1.287	1.715	1.532	1.452	1.176	1.607	1.561
B4DFV1	Protein kinase C OS=Homo sapiens PE=2 SV=1 - [B4DFV1_HUM	0.935	1.156					1.271	0.869
Q8N1G0	Zinc finger protein 687 OS=Homo sapiens GN=ZNF687 PE=1 SV=	1.082	1.086	0.607	0.652	0.964	1.114	1.003	0.791
G3V179	Protein FAM118B OS=Homo sapiens GN=FAM118B PE=1 SV=1 -	2.079	1.915	1.974	1.387	2.232	1.272	1.811	1.579
B3KMZ0	cDNA FLJ12979 fis, clone NT2RP2006334, highly similar to WD re	1.602	1.292	1.557	1.057	1.455	0.966	1.462	1.353
D3DW85	Iron-responsive element binding protein 2, isoform CRA_a OS=H	1.234	1.070	0.968	0.674	1.259	1.040	0.994	0.749
Q53GT1	Kelch-like protein 22 OS=Homo sapiens GN=KLHL22 PE=1 SV=2	3.235	4.063	2.161	2.585	3.211	3.152	3.220	3.375
F8W1P7	Natural resistance-associated macrophage protein 2 OS=Homo sa	1.239	1.379	1.153	1.126	1.176	1.255	1.348	0.831
Q96NL8	Protein C8orf37 OS=Homo sapiens GN=C8orf37 PE=1 SV=1 - [C	0.793	0.602	0.754	0.554	0.759	0.931	0.550	0.571
F2YHL7	Apolipoprotein B mRNA editing enzyme cytidine deaminase OS=H	1.508	1.339	1.295	1.037	1.539	1.179	1.247	1.390
I3L2J0	Protein capicua homolog OS=Homo sapiens GN=CIC PE=1 SV=1	1.121	1.331	0.934	0.829	1.368	1.394	1.243	1.333

Q9UPM8	AP-4 complex subunit epsilon-1 OS=Homo sapiens GN=AP4E1 PE	1.723	1.331	1.555	1.160	1.397	1.248	1.674	1.345
B2R6N3	cDNA, FLJ93032, highly similar to Homo sapiens zinc finger, A20	1.048	0.768	0.695	0.401	0.981	0.802	0.703	0.482
H0YLX2	DNA-binding protein RFX7 OS=Homo sapiens GN=RFX7 PE=1 SV	1.083	1.679	1.138	0.957	0.978	1.506	1.781	1.692
Q9H9A7	RecQ-mediated genome instability protein 1 OS=Homo sapiens G	1.256	1.539	2.262	2.207	1.259	1.454	1.605	1.560
P19474	E3 ubiquitin-protein ligase TRIM21 OS=Homo sapiens GN=TRIM2	1.140	1.039	0.675	0.634	0.971	1.169	1.153	0.877
Q59GK2	N-deacetylase/N-sulfotransferase (Heparan glucosaminyl) 1 variar	1.625	2.236	1.557	1.817	1.556	1.712	1.887	1.690
P14316	Interferon regulatory factor 2 OS=Homo sapiens GN=IRF2 PE=1	0.931	1.445	0.901	0.708	0.912	0.926	1.030	1.104
Q8N1W1	Rho guanine nucleotide exchange factor 28 OS=Homo sapiens GI	2.010	1.017	1.150	0.854	1.308	1.038	1.175	1.036
B9EK47	HEAT repeat containing 5B OS=Homo sapiens GN=HEATR5B PE=	1.238	1.201	1.372	0.987	1.208	1.110	1.139	0.957
A0A087WX08	Gamma-adducin OS=Homo sapiens GN=ADD3 PE=4 SV=1 - [A0/	1.116	0.947	1.041	0.695	0.841	0.915	1.062	0.887
Q2KHM9	Uncharacterized protein KIAA0753 OS=Homo sapiens GN=KIAA0	1.479	1.722	1.293	1.075	1.176	1.060	1.265	1.029
P10244	Myb-related protein B OS=Homo sapiens GN=MYBL2 PE=1 SV=1	1.973	3.718	4.018	2.932	1.517	2.462	4.035	4.235
A0A024R8L8	Envoplakin, isoform CRA_a OS=Homo sapiens GN=EVPL PE=4 SV	1.192	1.288	1.148	1.191	1.281	1.260	1.211	1.261
Q9H490	Phosphatidylinositol glycan anchor biosynthesis class U protein OS	1.609	1.869	1.745	1.598	1.614	1.297	1.869	1.658
Q8TE77	Protein phosphatase Slingshot homolog 3 OS=Homo sapiens GN=	1.139	0.912	1.017	0.668	1.189	1.078	0.993	0.751
E7EMV7	TNFAIP3-interacting protein 1 OS=Homo sapiens GN=TNIP1 PE=	1.204	0.813	0.947	0.731	1.287	1.170	1.186	0.741
Q6ZMK4	FLJ00323 protein (Fragment) OS=Homo sapiens GN=FLJ00323 P	1.635	1.206	1.048	0.741	1.287	1.351	1.322	1.070
O43318	Mitogen-activated protein kinase kinase kinase 7 OS=Homo sapie	0.611	0.481	0.477	0.401	0.723	0.701	0.516	0.292
Q9NQZ2	Something about silencing protein 10 OS=Homo sapiens GN=UTI	1.571	1.489	1.113	0.995	1.349	1.410	1.362	1.157
A0A024RAL3	Zinc finger, FYVE domain containing 16, isoform CRA_a OS=Hom	1.298	1.172	1.397	1.018	1.420	1.213	1.327	1.089
Q8IWV8	E3 ubiquitin-protein ligase UBR2 OS=Homo sapiens GN=UBR2 PE	1.339	1.082	1.164	1.175	1.462	1.185	1.618	1.092
Q6PII3	Coiled-coil domain-containing protein 174 OS=Homo sapiens GN=	1.277	1.301	1.259	0.886	1.199	1.572	1.361	0.965
A0JP11	Phosphoinositide-3-kinase, regulatory subunit 4 OS=Homo sapier	1.426	1.012	1.250	0.930	1.141	1.089	1.138	0.919
O43379	WD repeat-containing protein 62 OS=Homo sapiens GN=WDR62	1.353	1.123	1.407	1.244	1.272	1.096	1.088	1.129
F8W689	DNA-binding protein RFX5 (Fragment) OS=Homo sapiens GN=RF		2.173	1.847		1.304	0.750	1.934	1.777
Q96BY6	Dedicator of cytokinesis protein 10 OS=Homo sapiens GN=DOCK	1.844	1.297	1.461	1.036	1.488	1.100	1.358	1.182
B4E126	cDNA FLJ61199, highly similar to UBX domain-containing protein	1.994	1.276	1.559	1.078	1.566	1.391	1.633	1.133
Q6Y288	Beta-1,3-glucosyltransferase OS=Homo sapiens GN=B3GALTL PE	5.210	3.814	4.555	4.852	4.267	1.979	5.180	6.298
Q14728	Major facilitator superfamily domain-containing protein 10 OS=Ho	1.363	1.491	1.288	1.160	1.134	1.240	1.331	1.035
Q16626	Male-enhanced antigen 1 OS=Homo sapiens GN=MEA1 PE=1 SV=	0.720	0.693	0.693	0.525	0.927	1.128	1.005	0.740
B4E0T7	Leucine-rich repeat and calponin homology domain-containing pr	1.797	1.419	1.431	1.251	1.341	1.326	1.375	1.861
Q96K49	Transmembrane protein 87B OS=Homo sapiens GN=TMEM87B PI	2.384	2.262	1.907	1.674	1.976	1.349	1.780	1.749
D3DUE4	Ankyrin repeat and SAM domain-containing protein 3 OS=Homo s	1.808	1.400	2.036	1.684	1.472	1.171	1.622	1.704
P21397	Amine oxidase [flavin-containing] A OS=Homo sapiens GN=MAO/	1.024	1.596	1.092	1.440	0.999	1.092	1.069	1.215
A0A024R1R9	HCG31740, isoform CRA_b OS=Homo sapiens GN=hCG_31740 PI	1.050	1.448		0.562	1.212	1.285	1.133	0.942
B4DLQ8	cDNA FLJ55718, highly similar to Sterol regulatory element-bindir	1.175	1.221	0.448	0.286	0.848	0.750	0.717	0.498
Q6PJF5	Inactive rhomboid protein 2 OS=Homo sapiens GN=RHBDF2 PE=	1.125	1.742	1.040	1.133	1.051	1.199	1.310	1.186
G3V1Y1	ARP6 actin-related protein 6 homolog (Yeast), isoform CRA_d OS	1.645	1.563	1.485	1.026	1.568	1.630	1.752	1.630
A4UHR0	C2ORF3 variant 3 OS=Homo sapiens GN=C2orf3 PE=2 SV=1 - [A	0.842	0.708	0.913		0.751	1.721	1.063	0.854
Q92560	Ubiquitin carboxyl-terminal hydrolase BAP1 OS=Homo sapiens GN	0.949	0.980	0.737	0.579	0.856	1.080	1.066	0.778

B4E1V0	cDNA FLJ54839, highly similar to Lactotransferrin (EC 3.4.21.-) O	2.100	0.556	1.157	0.773	1.896	1.107	1.374	1.189
Q9Y2V7	Conserved oligomeric Golgi complex subunit 6 OS=Homo sapiens	1.304	1.157	1.261	0.874	1.187	1.204	1.012	0.968
Q9H4L5	Oxysterol-binding protein-related protein 3 OS=Homo sapiens GN	0.942	0.660	1.345	0.868	0.902	1.165	1.055	1.108
Q8IV36	Protein HID1 OS=Homo sapiens GN=HID1 PE=1 SV=1 - [HID1_H	1.384	1.294	1.211	0.704	1.095	0.923	1.177	1.060
Q15147	1-phosphatidylinositol 4,5-bisphosphate phosphodiesterase beta-	1.313	1.163	1.218	0.885	1.025	1.135	1.236	1.082
Q9H467	CUE domain-containing protein 2 OS=Homo sapiens GN=CUEDC	1.367	1.175	1.546	1.057	1.197	1.183	1.370	1.436
Q5F1R6	DnaJ homolog subfamily C member 21 OS=Homo sapiens GN=D			1.438	2.176	1.990			
Q9BZ95	Histone-lysine N-methyltransferase NSD3 OS=Homo sapiens GN=	1.144	1.660	1.204	1.264	0.902	1.179	1.483	1.302
Q9NTM9	Copper homeostasis protein cutC homolog OS=Homo sapiens GN	0.891	0.804	0.715	0.457	0.845	0.957	0.703	0.617
O60293	Zinc finger C3H1 domain-containing protein OS=Homo sapiens G	0.957	1.054	0.879	0.733	0.923	1.005	1.057	0.732
Q9UII4	E3 ISG15--protein ligase HERC5 OS=Homo sapiens GN=HERC5 P	1.177	1.359	2.208	1.134	1.521	1.212	1.162	1.243
A0A024RBL8	F-box protein 21, isoform CRA_c OS=Homo sapiens GN=FBXO21	2.219	1.859	2.366	1.586	1.606	1.398	1.786	1.475
A8K2B9	cDNA FLJ75615, highly similar to Homo sapiens DTW domain cor					0.982		1.031	
Q8TEY7	Ubiquitin carboxyl-terminal hydrolase 33 OS=Homo sapiens GN=	1.465	1.393	1.186	0.730	1.279	1.424	1.425	0.944
Q9UGJ1	Gamma-tubulin complex component 4 OS=Homo sapiens GN=TU	1.463	0.970	1.488	1.178	1.284	0.935	1.085	1.051
P50747	Biotin--protein ligase OS=Homo sapiens GN=HLCS PE=1 SV=1 -	1.279	0.983	1.123	1.939	0.593	1.619	1.131	1.127
A0A024R1T4	Trinucleotide repeat containing 6B, isoform CRA_b OS=Homo sap	0.611	0.948	0.660	0.457	0.935	1.091	0.765	0.481
Q9H1E5	Thioredoxin-related transmembrane protein 4 OS=Homo sapiens	1.280	1.368	1.093	0.906	0.980	1.279	1.329	0.847
E9PC87	Transmembrane and coiled-coil domains protein 1 OS=Homo sap	1.080	1.163	0.910	0.859	0.828	1.063	1.066	0.829
P41229	Lysine-specific demethylase 5C OS=Homo sapiens GN=KDM5C P	1.158	1.026	0.813	0.463	1.109	1.293	1.002	0.667
Q6ZNF7	cDNA FLJ16146 fis, clone BRAMY2040159, highly similar to Homo	1.362	1.135	1.304	1.029	1.117	0.998	1.220	1.103
E2GH18	T-cell factor-4 variant C OS=Homo sapiens GN=TCF7L2 PE=2 SV	1.452	1.865	1.396	1.309	1.447	1.426	1.551	1.667
Q2TB39	TCF3 protein OS=Homo sapiens GN=TCF3 PE=1 SV=1 - [Q2TB3	1.965	1.947	1.671	1.378	1.980	1.441	2.159	2.187
F5H6S1	Lysine-specific demethylase 6A OS=Homo sapiens GN=KDM6A P	1.323	1.456	0.956	0.803	1.185	1.395	1.454	1.237
B4E3N4	cDNA FLJ58238, highly similar to Chloride channel protein 7 OS=	1.398	1.220	1.128	0.963	1.284	1.320	1.346	1.146
Q6PIJ6	F-box only protein 38 OS=Homo sapiens GN=FBXO38 PE=1 SV=	0.870	1.023	0.750	0.539	1.136	1.259	1.049	0.586
Q13535	Serine/threonine-protein kinase ATR OS=Homo sapiens GN=ATR	1.093	1.269	0.917	0.787	1.256	1.125	1.120	0.848
Q96RG4	Insulin receptor substrate 2 insertion mutant (Fragment) OS=Ho	1.148	0.674	1.017	0.924	0.867	0.846	0.720	0.483
Q9ULH7	MKL/myocardin-like protein 2 OS=Homo sapiens GN=MKL2 PE=1	0.833	0.946	1.012	0.933	1.107	1.074	1.222	0.865
Q15058	Kinesin-like protein KIF14 OS=Homo sapiens GN=KIF14 PE=1 SV	1.488	1.725	1.216	1.168	1.151	1.215	1.157	1.039
Q8WV41	Sorting nexin-33 OS=Homo sapiens GN=SNX33 PE=1 SV=1 - [SN	1.214	1.039	1.291	0.990	1.126	0.970	1.246	1.150
E7ET89	Probable E3 ubiquitin-protein ligase DTX2 OS=Homo sapiens GN=	0.970	1.280	1.006	0.295	1.161	1.107	0.754	0.513
Q8N5G2	Macoilin OS=Homo sapiens GN=TMEM57 PE=1 SV=1 - [MACOI_	0.674	1.005	0.592	0.693	0.962	1.090	0.801	
Q9NZ52	ADP-ribosylation factor-binding protein GGA3 OS=Homo sapiens	1.926	1.295	1.467	1.426	1.572	1.075	1.774	1.342
Q8TF42	Ubiquitin-associated and SH3 domain-containing protein B OS=H	1.216	0.753	0.754	0.712	1.298	0.937	0.777	0.572
B7Z7F2	cDNA FLJ53518, highly similar to Protein O-linked-mannosebeta-	1.125	1.096	0.931	0.992	0.823	1.131	1.072	1.227
B2RAM2	cDNA, FLJ94999, highly similar to Homo sapiens calpain 7 (CAPN	1.079	0.831	1.005	0.863	0.908	1.206	1.079	0.950
Q9H900	Protein zwilch homolog OS=Homo sapiens GN=ZWILCH PE=1 SV	1.357	0.765	0.877	0.644	1.102	0.945	0.991	0.950
Q8N697	Solute carrier family 15 member 4 OS=Homo sapiens GN=SLC15	0.806	0.892	0.527	0.662	1.174	1.325	1.121	0.808
O14939	Phospholipase D2 OS=Homo sapiens GN=PLD2 PE=1 SV=2 - [PL	0.926	1.368	1.012	1.076	1.160	1.028	1.140	1.047

A8K5H6	cDNA FLJ76659, highly similar to Homo sapiens exonuclease 1 (E	1.311	1.596	0.984	0.991	1.320	1.354	1.267	0.901
A8KAQ3	cDNA FLJ76489, highly similar to Homo sapiens sortilin 1 (SORT1	0.821	1.159	0.748	0.712	0.782	1.017	1.345	1.532
P19793	Retinoic acid receptor RXR-alpha OS=Homo sapiens GN=RXRA PE	1.018	1.419	0.944	0.461	0.902	1.105	0.974	0.887
Q96BZ9	TBC1 domain family member 20 OS=Homo sapiens GN=TBC1D20	3.246	3.835	3.327	2.202	2.406	1.975	3.673	3.329
P02671	Fibrinogen alpha chain OS=Homo sapiens GN=FGA PE=1 SV=2 -	0.946				1.238	0.629		
Q9HCG7	Non-lysosomal glucosylceramidase OS=Homo sapiens GN=GBA2	0.880	0.980	0.895	0.638	1.263	1.377	1.136	0.639
Q13472	DNA topoisomerase 3-alpha OS=Homo sapiens GN=TOP3A PE=1	1.006	1.474	1.227	0.963	0.884	1.467	1.345	1.419
Q5VWG9	Transcription initiation factor TFIID subunit 3 OS=Homo sapiens G	1.399	1.824	1.548	1.318	1.319	1.290	1.575	1.802
A0A087WVT1	Zinc finger protein 48 OS=Homo sapiens GN=ZNF48 PE=4 SV=1	1.541	2.181	1.786	1.791	1.366	2.161	1.983	1.945
Q9BZQ6	ER degradation-enhancing alpha-mannosidase-like protein 3 OS=	1.386	1.259	1.097	1.196	1.381	1.429	1.407	1.199
G3V450	Ectonucleoside triphosphate diphosphohydrolase 5 (Fragment) OS	1.607	1.242	1.329	1.661	1.480	1.226	1.432	1.352
H3BP35	Diphosphomevalonate decarboxylase (Fragment) OS=Homo sapie	1.319	0.877	1.138	1.065	0.938	0.931	1.108	0.886
B5BUA4	Shugoshin-like 1 (S. pombe), isoform CRA_a OS=Homo sapiens G	1.464	1.607	0.978	0.866	1.410	1.416	1.139	1.317
F8VY01	FYVE, RhoGEF and PH domain-containing protein 6 OS=Homo sa	0.936	0.778	0.482	0.686	0.922	1.109	1.009	0.371
Q6UXT9	Abhydrolase domain-containing protein 15 OS=Homo sapiens GN	2.501	3.129	2.736	2.154	1.027	1.276	3.236	2.874
Q8IYT2	Cap-specific mRNA (nucleoside-2'-O-)-methyltransferase 2 OS=Ho	1.164	1.207	0.882	0.847	1.131	1.148	1.100	0.903
Q6W2J9	BCL-6 corepressor OS=Homo sapiens GN=BCOR PE=1 SV=1 - [B	1.448	2.353	1.232	1.062	1.083	1.952	1.876	1.608
Q02388	Collagen alpha-1(VII) chain OS=Homo sapiens GN=COL7A1 PE=	1.222	1.275	1.041	0.778	1.368	1.253	1.189	1.390
P40306	Proteasome subunit beta type-10 OS=Homo sapiens GN=PSMB10	1.036		0.782		1.273	0.906		0.760
A0A024R1W0	Breast cancer 1, early onset, isoform CRA_j OS=Homo sapiens GI	1.692	2.052	1.444	1.079	1.720	1.713	1.985	1.835
M0R0A9	IgG receptor FcRn large subunit p51 OS=Homo sapiens GN=FCG	1.352	1.413	1.241	1.177	1.275	1.070	1.561	1.713
G5E9C0	SP110 nuclear body protein, isoform CRA_b OS=Homo sapiens G	0.567	0.729	0.624	0.614	0.601	1.163	0.433	0.270
Q9ULL5	Proline-rich protein 12 OS=Homo sapiens GN=PRR12 PE=1 SV=2	0.943	1.393	0.902	0.747	1.087	1.209	1.324	1.039
P07996	Thrombospondin-1 OS=Homo sapiens GN=THBS1 PE=1 SV=2 - [	1.331	1.240	0.858	2.233	1.660	0.852	0.967	2.547
Q9Y6X8	Zinc fingers and homeoboxes protein 2 OS=Homo sapiens GN=ZI	0.853	1.183	0.974	0.718	0.964	1.150	0.971	0.900
Q59GT6	Rab geranylgeranyltransferase, beta subunit variant (Fragment) O	4.067	3.206	4.091	3.064	3.036	1.401	3.655	3.295
Q96N83	cDNA FLJ31254 fis, clone KIDNE2005526, highly similar to Homo	1.260	1.433	1.165	1.272	1.066	1.331	1.196	1.450
Q9NWR8	Mitochondrial calcium uniporter regulatory subunit MCUb OS=Hor	2.373	2.307	1.836	1.975	1.617	1.143	2.183	2.096
Q0IIN7	Nuclear receptor coactivator 3 OS=Homo sapiens GN=NCOA3 PE	1.102	0.574	0.522	0.289	1.252	0.781	0.669	0.437
I6W807	Cyclin dependent kinase 19 variant 2 OS=Homo sapiens GN=CDK	1.336				0.751	1.110	1.105	
Q05BS6	UNC93B1 protein (Fragment) OS=Homo sapiens GN=UNC93B1 P	1.463	1.308	1.134	1.475	1.245	1.071	1.204	1.080
B3KXW2	cDNA FLJ46178 fis, clone TESTI4003944, highly similar to Homo	0.919	1.135	0.652	0.578	0.848	0.904	0.769	0.723
Q6NS38	Alpha-ketoglutarate-dependent dioxygenase alkB homolog 2 OS=	1.127	1.487	1.070		1.056	1.335	1.442	
Q6ZMG9	Ceramide synthase 6 OS=Homo sapiens GN=CERS6 PE=1 SV=1	1.338	1.472	1.361	1.300	1.128	1.331	1.398	1.371
Q5VW36	Focadhesin OS=Homo sapiens GN=FOCAD PE=1 SV=1 - [FOCAD	1.763	1.088	1.207	1.120	1.338	1.137	1.296	1.048
A6H8W6	SIPA1L1 protein OS=Homo sapiens GN=SIPA1L1 PE=2 SV=1 - [A	1.319	1.612	1.435	0.997	0.974	1.206	1.506	1.180
B3KQZ4	cDNA FLJ33327 fis, clone BNGH42009025, weakly similar to Hom	1.046	0.628	0.898	0.562	0.841	0.557	0.797	0.752
E9PQC4	Kinetochore protein Nuf2 OS=Homo sapiens GN=NUF2 PE=1 SV=	1.911	1.222	1.532	1.352	1.696	1.175	1.236	1.005
A8K356	cDNA FLJ77750, highly similar to Homo sapiens PHD finger prote	1.427	1.026	1.298	0.633	1.105	1.073	1.223	0.602
P50748	Kinetochore-associated protein 1 OS=Homo sapiens GN=KNTC1 I	1.639	1.193	1.532	1.089	1.235	1.284	1.339	1.253

B7Z2U2	TOM1-like protein 2 OS=Homo sapiens GN=TOM1L2 PE=2 SV=1	1.891	1.337	1.411	1.066	1.542	1.213	2.065	1.300
B7Z6T2	cDNA FLJ50877, highly similar to Type II inositol-3,4-bisphosphat	1.365	0.708	0.796	0.864	1.249	1.031	0.835	0.674
H0YHE2	Intraflagellar transport protein 81 homolog (Fragment) OS=Homo	1.660	0.875	0.774	0.656	1.307	0.856	0.912	0.730
A8K6U0	cDNA FLJ77572, highly similar to Homo sapiens intracellular mem	1.185	1.145	0.808	0.897	1.141	1.047	1.067	1.045
X5D9B8	Ring finger and WD repeat domain 2 isoform H (Fragment) OS=H	2.169	3.608	3.800	3.483	2.150	1.915	3.746	4.966
A0A024R9D8	Sperm associated antigen 1, isoform CRA_b OS=Homo sapiens G	2.161	1.690	1.904	1.411	1.667	1.075	1.593	1.619
Q70CQ2	Ubiquitin carboxyl-terminal hydrolase 34 OS=Homo sapiens GN=U	1.443	1.210	1.292	0.953	1.532	1.097	1.399	1.111
Q9NVN3	Synembryn-B OS=Homo sapiens GN=RIC8B PE=1 SV=2 - [RIC8B	1.768	1.278	1.992	1.091	1.530	1.055	1.708	1.408
B1ALB4	Protein SMG7 (Fragment) OS=Homo sapiens GN=SMG7 PE=1 SV	0.663	0.583	0.658	0.452	0.891	1.129	0.992	0.770
P21359	Neurofibromin OS=Homo sapiens GN=NF1 PE=1 SV=2 - [NF1_HU	1.647	1.314	1.593	1.250	1.457	1.101	1.880	1.365
B3KRE9	POU domain protein OS=Homo sapiens PE=2 SV=1 - [B3KRE9_H	1.018	1.030	0.814	0.460	0.960	0.977	0.951	0.930
H7BXF5	Histone deacetylase complex subunit SAP130 OS=Homo sapiens	1.004	1.158	0.672	0.499	0.935	1.142	0.919	0.627
Q9NP50	Protein FAM60A OS=Homo sapiens GN=FAM60A PE=1 SV=1 - [FA	2.972	3.620	1.186	0.874	2.305	1.874	1.854	0.994
Q9UH51	Low density lipoprotein receptor (Fragment) OS=Homo sapiens G	2.188	1.430	0.694	0.571	1.773	1.251	1.029	0.641
B4DMI0	Protein FAM172A OS=Homo sapiens GN=FAM172A PE=2 SV=1 -	0.581	0.673	0.653	0.408	0.578	1.307	0.608	0.507
B2RDV4	cDNA, FLJ96786 OS=Homo sapiens PE=2 SV=1 - [B2RDV4_HUM	2.772	2.467	2.462	2.452	2.149	1.869	3.020	3.257
Q8N531	F-box/LRR-repeat protein 6 OS=Homo sapiens GN=FBXL6 PE=2 S	3.263	4.954	2.944	2.725	1.144	1.351	1.268	1.033
O94804	Serine/threonine-protein kinase 10 OS=Homo sapiens GN=STK10	1.305	0.984	1.290	0.695	1.133	0.881	1.042	0.832
H0Y5C0	Latrophilin-2 (Fragment) OS=Homo sapiens GN=LPHN2 PE=1 SV	1.199	1.442	1.205	1.195	1.008	1.165	1.356	1.511
A8KA01	Palmitoyltransferase OS=Homo sapiens PE=2 SV=1 - [A8KA01_H	1.561	1.785	1.698	1.399	1.224	1.576	1.838	1.393
B2R6Z9	cDNA, FLJ93200, highly similar to Homo sapiens dolichyl-phospha	1.463	1.381	0.965	1.181	1.178	1.065	1.595	1.450
Q9H3U5	Major facilitator superfamily domain-containing protein 1 OS=Hon		1.290				1.157		
B4DHQ5	cDNA FLJ57110 OS=Homo sapiens PE=2 SV=1 - [B4DHQ5_HUMA	1.009	1.305	0.898	1.215	0.984	0.976	1.482	1.432
Q96DT7	Zinc finger and BTB domain-containing protein 10 OS=Homo sapi	1.101	1.515	0.849	1.105	1.205	1.158	0.871	1.171
Q92556	Engulfment and cell motility protein 1 OS=Homo sapiens GN=ELM	0.980	0.887	0.951	0.816	1.011	0.982	1.094	0.983
Q5CZA0	Putative uncharacterized protein DKFZp686D1070 (Fragment) OS	1.466	1.475	1.195	1.024	1.478	1.465	1.579	1.310
Q9H0V1	Transmembrane protein 168 OS=Homo sapiens GN=TMEM168 PE	0.918	1.447	1.392	1.140	1.263	1.476	1.385	1.305
C9JQV3	Serine/threonine-protein kinase 11-interacting protein OS=Homo	2.074	2.666	2.111	2.154	2.176	1.161	2.275	2.366
A8K4N2	DNA topoisomerase OS=Homo sapiens PE=2 SV=1 - [A8K4N2_HU	0.641	1.252	0.776	0.629	0.737	1.006	0.697	0.738
P46020	Phosphorylase b kinase regulatory subunit alpha, skeletal muscle	1.717	1.113	1.815	1.417	1.603	1.139	1.579	1.368
P25054	Adenomatous polyposis coli protein OS=Homo sapiens GN=APC P	1.208	1.608	1.182	0.995	1.100	1.196	1.260	1.009
Q8NAN7	cDNA FLJ35062 fis, clone OCBBF2019195, highly similar to VERY	1.387	1.541	1.027	0.968	1.233	1.390	1.012	1.133
Q9BX95	Sphingosine-1-phosphate phosphatase 1 OS=Homo sapiens GN=						1.376	1.283	
Q8N9R8	Protein SCAI OS=Homo sapiens GN=SCAI PE=1 SV=2 - [SCAI_H	1.318	1.066	1.281	0.903	1.339	1.145	1.610	1.454
Q9Y2H2	Phosphatidylinositide phosphatase SAC2 OS=Homo sapiens GN=I	0.513	0.493	0.880	0.419	0.711	0.863	0.701	0.442
Q9BYW2	Histone-lysine N-methyltransferase SETD2 OS=Homo sapiens GN	1.159	1.471	0.892	0.668	1.062	1.318	1.346	1.120
Q96QE3	ATPase family AAA domain-containing protein 5 OS=Homo sapien	1.162	1.839	1.335	1.053	1.147	1.355	1.120	0.949
Q9HD67	Unconventional myosin-X OS=Homo sapiens GN=MYO10 PE=1 SV	1.660	0.631	0.776	0.589	1.285	0.855	0.972	0.582
O00418	Eukaryotic elongation factor 2 kinase OS=Homo sapiens GN=EEF	1.113	0.886	1.230	0.712	1.023	0.686	1.028	0.821
P08247	Synaptophysin OS=Homo sapiens GN=SYP PE=1 SV=3 - [SYPH_I	1.419	1.605	1.575	1.164	1.163	1.167	1.136	1.346

Q9H116	GDNF-inducible zinc finger protein 1 OS=Homo sapiens GN=GZF	1.467	1.783	1.286	1.074	1.418	1.703	1.810	1.176
Q9ULI0	ATPase family AAA domain-containing protein 2B OS=Homo sapie	1.726	2.550	2.122	1.611	1.367	1.365	1.494	1.578
Q9H5U6	Zinc finger CCHC domain-containing protein 4 OS=Homo sapiens	1.408	1.683	1.373	1.232	1.494	1.725	1.319	0.972
Q9H176	ZNF143 protein (Fragment) OS=Homo sapiens GN=ZNF143 PE=	1.057	1.653	1.016	0.978	0.929	1.511	1.043	1.309
O75398	Deformed epidermal autoregulatory factor 1 homolog OS=Homo	1.825	2.870	2.838	2.847	1.874	2.258	3.528	2.618
Q9Y2H5	Pleckstrin homology domain-containing family A member 6 OS=H	1.042	1.999	1.100	0.906	0.663	0.944	1.760	1.325
Q6IA51	FLJ13188 protein OS=Homo sapiens GN=FLJ13188 PE=2 SV=1 -	2.229	2.195	2.271	1.424	1.892	1.674	2.298	1.756
B3KTI5	cDNA FLJ38324 fis, clone FCBBF3024663, weakly similar to Zyg-1	1.226	1.008	1.378	0.892	0.994	1.097	1.337	1.019
Q6SJ93	Protein FAM111B OS=Homo sapiens GN=FAM111B PE=1 SV=1 -	1.359	0.497			1.268	2.542	3.344	1.783
Q5BKX5	UPF0692 protein C19orf54 OS=Homo sapiens GN=C19orf54 PE=	1.316	0.844	1.008	0.946	1.059	0.989	1.027	1.000
Q59FW6	Elongation factor, RNA polymerase II, 2 variant (Fragment) OS=H	0.930	1.037	0.677	0.601	0.857	1.232	0.944	0.899
P49366	Deoxyhypusine synthase OS=Homo sapiens GN=DHPS PE=1 SV=	1.131	0.723	0.872	0.581	0.913	0.897	0.823	0.653
Q96PU5	E3 ubiquitin-protein ligase NEDD4-like OS=Homo sapiens GN=NE	1.757	1.255	2.174	1.652	1.642	1.021	1.098	0.815
A0A024RDI1	Chromosome 4 open reading frame 16, isoform CRA_c OS=Homo	0.980	1.336	1.166	1.192	1.028	1.127	1.314	1.240
Q7Z7B1	Phosphatidylinositol-glycan biosynthesis class W protein OS=Hom	1.373	1.665	1.542	1.210	1.058	1.106	1.160	1.267
H3BSG0	UPF0183 protein C16orf70 (Fragment) OS=Homo sapiens GN=C1	1.811	1.235	1.895	1.613	1.652	1.157	1.439	1.404
Q9Y4F1	FERM, RhoGEF and pleckstrin domain-containing protein 1 OS=H	0.815	1.069	0.850	0.589	0.847	0.777	0.873	1.038
B4DPV1	cDNA FLJ51927, highly similar to Ribosomal protein S6 kinase alp	1.071	0.760		0.523	1.226		0.759	
Q3KQV9	UDP-N-acetylhexosamine pyrophosphorylase-like protein 1 OS=H	1.154	1.017	1.464	1.033	1.221	0.975	1.086	0.979
Q0D2N8	SARM1 protein (Fragment) OS=Homo sapiens GN=SARM1 PE=1	1.434	1.473	1.334	1.182	1.127	1.067	1.360	1.112
O43149	Zinc finger ZZ-type and EF-hand domain-containing protein 1 OS=	1.662	0.916	1.401	1.073	1.426	1.215	1.379	1.217
B3KU05	cDNA FLJ39026 fis, clone NT2RP7005675 OS=Homo sapiens PE=	1.496	1.115	1.222	1.071	1.244	1.060	1.285	1.168
Q96N21	AP-4 complex accessory subunit tepsin OS=Homo sapiens GN=EI	1.347		1.385	1.132	1.682			1.277
Q12986	Transcriptional repressor NF-X1 OS=Homo sapiens GN=NFX1 PE=	2.044	2.286	2.276	1.206	1.894	1.644	2.090	0.932
A8K7F3	cDNA FLJ75847, highly similar to Homo sapiens G protein-couplec	1.773	1.775	1.593	1.536	1.495	1.235	1.898	1.678
Q96HR8	H/ACA ribonucleoprotein complex non-core subunit NAF1 OS=Ho	0.498	0.699	0.489	0.422	1.051	1.463	0.837	0.597
Q4LE74	MYO9B variant protein (Fragment) OS=Homo sapiens GN=MYO9I	1.785	1.069	1.588	1.034	1.361	1.228	1.375	1.021
A8K4A2	cDNA FLJ76153, highly similar to Homo sapiens potassium chann	0.656	0.928	0.475	0.210	0.638	0.861	0.608	0.624
E5RIH5	TELO2-interacting protein 2 OS=Homo sapiens GN=TTI2 PE=1 S	1.400	1.034	1.319	1.188	1.411	1.350	1.136	0.956
Q9UPX8	SH3 and multiple ankyrin repeat domains protein 2 OS=Homo sa	1.620	4.184	3.540	2.925	1.443	1.168	3.142	1.262
Q9H4Z3	Phosphorylated CTD-interacting factor 1 OS=Homo sapiens GN=F	0.982	0.960	0.882	0.642	1.201	0.980	0.867	0.759
J3KNZ9	Probable fibrosin-1 OS=Homo sapiens GN=FBRS PE=1 SV=1 - [J	1.669	1.897	1.419		1.682	1.504	1.623	1.224
Q3ZTS5	BTB domain containing 1 OS=Homo sapiens GN=BTBD1 PE=2 SV	1.360	1.439	1.514	1.015	1.400	1.267	1.754	1.435
H7C5R8	Poly [ADP-ribose] polymerase 14 (Fragment) OS=Homo sapiens (	1.311	1.471	1.099	0.991	1.276	1.210	1.453	1.346
Q96L73	Histone-lysine N-methyltransferase, H3 lysine-36 and H4 lysine-2	1.222	2.124	1.247	0.865	1.231	1.655	1.625	1.116
Q9UJK0	Ribosome biogenesis protein TSR3 homolog OS=Homo sapiens G	1.293	0.924	1.059	0.641	1.125	1.133	1.157	0.986
D3DSM4	Collagen, type XVIII, alpha 1, isoform CRA_d OS=Homo sapiens	2.389	2.804	1.840	1.872	2.166	1.330	2.455	1.841
B3KSA6	cDNA FLJ35854 fis, clone TESTI2007113, highly similar to Afadin-	0.363	0.363	0.343	0.292	0.385	1.023	0.559	0.368
B4DS26	cDNA FLJ50636, highly similar to Transcription factor 8 OS=Homo	1.163	1.463	0.916	0.488	0.998	1.406	0.980	0.868
Q8N4X5	Actin filament-associated protein 1-like 2 OS=Homo sapiens GN=	1.354	0.836	1.035	0.725	1.243	1.070	0.817	0.920

Q8TAD4	Zinc transporter 5 OS=Homo sapiens GN=SLC30A5 PE=1 SV=1 -	1.403	1.688	1.277	1.372	0.998	1.532	1.648	1.392
Q68CP0	Putative uncharacterized protein DKFZp686B2325 (Fragment) OS	1.388	1.336	1.077	0.950	1.106	1.207	1.218	1.170
A5D8W8	Gamma-glutamyltransferase 7 OS=Homo sapiens GN=GGT7 PE=	2.150	1.848	2.560	2.569	1.803	1.826	1.577	
B8ZZ31	Phosphatidylinositol N-acetylglucosaminyltransferase subunit Q (F	0.935	0.981	1.047	1.039	0.886	1.007	1.219	0.918
A8K4U4	cDNA FLJ77113 OS=Homo sapiens PE=2 SV=1 - [A8K4U4_HUMA	1.331	0.941	1.148	0.998	1.248	1.055	1.093	0.999
Q14156	Protein EFR3 homolog A OS=Homo sapiens GN=EFR3A PE=1 SV=	1.835	1.736	0.720	1.270	1.122	1.207	1.871	1.497
Q330K2	NADH dehydrogenase (ubiquinone) complex I, assembly factor 6	1.138	1.513	1.029	1.334	1.332	1.556		1.530
O60566	Mitotic checkpoint serine/threonine-protein kinase BUB1 beta OS=	2.228	1.998	2.181	1.702	1.756	1.397	2.160	1.820
B4DIV9	cDNA FLJ61281, highly similar to Sodium/hydrogen exchanger 8 (	1.139	1.025		1.061	1.051	1.125	0.809	1.238
B3KQN7	cDNA FLJ90789 fis, clone THYRO1001523, highly similar to G-pro	1.238	1.553	0.982	0.665	1.364	1.275	1.004	0.733
O75182	Paired amphipathic helix protein Sin3b OS=Homo sapiens GN=SI	1.462	1.828	1.142	0.891	1.305	1.424	1.787	1.284
Q8N357	Solute carrier family 35 member F6 OS=Homo sapiens GN=SLC3!	1.851	1.569	1.603	1.581	1.896	1.205	1.583	1.779
D6W5D1	KIAA1212, isoform CRA_a OS=Homo sapiens GN=KIAA1212 PE=	1.614	1.300	1.450	1.249	1.238	1.147	1.365	1.349
Q6ZTE7	cDNA FLJ44731 fis, clone BRACE3025719, highly similar to Homo	1.115	1.030	1.207	0.714	0.987	1.243	1.296	1.175
Q6P0N0	Mis18-binding protein 1 OS=Homo sapiens GN=MIS18BP1 PE=1	1.076	1.406	1.013	0.563	0.873	1.227	1.224	0.875
P14384	Carboxypeptidase M OS=Homo sapiens GN=CPM PE=1 SV=2 - [(	0.367	0.406	0.298	0.252	0.501	0.893	0.369	0.304
Q96A19	Coiled-coil domain-containing protein 102A OS=Homo sapiens GN	0.954				0.668	1.066	1.023	
Q6NWY9	Pre-mRNA-processing factor 40 homolog B OS=Homo sapiens GN	0.982	0.963	0.820	0.684	0.839	1.071	0.840	0.797
B7ZLE5	FN1 protein OS=Homo sapiens GN=FN1 PE=2 SV=1 - [B7ZLE5_H	0.975	0.782	0.740	0.723	1.010	0.850	0.994	0.516
A0PJI7	CIR protein (Fragment) OS=Homo sapiens GN=CIR PE=2 SV=1 -	1.745	2.196	1.373	1.525	1.440	1.387	1.622	1.554
A0A024RBQ3	Calcium/calmodulin-dependent protein kinase kinase 2, beta, isof	0.588				0.876	1.022	0.826	
Q8TEU7	Rap guanine nucleotide exchange factor 6 OS=Homo sapiens GN	1.073	0.877	1.043	0.928	1.095	1.018	1.115	0.902
B2RCF3	Glycylpeptide N-tetradecanoyltransferase OS=Homo sapiens PE=:	1.258	1.041	1.095	0.598	0.936	1.022	1.044	0.959
Q92994	Transcription factor IIIB 90 kDa subunit OS=Homo sapiens GN=E	1.621	1.681	1.409	0.918	1.246	1.687	1.090	1.372
Q6P6B1	Glutamate-rich protein 5 OS=Homo sapiens GN=ERICH5 PE=2 S\	1.066	1.298	1.478	1.437	1.047	1.288	1.501	1.603
B4DI80	cDNA FLJ59530, highly similar to Protein O-mannosyl-transferase	1.907	1.592	1.416	1.140	1.408	1.239	1.614	1.251
Q96P48	Arf-GAP with Rho-GAP domain, ANK repeat and PH domain-conta	1.180	0.807	0.881	0.869	1.008	1.247	1.463	1.142
Q09472	Histone acetyltransferase p300 OS=Homo sapiens GN=EP300 PE=	1.281	1.318	1.010	0.734	1.293	1.212	1.145	0.788
E9PG22	Centrosomal protein of 97 kDa OS=Homo sapiens GN=CEP97 PE=	2.040	1.538	1.740	1.414	1.636	1.266	1.946	1.830
P19174	1-phosphatidylinositol 4,5-bisphosphate phosphodiesterase gamm	1.456	1.220	1.679	1.597	1.401	1.111	1.437	1.327
Q96ES6	Major facilitator superfamily domain-containing protein 3 OS=Hor	1.654	1.474	1.489	1.743	1.222	1.256	1.805	1.560
Q5JWM4	Threonine aspartase 1 (Fragment) OS=Homo sapiens GN=TASP1	1.215	1.690	1.208	0.950	2.039	1.511	1.980	1.191
B4E325	cDNA FLJ54048, highly similar to 55 kDa erythrocyte membrane p	1.512	1.563	1.731	1.104	0.811	1.081	1.516	1.419
Q2TA85	C10orf54 protein (Fragment) OS=Homo sapiens GN=C10orf54 PE	1.427	1.482	0.959	1.155	1.238	1.233	0.901	0.781
Q9Y672	Dolichyl pyrophosphate Man9GlcNAc2 alpha-1,3-glucosyltransfera	2.006	2.631	2.305	2.123	1.682	1.618	2.329	2.097
Q5T5U3	Rho GTPase-activating protein 21 OS=Homo sapiens GN=ARHGA	1.030	1.375	0.856	0.877	1.043	1.058	1.078	0.809
B7Z2D6	Cystathionine beta-synthase OS=Homo sapiens PE=2 SV=1 - [B7	2.001	1.310	1.595	1.321	1.704	0.931	1.241	1.285
A0A075B6H4	Cysteine-rich protein 2-binding protein OS=Homo sapiens GN=CS	1.681	1.930	1.329	1.376	0.960	2.257	1.457	1.541
J3QTA2	BAG family molecular chaperone regulator 1 OS=Homo sapiens G	1.980	2.440	3.240	2.531	1.807	1.158	2.319	1.871
A0A024R2T5	CUB domain containing protein 1, isoform CRA_a OS=Homo sapie	0.550	0.894	0.686	1.032	0.922	1.086	1.070	1.210

A0A087WXN9	MTSS1-like protein OS=Homo sapiens GN=MTSS1L PE=4 SV=1 -	0.911				1.147	1.382	0.798	
A0A087WUL7	Nuclear receptor-binding factor 2 OS=Homo sapiens GN=NRBF2	1.493	1.079	1.335	1.118	1.335	1.436	1.137	1.013
A0A024R8W1	Chromosome 17 open reading frame 70, isoform CRA_a OS=Hom	0.839	1.664	1.038	0.958	1.546	1.769	1.751	1.408
A6NML8	Diaphanous homolog 2 (Drosophila), isoform CRA_c OS=Homo sa	1.373	1.176	1.455	1.039	1.243	1.054	1.435	1.077
Q13017	Rho GTPase-activating protein 5 OS=Homo sapiens GN=ARHGAP	1.217	0.952	1.204	0.751	1.156	1.041	0.907	0.897
B3KQR3	cDNA PSEC0065 fis, clone NT2RP2000997, highly similar to Protei	0.243	0.394	0.391	0.262	0.502	1.170	0.498	0.338
P02765	Alpha-2-HS-glycoprotein OS=Homo sapiens GN=AHSG PE=1 SV=	1.244	0.209	0.833	0.424	1.877	1.212	1.103	0.765
B3KT69	cDNA FLJ37759 fis, clone BRHIP2023888 OS=Homo sapiens PE=	3.509	2.413	2.995	2.223	2.679	1.432	2.907	2.405
A0A024R637	TBC1 domain family, member 4, isoform CRA_b OS=Homo sapier	1.818	1.471	1.580	1.602	1.195	0.951	1.260	1.366
Q6XZF7	Dynamin-binding protein OS=Homo sapiens GN=DNMBP PE=1 S\	1.141	0.605	0.820	0.578	1.275	0.846	0.840	0.812
D3DVC4	Nestin, isoform CRA_c OS=Homo sapiens GN=NES PE=3 SV=1 -	2.265	2.672	1.588	2.112	1.554	1.282	1.735	1.109
E5KS22	Mitochondrial DNA polymerase subunit gamma-2 OS=Homo sapie	1.194	1.332	1.178	0.990	1.116	1.147	1.243	0.962
L7P8G6	Methylenetetrahydrofolate reductase OS=Homo sapiens GN=MTH	1.552	1.801	1.192	1.796	1.402	1.711	2.072	1.593
P05997	Collagen alpha-2(V) chain OS=Homo sapiens GN=COL5A2 PE=1	1.036	0.588	0.617	0.322	0.933	0.891	0.801	0.555
A8K5S1	cDNA FLJ78650, highly similar to Homo sapiens mucosa associate	1.274	0.983	2.054	1.948	1.280	0.976	1.009	0.859
Q8N2H3	Pyridine nucleotide-disulfide oxidoreductase domain-containing pr	1.827	1.627	2.081	1.651	1.386	1.425	1.713	1.225
B4DTH9	cDNA FLJ53190, highly similar to Beta-glucuronidase (EC 3.2.1.31	1.626	1.310	1.979	1.587	1.544	1.319	2.120	1.904
B3KUN5	cDNA FLJ40308 fis, clone TESTI2029264, highly similar to Proteir	1.762	1.410	1.752	1.466	1.477	1.410	1.781	1.657
Q92793	CREB-binding protein OS=Homo sapiens GN=CREBBP PE=1 SV=:	0.856	0.983	0.688	0.525	0.850	1.160	0.937	0.792
A0AVN2	BRCA1 associated RING domain 1 OS=Homo sapiens GN=BARD1	1.951	2.316	3.211	5.425	1.847	1.643	2.418	3.906
Q2NL81	Sema domain, immunoglobulin domain (Ig), transmembrane dom	0.713	0.925	0.637	0.687	0.702	0.707	0.493	0.411
A8K720	cDNA FLJ75040, highly similar to Homo sapiens serum response t	1.078	1.077	0.893		1.029	1.566	0.931	1.009
Q53SW3	Putative uncharacterized protein DPYSL5 (Fragment) OS=Homo s	1.228	0.964	1.606	1.269	1.041	1.062	1.622	1.516
Q8NHM5	Lysine-specific demethylase 2B OS=Homo sapiens GN=KDM2B PE	1.060	1.590	1.491	1.326	1.028	1.337	1.230	1.647
B7ZLI0	Reticulon 4 receptor-like 2 OS=Homo sapiens GN=RTN4RL2 PE=:	1.179	1.347	0.590	0.635	1.038			
Q8TDR2	Serine/threonine-protein kinase 35 OS=Homo sapiens GN=STK35		1.158						
Q8TEQ8	GPI ethanolamine phosphate transferase 3 OS=Homo sapiens GN	1.625	1.693	1.399	1.429	1.516	1.271	1.679	1.425
Q16519	Protein S (Fragment) OS=Homo sapiens GN=PROS1 PE=4 SV=1	1.114	1.111	0.832	1.001	1.293	0.684	0.892	0.993
B7ZKS3	Ubiquitin specific peptidase 48 OS=Homo sapiens GN=USP48 PE=	1.399	1.358	1.137	0.826	1.564	1.230	1.118	0.921
Q13546	Receptor-interacting serine/threonine-protein kinase 1 OS=Homo						1.004		
Q92845	Kinesin-associated protein 3 OS=Homo sapiens GN=KIFAP3 PE=1	1.627	1.237	1.609	1.511	1.727	0.997	1.596	1.093
A0A087X0S5	Collagen alpha-1(VI) chain OS=Homo sapiens GN=COL6A1 PE=4	0.912	0.736	1.570	1.413	1.080	1.329	1.693	1.517
Q9UJY5	ADP-ribosylation factor-binding protein GGA1 OS=Homo sapiens (	1.630	1.198	1.519	1.233	1.440	0.983	1.276	1.298
B7Z7G0	cDNA FLJ53676, highly similar to Leucine-rich repeat-containing p	1.354	1.969	1.264	1.508	1.374	1.680	1.770	1.220
Q14686	Nuclear receptor coactivator 6 OS=Homo sapiens GN=NCOA6 PE	1.225	1.326	0.847	0.717	0.903	1.135	1.136	0.939
Q9P2D1	Chromodomain-helicase-DNA-binding protein 7 OS=Homo sapien	1.018	1.698	1.383	1.268	0.803	1.412	1.497	1.577
Q8IWQ7	CDC42-binding protein kinase beta OS=Homo sapiens GN=CDC4:	1.413	0.773	0.664	0.332	0.983	1.161	1.079	0.763
Q9H6R7	WD repeat-containing protein C2orf44 OS=Homo sapiens GN=C2	2.381	1.888	2.577	2.456	1.502	0.998	1.871	1.551
P40189	Interleukin-6 receptor subunit beta OS=Homo sapiens GN=IL6ST	1.002	0.725	0.290	0.167	0.970	0.977	0.600	0.342
P21953	2-oxoisovalerate dehydrogenase subunit beta, mitochondrial OS=	1.645	1.829	1.451	1.744	1.240	1.265	1.858	1.883

Q9UPY3	Endoribonuclease Dicer OS=Homo sapiens GN=DICER1 PE=1 SV	2.063	1.533	1.429	1.150	1.677	1.086	1.099	1.053
Q9UBK8	Methionine synthase reductase OS=Homo sapiens GN=MTRR PE=		0.693	1.016				0.982	
I3L4J3	KAT8 regulatory NSL complex subunit 1 OS=Homo sapiens GN=K	1.221	1.306	0.680	0.488	1.195	1.194	1.181	0.682
Q16254	Transcription factor E2F4 OS=Homo sapiens GN=E2F4 PE=1 SV=	0.642	0.688	0.666	0.492	0.707	1.039	0.913	1.071
Q9Y6R9	Coiled-coil domain-containing protein 61 OS=Homo sapiens GN=	0.501	0.487	0.613	0.498	0.717	0.899	0.564	0.525
Q9NNW5	WD repeat-containing protein 6 OS=Homo sapiens GN=WDR6 PE	1.224	1.026	1.149	0.871	1.262	1.237	1.531	1.111
Q9NSY1	BMP-2-inducible protein kinase OS=Homo sapiens GN=BMP2K PE	1.644	1.580	1.308	1.225	1.131	1.004	1.631	1.120
Q9BQS8	FYVE and coiled-coil domain-containing protein 1 OS=Homo sapie	2.187	1.841	2.502	2.044	1.952	1.221	2.338	1.756
Q53FS1	Carbohydrate (Chondroitin 4) sulfotransferase 12 variant (Fragme	0.907	1.161	1.116	1.034	0.916	1.028	1.216	0.994
Q7Z4C7	MSTP128 OS=Homo sapiens PE=2 SV=1 - [Q7Z4C7_HUMAN]	3.594	3.168	2.935	3.008	2.265	1.922	2.658	2.925
A0A024R9E6	TAF2 RNA polymerase II, TATA box binding protein (TBP)-associa	0.896	1.159	1.152	0.987	0.863	1.187	1.284	1.139
B7ZLC8	JMJD1C protein OS=Homo sapiens GN=JMJD1C PE=2 SV=1 - [B	1.211	1.788	0.795	0.586	1.021	1.277	1.020	0.989
B4DW34	cDNA FLJ56798, highly similar to Acid sphingomyelinase-like phos	1.471	1.256	1.267	1.292	1.114	1.270	1.413	1.329
B4DL36	cDNA FLJ55706 OS=Homo sapiens PE=2 SV=1 - [B4DL36_HUMA	1.456	1.064	0.907	0.936	0.996	0.985	1.100	0.992
B7Z444	cDNA FLJ60653 OS=Homo sapiens PE=2 SV=1 - [B7Z444_HUMA	1.294	1.373	1.042	1.254	1.386	1.209	1.389	1.020
Q6ZMP0	Thrombospondin type-1 domain-containing protein 4 OS=Homo s	0.316	0.382			0.432	0.882	0.449	0.145
A0A024QZD9	Zinc finger protein 668, isoform CRA_a OS=Homo sapiens GN=Z	1.628	1.552	1.081	1.238	1.135	1.554	1.290	1.257
B4E140	Transporter OS=Homo sapiens PE=2 SV=1 - [B4E140_HUMAN]	2.327	2.998	1.592	1.496	2.165	1.399	1.818	1.536
Q96AE7	Tetratricopeptide repeat protein 17 OS=Homo sapiens GN=TTC17	1.053	1.449	1.102	0.972	1.186	1.608	1.089	0.982
Q8N3F8	MICAL-like protein 1 OS=Homo sapiens GN=MICALL1 PE=1 SV=2	1.184	0.878	0.851	0.755	0.937	0.973	1.031	0.709
Q658V6	Putative uncharacterized protein DKFZp666G172 (Fragment) OS=	1.778	1.609	1.314	1.291	1.635	1.201	1.845	1.502
Q96G28	Coiled-coil domain-containing protein 104 OS=Homo sapiens GN=	0.863	0.682	1.254	0.877	0.977	0.942	0.889	1.047
Q8IWV7	E3 ubiquitin-protein ligase UBR1 OS=Homo sapiens GN=UBR1 PE	5.155	3.222	5.483	2.732	2.064	1.898	2.439	2.968
Q9NS87	Kinesin-like protein KIF15 OS=Homo sapiens GN=KIF15 PE=1 SV	1.432	0.907	1.158	0.947	1.116	1.168	1.102	0.672
B3KWZ7	cDNA FLJ44373 fis, clone TRACH3009455, highly similar to Phosp	0.952	0.640		0.608	0.544		0.693	0.547
Q9C0D5	Protein TANC1 OS=Homo sapiens GN=TANC1 PE=1 SV=3 - [TAN	1.422	1.142	1.267	0.814	1.110	1.049	0.835	0.998
Q96RF0	Sorting nexin-18 OS=Homo sapiens GN=SNX18 PE=1 SV=2 - [SN	1.392	1.036	1.085	0.811	0.986	1.125	1.310	1.077
Q5T3J3	Ligand-dependent nuclear receptor-interacting factor 1 OS=Homo	0.985	1.150	0.715	0.529	0.788	1.222	0.822	0.769
H3BTR4	Centromere protein T OS=Homo sapiens GN=CENPT PE=1 SV=1	1.129	1.943	1.608	1.037	1.062	1.326	1.291	1.647
B3KNP9	cDNA FLJ30114 fis, clone BNGH42000532, highly similar to Zinc f	0.746	0.838	0.651	0.532	0.616	0.891	0.803	0.707
Q9BRS2	Serine/threonine-protein kinase RIO1 OS=Homo sapiens GN=RIO	1.224	1.000	1.257	0.911	1.274	1.124	1.025	1.010
P0C7T5	Ataxin-1-like OS=Homo sapiens GN=ATXN1L PE=1 SV=1 - [ATX1	1.115	1.314	0.943	0.677	1.180	1.317	1.065	0.979
Q15699	ALX homeobox protein 1 OS=Homo sapiens GN=ALX1 PE=1 SV=	0.569	0.526						
Q9UNA1	Rho GTPase-activating protein 26 OS=Homo sapiens GN=ARHGA	1.364	1.054	1.515	1.083	1.269	1.159	1.174	1.297
B3KXZ9	cDNA FLJ46477 fis, clone THYMU3025118, highly similar to Cell s		2.114		1.368	1.259	1.858	2.129	2.452
O15056	Synaptojanin-2 OS=Homo sapiens GN=SYNJ2 PE=1 SV=3 - [SYN	1.375	1.050	1.056	0.839	1.340	0.893	0.941	0.888
Q6PHR2	Serine/threonine-protein kinase ULK3 OS=Homo sapiens GN=ULK			1.246	0.580	0.495	1.077	0.791	1.093
Q9H5K3	Protein O-mannose kinase OS=Homo sapiens GN=POMK PE=1 S	1.978	2.003	1.483	1.341	1.659	1.551	1.447	1.188
Q96HP0	Dedicator of cytokinesis protein 6 OS=Homo sapiens GN=DOCK6						1.055		
H0YA68	Alpha-mannosidase (Fragment) OS=Homo sapiens GN=MAN2B2	0.909	0.976	0.922	1.054	0.901	0.798	1.143	1.141

A0A024RCG1	Chromosome X open reading frame 34, isoform CRA_c OS=Homo	1.871	1.371			1.526	1.718	1.963	1.710
Q9HCG4	KIAA1608 protein (Fragment) OS=Homo sapiens GN=KIAA1608 I	1.375	1.052	0.974			1.408	1.394	1.563
B4E0A0	cDNA FLJ51613, highly similar to Homo sapiens cytochrome P450						1.098		
Q68CU9	Acetyl-coenzyme A synthetase (Fragment) OS=Homo sapiens GN	1.324	0.981	1.460	1.178	1.420	1.044	1.019	1.064
B7Z3E3	Reticulon OS=Homo sapiens PE=2 SV=1 - [B7Z3E3_HUMAN]	2.121	2.334	2.023	2.257	1.916	1.507	2.295	2.326
P28749	Retinoblastoma-like protein 1 OS=Homo sapiens GN=RBL1 PE=1	1.733	1.700	1.279	0.790	1.589	2.589	1.478	1.159
E7EN73	Dyslexia-associated protein KIAA0319-like protein (Fragment) OS	1.392	1.541	1.274	1.103	1.317	1.080	1.119	1.303
Q5VWN6	Protein FAM208B OS=Homo sapiens GN=FAM208B PE=1 SV=1 -	1.140	1.370	1.254	1.084	1.014	1.190	1.173	1.343
B2RDK5	cDNA, FLJ96655, highly similar to Homo sapiens stromal membra	2.534	1.447	2.455	1.942	1.769	1.353	2.204	2.049
A7E234	ZNF644 protein OS=Homo sapiens GN=ZNF644 PE=2 SV=1 - [A7	0.961	1.599	0.971	1.183	0.952	1.505	1.351	1.278
A0A087WZG9	Retrotransposon-derived protein PEG10 OS=Homo sapiens GN=P	2.506	1.316	1.433		1.789	1.030	1.243	
B4E2H5	cDNA FLJ58741, highly similar to JmjC domain-containing histone	1.220	1.565	1.021	0.613	1.368	1.318	1.296	0.655
Q96HE9	Proline-rich protein 11 OS=Homo sapiens GN=PRR11 PE=1 SV=1	1.303	1.896	1.099	0.696	1.263	1.248	1.309	1.085
Q9Y6K1	DNA (cytosine-5)-methyltransferase 3A OS=Homo sapiens GN=D	2.567	1.730	2.033	2.919	2.312	1.789	3.084	2.389
B2RWP4	TACC2 protein OS=Homo sapiens GN=TACC2 PE=2 SV=1 - [B2RV	1.371	1.059	1.157	0.741	1.364	0.935	1.047	0.768
Q59H82	PR-domain zinc finger protein 2 variant (Fragment) OS=Homo sa	1.606	2.176	1.696	1.185	1.088	1.780	1.702	1.769
H0UI63	Zinc finger, NFX1-type containing 1, isoform CRA_b OS=Homo sa	1.313	0.745	0.838	0.523	0.932	0.968	0.965	0.671
B4DZ85	Nuclear receptor coactivator 4 OS=Homo sapiens GN=NCOA4 PE	2.180	1.676			2.235	1.784	1.055	0.452
E7EWP2	Triple functional domain protein OS=Homo sapiens GN=TRIO PE=	1.234	1.145	1.163	0.918	1.648	1.173	0.926	0.799
A8K942	cDNA FLJ76454, highly similar to Homo sapiens ring finger protei	0.934	1.141	0.905	0.833	0.865	1.438	1.439	1.251
B3KST1	cDNA FLJ36882 fis, clone BLADE2000340, highly similar to Zinc fi	1.124							
B4DMX1	cDNA FLJ58025, highly similar to Homo sapiens mitochondrial tra	0.962	1.042	0.834	0.834	0.965	1.108	0.973	0.868
O75420	PERQ amino acid-rich with GYF domain-containing protein 1 OS=	1.438	1.016	1.364	0.855	1.254	1.007	1.172	0.932
Q8NAF0	Zinc finger protein 579 OS=Homo sapiens GN=ZNF579 PE=1 SV=	0.983	0.518	0.281	0.265	0.733	0.908	0.871	0.746
Q96ST8	Centrosomal protein of 89 kDa OS=Homo sapiens GN=CEP89 PE=	0.969	1.467	1.360	0.830	1.078	1.119	1.229	1.199
P21675	Transcription initiation factor TFIID subunit 1 OS=Homo sapiens (	1.178	1.112	1.136	0.880	1.031	1.197	1.242	1.221
Q32P44	Echinoderm microtubule-associated protein-like 3 OS=Homo sapi	2.383	2.126	2.256	2.048	1.786	1.736	1.549	1.614
A0A024R307	Three prime repair exonuclease 1, isoform CRA_d OS=Homo sapi	1.369	1.867	1.307	1.089	1.408	1.688	1.414	1.202
Q9H7F4	Transmembrane protein 185B OS=Homo sapiens GN=TMEM185B	1.397	1.932	1.179	0.845	1.450	2.023	1.718	1.046
D6RJG9	Sorting nexin-14 (Fragment) OS=Homo sapiens GN=SNX14 PE=4	1.310	1.278	0.844	0.916	0.971	1.079	0.936	0.899
Q9UL54	Serine/threonine-protein kinase TAO2 OS=Homo sapiens GN=TA(	1.068	1.355	1.013	0.907	1.121	1.143	1.352	1.151
Q8WYQ5	Microprocessor complex subunit DGCR8 OS=Homo sapiens GN=D	1.152	1.943	1.654	1.041	1.443	1.726	2.008	1.394
A8K6L3	cDNA FLJ76883, highly similar to Homo sapiens FKSG44 gene (FK	1.953	2.250	2.647	2.299			2.015	
Q96Q15	Serine/threonine-protein kinase SMG1 OS=Homo sapiens GN=SM	1.334	0.996	1.060	0.883	1.185	1.093	1.089	0.908
Q8TCN5	Zinc finger protein 507 OS=Homo sapiens GN=ZNF507 PE=1 SV=	1.184	1.357	1.084	0.875		1.307	1.354	1.124
B3KXK4	cDNA FLJ45605 fis, clone BRTHA3021971, highly similar to Nucle	1.483	0.906	1.047		1.018	0.936	0.977	0.854
H0Y3N9	Histone lysine demethylase PHF8 (Fragment) OS=Homo sapiens (	1.105	0.929			0.627			
B2R6A9	cDNA, FLJ92868, highly similar to Homo sapiens HIRA interacting	1.498	1.232	1.254	0.899	1.586	1.182	1.280	1.163
Q9NUU6	Inactive ubiquitin thioesterase FAM105A OS=Homo sapiens GN=F	1.714	1.970	2.059	1.821	1.441	1.577	2.684	2.095
B7Z4Q5	Receptor-type tyrosine-protein phosphatase OS=Homo sapiens PI	2.255	2.661	1.720	1.621	1.754	1.490	1.986	2.062

Q9H2D6	TRIO and F-actin-binding protein OS=Homo sapiens GN=TRIOBP	1.342	0.995	1.023	0.903	1.105	1.094	1.005	0.924
P05121	Plasminogen activator inhibitor 1 OS=Homo sapiens GN=SERPINI	2.712	0.972	0.327		3.008	1.197	0.639	0.408
Q6UB28	Methionine aminopeptidase 1D, mitochondrial OS=Homo sapiens	1.121	1.137	1.126	0.852	0.875	1.322	1.159	1.084
A0A087WWW8	Calcineurin-binding protein cabin-1 OS=Homo sapiens GN=CABIN	1.230	1.172	0.796	0.863	1.086	1.307	0.899	1.070
Q9UHV7	Mediator of RNA polymerase II transcription subunit 13 OS=Hom	1.225	1.616	0.779	0.856	1.601	1.317	1.507	1.334
Q6AHY3	Putative uncharacterized protein DKFZp686G10254 (Fragment) O	1.211	1.182	0.975	1.048	1.027	1.260	1.241	0.939
B4DTP6	cDNA FLJ56908, highly similar to MORC family CW-type zinc finge						1.069		
O95365	Zinc finger and BTB domain-containing protein 7A OS=Homo sap	2.031	2.396	1.965	1.824	1.646	1.153	1.968	2.187
Q6UWJ1	Transmembrane and coiled-coil domain-containing protein 3 OS=	1.276	1.255	1.366	1.536	1.265	1.084	1.430	1.138
B4E2D5	Transcription factor SPT20 homolog OS=Homo sapiens GN=SUPT					1.100	0.999		
H3BNC0	Protein arginine N-methyltransferase 7 OS=Homo sapiens GN=PF	1.502	1.080	1.788	1.405	1.231	1.129	1.606	1.468
Q86XA9	HEAT repeat-containing protein 5A OS=Homo sapiens GN=HEATF	1.198	0.842	0.960	0.754	1.114	0.875	0.953	0.943
Q9Y2F5	Little elongation complex subunit 1 OS=Homo sapiens GN=ICE1	1.488	1.817	1.258	0.851	1.474	1.610	1.654	1.337
B9EG70	Nuclear protein, ataxia-telangiectasia locus OS=Homo sapiens GN	1.246	1.803	1.103	0.851	0.714	1.466	1.669	1.249
B4DTZ7	cDNA FLJ57146, highly similar to Homo sapiens LMBR1 domain c	1.213	1.265	1.225	0.992		1.211	1.313	1.296
Q96EK7	Constitutive coactivator of peroxisome proliferator-activated recep	1.210	1.333	1.144	0.955	1.028	1.172	1.086	0.656
E9PM19	Tyrosine-protein kinase OS=Homo sapiens GN=TYK2 PE=1 SV=1	1.842	2.967	2.082	1.981	1.954	2.014	1.878	1.845
B3KTA8	cDNA FLJ37971 fis, clone CTONG2009958, highly similar to CERU	2.419	1.615	1.684	1.428	1.588	1.019	1.572	1.302
Q9C026	E3 ubiquitin-protein ligase TRIM9 OS=Homo sapiens GN=TRIM9	1.498	0.919	0.908	0.866	1.350	0.908	0.935	0.886
B4DQP4	cDNA FLJ51279, highly similar to Homo sapiens zinc finger protei	1.065	0.804	0.876		0.826	0.974	0.684	0.311
Q5VSY0	G kinase-anchoring protein 1 OS=Homo sapiens GN=GKAP1 PE=	1.124	1.251	1.428	0.904	1.188	1.146	1.364	1.184
Q8N5S9	Calcium/calmodulin-dependent protein kinase kinase 1 OS=Homo	0.667							
P35568	Insulin receptor substrate 1 OS=Homo sapiens GN=IRS1 PE=1 S	1.366	0.468	0.447	0.542	1.038	0.577	0.413	0.439
E7ESG2	Claspin OS=Homo sapiens GN=CLSPN PE=1 SV=1 - [E7ESG2_HU	1.194	1.464	1.151	0.574	1.138	1.809	1.072	0.833
A0A024R4H4	Kinesin family member 1B, isoform CRA_b OS=Homo sapiens GN	1.136		1.144	0.862	0.800	1.049	0.966	0.995
A8K702	cDNA FLJ78285, highly similar to Homo sapiens gasdermin doma	0.784	0.509	0.836	0.681	0.755	1.020	0.781	0.617
Q7RTS9	Dymeclin OS=Homo sapiens GN=DYM PE=1 SV=1 - [DYM_HUMA	1.878	1.593	1.682	1.401	1.628	1.124	1.686	1.467
B7WNX0	Histone-lysine N-methyltransferase SUV420H1 OS=Homo sapiens	1.174	1.996	1.341	0.955	1.183	1.486	1.173	0.994
M0QZS0	Rho guanine nucleotide exchange factor 18 (Fragment) OS=Hom	1.495	0.945	1.103	0.909	1.162	1.090	1.218	0.944
Q9H792	Pseudopodium-enriched atypical kinase 1 OS=Homo sapiens GN=	0.935	1.279	1.081	0.649	0.866	1.308	1.132	0.897
Q5TGY3	AT-hook DNA-binding motif-containing protein 1 OS=Homo sapier	1.052	1.523	1.796	1.159	1.656	1.447	0.989	2.004
Q9C0B7	Transport and Golgi organization protein 6 homolog OS=Homo sa	1.428	1.168	1.600	1.186	1.364	1.287	1.499	1.280
Q58WW2	DDB1- and CUL4-associated factor 6 OS=Homo sapiens GN=DCA	1.238	0.768	0.876	0.615	1.113	1.077	0.909	0.780
B7Z499	cDNA FLJ50571, highly similar to Myotubularin (EC 3.1.3.48) OS=	1.069	0.990			1.038	1.754	0.635	
P22681	E3 ubiquitin-protein ligase CBL OS=Homo sapiens GN=CBL PE=1	1.598	1.063	1.100	1.085	0.990	0.903	1.049	1.074
Q96IV0	Peptide-N(4)-(N-acetyl-beta-glucosaminyl)asparagine amidase OS	0.728	0.560	0.687	0.444	0.866	0.975	0.693	0.593
P26374	Rab proteins geranylgeranyltransferase component A 2 OS=Homo	0.754	0.621	0.822	0.683	1.111	1.084	0.951	0.688
Q07954	Prolow-density lipoprotein receptor-related protein 1 OS=Homo s	0.978	0.965	0.875	0.850	0.942	0.902	0.989	1.069
Q8NCN4	E3 ubiquitin-protein ligase RNF169 OS=Homo sapiens GN=RNF16	1.198	1.170	0.685	0.316	0.798	1.207	1.010	0.836
Q9BV73	Centrosome-associated protein CEP250 OS=Homo sapiens GN=C	0.942	0.822	1.185	1.080	0.783	0.881	0.968	1.069

O60476	Mannosyl-oligosaccharide 1,2-alpha-mannosidase IB OS=Homo s	1.361	1.576	1.428	1.258	1.193	1.129	1.292	1.103
Q8N0Z3	Spindle and centriole-associated protein 1 OS=Homo sapiens GN=	1.401	1.853	1.239	1.343	1.228	1.465	1.660	1.533
B7Z3T1	cDNA FLJ50782, highly similar to Receptor-type tyrosine-protein	1.723	0.702	1.322	0.727	1.569	1.320	1.000	1.429
O96006	Zinc finger BED domain-containing protein 1 OS=Homo sapiens G	3.195	3.689	2.021	1.241	3.260	1.975	2.853	2.014
B7ZML1	CHD9 protein OS=Homo sapiens GN=CHD9 PE=2 SV=1 - [B7ZML	1.615	1.789	1.440	1.276	1.365	1.752	1.425	1.355
A8K973	cDNA FLJ77998, highly similar to Homo sapiens synaptotagmin-li	0.365	0.411	0.447	0.418	0.546	0.988	0.557	0.520
O43150	Arf-GAP with SH3 domain, ANK repeat and PH domain-containing	0.829	0.819	0.712	0.529	1.031	1.379	0.895	0.637
Q7KYR7	Butyrophilin subfamily 2 member A1 OS=Homo sapiens GN=BTN	1.396	1.816	1.251	1.534	1.277	2.055	1.791	1.855
Q8WY91	THAP domain-containing protein 4 OS=Homo sapiens GN=THAP	1.443	2.369	1.862	1.699	1.396	1.773	2.051	1.859
O75751	Solute carrier family 22 member 3 OS=Homo sapiens GN=SLC22	0.864	1.083	0.710	0.670	0.845	0.921	0.837	0.638
Q9NTJ4	Alpha-mannosidase 2C1 OS=Homo sapiens GN=MAN2C1 PE=1 S	1.372	0.950	1.240	0.840	1.256	1.080	0.933	0.946
D3DRG7	Dihydropyrimidinase-like 4, isoform CRA_a OS=Homo sapiens GN	1.093		1.500				1.274	1.646
B2RUU3	Dedicator of cytokinesis 1 OS=Homo sapiens GN=DOCK1 PE=2 S	0.203	0.182	0.184	0.145	0.266	0.770	0.252	0.167
O75113	NEDD4-binding protein 1 OS=Homo sapiens GN=N4BP1 PE=1 SV					1.496			
B2RBY8	cDNA, FLJ95771, highly similar to Homo sapiens ectonucleotide p	0.637	0.930	1.004	1.148	0.825	1.474	1.439	1.022
A0A024R984	HCG40889, isoform CRA_a OS=Homo sapiens GN=hCG_40889 P	1.145	1.069	0.811	0.877	1.228	1.040	1.073	0.895
P05543	Thyroxine-binding globulin OS=Homo sapiens GN=SERPINA7 PE=	2.082		1.146		1.973			
B7ZLW1	CAMSAP1 protein OS=Homo sapiens GN=CAMSAP1 PE=2 SV=1 -	1.597	1.070	1.078	0.931	1.136	1.114	1.053	1.107
B2REA4	PR domain zinc finger protein 1 (Fragment) OS=Homo sapiens G	1.315	1.323	1.159	1.154	1.055	1.073	1.291	1.100
E9PJ42	Transmembrane protein 41B OS=Homo sapiens GN=TMEM41B P	1.022	1.105	0.861	0.792	0.841	1.164	0.957	0.790
Q8NF91	Nesprin-1 OS=Homo sapiens GN=SYNE1 PE=1 SV=4 - [SYNE1_H	1.358	1.333	1.040	0.925	1.229	1.633	1.030	1.168
B1AKL4	Eukaryotic translation initiation factor 4E transporter OS=Homo s	0.915	0.951	0.866	0.684	1.219	1.574	0.999	0.732
Q5T985	Inter-alpha-trypsin inhibitor heavy chain H2 OS=Homo sapiens G	2.016	0.509	1.498	0.949	1.725	1.104	1.201	1.181
C9J5X9	Fanconi anemia group B protein (Fragment) OS=Homo sapiens G	2.165	2.272	1.311	1.868	1.834	1.790	1.489	1.866
Q13395	Probable methyltransferase TARBP1 OS=Homo sapiens GN=TAR	1.149	0.991	1.074	0.820	1.027	1.006	1.175	0.944
A0A024R9L9	Vacuolar protein sorting 39 (Yeast), isoform CRA_b OS=Homo sa	1.148	1.823	2.151	1.099	2.183	1.193	1.104	1.208
C9JJN9	Vacuolar protein sorting-associated protein 8 homolog OS=Homo	1.797	1.172	1.463	1.298	1.812	1.007	1.378	1.750
Q96RS0	Trimethylguanosine synthase OS=Homo sapiens GN=TGS1 PE=1	1.483	0.955	1.408	0.789	1.180	1.071	1.276	0.910
Q9H8Y5	Ankyrin repeat and zinc finger domain-containing protein 1 OS=H	1.897	1.610	1.300	0.920	1.504	1.193	1.409	0.959
Q99985	Semaphorin-3C OS=Homo sapiens GN=SEMA3C PE=1 SV=2 - [S	1.027				0.695			
B4DMX4	cDNA FLJ57154, highly similar to Alpha-fetoprotein OS=Homo sa	1.844	0.449	1.122	0.706	1.617	1.050	1.123	1.026
A0A024R734	Nuclear factor of activated T-cells 5, tonicity-responsive, isoform	1.336	0.864	0.809	0.653	1.235	0.795	0.803	0.735
P78357	Contactin-associated protein 1 OS=Homo sapiens GN=CNTNAP1	0.781	0.585	0.678	0.729	0.583	0.941	0.471	0.597
Q9NPG3	Ubinuclein-1 OS=Homo sapiens GN=UBN1 PE=1 SV=2 - [UBN1_	0.863	1.170	0.560	0.598	0.859	1.075	0.765	0.681
Q5H9F3	BCL-6 corepressor-like protein 1 OS=Homo sapiens GN=BCORL1	0.896	1.829	1.146	0.799	1.003	1.144	1.323	1.241
Q9P2R6	Arginine-glutamic acid dipeptide repeats protein OS=Homo sapien	1.502	1.565	1.123	0.828	1.701	1.012	1.556	1.458
B4E0V7	cDNA FLJ51709, highly similar to Myotubularin-related protein 9	1.326	0.802	0.977	1.056	0.859	0.852	1.249	1.081
Q4ZG32	Putative uncharacterized protein EPB41L5 (Fragment) OS=Homo	1.358	1.200	1.183	1.013	1.199	1.094	1.257	1.257
Q5SRI9	Glycoprotein endo-alpha-1,2-mannosidase OS=Homo sapiens GN	1.484	1.723	1.389	1.436	1.132	1.534	1.557	1.407
Q8TF46	DIS3-like exonuclease 1 OS=Homo sapiens GN=DIS3L PE=1 SV=	1.726	1.372	1.552	0.781	1.529	1.650	1.329	1.148

Q86X52	Chondroitin sulfate synthase 1 OS=Homo sapiens GN=CHSY1 PE=	0.829	1.076	1.075	1.202	0.880	1.161	1.211	1.034
Q5JWZ0	PHD finger protein 20 (Fragment) OS=Homo sapiens GN=PHF20	1.405	1.347	0.950	0.507	1.485	1.683	1.365	1.079
B4DVC8	cDNA FLJ56250 OS=Homo sapiens PE=2 SV=1 - [B4DVC8_HUMA		1.126	0.731	0.692	0.874	0.980	0.834	0.766
P08572	Collagen alpha-2(IV) chain OS=Homo sapiens GN=COL4A2 PE=1	1.607	1.405	1.124	1.039	1.468	1.684	1.800	1.399
O43314	Inositol hexakisphosphate and diphosphoinositol-pentakisphospha	0.950	1.024	0.898	0.701	0.793	0.987	0.931	0.977
B4DET6	cDNA FLJ59314, highly similar to UV radiation resistance-associat	1.197	1.000	1.015	1.059	1.242		1.337	0.991
Q5T3F8	CSC1-like protein 2 OS=Homo sapiens GN=TMEM63B PE=1 SV=1	1.900	1.984	1.622	1.415	1.760	1.464	1.603	1.410
P29375	Lysine-specific demethylase 5A OS=Homo sapiens GN=KDM5A PE	1.189	1.420	0.799	0.694	0.960	1.151	1.153	0.893
B3KQD7	cDNA FLJ90282 fis, clone NT2RP1000551, highly similar to Interfe	1.342	1.123	1.461	1.111	1.131	1.124	1.424	1.707
A4D2N7	Ubiquitin carboxyl-terminal hydrolase OS=Homo sapiens GN=USP	1.830	2.108	1.565	1.620	1.449	1.517	1.633	1.187
H0YBR0	Trafficking protein particle complex subunit 9 (Fragment) OS=Hor	1.543	1.000	1.231	0.508	1.164	0.998	1.340	1.440
B4E216	cDNA FLJ57339, highly similar to Complement C3 OS=Homo sapi	2.761	0.859	1.846	1.382	3.426	1.295	2.232	1.751
P38935	DNA-binding protein SMUBP-2 OS=Homo sapiens GN=IGHMBP2 P	1.295	1.608	1.433	1.063	1.017	1.108	1.364	1.158
Q9NZN5	Rho guanine nucleotide exchange factor 12 OS=Homo sapiens GN	0.871	0.929	0.847	0.912	1.095	0.964	0.897	0.907
Q13641	Trophoblast glycoprotein OS=Homo sapiens GN=TPBG PE=1 SV=	1.289	1.036	0.385	0.307	1.298	0.949	0.793	0.598
J3QLU9	Receptor tyrosine-protein kinase erbB-2 OS=Homo sapiens GN=E	1.515	1.713	1.372	1.328	1.427	1.237	1.357	1.206
Q9ULG1	DNA helicase INO80 OS=Homo sapiens GN=INO80 PE=1 SV=2 -	1.013	1.456	1.210	1.150	1.372	1.145	1.184	1.157
Q8N655	Uncharacterized protein C10orf12 OS=Homo sapiens GN=C10orf1	1.165	1.886	1.137	1.137	1.092	1.346	1.413	1.250
B4DJU9	cDNA FLJ55278, highly similar to AF4/FMR2 family member 1 OS	1.416	1.812	1.631	1.144	1.087	1.157	1.452	1.337
Q9GZV5	WW domain-containing transcription regulator protein 1 OS=Hom	2.546	1.313	1.440	1.165	2.130		1.214	0.985
B7Z450	cDNA FLJ51888, highly similar to Maternal embryonic leucine zipp	0.752	0.742	0.664	0.469	0.800	0.979	1.138	0.777
B3KW08	cDNA FLJ41852 fis, clone NT2RI3004133, highly similar to Export	1.327	0.811	1.638	0.889	1.714	1.346	1.661	1.153
P50443	Sulfate transporter OS=Homo sapiens GN=SLC26A2 PE=1 SV=2	0.990	1.497	0.932	0.839	1.198	1.070	1.085	0.886
Q58EX2	Protein sidekick-2 OS=Homo sapiens GN=SDK2 PE=1 SV=3 - [SD	0.725	0.941	0.773	0.834	0.608	1.016	0.882	1.050
Q6R327	Rapamycin-insensitive companion of mTOR OS=Homo sapiens GN	2.315	1.440	1.156	1.606	1.618	1.198	1.702	1.707
Q03164	Histone-lysine N-methyltransferase 2A OS=Homo sapiens GN=KM	1.070	1.426	1.254	1.241	1.015	1.086	1.130	1.012
Q01973	Tyrosine-protein kinase transmembrane receptor ROR1 OS=Homo	1.462	1.134	0.654	0.633	1.535	1.221	1.059	1.072
Q8WYL5	Protein phosphatase Slingshot homolog 1 OS=Homo sapiens GN=		1.046	1.184		1.240	1.309		
Q9ULW8	Protein-arginine deiminase type-3 OS=Homo sapiens GN=PADI3						0.656		
H3BRJ5	Uncharacterized protein OS=Homo sapiens PE=4 SV=1 - [H3BRJ5	1.848	1.292	2.479		1.507	1.537	1.135	
B4DHJ4	cDNA FLJ57931, highly similar to MORC family CW-type zinc finge	2.261	2.447	1.896		1.439	1.260	1.901	0.908
O75191	Xylulose kinase OS=Homo sapiens GN=XYLB PE=1 SV=3 - [XYLB	1.519	1.207	1.576	1.186	1.226	1.408	1.373	1.359
H0YIQ8	KAT8 regulatory NSL complex subunit 2 (Fragment) OS=Homo sa	1.670	1.650	0.971	0.640	1.541	1.371	1.464	1.033
A2AJT9	Uncharacterized protein CXorf23 OS=Homo sapiens GN=CXorf23	0.309	0.302	0.224	0.129	0.533	0.712	0.525	0.312
O94915	Protein furry homolog-like OS=Homo sapiens GN=FRYL PE=1 SV	1.226	1.280	1.008	0.721	1.248	1.284	1.006	0.867
Q5T321	Neurobeachin OS=Homo sapiens GN=NBEA PE=1 SV=1 - [Q5T32	3.316	3.042	3.397	2.380	1.842	0.673	2.420	1.987
Q8WXB7	X-linked zinc finger protein (Fragment) OS=Homo sapiens GN=ZF	1.160	1.374	1.112	0.562	1.153	0.942	1.109	0.782
A0A024R863	Mitogen-activated protein kinase associated protein 1, isoform CR	1.450	1.106	1.193	0.805	0.998	0.821	0.901	0.882
D6PAV9	Intersectin 1 short form A variant 2 OS=Homo sapiens GN=ITSN1		0.829	0.842	0.818	0.811	0.754	0.748	0.560
A0A087X0P0	Centromere-associated protein E OS=Homo sapiens GN=CENPE F	1.481	1.781	1.289	1.152	1.319	1.989	2.156	1.156

B4E3U6	cDNA FLJ58239, highly similar to Zinc finger protein 317 OS=Hor						1.430	0.834	0.531
Q96S94	Cyclin-L2 OS=Homo sapiens GN=CCNL2 PE=1 SV=1 - [CCNL2_H	1.460	1.706				1.538	1.285	
Q8IWU2	Serine/threonine-protein kinase LMTK2 OS=Homo sapiens GN=LI	1.227	2.168	1.432	1.360	0.910	1.505	1.620	1.761
B3KXK0	cDNA FLJ45575 fis, clone BRTHA3011194, highly similar to Chlori	1.018	0.924	1.009	0.888	0.959	1.060	1.114	0.789
B2R636	Ubiquitin carboxyl-terminal hydrolase OS=Homo sapiens PE=2 SV	0.875	0.983	0.608	0.406	1.296	1.621	1.148	0.602
A0A087WY08	Thrombospondin type-1 domain-containing protein 7A OS=Homo	1.199	1.145	0.822	0.604	1.031	0.917	0.925	0.590
O75843	AP-1 complex subunit gamma-like 2 OS=Homo sapiens GN=AP1G	2.036	0.998	1.473		1.457	1.307	1.763	1.128
Q92625	Ankyrin repeat and SAM domain-containing protein 1A OS=Homo	1.056	1.169	1.257	1.163	1.356	1.184	1.313	1.337
A0JNW5	UHRF1-binding protein 1-like OS=Homo sapiens GN=UHRF1BP1L	1.141	0.724	0.760	0.782	1.215	0.743	1.011	0.723
Q5QP82	DDB1- and CUL4-associated factor 10 OS=Homo sapiens GN=DC	1.070	1.277	0.971		0.779	1.162	1.185	0.730
A0JLU2	BUB1 protein (Fragment) OS=Homo sapiens GN=BUB1 PE=2 SV=	1.104	1.657	1.448	0.633	1.205	1.907	2.767	1.628
X5D7Q2	Tuberous sclerosis 2 isoform D (Fragment) OS=Homo sapiens GN	1.369	0.999	1.156	0.941	1.146	0.983	0.874	0.605
Q3B7T1	Erythroid differentiation-related factor 1 OS=Homo sapiens GN=E		1.093			1.284		1.462	0.847
Q9NPA5	Zinc finger protein 64 homolog, isoforms 1 and 2 OS=Homo sapie	2.700	1.325	1.371	1.166	0.979	1.528	1.339	1.368
Q9UIW2	Plexin-A1 OS=Homo sapiens GN=PLXNA1 PE=1 SV=3 - [PLXA1_H	1.162	1.511	1.105	1.290	1.086	1.435	1.226	1.073
A6XGL3	Protease serine 1 OS=Homo sapiens GN=PRSS1 PE=2 SV=1 - [A				1.209	1.024		1.119	
Q6NSJ0	Uncharacterized family 31 glucosidase KIAA1161 OS=Homo sapie	2.073	1.989	1.280	1.623	1.245	1.548	1.852	1.170
Q9Y2Z2	Protein MTO1 homolog, mitochondrial OS=Homo sapiens GN=MT	1.787	1.609	1.531	1.335	1.409	1.452	1.758	1.498
Q9P2K8	Eukaryotic translation initiation factor 2-alpha kinase 4 OS=Homo	1.189	0.797	1.162	0.772	1.100	0.916	1.028	0.838
Q8WXG6	MAP kinase-activating death domain protein OS=Homo sapiens G	1.359	0.807	0.777	0.791	1.013	1.018	0.898	0.729
O14686	Histone-lysine N-methyltransferase 2D OS=Homo sapiens GN=KN	1.225	1.421	1.068	0.653	1.050	1.275	0.948	1.268
A0A087WYE2	Probable inactive glycosyltransferase 25 family member 3 OS=Ho	1.255	0.996	0.986	0.652	1.146	1.088	1.218	1.284
Q6ZU80	Centrosomal protein of 128 kDa OS=Homo sapiens GN=CEP128 I	1.187	1.731	1.594	1.696	1.311	1.463	1.653	2.257
A8KA68	cDNA FLJ75833, highly similar to Homo sapiens membrane-bound	1.456	1.966	1.740	1.801	1.127	1.312	1.476	1.797
O95243	Methyl-CpG-binding domain protein 4 OS=Homo sapiens GN=MB	1.686	1.511	1.203	1.013	1.224	1.214	0.988	0.818
Q71F56	Mediator of RNA polymerase II transcription subunit 13-like OS=H	2.214	2.071	1.055	0.609	2.002	1.151	1.509	0.918
Q6P4A8	Phospholipase B-like 1 OS=Homo sapiens GN=PLBD1 PE=1 SV=2	1.367	1.331	1.504	1.345	1.562	1.343	1.753	1.757
Q66GS9	Centrosomal protein of 135 kDa OS=Homo sapiens GN=CEP135 I	1.729	1.803	1.471	1.420	1.457	1.404	1.631	1.737
A0A087WU78	Nance-Horan syndrome protein OS=Homo sapiens GN=NHS PE=4	1.522	0.853	1.217	0.880	1.213	1.021	1.186	1.009
Q96NW4	Ankyrin repeat domain-containing protein 27 OS=Homo sapiens G	1.382	1.296	1.073	1.188	1.209	1.378	1.314	1.199
P55268	Laminin subunit beta-2 OS=Homo sapiens GN=LAMB2 PE=1 SV=	0.977	0.760	0.458	0.317	1.060	0.958	0.850	0.483
O15085	Rho guanine nucleotide exchange factor 11 OS=Homo sapiens GI	1.226	1.305	1.306	1.048	1.175	1.239	1.304	1.255
M0R0W6	Tyrosine-protein kinase receptor UFO OS=Homo sapiens GN=AXL	1.347	1.058	0.902		1.304	0.804	1.055	0.813
P01023	Alpha-2-macroglobulin OS=Homo sapiens GN=A2M PE=1 SV=3 -	1.803	0.342	1.018	0.427	1.522	0.883	0.948	0.791
Q9Y5T5	Ubiquitin carboxyl-terminal hydrolase 16 OS=Homo sapiens GN=U	1.427	1.208	1.318	1.153	1.160	1.024	1.355	1.282
A0A067XG54	Phospholipid-transporting ATPase IG OS=Homo sapiens GN=ATP1	1.814	1.410	1.238	1.519	1.389	1.203	1.676	1.657
A8K251	cDNA FLJ77884 OS=Homo sapiens PE=2 SV=1 - [A8K251_HUMA						1.098		
Q8NCW6	Polypeptide N-acetylgalactosaminyltransferase 11 OS=Homo sapi	2.331	1.748	1.589	1.506	2.066	1.328	1.687	1.243
Q6P4F7	Rho GTPase-activating protein 11A OS=Homo sapiens GN=ARHG	1.401	1.497			1.375	1.293	1.247	0.751
X5D2T7	Seizure related 6-like protein 2 isoform E (Fragment) OS=Homo s	1.758	1.237	1.297	1.241			1.784	1.919

A2A2F0	Ral GTPase-activating protein subunit beta (Fragment) OS=Homo	1.954	1.587	1.770	1.245	1.624	0.998	2.040	1.492
Q5SW02	Zinc finger MYM-type protein 1 OS=Homo sapiens GN=ZMYM1 PI	1.543	2.252	1.438	1.499	1.510	1.450	2.074	1.309
Q5JC44	KLHL9 protein OS=Homo sapiens PE=2 SV=1 - [Q5JC44_HUMAN	2.234	2.555	2.481	1.722	1.802	1.732	2.215	1.516
Q5VUA4	Zinc finger protein 318 OS=Homo sapiens GN=ZNF318 PE=1 SV=	0.721	0.677	0.283		0.843	0.848	0.424	0.308
B2RXG1	SLC45A4 protein OS=Homo sapiens GN=SLC45A4 PE=2 SV=1 - [	1.037	1.063	1.015	0.759	0.997	1.169	0.921	0.763
Q53SQ1	Putative uncharacterized protein R3HDM (Fragment) OS=Homo s	1.036	0.844	0.953	0.813	0.804	0.802	1.101	0.848
Q86YV5	Tyrosine-protein kinase SgK223 OS=Homo sapiens GN=SGK223 F	2.142	1.217	1.187	0.436	1.544	1.091	1.484	0.891
A8K1E4	cDNA FLJ77411, highly similar to Homo sapiens gamma-tubulin c	1.091	0.891	0.934	0.693	1.066	0.891	0.936	0.727
B4DR21	cDNA FLJ58156, highly similar to Membrane-bound transcription		0.363	0.350	0.204	0.825	0.918	0.636	0.343
Q7Z7M9	Polypeptide N-acetylgalactosaminyltransferase 5 OS=Homo sapie	2.299	2.943	2.434	2.323	2.290	1.499	2.437	2.023
A0A087WWH3	Glioma tumor suppressor candidate region gene 1 protein OS=Ho		1.378	0.742	0.886	1.169		0.968	1.229
P55201	Peregrin OS=Homo sapiens GN=BRPF1 PE=1 SV=2 - [BRPF1_HU	1.351	1.667	1.245	1.376	1.083	1.511	1.439	1.528
B2R7F8	cDNA, FLJ93426, highly similar to Homo sapiens plasminogen (PL				1.320	0.851			
Q9UKA4	A-kinase anchor protein 11 OS=Homo sapiens GN=AKAP11 PE=1	1.560	2.091	2.468	1.664	1.415	1.437	2.118	1.696
Q9UPR3	Protein SMG5 OS=Homo sapiens GN=SMG5 PE=1 SV=3 - [SMG5	1.992	1.857	2.121		2.273	1.771	2.175	2.032
Q6NYC8	Phostensin OS=Homo sapiens GN=PPP1R18 PE=1 SV=1 - [PPR18	2.035	2.163	1.395	1.008	1.859	2.272	1.695	1.489
Q15911	Zinc finger homeobox protein 3 OS=Homo sapiens GN=ZFHX3 PE	1.055	1.291	0.801	0.540	1.034	1.111	1.003	0.828
B2RBH9	cDNA, FLJ95516 OS=Homo sapiens PE=2 SV=1 - [B2RBH9_HUM	1.418	1.167	0.775		1.571	1.149	0.874	0.457
A8K9T5	E3 ubiquitin-protein ligase OS=Homo sapiens PE=2 SV=1 - [A8K9	0.502	0.766			0.592	0.941	0.503	
A0A024RDI4	Ankyrin 2, neuronal, isoform CRA_a OS=Homo sapiens GN=ANK2	1.536	1.783	1.694	1.261	2.269	2.568	2.322	2.452
Q9NUA8	Zinc finger and BTB domain-containing protein 40 OS=Homo sapi	0.945	1.866	1.273	1.022	0.891	1.771	1.508	1.242
Q99708	DNA endonuclease RBBP8 OS=Homo sapiens GN=RBBP8 PE=1 S	1.114	1.034	1.056	0.777	1.428	1.085	1.374	1.075
B2R787	cDNA, FLJ93330, highly similar to Homo sapiens phosphatidylinos	1.499	1.052	0.970	1.027	1.221	1.221	1.712	1.139
Q53T94	TATA box-binding protein-associated factor RNA polymerase I sub	0.915	0.938			0.985	1.337	0.703	0.896
A8K5X3	cDNA FLJ77553 OS=Homo sapiens PE=2 SV=1 - [A8K5X3_HUMA	1.782	2.549	1.879	1.465	1.479	1.196	1.715	1.436
Q3T906	N-acetylglucosamine-1-phosphotransferase subunits alpha/beta C	0.927	0.988	0.944	0.857	1.661	1.460	1.274	1.167
Q99650	Oncostatin-M-specific receptor subunit beta OS=Homo sapiens GI	1.768	1.289	0.465	0.399	1.715	1.041	0.971	
Q6UB99	Ankyrin repeat domain-containing protein 11 OS=Homo sapiens (	0.907	1.239	0.754	0.431	0.773	0.963	1.091	0.616
B4DGQ2	cDNA FLJ51835, highly similar to DNA-repair protein complement	2.276	1.653			1.873	1.216	1.248	
Q9NYQ6	Cadherin EGF LAG seven-pass G-type receptor 1 OS=Homo sapie	2.051	2.157	2.398	2.188	1.374	1.655	1.373	1.256
Q9UBY0	Sodium/hydrogen exchanger 2 OS=Homo sapiens GN=SLC9A2 PI		1.457	0.789	1.021	1.003	1.449	1.564	0.985
Q9H2Y7	Zinc finger protein 106 OS=Homo sapiens GN=ZNF106 PE=1 SV=	1.877	2.693	1.213	0.822	1.457	1.766	1.970	0.870
Q14517	Protocadherin Fat 1 OS=Homo sapiens GN=FAT1 PE=1 SV=2 - [F	1.307	1.194	1.230	0.944	1.400	1.248	1.415	1.154
B2R7A1	cDNA, FLJ93348, highly similar to Homo sapiens protein phospha	1.381	1.605	0.926		1.079	1.491	2.202	1.370
Q13315	Serine-protein kinase ATM OS=Homo sapiens GN=ATM PE=1 SV=	1.863	1.568	1.677	1.207	1.927	1.311	1.448	1.545
Q7Z2K8	G protein-regulated inducer of neurite outgrowth 1 OS=Homo sap	1.495	2.058	1.457	1.334	1.262	1.221	1.592	1.295
C9J5N2	Beta/gamma crystallin domain-containing protein 3 OS=Homo sa	1.432	1.268	1.308	1.290	1.255	1.084	1.330	1.174
O95613	Pericentrin OS=Homo sapiens GN=PCNT PE=1 SV=4 - [PCNT_HU	1.645	1.713	1.759	1.354	1.759	1.424	1.515	1.414
Q8WUI4	Histone deacetylase 7 OS=Homo sapiens GN=HDAC7 PE=1 SV=2	0.917	0.753	0.762	0.527	1.150	1.133	0.916	0.568
O75592	E3 ubiquitin-protein ligase MYCBP2 OS=Homo sapiens GN=MYCB	1.114	1.012	0.790	0.634	0.929	0.930	0.879	0.706

B4E3Q1	cDNA FLJ61580, highly similar to Calsyntenin-1 OS=Homo sapien	3.101	4.378	2.710	1.533	1.639	2.237	3.373	2.595
Q9Y2J4	Angiomotin-like protein 2 OS=Homo sapiens GN=AMOTL2 PE=1 S	1.142	0.649		0.596	1.245		0.533	
A8K2T0	cDNA FLJ78471 OS=Homo sapiens PE=2 SV=1 - [A8K2T0_HUMA	0.792	0.838	0.688	0.571	0.643	0.898	0.728	0.776
A8K855	EF-hand calcium-binding domain-containing protein 7 OS=Homo	1.111	1.302	0.762	0.909	1.089	1.123	0.961	0.994
J3KNV1	Zinc finger protein 292 OS=Homo sapiens GN=ZNF292 PE=1 SV=			1.242			1.931	1.513	1.362
A4PB67	YY1AP-related protein1 OS=Homo sapiens PE=2 SV=1 - [A4PB67	1.221	1.587	1.124	0.969	1.036	1.444	1.520	0.918
Q9Y4E6	WD repeat-containing protein 7 OS=Homo sapiens GN=WDR7 PE	1.203	0.645	0.906	0.764	0.786	0.931	0.938	0.873
B2R9G9	cDNA, FLJ94385, highly similar to Homo sapiens cofactor require	2.058	2.774	2.168	1.242	1.596	1.786	2.233	1.564
Q9NVE5	Ubiquitin carboxyl-terminal hydrolase 40 OS=Homo sapiens GN=U	2.017	1.392	1.745	1.381	1.953	1.258	1.836	1.264
G3V1N5	Ubiquitin carboxyl-terminal hydrolase 28 OS=Homo sapiens GN=U	1.028	0.902	0.691	0.527	0.771	0.950	0.870	0.353
Q9HCD6	Protein TANC2 OS=Homo sapiens GN=TANC2 PE=1 SV=3 - [TAN	1.232	0.973	0.940	0.705	0.899	0.835	0.834	0.776
Q15311	RalA-binding protein 1 OS=Homo sapiens GN=RALBP1 PE=1 SV=	1.679	1.284	1.553	1.218	1.787	1.245	1.214	1.262
B2RDI7	cDNA, FLJ96629, highly similar to Homo sapiens ring finger prote	1.693	1.153	1.720	1.670	1.202	0.826	1.592	1.448
D6RJI3	Fibrillin-2 OS=Homo sapiens GN=FBN2 PE=4 SV=1 - [D6RJI3_HU	1.401	1.520	0.992				0.890	0.841
Q08E86	KIAA0100 protein OS=Homo sapiens GN=KIAA0100 PE=1 SV=1	1.869	1.889	1.308	1.222	1.790	1.563	1.859	1.837
B4DZN1	cDNA FLJ56624, highly similar to Histone deacetylase 6 OS=Hom	1.422	1.004	1.419	0.977	1.162	1.001	1.215	0.983
P42858	Huntingtin OS=Homo sapiens GN=HTT PE=1 SV=2 - [HD_HUMA	1.530	1.211	1.151			1.406	1.431	1.506
Q8TD26	Chromodomain-helicase-DNA-binding protein 6 OS=Homo sapien	1.127	1.214	0.944	0.735	1.012	0.936	1.062	0.991
Q9C0I1	Myotubularin-related protein 12 OS=Homo sapiens GN=MTMR12	1.074	0.801	0.908	0.827	0.878	0.850	0.840	0.936
Q9ULJ6	Zinc finger MIZ domain-containing protein 1 OS=Homo sapiens G	1.260	1.130	0.999	0.668	1.374	1.076	1.027	0.802
Q562E7	WD repeat-containing protein 81 OS=Homo sapiens GN=WDR81	1.322	0.947	1.038	0.876	1.058	0.946	0.990	0.681
P35869	Aryl hydrocarbon receptor OS=Homo sapiens GN=AHR PE=1 SV=	0.782	0.590	0.313		0.719	0.660	0.594	0.278
Q9P1Y5	Calmodulin-regulated spectrin-associated protein 3 OS=Homo sa		1.744	1.585	0.938	1.043			0.978
Q16787	Laminin subunit alpha-3 OS=Homo sapiens GN=LAMA3 PE=1 SV=	1.239	1.140	1.106	0.604	1.159	0.972	1.399	1.097
Q8TDR0	TRAF3-interacting protein 1 OS=Homo sapiens GN=TRAF3IP1 PE	0.970	0.680	0.784				0.782	0.741
Q9UGL1	Lysine-specific demethylase 5B OS=Homo sapiens GN=KDM5B PE	2.533	2.750	1.891	0.998	2.085	1.963	1.730	
B4DIT2	cDNA FLJ60067, highly similar to Oligophrenin 1 OS=Homo sapie		2.585				1.995		
A0A024R8K8	Helicase with zinc finger, isoform CRA_a OS=Homo sapiens GN=H	1.889	2.316	1.969	0.925	2.035	1.830	2.075	1.707
H0YKX0	E3 ubiquitin-protein ligase RNF31 (Fragment) OS=Homo sapiens	1.119	0.852	1.172		1.071	0.985	1.022	0.913
Q86YT6	E3 ubiquitin-protein ligase MIB1 OS=Homo sapiens GN=MIB1 PE	0.849	0.924	0.666	0.499	1.024	1.040	0.864	0.673
Q86SQ4	G-protein coupled receptor 126 OS=Homo sapiens GN=GPR126 P	1.414	1.374			1.133			
Q9UHP3	Ubiquitin carboxyl-terminal hydrolase 25 OS=Homo sapiens GN=U	1.125	1.127	1.130	0.893	1.079	1.055	1.122	1.096
A6QL63	Ankyrin repeat and BTB/POZ domain-containing protein BTBD11 (	1.466	1.254	1.317	1.008	1.389	0.852	1.358	1.231
O60447	Ecotropic viral integration site 5 protein homolog OS=Homo sapie	1.413	1.602	0.870	1.337	0.905	0.929	1.451	1.122
G8JLP4	Meiosis arrest female protein 1 OS=Homo sapiens GN=KIAA0430	1.162	1.173	0.815	0.717	1.137	1.098	0.809	0.796
Q9BUM1	Glucose-6-phosphatase 3 OS=Homo sapiens GN=G6PC3 PE=1 SV	1.150	1.105	0.972	0.940	1.241	1.022	1.184	0.884
Q8NG31	Protein CASC5 OS=Homo sapiens GN=CASC5 PE=1 SV=3 - [CAS(	1.033	1.034	0.827	0.765	1.092	1.248	1.113	0.958
Q9NS15	Latent-transforming growth factor beta-binding protein 3 OS=Hor	2.458	2.592	2.362	2.206	2.386	1.003	2.543	2.549
Q6IQ32	ADNP homeobox protein 2 OS=Homo sapiens GN=ADNP2 PE=1 S	1.613	1.614	1.418	0.837	1.366	1.149	1.572	1.653
Q5T5P2	Sickle tail protein homolog OS=Homo sapiens GN=KIAA1217 PE=	1.212	1.252	0.907	0.712	1.028	0.758	0.722	0.811

Q9H6A9	Pecanex-like protein 3 OS=Homo sapiens GN=PCNXL3 PE=1 SV=						1.472	1.396	
Q92766	Ras-responsive element-binding protein 1 OS=Homo sapiens GN=	1.197	0.675			1.041	1.160	1.063	0.806
B3KNW3	cDNA FLJ30571 fis, clone BRAWH2006044, highly similar to Intra	1.596	1.333	1.203	0.937	1.242	1.193	1.393	1.105
P01031	Complement C5 OS=Homo sapiens GN=C5 PE=1 SV=4 - [CO5_H	0.889	0.810	0.613	0.780	1.034	0.954	0.837	0.819
F1T0K4	DmX-like protein 1 OS=Homo sapiens GN=DMXL1 PE=2 SV=1 - [		0.504	0.524	0.406	0.632	0.732	1.101	1.015
A0A087WVU5	B-cell CLL/lymphoma 9 protein OS=Homo sapiens GN=BCL9 PE=	0.747	0.928	0.547	0.613	0.895	1.029	0.983	0.764
O43306	Adenylate cyclase type 6 OS=Homo sapiens GN=ADCY6 PE=1 SV	0.736	0.956	0.716	0.623	0.625	1.076	0.809	0.554
A0A024R998	Protein phosphatase 1 regulatory subunit 12 OS=Homo sapiens G	0.897	1.108	0.914	0.786	1.069	1.257	1.056	0.797
Q7Z7M0	Multiple epidermal growth factor-like domains protein 8 OS=Hom	0.971	0.820	0.967	0.669	0.906	0.839	0.872	0.929
B7ZVX0	DOCK9 protein OS=Homo sapiens GN=DOCK9 PE=2 SV=1 - [B7Z	2.695	1.801			2.446			
Q8IYH5	ZZ-type zinc finger-containing protein 3 OS=Homo sapiens GN=Z		1.705	0.995			1.977		
O94887	FERM, RhoGEF and pleckstrin domain-containing protein 2 OS=H	2.466	3.703	2.338	2.298	1.860	0.900	2.259	2.120
Q9UHJ3	Scm-like with four MBT domains protein 1 OS=Homo sapiens GN=	1.640	1.976	1.420	1.240	1.062	0.968	1.354	1.434
H0YG46	M-phase phosphoprotein 9 (Fragment) OS=Homo sapiens GN=M			0.698	0.623	1.237	1.156	1.293	
Q6GYQ0	Ral GTPase-activating protein subunit alpha-1 OS=Homo sapiens	1.647	1.289	1.301				1.542	
A0A024R914	Centrosomal protein 350kDa, isoform CRA_a OS=Homo sapiens G	0.885	1.244	0.777	0.634	0.992	1.216	1.179	1.047
Q59GL0	Rearranged L-myc fusion sequence variant (Fragment) OS=Homo	1.622	1.848	2.044	1.352	1.309	1.440	1.309	1.449
A1A4S6	Rho GTPase-activating protein 10 OS=Homo sapiens GN=ARHGA	0.843	0.732	0.865	0.623	0.804	0.849	1.054	1.178
Q8IY92	Structure-specific endonuclease subunit SLX4 OS=Homo sapiens	1.029	1.387	0.816		0.872	1.163	1.155	1.166
Q6ZT12	E3 ubiquitin-protein ligase UBR3 OS=Homo sapiens GN=UBR3 PE	1.337	1.291	1.514	0.935	1.144	1.214	1.145	1.142
Q5TAX3	Terminal uridylyltransferase 4 OS=Homo sapiens GN=ZCCHC11 P	1.067	0.782	1.002	0.673	0.963	0.902	1.141	0.767
A0A024R856	HCG96198, isoform CRA_a OS=Homo sapiens GN=hCG_96198 PE	1.249	1.500	1.113	0.814	0.928	1.201	1.191	1.264
O95248	Myotubularin-related protein 5 OS=Homo sapiens GN=SBF1 PE=	0.871					0.730		
D3DVH3	Inositol polyphosphate-4-phosphatase, type I, 107kDa, isoform C	1.904	1.655	1.575	0.898	1.502	0.992	1.468	1.143
Q8IZT6	Abnormal spindle-like microcephaly-associated protein OS=Homo	1.315	1.469	0.836	0.741	1.183	1.143	1.003	1.099
B4E3H3	Phosphoinositide phospholipase C OS=Homo sapiens PE=2 SV=1	1.093	0.868	0.872	0.867	0.694	0.555	1.006	0.864
Q15751	Probable E3 ubiquitin-protein ligase HERC1 OS=Homo sapiens GN	1.053	0.885	0.956	0.887	1.096	1.080	1.111	0.741
Q9BXB1	Leucine-rich repeat-containing G-protein coupled receptor 4 OS=	1.236	1.072	0.854	0.656	1.253	0.705	0.670	0.501
B3KWN0	cDNA FLJ43389 fis, clone OCBBF2007068, highly similar to Ankyr						0.987		
A0A024R3H2	Sortilin-related receptor, L(DLR class) A repeats-containing, isofor	1.028	1.099		0.686		1.221	1.029	0.689
Q9UBF8	Phosphatidylinositol 4-kinase beta OS=Homo sapiens GN=PI4KB	1.161	1.132	1.068	0.900	1.102	1.116	1.220	0.961
Q8TDJ6	DmX-like protein 2 OS=Homo sapiens GN=DMXL2 PE=1 SV=2 - [	1.336	0.922	1.457	0.733	1.285	0.783	1.108	0.967
Q96BY7	Autophagy-related protein 2 homolog B OS=Homo sapiens GN=A	1.704	1.480	2.212	1.486	1.683	1.121	1.752	1.558
Q92859	Neogenin OS=Homo sapiens GN=NEO1 PE=1 SV=2 - [NEO1_HU	1.172	1.902	0.972	0.904	0.891	1.146	1.074	0.982
Q4KWH8	1-phosphatidylinositol 4,5-bisphosphate phosphodiesterase eta-1	1.078	0.762	1.027	0.716	0.979	1.014	1.015	0.779
A1YBP1	Breast and ovarian cancer susceptibility protein 2 truncated variar	1.198	2.268				1.712	1.491	1.714
Q92545	Transmembrane protein 131 OS=Homo sapiens GN=TMEM131 PE	1.125	1.125	1.079	0.788	0.709	1.090	1.114	0.864
Q96PE2	Rho guanine nucleotide exchange factor 17 OS=Homo sapiens GI	1.602	1.660	1.517	1.255	1.476	1.748	1.730	1.364
Q7Z7G8	Vacuolar protein sorting-associated protein 13B OS=Homo sapien	1.626	2.798	2.202	1.886	2.059	1.930	2.140	1.812
Q59H55	Protein tyrosine phosphatase, non-receptor type 13 isoform 2 var		1.098		1.017	1.199			

O94854	Uncharacterized protein KIAA0754 OS=Homo sapiens GN=KIAA0	0.706	1.137	0.872				0.969	
Q12767	Uncharacterized protein KIAA0195 OS=Homo sapiens GN=KIAA0	1.248	1.355	1.205	1.088	0.880	1.121	1.372	1.267
A8K0I1	Diacylglycerol kinase OS=Homo sapiens PE=2 SV=1 - [A8K0I1_H	1.358	0.659	0.871	0.753	1.183	0.980	1.117	0.879
Q59EX8	Low density lipoprotein receptor-related protein 6 variant (Fragme	0.782	1.204			0.720	0.894	0.965	
O15078	Centrosomal protein of 290 kDa OS=Homo sapiens GN=CEP290 I	0.738	0.844	0.863	0.716	1.213	1.201	1.500	0.925
Q9UIF8	Bromodomain adjacent to zinc finger domain protein 2B OS=Hom	1.122	1.040	0.704	0.786	0.985	1.030	0.913	0.781
Q13433	Zinc transporter ZIP6 OS=Homo sapiens GN=SLC39A6 PE=1 SV=	0.696	0.940	0.675	0.686	0.718	0.989	0.858	0.762
A0A024RAB6	Heparan sulfate proteoglycan 2 (Perlecan), isoform CRA_b OS=H					1.784	1.458	1.101	
Q9NR48	Histone-lysine N-methyltransferase ASH1L OS=Homo sapiens GN	0.606	0.989			0.903	1.264	0.705	0.500
Q8TEK3	Histone-lysine N-methyltransferase, H3 lysine-79 specific OS=Hor	1.315	2.110	1.335	1.438	1.193	1.744	1.581	2.071
F5H7B7	ATP-binding cassette sub-family A member 13 (Fragment) OS=H	0.116	0.161	0.105	0.113	0.153	0.855	0.151	0.132
Q5FWF5	N-acetyltransferase ESCO1 OS=Homo sapiens GN=ESCO1 PE=1 S	2.231	2.226	2.108	1.381	1.958	1.325	2.119	1.759
Q6ZNJ1	Neurobeachin-like protein 2 OS=Homo sapiens GN=NBEAL2 PE=	1.202	0.603	1.018	0.721	1.140	1.015	1.081	0.707
Q8NEZ4	Histone-lysine N-methyltransferase 2C OS=Homo sapiens GN=KM	1.199	2.747	2.324	2.410	2.656	2.005	2.347	2.499
A0A087WTM7	Apolipoprotein B-100 OS=Homo sapiens GN=APOB PE=4 SV=1 -	5.809	3.113	2.780	2.768	4.958	1.424	2.585	1.905
C4B7M2	Spatacsin OS=Homo sapiens GN=SPG11 PE=1 SV=1 - [C4B7M2_	1.138	1.021	0.881	0.701	0.766		0.681	0.819
A0A087WV90	Dystrophin OS=Homo sapiens GN=DMD PE=4 SV=1 - [A0A087W	0.289	0.223	0.328	0.256	0.407	0.831	0.370	0.337
Q5T011	Protein SZT2 OS=Homo sapiens GN=SZT2 PE=2 SV=3 - [SZT2_H	1.063	0.662	1.372		1.087			
B0I1S0	DYNC2H1 variant protein OS=Homo sapiens PE=2 SV=1 - [B0I1S	1.600	1.669	1.354	1.074	1.441	1.297	1.455	1.432

Table 2S. Reactome biological pathway analysis of affected proteins

Pathway name	Ad5								dl1520							
	6 h		12 h		18 h		24 h		6 h		12 h		18 h		24 h	
	Count	Percent of total	Count	Percent of total	Count	Percent of total	Count	Percent of total	Count	Percent of total	Count	Percent of total	Count	Percent of total	Count	Percent of total
Chromatin organization	12	5.2	14	6.1	7	3.0	8	3.5	6	2.6	3	1.3	8	3.5	8	3.5
DNA Replication	5	4.6	8	7.4	6	5.6	5	4.6	2	1.9	2	1.9	5	4.6	7	6.5
Cell Cycle	26	4.4	28	4.7	29	4.9	25	4.2	13	2.2	6	1.0	30	5.0	33	5.5
Metabolism of proteins	59	4.3	57	4.2	48	3.5	42	3.1	36	2.7	4	0.3	51	3.8	44	3.2
Cellular responses to stress	16	4.3	20	5.4	19	5.1	16	4.3	10	2.7	4	1.1	19	5.1	21	5.7
Gene Expression	69	3.9	62	3.5	64	3.6	44	2.5	38	2.2	5	0.3	58	3.3	57	3.2
Organelle biogenesis and maintenance	13	3.8	11	3.2	6	1.7	5	1.4	4	1.2	0	0.0	5	1.4	5	1.4
Programmed Cell Death	6	3.5	6	3.5	5	2.9	4	2.3	3	1.8	0	0.0	2	1.2	5	2.9
Hemostasis	21	3.5	25	4.2	16	2.7	18	3.0	14	2.3	2	0.3	16	2.7	16	2.7
DNA Repair	9	3.1	13	4.4	15	5.1	11	3.8	7	2.4	2	0.7	7	2.4	10	3.4
Metabolism	62	3.1	51	2.5	53	2.6	51	2.5	31	1.5	4	0.2	45	2.2	51	2.5
Vesicle-mediated transport	22	3.0	17	2.3	18	2.5	14	1.9	14	1.9	4	0.5	18	2.5	17	2.3
Transmembrane transport of small molecules	15	2.2	19	2.8	17	2.5	20	3.0	11	1.6	0	0.0	17	2.5	15	2.2
Signal Transduction	49	1.9	43	1.7	40	1.6	37	1.5	26	1.0	4	0.2	34	1.3	39	1.5
Immune System	36	1.9	31	1.6	40	2.1	32	1.7	22	1.2	3	0.2	31	1.6	37	1.9
Extracellular matrix organization	5	1.7	6	2.0	5	1.7	8	2.7	5	1.7	1	0.3	4	1.4	6	2.0

Count is the number of proteins whose abundance significantly changed in Ad5 or dl1520 infected A549 cells compared to uninfected A549 cells.
Percent of total is shown for the percentage of the affected protein numbers (count) in the total numbers of proteins allocated to each of the functional classes, according to the Reactome database. The highest three percentage values are highlighted in orange in each time point.

Supplementary Table S1: Proteins identified in this study. Spreadsheet of TMT time-course data for the identified proteins. For each protein the table lists UniProt accession, the corresponding protein name and description, and the relative abundance ratio. The relative abundance ratio is shown for wild-type (Ad5) or dl1520 adenovirus infected cells compared to uninfected cells over time. The data were quantified using Proteome Discoverer software v1.4 (Thermo Scientific) and searched against a combined database consisting of the UniProt Human database plus adenovirus protein sequences using the SEQUEST algorithm.

Supplementary Table S2: Reactome biological pathway analysis of affected proteins. Cellular proteins having significantly changed abundance following Ad5 and dl1520 (E1B-55k deleted) infection were analysed using the Reactome database to classify each protein by biological pathway. The table lists the numbers and percentages of affected proteins with the allocated Reactome pathway names to reflect how infection-related protein abundance changes target each represented biological pathway.
